# Targeting Oxalate Production by Combining Enzyme Inhibition and Proteolysis Activation: A Novel Therapeutic Approach for Primary Hyperoxaluria Type 1

**DOI:** 10.1021/acs.jmedchem.5c02055

**Published:** 2026-01-02

**Authors:** Fabio Arias, Sumati Rohilla, Yudibeth Sixto-López, Koral S. E. Richard, Sandeep Das, Sumit K. Anand, Pilar Maria Luque-Navarro, Guillermo Bañuelos-Sanchez, Juan Luis Pacheco-García, Reethika Gade, M. Peyton McKinney, Dhananjay Kumar, Jemiah Maxie, W. Rylan Corr, Nilesh Pandey, Harpreet Kaur, Jibin Ding, Lin Tan, Elisha Scott, Hyung Nam, Eyal Gottlieb, A. Wayne Orr, Nirav Dhanesha, Arif Yurdagul, Angel L. Pey, Francisco Franco-Montalbán, José A. Gómez Vidal, Oren Rom, Mónica Díaz-Gavilán

**Affiliations:** † Departamento de Química Farmacéutica y Orgánica, Facultad de Farmacia, 16741 Universidad de Granada, Campus Cartuja s/n, 18071 Granada, Spain; ‡ Unidad de Excelencia en Química Aplicada a Biomedicina y Medioambiente, Universidad de Granada, Av. Fuentenueva s/n, 18071 Granada, Spain; § Department of Pathology and Translational Pathobiology, 23346 Louisiana State University Health Sciences Center-Shreveport, Shreveport, Louisiana 71103, United States; ∥ Departamento de Química Física e Instituto de Biotecnología, Universidad de Granada, Av. Fuentenueva s/n, 18071 Granada, Spain; ⊥ Department of Molecular and Cellular Physiology, Louisiana State University Health Sciences Center-Shreveport, Shreveport, Louisiana 71103, United States; # Metabolomics Core Facility, Department of Bioinformatics and Computational Biology, 4002 The University of Texas MD Anderson Cancer Center, Houston, Texas 77030, United States

## Abstract

Primary hyperoxaluria type 1 (PH1) is a rare genetic disorder caused by hepatic oxalate overproduction due to alanine-glyoxylate aminotransferase (AGXT) deficiency. Therapeutic strategies targeting glycolate oxidase (GO) and lactate dehydrogenase A (LDHA), key enzymes in glyoxylate metabolism, have shown promise in reducing oxalate burden. However, recently approved siRNA therapies remain limited by high cost, unfavorable pharmacokinetics, and limited global accessibility. We report the development of compound **2**, a dual GO/LDHA inhibitor (*K*_i_ = 390 and 40 nM, respectively) that also promotes hydrophobic tag-mediated autophagic degradation of LDHA. Its efficacy was evaluated in *Agxt* ^–/–^ mice, both in primary hepatocytes and through oral administration. Treatment significantly reduced hepatic LDHA levels, urinary oxalate excretion, and renal calcium-oxalate crystal deposition. These findings support compound **2** as a first-in-class, orally bioavailable small molecule with dual inhibitory and proteolytic activity, offering a novel therapeutic candidate for PH1 and other oxalate-related pathologies.

## Graphical abstract

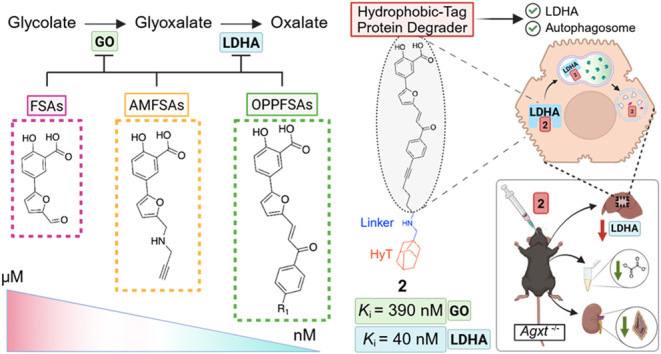

## Introduction

Primary hyperoxaluria type 1 (PH1) is a rare autosomal recessive disorder characterized by the excessive hepatic production of oxalate, which is formed from glyoxylate when its normal metabolic conversion to glycine is impaired (Figure 1A).^1,2^ Oxalate accumulation leads to recurrent nephrolithiasis, nephrocalcinosis, and progressive renal failure, often culminating in systemic oxalosis.^2,3^ PH1 is caused by mutations in the *AGXT* gene, which encodes alanine-glyoxylate aminotransferase (AGXT), the key hepatic enzyme that converts glyoxylate to glycine.^4^ Glycolate oxidase (GO) is a peroxisomal enzyme specifically expressed in hepatocytes that converts glycolate to glyoxylate.^5^ In the absence of AGXT activity, glyoxylate is rapidly metabolized to oxalate by lactate dehydrogenase isozyme A (LDHA).^6^ Beyond PH1,^7^ dysregulated oxalate metabolism has been implicated in more prevalent conditions such as systemic inflammation,^1^ metabolic dysfunction-associated steatotic liver disease (MASLD),^8–10^ and cardiovascular disorders.^11,12^ Thus, the pathological effects associated with elevated oxalate levels underscore the need for novel strategies to effectively reduce oxalate burden.

**Figure 1:**
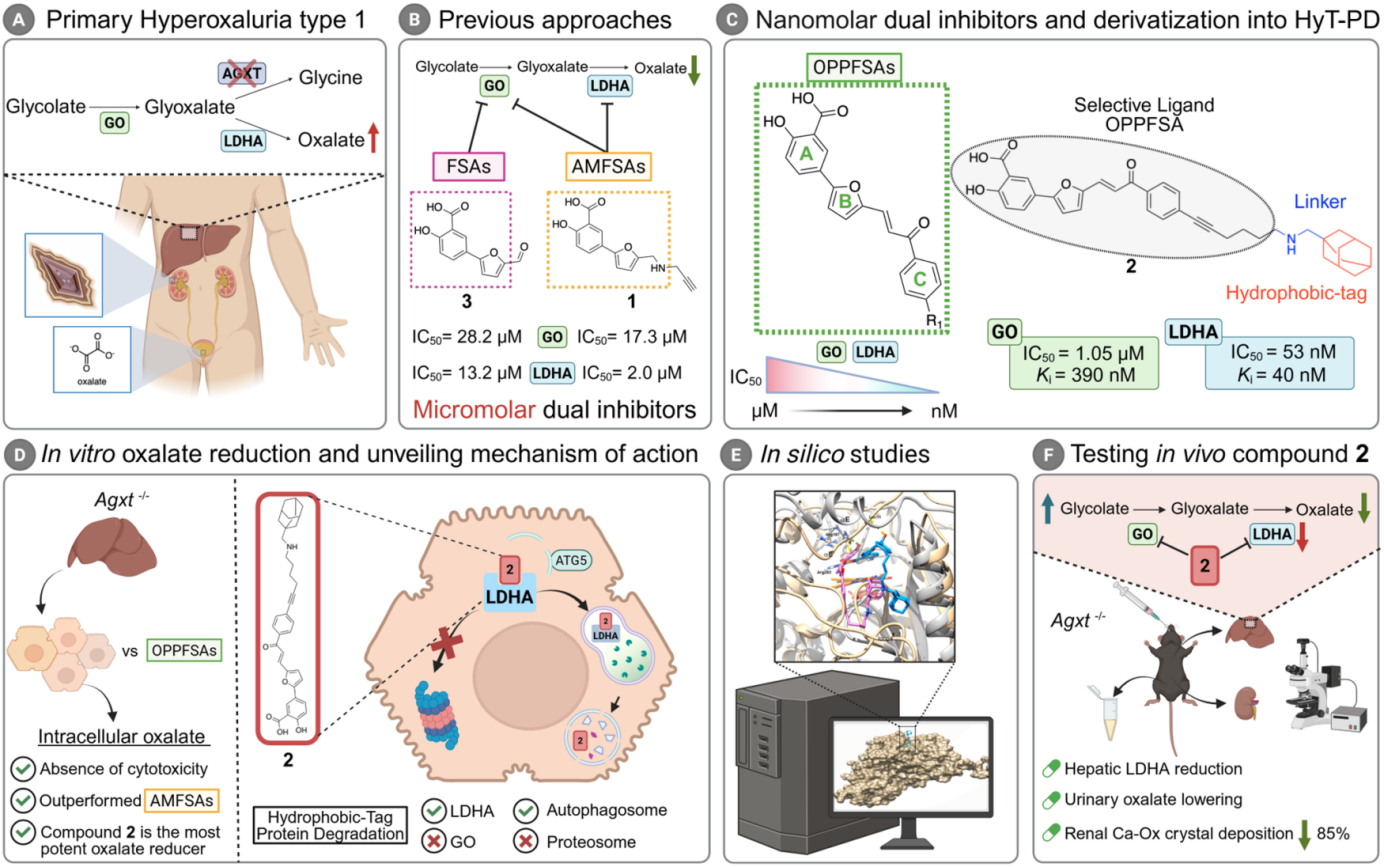
Background and summary of the study. (A) Simplified schematic of primary hyperoxaluria type 1 (PH1). (B) Overview of previous therapeutic strategies developed by our research group: furyl salicylic acids (FSAs)^32^ and aminomethyl furyl salicylic acids (AMFSAs).^31^ (C) General structure of oxophenylpropenyl furyl salicylic acids (OPPFSAs) and their derivatization into compound **2**, a hydrophobic-tag protein degrader (HyT-PD). (D) *In vitro* evaluation of the most potent inhibitors. (E) *In silico* characterization of compound-target interactions and pharmacokinetic properties. (F) *In vivo* translation of the findings.

Therapeutic strategies for PH1 have recently been transformed by RNA interference (RNAi)-based approaches. Liver-targeted small interfering RNAs (siRNAs) such as lumasiran^13^ and nedosiran^14^—targeting GO and LDHA, respectively—have received regulatory approval (EMA and FDA, 2020; FDA, 2023) and are now in clinical use.^15^ Additionally, small-molecule inhibitors such as stiripentol, a repurposed anticonvulsant with LDHA inhibitory activity have shown modest efficacy but are limited by hepatotoxicity.^16,17^ Despite this array of different strategies, no multitargeted therapy for PH1 has yet reached clinical trials.^18,19^ Moreover, the existing therapies face several notable limitations, including high cost,^20^ and limited global accessibility.^21,22^ Hence, there is a critical need for alternative therapeutic approaches that are cost-effective, pharmacologically efficient, and orally bioavailable.

Small molecules are emerging as attractive therapeutic alternatives due to their lower production cost,^23^ oral bioavailability, and favorable pharmacokinetics.^24^ Notably, dual inhibition of GO and LDHA addresses multiple upstream pathways of oxalate biosynthesis, potentially offering superior therapeutic outcomes compared to monotherapy or siRNA-based combinations.^25^ Small molecule-induced protein degradation has emerged as a transformative strategy in drug development for metabolic diseases.^26^ Unlike genetic silencing, small molecule-induced protein degradation enables selective post-translational cleavage of pathogenic proteins by hijacking the cell’s proteolytic systems, such as the ubiquitin-proteasome system^27^ or autophagy-lysosomal pathway.^28^ Among the various strategies of small molecule-induced protein degradation, hydrophobic-tag protein degraders (HyT-PDs) are constructed by linking a selective ligand (SL) for the protein of interest (PoI) to a hydrophobic tag (HyT).^29^ These bifunctional molecules induce proteolysis by mimicking misfolded proteins, effectively directing the PoI toward degradation pathways.^30^ This strategy may overcome the limitations of siRNA by using a single small molecule that selectively binds and degrades GO and LDHA.

To explore this concept, we built upon our previous findings showing that compound **1** (Figure 1B), a furyl salicylic acid (FSA), acts as a dual micromolar inhibitor of the human GO (*h*GO) and LDHA (*h*LDHA),^31^ and could serve as a starting point for the development of SL. In the present study, we report the development of 5-[5-(3-oxo-3-phenylpropenyl)-2-furyl]­salicylic acids (OPPFSAs), a novel family of dual inhibitors with enhanced potency to the nanomolar range (Figure 1C). Moreover, we introduce compound **2**, the first dual inhibitor that also promotes LDHA degradation via HyT-mediated proteolysis in the autophagy-lysosomal pathway (Figure 1D) a mechanism supported by extensive *in silico* characterization (Figure 1E).

Importantly, we demonstrate its *in vivo* efficacy through lowering urinary oxalate, resulting in a marked reduction of renal calcium-oxalate crystal deposition in a murine PH1 model (Figure 1F). These findings highlight the therapeutic potential of integrating dual enzymatic inhibition with targeted protein degradation as a novel approach for treating oxalate-related disorders.

## Results

### Design Strategy and Synthesis of OPPFSAs and Their Derivatives

Compound **3** ^32^ (Figure 1B), which features a formyl group, serves as a precursor for the design of dual *h*GO/*h*LDHA inhibitors. Our docking studies suggest that this functionality plays a nonessential role in binding to the targeted proteins. Thus, in our previous work,^31^ the chemical versatility of the formyl group was exploited to prepare *N*-substituted aminomethylfuryl salicylic acids (AMFSAs, Figure 1B) *via* reductive amination.

Although AMFSAs act as dual inhibitors, their inhibitory activity remains at the micromolar range, suggesting that further structural optimization is needed to enhance their potency. To this end, we envisioned OPPFSAs as a vinylogous series of compound **3**, incorporating α,β-unsaturated ketones and three aromatic rings, designated A-C (Figure 1C). These new functionalities are designed to modulate the physicochemical properties of the molecules and to explore novel interactions with biological targets, extending beyond the catalytic site. A family of OPPFSAs was developed by introducing various *para*-substituents on ring C, including terminal polar groups, halogens and alkyl chains (compounds **2** and **4**–**26**, Table 1). The polar functionalities at this position can influence the electronic character of ring C, enable polar interactions with the biological targets, and enhance the water solubility of the molecules. To this end, we incorporated electron withdrawing nitro and cyano groups (**5** and **6**) and the electron donating hydroxy group (**7**). Additionally, we included a benzylic alcohol (**8**), capable of acting as a hydrogen bond donor/acceptor, and aliphatic carboxylic acids (**9** and **10**) to facilitate hydrogen bonding or ionic interactions.

**Table 1:**
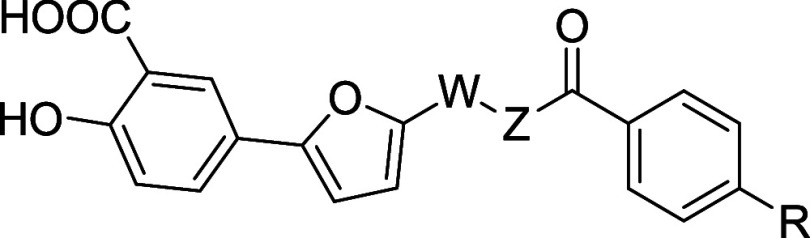
Structure of Oxophenylpropenyl Furylsacylic Acids (OPPFSAs) and Their Hydrogenated Derivatives and Total Yields of Synthesis

–W–Z–	compound	R	yield^a^ (%)/*n*
–CH=CH–	**4**	–H	58/2
	**5**	–NO_2_	49/2
	**6**	–CN	68/3
	**7**	–OH	65/2
	**8**	–CH_2_OH	66/3
	**9**	–CH=CH–CO_2_H	59/3
	**10**	–(CH_2_)_2_–CO_2_H	43/3
	**11**	–Br	32/2
	**12**	–I	77/3
	**13**	–OCH_3_	82/2
	**14**	–SCH_3_	66/3
	**15**	–N(CH_3_)_2_	72/2
	**16**	–CH_3_	72/3
	**17**	–CF_3_	62/2
	**18**	–CH_2_CH_3_	45/2
	**19**	–CH(CH_3_)_2_	71/3
	**20**	–(CH_2_)_3_CH_3_	72/3
	**21**	–(CH_2_)_5_CH_3_	48/2
	**22**	–(CH_2_)_7_CH_3_	79/2
	**23**	–C≡C-(CH_2_)_4_Cl	81/2
	**24**	–CH=CH–CO_2_C(CH_3_)_3_	64/2
	**25**	–(CH_2_)_2_–CO_2_C(CH_3_)_3_	48/2
	**26**	–CH=CH–CONH–CH_2_–adamant-1-yl	74/3
	**2**	–C≡C-(CH_2_)_4_NH–CH_2_–adamant-1-yl	49/4
-CH_2_–CH_2_–	**27**	–CF_3_	72/4
	**28**	–(CH_2_)_3_CH_3_	69/4
	**29** ^b^	–(CH_2_)_3_CH_3_	52/4

a Experimental total yields after purification.

b In compound **29** the ketone group has been reduced to an alcohol. *n* = Number of steps in the synthetic route.

To increase molecular lipophilicity and promote hydrophobic interactions with the targets, halogens (**11**-**12**) and alkyl chains (**2**, **13**-**26**) were introduced as R substituents on ring C. Halogens also exert a mild electron-withdrawing effect, whereas alkyl chains act as weak electron donors. Linear alkyl chains of up to eight carbon in length (**16**, **18**, **20**-**22**) were employed to investigate the impact of extending the molecule beyond the catalytic site, following the design rationale of previous reported GO inhibitors.^33^

Further modifications on the alkyl chains were introduced to evaluate distinct chemical properties. To improve water solubility, and assess potential polar interactions with the targeted enzymes, isosteric substitutions at the benzylic methylene were carried out, incorporating oxygen (**13**), sulfur (**14**) and nitrogen (**15**). The isosteric replacement of a methyl group (**16**) with a trifluoromethyl group (**17**) aimed to probe electronic effects. Branching was explored using the *N*,*N*-dimethylamino group (**15**) and the isopropyl group (**19**). Unsaturations in compounds **23** and **24** were designed to reduce the rotational freedom of the side chain while increasing the electronic density beyond ring C, potentially enabling π-type interactions with the target enzymes. Finally, the *tert*-butyl carboxylates **24** and **25**, which serve as precursors to carboxylic acids **9** and **10**, respectively, were also included in the screening against recombinant enzymes.

Importantly, compounds **2** and **26** were specifically designed as a HyT-PDs, incorporating adamantane HyT moieties linked *via* a six- or three-carbon spacer to the OPPFSA warhead, respectively. This feature was intended to promote target degradation through hydrophobic tagging.

In OPPFSAs, the α,β-unsaturated ketone located between rings B and C contributes to a planar, highly conjugated structure. To explore structure–activity relationships, we synthesized a small set of hydrogenated derivatives of OPPFSAs (**27**-**29**, Table 1). The removal of this double bond increases the rotational freedom between rings B and C, disrupts the conjugation between the FSA core and ring C, and eliminates the coplanarity among the three aromatic rings. These modifications potentially enhance the molecule’s adaptability to the binding sites of both targets.

Additionally, structurally related analogues that deviate from the canonical OPPFSA scaffold (“*other structures*”) were designed by (i) replacing ring C with alkyl chains; (ii) substituting ring C with heteroaromatic rings such as pyrrole and furan; and (iii) fully hydrogenating ring B and the α,β-unsaturated ketone moiety (Figure S1 and Section S1).

A versatile synthetic route to OPPFSAs (**4–25**, Scheme 1) was developed, involving two to three steps (steps a-c) with total yields ranging between 32 and 82% (Table 1). The initial Suzuki-Miyaura coupling of methyl 5-iodosalicylate (**30**) and 5-formylfuran-2-boronic acid (**31**) yielded the ester-protected aldol substrate **32** (step a). Subsequent aldol condensation with selected acetophenones (**33**) afforded α,β-unsaturated ketones type **34** (step b). In some cases, this step also induced simultaneous hydrolysis of the methyl ester, directly yielding the final acidic OPPFSA. When hydrolysis did not occur concurrently, it was achieved by refluxing in pyridine (step c).

**Scheme 1:**
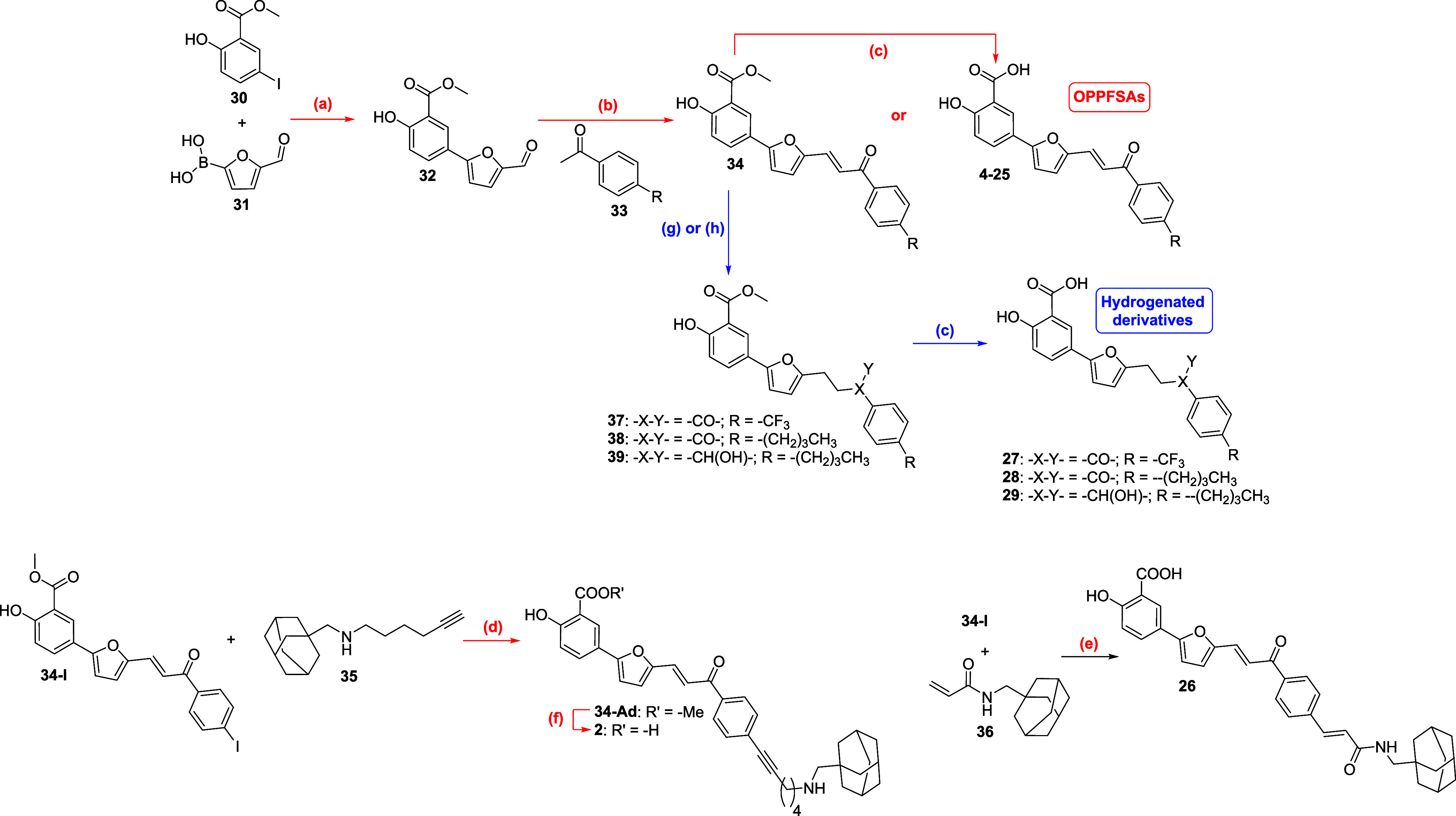
Synthesis of Oxophenylpropenylfuryl Salicylic Acids (OPPFSAs) and Hydrogenated Derivatives^a^

a Conditions: (a) NEt_3_, Pd­(OAc)_2_, DMF, rt (yield 87%). (b) (i) NaOH, THF/MeOH/H_2_O, rt; (ii) HCl (yields 43–96%). (c) (i) Py, reflux; (ii) HCl 1N (yields 85–97%); (d) (Ph_3_P)_2_PdCl_2_, CuI, TBAF/hexane 1M, THF (anhydrous), Ar, rt, 18 h (yield 67%). (e) Pd­(OAc)_2_, DABCO, K_2_CO_3_, DMF (anhydrous), 120 °C, 12 h (91%). (f) (i) NaOH, MeOH/H_2_O (3:1), 40 °C, 12 h; (ii) HCl 1N (yield 90%). (g) H_2_, Raney Nickel, THF, rt (yield 95%). (h) H_2_, Pd/C, THF, rt (yield 70%).

The synthesis of compounds **2** and **26** required the preparation of the corresponding adamantane-functionalized terminal alkyne (**35**) or alkene (**36**), respectively. These intermediates were coupled with halogenated precursor **34**-I *via* a Sonogashira (step d) or Heck reaction (step e). While the coupling directly yielded the final acid **26**, the synthesis of compound **2** required an additional ester hydrolysis step using aqueous sodium hydroxide (step f).

To obtain hydrogenated derivatives, selective hydrogenation of the double bond or nonselective hydrogenation of both the double bond and the ketone of intermediates type **34** was carried out using Raney Nickel or palladium on charcoal catalysts, respectively (steps g and h, respectively). Following hydrolysis of the intermediate esters (step c), the final partial (**27** and **28**) or total (**29**) hydrogenated derivatives were obtained (Table 1).

### OPPFSAs Exhibit Enhanced Potency Compared to Reference Compound **1** as Dual Inhibitors of Recombinant *h*GO and *h*LDHA

Once a diverse array of final compounds was successfully synthesized, their inhibitory activity against the recombinant enzymes *h*GO and *h*LDHA was evaluated using fluorometric kinetic assays. An initial screening at 10 μM allowed us to assess inhibitory activity, compare with molecules from previous studies,^31,32^ establish preliminary structure–activity relationships, and discard low potency molecules. The relative activity of *h*GO and *h*LDHA, as shown in Figure 2A,B and Table S1 (Section S2), ranged from 0 to 84% for *h*GO and 0 to 92% for *h*LDHA. Interestingly, 21 out of the 33 newly synthesized and tested compounds exhibited greater inhibitory effect against *h*GO and *h*LDHA than the reference compound **1** at 10 μM (yellow rectangle, Figure 2A). Among them, 9 compounds inhibited both enzymes by 90% or more (red rectangle, Figure 2A). Notably, approximately half of the new compounds achieved over 80% inhibition at 10 μM for at least one of the enzymes (Figure 2A,B).

**Figure 2:**
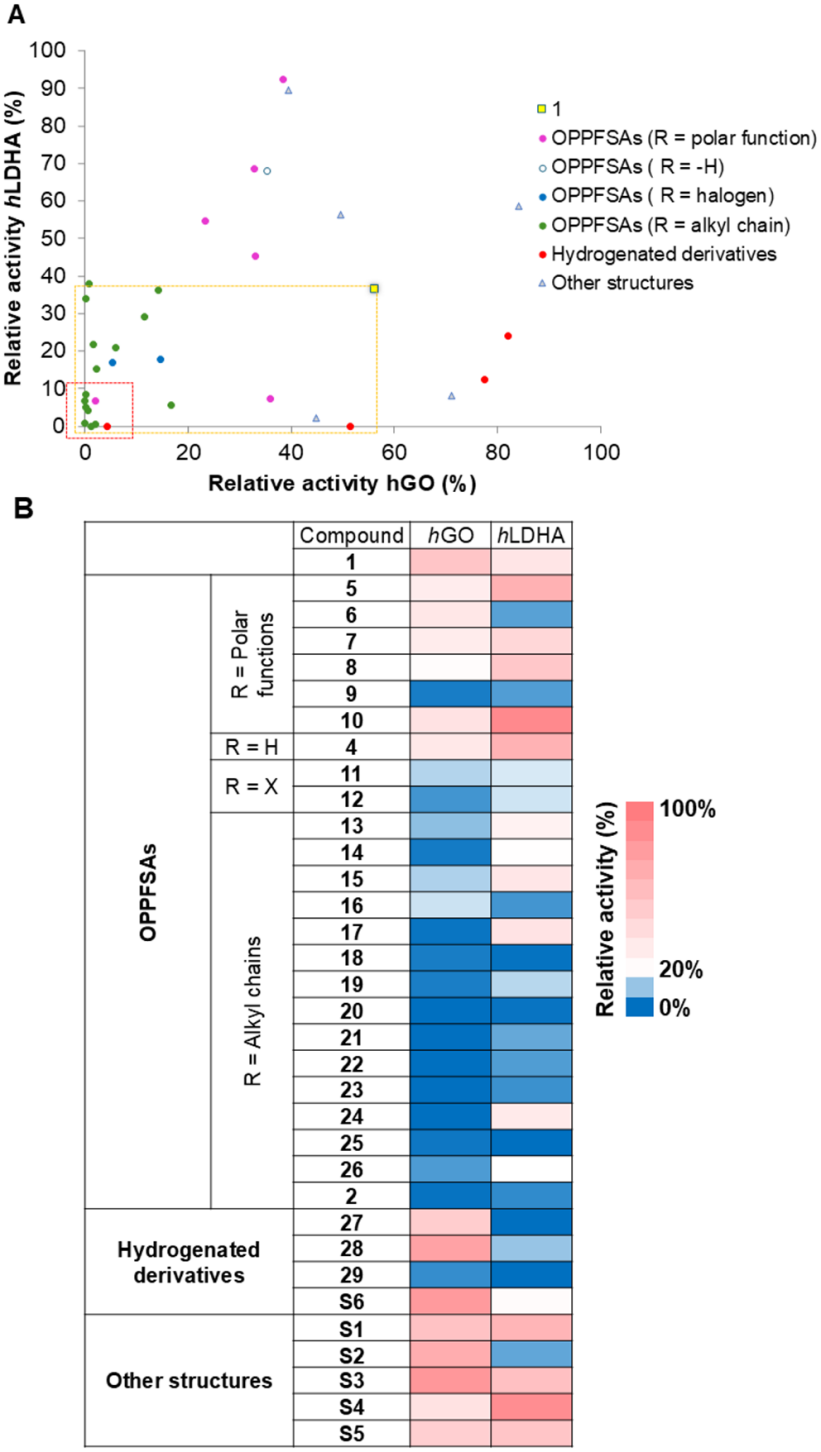
Evaluation of inhibitors (10 μM) against recombinant enzymes *h*GO and *h*LDHA. (A) Distribution of inhibitors according to the remaining activity of recombinant enzymes after treatment. Comparison with the reference compound **1**. Yellow rectangle: compounds that decrease enzyme activity in a greater extent than **1**. Red rectangle: compounds that produce more than 90% inhibition for both enzymes. (B) Heatmap of enzymatic activity of recombinant *h*GO and *h*LDHA following treatment with representative compounds. Activity values are expressed relative to vehicle treated controls (set at 100%) and represent the mean of four replicates (*n* = 4).

Among the different structural families (OPPFSAs, hydrogenated derivatives and other structures) OPPFSAs presenting alkyl chains (R = alkyl chain) were the most potent inhibitors. The presence of the phenyl ring C in the OPPFSA scaffold was found to be essential for potent inhibitory effect against both enzymes. Substitution of this ring with alkyl or heteroaryl moieties (**S1–S5**, *other structures*), resulted in a marked loss of inhibitory effect, particularly against *h*GO (Figure 2A,B).

The nature of the *para*-substituent (R) on the phenyl ring C played a critical role. A direct correlation was observed between the hydrophobic character of R^34^ and the inhibitory potency against both *h*GO and *h*LDHA (Figure 2A). Hydrophobic substituents, such as halogens (**11** and **12**) and alkyl chains (**2** and **13–26**) yielded potent dual inhibitors (Figure 2B). Linear alkyl chains up to eight carbon atoms (**16–22**) were well tolerated. Longer chains were not evaluated to avoid potential pharmacokinetic liabilities. Neither branching (*e.g.*, ^*i*^Pr in **19**) nor the presence of unsaturation (double or triple bonds in **9**, **23** and **24**) negatively impacted activity. Notably, butyl (**20**), 6-chlorohex-1-ynyl (**23**) and the bulky adamantyl-terminated hexynyl chain (**2**) conferred particularly strong inhibition.

Isosteric replacement of methylene groups in R with heteroatoms such as oxygen, sulfur (**13** and **14** *vs* **18**) or nitrogen (**15** *vs* **19**) generally reduced dual inhibitory activity, with sulfur (**14**) being the best tolerated among these. The loss of activity affecting mainly *h*LDHA inhibition, observed when the methyl group (R) in compound **16** was replaced by either an electron-withdrawing trifluoromethyl group (**17**) or an electron-donating methoxy group (**13**) supports the hypothesis that hydrophobicity, rather than electronic effects, modulate the inhibitory potency. In contrast, substituents with polar terminal groups were generally less effective, except for compound **9**, which contains a carboxyvinyl group and showed good inhibition.

The presence of the planar α,β-unsaturated ketone moiety was found to be important for *h*GO inhibition, as hydrogenation of the alkene to alkane, or of both the alkene and ketone to an alkane-alcohol, generally resulted in reduced potency (“Hydrogenated derivatives”, Figure 2A), except for compound **29** (Figure 2B).

Following the initial screening, those compounds with inhibition percentages over 30% were selected for IC_50_ determination (Table 2; Figures S2–S27 and Tables S2–S27 in Section S3; Figures S28–S52 and Tables S28–S52 in Section S4). For *h*GO, most IC_50_ values ranged between 0.8 and 5 μM, whereas for *h*LDHA, many compounds exhibited nanomolar inhibitory activity. Notably, compounds **2** [R = 6-(adamantylmethylamino)­hexin-1-inyl], **20** (R = butyl), **22** (R = octyl), and **23** (R = 6-chlorohex-1-inyl) showed IC_50_ values below 200 nM against *h*LDHA. Additionally, compound **23** demonstrated nanomolar potency against *h*GO.

**Table 2:** Summary of IC_50_ Values Obtained for Representative Compounds on Recombinant Glycolate Oxidase and Lactate Dehydrogenase A^a^ ^,^ ^b^

compound	IC_50_ (*h*GO)	IC_50_ (*h*LDHA)	compound	IC_50_ (*h*GO)	IC_50_ (*h*LDHA)
**1**	17.3 ± 3.5	2.0 ± 0.1	**18**	3.6 ± 0.2^b^	1.4 ± 0.6
**2**	1.1 ± 0.1	0.05 ± 0.004	**19**	2.5 ± 0.1	0.29 ± 0.06
**4**	10.6 ± 0.7	ND	**20**	1.8 ± 0.1	0.07 ± 0.02
**5**	7.9 ± 1.4	ND	**21**	1.9 ± 0.1	0.45 ± 0.21
**6**	ND	1.3 ± 0.2	**22**	1.2 ± 0.02	0.08 ± 0.03
**7**	ND	4.0 ± 1.1	**23**	0.78 ± 0.06	0.09 ± 0.02
**8**	3.9 ± 0.6	ND	**24**	2.4 ± 0.3	0.36 ± 0.05
**9**	1.0 ± 0.1	3.4 ± 1.2	**25**	2.3 ± 0.3	0.75 ± 0.11
**10**	5.8 ± 0.4	ND	**26**	3.2 ± 0.2	0.61 ± 0.02
**11**	2.5 ± 0.5	1.5 ± 0.4	**27**	11.4 ± 0.7	0.40 ± 0.16
**12**	1.9 ± 0.2	3.2 ± 1.0	**28**	6.2 ± 0.2	4.0 ± 0.1
**13**	4.4 ± 0.2	6.1 ± 1.2	**29**	3.2 ± 0.01	0.82 ± 0.19
**14**	1.8 ± 0.2	3.7 ± 0.4	**S1**	19.0 ± 1.3	ND
**15**	3.2 ± 0.1	1.0 ± 0.3	**S2**	ND	3.6 ± 2.0
**16**	2.3 ± 0.3	1.5 ± 0.6	**S6**	ND	4.8 ± 1.7
**17**	4.2 ± 0.3	4.7 ± 0.5			

a Comparison with the reference compound **1**.

b Data are expressed as IC_50_ ± SD (μM). ND: IC_50_ not determined as the inhibition percentage was lower than 30% at 10 μM in the initial screening. IC_50_ values were determined as a mean of four replicates with ten concentrations of inhibitor each (*h*GO concentration, 25 nM; *h*LDHA concentration, 0.05 units/mL; glycolate concentration, 180 μM; pyruvate concentration, 1 mM). Sections S3–S4 for semi logarithmic plots.

It is worth highlighting that compounds **2** and **26** were specifically designed based on compounds **23** and **9**, respectively. In the first case, the terminal chlorine atom of **23** was replaced with an adamantanemethylamino moiety; in the second, the terminal carboxylic group of compound **9** was modified *via* amidation with the same adamantanemethylamino group.

This design strategy was rationalized after compounds **9** and **23** were identified as potent dual inhibitors (in the case of **23**, the most active within the series) and as chemically versatile scaffolds, amenable to structural modifications on the terminal positions of their side chains. The rigid three-carbon side chain in compound **9** and the flexible six-carbon chain in compound **23** were thus repurposed as linkers to incorporate the HyT adamantane moiety, aiming to generate potential HyT-PDs.

Despite the difference in bulkiness between chlorine (in **23**) and adamantane (in **2**), this substitution did not alter the hydrophobic character of this region of the molecule. The incorporation of the adamantane HyT moiety in **2** and **26** preserved the affinity for both *h*GO and *h*LDHA. Compound **2** exhibited excellent IC_50_ values of 1.05 μM and 53 nM, respectively.

### OPPFSAs Reduce Extracellular and Intracellular Oxalate in PH1 Mouse Primary Hepatocytes, with Compound **2** Achieving Oxalate Levels below Baseline

Encouraged by the results obtained in recombinant enzymes, we next evaluated the compounds in a more physiologically relevant model: primary hepatocytes isolated from *Agxt* ^–/–^ mice (Figure S53).^8^ To induce oxalate overproduction, cells were stimulated with 5 mM glycolic acid (GA), and the compounds’ ability to reduce oxalate production was assessed.^31^ In our previous study, compound **1** reduced extracellular oxalate to undetectable levels at a concentration of 10 μM indicating strong activity and establishing the benchmark for subsequent comparisons.^31^

Based on the IC_50_ results (Table 2), the top 18 compounds that outperformed compound **1** were selected for the *in vitro* cellular experiments. To rule out cytotoxic effects, we assessed cell viability in primary hepatocytes from *Agxt* ^–/–^ mice using the CellTiter-Blue Assay. No evidence of cell death was observed for any of the compounds at 50 μM (Figure S54). Next, *Agxt* ^–/–^ primary hepatocytes were loaded with GA (5 mM) and either treated with vehicle (DMSO) or the selected inhibitors at 10 and 50 μM. To set a proper baseline, each set of cells had a control without GA stimulation. After 24 h, extracellular oxalate levels were quantified to assess the extent of reduction achieved by each compound as fold change relative to GA loading (Figure S55). Notably, all tested OPPFSAs significantly decreased extracellular oxalate concentration to levels comparable to compound **1** and without significant differences among them.

Since extracellular measurements may overestimate efficacy, we next examined intracellular oxalate levels. Under identical conditions, compound **1** failed to significantly reduce intracellular oxalate compared to GA-stimulated controls, with a substantial decrease to basal levels detected only at 50 μM (Figure 3). At 50 μM, statistically significant reductions in intracellular oxalate levels were observed only for compounds **1**, **2**, **16**, **21**, and **26** with no significant differences detected among them. To draw more definitive conclusions, further evaluation was conducted at 10 μM. In contrast to compound **1**, several OPPFSAs were able to significantly reduce intracellular oxalate levels at this lower concentration. Notably, significant reductions with respect to GA (*) and cells treated with compound **1** (^@^) were observed with the alkyl-substituted derivatives **16** (R = -Me), **19** (R = -^*i*^Pr), **21** (R = -hexyl) and the adamantyl-terminated derivative **2**. Among these, compound **2** exhibited the most pronounced effect, reducing intracellular oxalate to levels well below the physiological baseline observed in untreated control cells (Figure 3). These results underscore the importance of directly quantifying intracellular oxalate as a more accurate and sensitive indicator of metabolic inhibition.

**Figure 3:**
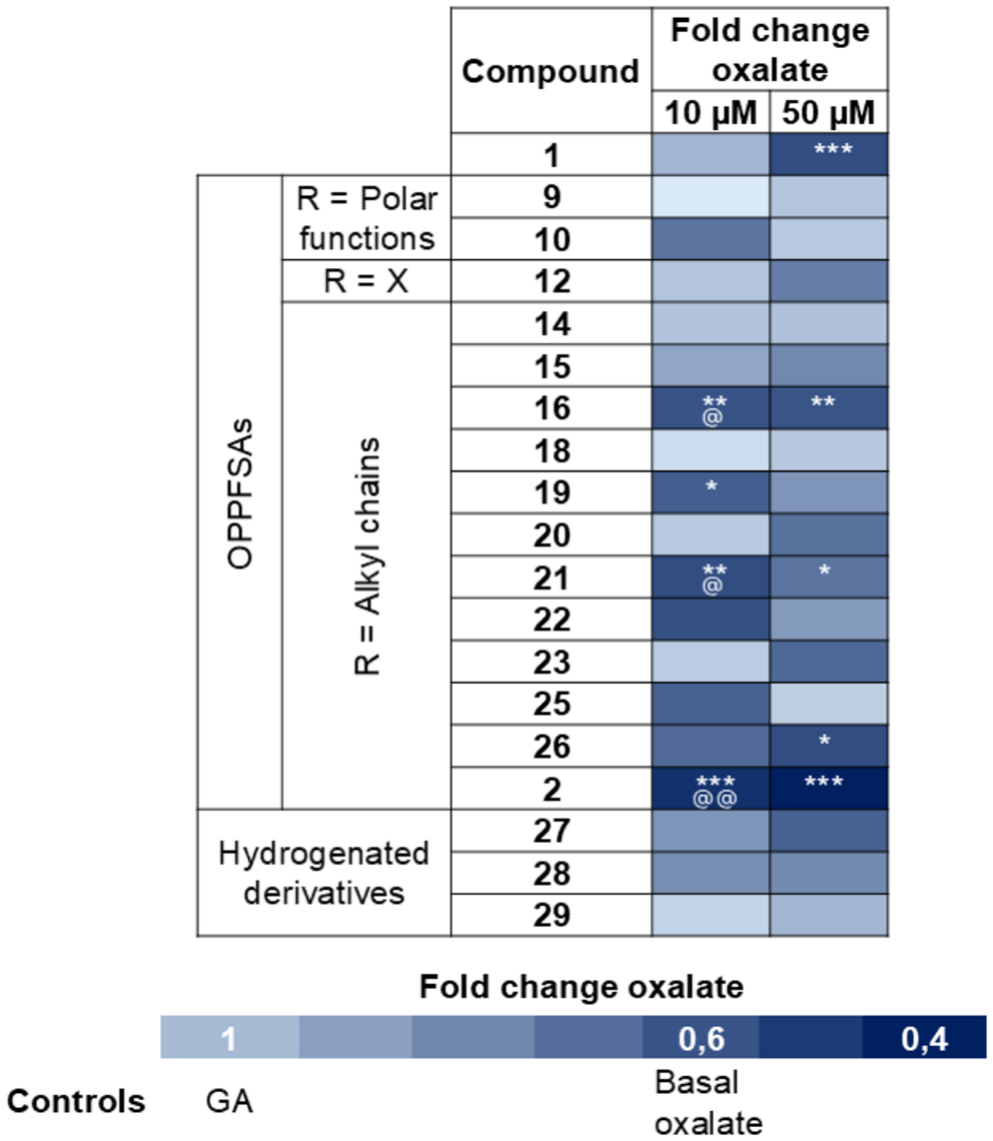
Heatmap of intracellular oxalate levels (fold change) in primary hepatocytes from *Agxt* ^–/–^ mice following treatment with dual inhibitors. *Agxt* ^–/–^ primary hepatocytes were isolated, incubated with glycolic acid (GA, 5 mM) and treated with either vehicle (DMSO) or inhibitors at 10 μM and 50 μM for 24 h (*n* = 4). Statistical analysis: Comparisons between GA control and inhibitor-treated groups were performed using one-way ANOVA followed by Tukey’s post hoc test. Significance levels (*p < 0.05; **p < 0.01; ***p < 0.001). Additional comparisons between compounds that significantly reduced oxalate and reference compound **1**, were analyzed using one-way ANOVA followed by Tukey’s post hoc test. Significance levels (^@^ *p* < 0.05; ^@@^ *p* < 0.01).

While many members of the OPPFSA family showed robust inhibition *in vitro* and effectively reduced extracellular oxalate, their translation to intracellular assays revealed substantial variability. One possible explanation lies in differences in solubility and membrane permeability, two key factors influencing cellular uptake and intracellular target availability.

To explore this, we used Molecular Operative Environment (MOE) to calculate physicochemical parameters related to bioavailability: *C* log *P* (octanol/water partition coefficient) and *h* log *D* (distribution coefficient at pH 7.4). The *C* log *P* values for the tested OPPFSAs ranged from 4.0 to 8.2, with compound **2** showing the highest lipophilicity. Corresponding *h* log *D* values ranged between 3.8 to 8.1. Compounds **16** and **19** showed *h* log *D* values of 4.7 and 5.7, respectively, while **20** and **23** exhibited higher values (6.21 and 6.25), suggesting that subtle differences in partitioning behavior may underline the observed variability in cellular performance.

Despite generally similar *h* log *D*/*C* log *P* ratios across the family, compound **2** displayed the lowest ratio (0.6), due to an *h* log *D* value of 5.2, indicating a distinct and more favorable distribution behavior under physiological conditions (Figure S56).

Although these predictions are limited by the *in silico* nature of the analyses, the findings suggest that solubility and partitioning properties may contribute to the divergent cellular effects observed among OPPFSA analogs and should be considered when translating *in vitro* potency to complex biological models.

### Compound **2** is a Noncompetitive Inhibitor of *h*GO and *h*LDHA

The favorable phenotypic effect observed for compound **2** in PH1 primary hepatocytes prompted further characterization of its biological profile. Thus, inhibition kinetics of compound **2** was carried out to determine the affinity constant of the inhibitor (*K*_i_) and its mechanism of inhibition in competence with the enzymatic substrates, GA for *h*GO, and pyruvic acid (PA) for *h*LDHA (Table 3). For comparison, compounds **20** and **23**, the most potent dual inhibitors based on IC_50_ values (Table 2), were also analyzed. The enzymatic activity of recombinant *h*GO and *h*LDHA was measured in the presence of four/five different inhibitor concentrations and ten different substrate concentrations. As a result, *K*_i_ values of the inhibitors ranged from 270 to 400 nM for *h*GO and from 26 to 90 nM for *h*LDHA (Table 3).

**Table 3:** *K*_i_ Values Obtained for Selected Compounds on Recombinant Human Glycolate Oxidase (*h*GO) and Lactate Dehydrogenase Enzymes (*h*LDHA)^a^

compound	*K*_i_	IC 95%	α	enzyme
**1** ^31^	24.74	18.32–31–22	5.2	*h*GO
**2**	0.39	0.16–0,62	2.4	
**20**	0.27	0.11–0.42	5.8	
**23**	0.27	0.12–0,41	1.6	
**1**	6.14	3.66–8.62	1	*h*LDHA
**2**	0.04	0.03–0.05	1	
**20**	0.09	0.08–0.11	1	
**23**	0.03^b^	0.02–0.04		

a Units: μM. IC 95%: 95% confidence interval. *K*_i_ obtained in experiments using ten different substrate concentrations and four/five inhibitor concentrations each (*n* = 3). Values of α = 1 indicate pure noncompetitive inhibition; values of α > 1 indicate noncompetitive mixed type inhibition (mixed competitive).

b Competitive inhibitor.

These values represent an increase of 63 to 92-fold in the affinity for *h*GO and 68 to 205-fold in the affinity for *h*LDHA, with respect to **1**.^31^ The three compounds, **2**, **20** and **23**, like **1**,^31^ behave as noncompetitive inhibitors of *h*GO (α > 1, mixed competitive).^35^ In the case of *h*LDHA, compounds **2** and **20** exhibit a pure noncompetitive inhibition profile (α = 1) with respect to the substrate pyruvate, similar to compound **1**.^31^ In contrast, compound **23** is a pyruvate-competitive inhibitor of *h*LDHA with a *K*_*i*_ value of 26 nM (data and linear/nonlinear plots of all the studied compounds are presented in Sections S6 (Tables S53–S61 and Figures S57–S62) and S7 (Tables S63–S74 and Figures S64–S69).

Given the low nanomolar *K*_i_ values obtained for compounds **2**, **20** and **23** against recombinant *h*LDHA, and *h*GO, potential enzyme titration effects were evaluated.^36^ Morrison *K*_i_ values and their ratio to enzyme concentration indicate that, although some experiments were conducted within the tight-binding regime, they remained outside the enzyme titration region (Tables S62 and S75, and Figures S63 and S70). Therefore, enzyme titration did not significantly influence the kinetic parameters determined in this study.

To assess the selectivity of compound **2** among LDH isoforms we further characterized its inhibitory behavior against recombinant *h*LDHB. Compound **2** was found to be a pure noncompetitive inhibitor (α = 1) of *h*LDHB, with an IC_50_ value of 0.24 ± 0.01 μM and a *K*_i_ of 1.7 μM. These results indicate a modest 42-fold selectivity of compound **2** for *h*LDHA over *h*LDHB, based on *K*_i_ comparison (data and linear and nonlinear plots in Tables S76–S80 and Figures S71–S73 in Section S8).

Collectively, these results demonstrate that OPPFSAs exhibit a markedly improved inhibitory profile compared to their AMFSA and FSA predecessors; however, they do not fully account for the exceptional intracellular oxalate reducing activity observed in compound **2**.

### Compound **2** Induces LDHA Degradation *via* the Autophagy–Lysosomal Pathway in PH1 Mouse Hepatocytes

Compound **2** was intentionally designed to include an adamantane moiety aiming to promote target proteolysis as a HyT-PD. To evaluate whether this mechanism contributes to the compound’s potent intracellular oxalate-lowering activity in PH1 primary hepatocytes, we treated hepatocytes isolated from *Agxt* ^–/–^ mice with increasing concentrations of compound **2** (2, 10, 20, and 50 μM). While GO protein levels remained unchanged (Figure 4A), LDHA abundance was significantly reduced at 50 μM compared to vehicle-treated controls (Figure 4B). As expected, we observed no significant differences in the mRNA expression of *Ldha* or *Hao1* (which encodes GO) at any concentration (Figure 4C,D). These results indicate that compound **2** induces LDHA degradation in *Agxt* ^–/–^ mouse primary hepatocytes.

**Figure 4:**
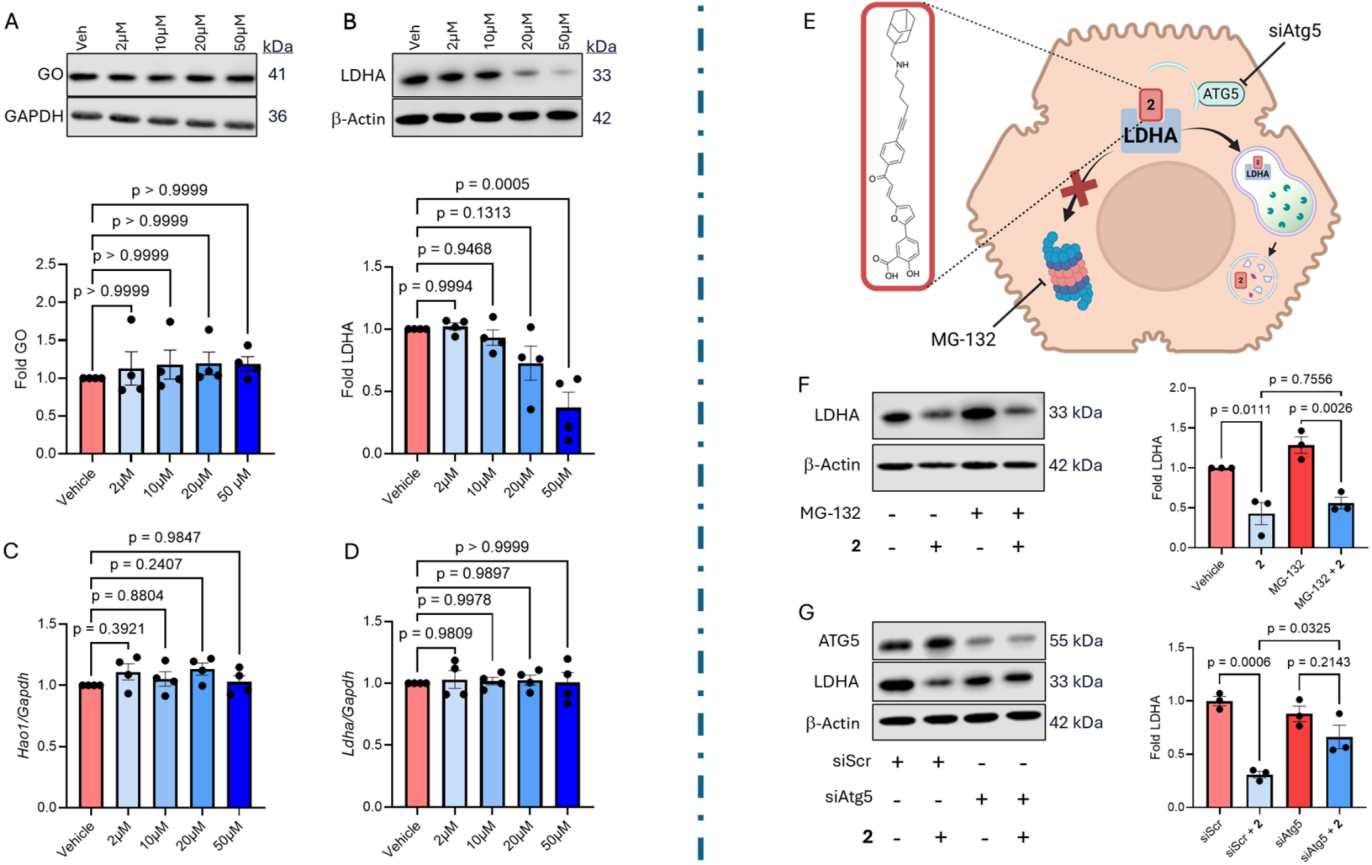
Mechanism of action of compound **2**. (A–D). *Agxt* ^–/–^ primary hepatocytes were isolated and treated with either vehicle (DMSO) or increasing concentrations of **2** (2, 10, 20, and 50 μM) for 24 h. (A) GO protein abundance and quantification relative to GAPDH (*n* = 4). (B) LDHA protein abundance and quantification relative to β-Actin (*n* = 4). (C) Relative *Hao1* mRNA expression (*n* = 4). (D) Relative *Ldha* mRNA expression (*n* = 4). (E–G) Determination of the degradation mechanism of LDHA mediated by **2**. (E) Representation of possible LDHA degradation mechanisms and strategy of study. (F) LDHA protein abundance and quantification relative to β-Actin (*n* = 3) in the presence or absence of compound **2** (50 μM) and the proteasome inhibitor MG-132 (10 μM). (G) LDHA and ATG5 protein abundance and quantification relative to β-Actin (*n* = 3) in the presence or absence of **2** (50 μM), siRNA against the autophagy mediator *Atg5* or control scramble siRNA. Data are expressed as mean ± SEM. Normality was assessed using the Shapiro-Wilk test. Comparisons among multiple groups that passed normality testing were analyzed using one-way ANOVA followed by Tukey’s post hoc test. Groups that did not meet normality assumptions were analyzed using the Kruskal–Wallis test.

Having confirmed that compound **2** can activate LDHA proteolysis, we next examined whether compound **26**, which also bears an adamantane tag, could trigger the same response. Despite its relatively potent dual inhibitory activity (Table 2) and significant intracellular oxalate reduction at 50 μM (Figure 3), compound **26** did not decrease GO or LDHA protein levels (Figure S74, Section S9).

Given that compound **2** is the only member of the OPPFSA family promoting LDHA degradation, we next sought to elucidate the underlying proteolytic mechanism. Previous reports suggest that HyT-PDs can activate one or both of the major proteolytic systems, the ubiquitin-proteasome system and the autophagy-lysosomal pathway.^29,30^ We thus investigated the involvement of these pathways in the degradation of LDHA upon binding to compound **2** (Figure 4E). To this end, we assessed LDHA protein abundance following treatment with **2** in the presence or absence of pathway-specific inhibitors. First, we cotreated cells with compound **2** and MG-132, a well-characterized proteasome inhibitor.^37^ No significant increase in LDHA protein levels was observed in cells treated with **2** and MG-132, compared to cells treated with compound **2** alone, therefore ruling out proteasome involvement in this process (Figure 4F).

To determine whether compound **2** induces LDHA degradation *via* the autophagy-lysosomal pathway, we silenced autophagy-related gene 5 (*Atg5*) using siRNA (siAtg5). The encoded protein, ATG5, is essential for autophagosome formation and has previously been shown to be required for the action of compounds that engage this pathway.^38^ Transfection with siAtg5 reduced ATG5 expression by approximately 70% (Figure S75). Under these conditions, the reduction in LDHA protein levels induced by compound **2** was abolished, and LDHA protein levels were significantly higher than in cells treated with the scrambled siRNA (siScr) (Figure 4G). These results indicate that compound **2** promotes LDHA degradation *via* an autophagy-dependent lysosomal pathway.

### *In Silico* Analysis of Compound **2** Reveals Enzyme-Specific Differences in Binding Dynamics and Spatial Disposition

To investigate the molecular basis for the differential HyT-PD activity of **2**, and study its binding to *h*GO and *h*LDHA, we performed atomistic molecular dynamics (MD) simulation. Previous to MD simulation, docking poses of **2** were obtained on the crystal structures of *h*LDHA isozyme form (PDB ID: 1I10)^39^ and *h*GO (PDB ID: 2RDT)^33^ and are displayed in Figures S76–S78 (Section S11). The docking pose of **2** on *h*GO served as a template to construct a homology model of *h*GO. The docking pose of **2** on *h*LDHA (subunit B) was used on MOE to build a dimeric complex with subunit A of *h*LDHA containing cofactor 1,4-dihydronicotinamide adenine dinucleotide (PDB ID: NAI) and oxamic acid (PDB ID: OXM) inhibitor. The *h*GO homology model and the *h*LDHA dimeric complex were later corrected and prepared using MOE QuickPrep module for further MD analysis (*vide infra*). The stability of protein–ligand complexes during MD simulations was assessed using Root Mean Square Deviation (RMSD) analysis and validated by a Solvent Accessible Surface Area (SASA) analysis in a time frame of 100 ns (Figures S79–S88, Section S12).

Docked poses of **2** on *h*GO and hLDHA show the introduction of its salicylic acid warhead into the catalytic sites of the enzymes, interacting with key substrate-binding and catalytic residues (detailed binding interactions are presented in Section S11). Given the size of **2**, the furan ring and side chain are displayed toward the hydrophobic access channel of *h*GO and, in the case of *h*LDHA, toward the cofactor hydrophobic cleft. On *h*GO, the furan ring shows a π-stacking interaction with the key residue Trp110. On *h*LDHA the intermediate carbonylic group in **2** H-bonds the backbone chain of Ala29 at the entrance of the catalytic site pocket, as does the NAD^+^ cofactor through one of its oxygen-phosphate groups. In the case of *h*GO, the adamantane is displayed toward a hydrophobic cleft set by residues Ile115, Leu143, Val139 and Tyr134. This region has been identified as an allosteric binding site for novel *h*GO inhibitors.^40^ The adamantane orientation is also helped by a H-bond interaction established between the backbone chain of Ala111 and the protonated methylamino moiety of **2**. In the case of *h*LDHA, the adamantane methylamino moiety is displayed toward the end of the NAD^+^ cofactor cleft, with the adamantane group on the hydrophobic region set by Ile115, Val52, Ile119, and Phe118. *In silico* docking values of free energy of binding (ΔG) of **2** in *h*GO (PDB ID: 2RDT) and *h*LDHA are −13.15 and −11.93 kcal/mol, respectively.

The MD simulation initiates with a homology model of *h*GO, using the docked pose of **2** on PDB ID 2RDT as template. RMDS cluster analysis on the **2**-***h*** **GO** ensemble after the simulation resulted in the most populated cluster-representative structure depicted in Figures 5A,C and S83 (blue). As shown, the salicylic moiety of ligand **2** remains inside the catalytic pocket establishing two H-bonds with catalytically important residue Arg263 through its carboxylic acid. Compared to frame 1 (*t* = 0, Figure 5A,C, pink), the ligand shifts toward the α3 helix, which is displaced outward. This displacement affects several catalytic site residues, including Trp110 and His260, which are repositioned away from the catalytic site. Additionally, a third H-bond is observed between the carbonyl group of **2** and the backbone of Lys211 (Figure 5A). This interaction seems to induce a distortion in the αE helix, which is essential for preserving the structural integrity of the catalytic site. The novel ligand-protein conformation is partially driven by the adamantane moiety’s propensity to occupy the hydrophobic cleft formed by residues Ile115, Leu143, Val139, and Tyr134. Furthermore, it establishes new interactions with loop 4 residues, including Phe186 and Gly187, thereby promoting the closed loop conformation. However, this inward orientation of the adamantane moiety (Figure 5C, blue) is unfavorable for inducing protein degradation supporting the absence of *h*GO degradation activity observed in the presence of ligand **2**

**Figure 5:**
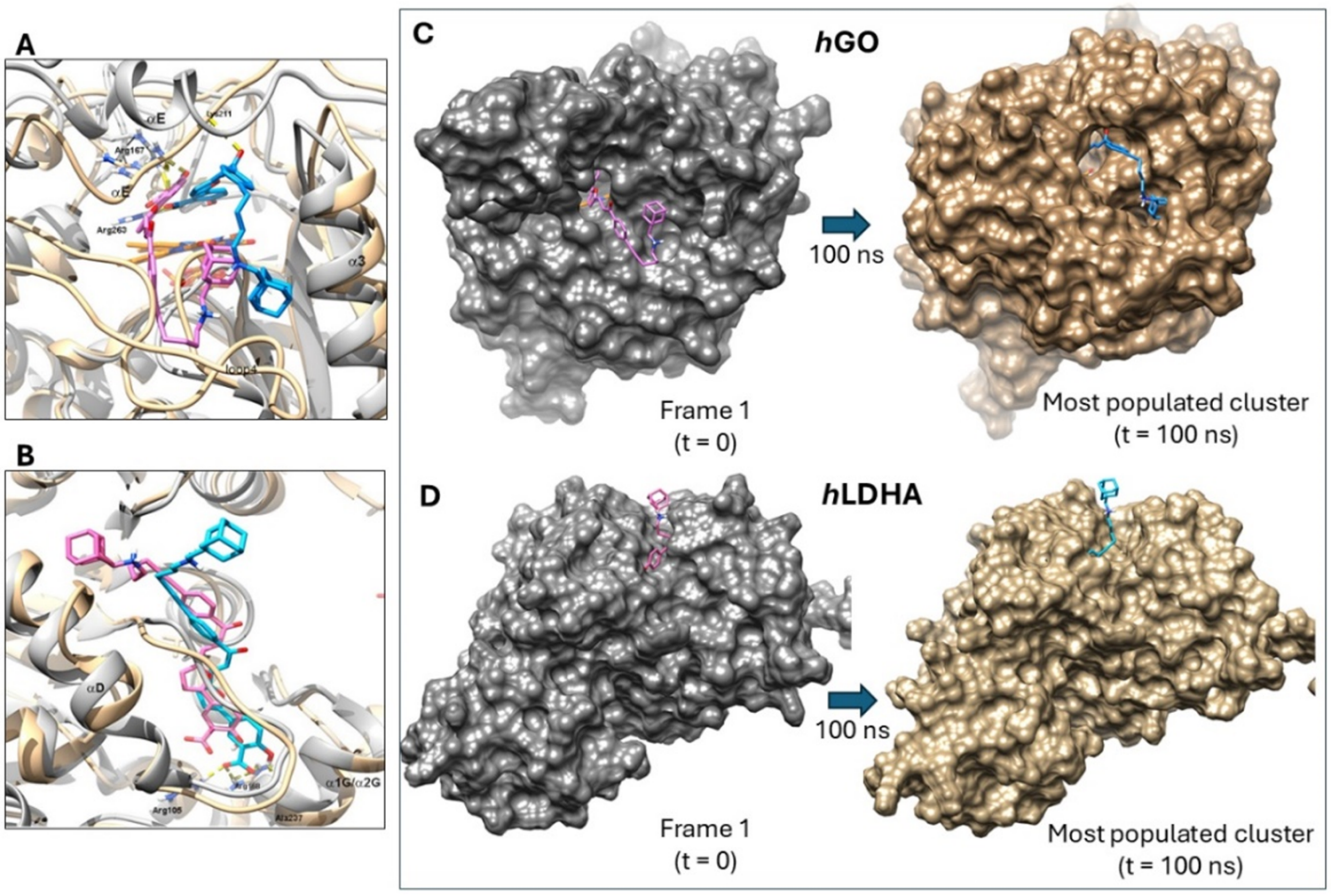
Molecular dynamics simulation. (A). Superposition of the representative pose of **2** (blue) within the most populated cluster of *h*GO (2RDT) (tan) and the initial conformation of **2** (pink) in the **2**-*h*GO ensemble (gray) used to initiate the MD simulation (frame 1). Hydrogen bonds are represented by dashed yellow lines. (B) Superposition of the representative pose of **2** (blue) in the most populated cluster of *h*LDHA (1I10) subunit B (tan) and the initial conformation of **2** (pink)-*h*LDHA dimeric ensemble (gray) (frame 1) that started the MD simulation. Hydrogen bonds are represented by dashed yellow lines. (C, D) Surface representations of the starting frame 1 of **2** that initiated the MD simulation (left), and the most populated clusters of **2** at the end of the MD simulation (right) on *h*GO (C) and *h*LDHA (D). Exposure of the adamantane hydrophobic tag over the protein surface can be observed in *h*LDHA but not in *h*GO.

An RMDS cluster analysis was also carried out to the **2**-***h*** **LDHA** dimeric ensemble MD simulation. The most populated cluster-representative structure is shown in Figures 5B,D and S88 (blue). Here, the pose of **2** on *h*LDHA subunit B is noteworthy. Its salicylic moiety is inserted into the catalytic site establishing H-bonding interactions with important residues such as Arg105 and Arg168 (Figure 5B), which are responsible for the pyruvate/lactate binding, and the backbone chain of Ala237.^41^ The furan and unsaturated phenylketone are displayed toward the entrance of the catalytic site and along the NAD^+^ cofactor binding site, keeping a uniform distance with close residues such as Val30 and Arg98 among others. On the other hand, the adamantane moiety is set outside the substrate and cofactor binding site, protruding the protein surface and exposing both the amino and adamantane ring to the surrounding solvent. The outside disposition of the adamantane moiety is clearly observed when overlaying the initial **2**-*h*LDHA dimeric ensemble (*t* = 0, pink) and the pose of **2** in the most populated cluster of *h*LDHA (blue) after the MD simulation (Figure 5B,D). The most notable differences between the two binding poses are the deeper insertion of **2** into the catalytic pocket after the simulation, caused by the outward movement of the α-1G/α-2G helix, and the external positioning of the adamantane ring. This outward orientation of the adamantane (Figure 5D), along with the stabilization of the dimeric complex by **2**, likely accounts for the ligand-induced protein degradation observed in hepatocytes.^29,30^

By contrast, docking simulations performed with the other potential HyT-PD, compound **26**, using the same crystal structures of *h*GO and *h*LDHA, revealed that the adamantane tag remains buried within the protein structure and is not exposed on the protein surface (Figures S89 and S90). This orientation likely limits recognition by the molecular degradation machinery, thereby providing a plausible structural explanation for the absence of targeted degradation observed in the presence of compound **26**.

### Compound **2** Presents Molecular Chameleonicity with Solvent-Triggered Intramolecular Bond Formation

To complete the *in silico* characterization of compound **2**, we further analyzed its theoretical pharmacokinetic properties within the beyond Rule-of-5 (bRo5) chemical space. Protein degraders often display poor solubility and limited permeability due to their structural complexity. However, molecular chameleonicity—the ability of a compound to adapt its conformation to different environments—has been proposed as a compensatory mechanism that may overcome unfavorable ADME predictions.

This adaptive behavior refers to a molecule’s capacity to form intramolecular hydrogen bonds (IMHBs) in nonpolar media, effectively masking polar groups, while adopting more extended conformations in aqueous environments to improve solubility.^42^ Chameleonicity directly correlates with dynamic molecular descriptors such as tridimensional polar surface area (3D-PSA), radius of gyration (*R*_gyr_), and IMHB formation across solvent.^42,43^ Conformational clustering based on these parameters typically identifies three populations: (i) polar-open states in water, (ii) folded-nonpolar states in chloroform, and (iii) intermediate conformations. These clusters help relate chameleonic behavior to permeability indices like cChameCS and cChameP.

Conformational sampling of compound **2** revealed a strong, environment-dependent reorganization of polarity and shape. Conformers generated in water and chloroform occupied distinct regions across all three descriptors—3D-PSA, *R*_gyr_, and IMHBs.

In water, 3D-PSA values clustered at high levels, reflecting extended conformations that favor solvent hydrogen bonding. In contrast, chloroform exposure caused a ∼10 Å^2^ decrease in PSA, indicating polarity masking *via* chameleonic folding (Figure S91A). Shielding polar surface area necessarily requires a geometrical contraction, and this is captured by the *R*_gyr_ distributions. Whereas the aqueous ensemble spans a wide range of sizes (4.8–10 Å), the chloroform ensemble collapses into a narrow band centered at ≈5 Å (Figure S91B). This polarity–size trade-off is underpinned by a surge in IMHB formation: virtually all chloroform conformers form two or more internal hydrogen bonds, compared with a single IMHB in most aqueous structures. The data provide a direct mechanistic link between folding and polarity masking (Figure S91C).

Bringing these descriptors together highlights their synergistic interplay. Low-PSA, low-*R*_gyr_ conformers are exclusively associated with ≥ 2 IMHBs and are sampled only in chloroform, whereas high-PSA, high-*R*_gyr_ conformers with ≤1 IMHB populate the aqueous ensemble. The scatter plot therefore visualizes the solvent-specific conformational partitioning that is the essence of IMHB-mediated chameleonicity (Figure 6A).

**Figure 6:**
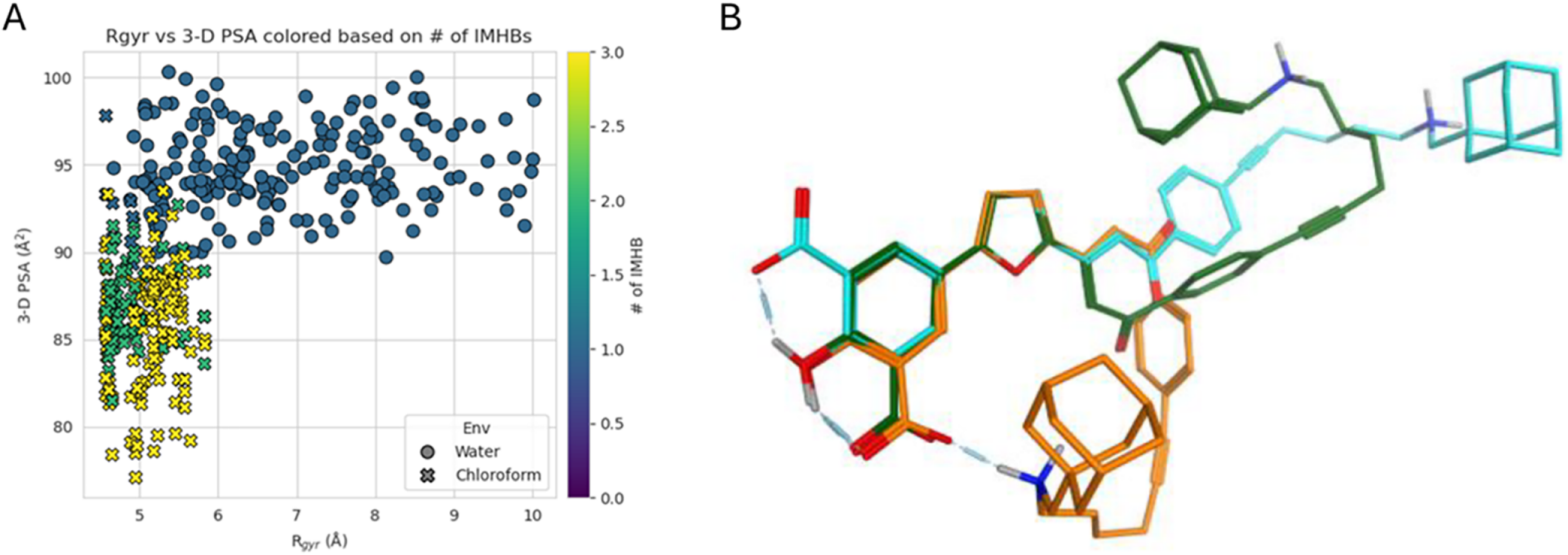
Compound’s **2** chameleonicity properties. (A) Scatter plot of radius of gyration (*R*_gyr_) *versus* tridimensional polar surface area (3D-PSA) for all conformers; points are colored by intramolecular hydrogen bonds (IMHB) count (purple =  0, blue = 1, green = 2, yellow = 3). Cross markers denote chloroform conformers; circles denote water conformers. (B) Superposition of the centroid conformers of compound **2** representing the closed (orange), semiclosed (green) and open (cyan) states identified by k-means clustering.

Clustering of the full data set yielded three centroids (Figure 6B) that trace a stepwise opening pathway: a highly folded, double-IMHB “closed” state, a partially unfolded “semiclosed” state with one IMHB, and a fully extended “open” state devoid of internal bonds. The progression between these states rationalizes the continuous distributions observed in Figures 6A and S91A–C. It is worth noting that in compound **2**, which is zwitterionic at physiological pH, the protonated amine (NH^+^) interacts intramolecularly with the carboxylate group through hydrogen bonding. Given the zwitterionic nature of the molecule, this contact could also be regarded as an intramolecular ion pair in physiological conditions. Such intramolecular contacts are well recognized to stabilize folded conformers and are likely to contribute to the compound’s chameleonic behavior.

The separation between the aqueous and chloroform ensembles is captured quantitatively by cChameCS = 0.312 and cChameP = 0.093. Values in this range indicate moderate but meaningful chameleonicity—sufficient to reduce polarity in nonpolar environments without compromising conformational diversity in water. Similar magnitudes have been associated with orally bioavailable bRo5 molecules in previous benchmark studies.^42,43^

### Oral Administration of Compound **2** Degrades Hepatic LDHA, Decreases Urinary Oxalate, and Diminishes Renal Deposition of Calcium-Oxalate Crystals in PH1 Mice

Once binding affinity, potency and mechanism of action for compound **2** had been successfully characterized, we sought to translate our findings *in vivo*. *Agxt* ^–/–^ mice were orally administered once a day for ten consecutive days, with either a vehicle solution (0.6% methylcellulose and 0.5% Tween 80 in water), or compound **2** at a dose of 20 mg/kg/day dissolved in the same formulation. To monitor changes in excreted levels of oxalic and glycolic acid, urine was collected starting 2 days prior to dosing to set a baseline and throughout the treatment regimen. On day ten, liver, kidney and plasma samples were harvested to further investigate the systemic effects of compound **2** (Figure 7A).

**Figure 7:**
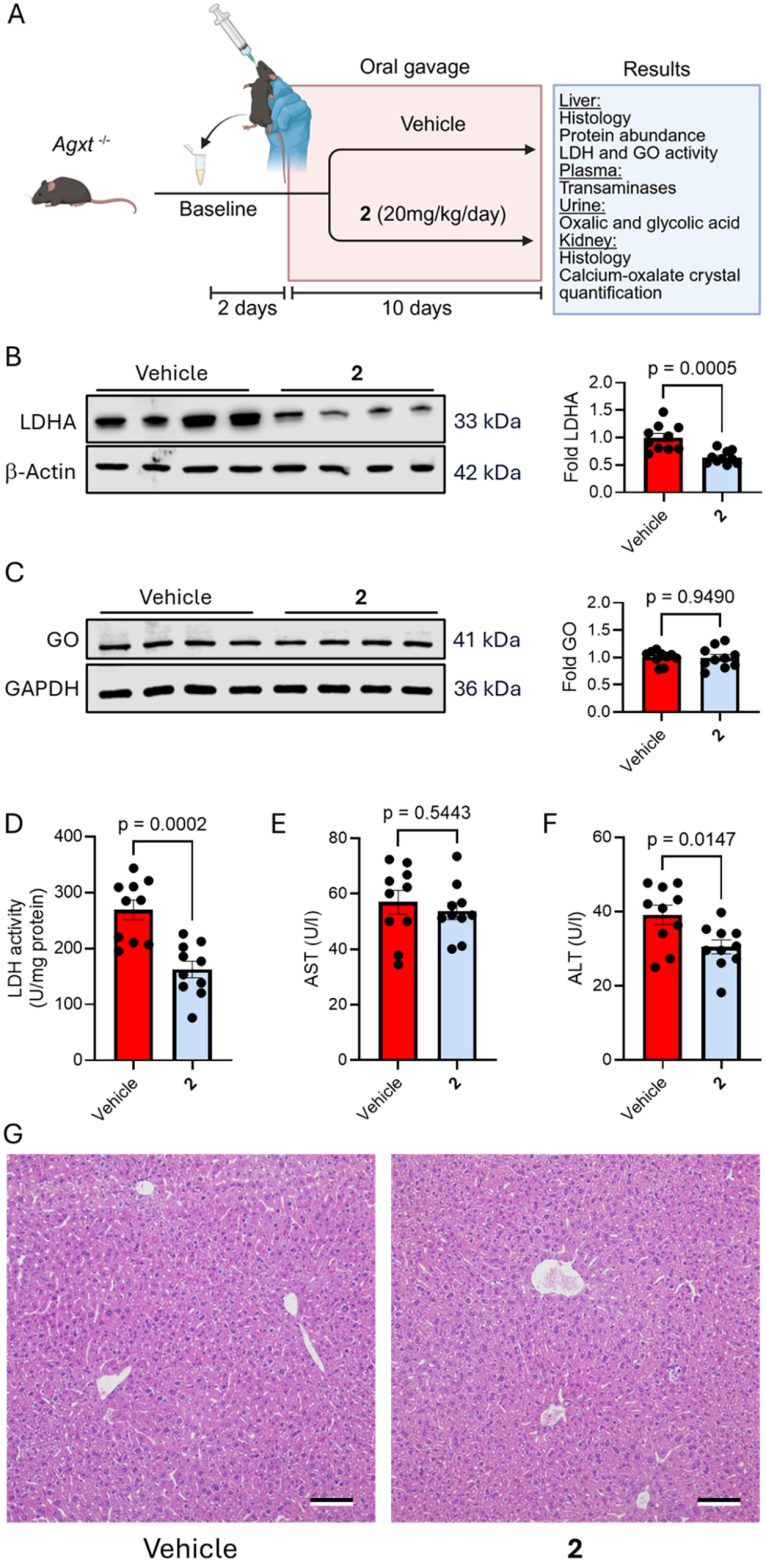
Therapeutic effects of compound **2** in the liver of PH1 mice. (A) Schematic representation of the experimental approach and end point analyses. Male *Agxt* ^–/–^ mice were orally administered with either vehicle (0.6% methylcellulose and 0.5% Tween 80 in water, *n* = 10) or compound **2** (20 mg/kg body weight, *n* = 10) daily for 10 days. (B) LDHA protein abundance relative to β-Actin in the liver at the end of the treatment. (C) GO protein abundance relative to GAPDH in the liver at the end of the treatment. (D) LDH activity measured in the liver at the end of the treatment. (E, F) Activity of AST and ALT measured in plasma at the end of the treatment. (G) H&E (hematoxylin and eosin) staining of liver sections from mice at the end of the treatment (scale bar 100 μm). Data are expressed as mean ± SEM. Normality was assessed using the Shapiro-Wilk test. Comparisons between two groups were performed using Student’s *t*-test, as normality was assumed in all cases.

We first focused on the liver, since it is the source of oxalate overproduction in PH1.^44^ Consistent with the *in vitro* results in *Agxt* ^–/–^ primary hepatocytes, we observed a significant reduction in hepatic LDHA expression in the treatment group as compared to vehicle (Figure 7B), with no changes in GO expression (Figure 7C). This reduction in LDHA abundance, together with the reported inhibition of this isozyme (Table 3) led to a 40% decrease in LDH hepatic activity (Figure 7D). In parallel, GO activity in livers from mice treated with compound **2** showed a 25% decrease (Figure S92). The observed hepatic phenotype following oral administration of compound **2** suggests that this inhibitor accumulates in the liver at levels comparable to those achieved in the *in vitro* study using primary hepatocytes (Figure 4B). To further investigate this, we conducted UPLC-Orbitrap HRMS/MS analyses to quantify the concentration of compound **2** in treated primary hepatocytes and in liver samples from treated mice, in both LDHA was degraded. We found that hepatic levels of compound **2** were comparable to those measured in isolated hepatocytes (Figure S93).

Although compound **2** demonstrated potent pharmacological activity, assessing its potential hepatotoxic effects was crucial. Notably, plasma activity of aspartate transaminase (AST), remained unchanged (Figure 7E), and a modest but statistically significant reduction in alanine aminotransferase (ALT) was observed in the treatment group compared to vehicle (Figure 7F). Finally, histological examination of liver sections using H&E (hematoxylin and eosin) staining revealed no morphological changes between the groups (Figure 7G). Moreover, there were no statistically significant differences in blood glucose, body weight, liver weight, or liver-to-body weight ratio between the groups (Figure S94A–D). These findings indicate that treatment with compound **2** has no hepatotoxic effects.

To evaluate the potential effects of compound **2** on key extrahepatic tissues, LDH activity was measured in kidney, skeletal muscle, and heart tissues. No significant differences in enzymatic activity were observed in these tissues compared to untreated mice (Figure S95A–C). To further support the tissue specificity suggested by the LDH activity results, UPLC-Orbitrap HRMS/MS analyses were performed to quantify the concentrations of compound **2** across these tissues. Compound **2** was detected exclusively in the liver (Figure S96), suggesting it undergoes an extensive hepatic uptake with minimal distribution to extrahepatic tissues.

After characterizing the hepatic effects of compound **2**, we next investigated its impact on the renal system. Urinary oxalate concentration, the primary parameter used to monitor the progression of PH1,^44^ was measured to evaluate treatment efficacy (Figure 8A). Baseline urinary oxalate levels were determined in wild-type *Agxt* ^+/+^ and untreated hyperoxaluric *Agxt* ^–/–^ mice (Figures 8A and S97) for comparison. Mice subjected to treatment with compound **2** exhibited a significant reduction in urinary oxalate levels after just 1 day of dosing, with a sustained ∼50% decrease from day 3 onward (Figure 8A). Concurrently, and consistent with GO inhibition,^45,46^ urinary glycolate levels progressively increased over time, reaching significance on day 4 (Figure 8B) and markedly exceeding those observed in baseline measurements (Figures 8B and S97).

**Figure 8:**
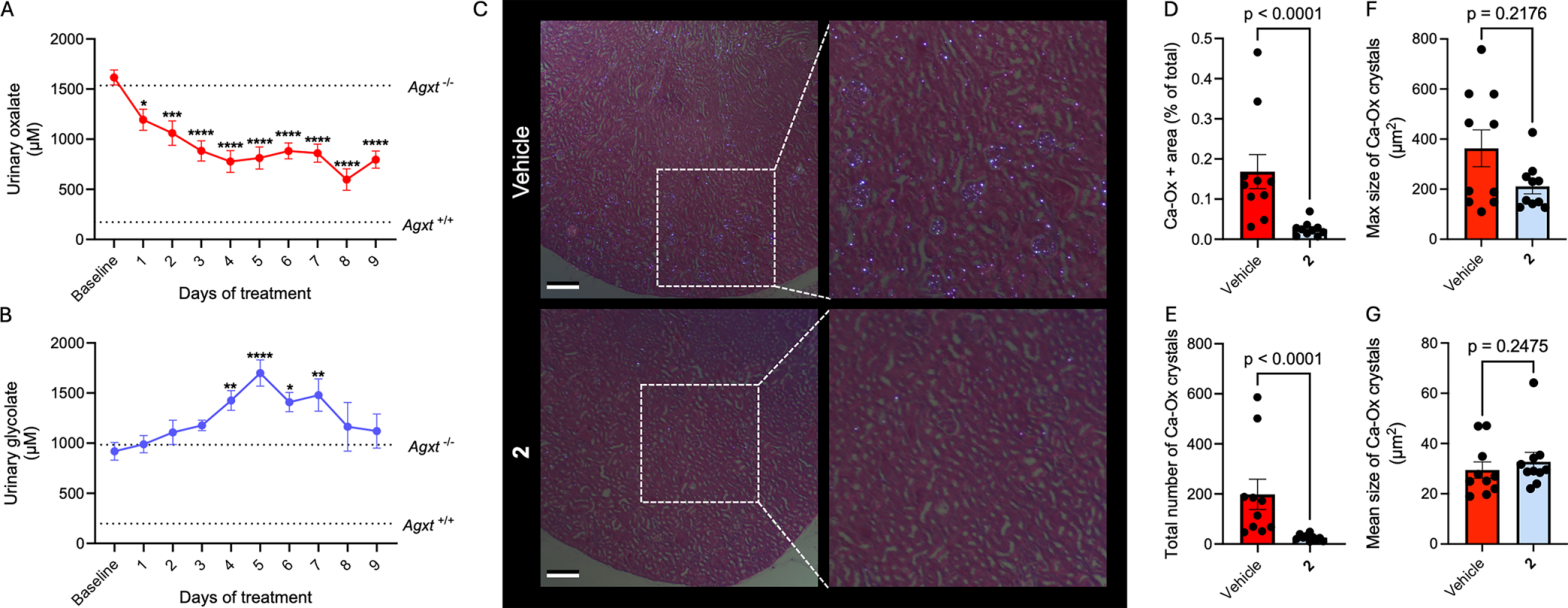
Therapeutic effects of compound **2** at the renal level of PH1 mice. Male *Agxt* ^–/–^ mice were orally administered with either vehicle (0.6% methylcellulose and 0.5% Tween 80 in water, *n* = 10) or **2** (20 mg/kg body weight, *n* = 10) daily for 10 days. (A) Urinary oxalate concentration during treatment. (B) Urinary glycolate concentration during treatment. (C) Polarized light microscopy of kidney tissue stained with H&E at the end of the treatment (scale bar = 200 μm). (D) Quantification of calcium-oxalate (Ca-Ox) deposition by % area per image (*n* = 10). (E) Total number of Ca-Ox crystals per section. (F) Size of the largest Ca-Ox crystal per section. (G) Mean Ca-Ox crystal size per section. Histopathological analyses were performed by two blinded and one nonblinded pathologist, and results were combined to obtain the overall means for each parameter. Data are expressed as mean ± SEM. Normality was assessed using the Shapiro-Wilk test. Comparisons among multiple groups that passed normality testing were analyzed using one-way ANOVA followed by Tukey’s post hoc test. Groups that did not meet normality assumptions were analyzed using the Kruskal–Wallis test followed by Dunn’s post hoc test. Comparisons between two groups were performed using the Mann–Whitney test, as normality was not assumed.

This metabolic shift led us to hypothesize that calcium-oxalate crystal deposition within the kidneys might also be altered. To test this, kidney sections from mice treated with either vehicle or compound **2** were stained with H&E and imaged under polarized light to visualize calcium-oxalate crystals as bright birefringent spots (Figure 8C). Quantification of the percentage area occupied by these crystals within the kidney sections showed a striking reduction in mice treated with compound **2** (Figure 8D).

To better understand the source of this reduction, we performed a comprehensive analysis of parameters commonly reported for renal calcium-oxalate deposits.^47^ Treatment with compound **2** resulted in an approximately 87% decrease in total number of crystals (Figure 8E), accompanied by a decreasing trend in the size of the largest crystal per field of view (Figure 8F). In contrast, the mean crystal size showed no significant changes (Figure 8G). Altogether, these findings indicate that compound **2** prevents the formation of new crystals and suggest that the growth of preexisting ones is halted. Collectively, these comprehensive studies highlight the potential of HyT-PD, and particularly compound **2**, as an effective oxalate-lowering therapy for PH1.

## Discussion and Conclusion

Lumasiran and nedosiran represent important therapeutic milestones in the management of PH1.^48^ Both FDA-approved drugs employ GalNAc-conjugated RNA interference strategies targeting either GO^46^ or LDHA,^14^ offering a hepatocyte-specific means to reduce oxalate synthesis. While these therapies have markedly improved outcomes in many patients,^15^ variability in therapeutic response remains a challenge.^49–53^ Moreover, their clinical use is further constrained by restrictive administration routes and high commercialization costs.^23^ To overcome these limitations, we aimed to develop an alternative, orally bioavailable, and cost-effective therapy that combines dual GO/LDHA targeting and a dual inhibition-degradation mechanism. In this study, we optimized the chemical structure of previous GO and LDHA dual inhibitors to (1) enhance their potency into the nanomolar range, (2) promote selective degradation of target enzymes, and (3) demonstrate potent *in vivo* efficacy. We report the synthesis and characterization of 33 novel compounds, derived from oxophenylpropenyl furylsacylic acid. Based on their inhibitory profile in recombinant enzyme assays, 18 inhibitors were selected for intracellular oxalate quantification in *Agxt* ^–/–^ primary hepatocytes. Among them compound **2** emerged as the most potent in lowering intracellular oxalate. Oral administration of compound **2** to *Agxt* ^–/–^ mice significantly decreased hepatic LDHA abundance and enzymatic activity, thereby lowering urinary oxalate levels, and abolished renal calcium-oxalate crystal deposition without evidence of hepatotoxicity.

Traditionally, GO inhibitors have been designed to form H-bonds with key active-site residues and to engage the hydrophobic access channel.^33^ Similarly, LDHA inhibitors occupy both the polar substrate pocket and the NAD^+^-associated hydrophobic cleft.^54^ Because both enzymes act on α-hydroxy- or α-ketoacid substrates, their inhibitors often share structural features, motivating dual-targeting approaches previously explored by our group.^31^ Guided by these insights, here, we designed the OPPFSAs scaffold where the β-hydroxyacid warhead mimics substrate binding, while C5 substituents fit either the GO hydrophobic channel or the LDHA cofactor cleft. Optimization led to compounds **2**, **20** and **23** which achieved nanomolar *K*_i_ values improving affinity up to 90 and 205-fold for *h*GO and *h*LDHA respectively, when compared to the lead **1**.

Given that recombinant enzyme assays do not fully reflect the complexity of PH1 pathophysiology, we next evaluated oxalate reduction in *Agxt* ^–/–^ primary hepatocytes. All OPPFSAs reduced extracellular oxalate, but intracellular measurements revealed distinct efficacy. Highly potent inhibitors such as compounds **20** and **23**, performed poorly in this cellular context, likely due to poor water solubility and slow membrane translocation; features common to previously reported GO inhibitors.^55^ In contrast, compounds **16** and **19**, bearing shorter alkyl chains, demonstrated greater intracellular efficacy, underscoring that cellular access rather than intrinsic potency governs pharmacological activity.

Compound **2** retained strong intracellular potency despite similarities with the less active analogs. The presence of a terminal amino group likely improved solubility and distribution at physiological pH, supported by *in silico* analyses showing balanced polarity and chameleonic flexibility, adopting a polar conformation in aqueous media and a compact disposition in hydrophobic environments. This conformational adaptability reconciles solubility with membrane permeability, thereby providing a mechanistic rationale for its superior cellular performance.

Designed as a hydrophobic tag–based proteolysis degrader, compound **2** induced a dose-dependent reduction of LDHA abundance in hepatocytes independent of mRNA expression. This shift in enzymatic activity is comparable to the effects of irreversible enzyme inhibitors^56^ or siRNA-mediated knockdown.^57^ To elucidate the mechanism, we explored how compound **2** mediates this effect in the presence of proteasome and autophagy inhibitors, demonstrating that compound **2** promotes the lysosome-dependent degradation of LDHA.

Molecular dynamics simulations show compound **2** binding initially to the NADH cofactor site, followed by a conformational rearrangement that pushes the salicylic acid warhead deeper into the catalytic pocket and exposes the adamantane moiety above the protein surface, enabling recognition by the degradation machinery. A similar behavior was not observed for compound **26**, in which the adamantane moiety remains buried within the protein structure, thereby preventing any potential proteolytic activity. Together, these observations explain both the noncompetitive inhibition and lysosome-dependent degradation selectively induced by compound **2**.

Building on the demonstrated oxalate-lowering efficacy of compound **2** in cellular models, we evaluated next its therapeutic performance *in vivo*. To our knowledge, only one small-molecule LDHA degrader has been previously reported; MS6105, designed as a PROTAC for pancreatic cancer, induces proteolysis *via* the ubiquitin-proteasome system but requires intraperitoneal administration for its *in viv*o activity.^58^ Oral administration is a key factor in improving treatment adherence and patient convenience during chronic therapies.^59,60^ Through this route, mice with PH1 (*Agxt* ^–/–^) were administered compound **2** at half the molar dose reported in our previous studies. As a result, urinary oxalate significantly decreased within 24 h, with maximal reduction by day 4 and sustained effect thereafter. The oral route ensured sufficient hepatic exposure to lower LDHA protein and activity by ∼50% without affecting LDH-rich tissues such as skeletal muscle or heart, suggesting a hepato-selective distribution that was further confirmed by mass spectrometry analysis. GO activity decreased by ∼25%, consistent with the observed rise in urinary glycolate midtreatment that was lost after day 7, suggesting a less prominent effect on this enzyme.

The most compelling evidence of compound **2**’s therapeutic efficacy was an 85% reduction in renal calcium-oxalate crystal deposition. The observed distribution of residual crystals suggests that pre-existing deposits ceased growing, substantially lowering the risk of oxalate urolithiasis. These findings, together with the absence of any detectable toxicity, underscore the strong potential of compound **2** as a promising drug candidate for the treatment of PH1.

MD simulations provided a structural rationale for the absence of GO degradation. Unlike canonical GO inhibitors that stabilize an open loop-4 conformation and maintain the hydrophobic channel accessible, compound **2** induced loop closure through hydrophobic contacts between its adamantane group and loop-4 residues, further stabilized by a Lys211 hydrogen bond. This buried the adamantane group preventing its surface exposure and therefore impeding proteolysis. A strategy we aim to pursue in future designs is to introduce a more rigid or extended linker to expose the adamantane group to the cellular milieu, in order to enable GO degradation.

In conclusion, compound **2** represents a first-in-class, orally bioavailable small-molecule that lowers hepatic oxalate production and renal calcium-oxalate deposition through a dual mechanism combining GO and LDHA inhibition with selective degradation of the latter. This HyT-PD scaffold offers a promising and accessible alternative to RNA-based therapeutics. Beyond PH1, its mechanism may have broader applications in diseases linked to hepatic oxalate overproduction, including metabolic dysfunction-associated steatohepatitis (MASH) and cardiovascular disorders. Continued optimization of linker geometry and pharmacokinetic properties will be essential for advancing this chemical series toward clinical translation.

## Experimental Section

### Chemical Methods

#### General Considerations

All solvents and chemicals were used as purchased without further purification. The progress of the reactions was controlled by (a) thin layer chromatography (TLC) on aluminum plates (Merck AL Silicagel 60 F254) and visualized by UV lamp (254 nm) or by staining with iodine or solutions of (i) permanganate in distilled water (1% p/v), (ii) vanillin (0.5 g) in sulfuric acid/EtOH (1:3, 100 mL), (iii) ninhydrin (1 g) in EtOH (450 mL); (b) Liquid Chromatography–Mass Spectrometry (LC-MS) in an Agilent 1200 HPLC coupled with an Agilent 6110 single quadrupole, with electrospray ionization (ESI) in positive or negative mode. The HPLC column used was a Zorbax Eclipse XDB-C18 5 μM, 4.6 × 150 mm^2^ column or a Waters XBridge Column C18 3.5 μM, 2.1 × 100 mm^2^. Purification by flash column chromatography (FCC) was performed on Silicagel Merck 60 (230–400 mesh ASTM). Automated FCC was carried out using a Biotage Isolera One apparatus with UV–vis detector. Purification by preparative layer chromatography (PLC) was made on Silicagel Merck 60 F_254_, 0,5 mm plates. The noncrystalline compounds were shown to be homogeneous by chromatographic methods and characterized by nuclear magnetic resonance (NMR) and high-resolution mass spectrometry (HRMS). ^1^H NMR and ^13^C NMR spectra have been recorded in a 2-channel 400 MHz Bruker Nanobay Avance III HD, a 2-channel 400 MHz Bruker Avance NEO, and a 2-channel 500 MHz Bruker Avance NEO spectrometers using DMSO-*d*_6_, methanol-*d*_4_, chloroform-*d* or acetone-*d*_6_. Chemical shifts (δ) are quoted in parts per million (ppm) and are referenced to residual H in the deuterated solvent as the internal standard. Coupling constants (*J*) are expressed in Hz. Splitting patterns are designated as follows: bb, broad band; bs, broad singlet; d, doublet; dd, double doublet; m, multiplet; pdt, pseudo double triplet; q, quadruplet; s, singlet; t, triplet. HRMS were recorded by time-of-flight (TOF) mass spectrometry with ESI in positive or negative mode, using a QTOF apparatus Bruker Compact. Purity of the compounds was assessed by HPLC using an Agilent 1200 instrument with diode-array detector equipped with a suitable column (General HPLC methods, below). Column temperature was set at 25 °C. Absorbance was measured at 214 and 254 nm. Purity of final compounds was higher than 95% by HPLC (except for compound **7**, **15**). An EnSpire Multimode Plate Reader (PerkinElmer) was used to measure fluorescence. For fluorometric assays, OptiPlate black opaque 96-well microplates (PerkinElmer) were used. For all assays, the final volume in each well was 200 μL. Multichannel pipettes were used for the addition of the reagents in the wells. Interferences in the kinetic fluorometric protocols were discarded as the slope in the linear interval was corrected by subtraction of the baseline reading registered before the addition of the substrate.

#### General HPLC Methods

General HPLC setup: Detection λ = 214 and 254 nm; flow rate 0.8 mL/min; solvent A [water (0.1% HCOOH)/acetonitrile (0.1% HCOOH)]; solvent B [acetonitrile (0.1% HCOOH) 100%]. Injection volume 10 μL.

Column 1: Zorbax Eclipse XDB-C18 5 μM, 4.6 × 150 mm^2^.

Column 2: Waters XBridge Column-C18 2.5 μm, 4.6 × 75 mm^2^.

Column 3: Waters XBridge Column-C8 2.5 μm, 4.6 × 75 mm^2^.

*HPLC method A*: Solvent A (95/5). Isocratic A 2 min + gradient A → B 8 min + isocratic B 1 min (compound **8**).

*HPLC method B*: Solvent A (90/10). Isocratic A 2 min + gradient A → B 8 min + isocratic B 1 min (compounds **1** and **S1**).

*HPLC method C*: Solvent A (70/30). Isocratic A 2 min + gradient A → B 8 min + isocratic B 1 min (compounds **7**, **9**, **10** and **S3**).

*HPLC method D*: Solvent A (60/40). Isocratic A 2 min + gradient A → B 8 min + isocratic B 1 min (compound **S5**).

*HPLC method E*: Solvent A (50/50). Isocratic A 2 min + gradient A → B 8 min + isocratic B 1 min (compounds **6**, **14**-**16**, **25**, **27**, **S2**, **S4** and **S6**).

*HPLC method F*: Solvent A (40/60). Isocratic A 2 min + gradient A → B 8 min + isocratic B 1 min (compound **23**).

*HPLC method G*: Solvent A (30/70). Isocratic A 2 min + gradient A → B 8 min + isocratic B 1 min (compounds **12**, **18**-**21**, **24**, **28** and **29**).

*HPLC method H*: Solvent A (10/90). Isocratic A 2 min + gradient A → B 8 min + isocratic B 1 min (compound **22**).

*HPLC method I*: Solvent A (60/40). Isocratic A 2 min + gradient A → B 17 min + isocratic B 1 min (compounds **4**, **5**, **11** and **13**).

*HPLC method J*: Solvent A (70/30). Isocratic A 2 min + gradient A → B 17 min + isocratic B 1 min (compound **17**).

*HPLC method K*: Solvent A (80/20). Isocratic A 1 min + gradient A → B 5 min + isocratic B 1 min (compound **2**).

#### General Conditions for Aldol Condensation

*Protocol A*: To a stirred solution of compound type **33** (1.5 equiv) in solvent mixture containing mainly MeOH, a 10 N NaOH aqueous solution was added dropwise. After 10 min, a solution of **32** (1 equiv) was added. After 24 h stirring at rt, the reaction was quenched by addition of HCl 3N until pH 3 at rt. The organic solvents were evaporated under reduced pressure and 20 mL of AcOEt were added. The organic layer was washed with water and brine, dried over MgSO_4_, filtered and concentrated under reduced pressure. The final products (salicylate esters or salicylic acids) were purified by FCC. *Protocol B*: To a stirred solution of compound type **33** (1.2 equiv) in EtOH (1 mL), 10 N NaOH aqueous solution (3 or 30 equiv) was added dropwise at 0 °C and the resulting solution was stirred at the same temperature for 30 min. After this time, **3** ^31^ (50 mg, 0.215 mmol, 1 equiv) was added and the reaction was allowed to reach rt. Upon reaction completion (3 h or overnight), it was acidified with HCl (1 N) to pH 1–2 at 0 °C and the solvent was evaporated under reduced pressure. The residue was percolated through a short column of silica gel eluting with DCM/MeOH (80:20) and the product was purified by PLC.

#### General Conditions for Selective Double Bond Hydrogenation in α,β-Unsaturated Ketones Type 34

The corresponding α,β-unsaturated ketone type **34** (1 equiv) was dissolved in THF at rt. Then, Raney Nickel activated catalyst (50% slurry in water) (1 mL) was added. For the reaction to take place, hydrogen (g) was bubbled through the solution for 1 h. Once completed, the reaction mixture was filtered through Celite and concentrated under reduced pressure. FCC purification was performed by elution using mixtures of petrol ether and AcOEt.

#### General Conditions for Hydrolysis of Methyl Salicylates

The corresponding methyl salicylate (1 equiv) was dissolved in pyridine (10 mL/mmol) and heated up to reflux during 15 h. After this time, pyridine was removed using a high vacuum rotavapor. The residue was dissolved in AcOEt, and the remaining trace of pyridine was extracted with aqueous HCl 1N. The organic layer was dried over MgSO_4_, filtered and concentrated under reduced pressure. FCC purification was performed using petrol ether/DCM/MeOH mixtures acidified with 1% AcOH.

##### 5-{5-{(*E*)-3-{4-[6-(Adamantan-1-ylmethylamino)­hex-1-ynyl]­phenyl}-3-oxoprop-1-enyl}­furan-2-yl}-2-hydroxybenzoic Acid (**2**)

Compound **34-Ad** (148 mg, 0.25 mmol, 1 equiv) was added on a solution of NaOH (40 mg, 1 mmol, 4 equiv) in MeOH/water (3:1). The mixture was let to stir at 40 °C for 12 h. After this time, the organic solvent was removed under vacuum and a volume of DCM was added. The resulting organic phase was washed with aqueous HCl (1N) (x2), dried over anhydrous MgSO_4_, filtered and evaporated under vacuum. FCC purification (when necessary): Gradient elution with mixtures petrol ether/DCM/MeOH (44:44:2 → 40:40:20) + AcOH (1%). Orange solid, 90% yield (130 mg, 0.23 mmol). ^1^H NMR (500 MHz, DMSO-*d*_6_) δ 8.17 (bb, 1H), 8.14 (s, 1H), 8.05 (d, *J* = 8.0 Hz, 2H), 7.75 (d, *J* = 8.7 Hz, 1H), 7.59–7.53 (m, 3H), 7.48 (d, *J* = 15.1 Hz, 1H), 7.17 (d, *J* = 3.0 Hz, 1H), 6.90 (d, *J* = 3.2 Hz, 1H), 6.75 (d, *J* = 8.5 Hz, 1H), 2.96 (bs, 2H), 2.64 (s, 2H), 2.55 (t, *J* = 6.6 Hz, 2H), 1.97 (s, 3H), 1.84 (bs, 2H), 1.70–1.53 (m, 14H). ^13^C NMR (126 MHz, DMSO-*d*_6_) δ 187.33, 170.71, 165.16, 157.69, 149.48, 136.88, 131.59, 130.40, 128.51, 128.41, 127.43, 126.38, 121.32, 120.17, 117.25, 117.10, 115.98, 106.43, 93.75, 80.71, 58.49, 48.07, 39.04, 36.01, 31.85, 27.33, 25.06, 24.30, 18.43. HRMS: *m*/*z* calcd. for [M + H] C_37_H_40_NO_5_ 578.2901; found, 578.2909 (deviation −1.3 ppm). HPLC (method K) (λ = 254 nm), 95.4%; (λ = 214 nm), 97.2%; ^*t*^R = 7.16 min (column 3).

##### (*E*)-2-Hydroxy-5-[5-(3-phenyl-3-oxoprop-1-en-1-yl)­furan-2-yl]­benzoic Acid (**4**)

General conditions for aldol condensation (protocol B) using acetophenone (30 μL, 0.26 mmol, 1.2 equiv) and 10 M NaOH aqueous solution (65 μL, 0.65 mmol, 3 equiv). Reaction time, 3 h. PLC purification: DCM/AcOH (99:1). Orange solid (48 mg, 0,14 mmol, 67% yield). mp, decomposes at approximately 180 °C. ^1^H NMR (400 MHz, methanol-*d*_4_) δ 8.35 (s, 1H), 8.05 (d, *J* = 7.3 Hz, 2H), 7.83 (d, *J* = 8.3 Hz, 1H), 7.64–7.50 (m, 5H), 6.96 (s, 2H), 6.81 (s, 1H). ^13^C NMR (101 MHz, methanol-*d*_4_) δ 191.9, 163.7, 158.3, 151.9, 139.5, 134.0, 132.0, 131.4, 129.8 (2C), 129.5 (2C), 128.1, 122.0, 121.1, 118.7 (2C), 108.0. HRMS (TOF, ES^–^): *m*/*z* calcd for C_20_H_13_O_5_ (M - H^+^) 333.0763, found 333.0757 (deviation −1.8 ppm). HPLC (method I) (λ = 254 nm), 96.4%; (λ = 214 nm), 95.3%; ^*t*^R = 12.75 min (column 1).

##### (*E*)-2-Hydroxy-5-{5-[3-(4-nitrophenyl)-3-oxoprop-1-en-1-yl]­furan-2-yl}­benzoic Acid (**5**)

General conditions for aldol condensation (protocol B) using 4-nitroacetophenone (43 mg, 0.26 mmol, 1.2 equiv) and 10 M NaOH aqueous solution (65 μL, 0.65 mmol, 3 equiv). Reaction time, 3 h. PLC purification: DCM/AcOH (99:1). Reddish-brown solid (46 mg, 0,06 mmol, 56% yield). mp, decomposes at approximately 180 °C. ^1^H NMR (400 MHz, methanol-*d*_4_) δ 8.40–8.35 (m, 3H), 8.28 (d, *J* = 9 Hz, 2H), 7.78 (dd, *J* = 8.6, 2.2 Hz, 1H), 7.65 (d, *J* = 15.1 Hz, 1H), 7.57 (d, *J* = 15.1 Hz, 1H), 7.04 (d, *J* = 3.6 Hz, 1H), 6.89 (d, *J* = 8.6 Hz, 1H), 6.82 (d, *J* = 3.6 Hz, 1H). ^13^C NMR (101 MHz, methanol-*d*_4_) δ 189.9, 164.0, 159.5, 151.5, 144.5, 133.0, 130.6 (2C), 130.3, 128.4, 124.8 (2C), 123.9, 122.4, 121.2, 120.7, 118.1, 117.7, 107.9. HRMS (TOF, ES^–^): *m*/*z* calcd for C_20_H_12_NO_7_ (M–H^+^) 378.0614, found 378.0638. HPLC (method I) (λ = 254 nm), 97.0%; (λ = 214 nm), 97.9%; ^*t*^R = 13.01 min (column 1).

##### (*E*)-5-{5-[3-(4-Cyanophenyl)-3-oxoprop-1-en-1-yl]­furan-2-yl}-2-hydroxybenzoic Acid (**6**)

General conditions for hydrolysis of methyl salicylates, using **34-CN**. FCC purification: Gradient elution using petrol ether/DCM/MeOH (49:49:2 → 40:40:20) acidified with 1% AcOH. Reddish-orange solid (87% yield). ^1^H NMR (400 MHz, DMSO-*d*_6_) δ 8.25 (d, *J* = 8.6 Hz, 2 H), 8.25 (d, *J* = 2,3 Hz, 1H), 8.08 (dd, *J* = 8.7, 2.3 Hz, 1H), 8.07–8.03 (m, 2H), 7.62 (s, 2H), 7.26 (d, *J* = 3.6 Hz, 1H), 7.15 (d, *J* = 3.6 Hz, 1H), 7.08 (d, *J* = 8.7 Hz, 1H). ^13^C NMR (101 MHz, DMSO-*d*_6_) δ 187.8, 171.3, 161.7, 155.8, 150.3, 141.1, 132.8, 131.6, 131.2, 128.9, 126.3, 121.2, 120.6, 118.3, 118.1, 117.5, 114.8, 114.2, 108.3. HRMS: *m*/*z* calcd. for [M – H] C_18_H_15_O_6_ 327.0869; found, 327.0870 (deviation: 0.3 ppm). HPLC (method E) (λ = 254 nm), 95.6%; (λ = 214 nm), 97.9%; ^*t*^R = 6.23 min (column 2).

##### (*E*)-2-Hydroxy-5-{5-[3-(4-hydroxyphenyl)-3-oxoprop-1-en-1-yl]­furan-2-yl}­benzoic Acid (**7**)

General conditions for aldol condensation (protocol A) using **32** (120 mg, 0.48 mmol, 1 equiv), *p*-hydroxyacetophenone (133 mg, 0.97 mmol, 2 equiv), NaOH 10 N (0.48 mL, 4.8 mmol, 10 equiv), THF (2 mL) and MeOH (5 mL). FCC purification: Gradient elution using petrol ether/DCM/MeOH (49:49:2 → 45:45:10) acidified with 1% AcOH. Reddish-orange solid (75% yield). ^1^H NMR (500 MHz, methanol-*d*_4_) δ 8.31 (d, *J* = 2.2 Hz, 1H), 8.03–8.00 (m, 2H), 7.94 (dd, *J* = 8.7, 2.3 Hz, 1H), 7.59 (d, *J* = 15.3 Hz, 1H), 7.55 (d, *J* = 15.3 Hz, 1H), 7.02 (d, *J* = 8.7 Hz, 1H), 6.95 (d, *J* = 3.6 Hz, 1H), 6.93–6.89 (m, 2H), 6.86–6.84 (m, 1H). ^13^C NMR (126 MHz, methanol-*d*_4_) δ 190.3, 173.5, 163.9, 163.5, 157.3, 152.3, 132.6, 132.4, 132.2, 131.1, 127.6, 122.7, 120.2, 119.1, 119.0, 116.5, 108.3. HRMS: *m*/*z* calcd. for [M + H] C_20_H_15_O_6_ 351.0869; found, 351.0881 (deviation +3.4 ppm). HPLC (method C) (λ = 254 nm), 85.3%; (λ = 214 nm), 84.6%; ^*t*^R = 9.13 min (column 2).

##### (*E*)-2-Hydroxy-5-{5-{3-[4-(hydroxymethyl)­phenyl]-3-oxoprop-1-en-1-yl}­furan-2-yl}­benzoic Acid (**8**)

General conditions for hydrolysis of methyl salicylates, using **34-CH**_**2**_ **OH** (65 mg, 0,17 mmol). FCC purification: Gradient elution using petrol ether/DCM/MeOH (49:49:2 → 45:45:10) acidified with 1% AcOH. Reddish-orange solid (91% yield). ^1^H NMR (400 MHz, DMSO-*d*_6_) δ 8.19 (d, *J* = 2.4 Hz, 1H), 8.07 (d, *J* = 8.3 Hz, 2H), 7.86 (dd, *J* = 8.6, 2.4 Hz, 1H), 7.57 (s, 2H), 7.51 (d, *J* = 8.3 Hz, 2H), 7.18 (d, *J* = 3.6 Hz, 1H), 6.97 (d, *J* = 3.6 Hz, 1H), 6.85 (d, *J* = 8.6 Hz, 1H), 5.37 (s, 1H), 4.61 (s, 2H). ^13^C NMR (101 MHz, DMSO) δ 188.0, 171.1, 163.8, 156.7, 149.8, 147.9, 136.3, 130.0, 129.4, 128.2, 126.4, 126.3, 120.4, 118.5, 117.3, 117.0, 106.8, 62.5. HRMS: *m*/*z* calcd. for [M + H] C_21_H_15_O_6_ 363.0869; found, 363.0858 (deviation −3.0 ppm). HPLC (method A) (λ = 254 nm), 100%; (λ = 214 nm), 95.6%; ^*t*^R = 10.82 min (column 2).

##### 2-Hydroxy-5-{5-{(*E*)-3-[4-((*E*)-3-hydroxy-3-oxoprop-1-enyl)­phenyl]-3-oxoprop-1-enyl}­furan-2-yl}­benzoic Acid (**9**)

Compound **24** (20 mg, 0.04 mmol, 1 equiv) was dissolved in DCM (4 mL). Trifluoroacetic acid (1.49 g, 1 mL, 13 mmol, large excess), was added on the solution and stirring at rt was maintained for 2 h. After this time solvents were removed under vacuum. FCC purification: Gradient elution using petrol ether/dicloromethane/methanol (49:49:2 → 45:45:10) acidified with 1% AcOH. Reddish-orange solid (93% yield). ^1^H NMR (400 MHz, DMSO-*d*_6_) δ 8.25 (d, *J* = 2.3 Hz, 1H), 8.14 (d, *J* = 8.5 Hz, 2H), 8.10 (dd, *J* = 8.7, 2.3 Hz, 1H), 7.89 (d, *J* = 8.4 Hz, 2H), 7.68 (d, *J* = 16.0 Hz, 1H), 7.66 (d, *J* = 15.3 Hz, 1H), 7.59 (d, *J* = 15.3 Hz, 1H), 7.23 (d, *J* = 3.6 Hz, 1H), 7.14 (d, *J* = 3.6 Hz, 1H), 7.09 (d, *J* = 8.7 Hz, 1H), 6.70 (d, *J* = 16.1 Hz, 1H). ^13^C NMR (101 MHz, DMSO-*d*_6_) δ 187.9, 171.4, 167.3, 161.4, 155.3, 150.5, 142.6, 138.6, 138.4, 131.6, 130.3, 128.8, 128.5, 126.1, 121.7, 120.9, 120.3, 118.1, 117.9, 113.9, 108.2. HRMS: *m*/*z* calcd. for C_23_H_17_O_7_ 405.0974; found, 405.0976 (deviation +0.5 ppm). HPLC (method C) (λ = 254 nm), 100%; (λ = 214 nm), 100%; ^*t*^R = 9.77 min (column 2).

##### (*E*)-5-{5-{3-[4-(2-Carboxyethyl)­phenyl]-3-oxoprop-1-en-1-yl}­furan-2-yl}-2-hydroxybenzoic Acid (**10**)

Compound **25** (1 equiv) was dissolved in DCM (4 mL). Trifluoroacetic acid (1.49 g, 1 mL, 13 mmol, large excess), was added on the solution and stirring at rt was maintained for 2 h. FCC purification: Gradient elution using petrol ether/dicloromethane/methanol (49:49:2 → 45:45:10) acidified with 1% acetic acid. Reddish-orange solid (90% yield). ^1^H NMR (500 MHz, DMSO-*d*_6_) δ 8.24 (d, *J* = 2.3 Hz, 1H), 8.09 (dd, *J* = 8.7, 2.3 Hz, 1H), 8.04 (d, *J* = 8.3 Hz, 2H), 7.63 (d, *J* = 15.0 Hz, 1H), 7.56 (d, *J* = 15.0 Hz, 1H), 7.44 (d, *J* = 8.3 Hz, 2H), 7.20 (d, *J* = 3.6 Hz, 1H), 7.13 (d, *J* = 3.6 Hz, 1H), 7.09 (d, *J* = 8.7 Hz, 1H), 2.93 (t, *J* = 7.5 Hz, 2H), 2.61 (t, *J* = 7.6 Hz, 2H). ^13^C NMR (126 MHz, DMSO) δ 188.1, 173.6, 171.4, 161.3, 155.1, 150.5, 146.5, 135.7, 131.6, 129.9, 128.7, 128.5, 126.1, 120.9, 119.9, 118.1, 113.9, 108.1, 34.7, 30.3. HRMS: *m*/*z* calcd. for C_23_H_17_O_7_ 407.1053; found, 407.1090 (deviation +2.1 ppm). HPLC (method C) (λ = 254 nm), 100%; (λ = 214 nm), 100%; ^*t*^R = 9.54 min (column 2).

##### (*E*)-2-Hydroxy-5-{5-[3-(4-bromophenyl)-3-oxoprop-1-en-1-yl]­furan-2-yl}­benzoic Acid (**11**)

General conditions for aldol condensation (protocol B) using 4-bromoacetophenone (51 mg, 0.26 mmol, 1.2 equiv) and 10 M NaOH aqueous solution (650 μL, 6.5 mmol, 30 equiv). Reaction time: overnight. PLC purification: DCM/AcOH (99:1). Red-orange solid (33 mg, 37% yield). mp, decomposes at approximately 180 °C. ^1^H NMR (400 MHz, methanol-*d*_4_) δ 8.38 (d, *J* = 2.2 Hz, 1H), 8.00–7.96 (m, 2H), 7.79 (dd, *J* = 8.6, 2.2 Hz, 1H), 7.72–7.68 (m, 2H), 7.60 (d, *J* = 15.2 Hz, 1H), 7.52 (d, *J* = 15.2 Hz, 1H), 6.98 (d, *J* = 3.6 Hz, 1H), 6.91 (d, *J* = 8.6 Hz, 1H), 6.80 (d, *J* = 3.6 Hz, 1H). ^13^C NMR (101 MHz, methanol-*d*_4_) δ 190.5, 163.7, 158.9, 151.7, 138.5, 133.1 (2C), 132.3, 131.2 (2C), 130.7, 128.8, 128.3, 121.6, 121.6, 119.7, 118.3, 117.9, 107.9. HRMS (TOF, ES^–^): *m*/*z* calcd for C_20_H_12_O_5_Br (M–H^+^) 410.9868, found 410.9856. HPLC (method I) (λ = 254 nm), 98.1%; (λ = 214 nm), 98.2%; ^*t*^R = 14.63 min (column 1).

##### (*E*)-2-Hydroxy-5-{5-[3-(4-iodophenyl)-3-oxoprop-1-en-1-yl]­furan-2-yl}­benzoic Acid (**12**)

General conditions for hydrolysis of methyl salicylates, using **34-I** (90 mg, 0.19 mmol, 1 equiv) FCC purification: Gradient elution using petrol ether/DCM/MeOH (49:49:2 → 45:45:10) acidified with 1% AcOH. Reddish-orange solid (94% yield). ^1^H NMR (400 MHz, methanol-*d*_4_) δ 8.39 (d, *J* = 2.3 Hz, 1H), 7.99 (pdt, *J* = 8.6, 2.5 Hz, 2H), 7.79 (dd, *J* = 8.59, 3.14 Hz, 1H), 7.70 (pdt, *J* = 8.6 Hz, 2.4 Hz, 2H), 7.60 (d, *J* = 15.2 Hz, 1H), 7.51 (d, *J* = 15.2 Hz, 1H), 6.98 (d, *J* = 3.6 Hz, 1H), 6.90 (d, *J* = 8.6 Hz, 1H), 6.80 (d, *J* = 3.6 Hz, 1H). ^13^C NMR (101 MHz, methanol-*d*_4_) δ 190.5, 163.7, 158.9, 151.7, 138.5, 133.1, 132.3, 131.2, 130.7, 128.8, 128.2, 121.6, 121.6, 119.7, 118.3, 117.9, 107.9. HRMS: *m*/*z* calcd. for [M-H] C_20_H_12_O_5_I 458.9730; found, 458.9760 (deviation −8.0 ppm). HPLC (method G) (λ = 254 nm), 95.9%; (λ = 214 nm), 97.2%; ^*t*^R = 4.53 min (column 2).

##### (*E*)-2-Hydroxy-5-{5-[3-(4-methoxyphenyl)-3-oxoprop-1-en-1-yl]-2-furanyl}­benzoic Acid (**13**)

General conditions for aldol condensation (protocol B) using 4-methoxyacetophenone (43 mg, 0.26 mmol, 1.2 equiv) and 10 M NaOH aqueous solution (650 μL, 6.5 mmol, 30 equiv). Reaction time: overnight. PLC purification: DCM/AcOH (99:1). Red-orange solid (74 mg, 0.20 mmol, 94% yield). Mp, decomposes at approximately 180 °C. ^1^H NMR (500 MHz, MeOD) δ 8.38 (s, 1H), 8.09 (d, *J* = 8.3 Hz, 2H), 7.78 (d, *J* = 8.5 Hz, 1H), 7.57 (m, 2H), 7.05 (d, *J* = 8.3 Hz, 2H), 6.92 (t, *J* = 5.6 Hz, 2H), 6.78 (d, *J* = 3.5 Hz, 1H), 3.89 (s, 3H). ^13^C NMR (126 MHz, MeOD) δ 190.2, 165.3, 163.8, 158.5, 151.8, 132.2, 131.9 (2C), 131.3, 130.7, 128.2, 121.7, 120.7, 118.3, 118.2, 115.0 (2C), 107.7, 56.1. HRMS (TOF, ES^–^): *m*/*z* calcd for C_21_H_13_O_6_ (M - H^+^) 363.0869, found 363.0843. HPLC (method I) (λ = 254 nm), 95.9%; (λ = 214 nm), 96.4%; ^*t*^R = 11.50 min (column 1).

##### (*E*)-2-Hydroxy-5-{5-{3-(4-(methylthio)­phenyl)-3-oxoprop-1-en-1-yl}­furan-2-yl}­benzoic Acid (**14**)

General conditions for hydrolysis of methyl salicylates, using **34-SMe** (41 mg, 0.1 mmol, 1 equiv). FCC purification: Gradient elution using petrol ether/DCM/MeOH (49:49:2 → 45:45:10) acidified with 1% AcOH. Reddish-orange solid (85% yield). ^1^H NMR (500 MHz, methanol-*d*_4_) δ 8.39 (d, *J* = 2.3 Hz, 1H), 8.01 (d, *J* = 8.5 Hz, 2H), 7.79 (dd, *J* = 8.6, 2.3 Hz, 1H), 7.56 (d, *J* = 3.7 Hz, 2H), 7.36 (d, *J* = 8.5 Hz, 2H), 6.95 (d, *J* = 3.6 Hz, 1H), 6.91 (d, *J* = 8.6 Hz, 1H), 6.79 (d, *J* = 3.6 Hz, 1H), 2.55 (s, 3H). ^13^C NMR (126 MHz, methanol-*d*_4_) δ 190.6, 163.6, 158.6, 151.9, 147.8, 135.6, 131.7, 130.8, 130.0, 128.2, 126.1, 121.8, 121.1, 119.4, 118.3, 118.2, 107.8, 14.6. HRMS: *m*/*z* calcd. for [M-H] C_21_H_15_O_5_S 379.0640; found, 379.0662 (deviation 5.8 ppm). HPLC (method E) (λ = 254 nm), 98.4%; (λ = 214 nm), 99.2%; ^*t*^R = 8.41 min (column 2).

##### (*E*)-5-{5-{3-[4-(*N,N*-Dimethylamino)­phenyl]-3-oxoprop-1-en-1-yl}­furan-2-yl}-2-hydroxybenzoic Acid (**15**)

General conditions for aldol condensation (protocol A) using **32** (130 mg, 0.5 mmol, 1 equiv), *p*-(*N*,*N*-dimethylamino)­acetophenone (129 mg, 0.79 mmol, 1.5 equiv), NaOH 10 N (0.53 mL, 5.3 mmol, 10 equiv), DCM (1 mL) and MeOH (5 mL). FCC purification: Gradient elution using petrol ether/DCM/MeOH (49:49:2 → 45:45:10) acidified with 1% AcOH. Reddish-orange solid (83% yield). ^1^H NMR (500 MHz, methanol-*d*_4_) δ 8.39 (d, *J* = 2.2 Hz, 1H), 8.01 (d, *J* = 9.0 Hz, 2H), 7.79 (dd, *J* = 8.6, 2.2 Hz, 1H), 7.59 (d, *J* = 15.2 Hz, 1H), 7.51 (d, *J* = 15.2 Hz, 1H), 6.91 (d, *J* = 8.6 Hz, 1H), 6.89 (d, *J* = 3.5 Hz, 1H), 6.80–6.76 (m, 3H), 3.09 (s, 6H). ^13^C NMR (126 MHz, methanol-*d*_4_) δ 189.5, 163.5, 158.1, 155.4, 152.2, 132.0, 130.7, 130.2, 128.1, 126.8, 121.9, 119.9, 118.9, 118.3, 112.1, 107.6, 40.1. HRMS: *m*/*z* calcd. for [M + H] C_22_H_20_NO_5_ 378.1341; found, 378.1339 (deviation −0.5 ppm). HPLC (method E) (λ = 254 nm), 94.6%; (λ = 214 nm), 93.2%; ^*t*^R = 6.92 min (column 2).

##### (*E*)-2-Hydroxy-5-{5-[3-oxo-3-(*p*-tolyl)­prop-1-en-1-yl]­furan-2-yl}­benzoic Acid (**16**)

General conditions for hydrolysis of methyl salicylates, using **34-Me** (110 mg, 0.3 mmol, 1 equiv) and pyridine (5 mL). FCC purification: Gradient elution using petrol ether/DCM/MeOH (49:49:2 → 45:45:10) acidified with 1% AcOH. Reddish-orange solid (86% yield). ^1^H NMR (500 MHz, DMSO-*d*_6_) δ 8.17 (d, *J* = 2.4 Hz, 1H), 8.01 (d, *J* = 8.2 Hz, 2H), 7.85 (dd, *J* = 8.5, 2.2 Hz, 1H), 7.56 (s, 2H), 7.39 (d, *J* = 8.1 Hz, 2H), 7.17 (d, *J* = 3.6 Hz, 1H), 6.96 (d, *J* = 3.6 Hz, 1H), 6.84 (d, *J* = 8.6 Hz, 1H), 2.41 (s, 3H). ^13^C NMR (126 MHz, DMSO-*d*_6_) δ 187.9, 171.0, 159.1, 149.8, 143.3, 135.3, 130.0, 129.4, 129.4, 128.4, 126.3, 120.4, 117.3, 117.0, 106.8, 21.2. HRMS: *m*/*z* calcd. for C_21_H_15_O_5_ 347.0919; found, 347.0887 (deviation −9.2 ppm). HPLC (method E) (λ = 254 nm), 98.2%; (λ = 214 nm), 100%; ^*t*^R = 8.10 min (column 2).

##### (*E*)-2-Hydroxy-5-{5-{3-[4-(trifluoromethyl)­phenyl]-3-oxoprop-1-en-1-yl}­furan-2-yl}-benzoic Acid (**17**)

General conditions for aldol condensation (protocol B) using 4-trifluoromethylacetophenone (51 mg, 0.26 mmol, 1.2 equiv) and 10 M NaOH aqueous solution (65 μL, 0.65 mmol, 3 equiv). Reaction time: 3 h. PLC purification: DCM/petroleum ether/AcOH (90:9:1). Red solid (62 mg, 0.15 mmol, 71% yield). mp, decomposes at approximately 180 °C. ^1^H NMR (400 MHz, methanol-*d*_4_) δ 8.34 (s, 1H), 8.17 (d, *J* = 7.9 Hz, 2H), 7.80 (d, *J* = 8.1 Hz, 3H), 7.57 (d, *J* = 15.2 Hz, 1H), 7.46 (d, *J* = 15.2 Hz, 1H), 6.95 (s, 1H), 6.91 (d, *J* = 8.6 Hz, 1H), 6.78 (s, 1H). ^13^C NMR (101 MHz, methanol-*d*_4_) δ 190.4, 174.9, 163.6, 158.6, 151.7, 142.6, 134.8 (q, *J* = 32 Hz), 132.7, 131.4, 130.4, 129.9 (2C), 128.1, 126.7 (q, *J* = 3.9 Hz, 2C), 125.2 (q, *J* = 272 Hz), 121.9, 121.8, 118.5, 118.0, 108.2. HRMS (TOF, ES^–^): *m*/*z* calcd for C_21_H_12_O_5_F_3_ (M – H^+^) 401.0637, found 401.0661. HPLC (method J) (λ = 254 nm), 98.8%; (λ = 214 nm), 97.8%; ^*t*^R = 14.29 min (column 1).

##### (*E*)-5-{5-[3-(4-Ethylphenyl)-3-oxoprop-1-en-1-yl]­furan-2-yl}-2-hydroxybenzoic Acid (**18**)

General conditions for aldol condensation (protocol A) using **32** (150 mg, 0.6 mmol, 1 equiv), *p*-ethylacetophenone, (180 μL, 1.2 mmol, 2 equiv), NaOH 10 N (0.3 mL, 3 mmol, 5 equiv), DCM (0.5 mL) and MeOH (10 mL). FCC purification: Gradient elution using petrol ether/DCM/MeOH (49:49:2 → 45:45:10) acidified with 1% AcOH. Reddish-orange solid (52% yield). ^1^H NMR (400 MHz, methanol-*d*_4_) δ 8.30 (d, *J* = 2.3 Hz, 1H), 7.99 (d, *J* = 8.3 Hz, 2H), 7.95 (dd, *J* = 8.7, 2.3 Hz, 1H), 7.57 (d, *J* = 3.8 Hz, 2H), 7.39 (d, *J* = 8.3 Hz, 2H), 7.03 (d, *J* = 8.7 Hz, 1H), 6.98 (d, *J* = 3.6 Hz, 1H), 6.86 (d, *J* = 3.6 Hz, 1H), 2.75 (q, *J* = 7.6 Hz, 2H), 1.28 (t, *J* = 7.6 Hz, 4H). ^13^C NMR (101 MHz, methanol-*d*_4_) δ 191.5, 163.6, 157.5, 152.2, 151.5, 137.2, 132.6, 131.7, 129.8, 129.3, 127.6, 122.7, 120.6, 119.2, 119.1, 108.5, 29.9, 15.7. HRMS: *m*/*z* calcd. for C_22_H_19_O_5_ 363.1232; found, 363.1242 (deviation +2.8 ppm). HPLC (method G) (λ = 254 nm), 100%; (λ = 214 nm), 100%; ^*t*^R = 3.54 min (column 2).

##### (*E*)-2-Hydroxy-5-{5-[3-(4-isopropylphenyl)-3-oxoprop-1-en-1-yl]­furan-2-yl}­benzoic Acid (**19**)

General conditions for hydrolysis of methyl salicylates, using **34-iPr** (150 mg, 0.6 mmol, 1 equiv). FCC purification: Gradient elution using petrol ether/DCM/MeOH (49:49:2 → 45:45:10) acidified with 1% AcOH. Reddish-orange solid (89% yield). ^1^H NMR (400 MHz, methanol-*d*_4_) δ 8.30 (d, *J* = 2.2 Hz, 1H), 8.00 (d, *J* = 8.4 Hz, 2H), 7.93 (dd, *J* = 8.7, 2.3 Hz, 1H), 7.56 (d, *J* = 3.8 Hz, 2H), 7.41 (d, *J* = 8.2 Hz, 2H), 7.02 (d, *J* = 8.7 Hz, 1H), 6.97 (d, *J* = 3.6 Hz, 1H), 6.85 (d, *J* = 3.6 Hz, 1H), 3.00 (p, *J* = 6.9 Hz, 1H), 1.30 (d, *J* = 6.9 Hz, 6H). ^13^C NMR (101 MHz, methanol-*d*_4_) δ 191.5, 163.6, 157.5, 156.0, 152.2, 137.3, 132.5, 131.7, 129.8, 127.9, 127.6, 122.6, 120.7, 119.1, 119.0, 108.4, 35.5, 24.1. HRMS: *m*/*z* calcd. for C_23_H_19_O_5_ 375.1232; found, 375.1219 (deviation −3.5 ppm). HPLC (method G) (λ = 254 nm), 96.2%; (λ = 214 nm), 97.9%; ^*t*^R = 4.28 min (column 2).

##### (*E*)-5-{5-[3-(4-Butylphenyl)-3-oxoprop-1-en-1-yl]­furan-2-yl}-2-hydroxybenzoic Acid (**20**)

General conditions for hydrolysis of methyl salicylates, using **34-Bu** (83 mg, 0.20 mmol, 1 equiv) and pyridine (5 mL). FCC purification gradient elution using petrol ether/DCM/MeOH (49:49:2 → 45:45:10) acidified with 1% AcOH. Reddish-orange solid (95% yield). ^1^H NMR (500 MHz, methanol-*d*_4_) δ 8.29 (d, *J* = 2.2 Hz, 1H), 7.96 (d, *J* = 8.3 Hz, 2H), 7.90 (dd, *J* = 8.7, 2.3 Hz, 1H), 7.54 (d, *J* = 9.5 Hz, 1H), 7.34 (d, *J* = 8.3 Hz, 2H), 6.99 (d, *J* = 8.7 Hz, 1H), 6.95 (d, *J* = 3.6 Hz, 1H), 6.82 (d, *J* = 3.6 Hz, 1H), 2.72–2.66 (m, 2H), 1.68–1.58 (m, 2H), 1.38 (h, *J* = 7.4 Hz, 3H), 0.95 (t, *J* = 7.4 Hz, 3H). ^13^C NMR (126 MHz, methanol-*d*_4_) δ 191.4, 163.5, 152.1, 150.1, 137.1, 132.4, 131.7, 130.5, 129.9, 129.7, 129.7, 127.7, 120.7, 119.0, 119.0, 108.4, 36.7, 34.5, 23.4, 14.2. HRMS: *m*/*z* calcd. for C_24_H_23_O_5_ 391.1545; found; 391.1557 (deviation +3.1 ppm). HPLC (method G) (λ = 254 nm), 97.8%; (λ = 214 nm), 98.5%; ^*t*^R = 6.01 min (column 2).

##### (*E*)-5-{5-[3-(4-Hexylphenyl)-3-oxoprop-1-en-1-yl]­furan-2-yl}-2-hydroxybenzoic Acid (**21**)

General conditions for aldol condensation (protocol A) using **32** (150 mg, 0.6 mmol, 1 equiv), *p*-hexylacetophenone (197 μL, 186 mg, 0.9 mmol, 1.5 equiv), NaOH 10 N (0.6 mL, 6 mmol, 10 equiv), DCM (1 mL) and MeOH (5 mL). FCC purification: Gradient elution using petrol ether/DCM/MeOH (49:49:2 → 45:45:10) acidified with 1% AcOH. Reddish-orange solid (55% yield). ^1^H NMR (500 MHz, methanol-*d*_4_) δ 8.29 (d, *J* = 2.1 Hz, 1H), 7.98 (d, *J* = 8.1 Hz, 2H), 7.95 (dd, *J* = 8.7, 2.2 Hz, 1H), 7.60–7.52 (m, 2H), 7.35 (d, *J* = 8.1 Hz, 2H), 7.03 (d, *J* = 8.7 Hz, 1H), 6.97 (d, *J* = 3.6 Hz, 1H), 6.86 (d, *J* = 3.6 Hz, 1H), 2.70 (t, *J* = 7.7 Hz, 2H), 1.66 (p, *J* = 7.5 Hz, 2H), 1.38–1.30 (m, 6H), 0.93–0.88 (m, 3H). ^13^C NMR (126 MHz, methanol-*d*_4_) δ 191.5, 173.2, 163.6, 157.4, 152.2, 150.2, 137.1, 132.7, 131.7, 129.9, 129.7, 127.6, 122.7, 120.6, 119.1, 119.1, 114.6, 108.5, 37.0, 32.9, 32.3, 30.0, 23.7, 14.4. HRMS: *m*/*z* calcd. for [M + H] C_26_H_27_O_5_ 419.1839; found, 419.1853 (deviation 3.4 ppm). HPLC (method G) (λ = 254 nm), 100%; (λ = 214 nm), 100%; ^*t*^R = 9.83 min (column 2).

##### (*E*)-2-Hydroxy-5-{5-[3-(4-octylphenyl)-3-oxoprop-1-en-1-yl]­furan-2-yl}­benzoic Acid (**22**)

General conditions for aldol condensation (protocol A) using **32** (179 mg, 0.73 mmol, 1 equiv), *p*-octylacetophenone (200 mg, 0.81 mmol, 1.1 equiv), NaOH 10 N (0.7 mL, 7.3 mmol, 10 equiv), THF (1 mL) and MeOH (5 mL). FCC purification: Gradient elution using petrol ether/DCM/MeOH (49:49:2 → 45:45:10) acidified with 1% AcOH. Reddish-orange solid (91% yield). ^1^H NMR (500 MHz, chloroform-*d*) δ 10.71 (s, 1H), 8.31 (s, 1H), 7.99 (d, *J* = 7.4 Hz, 2H), 7.91 (d, *J* = 7.5 Hz, 1H), 7.62 (d, *J* = 15.0 Hz, 1H), 7.50 (d, *J* = 15.1 Hz, 1H), 7.32 (d, *J* = 7.6 Hz, 2H), 7.09 (d, *J* = 8.0 Hz, 1H), 6.75 (d, *J* = 49.6 Hz, 2H), 2.68 (t, *J* = 7.6 Hz, 2H), 1.64 (q, *J* = 7.0 Hz, 2H), 1.36–1.24 (m, 10H), 0.88 (t, *J* = 6.8 Hz, 3H). ^13^C NMR (126 MHz, chloroform-*d*) δ 190.1, 173.3, 162.5, 155.4, 151.2, 148.8, 136.0, 132.9, 130.6, 128.9, 127.0, 122.1, 119.1, 118.9, 118.7, 112.0, 107.6, 36.2, 32.0, 31.3, 29.6, 29.5, 29.4, 22.8, 14.3. HRMS: *m*/*z* calcd. for [M + H] C_28_H_31_O_5_ 447.2152; found, 447.2166 (deviation +3.2 ppm). HPLC (method H) (λ = 254 nm), 100%; (λ = 214 nm), 100%; ^*t*^R = 5.00 min (column 2).

##### (*E*)-5-{5-{3-[4-(6-Chlorohex-1-yn-1-yl)­phenyl]-3-oxoprop-1-en-1-yl}­furan-2-yl}-2-hydroxybenzoic Acid (**23**)

General conditions for aldol condensation (protocol A) using 4-(6-chlorohexyn-1-yl)­acetophenone. FCC purification: Gradient elution using petrol ether/DCM/MeOH (49:49:2 → 45:45:10) acidified with 1% AcOH. Reddish-orange solid (93% yield). ^1^H NMR (500 MHz, methanol-*d*_4_) δ 8.34–8.31 (m, 1H), 8.03 (d, *J* = 8.3 Hz, 2H), 7.95 (dd, *J* = 8.7, 1.8 Hz, 1H), 7.60 (t, *J* = 14.8 Hz, 2H), 7.53 (d, *J* = 8.2 Hz, 2H), 7.03 (d, *J* = 8.7 Hz, 1H), 7.01 (d, *J* = 3.6 Hz, 1H), 6.88 (d, *J* = 3.6 Hz, 1H), 3.66 (t, *J* = 6.5 Hz, 2H), 2.53 (t, *J* = 7.0 Hz, 2H), 1.97 (dt, *J* = 14.6, 6.6 Hz, 2H), 1.79 (p, *J* = 7.1 Hz, 2H). ^13^C NMR (126 MHz, methanol-*d*_4_) δ 190.8, 163.7, 157.8, 152.2, 138.3, 132.8, 132.5, 132.0, 130.1, 129.5, 127.7, 122.6, 121.1, 119.1, 118.7, 108.5, 94.2, 81.6, 45.3, 32.9, 27.0, 19.5. HRMS: *m*/*z* calcd. for C_26_H_22_ClO_5_ 449.1143; found, 449.1150 (deviation +1.6 ppm). HPLC (method F) (λ = 254 nm), 100%; (λ = 214 nm), 100%; ^*t*^R = 9.28 min (column 2).

##### 5-{5-{(*E*)-3-{4-[(*E*)-3-(*tert*-Butoxy)-3-oxoprop-1-en-1-yl]­phenyl}-3-oxoprop-1-en-1-yl}­furan-2-yl}-2-hydroxybenzoic Acid (**24**)

General conditions for aldol condensation (protocol A) using. **32** (81 mg, 0.33 mmol, 1 equiv), the methylketone **33-Acrylate-*t*Bu** (81 mg, 0.33 mmol, 1 equiv), NaOH 10 N (1 mL, 10 mmol, 30 equiv), DCM (1 mL) and MeOH (5 mL). FCC purification: Gradient elution using petrol ether/DCM/MeOH (49:49:2 → 45:45:10) acidified with 1% AcOH. Reddish-orange solid (73% yield). ^1^H NMR (400 MHz, methanol-*d*_4_) δ 8.26 (s, 1H), 8.05 (d, *J* = 7.8 Hz, 2H), 7.92 (d, *J* = 8.4 Hz, 1H), 7.72 (d, *J* = 7.8 Hz, 2H), 7.64–7.54 (m, 2H), 7.49 (d, *J* = 15.2 Hz, 1H), 7.01 (d, *J* = 8.6 Hz, 1H), 6.98 (d, *J* = 2.6 Hz, 1H), 6.84 (d, *J* = 2.6 Hz, 1H), 6.55 (d, *J* = 16.0 Hz, 1H), 1.54 (s, 9H). ^13^C NMR (101 MHz, methanol-*d*_4_) δ 190.8, 167.6, 157.6, 152.2, 143.6, 140.4, 140.2, 132.7, 132.1, 130.1, 129.4, 127.7, 123.5, 122.7, 121.2, 119.1, 118.8, 108.6, 82.1, 28.4. HPLC (method G) (λ = 254 nm), 94.0%; (λ = 214 nm), 97.7%; ^*t*^R = 5.09 min (column 2).

##### (*E*)-5-{5-{3-{4-[3-(*tert*-Butoxy)-3-oxopropyl]­phenyl}-3-oxoprop-1-en-1-yl}­furan-2-yl}-2-hydroxybenzoic Acid (**25**)

General conditions for aldol condensation (protocol A) using **32** (147 mg, 0.6 mmol, 1 equiv), the methylketone **33-Propionate-*t*Bu** (176 mg, 0.7 mmol, 1.15 equiv), NaOH 10 N (3 mL, 30 mmol, 50 equiv), DCM (1 mL) and MeOH (5 mL). FCC purification: Gradient elution using petrol ether/DCM/MeOH (49:49:2 → 45:45:10) acidified with 1% AcOH. Reddish-orange solid (55% yield). ^1^H NMR (400 MHz, methanol-*d*_4_) δ 8.32 (d, *J* = 2.2 Hz, 1H), 8.01 (d, *J* = 8.3 Hz, 2H), 7.95 (dd, *J* = 8.7, 2.2 Hz, 1H), 7.62–7.53 (m, 2H), 7.41 (d, *J* = 8.3 Hz, 2H), 7.02 (d, *J* = 8.7 Hz, 1H), 6.99 (d, *J* = 3.6 Hz, 1H), 6.87 (d, *J* = 3.6 Hz, 1H), 2.99 (t, *J* = 7.5 Hz, 2H), 2.61 (t, *J* = 7.5 Hz, 2H), 1.41 (s, 9H). ^13^C NMR (101 MHz, methanol-*d*_4_) δ 191.4, 173.8, 163.6, 157.7, 152.2, 148.0, 137.6, 132.5, 131.8, 130.0, 129.8, 127.7, 122.6, 120.8, 119.0, 108.4, 81.8, 37.4, 32.1, 28.3. HRMS: *m*/*z* calcd. for C_23_H_17_O_7_ 461.1679; found, 461.1621 (deviation −2.5 ppm). HPLC (method E) (λ = 254 nm), 100%; (λ = 214 nm), 98.7%; ^*t*^R = 10.35 min (column 2).

##### 5-{5-{(*E*)-3-{4-[(*E*)-3-(Adamantan-1-ylmethylamino)-3-oxoprop-1-enyl]­phenyl}-3-oxoprop-1-enyl}­furan-2-yl}-2-hydroxybenzoic Acid (**26**)

Solid compounds **34-I** (100 mg, 0.23 mmol, 1 equiv), **36** (61 mg, 0.28 mmol, 1.2 equiv), Pd­(OAc)_2_ (10 mg, 2 mol %), DABCO (10 mg, 4 mol %) and K_2_CO_3_ (31 mg, 0.23 mol, 1 equiv) were mixed in a sealed vial. The vial was then purged with argon before the addition of anhydrous DMF. The reaction was then stirred at 120 °C for 12 h. After this time, the reaction was let to cool until rt and the solvent was partially removed under vacuum. The residue was filtered and the filtrate was diluted with AcOEt and washed with HCl 1N (x2) and brine. The organic phase was dried on anhydrous MgSO_4_, filtered and evaporated under vacuum. FCC purification: Gradient elution with mixtures petrol ether/DCM/MeOH (50:50:0 → 45:45:10 (1% AcOH) → 0:70:30). Pale orange solid (116 mg, 0.21 mmol, 91%). ^1^H NMR (500 MHz, DMSO-*d*_6_) δ 8.20 (bs, 1H), 8.15 (d, *J* = 8.1 Hz, 2H), 8.03 (m, 1H), 7.95 (d, *J* = 8.4 Hz, 1H), 7.74 (d, *J* = 8.0 Hz, 2H), 7.62 (d, *J* = 16.9 Hz, 1H), 7.59 (d, *J* = 16.0 Hz, 1H), 7.49 (d, *J* = 15.8 Hz, 1H), 7.21 (d, *J* = 3.3 Hz, 1H), 7.04 (d, *J* = 3.0 Hz, 1H), 6.93 (d, *J* = 7.8 Hz, 1H), 6.90 (d, *J* = 15.5 Hz, 1H), 2.93 (d, *J* = 6.2 Hz, 2H), 1.94 (s, 3H), 1.91 (s, 1H), 1.70–1.57 (m, 6H), 1.48 (bs, 6H). ^13^C NMR (126 MHz, DMSO-*d*_6_) δ 187.7, 172.1, 171.1, 164.8, 156.2, 150.0, 139.3, 138.0, 137.3, 130.3, 128.9, 127.8, 126.3, 124.9, 120.7, 117.6, 117.2, 107.3, 50.4, 40.0, 36.5, 33.8, 27.7, 21.1. HRMS: *m*/*z* calcd. for [M + Na]^+^ C_34_H_33_NNaO_6_ 574.2200; found, 574.2174 (deviation +4.6 ppm). HPLC (method G) (λ = 254 nm), 100%; (λ = 214 nm), 96.6%; ^*t*^R = 4.73 min (column 2).

##### 2-Hydroxy-5-{5-{3-oxo-3-[4-(trifluoromethyl)­phenyl]­propyl}­furan-2-yl}­benzoic Acid (**27**)

General conditions for hydrolysis of methyl salicylates, using **37** (40 mg, 0.1 mmol, 1 equiv). FCC purification. Gradient elution using petrol ether/DCM/MeOH (49:49:2 → 45:45:10) acidified with 1% AcOH. Yellow solid (93% yield). ^1^H NMR (400 MHz, acetone-*d*_6_) δ 8.24 (d, *J* = 8.1 Hz, 2H), 8.15 (d, *J* = 2.2 Hz, 1H), 7.86 (d, *J* = 8.2 Hz, 2H), 7.74 (dd, *J* = 8.6, 2.1 Hz, 1H), 6.92 (d, *J* = 8.7 Hz, 1H), 6.60 (d, *J* = 3.2 Hz, 1H), 6.20 (d, *J* = 3.1 Hz, 1H), 3.53 (t, *J* = 7.3 Hz, 2H), 3.12 (t, *J* = 7.3 Hz, 2H). ^13^C NMR (101 MHz, acetone- *d*_6_) δ 198.6, 173.2, 161.9, 155.1, 152.7, 141.0, 134.4 (q, *J* = 25.3 Hz), 131.2, 129.6, 126.5 (q, *J* = 3.8 Hz), 126.0, 123.8, 123.5, 118.4, 115.0, 108.4, 105.5, 37.9, 23.1. HRMS: *m*/*z* calcd. for [M + H] C_21_H_14_O_5_F_3_ 403.0793; found, 403.0798 (deviation 1.2 ppm). HPLC (method E) (λ = 254 nm), 100%; (λ = 214 nm), 100%; ^*t*^R = 10.19 min (column 2).

##### 5-{5-[3-(4-Butylphenyl)-3-oxopropyl]­furan-2-yl}-2-hydroxybenzoic Acid (**28**)

General conditions for hydrolysis of methyl salicylates, using **38** (57 mg, 0.14 mmol, 1 equiv). FCC purification: Gradient elution using petrol ether/DCM/MeOH (49:49:2 → 45:45:10) acidified with 1% AcOH. Clear oil (97% yield). ^1^H NMR (400 MHz, methanol-*d*_4_) δ 8.15 (d, *J* = 2.4 Hz, 1H), 7.97–7.90 (m, 2H), 7.63 (dd, *J* = 8.6, 2.4 Hz, 1H), 7.31 (d, *J* = 8.2 Hz, 2H), 6.88 (d, *J* = 8.6 Hz, 1H), 6.47 (d, *J* = 3.2 Hz, 1H), 6.13 (d, *J* = 3.2 Hz, 1H), 3.40 (t, *J* = 7.3 Hz, 2H), 3.10 (t, *J* = 7.3 Hz, 2H), 2.68 (t, *J* = 7.7 Hz, 2H), 1.66–1.57 (m, 2H), 1.40–1.33 (m, 2H), 0.95 (t, *J* = 7.3 Hz, 3H). ^13^C NMR (101 MHz, methanol-*d*_4_) δ 200.9, 162.0, 155.3, 153.5, 150.3, 135.9, 130.6, 129.8, 129.4, 126.5, 123.8, 118.1, 108.4, 105.2, 37.8, 36.6, 34.5, 23.9, 23.3, 14.2. HRMS: *m*/*z* calcd. for [M + Na] C_24_H_24_NaO_5_ 415.1505; found, 415.1516 (deviation +2.6 ppm). HPLC (method G) (λ = 254 nm), 98.0%; (λ = 214 nm), 95.3%; ^*t*^R = 5.95 min (column 2).

##### 5-{5-[3-(4-Butylphenyl)-3-hydroxypropyl]­furan-2-yl}-2-hydroxybenzoic Acid (**29**)

General conditions for hydrolysis of methyl salicylates, using **39** (37 mg, 0.09 mmol, 1 equiv). FCC purification: Gradient elution using petrol ether/DCM/MeOH (49:49:2 → 45:45:10) acidified with 1% AcOH. Clear oil (96% yield). ^1^H NMR (400 MHz, methanol-*d*_4_) δ 8.10 (d, *J* = 2.3 Hz, 1H), 7.72 (dd, *J* = 8.7, 2.3 Hz, 1H), 7.27 (d, *J* = 8.1 Hz, 2H), 7.15 (d, *J* = 8.0 Hz, 2H), 6.94 (d, *J* = 8.7 Hz, 1H), 6.50 (d, *J* = 3.2 Hz, 1H), 6.08 (d, *J* = 3.2 Hz, 1H), 4.68–4.63 (m, 1H), 2.75–2.68 (m, 2H), 2.61–2.56 (m, 2H), 2.16–2.03 (m, 2H), 1.57 (ddt, *J* = 9.0, 7.6, 3.5 Hz, 2H), 1.38–1.30 (m, 2H), 0.93 (t, *J* = 7.4 Hz, 3H). ^13^C NMR (101 MHz, methanol-*d*_4_) δ 173.4, 162.1, 156.4, 152.8, 143.2, 143.2, 131.7, 129.4, 127.1, 126.0, 124.4, 118.6, 114.1, 108.2, 105.6, 74.2, 38.6, 36.3, 35.0, 25.5, 23.3, 14.3. HRMS: *m*/*z* calcd. for [M – H] C_24_H_25_O_5_ 393.1706; found, 393.1707 (deviation +0.5 ppm). HPLC (method G) (λ = 254 nm), 100%; (λ = 214 nm), 96.4%; ^*t*^R = 5.00 min (column 2).

##### Methyl 5-(5-Formylfuran-2-yl)-2-hydroxybenzoate (**32**)

To a stirred solution of methyl 5-iodosalicylate (**30**) (200 mg, 0.719 mmol, 1 equiv) and 5-formyl-2-furanylboronic acid (**31**) (151 mg, 1.078 mmol, 1.5 equiv) in DMF (10 mL), triethylamine (300 μL, 2.157 mmol, 3 equiv) and Pd­(OAc)_2_ (8 mg, 0.05 equiv) were added. The solvent was bubbled with argon. The reaction mixture was stirred at rt overnight, under Ar. After consumption of the starting material, determined by TLC (petrol ether/AcOEt) (70:30), the reaction mixture was concentrated in a high vacuum rotavapor. The solid residue was dissolved in AcOEt and washed with water and brine. The organic layer was dried over MgSO_4_, filtered and concentrated under reduced pressure. The residue was purified by FCC (gradient elution using petrol ether/AcOEt) (80:20 → 50:50) to yield **31** as a yellowish-orange solid (154 mg, 0.63 mmol, 87% yield). ^1^H NMR (400 MHz, chloroform-*d*) δ 10.99 (s, 1H), 9.61 (s, 1H), 8.31 (d, *J* = 2.3 Hz, 1H), 7.87 (dd, *J* = 8.7, 2.3 Hz, 1H), 7.31 (d, *J* = 3.7 Hz, 1H), 7.05 (d, *J* = 8.7 Hz, 1H), 6.74 (d, *J* = 3.7 Hz, 1H), 4.00 (s, 3H). ^13^C NMR (101 MHz, chloroform-*d*) δ 177.0, 170.2, 162.7, 158.7, 151.9, 132.6, 127.2, 120.8, 118.6, 112.9, 106.8, 52.7. HRMS: *m*/*z* calcd. for [M + H] C_13_H_11_O_5_ 247.0606; found, 247.0593 (deviation −1.1 ppm).

##### *tert*-Butyl (*E*)-3-(4-Acetylphenyl)­acrylate (33-Acrylate-*t*Bu)

Working in a sealed tube in an inert argon atmosphere, 4-bromoacetophenone (100 mg, 0.50 mmol, 1 equiv), palladium­(II) acetate (9 mg, 0.04 mmol, 0.02 equiv), 1,4-diazabicylo­[2,2,2]­octane (DABCO) (9 mg, 0.08 mmol, 0.04 equiv) and potassium carbonate (69 mg, 0.50 mmol, 1 equiv) were dissolved in anhydrous *N,N*-dimethylformamide. After addition of *tert*-butyl acrylate (1.5 equiv), the reaction mixture was heated to 120 °C during 15 h. Once cooled down, the reaction was filtered through Celite and concentrated under reduced pressure. FCC purification was performed by gradient elution using petrol ether:AcOEt (100:0 → 80:20). Yellowish oil (81% yield). ^1^H NMR (500 MHz, chloroform-*d*) δ 7.95 (d, *J* = 8.4 Hz, 2H), 7.63–7.55 (m, 3H), 6.45 (d, *J* = 16.0 Hz, 1H), 2.61 (s, 3H), 1.54 (s, 9H). ^13^C NMR (126 MHz, chloroform-*d*) δ 197.5, 165.9, 142.1, 139.2, 137.9, 129.0, 128.1, 122.9, 81.1, 28.3, 26.8.

##### *tert*-Butyl 3-(4-Acetylphenyl)­propanoate (33-Propionate-*t*Bu)

Compound **33-Acrylate-*t*Bu** (1 equiv) was dissolved in THF and 1 mL of Raney Nickel activated catalyst (50% slurry in water) was then added. Hydrogen (g) was bubbled through the solution during 1h. Once completed, the reaction mixture was filtered through Celite and concentrated under reduced pressure. FCC purification was performed by elution using mixtures of petrol ether and AcOEt. The final product was obtained as a yellowish oil (96% yield). ^1^H NMR (400 MHz, chloroform-*d*) δ 7.88 (d, *J* = 8.2 Hz, 2H), 7.29 (d, *J* = 8.2 Hz, 2H), 2.96 (t, *J* = 7.7 Hz, 2H), 2.57 (s, 3H), 2.56 (t, *J* = 7.6 Hz, 2H), 1.40 (s, 9H) (spectral data agree with the ones reported previously).^61^

##### Methyl 5-{5-{(*E*)-3-{4-[6-(Adamantan-1-ylmethylamino)­hex-1-ynyl]­phenyl}-3-oxoprop-1-enyl}­furan-2-yl}-2-hydroxybenzoate (**34-Ad**)

The solid reagents, **34-I** (640 mg, 1.35 mmol, 1 equiv), bis­(triphenylphosphine)­palladium­(II) dichloride (53 mg, 0.076 mmol, 0.05 equiv) and copper­(I) iodide (24 mg, 0.126 mmol, 0.1 equiv) were mixed in a round-bottom flask and the mixture was purged under argon before the addition of anhydrous THF. Then a 1 M solution of tetrabutylammonium fluoride in hexanes (554 mg, 2.12 mL, 2.12 mmol, 1.6 equiv) and **35** (315 mg, 1.28 mmol, 1 equiv) were added to start the reaction. After 18 h stirring at rt, the reaction was filtered through Celite and concentrated under reduced pressure. FCC purification: Gradient elution with mixtures petrol ether/DCM/MeOH (50:50:2 → 50:50:15). Orange solid, 67% yield (497 mg, 0.86 mmol). ^1^H NMR (500 MHz, chloroform-*d*) δ 10.92 (bb, 1H), 8.20 (d, *J* = 2.3 Hz, 1 H), 7.97 (d, *J* = 8.5 Hz, 2H), 7.87 (dd, *J* = 8.9, 2.4 Hz, 1H), 7.57 (d, *J* = 15.3 Hz, 1H), 7.55 (d, *J* = 8.5 Hz, 2H), 7.42 (d, *J* = 15.3 Hz, 1H), 7.07 (d, J = 8.7 Hz, 1H), 6.80 (d, *J* = 3.5 Hz, 1H), 6.68 (d, *J* = 3.5 Hz, 1H), 4.01 (s, 3H), 3.10 (m, 2H), 2.65 (m, 2H), 2.50 (t, *J* = 7.0 Hz, 2H), 2.14 (m, 2H), 2.02 (bs, 3H), 1.76–1.63 (m, 16H). ^13^C NMR (126 MHz, chloroform-*d*) δ 189.2, 170.3, 162.0, 155.8, 151.0, 137.3, 132.0, 132.0, 130.8, 128.5, 128.3, 126.2, 121.8, 119.3, 118.6, 118.4, 112.9, 107.5, 92.6, 81.4, 59.5, 52.8, 48.9, 40.1, 36.4, 32.9, 28.1, 25.9, 24.4, 19.2. HRMS: *m*/*z* calcd. for [M + H] C_38_H_42_NO_5_ 592.3057; found, 592.3082 (deviation −4.2 ppm).

##### Methyl (*E*)-5-{5-[3-(4-Butylphenyl)-3-oxoprop-1-en-1-yl]­furan-2-yl}-2-hydroxybenzoate (**34-Bu**)

General conditions for aldol condensation (protocol A) using **32** (90 mg, 0.36 mmol), *p*-butylacetophenone (134 μL, 0.73 mmol), NaOH 10 N (1.8 mmol, 5 equiv), DCM (minimum amount for solids dissolution) and MeOH (5 mL). FCC purification: Gradient elution using petrol ether/AcOEt (100:0 → 80:20). Reddish-orange solid (87% yield). ^1^H NMR (400 MHz, chloroform-*d*) δ 10.92 (s, 1H), 8.22 (d, *J* = 2.3 Hz, 1H), 7.98 (d, *J* = 8.2 Hz, 2H), 7.87 (dd, *J* = 8.8, 2.3 Hz, 1H), 7.59 (d, *J* = 15.3 Hz, 1H), 7.48 (d, *J* = 15.3 Hz, 1H), 7.32 (d, *J* = 8.2 Hz, 2H), 7.07 (d, *J* = 8.7 Hz, 1H), 6.79 (d, *J* = 3.6 Hz, 1H), 6.69 (d, *J* = 3.6 Hz, 1H), 4.03 (s, 3H), 2.70 (t, *J* = 7.7 Hz, 2H), 1.65 (m, 2H), 1.38 (m, 2H), 0.95 (t, *J* = 7.3 Hz, 3H). ^13^C NMR (101 MHz, chloroform-*d*) δ 189.8, 170.4, 161.9, 155.6, 151.2, 148.6, 136.2, 132.0, 130.3, 128.8, 128.7, 126.2, 121.9, 119.0, 118.8, 118.6, 112.9, 107.4, 52.8, 35.9, 33.5, 22.5, 14.1. HRMS: *m*/*z* calcd. for [M + H] C_25_H_25_O_5_= 405.1702; found, 405.1689 (deviation −3.2 ppm).

##### Methyl (*E*)-2-Hydroxy-5-{5-{3-oxo-3-[4-(trifluoromethyl)­phenyl]­prop-1-en-1-yl}­furan-2-yl}­benzoate (**34-CF3**)

General conditions for aldol condensation (protocol A) using **32** (114 mg, 0.46 mmol), *p*-trifluoromethylacetophenone (140 μL, 0.69 mmol), NaOH 10 N (460 μL, 4.6 mmol, 10 equiv), DCM (minimum amount for solids dissolution) and MeOH (5 mL). FCC purification: Gradient elution using petrol ether/AcOEt (100:0 → 80:20). Reddish-orange solid (93% yield). ^1^H NMR (400 MHz, chloroform-*d*) δ 10.93 (s, 1H), 8.22 (d, *J* = 2.3 Hz, 1H), 8.12 (d, *J* = 8.1 Hz, 2H), 7.87 (dd, *J* = 8.7, 2.3 Hz, 1H), 7.77 (d, *J* = 8.0 Hz, 2H), 7.60 (d, *J* = 15.3 Hz, 1H), 7.41 (d, *J* = 15.3 Hz, 1H), 7.07 (d, *J* = 8.7 Hz, 1H), 6.85 (d, *J* = 3.6 Hz, 1H), 6.71 (d, *J* = 3.6 Hz, 1H), 4.03 (s, 3H). ^13^C NMR (101 MHz, chloroform-*d*) δ 189.1, 170.2, 162.0, 156.1, 150.7, 141.3 (q, *J* = 1.0 Hz, C), 133.9 (q, *J* = 32.6 Hz, C), 131.9, 131.5, 128.7, 126.2, 125.7 (q, *J* = 3.7 Hz, CH), 123.7 (q, *J* = 272.7 Hz, CF_3_), 121.5, 119.9, 118.5, 118.0, 112.8, 107.5, 52.7. HRMS: *m*/*z* calcd. for [M + H] C_22_H_16_O_5_F_3_ 417.0950; found, 417.0971 (deviation 5.0 ppm).

##### Methyl (*E*)-2-Hydroxy-5-{5-{3-[4-(hydroxymethyl)­phenyl]-3-oxoprop-1-en-1-yl}­furan-2-yl}­benzoate (34-CH_2_OH)

General conditions for aldol condensation (protocol A) using **32** (150 mg, 0.60 mmol), *p*-(hydroxymethyl) acetophenone (136 mg, 0.91 mmol), NaOH 10 N (0.3 mL, 3 mmol, 5 equiv), DCM (minimum amount for solids dissolution) and MeOH (10 mL). FCC purification: Gradient elution using petrol ether/AcOEt (80:20 → 20:80). Reddish-orange solid (83% yield). ^1^H NMR (500 MHz, chloroform-*d*) δ 10.92 (s, 1H), 8.22 (d, *J* = 2.3 Hz, 1H), 8.04 (d, *J* = 8.2 Hz, 2H), 7.87 (dd, *J* = 8.7, 2.3 Hz, 1H), 7.59 (d, *J* = 15.3 Hz, 1H), 7.51 (d, *J* = 8.1 Hz, 2H), 7.46 (d, *J* = 15.3 Hz, 1H), 7.07 (d, *J* = 8.7 Hz, 1H), 6.81 (d, *J* = 3.5 Hz, 1H), 6.69 (d, *J* = 3.5 Hz, 1H), 4.81 (s, 2H), 4.03 (s, 3H). ^13^C NMR (126 MHz, chloroform-*d*) δ 189.8, 170.3, 162.0, 155.7, 151.0, 145.9, 137.7, 132.0, 130.7, 128.9, 126.9, 126.2, 121.8, 119.2, 118.7, 118.6, 112.9, 107.5, 64.9, 52.8. HRMS: *m*/*z* calcd. for [M + H] C_22_H_19_O_6_ 379.1182; found, 379.1194 (deviation 3.2 ppm).

##### Methyl (*E*)-5-{5-[3-(4-Cyanophenyl)-3-oxoprop-1-en-1-yl]­furan-2-yl}-2-hydroxybenzoate (**34-CN**)

General conditions for aldol condensation (protocol A) using **32** (95 mg, 0.38 mmol), *p*-cyanoacetophenone (85 mg, 0.58 mmol), NaOH 10 N (190 μL, 1.9 mmol, 5 equiv), DCM (minimum amount for solids dissolution) and MeOH (5 mL). FCC purification: Gradient elution using petrol ether/AcOEt (100:0 → 70:30). Reddish-orange solid (90% yield). ^1^H NMR (500 MHz, chloroform-*d*) δ 10.94 (s, 1H), 8.23 (d, *J* = 2.4 Hz, 1H), 8.11 (d, *J* = 10.0 Hz, 2H), 7.88 (dd, *J* = 8.8, 2.3 Hz, 1H), 7.81 (d, *J* = 10.0 Hz, 2H), 7.62 (d, *J* = 15.2 Hz, 1H), 7.39 (d, *J* = 15.3 Hz, 1H), 7.08 (d, *J* = 8.8 Hz, 1H), 6.88 (d, *J* = 3.6 Hz, 1H), 6.73 (d, *J* = 3.6 Hz, 1H), 4.03 (s, 3H). ^13^C NMR (126 MHz, chloroform-*d*) δ 188.7, 170.3, 162.2, 156.5, 150.7, 141.9, 132.6, 132.1, 132.0, 128.9, 126.4, 121.6, 120.5, 118.7, 118.3, 117.6, 115.9, 112.9, 107.8, 52.8. HRMS: *m*/*z* calcd. for [M + H] C_22_H_14_NO_5_ 393.0767; found, 393.0797 (deviation −7.6 ppm).

##### Methyl (*E*)-5-{5-[3-(4-Ethylphenyl)-3-oxoprop-1-en-1-yl]­furan-2-yl}-2-hydroxybenzoic Acid (**34-Et**)

General conditions for aldol condensation (protocol A) using **32** (150 mg, 0.60 mmol), *p*-ethylacetophenone (180 μL, 1.2 mmol, 2 equiv), NaOH 10 N (0.3 mL, 3 mmol, 5 equiv), DCM (minimum amount for solids dissolution) and MeOH (10 mL). FCC purification: Gradient elution using petrol ether/AcOEt (100:0 → 70:30). Reddish-orange solid (43% yield). ^1^H NMR (400 MHz, chloroform-*d*) δ 10.91 (s, 1H), 8.23 (d, *J* = 2.3 Hz, 1H), 7.99 (d, *J* = 8.2 Hz, 2H), 7.87 (dd, *J* = 8.7, 2.3 Hz, 1H), 7.59 (d, *J* = 15.3 Hz, 1H), 7.48 (d, *J* = 15.4 Hz, 1H), 7.34 (d, *J* = 8.3 Hz, 2H), 7.08 (d, *J* = 8.7 Hz, 1H), 6.79 (d, *J* = 3.6 Hz, 1H), 6.69 (d, *J* = 3.5 Hz, 1H), 4.03 (s, 3H), 2.74 (q, *J* = 7.6 Hz, 2H), 1.29 (t, *J* = 7.6 Hz, 3H). ^13^C NMR (101 MHz, chloroform-*d*) δ 189.8, 170.4, 162.0, 155.6, 151.2, 149.8, 136.2, 132.0, 130.4, 128.8, 128.3, 126.2, 121.9, 119.0, 118.8, 118.6, 112.9, 107.4, 77.4, 52.8, 29.2, 15.4. HRMS: *m*/*z* calcd. for [M + H] C_23_H_21_O_5_ 377.1389; found, 377.1385 (deviation −1.1 ppm).

##### Methyl (*E*)-5-{5-[3-(4-Hexylphenyl)-3-oxoprop-1-en-1-yl]­furan-2-yl}-2-hydroxybenzoate (**34-Hex**)

General conditions for aldol condensation (protocol A) using **32** (150 mg, 0.60 mmol), *p*-hexylacetophenone (197 μL, 0.9 mmol, 1.5 mmol equiv), NaOH 10 N (0.6 mL, 6 mmol, 10 equiv), DCM (minimum amount for solids dissolution) and MeOH (5 mL). FCC purification: Gradient elution using petrol ether/AcOEt) (100:0 → 70:30). Reddish-orange solid (36% yield). ^1^H NMR (500 MHz, chloroform-*d*) δ 10.92 (s, 1H), 8.21 (d, *J* = 2.3 Hz, 1H), 7.98 (d, *J* = 8.2 Hz, 2H), 7.86 (dd, *J* = 8.7, 2.3 Hz, 1H), 7.58 (d, *J* = 15.3 Hz, 1H), 7.47 (d, *J* = 15.3 Hz, 1H), 7.32 (d, *J* = 8.0 Hz, 2H), 7.07 (d, *J* = 8.7 Hz, 1H), 6.78 (d, *J* = 3.5 Hz, 1H), 6.68 (d, *J* = 3.5 Hz, 1H), 4.02 (s, 3H), 2.69 (t, *J* = 7.7 Hz, 2H), 1.65 (m, 2H), 1.37–1.29 (m, 6H), 0.91–0.87 (m, 3H). ^13^C NMR (126 MHz, chloroform-*d*) δ 189.7, 170.3, 161.9, 155.5, 151.1, 148.6, 136.1, 132.0, 130.3, 128.8, 128.7, 126.1, 121.9, 118.9, 118.8, 118.5, 112.8, 107.4, 52.7, 36.2, 31.8, 31.3, 29.1, 22.7, 14.2. HRMS: *m*/*z* calcd. for [M + H] C_27_H_29_O_5_ 433.2010; found 433.1987 (deviation +5.1 ppm).

##### Methyl (*E*)-2-Hydroxy-5-{5-[3-(4-iodophenyl)-3-oxoprop-1-en-1-yl]­furan-2-yl}­benzoate (**34-I**)

General conditions for aldol condensation (protocol A) using **32** (110 mg, 0.45 mmol), *p*-iodoacetophenone (220 mg, 0.89 mmol), NaOH 10 N (0.45 mL, 4.5 mmol, 10 equiv), DCM (minimum amount for solids dissolution) and MeOH (5 mL). FCC purification: Gradient elution using petrol ether/AcOEt (95:5 → 70:30). Reddish-orange solid (200 mg, 0.42 mmol, 94% yield). ^1^H NMR (400 MHz, chloroform-*d*) δ 10.93 (s, 1H), 8.22 (d, *J* = 2.3 Hz, 1H), 7.89–7.84 (m, 3H), 7.75 (dt, *J* = 8.0 Hz, *J* = 4.0 Hz, 2H), 7.59 (d, *J* = 15.3 Hz, 1H), 7.39 (d, *J* = 15.3 Hz, 1H), 7.07 (d, *J* = 8.7 Hz, 1H), 6.82 (d, *J* = 3.6 Hz, 1H), 6.70 (d, *J* = 3.6 Hz, 1H), 4.03 (s, 3H). ^13^C NMR (101 MHz, chloroform-*d*) δ 189.3, 170.3, 162.1, 156.0, 150.9, 138.0, 137.8, 132.0, 131.1, 130.0, 126.3, 121.7, 119.6, 118.6, 118.1, 112.9, 107.6, 100.5, 52.8. HRMS: *m*/*z* calcd. for [M-H] C_21_H_14_O_5_I 472.9886; found, 472.9848 (deviation: 6.5 ppm).

##### Methyl (*E*)-2-Hydroxy-5-{5-[3-(4-isopropylphenyl)-3-oxoprop-1-en-1-yl]­furan-2-yl}­benzoate (**34-iPr**)

General conditions for aldol condensation (protocol A) using **32** (150 mg, 0.60 mmol), *p*-isopropylacetophenone (250 mg, 1.54 mmol, 2.6 equiv), NaOH 10 N (0.5 mL, 5 mmol, 5 equiv), DCM (minimum amount for solids dissolution) and MeOH (10 mL). FCC purification: Gradient elution using petrol ether/AcOEt (100:0 → 70:30). Reddish-orange solid (91% yield). ^1^H NMR (500 MHz, chloroform-*d*) δ 10.92 (s, 1H), 8.22 (d, *J* = 2.3 Hz, 1H), 8.00 (d, *J* = 8.2 Hz, 2H), 7.87 (dd, *J* = 8.7, 2.3 Hz, 1H), 7.59 (d, *J* = 15.3 Hz, 1H), 7.48 (d, *J* = 15.3 Hz, 1H), 7.37 (d, *J* = 8.3 Hz, 2H), 7.08 (d, *J* = 8.7 Hz, 1H), 6.80 (d, *J* = 3.6 Hz, 1H), 6.69 (d, *J* = 3.5 Hz, 1H), 4.03 (s, 3H), 3.00 (m, 1H), 1.31 (s, 3H), 1.29 (s, 3H). ^13^C NMR (126 MHz, chloroform-*d*) δ 189.8, 170.4, 161.9, 155.6, 154.4, 151.2, 136.3, 132.0, 130.4, 128.9, 126.9, 126.2, 121.9, 119.0, 118.9, 118.6, 112.9, 107.4, 52.8, 34.4, 23.9. HRMS: *m*/*z* calcd. for [M + H] C_24_H_23_O_5_ 391.1545; found, 391.1557 (deviation +3.1).

##### Methyl (*E*)-2-Hydroxy-5-{5-[3-oxo-3-(*p*-tolyl)­prop-1-en-1-yl]­furan-2-yl}­benzoate (**34-Me**)

General conditions for aldol condensation (protocol A) using **32** (150 mg, 0.60 mmol), *p*-methylacetophenone (160 μL, 1.2 mmol), DCM (minimum amount for solids dissolution) and MeOH (10 mL). FCC purification: Gradient elution using petrol ether/AcOEt (100:0 → 50:50). Reddish-orange solid (96% yield). ^1^H NMR (500 MHz, chloroform-*d*) δ 10.92 (s, 1H), 8.22 (d, *J* = 2.3 Hz, 1H), 7.96 (d, *J* = 8.2 Hz, 2H), 7.87 (dd, *J* = 8.7, 2.3 Hz, 1H), 7.58 (d, *J* = 15.4 Hz, 1H), 7.47 (d, *J* = 15.3 Hz, 1H), 7.32 (d, *J* = 7.8 Hz, 2H), 7.07 (d, *J* = 8.7 Hz, 1H), 6.79 (d, *J* = 3.5 Hz, 1H), 6.69 (d, *J* = 3.5 Hz, 1H), 4.03 (s, 3H), 2.45 (s, 3H). ^13^C NMR (126 MHz, chloroform-*d*) δ 189.7, 170.4, 161.9, 155.6, 151.1, 143.6, 135.9, 132.0, 130.4, 129.5, 128.7, 126.1, 121.9, 118.9, 118.8, 118.5, 112.9, 107.4, 52.8, 21.8. HRMS: *m*/*z* calcd. for [M + H] C_22_H_19_O_5_ 363.1232; found, 363.1242 (deviation 2.8 ppm).

##### Methyl (*E*)-2-Hydroxy-5-{5-{3-[4-(methylthio)­phenyl]-3-oxoprop-1-en-1-yl}­furan-2-yl}­benzoate (**34-SMe**)

General conditions for aldol condensation (protocol A) using **32** (95 mg, 0.38 mmol), *p*-(methylthio)­acetophenone (96 mg, 0.58 mmol), NaOH 10 N (0.19 mL, 1.9 mmol, 5 equiv), DCM (minimum amount for solids dissolution) and MeOH (5 mL). FCC purification: Gradient elution using petrol ether/AcOEt (100:0 → 80:20). Reddish-brown solid (89% yield). ^1^H NMR (500 MHz, chloroform-*d*) δ 10.92 (s, 1H), 8.20 (d, *J* = 2.3 Hz, 1H), 7.98 (d, *J* = 8.6 Hz, 2H), 7.86 (dd, *J* = 8.7, 2.3 Hz, 1H), 7.58 (d, *J* = 15.2 Hz, 1H), 7.45 (d, *J* = 15.3 Hz, 1H), 7.35–7.29 (m, 2H), 7.06 (d, *J* = 8.7 Hz, 1H), 6.79 (d, *J* = 3.6 Hz, 1H), 6.68 (d, *J* = 3.5 Hz, 1H), 4.02 (s, 3H), 2.54 (s, 3H). ^13^C NMR (126 MHz, chloroform-*d*) δ 188.8, 170.3, 161.9, 155.6, 151.1, 145.6, 134.7, 132.0, 130.4, 129.0, 126.1, 125.2, 121.8, 119.0, 118.5, 118.5, 112.8, 107.5, 52.8, 15.0. HRMS: *m*/*z* calcd. for [M + H] C_22_H_17_O_5_S 393.0767; found, 393.0797 (deviation −7.6 ppm).

##### *N*-(Adamantan-1-ylmethyl)­hex-5-yn-1-amine (**35**)

Adamantan-1-ylmethyl)­amine (268 mg, 270 μL, 1.62 mmol, 2 equiv) and potassium carbonate (112 mg, 0.81 mmol, 1 equiv) were introduced in a dry two-necked round-bottom flask and the mixture was purged with argon. Then, anhydrous acetonitrile (10 mL) was added, followed by 6-iodohexyne (168 mg, 106 μL, 0.81 mmol, 1 equiv). The reaction was then diluted with an additional volume of anhydrous acetonitrile (10 mL) and let to stir at 80 °C (reflux), under argon, for 18 h.^62^ The reaction was then cooled down to rt and the solvents were evaporated under vacuum. The residue was suspended in DCM and the organic phase was washed with an aqueous solution of NaOH (5 M). The organic phase was dried over anhydrous MgSO_4_, filtered and evaporated under vacuum. FCC purification (automatic): Gradient elution with mixtures DCM/MeOH (99:1 → 90:10). White solid, 80% yield (160 mg, 0.65 mmol). ^1^H NMR (500 MHz, chloroform-*d*) δ 3.34 (bb, 1H), 2.70 (t, *J* = 7.5 Hz, 2H), 2.32 (s, 2H), 2.21 (m, 2H), 1.98–1.93 (m, 4H), 1.73–1.60 (m, 8H), 1.59–1.51 (m, 8H). ^13^C NMR (126 MHz, chloroform-*d*) δ 84.3, 68.7, 62.2, 50.1, 40.8, 37.1, 33.3, 28.5, 28.0, 26.3, 18.4.

##### *N*-(Adamantan-1-ylmethyl)­acrylamide (**36**)

A solution of (adamantan-1-ylmethyl)­amine (200 mg, 1.21 mmol, 1 equiv) and diisopropylethylamine (312 mg, 2.42 mmol, 2 equiv) in anhydrous THF (8 mL), was prepared under argon. The solution was cooled to 0 °C and acryloyl chloride (120 mg, 1.33 mmol, 1.1 equiv) was added dropwise. The reaction was then warmed at rt and let to stir during 20 min. The reaction was then quenched by addition of a saturated solution of sodium bicarbonate (3 mL) followed by addition of water (3 mL). The organic phase was then separated, the THF was evaporated under vacuum and the residue was dissolved in AcOEt. The new organic phase was washed with HCl (5%) and brine, dried on anhydrous MgSO_4_, filtered and evaporated under vacuum. FCC purification (automatic): Gradient elution with mixtures petrol ether/AcOEt (90:10 → 20:80). White solid (quantitative yield). ^1^H NMR (500 MHz, chloroform-*d*) δ 6.28 (dd, *J* = 17.0, 1.5 Hz, 1H), 6.12 (dd, *J* = 16.9, 10.3 Hz, 1H), 5.64 (dd, *J* = 10.2, 1.5 Hz, 1H), 3.04 (d, *J* = 6.4 Hz, 2H), 1.98 (bs, 3H), 1.74–1.60 (m, 6H), 1.50 (d, *J* = 2.7 Hz, 6H). ^13^C NMR (126 MHz, chloroform-*d*) δ 165.89 (C), 131.17 (CH), 126.43 (CH_2_), 51.14 (CH_2_), 40.37 (CH_2_), 37.04 (CH_2_), 34.01 (C), 28.34 (CH).

##### Methyl 2-Hydroxy-5-{5-{3-oxo-3-[4-(trifluoromethyl)­phenyl]­propyl}­furan-2-yl}­benzoate (**37**)

General conditions for selective double bond hydrogenation in α,β-unsaturated ketones using **34-CF3** (76 mg, 0.18 mmol, 1 equiv) in THF (5 mL). FCC purification (petrol ether/AcOEt) (100:0 → 90:10). Clear oil (95% yield). ^1^H NMR (400 MHz, chloroform-*d*) δ 10.75 (s, 1H), 8.08 (d, *J* = 8.1 Hz, 2H), 8.05 (d, *J* = 2.3 Hz, 1H), 7.73 (d, *J* = 8.2 Hz, 2H), 7.68 (dd, *J* = 8.7, 2.3 Hz, 1H), 6.98 (d, *J* = 8.8 Hz, 1H), 6.44 (d, *J* = 3.3 Hz, 1H), 6.13 (d, *J* = 3.3 Hz, 1H), 3.98 (s, 3H), 3.41 (t, *J* = 7.4 Hz, 2H), 3.17 (t, *J* = 7.4 Hz, 2H). ^13^C NMR (101 MHz, chloroform-*d*) δ 197.8, 170.5, 160.8, 153.9, 151.9, 139.5, 134.6 (q, *J* = 32.7 Hz), 131.2, 128.5, 125.9 (q, *J* = 3.7 Hz), 124.7, 123.7 (q, *J* = 272.7 Hz), 123.1, 118.2, 112.6, 107.9, 104.9, 52.6, 37.6, 22.8.

##### Methyl 5-{5-[3-(4-Butylphenyl)-3-oxopropyl]­furan-2-yl}-2-hydroxybenzoate (**38**)

General conditions for selective double bond hydrogenation in α,β-unsaturated ketones, using **34-Bu** (147 mg, 0.36 mmol, 1 equiv). FCC purification (petroleum ether/AcOEt) (100:0 → 70:30). Clear oil (94% yield). ^1^H NMR (500 MHz, chloroform-*d*) δ 10.76 (s, 1H), 8.07 (d, *J* = 2.3 Hz, 1H), 7.91 (d, *J* = 8.0 Hz, 2H), 7.69 (dd, *J* = 8.7, 2.3 Hz, 1H), 7.27 (d, *J* = 7.9 Hz, 2H), 6.98 (d, *J* = 8.7 Hz, 1H), 6.44 (d, *J* = 3.2 Hz, 1H), 6.12 (d, *J* = 3.2 Hz, 1H), 3.99 (s, 3H), 3.40–3.33 (m, 2H), 3.14 (t, *J* = 7.5 Hz, 2H), 2.66 (t, *J* = 7.7 Hz, 2H), 1.61 (p, *J* = 7.6 Hz, 2H), 1.35 (h, *J* = 7.4 Hz, 2H), 0.93 (t, *J* = 7.3 Hz, 3H). ^13^C NMR (126 MHz, chloroform-*d*) δ 198.4, 170.6, 160.7, 154.5, 151.7, 149.1, 134.6, 131.2, 128.8, 128.3, 124.7, 123.2, 118.1, 112.5, 107.6, 104.8, 52.5, 37.1, 35.8, 33.4, 23.0, 22.5, 14.0.

##### Methyl 5-{5-[3-(4-Butylphenyl)-3-hydroxypropyl]­furan-2-yl}-2-hydroxybenzoate (**39**)

Compound **34-Bu** (1 equiv) was dissolved in THF. To the solution, palladium on activated charcoal 10% (0.05 equiv) was added. For the reaction to take place, H_2_ was bubbled in the solution for 3 h. Once completed, the reaction mixture was filtered through Celite and concentrated under reduced pressure. FCC purification: Gradient elution using petrol ether/AcOEt) (100:0 → 70:30). Clear oil (71% yield). ^1^H NMR (500 MHz, chloroform-*d*) δ 10.75 (s, 1H), 8.07 (d, *J* = 2.2 Hz, 1H), 7.70 (dd, *J* = 8.8, 2.3 Hz, 1H), 7.29 (d, *J* = 7.8 Hz, 2H), 7.18 (d, *J* = 7.8 Hz, 2H), 6.99 (d, *J* = 8.7 Hz, 1H), 6.44 (d, *J* = 3.2 Hz, 1H), 6.07 (d, *J* = 3.2 Hz, 1H), 4.74 (dd, *J* = 7.8, 5.5 Hz, 1H), 3.99 (s, 3H), 2.85–2.71 (m, 2H), 2.61 (t, *J* = 7.8 Hz, 2H), 2.25–2.07 (m, 2H), 1.63–1.56 (m, 2H), 1.36 (h, *J* = 7.4 Hz, 2H), 0.93 (t, *J* = 7.3 Hz, 3H). ^13^C NMR (126 MHz, chloroform-*d*) δ 131.1, 128.6, 125.9, 124.5, 118.0, 107.2, 104.6, 73.7, 52.4, 37.1, 35.3, 33.7, 24.7, 22.4, 14.0.

## Biological Methods

### Protein Expression and Purification

*Escherichia coli* BL21 (DE3) cells were transformed with a recombinant pET-15b expression vector encoding the human GO or LDHA or LDHB sequences (GenScript, Leiden, The Netherlands). For protein expression, 10 mL of LB medium supplemented with 0.1 mg·mL^–1^ ampicillin were inoculated with transformed cells and incubated overnight at 37 °C. A 5 mL aliquot of the overnight culture was then used to inoculate 500 mL of LB medium containing 0.1 mg·mL^–1^ ampicillin. Cultures were grown at 37 °C for 3 h until reaching an optical density (OD_600_) of approximately 0.8–1.0. The temperature was then lowered to 25 °C, and protein expression was induced by adding 0.5 mM IPTG. After 5 h of induction, cells were harvested by centrifugation and the pellets were stored at −80 °C.

Cell pellets were thawed the next day and resuspended in cold lysis buffer supplemented with PMSF, followed by sonication for cell disruption. For GO, the lysis buffer consisted of 20 mM NaH_2_PO_4_, 500 mM NaCl, 20 mM imidazole, and 50 μM FMN, pH 7.5. For LDHA/LDHB, the buffer was identical except it contained 300 mM NaCl and no FMN. Lysates were clarified by centrifugation (30,000*g*, 30 min, 4 °C), and the supernatants were loaded onto immobilized metal affinity chromatography (IMAC) columns (Cytiva, Barcelona, Spain). Columns were washed with 20 mL of lysis buffer, and proteins were eluted using the same buffer containing 500 mM imidazole.

Eluted proteins were further purified by size-exclusion chromatography. Samples were loaded onto a Superdex 200 16/60 column (GE Healthcare, Madrid, Spain) pre-equilibrated with 20 mM NaH_2_PO_4_, 500 mM NaCl, pH 7.5 for GO, or 20 mM HEPES, 200 mM NaCl, pH 7.5 for LDHA/LDHB. Fractions corresponding to the tetrameric species were pooled and concentrated. The purity of purified proteins was confirmed by SDS-PAGE.

Protein concentrations were determined spectrophotometrically using molar extinction coefficients calculated from the amino acid sequences of the human enzymes: ε_280_ = 48,000 M^–1^·cm^–1^ for GO, ε_280_ = 44,920 M^–1^·cm^–1^ for LDHA, and ε_280_ = 43,430 M^–1^·cm^–1^ for LDHB. Purified proteins were aliquoted, flash-frozen in liquid nitrogen, and stored at −80 °C.

The mean *K*_M_ values for the recombinant enzymes, based on three independent experiments, were 99 ± 33 μM for GA (*h*GO), and 311 ± 48 μM and 110 ± 33 μM for PA (*h*LDHA and *h*LDHB, respectively).

### Determination of GO Inhibition Percentage Using a Kinetic Fluorometric Assay

All assays on *h*GO were performed using substrate concentrations equivalent to those previously employed in the evaluation of compound **1**, specifically at 2-fold exceeding *K*_M_ value.^31^ The inhibitory activity of test compounds against recombinant GO was assessed using a kinetic fluorometric assay in black 96-well OptiPlates (PerkinElmer). Each plate included four replicates of: (i) 100% activity controls (containing enzyme and substrate, but not inhibitor), (ii) 0% activity controls (containing enzyme but neither substrate nor inhibitor) and (iii) each tested compound at 10 μM. The final reaction volume per well was 200 μL consisting of: 50 mM phosphate buffer (pH 7), GO (25 nM), glycolate (180 μM), Amplex Red (50 μM), Horseradish peroxidase (HRP, 2 units) and 1% DMSO.

To each well, 20 μL of either phosphate buffer pH 7 with 10% DMSO (for control wells) or a 100 μM solution of inhibitor in the same buffer was added. Then, 40 μL of GO solution (125 nM in phosphate buffer) was added, and the mixture was incubated at room temperature for 10 min. After incubation, 40 μL of an Amplex Red/HRP mixture (composed by Amplex Red 250 μM and 2 units of HRP) was added, followed by either 100 μL of MiliQ water for 0% activity control or 100 μL glycolate (solution 360 μM in water) which starts the reaction in the remaining wells. Fluorescence was immediately recorded every 60 s for the following 15 min at 27 °C using a PerkinElmer Multimode Plate Reader Enspire (λ_ex_ 560 ± 10 nm and λ_em_ 590 ± 10 nm). The initial rate of reaction was determined by calculating the slope of fluorescence increase (RFU/min) over the first 4 min. The percentage of enzyme inhibition was determined by comparing the slope for each compound to those of the 100 and 0% activity controls.

### Determination of GO Inhibition Using a Kinetic Fluorometric Assay

The same protocol described above was used to determine dose–response inhibition curves. In this format, the inhibitor was added to each well using 1:3 serial dilution prepared in 10% DMSO/phosphate buffer. A total of 10 concentrations were tested, starting from 400, 300, 200, or 100 μM depending on the compound. All other assay conditions, including reaction composition, timing, and fluorescence detection, remained unchanged.

### Determination of GO *K*_i_ Values Using a Kinetic Fluorometric Assay

Kinetic inhibition constants (*K*_i_) were determined using the same fluorometric assay format described above, with measurements performed in black 96-well OptiPlates (PerkinElmer). For each *K*_i_ determination, three replicates of four to five different inhibitor concentrations were tested in the presence of ten different glycolate concentrations. The final reaction volume per well was 200 μL and consisted of: 50 mM phosphate buffer (pH 7, 15 μL), inhibitor at the corresponding concentration in DMSO (5 μL), GO (40 μL of 125 nM solution in phosphate buffer: final concentration of 25 nM), Amplex Red/HRP mixture (40 μL containing Amplex Red 250 μM and HRP 2 units; final Amplex Red concentration: 50 μM) and glycolate (100 μL of a solution ranging from 20 mM to 1 μM; final concentration of 10 mM to 0.5 μM). In wells for *K*_M_ determination DMSO alone was used instead of inhibitor solution.

The rection was initiated by the addition of glycolate and immediately monitored by fluorescence at λ_ex_ 560 ± 10 nm and λ_em_ 590 ± 10 nm using a PerkinElmer Enspire Multimode Plate Reader. Fluorescence was recorded every 60 s over 15 min at 27 °C. The initial velocity (v_0_) was calculated from the linear portion of the fluorescence-time curve, corresponding to a 10 min interval, and expressed as the slope (RFU/min). v_0_ values were averaged across three replicates per condition.

### Determination of LDHA Inhibition Percentage Using a Kinetic Fluorometric Assay

All assays on *h*LDHA were performed using substrate concentrations equivalent to those previously employed in the evaluation of compound **1**, specifically at 3-fold exceeding *K*_M_ value.^31^

The inhibitory activity of test compounds against recombinant LDHA was assessed using a kinetic fluorometric assay in black 96-well OptiPlates (PerkinElmer). Each plate included four replicates of: (i) 100% activity controls (containing enzyme and substrate, but not inhibitor), (ii) 0% activity controls (containing enzyme but neither substrate nor inhibitor) and (iii) each tested compound at 10 μM.

The final reaction volume per well was 200 μL consisting of: 50 mM phosphate buffer (pH 7), LDHA (0.015 U/mL), pyruvate (1 mM), NADH (150 μM) and 1% DMSO. To each well, 40 μL of either phosphate buffer pH 7 with 10% DMSO (for control wells) or a 50 μM solution of the test compound (in the same buffer) was added. A fresh solution of 600 μM NADH in phosphate buffer was prepared and mixed with LDHA at 0.3 U/mL in a 5:1 ratio. Then, 60 μL of this NADH/LDHA mixture was added to each well, and background NADH degradation was monitored by measuring fluorescence every 60 s for 10 min at 27 °C using a PerkinElmer Multimode Plate Reader Enspire (λ_ex_ 340 ± 10 nm and λ_em_ 460 ± 10 nm).

Subsequently, 100 μL of either phosphate buffer (for 0% activity control) or 100 μL pyruvate (2 mM) was added to initiate the enzymatic reaction. Fluorescence was then recorded again under the same conditions to monitor the NADH consumption associated with LDHA activity. Inhibition percentages were calculated by comparing the slopes of NADH consumption between treated and control wells, after correcting for background NADH degradation.

### Determination of LDHA Inhibition Using a Kinetic Fluorometric Assay

The same protocol described above was used to determine dose–response inhibition curves. In this format, the inhibitor was added to each well using 1:3 serial dilution prepared in 10% DMSO/phosphate buffer. A total of 10 concentrations were tested, starting from 400, 300, 200, or 100 μM depending on the compound. All other assay conditions, including reaction composition, timing, and fluorescence detection, remained unchanged.

### Determination of LDHA *K*_i_ Values Using a Kinetic Fluorometric Assay

Kinetic inhibition constants (*K*_i_) were determined using the same fluorometric assay format described above, with measurements performed in black 96-well OptiPlates (PerkinElmer). For each *K*_i_ determination, three replicates of four to five different inhibitor concentrations were tested in the presence of ten different pyruvate concentrations. The final reaction volume per well was 200 μL and consisted of: 35 μL of 50 mM phosphate buffer (pH 7), 5 μL of the inhibitor at the corresponding concentration in DMSO, 60 μL of a freshly prepared NADH/LDHA mixture (500 μM NADH and 0.165 U/mL of LDHA; resulting in final concentrations of 150 μM for NADH and 0.015 U/mL for LDHA) and 100 μL of pyruvate prepared as a 3:2 serial dilution ranging from 2 mM to 52 μM; (final concentrations of 1 mM to 26 nM). In wells for *K*_M_ determination DMSO alone was used instead of inhibitor solution.

Fluorescence was registered as described above, and the initial velocity (v_0_) was calculated from the linear portion of the fluorescence-time curve, corresponding to a 10 min interval, and expressed as the slope (RFU/min). v_0_ values were averaged across three replicates per condition.

### Determination of LDHB Inhibition Using a Kinetic Fluorometric Assay

All assays on *h*LDHB were performed using substrate concentrations equivalent to those previously employed in the evaluation of compound **1** (1 mM), specifically at 9-fold exceeding *K*_M_ value.^31^

The determination of dose–response inhibition curves of test compounds against recombinant LDHB was assessed using a kinetic fluorometric assay in black 96-well OptiPlates (PerkinElmer). Each plate included four replicates of: (i) 100% activity controls (containing enzyme and substrate, but not inhibitor), (ii) 0% activity controls (containing enzyme but neither substrate nor inhibitor) and (iii) 10 different concentrations of each tested compound on 1:3 serial dilutions starting from 100 μM.

The final reaction volume per well was 200 μL consisting of: 50 mM phosphate buffer (pH 7), LDHB (0.015 U/mL), pyruvate (1 mM), NADH (150 μM) and 1% DMSO. To each well, 40 μL of either phosphate buffer pH 7 with 10% DMSO (for control wells) or a 50 μM solution of the test compound (in the same buffer) was added. A fresh solution of 600 μM NADH in phosphate buffer was prepared and mixed with LDHB at 0.3 U/mL in a 5:1 ratio. Then, 60 μL of this NADH/LDHB mixture was added to each well, and background NADH degradation was monitored by measuring fluorescence every 60 s for 10 min at 27 °C using a PerkinElmer Multimode Plate Reader Enspire (λ_ex_ 340 ± 10 nm and λ_em_ 460 ± 10 nm).

Subsequently, 100 μL of either phosphate buffer (for 0% activity control) or 100 μL pyruvate (2 mM) was added to initiate the enzymatic reaction. Fluorescence was then recorded again under the same conditions to monitor the NADH consumption associated with LDHA activity. Inhibition percentages for each concentration were calculated by comparing the slopes of NADH consumption between treated and control wells, after correcting for background NADH degradation.

### Determination of LDHB *K*_i_ Values Using a Kinetic Fluorometric Assay

Kinetic inhibition constants (*K*_i_) were determined using the same fluorometric assay format described above, with measurements performed in black 96-well OptiPlates (PerkinElmer). For each *K*_i_ determination, three replicates of four to five different inhibitor concentrations were tested in the presence of ten different pyruvate concentrations. The final reaction volume per well was 200 μL and consisted of: 35 μL of 50 mM phosphate buffer (pH 7), 5 μL of the inhibitor at the corresponding concentration in DMSO, 60 μL of a freshly prepared NADH/LDHB mixture (500 μM NADH and 0.165 U/mL of LDHB; resulting in final concentrations of 150 μM for NADH and 0.015 U/mL for LDHB) and 100 μL of pyruvate prepared as a 3:2 serial dilution ranging from 2 mM to 52 μM; (final concentrations of 1 mM to 26 nM). In wells for *K*_M_ determination DMSO alone was used instead of inhibitor solution.

Fluorescence was registered as described above, and the initial velocity (v_0_) was calculated from the linear portion of the fluorescence-time curve, corresponding to a 10 min interval, and expressed as the slope (RFU/min). v_0_ values were averaged across three replicates per condition.

### Animal Studies

All animal procedures were approved by the Institutional Animal Care & Use Committees of Louisiana State University Health Sciences Center-Shreveport (P-21–043, and P-24–025). All studies were performed in accordance with the institutional guidelines. Mice were randomly allocated to treatment groups followed by confirmation of equal body weights before treatment. *Agxt* ^–/–^ mice on the C57BL/6J background were generated using CRISPR/Cas9, with guide-RNA targeting exon 1 of Agxt.^8^ *Agxt* ^–/–^ mice were housed under controlled temperature (22 ± 2 °C) and humidity conditions (40–60%) on a 12-h light–dark cycle and fed ad libitum with a standard chow diet (LabDiet, 5053, 13% of calories from fat). Twelve-week-old male *Agxt* ^–/–^ were orally gavaged with either an aqueous solution of 0.6% Methyl cellulose (Sigma-Aldrich) + 0.5% Tween 80 (Sigma-Aldrich) or **2** at a 20 mg/kg/day dissolved in the same formulation. Urine was collected starting 2 days prior to dosing to set a baseline and throughout the treatment regimen. After 10 days, the mice were sacrificed, blood was collected, livers and kidneys were fixed in 4% formalin.

### Hepatocyte Isolation

Primary hepatocytes were isolated from 8–10-week-old male *Agxt* ^–/–^ mice. Following euthanasia, the left lateral lobe of the liver was carefully excised and rinsed with phosphate-buffered saline (PBS). Hepatocyte isolation was performed using the gentleMACS Perfuser system and associated kits from Miltenyi Biotec, following the described protocol.^63^ Briefly, the gentleMACS Perfuser was assembled according to the manufacturer’s instructions, consisting of a lid, clamp, grid, and base. The liver lobe was secured onto the grid, and the lid was attached to the base to form a sealed chamber. A prewarmed predigestion buffer was added to initiate the automated perfusion program. This program includes multiple steps: priming, initial perfusion, washing, equilibration, and enzymatic perfusion. An enzyme digestion solution containing collagenase and other matrix-degrading enzymes was perfused to facilitate hepatocyte dissociation. Upon completion of enzymatic digestion, the digested liver lobe was transferred into a gentleMACS C Tube for further mechanical dissociation via gentle stirring. The resulting cell suspension was filtered through a MACS SmartStrainer (Miltenyi Biotec) to remove tissue debris and nonparenchymal cells. The filtrate containing hepatocytes was collected and subjected to low-speed centrifugation (50*g* for 3 min) to pellet the cells. Isolated primary hepatocytes were then resuspended in warm thawing and plating medium and plated at approximately 80% confluence into collagen-coated plates (0.01% *w*/*v*, Advanced Biomatrix NC0476635). Cells were allowed to adhere for approximately 3–4 h and nonadhered cells and debris were rinsed with warm PBS. Cells were maintained in hepatocyte maintenance medium (William’s E Medium (Gibco, A1217601) with maintenance supplements (Gibco, CM4000) at 37 °C in a humidified atmosphere containing 5% CO_2_.

### Cell Culture Treatments and Measurements

Mouse primary hepatocytes were maintained in maintenance medium and utilized for experiments no longer than 48 h postisolation. Hepatocytes were plated at approximately 80% confluence and allowed to adhere overnight. Next day, cells were challenged with glycolic acid at a concentration of 5 mM and treated with either vehicle (0.1% DMSO) or the desired compound at 2,10, 20, or 50 μM for additional 24 h. Cell viability was determined using CellTiter-Blue assay (Promega, G8080) according to manufacturer’s protocol, at the highest tested dose for each compound. For proteasomal inhibition studies, overnight adhered primary hepatocytes were treated with 10 μM MG-132 (Sigma-Aldrich, 474790) for 4 h, followed by treatment with either compound **2** or DMSO for 24 h. For silencing studies, primary hepatocytes were treated during seeding with either siAtg5 (Mouse Atg5 ON-TARGETplus siRNA, Dharmacon, CO, L-064838–00–0005) or scRNA (ON-TARGETplus nontargeting pool, Dharmacon, CO, D-001810–10–05) overnight using Lipofectamine RNAiMAX (Invitrogen, 13778150) transfection reagent following the manufacturer’s protocol. At the next day, the hepatocytes were treated with either compound **2** or DMSO for 24 h. For oxalate quantification following treatment, cells were washed twice with PBS, gently scraped into PBS, and collected by centrifugation. Cell pellets were stored at −80 °C until further processing. Frozen cell pellets were thawed and lysed *via* sonication using a probe sonicator (Sonics Vibra-Cell, VCX130–50W). Oxalate levels in cell lysates were measured using the Oxalate Assay Kit (Abcam, ab196990) according to the manufacturer’s instructions. Final oxalate concentrations were normalized to total protein content determined using a standard BCA protein assay (Thermo Fisher Scientific or equivalent).^9^

### RNA Isolation and qRT–PCR

RNA isolation from primary hepatocytes was carried out using RNeasy Mini Kit (QIAGEN, 74106) following the manufacturer’s protocol. Complementary DNA (cDNA) was synthesized using the SuperScript III First-Strand Synthesis System (Invitrogen, 18080–051) in accordance with the manufacturer’s instructions, utilizing a Mastercycler nexus gradient thermocycler (Eppendorf). Primers were obtained from Integrated DNA Technologies (*Hao1*: Forward 5′-TTGGGCTACCTCCTCAATAGAA-3′; Reverse 5′-TCTGTCTGCTGATCTCACGGT-3′; *Ldha*: Forward 5′- CAAAGACTACTGTGTAACTGCGA-3′; Reverse 5′-TGGACTGTACTTGACAATGTTGG-3′; *Gapdh*: Forward 5′-CTGCGACTTCAACAGCAACT-3′; Reverse 5′- GAGTTGGGATAGGGCCTCTC-3′) and qRT–PCR was conducted with the SSoAdvanced Universal SYBR Green Supermix (Bio-Rad, 175271) on a CFX96 Touch Real-Time PCR Detection System (Bio-Rad). Gene expression data were normalized to the *Gapdh* housekeeping gene and analyzed using the ^ΔΔ^Ct method to determine fold changes relative to control groups.

### Protein Isolation and Western Blot

For protein extraction from liver tissue, approximately 50 mg of sample was used. The tissue was homogenized in RIPA lysis and extraction buffer (G Biosciences, 786–489) containing 1% Halt protease inhibitor cocktail (Thermo Scientific, 78429), 1% phosphatase inhibitor cocktail A (Alfa Aesar, J65354.LQ), and ceramic homogenization beads (Cayman Chemical Company, 10011152), using the Precellys Evolution homogenizer (Bertin Technologies). For cell lysates, RIPA buffer with the same inhibitor cocktails (Bio-Rad, 1610737) was used. Protein concentration was determined using the Quick Start Bradford Dye Reagent (Bio-Rad, 5000205). Western blotting was performed using the following primary antibodies: rabbit anti-AGXT (Sigma, HPA035370, 1:1000), rabbit anti-LDHA (Cell Signaling Technology, 2012, 1:1000), rabbit anti-HAO1 (Abcam, 194790, 1:1000), mouse anti-β-actin (Cell Signaling Technology, 3700S, 1:1000), mouse anti-GAPDH (Santa Cruz Biotechnology, sc365062, 1:5000) and rabbit anti-ATG5 (Cell Signaling Technology, 12994S, 1:1000). Fluorescent secondary antibodies—donkey antirabbit (Li-Cor, 926–68073, 1:20,000) and donkey antimouse (Li-Cor, 926–32212, 1:20,000)—were used for detection with a Li-Cor Odyssey XF Imager. Band intensities were quantified using Image Studio Lite v.5.2 and normalized to GAPDH or β-actin levels.

### Biochemical Analyses of Plasma and Livers

Immediately after euthanizing the mice, plasma was obtained by centrifuging whole blood collected in lithium heparin-coated PST tubes (BD Microtainer, 365985). Activity of aspartate transaminase (AST) and alanine transaminase (ALT) in plasma were determined using EnzyChrom assay kits specific for each enzyme (BioAssay Systems, EASTR-100 for AST and EALT-100 for ALT), following the protocols provided by the manufacturer. Lactate dehydrogenase (LDH) activity in liver samples was assessed using the LDH Assay Kit (Abcam, ab102526) in accordance with the manufacturer’s guidelines.

### Liver Glycolate Oxidase Activity

Approximately 30 mg of liver tissue were homogenized in phosphate buffer pH 7 with soft tissue ceramic beads in a Precellys Evolution homogenizer as described above. Glycolate Oxidase activity was assessed using a kinetic fluorometric assay in black 96-well OptiPlates (PerkinElmer). Each plate included three replicates of: (i) 1:3 serial dilutions of an H_2_O_2_ standard curve ranging from 20 μM to 250 nM; (ii) basal production of H_2_O_2_ (liver homogenate without substrate) and (iii) H_2_O_2_ production linked to GO activity (liver homogenate with glycolate substrate). The final reaction volume per well was 200 μL consisting of: 50 mM phosphate buffer (pH 7), tissue homogenate (∼0.6 mg/mL), glycolate (5 mM), Amplex Red (50 μM), and Horseradish peroxidase (HRP, 2 units).

For the H_2_O_2_ standard wells, 100 μL of freshly prepared H_2_O_2_ solution were mixed with 60 μL phosphate buffer and 40 μL of an Amplex Red/HRP mixture (Amplex Red 250 μM and HRP 2 units). For experimental wells, 50 μL phosphate buffer (pH 7.0) and 10 μL liver homogenate were added, followed by incubation at room temperature for 10 min. After incubation, 40 μL of the Amplex Red/HRP mixture were added, followed by either 100 μL Milli-Q water (for basal H_2_O_2_ production) or 100 μL glycolate solution (10 mM in water) to assess GO-dependent H_2_O_2_ generation. Fluorescence was immediately recorded every 60 s for the following 10 min at 27 °C using a PerkinElmer Multimode Plate Reader Enspire (λ_ex_ 535 ± 10 nm and λ_em_ 590 ± 10 nm). The initial reaction rate was determined by calculating the slope of fluorescence increase (RFU/min) over the first 4 min. GO enzymatic activity for each sample was calculated by subtracting basal H_2_O_2_ production from total H_2_O_2_ production in the presence of glycolate.

### Histology

Formalin-fixed tissues were sectioned on a M355S rotary microtome (Thermo Fisher Scientific) at 4-μm thickness and mounted on glass slides. Slides were stained for H&E (Thermo Fisher Scientific). Calcium-oxalate crystals were visualized using a Nikon Eclipse TS2R-FL microscope equipped with a polarized light filter. Image analysis was performed with *ImageJ* software. Histopathological evaluation was carried out by two blinded and one nonblinded pathologist, and the results were averaged to obtain the overall mean. All analyses were conducted using three fields of view per kidney.

### Oxalic and Glycolic Acid Analysis in Urine Using Enzymatic Assays

A volume of 50 μL of urine collected from mice before and during treatment, was diluted with 150 μL of ultrapure water and purified using Trinity Biotech Sample Purifier Tubes (591–100). Purified samples were then used to determine oxalate and glycolate concentrations. Oxalate levels were quantified using the Trinity Biotech Oxalate Assay Kit (591-D) and the glycolate levels were measured using the fluorometric assay from Abcam (ab282915) measure following the protocols provided by the manufacturer.

### Targeted Analysis of Compound **2** Distribution

Approximately 25 mg of liver, kidney gastrocnemius and heart tissue were homogenized in 500 μL methanol with soft tissue ceramic beads in a Precellys Evolution homogenizer as described above. After 1:1000 dilution detection of compound **2** was conducted using a Thermo Fisher Vanquish Horizon UHPLC system coupled to an Orbitrap Exploris 120 mass spectrometer. Chromatographic separation was achieved on a Thermo Accucore aQ column (2.6 μm; 2.1 × 100 mm^2^) maintained at 30 °C, with a flow rate of 0.3 μL/min. Mobile phase A consisted of LC-MS grade water (Thermo) with 0.1% formic acid. Mobile phase B consisted of LC-MS grade acetonitrile (Supelco) with 0.1% formic acid. The gradient began with 100% A for 1 min, followed by a linear increase to 98% B over 13 min, a hold at 98% B for 3 min, a rapid return to 100% A over 0.1 min, and a final hold at 100% A for 2.9 min. Targeted selective ion monitoring mode was utilized to detect compound **2** (578.2901 *m*/*z*) in positive ion mode. A calibration curve was made for each compound for quantitative analysis of samples.

## Computational Methods

### Docking Protocol

Docking studies were carried out with Autodock 4.2.6 (AD4)^64^ on the crystal structures of human lactate dehydrogenase M isozyme form (*h*LDHA, PDB ID: 1I10)^39^ and human glycolate oxidase (*h*GO, PDB ID: 2RDT).^33^ Ligand PDB structure was built and minimized on MOE.^65^ Once optimized, ligand PDB was prepared for docking using the prepare_ligand4.py script included MGLTools 1.5.4.^66^ Protein structures, on the other hand, were prepared for docking using MOE’s structure preparation module.^65^ Water and ligand molecules were removed and charges and nonpolar hydrogen atoms were added at pH 7.0. The produced structures were saved as a pdb files and prepared for docking using the prepare_receptor4.py script from MGLTools. AD4 was used to automatically dock the ligands into the *h*LDHA and *h*GO. For *h*LDHA (PDB ID 1I10) the docking grid was centered on the pyruvate-NADH binding site and set with the following grid parameters: 85 Å × 90 Å × 80 Å with 0.375 Å spacing. For *h*GO (PDB ID: 2RDT) the docking grid was centered on the FMN-CDST binding site and set with the following grid parameters: 80 Å × 80 Å × 80 Å with 0.375 Å spacing. In all calculations, AD4 parameter file was set to 100 GA runs, 2.500.000 energy evaluations and a population size of 150. The Lamarckian genetic algorithm local search (GALS) method was used for the docking calculations. All dockings were performed with a population size of 250 and a Solis and Wets local search of 300 rounds was applied with a probability of 0.06. A mutation rate of 0.02 and a crossover rate of 0.8 were used. The docking results from each of the 100 calculations were clustered based on root-mean square deviation (RMSD) (solutions differing by less than 2.0 Å, except where stated) between the Cartesian coordinates of the atoms and were ranked on the basis of free energy of binding. UCSF Chimera 1.15 ^67^ was used for molecules visualization and figures generation.^67^

### Homology Modeling and Molecular Dynamic Protocol

A set of homology models of *h*GO were constructed using the **2**-*h*GO (PDB ID 2RDT) docking pose obtained previously as a template. For this task, MOE Homology Model module was used with the default parameters. Ten models were created and classified according to their GB/VI energies, and the best performing model was selected. This model was next prepared using MOE QuickPrep module to correct the structure and to prepare the macromolecular complex for further MD analysis. For *h*LDHA, the obtained docking pose of ligand **2** on *h*LDHA (PDB ID 1I10, subunit B) was used on MOE to build a dimeric complex with subunit A of *h*LDHA (PDB ID 1I10), the latter containing cofactor 1,4-dihydronicotinamide adenine dinucleotide (PDB ID: NAI) and oxamic acid (PDB ID: OXM) inhibitor. The spatial conformation of the dimeric complex was based on the published crystal structure (1I10), saved as a pdb file and prepared for further MD analysis using MOE QuickPrep module.^68^ Both **2**-*h*GO and **2**-*h*LDHA pdb files were next used for atomistic molecular dynamic (MD) simulation on NAMD 2.14. **2** ligand, FMN, NAI, and OXM were parametrized using LigParGen server^69–71^ and the MD simulations ran using OPLS-AA/M force field.^72^ The initial ensembles were first submitted to a 30 ps (ps) energy minimization at 300 K on periodic boundary conditions (grid size: 84 × 73 × 42) containing the biomolecular complexes in a water box (0.15 M NaCl concentration) to remove high-energy contacts, followed by successive 300 ps NVT and a 1 ns (ns) NPT equilibration before the productive 100 ns MD simulation. During the MD simulation, water bonds were constrained using the SHAKE algorithm;^73^ for every 2 fs, neighbors were searched in grid cells with 1 nm as the cutoff value for short-range neighbor list, electrostatic, and van der Waals; long-range electrostatics were treated with the particle mesh Ewald method^74^ with a grid spacing of 1; constant temperature and pressure were maintained by coupling the system to an external bath at 300 K and 1 bar, using velocity rescaling^75^ and Parrinello–Rahman,^76^ respectively. A RESPA propagator with the integration time step of 1 fs was used.^77^ TIP3P model was applied for water,^78^ and UCSF Chimera 1.15 was used to perform the trajectory analyses and generate the final images. The trajectories data were subjected to an RMSD cluster analysis using the MD movie Tool in Chimera setting frame 0 as the starting one, and the default step size parameter of 34. The obtained most populated cluster-representative structures were saved as a pdb files and analyzed. Solvent accessible surface area (SASA), H-bonds and root-mean-square deviation (RMSD) calculations of the simulations were done using VMD^79^ with in-house TLC scripts.

#### Chameleonicity Evaluation

Conformational ensembles were generated using the LowModeMD search engine implemented in MOE 2024 (6, Montreal, Canada)^65^ with the MMFF94x force field and GB/VI implicit solvent model. Separate searches were performed in implicit water (ε = 80) and implicit chloroform (ε = 4.8). All conformers were energy-minimized to a root-mean-square (RMS) gradient ≤  0.005 kcal mol^–1^ Å^–1^.

Implicit Water (ε = 80): Search method: LowModeMD (default); Rejection limit: 100; Iteration limit: 10,000; Energy window: 7 kcal mol^–1^; RMSD limit: 0.25 Å; Conformation limit: 10,000. The search produced 526 unique conformers. The 200 lowest-energy conformers (Δ*E* ≤  10 kcal mol^–1^ from the global minimum) were retained for further analysis.

Implicit Chloroform (ε = 4.8): Search method: LowModeMD; Rejection limit: 500; Iteration limit: 10,000; Energy window: 12 kcal mol^–1^; RMSD limit: 0.35 Å; Conformation limit: 10,000. The search produced 294 unique conformers. The 200 lowest-energy conformers (Δ*E* ≤  10 kcal mol^–1^ from the global minimum) were retained for further analysis.

3D polar surface area (3D-PSA) was calculated with VEGA ZZ using the SRFCALC DOTS PSA 0.0 2 0 command. Intramolecular hydrogen bonds (IMHB) and radius of gyration (*R*_gyr_) were calculated using custom Python scripts built on the RDKit cheminformatics library.^80^ The resulting data from conformer ensembles were analyzed to compute environment-dependent distributions of 3D-PSA, *R*_gyr_, and IMHB counts. Density plots and 2D/3D scatter plots were constructed to compare conformational profiles across solvents. The data interpretation followed the established protocols from Rossi Sebastiano^43^ and Garcia Jimenez.^42^

### Statistical Analyses

All statistical analyses were performed using GraphPad Prism v.10 software and Microsoft Excel 365. All data were expressed either as mean ± SD or mean ± SEM and repeated with at least three independent experiments. Biological replications were performed as indicated and averaged for each individual experiment. Each data point presented represents an independent experiment or an individual subject. Before statistical comparisons, data were tested for equal variance and normality using Shapiro–Wilk and Kolmogorov–Smirnov tests. If data passed, an unpaired *t*-test was used to compare two groups and a one-way ANOVA followed by Tukey’s post hoc test for comparisons among more than two groups. Otherwise, nonparametric tests (Mann–Whitney *U*-test or Kruskal–Wallis test followed by Dunn’s post hoc test) were used. *P* < 0.05 was considered statistically significant.

## Supplementary Material









## Notes

Additional experimental details, including materials and methods, HPLC traces of compounds evaluated *in vitro* or *in vivo*, ^1^H and ^13^C NMR spectra for all compounds, IC_50_ curves, nonlinear and linear plots for *K*_i_ determination, supplementary docking and molecular dynamics figures (PDF)
2RDT homology (PDB)
1I10 (PDB)
Molecular formula strings for final compounds (CSV)

## ∇

Structural Biology Laboratory, Elettra-Sincrotrone Trieste, 34149 Basovizza, Trieste, Italy

## ○

Department of Molecular Sciences and Nanosystems, Ca’ Foscari University of Venice, 30172 Mestre-Venice, Italy

## Abbreviations Used

**3D-PSA**: tridimensional polar surface area
**AcOEt**: ethyl acetate
**AD4**: Autodock 4.2.6
**AGXT**: alanine-glyoxylate aminotransferase
**ALT**: alanine aminotransferase
**AMFSA**: aminomethylfuryl salicylic acid
**AST**: aspartate aminotransferase
***Atg5***: autophagy related gene 5
**bRo5**: beyond Rule of 5
**Ca-Ox**: calcium-oxalate
**EtOH**: ethanol
**FCC**: flash column chromatography
**FSA**: furylsalicylic acid
**GA**: glycolic acid
**GO**: glycolate oxidase
**H&E**: hematoxylin and eosin
***h*** **GO**: human isoform of glycolate oxidase
***h*** **LDHA**: human isoform of lactate dehydrogenase A
**HyT**: hydrophobic-tag
**HyT-PD**: hydrophobic-tag protein degrader
**IMHB**: intramolecular hydrogen bond
**LDHA**: lactate dehydrogenase isozyme A
**MASH**: metabolic dysfunction-associated steatohepatitis
**MASLD**: metabolic dysfunction-associated steatotic liver disease
**MeOH**: methanol
**MOE**: molecular operative environment
**NAI**: 1,4-dihydronicotinamide adenine dinucleotide
**OPPFSA**: oxophenylpropenylfuryl salicylic acid
**OXM**: oxamic acid
**PA**: pyruvic acid
**PH1**: primary hyperoxaluria type 1
**PLC**: preparative layer chromatography
**PoI**: protein of interest
**qRT–PCR**: quantitative real-time polymerase chain reaction
***R*_gyr_**: radius of gyration
**RMSD**: root-mean square deviation
**SA**: salicylic acid
**siRNA**: small interfering RNA
**siScr**: control scrambled siRNA
**SL**: selectivity ligand
