# Supplementary material for: Targeting Oxalate Production by Combining Enzyme Inhibition and Proteolysis Activation: A Novel Therapeutic Approach for Primary Hyperoxaluria Type 1
Source: J Med Chem. 2026 Jan 2;69(3):2258–86. doi: 10.1021/acs.jmedchem.5c02055 (PMC12795515; doi:10.1021/acs.jmedchem.5c02055)

SUPPORTING INFORMATION

Targeting oxalate production by combining enzyme inhibition and proteolysis activation: A novel therapeutic approach for primary hyperoxaluria type 1

Fabio Arias[a,b,c,‡] , Sumati Rohilla[c,‡], Yudibeth Sixto-López[a,b], Koral S.E. Richard[c], Sandeep Das[c], Sumit K. Anand[c], Pilar Maria Luque-Navarro[a,b], Guillermo Bañuelos-Sanchez[a], Juan Luis Pacheco-García[d,†], Reethika Gade[c], M. Peyton McKinney[c], Dhananjay Kumar[e], Jemiah Maxie[c], W. Rylan Corr[c], Nilesh Pandey[c], Harpreet Kaur[c], Jibin Ding[f], Lin Tan[f], Elisha Scott[c], Hyung Nam[c], Eyal Gottlieb[f], A. Wayne Orr[c,e], Nirav Dhanesha[c], Arif Yurdagul Jr [c,e], Angel L. Pey[b,d], Francisco Franco-Montalbán[a,b], José A. Gómez Vidal[a,b], Oren Rom[c,e,*], Mónica Díaz-Gavilán[a,b,*].

[a]Departamento de Química Farmacéutica y Orgánica, Facultad de Farmacia, Universidad de Granada. Campus Cartuja s/n, 18071, Granada, Spain.

[b]Unidad de Excelencia en Química Aplicada a Biomedicina y Medioambiente, Universidad de Granada, Av. Fuentenueva s/n, 18071, Granada, Spain.

[c]Department of Pathology and Translational Pathobiology, Louisiana State University Health Sciences Center-Shreveport, 71103, Shreveport, LA, USA.

[d]Departamento de Química Física e Instituto de Biotecnología, Universidad de Granada, Av. Fuentenueva s/n, 18071, Granada, Spain.

[e]Department of Molecular and Cellular Physiology, Louisiana State University Health Sciences Center-Shreveport, 71103, Shreveport, LA, USA.

[f]Metabolomics Core Facility, Department of Bioinformatics and Computational Biology, The University of Texas MD Anderson Cancer Center, 77030, Houston, TX, USA.

KEYWORDS: Primary Hyperoxaluria, Dual Inhibitors, Glycolate Oxidase, Lactate Dehydrogenase, Hydrophobic Tag, Protein Degradation, Oxalate Reduction.

[‡] These authors contributed equally to this work.

*Corresponding authors: Dr. M. Díaz-Gavilán, monicadg@ugr.es. Dr. O. Rom, oren.rom@lsuhs.edu.

TABLE OF CONTENTS

S1. Chemistry 3

S1. 1. “Other structures” 3

S1. 2. HPLC methods for purity determination 3

S1.3. Synthetic procedures 4

S2. Screening on recombinant enzymes 12

S3. Determination of IC_{50}s on recombinant *h*GO using a kinetic fluorometric protocol with Amplex® Red 13

S4. Determination of IC_{50}s on recombinant *h*LDHA using a kinetic fluorometric protocol based on NADH 26

S5. Evaluation of dual inhibitors on hepatocytes of PH1 mice (*in vitro*) 40

S6. Inhibition kinetics on *h*GO 42

S7. Inhibition kinetics on *h*LDHA 52

S8. Evaluation of compound 2 on enzyme *h*LDHB 63

S9. Assessment of GO and LDHA degradation by compound 26 67

S10. Determination of the mechanism for the compound 2-mediated degradation of *h*LDHA 68

S11. Docking of compound 2 68

S.11.1. Docking of compound 2 on *h*GO (PDB 2RDT) 68

S11.2. Docking of compound 2 on *h*LDHA (PDB 1I10) 70

S12. Homology modelling and molecular dynamic protocol 70

S12.1. MD simulation on *h*GO 70

S12.2. MD simulation on *h*LDHA 73

S13. Comparative binding-mode analysis of compounds 2 and 26. 76

S14. Characterization of chameleonicity of compound 2. 78

S15. Evaluation of compound 2 in *Agxt*$^{-/-}$ mice *in vivo* 79

S.16. References 82

S.17. HPLC TRACES 83

S.18. NMR SPECTRA 95

S1. Chemistry

S1. 1. "Other structures"

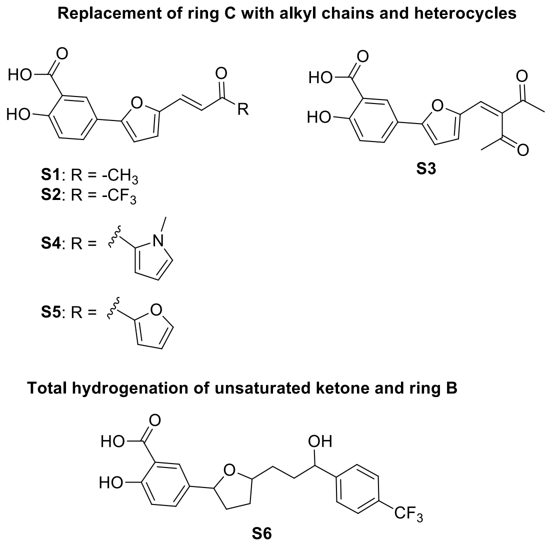

Figure S1. Structural analogues of OPPFSAs ("*other structures*").

S1. 2. HPLC methods for purity determination

General HPLC setup: Detection λ = 214 and 254 nm; flow rate 0.8 mL/min; solvent A [water (0.1% HCOOH) / acetonitrile (0.1% HCOOH)]; solvent B [acetonitrile (0.1% HCOOH) 100%]. Injection volume 10 µL.

Column 1: Zorbax Eclipse XDB-C18 5µM, 4.6 × 150 mm.

Column 2: Waters XBridge Column-C18 2.5 µm, 4.6 x 75 mm.

Column 3: Waters XBridge Column-C8 2.5 µm, 4.6 x 75 mm.

HPLC method B: Solvent A (90/10). Isocratic A 2 min +gradient A→ B 8 min +isocratic B 1 min.

HPLC method C: Solvent A (70/30). Isocratic A 2 min +gradient A→ B 8 min +isocratic B 1 min.

HPLC method D: Solvent A (60/40). Isocratic A 2 min +gradient A→ B 8 min +isocratic B 1 min.

HPLC method E: Solvent A (50/50). Isocratic A 2 min +gradient A→ B 8 min +isocratic B 1 min.

S1.3. Synthetic procedures

2-Hydroxy-5-[5-(propargylaminomethyl)furan-2-yl]benzoic acid (1)

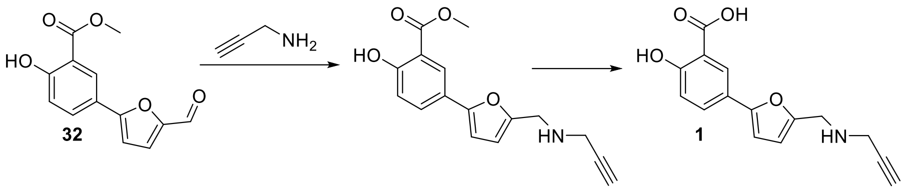

A solution of compound **32** (150 mg, 0.54 mmol, 1 equiv) was prepared under argon, in a mixture of DCM:MeOH (4:1) (5 mL) in a round bottom flask containing activated molecular sieves (3 Å). The flask was wrapped in aluminium foil before propargylamine (41 µL, 0.64 mmol, 1.2 equiv) was added at rt. The formation of the imine, in the absence of light, was followed by TLC during 3 h. After this time, the reaction was cooled to 0 ºC (ice bath) and $NaBH_3CN$ (84 mg, 1.33 mmol, 2.5 equiv) was added at this temperature. The ice bath was then removed, and the reaction was let to stir for 3 h more. The reaction was then quenched by addition of distilled water (5 mL). Next, the organic solvents were evaporated under reduced pressure and the aqueous phase was extracted with AcOEt (2 x 25 mL). The organic phases were combined, dried on MgSO4, filtered, and evaporated under reduced pressure. The residue was purified by FCC with hexanes/AcOEt mixtures (9:1 →3:1). Yield: 66% (100 mg).

The purified intermediate ester (140 mg, 0.49 mmol, 1 equiv) was dissolved in MeOH (10 mL) in a round bottom flask. Next, an aqueous solution of NaOH (5 M) was added dropwise and the mixture was stirred and heated to 50 ºC during 3 h. After disappearance of the starting material (TLC) the reaction was stopped by addition of HCl (1N). The mixture was evaporated under vacuum and purified by FCC through a small pad of silica using AcOEt and AcOEt:MeOH mixtures (up to 1:1). Yield: 96%. NMR spectra agreed with the previously reported.[1] **HPLC** (method B) (λ = 254 nm), 100%; (λ = 214 nm), 96.8%; ^{t}R = 7.99 min (column 1).

(*E*)-2-Hydroxy-5-[5-(3-oxobut-1-en-1-yl)furan-2-yl]benzoic acid (S1).

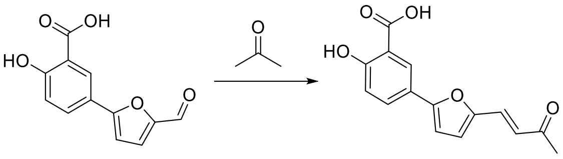

3 **S1**

In a round bottom flask equipped with a stirring bar, 50 mg of **3** (0.215 mmol) were dissolved in acetone (13.62 mmol)/H2O 1/1 (2 mL) and the mixture was cooled to 0 °C. Next, 100 µL of NaOH 1N were added dropwise and the reaction was stirred for 10 h at rt. Then, the reaction was quenched with HCl 1 N until pH 1 and concentrated in rotavapor. The crude product was purified by FCC with DCM/MeOH (20:1 → 15:1). Yellow solid, 51% yield (30 mg, 0.110 mmol). mp > 300 ºC. ^{1}**H NMR** (400 MHz, chloroform-*d*) δ 8.17 (d, *J* = 2.3 Hz, 1H), 7.95 (dd, *J* = 8.7, 2.4 Hz, 1H), 7.43 (d, *J* = 16.0 Hz, 1H), 7.10 – 7.01 (m, 3H), 6.58 (d, *J* = 16.0 Hz, 1H), 2.30 (s, 3H). ^{13}**C NMR** (101 MHz, DMSO-*d*6) δ 197.2 (CO), 171.2 (CO), 161.3 (C), 154.9 (C), 149.6 (C), 131.2 (CH), 129.2 (CH), 125.7 (CH), 123.2 (CH), 120.7 (C), 119.1 (CH), 118.0 (CH), 114.2 (C), 107.8 (CH), 27.4 (CH3). **HRMS** (TOF ES^{-}): *m/z* calcd for $C_{15}H_{11}O_5$ (M–H)$^{-}$ 271.0606, found 271.0615. **HPLC** (method B) (λ = 254 nm), 100%; (λ = 214 nm), 96.4%; ^{t}R =9.94 min (column 2).

(*E*)-2-Hydroxy-5-[5-(4,4,4-trifluoro-3-oxobut-1-enyl)furan-2-yl]benzoic acid (S2).

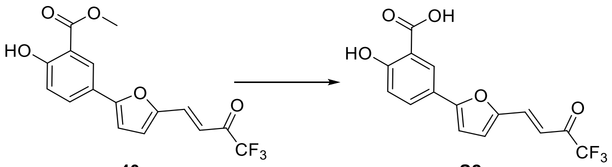

40 **S2**

Compound **40** (62 mg, 0.18 mmol, 1 equiv) was dissolved in pyridine (5 mL) and heated up to reflux (130 ºC) during 15 h. After this time, pyridine was removed using a high vacuum rotavapor. The residue was dissolved in AcOEt and the remaining trace of pyridine was extracted with aqueous HCl 1N. The organic layer was washed with brine, dried over $MgSO_4$, filtered and concentrated under reduced pressure. FCC purification: Gradient elution using petrol ether:AcOEt (9:1 →1:1) and DCM:MeOH (8:2). All mobile phases were acidified with 1% AcOH. Compound S2 was obtained as a mixture of two atropoisomers with *trans* configuration in 1:0.7 proportion (M = major; m = minor). ^{1}**H NMR** (400 MHz, methanol-d_4) δ 8.32 (d, *J* = 2.4 Hz, $1H_m$), 8.21 (d, *J* = 2.3 Hz, $1H_M$),

7.94 (dd, *J* = 8.7, 2.3 Hz, $1H_m$), 7.84 (dd, *J* = 8.7, 2.3 Hz, $1H_M$), 7.78 (d, *J* = 15.0 Hz, $1H_m$), 7.20 (d, *J* = 3.7 Hz, $1H_m$), 7.04 (d, *J* = 8.8 Hz, $1H_m$), 7.00 (d, *J* = 8.7 Hz, $1H_M$), 6.96 (d, J = 3.7 Hz, $1H_m$), 6.93 (d, *J* = 15.5 Hz, $1H_m$), 6.85 (d, *J* = 15.8 Hz, $1H_M$), 6.71 (d, *J* = 3.5 Hz, $1H_M$), 6.55 (d, *J* = 3.4 Hz, $1H_M$), 6.03 (d, *J* = 15.8 Hz, $1H_M$). **^{13}C NMR** (101 MHz, methanol-*d4*) *δ* 180.7, 180.3, 173.3, 173.1, 164.1, 162.9, 159.6, 155.0, 151.8, 151.3, 135.6, 132.9, 132.1, 128.1, 126.8, 125.8, 125.6, 124.9, 123.3, 123.0, 121.9, 120.6, 119.5, 119.2, 118.9, 116.6, 114.9, 114.5, 114.3, 112.9, 109.4, 107.1. **HRMS** (TOF ES$^-$): *m/z* calcd for $C_{15}H_8O_5F_3$ $(M–H)^-$ 325.0324, found 325.0324 (deviation 0.0 ppm). **HPLC** (method E) (λ = 254 nm), 97.8%; (λ = 214 nm), 95.1%; ^{t}R =7.07 min (column 2).

5-[5-(2-Acetyl-3-oxobut-1-enyl)furan-2-yl]-2-hydroxybenzoic acid (S3).

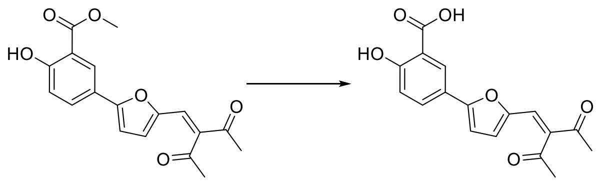

41 **S3**

Compound **41** (54 mg, 0.16 mmol, 1 equiv) was dissolved in pyridine (5 mL) and heated up to reflux (130 ºC) during 6 h. After this time, pyridine was removed using a high vacuum rotavapor. The residue was dissolved in AcOEt and the remaining trace of pyridine was extracted with aqueous HCl 1N. The organic layer was washed with brine, dried over $MgSO_4$, filtered and concentrated under reduced pressure. FCC purification: Gradient elution using petrol ether:AcOEt (9:1) → AcOEt:MeOH (9:1). All mobile phases were acidified with 1% AcOH. Yield 70%. **^{1}H NMR** (500 MHz, methanol-*d4*) *δ* 8.24 (d, *J* = 2.4 Hz, 1H), 7.78 (dd, *J* = 8.7, 2.3 Hz, 1H), 7.39 (s, 1H), 7.03 (d, *J* = 3.7 Hz, 1H), 7.00 (d, *J* = 8.7 Hz, 1H), 6.87 (d, *J* = 3.7 Hz, 1H), 2.51 (s, 3H), 2.40 (s, 3H). **^{13}C NMR** (126 MHz, methanol-*d4*) *δ* 207.2, 198.6, 173.4, 163.9, 159.2, 149.3, 138.4, 132.2, 127.8, 126.7, 123.0, 122.1, 119.1, 108.4, 31.6, 25.9. **HPLC** (method C) (λ = 254 nm), 100%; (λ = 214 nm), 100%; ^{t}R =7.73 min (column 2).

(*E*)-2-Hydroxy-5-{5-[3-(1-methylpyrrol-2-yl)-3-oxoprop-1-en-1-yl]furan-2-yl}benzoic acid (S4).

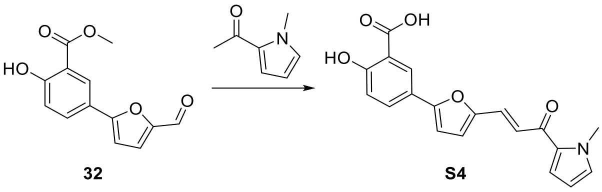

A solution of 2-acetyl-1-methylpyrrol (106 μL, 0.9 mmol, 1.5 equiv) in KOH (50 equiv)/EtOH was prepared and stirred. After 10 min, a solution of **32** (150 mg, 0.6 mmol, 1 equiv) in EtOH was added dropwise. The reaction was stirred at 50 ºC during 24 h, after which, the reaction was quenched by addition of HCl 3N until pH3. The organic solvents were evaporated under reduced pressure and 20 mL of AcOEt were added. The organic layer was washed with water and brine, dried over $MgSO_4$, filtered and concentrated under reduced pressure. The crude product was purified by FCC **^{1}H NMR** (400 MHz, methanol-d_4) δ 8.33 (d, J = 2.1 Hz, 1H), 7.85 (dd, J = 8.6, 2.1 Hz, 1H), 7.46 (d, J = 15.3 Hz, 1H), 7.40 (d, J = 15.3 Hz, 1H), 7.29 (dd, J = 4.2, 1.6 Hz, 1H), 7.05 (pt, J = 1.9 Hz, 1H), 6.96 (d, J = 8.6 Hz, 1H), 6.87 (d, J = 3.5 Hz, 1H), 6.79 (d, J = 3.5 Hz, 1H), 6.22 (dd, J = 4.1, 2.5 Hz, 1H), 4.00 (s, 3H). **^{13}C NMR** (101 MHz, methanol-d_4) δ 181.2, 173.8, 163.2, 157.4, 152.1, 133.8, 133.3, 131.4, 128.9, 127.7, 122.4, 121.4, 121.1, 119.1, 118.6, 109.7, 107.7, 38.0. **HRMS** (TOF ES$^+$): *m/z* calcd for $C_{19}H_{16}NO$ $(M+H)^+$ 338.1023, found 338.1013 (deviation +2.9 ppm). **HPLC** (method E) (λ = 254 nm), 100%; (λ = 214 nm), 100%; ^{t}R =4.98 min (column 2).

(*E*)-5-{5-[3-(furan-2-yl)-3-oxoprop-1-enyl]furan-2-yl}-2-hydroxybenzoic acid (S5).

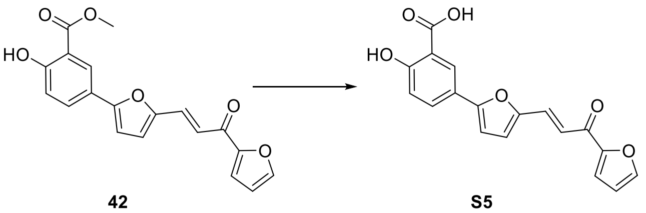

Compound **42** (78 mg, 0.23 mmol, 1 equiv) was dissolved in pyridine (8 mL) and heated up to reflux (130 ºC) overnight. After this time, pyridine was removed using a high vacuum rotavapor. The residue was dissolved in AcOEt and the remaining trace of pyridine was extracted with aqueous HCl 1N. The organic layer was washed with brine, dried over $MgSO_4$, filtered and concentrated under reduced pressure. FCC purification: Gradient elution using petrol ether:DCM:MeOH (49:49:2 → 45:50:5). All mobile phases were acidified with 1% AcOH. **^{1}H NMR** (400 MHz, methanol-d_4) δ 8.32 (d, J = 2.3 Hz,

1H), 7.93 (dd, J = 8.7, 2.3 Hz, 1H), 7.86 (m, 1H), 7.63 (d, J = 15.4 Hz, 1H), 7.53 (pd, J = 3.6 Hz, 1H), 7.42 (d, J = 15.4 Hz, 1H), 7.02 (d, J = 8.6 Hz, 1H), 7.00 (d, J = 3.6 Hz, 1H), 6.87 (d, J = 3.6 Hz, 1H), 6.71 (dd, J = 3.5, 1.7 Hz, 1H). **^{13}C NMR** (101 MHz, methanol-*d*$_4$) δ 179.5, 163.7, 157.9, 155.0, 152.0, 148.9, 132.4, 131.1, 127.7, 122.5, 121.1, 119.4, 119.0, 118.5, 113.8, 108.5. **HRMS** (TOF ES$^+$): *m/z* calcd for $C_{18}H_{13}O_6$ (M+H)$^+$ 325.0712, found 325.0702 (deviation -3.1 ppm). **HPLC** (method D) (λ = 254 nm), 98.1%; (λ = 214 nm), 97.8%; ^{t}R =7.00 min (column 2).

2-Hydroxy-5-{5-[3-hydroxy-3-(*p*-trifluoromethylphenyl)propyl]tetrahydrofuran-2-yl}benzoic acid (S6).

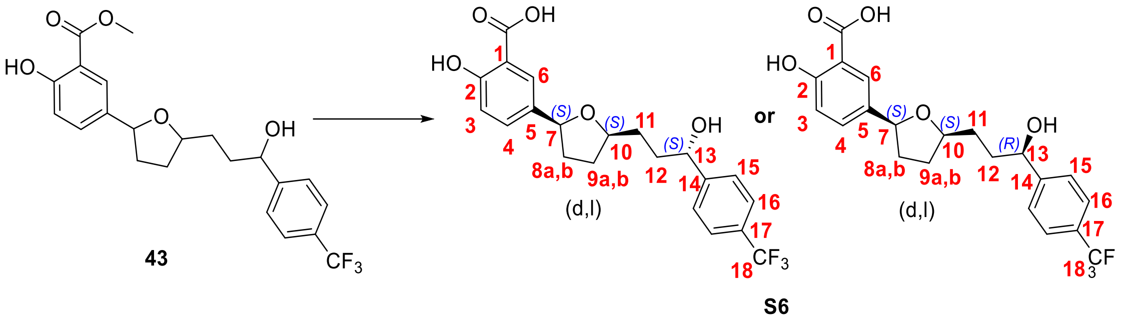

Compound **43** (70 mg, 0.17 mmol, 1 equiv) was dissolved in a small volume of DCM and afterwards diluted with MeOH (4 mL). Subsequently, NaOH 10 N (0.85 mL, 0.85 mmol, 5 equiv) was added and the mixture was heated at 50 ºC overnight. After this time, the reaction was let to cool at rt and quenched by addition of HCl (5%) to a final pH of 4. Organic solvents when then removed under vacuum and the aqueous phase was extracted with AcOEt (x2). The combined organic phases were washed with brine, dried over MgSO4, filtered and concentrated under reduced pressure. FCC purification: Gradient elution using DCM:MeOH (100:0 → 8:2). Compound S6 was purified as a single *cis* diastereomer (d,l). **^{1}H NMR** (500 MHz, methanol-*d*$_4$) δ 7.87 (bs, 1H, H6), 7.63 (d, J = 7.9 Hz, 2H, H-16), 7.55 (d, J = 8.1 Hz, 2H, H-15), 7.30 (m, 1H, H-4), 6.79 (d, J = 8.4 Hz, 1H H-3), 4.77 (m, 2H, H-7, H-13), 4.00 (m, 1H, H-10), 2.23 (m, 1H), 2.11 (m, 1H), 1.92 (m, 1H), 1.84-1.72 (m, 3H), 1.71-1.60 (m, 2H). **^{13}C NMR** (126 MHz, methanol-*d*$_4$) δ 177.4, 162.1, 151.2, 133.7, 132.6, 130.4, 130.1, 129.6, 127.65, 127.63, 126.16, 126.14, 118.4, 117.4, 82.4, 81.1, 80.9, 74.3, 74.2, 36.9, 36.8, 35.04, 35.01, 33.2, 33.1, 32.43, 32.39. **HRMS** (TOF ES$^-$): *m/z* calcd for $C_{21}H_{20}O_5F_3$ (M-H)$^-$ 409.1263, found 409.1248 (deviation -3.7 ppm). **HPLC** (method E) (λ = 254 nm), 100%; (λ = 214 nm), 100%; ^{t}R =7.00 min (column 2).

Methyl (*E*)-2-hydroxy-5-[5-(4,4,4-trifluoro-3-oxobut-1-en-1-yl)furan-2-yl]benzoate (40).

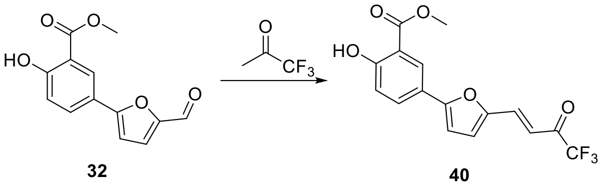

A solution of **32** (100 mg, 0.40 mmol, 1 eq) in benzene (3 mL, 7 mL/mmol) was prepared and stirred at rt. On this solution, AcOH (35 μL, 0.61 mmol, 1.5 eq) and piperidine (40 μL, 0.40 mmol, 1 eq) were added. The mixture was then cooled to 0 ºC (ice bath) and a solution of 1,1,1-trifluoro-2-propanone (141 μL, 1.62 mmol, 4 eq) in benzene (3 mL, 2 mL/mmol) was added. The reaction was then stirred at rt for 24h. After this time, the reaction crude was suspended in AcOEt and washed with brine. The organic layer was dried over $MgSO_4$ anhydrous, filtered and evaporated under vacuum before FCC purification: Gradient elution using mixtures of hexane:AcOEt (10:0→8:2). Orange solid, 75% yield (102 mg, 0.3 mmol). **^{1}H NMR** (500 MHz, chloroform-*d*) δ 11.00 (s, 1H), 8.24 (d, J = 2.3 Hz, 1H), 7.86 (dd, J = 8.7, 2.3 Hz, 1H), 7.68 (d, J = 15.4 Hz, 1H), 7.08 (d, J = 8.7 Hz, 1H), 6.98 (d, J = 3.6 Hz, 1H), 6.91 (d, J = 15.4 Hz, 1H), 6.75 (d, J = 3.7 Hz, 1H), 4.03 (s, 3H). **^{13}C NMR** (126 MHz, chloroform-*d*) δ 179.8 (q, J = 35.1 Hz), 170.2, 162.6, 157.9, 150.0, 134.4, 132.3, 126.7, 123.1, 121.1, 118.8, 116.7 (q, J = 290.5 Hz), 113.0, 112.9, 108.2, 52.9. **HRMS** (TOF ES$^-$): *m/z* calcd for $C_{16}H_{10}O_5F_3$ (M-H)$^-$ 339.0480, found 339.0464 (deviation -4.7 ppm).

Methyl 5-[5-(2-acetyl-3-oxobut-1-en-1-yl)furan-2-yl]-2-hydroxy-benzoate (41).

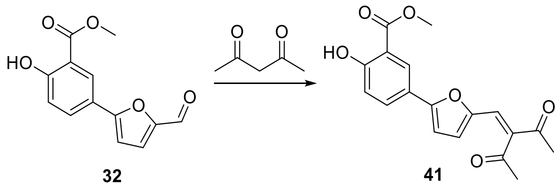

A solution of **32** (91 mg, 0.37 mmol, 1 equiv) in dry DCM (13 mL/mmol) was prepared under argon atmosphere, on activated molecular sieves (4 Å). On this solution, AcOH (32 μL, 0.55 mmol, 1.5 eq), piperidine (36 μL, 0.37 mmol, 1 equiv) and pentane-2,4-dione (76 μL, 0.74 mmol, 2 equiv) were added in this order. The mixture was then stirred at rt for 4 h. After this time, the reaction crude was washed with brine. The organic layer was dried over $MgSO_4$ anhydrous, filtered and evaporated under vacuum before FCC purification. FCC purification: gradient elution using mixtures of hexane:AcOEt

(10:0→7:3). Yellow solid, 94% yield (115 mg, 0.35 mmol). **^{1}H NMR** (500 MHz, chloroform-*d*) *δ* 10.90 (s, 1H), 8.16 (d, *J* = 2.3 Hz, 1H), 7.72 (dd, *J* = 8.7, 2.3 Hz, 1H), 7.14 (s, 1H), 7.04 (d, *J* = 8.7 Hz, 1H), 6.85 (d, *J* = 3.6 Hz, 1H), 6.67 (d, *J* = 3.7 Hz, 1H), 4.01 (s, 3H), 2.54 (s, 3H), 2.39 (s, 3H). **^{13}C NMR** (126 MHz, chloroform-*d*) *δ* 204.5, 195.8, 170.2, 162.2, 157.5, 148.0, 137.7, 131.8, 126.3, 124.8, 121.3, 121.0, 118.8, 112.9, 107.4, 52.8, 31.6, 26.3. **HRMS** (TOF ES$^-$): *m/z* calcd for $C_{18}H_{15}O_6$ (M-H)$^-$ 327.0869, found 327.0870 (deviation +0.3 ppm).

Methyl (*E*)-2-Hydroxy-5-{5-[3-(furan-2-yl)-3-oxoprop-1-en-1-yl]furan-2-yl}benzoic acid (42).

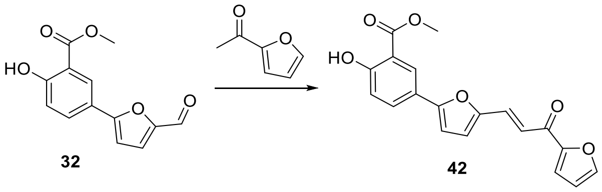

Compound **32** (95 mg, 0.38 mmol, 1 equiv) was dissolved in a minimum amount of DCM. To this solution, MeOH was added (5 mL) and the temperature was lowered to 0 ºC using an ice bath. At this temperature, an aqueous solution of NaOH (10 mM) (190 µL, 1.9 mmol, 5 equiv) was added followed by 2-furfural (64 mg, 0.58 mmol, 1.5 equiv). The ice bath was then removed and the reaction was let to stir at rt for 24 h. After this time, the reaction was quenched by addition of HCl 5% (5 mL). Organic solvents were removed under vacuum and the remaining aqueous phase was extracted with AcOEt (x2). The final organic layer was washed with brine, dried over anhydrous $MgSO_4$, filtered and evaporated under vacuum. FCC purification: Gradient elution using mixtures petrol ether:AcOEt (95:5→8:2). **^{1}H NMR** (500 MHz, chloroform-*d*) *δ* 10.92 (s, 1H), 8.22 (d, *J* = 2.3 Hz, 1H), 7.88 (dd, *J* = 8.8, 2.3 Hz, 1H), 7.67 (m, 1H), 7.65 (d, *J* = 15.4 Hz, 1H), 7.38-7.33 (m, 2H), 7.07 (d, *J* = 8.7 Hz, 1H), 6.81 (d, *J* = 3.6 Hz, 1H), 6.69 (d, *J* = 3.5 Hz, 1H), 6.60 (dd, *J* = 3.6, 1.7 Hz, 1H), 4.03 (s, 3H). **^{13}C NMR** (126 MHz, chloroform-*d*) *δ* 177.9, 170.3, 162.0, 155.8, 154.0, 151.0, 146.5, 132.0, 129.8, 126.2, 121.8, 119.3, 118.6, 118.2, 117.4, 112.9, 112.7, 107.5, 52.8. **HRMS** (TOF ES$^+$): *m/z* calcd for $C_{19}H_{15}O_6$ (M+H)$^+$ 339.0869, found 339.0879 (deviation +2.9 ppm).

Methyl 2-hydroxy-5-{5-[3-hydroxy-3-(*p*-trifluoromethylphenyl)propyl] tetrahydrofuran-2-yl}benzoate (43).

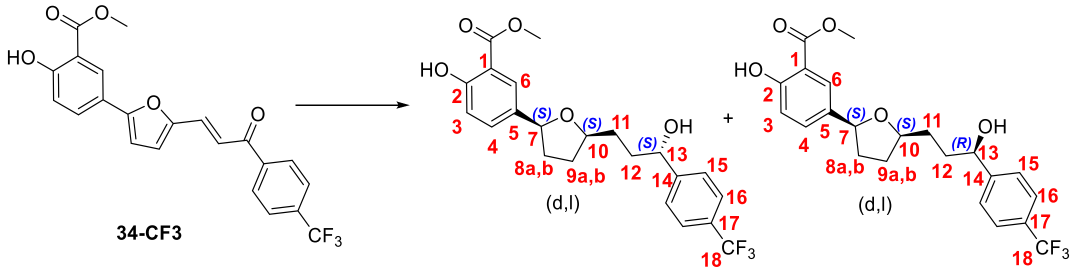

A solution of **34-CF3** (84 mg, 0.20 mmol, 1 equiv) in THF (3 mL) was prepared and stirred at rt. Next, palladium on charcoal (11 mg, 0.01 mmol, 5mol%) was added and hydrogen (g) was then bubbled through the solution for 1 h (disappearance of the starting material). The mixture was then filtered through Celite® and the filtrate was concentrated under reduced pressure. FCC purification: Gradient elution using mixtures of hexane:AcOEt (95:5→1:1). Yield 42% yield (35 mg, 0.08 mmol). Compound **43** was obtained as a mixture of two *cis* diastereoisomers in 1:1 proportion. **^{1}H NMR** (500 MHz, chloroform-*d*) δ 10.71 (s, 1H, Ph-OH), 10.71 (s, 1H, Ph-OH), 7.78 (bd, J = 2.3 Hz, 2H, H-4), 7.58 (d, J = 8.1 Hz, 2H, H-16), 7.57 (d, J = 8.1 Hz, 2H, H-16), 7.49-7.39 (m, 6H, H-4, H-15), 6.95 (d, J = 8.5 Hz, 2H, H-3), 4.85-4.77 (m, 4H, H-7, H-13), 4.08-3.96 (m, 2H, H-10), 3.94 (s, 3H, OCH_3), 3.93 (s, 3H, OCH_3), 3.5 (bb, 1H, OH), 3.1 (bb, 1H, OH), 2.31-2.22 (m, 2H, H-8a), 2.13-2.04 (m, 2H, H-9a), 2.00-1.87 (4H, H-12), 1.83-1.74 (m, 6H, H-8b, H-11), 1.72-1.59 (m, 2H, H-9b). **^{13}C NMR** (126 MHz, chloroform-*d*) δ 170.5 (CO), 161.2 (C2), 148.9 (C14), 133.7 (CH-4), 129.5 (C5), 127.3 (CH-6), 126.1 (CH-15), 125.4 (CH-16), 123.2 (C-14), 117.8 (CH-3), 111.9 (C-1), 80.6 (CH-7), 80.0 (CH-10), 73.8 (CH-13), 73.1 (CH-13), 52.3 (OCH_3), 36.9 (CH_2-12), 36.0 (CH_2-12), 33.9 (CH_2-8), 32.7 (CH_2-11), 31.5 (CH_2-9), 31.3 (CH_2-11). **HRMS** (TOF ES$^-$): *m/z* calcd for $C_{22}H_{22}F_3O_5$ $(M-H)^-$ 423.1414, found 423.1434 (deviation -4.7 ppm).

S2. Screening on recombinant enzymes

Table S1. Relative activities of recombinant enzymes glycolate oxidase (GO) and lactate dehydrogenase A (LDHA) after treatment with the studied compounds (concentration = 10 µM).

Compound	*h*GO (%)	*h*LDHA (%)
**1**	56	37
**2**	1	4
**4**	35	68
**5**	33	69
**6**	36	7
**7**	33	45
**8**	23	55
**9**	2	7
**10**	38	92
**11**	15	18
**12**	5	17
**13**	12	29
**14**	2	22
**15**	14	36
**16**	17	6
**17**	1	38
**18**	2	1
**19**	2	15
**20**	1	1
**21**	0	8
**22**	0	7
**23**	0	5
**24**	0	34
**25**	1	0
**26**	6	21
**27**	51	17
**28**	77	12
**29**	4	0
**S1**	58	66
**S2**	71	8
**S3**	84	59
**S4**	39	90
**S5**	50	56
**S6**	82	24

S3. Determination of IC_{50}s on recombinant *h*GO using a kinetic fluorometric protocol with Amplex® Red

Final IC_{50} values were obtained from the mean of four replicates for each inhibitor. In each IC_{50} replicate we used ten different inhibitor concentrations and 180 µM substrate concentration (glycolate). The relative enzymatic activity (%) obtained at each inhibitor concentration was calculated by comparison of the initial velocity (v_0) at each inhibitor concentration, with the v_0 value observed for in the absence of inhibitor (100% enzymatic activity). Before, v_0 was determined as the slope calculated in the linear interval of the "product *vs* time" graph representing the progression of the enzymatic reaction, at each inhibitor concentration and in the absence of inhibitor. This linear interval was of 4 min, after a total measuring time of 15 min.

Compound 2

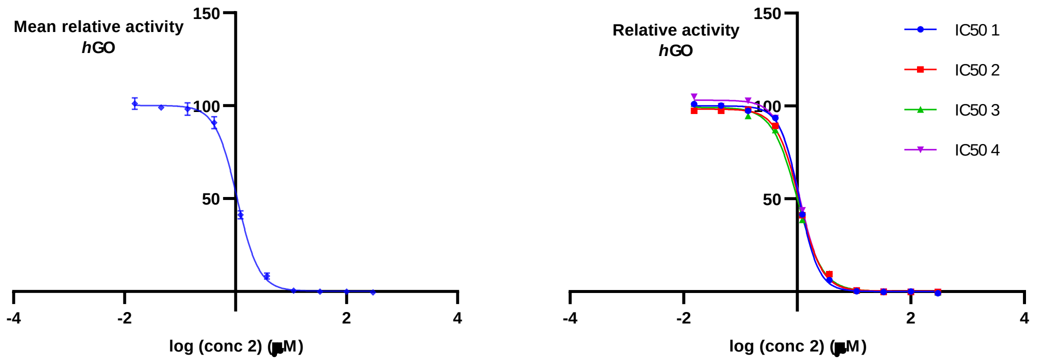

Figure S2. Left: Non-linear "log (conc. **2**) *vs* relative *h*GO activity" plot (media of four replicates). Error bars show standard deviation. Right: Non-linear "log (conc. **2**) *vs* relative *h*GO activity" plots of the four replicates (using ten concentrations of inhibitor and 180 µM glycolate).

Table S2. Statistical parameters of the IC_{50} value for inhibition of *h*GO obtained for **2**, using ten inhibitor concentrations and 180 µM substrate (glycolate) concentration (four replicates).

**IC_{50} (uM)**	1.05 ± 0.06
95% Confidence Intervals	1.023 to 1.115
R square	0.9979

Compound 4

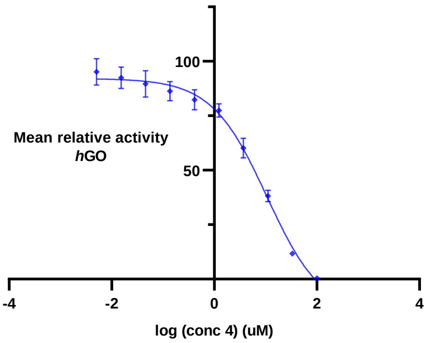

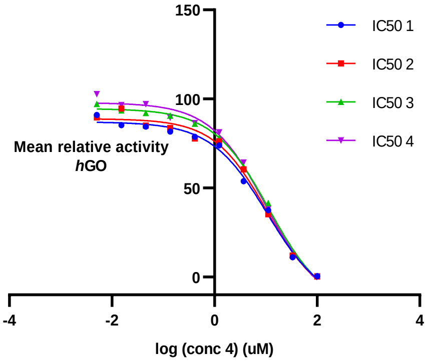

Figure S3. Left: Non-linear "log (conc. **4**) *vs* relative *h*GO activity" plot (media of four replicates). Error bars show standard deviation. Right: Non-linear "log (conc. **4**) *vs* relative *h*GO activity" plots of the four replicates (using ten concentrations of inhibitor and 180 µM glycolate).

Table S3. Statistical parameters of the IC_{50} value for inhibition of *h*GO obtained for **4**, using ten inhibitor concentrations and 180 µM substrate (glycolate) concentration (four replicates).

**IC_{50} (uM)**	10.61 ± 0.66
95% Confidence Intervals	7.665 to 17.99
R square	0.9845

Compound 5

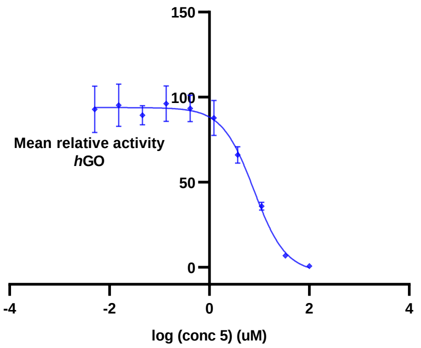

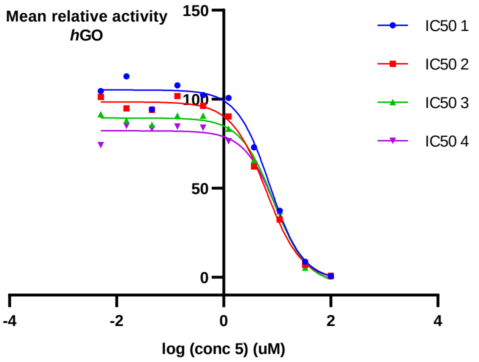

Figure S4. Left: Non-linear "log (conc. **5**) *vs* relative *h*GO activity" plot (media of four replicates). Error bars show standard deviation. Right: Non-linear "log (conc. **5**) *vs* relative *h*GO activity" plots of the four replicates (using ten concentrations of inhibitor and 180 µM glycolate).

Table S4. Statistical parameters of the IC_{50} value for inhibition of *h*GO obtained for **5**, using ten inhibitor concentrations and 180 µM substrate (glycolate) concentration (four replicates).

**IC_{50} (uM)**	7.92 ± 1.43
95% Confidence Intervals	5.936 to 10.74
R square	0.9588

Compound 8

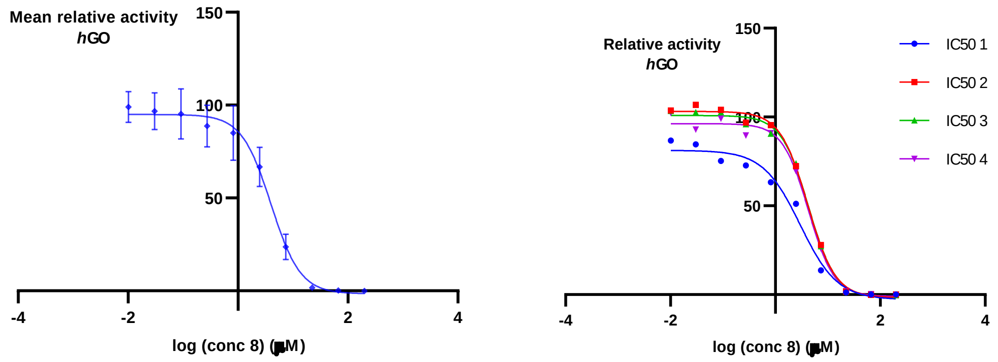

Figure S5. Left: Non-linear "log (conc. **8**) *vs* relative *h*GO activity" plot (media of four replicates). Error bars show standard deviation. Right: Non-linear "log (conc. **8**) *vs* relative *h*GO activity" plots of the four replicates (using ten concentrations of inhibitor and 180 µM glycolate).

Table S5. Statistical parameters of the IC_{50} value for inhibition of *h*GO obtained for **8**, using ten inhibitor concentrations and 180 µM substrate (glycolate) concentration (four replicates).

**IC_{50} (uM)**	3.91 ± 0.62
95% Confidence Intervals	3.145 to 5.036
R square	0.9609

Compound 9

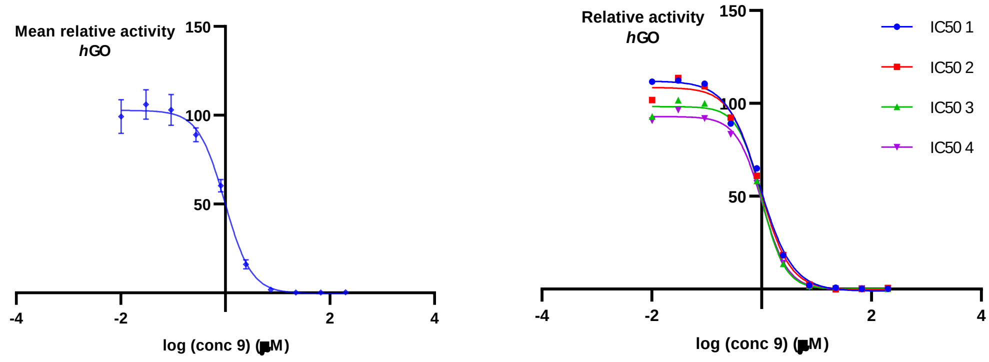

Figure S6. Left: Non-linear "log (conc. **9**) *vs* relative *h*GO activity" plot (media of four replicates). Error bars show standard deviation. Right: Non-linear "log (conc. **9**) *vs* relative *h*GO activity" plots of the four replicates (using ten concentrations of inhibitor and 180 µM glycolate).

Table S6. Statistical parameters of the IC_{50} value for inhibition of *h*GO obtained for **9**, using ten inhibitor concentrations and 180 µM substrate (glycolate) concentration (four replicates).

**IC_{50} (uM)**	0.97 ± 0.05
95% Confidence Intervals	0.8616 to 1.101
R square	0.9882

Compound 10

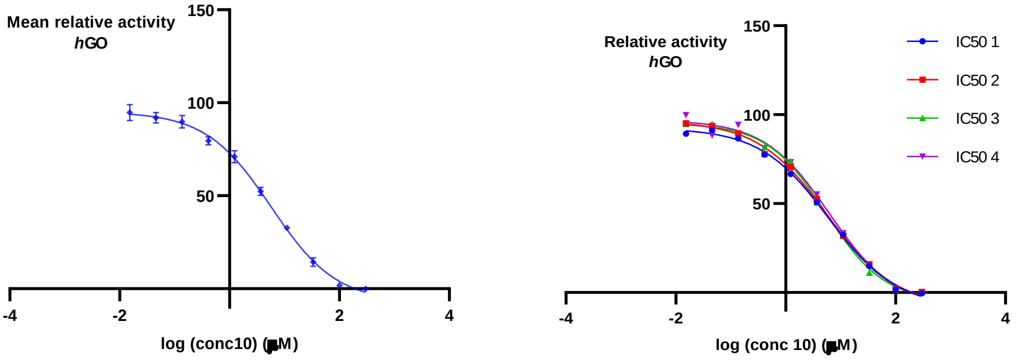

Figure S7. Left: Non-linear "log (conc. **10**) *vs* relative *h*GO activity" plot (media of four replicates). Error bars show standard deviation. Right: Non-linear "log (conc. **10**) *vs* relative *h*GO activity" plots of the four replicates (using ten concentrations of inhibitor and 180 µM glycolate).

Table S7. Statistical parameters of the IC_{50} value for inhibition of *h*GO obtained for **10**, using ten inhibitor concentrations and 180 µM substrate (glycolate) concentration (four replicates).

**IC_{50} (uM)**	5.76 ± 0.40
95% Confidence Intervals	4.870 to 6.746
R square	0.9949

Compound 11

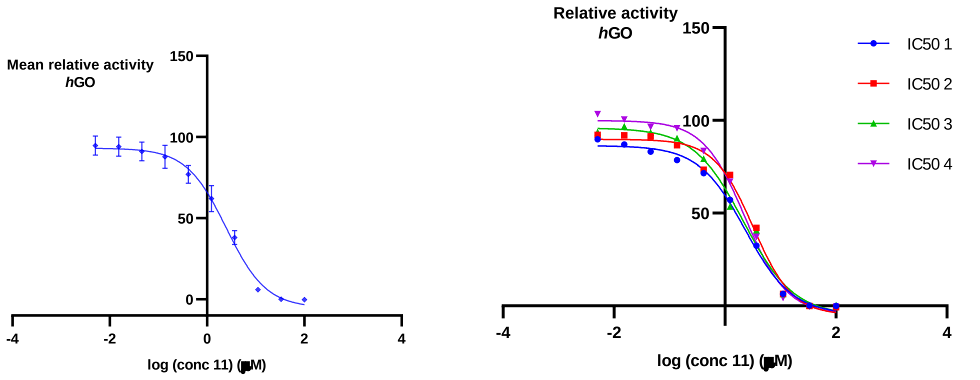

Figure S8. Left: Non-linear "log (conc. **11**) *vs* relative *h*GO activity" plot (media of four replicates). Error bars show standard deviation. Right: Non-linear "log (conc. **11**) *vs* relative *h*GO activity" plots of the four replicates (using ten concentrations of inhibitor and 180 µM glycolate).

Table S8. Statistical parameters of the IC_{50} value for inhibition of *h*GO obtained for **11**, using ten inhibitor concentrations and 180 µM substrate (glycolate) concentration (four replicates).

**IC_{50} (uM)**	2.51 ± 0.49
95% Confidence Intervals	2.006 to 3.090
R square	0.9813

Compound 12

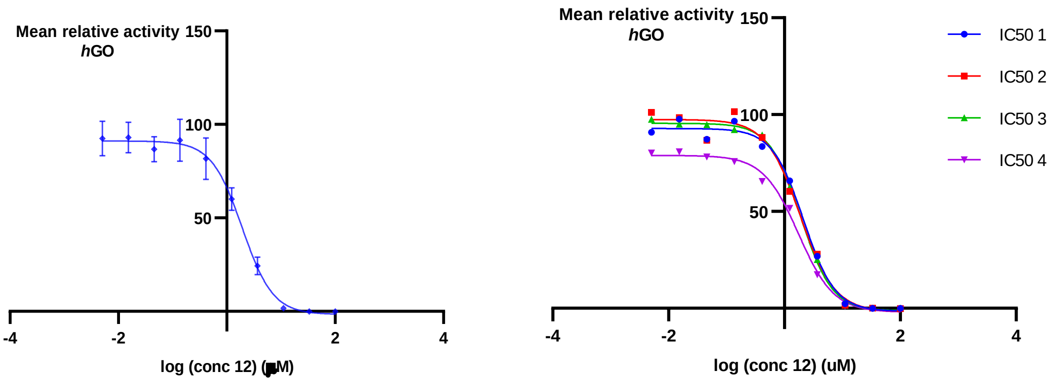

Figure S9. Left: Non-linear "log (conc. **12**) *vs* relative *h*GO activity" plot (media of four replicates). Error bars show standard deviation. Right: Non-linear "log (conc. **12**) *vs* relative *h*GO activity" plots of the four replicates (using ten concentrations of inhibitor and 180 µM glycolate).

Table S9. Statistical parameters of the IC_{50} value for inhibition of *h*GO obtained for **12**, using ten inhibitor concentrations and 180 µM substrate (glycolate) concentration (four replicates).

**IC_{50} (uM)**	1.92 ± 0.16
95% Confidence Intervals	1.581 to 2.344
R square	0.9742

Compound 13

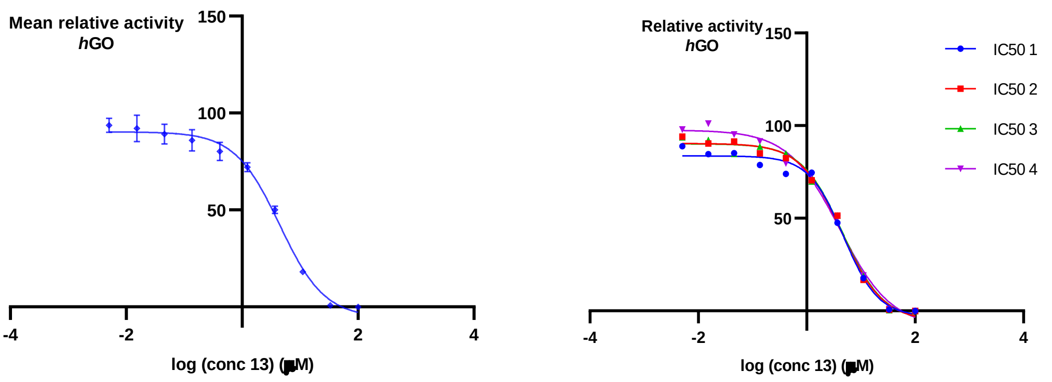

Figure S10. Left: Non-linear "log (conc. **13**) *vs* relative *h*GO activity" plot (media of four replicates). Error bars show standard deviation. Right: Non-linear "log (conc. **13**) *vs* relative *h*GO activity" plots of the four replicates (using ten concentrations of inhibitor and 180 µM glycolate).

Table S10. Statistical parameters of the IC_{50} value for inhibition of *h*GO obtained for **13**, using ten inhibitor concentrations and 180 µM substrate (glycolate) concentration (four replicates).

**IC_{50} (uM)**	4.44 ± 0.30
95% Confidence Intervals	3.755 to 5.344
R square	0.9870

Compound 14

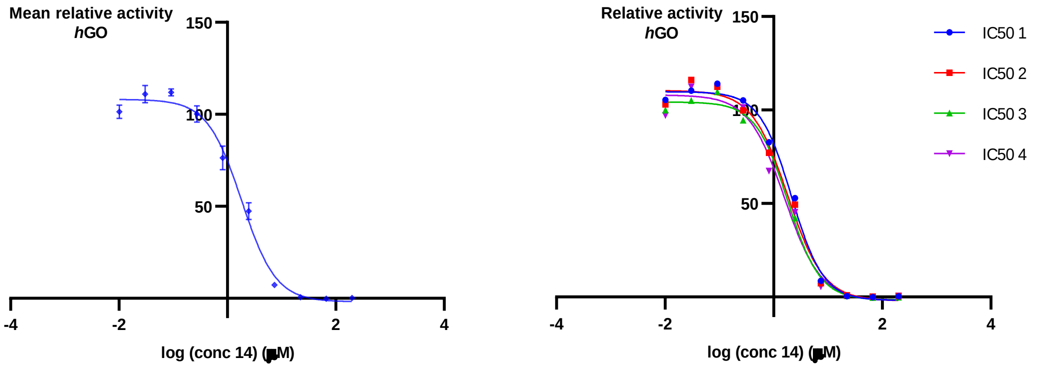

Figure S11. Left: Non-linear "log (conc. **14**) *vs* relative *h*GO activity" plot (media of four replicates). Error bars show standard deviation. Right: Non-linear "log (conc. **14**) *vs* relative *h*GO activity" plots of the four replicates (using ten concentrations of inhibitor and 180 µM glycolate).

Table S11. Statistical parameters of the IC_{50} value for inhibition of *h*GO obtained for **14**, using ten inhibitor concentrations and 180 µM substrate (glycolate) concentration (four replicates).

**IC_{50} (uM)**	1.83 ± 0.22
95% Confidence Intervals	1.594 to 2.105
R square	0.9886

Compound 15

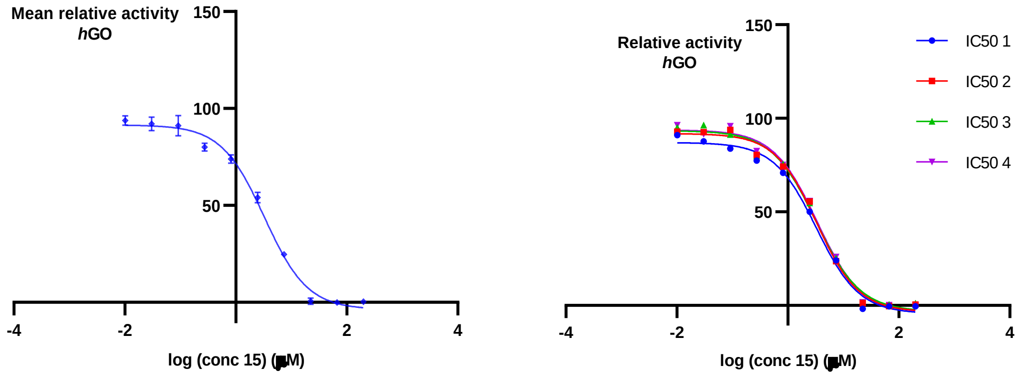

Figure S12. Left: Non-linear "log (conc. **15**) *vs* relative *h*GO activity" plot (media of four replicates). Error bars show standard deviation. Right: Non-linear "log (conc. **15**) *vs* relative *h*GO activity" plots of the four replicates (using ten concentrations of inhibitor and 180 µM glycolate).

Table S12. Statistical parameters of the IC_{50} value for inhibition of *h*GO obtained for **15**, using ten inhibitor concentrations and 180 µM substrate (glycolate) concentration (four replicates).

**IC_{50} (uM)**	3.28 ± 0.06
95% Confidence Intervals	2.826 to 3.800
R square	0.9904

Compound 16

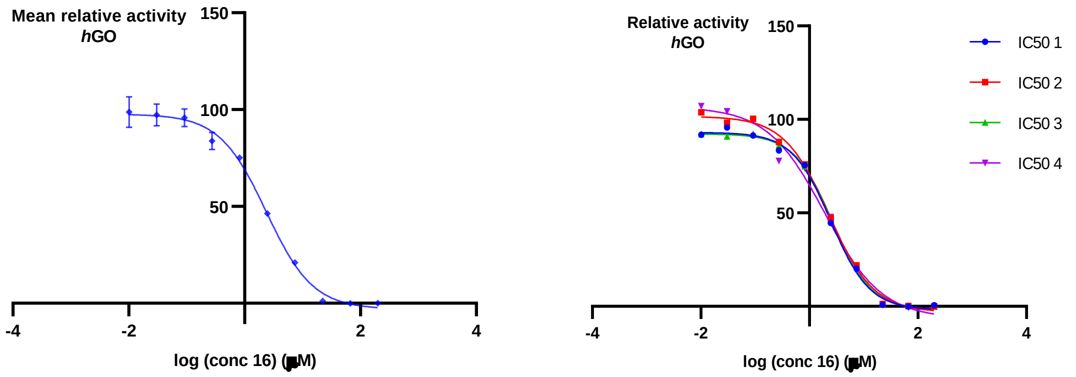

Figure S13. Left: Non-linear "log (conc. **16**) *vs* relative *h*GO activity" plot (media of four replicates). Error bars show standard deviation. Right: Non-linear "log (conc. **16**) *vs* relative *h*GO activity" plots of the four replicates (using ten concentrations of inhibitor and 180 µM glycolate).

Table S13. Statistical parameters of the IC_{50} value for inhibition of *h*GO obtained for **16**, using ten inhibitor concentrations and 180 µM substrate (glycolate) concentration (four replicates).

**IC_{50} (uM)**	2.33 ± 0.33
95% Confidence Intervals	2.034 to 2.745
R square	0.9906

Compound 17

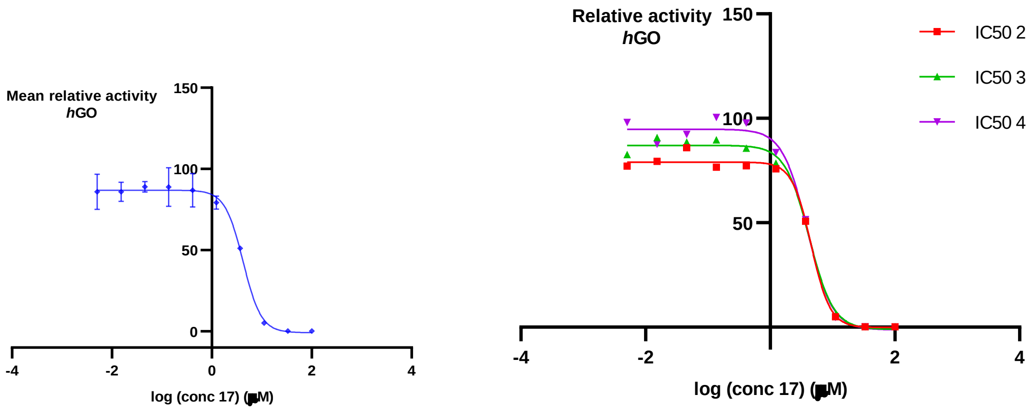

Figure S14. Left: Non-linear "log (conc. **17**) *vs* relative *h*GO activity" plot (media of three replicates). Error bars show standard deviation. Right: Non-linear "log (conc. **17**) *vs* relative *h*GO activity" plots of the three replicates (using ten concentrations of inhibitor and 180 µM glycolate).

Table S14. Statistical parameters of the IC_{50} value for inhibition of *h*GO obtained for **17**, using ten inhibitor concentrations and 180 µM substrate (glycolate) concentration (three replicates).

**IC_{50} (uM)**	4.37 ± 0.22
95% Confidence Intervals	3.637 to 4.947
R square	0.9786

Compound 18

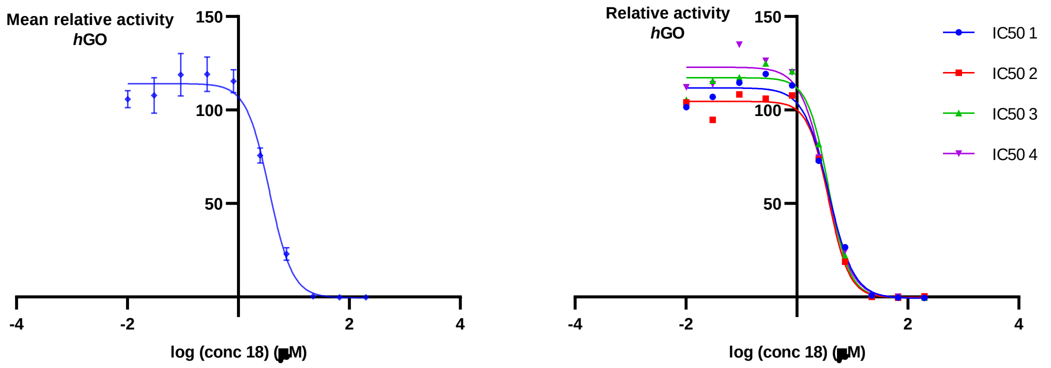

Figure S15. Left: Non-linear "log (conc. **18**) *vs* relative *h*GO activity" plot (media of four replicates). Error bars show standard deviation. Right: Non-linear "log (conc. **18**) *vs* relative *h*GO activity" plots of the four replicates (using ten concentrations of inhibitor and 180 µM glycolate).

Table S15. Statistical parameters of the IC_{50} value for inhibition of *h*GO obtained for **18**, using ten inhibitor concentrations and 180 µM substrate (glycolate) concentration (four replicates).

**IC_{50} (uM)**	3.63 ± 0.21
95% Confidence Intervals	3.139 to 4.184
R square	0.9807

Compound 19

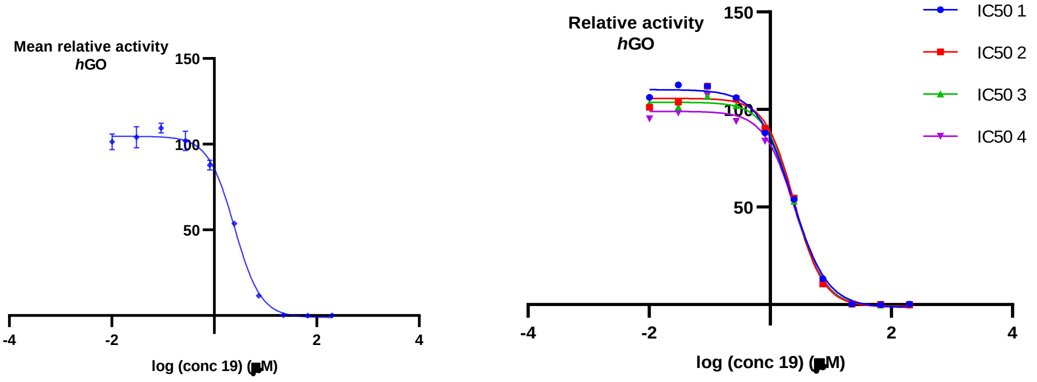

Figure S16. Left: Non-linear "log (conc. **19**) *vs* relative *h*GO activity" plot (media of four replicates). Error bars show standard deviation. Right: Non-linear "log (conc. **19**) *vs* relative *h*GO activity" plots of the four replicates (using ten concentrations of inhibitor and 180 µM glycolate).

Table S16. Statistical parameters of the IC_{50} value for inhibition of *h*GO obtained for **19**, using ten inhibitor concentrations and 180 µM substrate (glycolate) concentration (four replicates).

**IC_{50} (uM)**	2.47 ± 0.11
95% Confidence Intervals	2.257 to 2.696
R square	0.9938

Compound 20

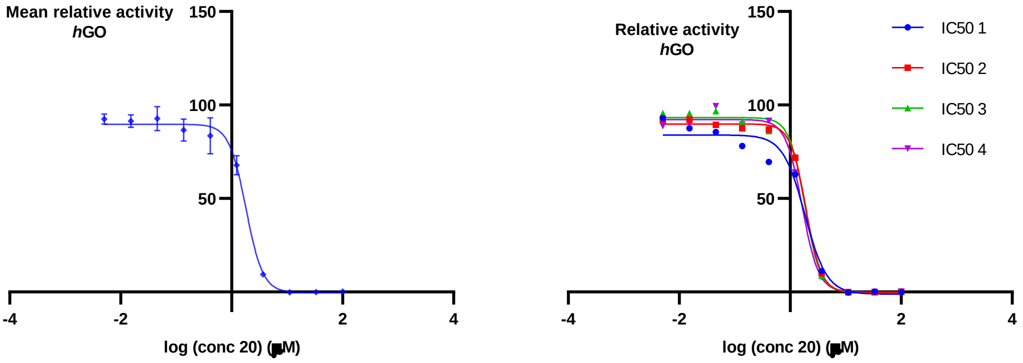

Figure S17. Left: Non-linear “log (conc. **20**) *vs* relative *h*GO activity” plot (media of four replicates). Error bars show standard deviation. Right: Non-linear “log (conc. **20**) *vs* relative *h*GO activity” plots of the four replicates (using ten concentrations of inhibitor and 180 µM glycolate).

Table S17. Statistical parameters of the IC_{50} value for inhibition of *h*GO obtained for **20**, using ten inhibitor concentrations and 180 µM substrate (glycolate) concentration (four replicates).

**IC_{50} (uM)**	1.81 ± 0.12
95% Confidence Intervals	1.626 to 2.019
R square	0.9876

Compound 21

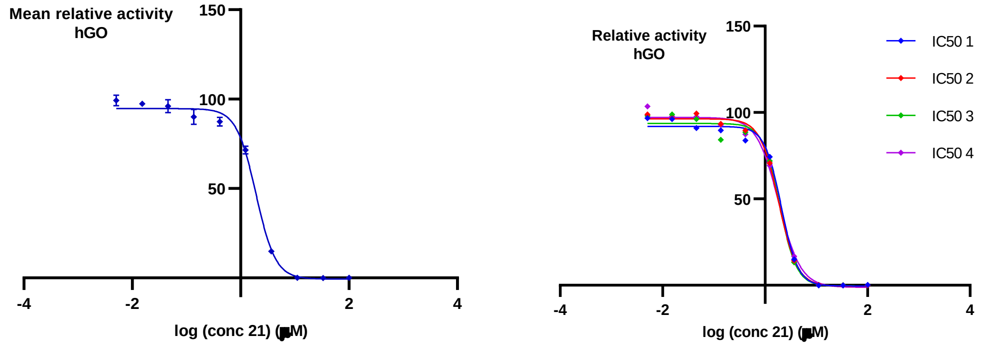

Figure S18. Left: Non-linear “log (conc. **21**) *vs* relative *h*GO activity” plot (media of four replicates). Error bars show standard deviation. Right: Non-linear “log (conc. **21**) *vs* relative *h*GO activity” plots of the four replicates (using ten concentrations of inhibitor and 180 µM glycolate).

Table S18. Statistical parameters of the IC_{50} value for inhibition of *h*GO obtained for **21**, using ten inhibitor concentrations and 180 µM substrate (glycolate) concentration (four replicates).

**IC_{50} (uM)**	1.92 ± 0.10
95% Confidence Intervals	1.773 to 2.079
R square	0.9936

Compound 22

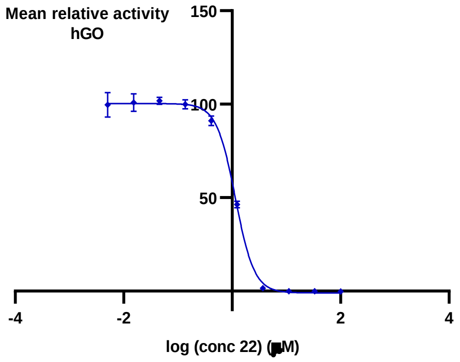

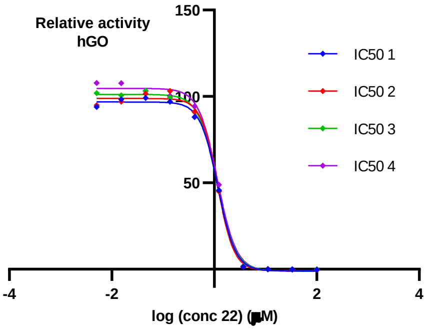

Figure S19. Left: Non-linear "log (conc. **22**) *vs* relative *h*GO activity" plot (media of four replicates). Error bars show standard deviation. Right: Non-linear "log (conc. **22**) *vs* relative *h*GO activity" plots of the four replicates (using ten concentrations of inhibitor and 180 µM glycolate).

Table S19. Statistical parameters of the IC_{50} value for inhibition of *h*GO obtained for **22**, using ten inhibitor concentrations and 180 µM substrate (glycolate) concentration (four replicates).

**IC_{50} (uM)**	1.15 ± 0.02
95% Confidence Intervals	1.092 to 1.211
R square	0.9963

Compound 23

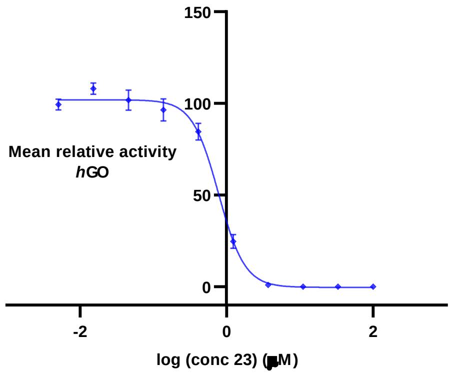

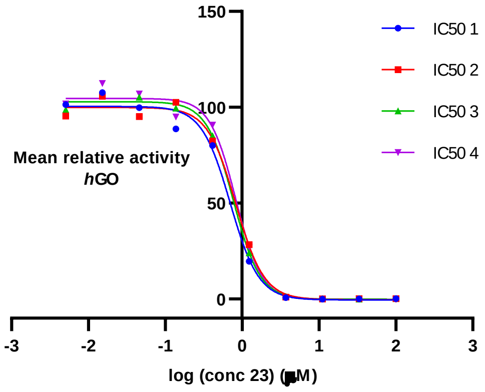

Figure S20. Left: Non-linear "log (conc. **23**) *vs* relative *h*GO activity" plot (media of four replicates). Error bars show standard deviation. Right: Non-linear "log (conc. **23**) *vs* relative *h*GO activity" plots of the four replicates (using ten concentrations of inhibitor and 180 µM glycolate).

Table S20. Statistical parameters of the IC_{50} value for inhibition of *h*GO obtained for **23**, using ten inhibitor concentrations and 180 µM substrate (glycolate) concentration (four replicates).

**IC_{50} (µM)**	0.78 ± 0.06
95% Confidence Intervals	0.7169 to 0.8442
R square	0.9933

Compound 24

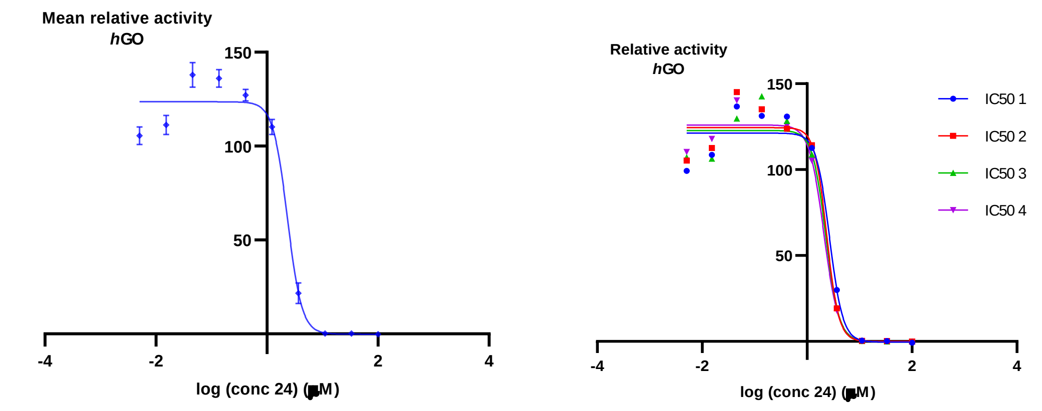

Figure S21. Left: Non-linear "log (conc. **24**) *vs* relative *h*GO activity" plot (media of four replicates). Error bars show standard deviation. Right: Non-linear "log (conc. **24**) *vs* relative *h*GO activity" plots of the four replicates (using ten concentrations of inhibitor and 180 µM glycolate).

Table S21. Statistical parameters of the IC_{50} value for inhibition of *h*GO obtained for **24**, using ten inhibitor concentrations and 180 µM substrate (glycolate) concentration (four replicates).

**IC_{50} (uM)**	2.35 ± 0.25
95% Confidence Intervals	1.965 to 2.774
R square	0.9709

Compound 25

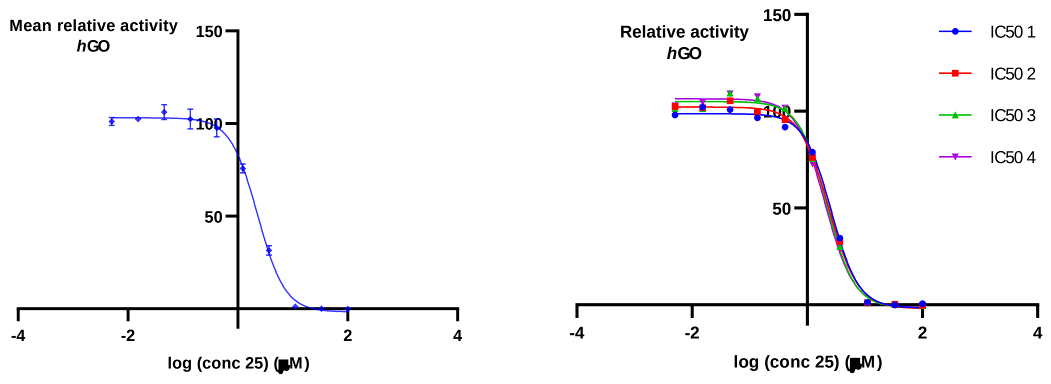

Figure S22. Left: Non-linear "log (conc. **25**) *vs* relative *h*GO activity" plot (media of four replicates). Error bars show standard deviation. Right: Non-linear "log (conc. **25**) *vs* relative *h*GO activity" plots of the four replicates (using ten concentrations of inhibitor and 180 µM glycolate).

Table S22. Statistical parameters of the IC_{50} value for inhibition of *h*GO obtained for **25**, using ten inhibitor concentrations and 180 µM substrate (glycolate) concentration (four replicates).

**IC_{50} (uM)**	2.34 ± 0.25
95% Confidence Intervals	2.122 to 2.475
R square	0.9952

Compound 26

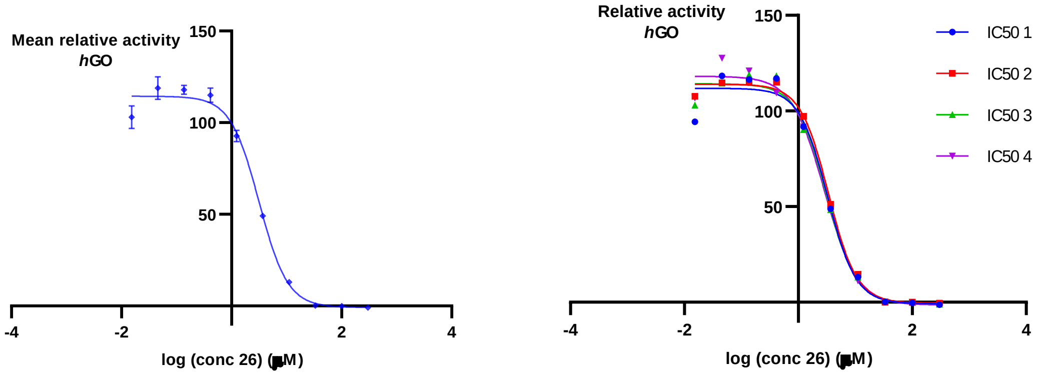

Figure S23. Left: Non-linear "log (conc. **26**) *vs* relative *h*GO activity" plot (media of four replicates). Error bars show standard deviation. Right: Non-linear "log (conc. **26**) *vs* relative *h*GO activity" plots of the four replicates (using ten concentrations of inhibitor and 180 µM glycolate).

Table S23. Statistical parameters of the IC_{50} value for inhibition of *h*GO obtained for **26**, using ten inhibitor concentrations and 180 µM substrate (glycolate) concentration (four replicates).

**IC_{50} (uM)**	3.15 ± 0.22
95% Confidence Intervals	2.81 to 3.55
R square	0.9894

Compound 27

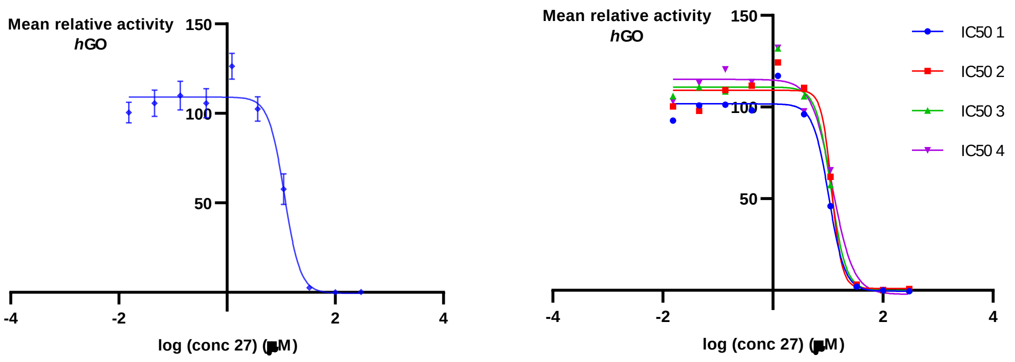

Figure S24. Left: Non-linear "log (conc. **27**) *vs* relative *h*GO activity" plot (media of four replicates). Error bars show standard deviation. Right: Non-linear "log (conc. **27**) *vs* relative *h*GO activity" plots of the four replicates (using ten concentrations of inhibitor and 180 µM glycolate).

Table S24. Statistical parameters of the IC_{50} value for inhibition of *h*GO obtained for **27**, using ten inhibitor concentrations and 180 µM substrate (glycolate) concentration (four replicates).

**IC_{50} (uM)**	11.39 ± 0.70
95% Confidence Intervals	10.18 to 13.01
R square	0.9706

Compound 28

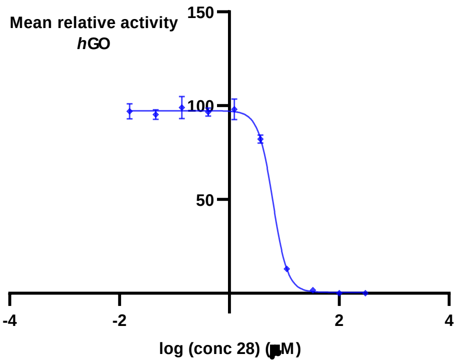

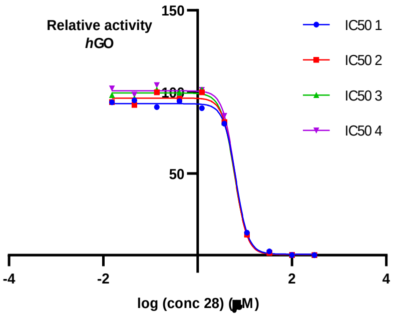

Figure S25. Left: Non-linear "log (conc. **28**) *vs* relative *h*GO activity" plot (media of four replicates). Error bars show standard deviation. Right: Non-linear "log (conc. **28**) *vs* relative *h*GO activity" plots of the four replicates (using ten concentrations of inhibitor and 180 µM glycolate).

Table S25. Statistical parameters of the IC_{50} value for inhibition of *h*GO obtained for **28**, using ten inhibitor concentrations and 180 µM substrate (glycolate) concentration (four replicates).

**IC_{50} (uM)**	6.22 ± 0.20
95% Confidence Intervals	5.823 to 6.625
R square	0.9959

Compound 29

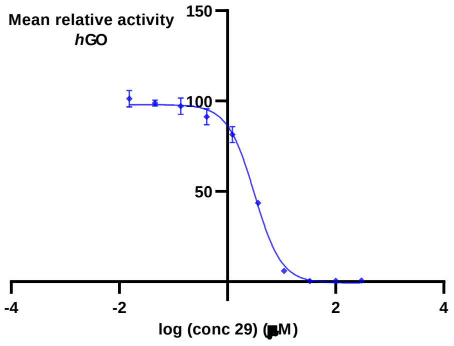

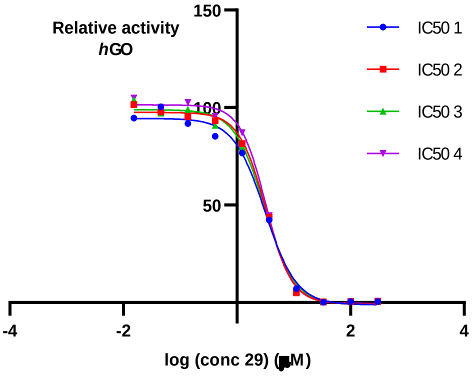

Figure S26. Left: Non-linear "log (conc. **29**) *vs* relative *h*GO activity" plot (media of four replicates). Error bars show standard deviation. Right: Non-linear "log (conc. **29**) *vs* relative *h*GO activity" plots of the four replicates (using ten concentrations of inhibitor and 180 µM glycolate).

Table S26. Statistical parameters of the IC_{50} value for inhibition of *h*GO obtained for **29**, using ten inhibitor concentrations and 180 µM substrate (glycolate) concentration (four replicates).

**IC_{50} (uM)**	3.14 ± 0.07
95% Confidence Intervals	2.900 to 3.425
R square	0.9944

Compound S1

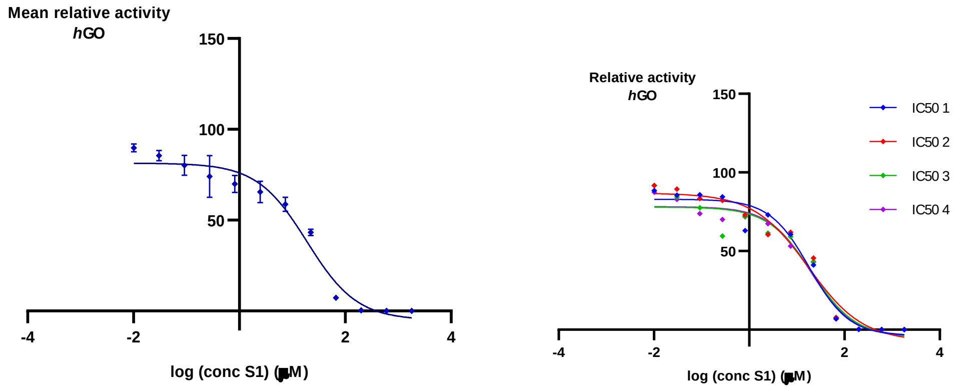

Figure S27. Left: Non-linear "log (conc. **S1**) *vs* relative *h*GO activity" plot (media of four replicates). Error bars show standard deviation. Right: Non-linear "log (conc. **S1**) *vs* relative *h*GO activity" plots of the four replicates (using ten concentrations of inhibitor and 180 µM glycolate).

Table S27. Statistical parameters of the IC_{50} value for inhibition of *h*GO obtained for **S1**, using ten inhibitor concentrations and 180 µM substrate (glycolate) concentration (four replicates).

**IC_{50} (uM)**	18.95 ± 1.34
95% Confidence Intervals	19.40 to 30.20
R square	0.9694

S4. Determination of IC_{50}s on recombinant *h*LDHA using a kinetic fluorometric protocol based on NADH

Compound 2

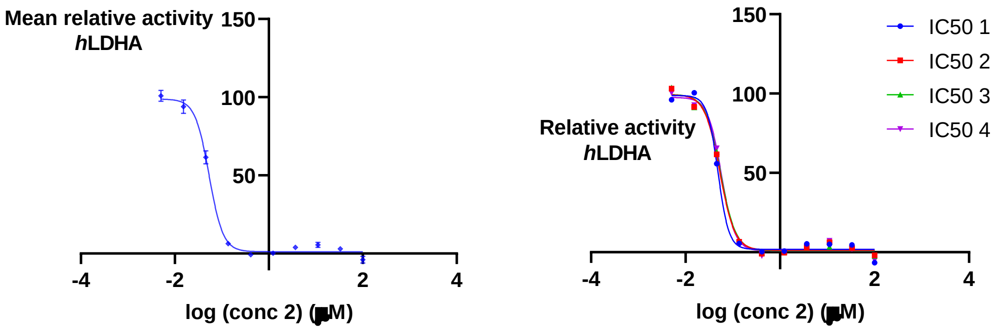

Figure S28. Left: Non-linear "log (conc. **2**) *vs* relative *h*LDHA activity" plot (media of four replicates). Error bars show standard deviation. Right: Non-linear "log (conc. **2**) *vs* relative *h*LDHA activity" plots of the four replicates (using ten concentrations of inhibitor and 180 µM pyruvate).

Table S28. Statistical parameters of the IC_{50} value for inhibition of *h*LDHA obtained for **2**, using ten inhibitor concentrations and 180 µM substrate (pyruvate) concentration (four replicates).

**IC_{50} (uM)**	0.053 ± 0.004
95% Confidence Intervals	0.05031 to 0.0571
R square	0.9925

Compound 6

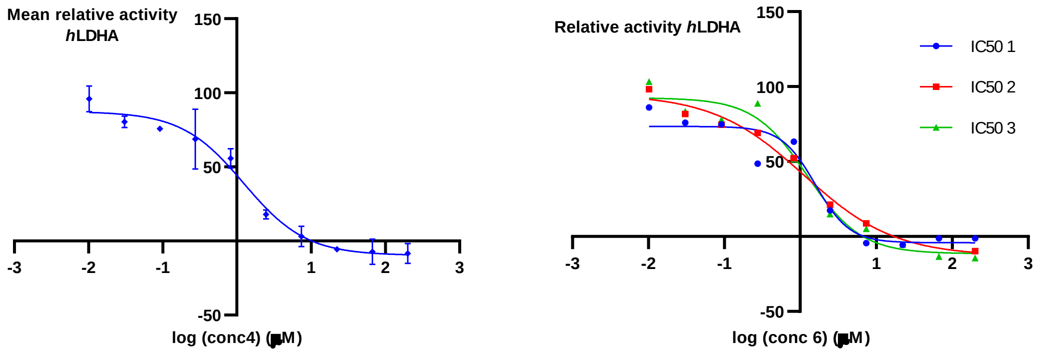

Figure S29. Left: Non-linear "log (conc. **6**) *vs* relative *h*LDHA activity" plot (media of four replicates). Error bars show standard deviation. Right: Non-linear "log (conc. **6**) *vs* relative *h*LDHA activity" plots of the four replicates (using ten concentrations of inhibitor and 180 µM pyruvate).

Table S29. Statistical parameters of the IC_{50} value for inhibition of *h*LDHA obtained for **6**, using ten inhibitor concentrations and 180 µM substrate (pyruvate) concentration (four replicates).

**IC_{50} (uM)**	0.053 ± 0.004
95% Confidence Intervals	0.0503 to 0.0571
R square	0.9925

Compound 7

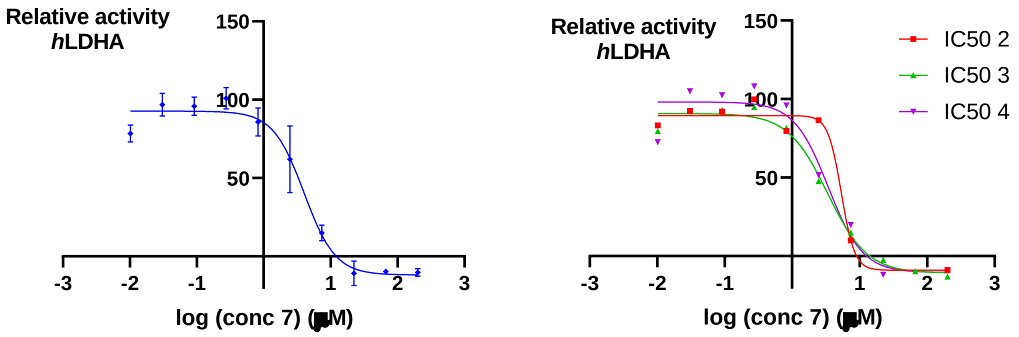

Figure S30. Left: Non-linear "log (conc. **7**) *vs* relative *h*LDHA activity" plot (media of four replicates). Error bars show standard deviation. Right: Non-linear "log (conc. **7**) *vs* relative *h*LDHA activity" plots of the four replicates (using ten concentrations of inhibitor and 180 µM pyruvate).

Table S30. Statistical parameters of the IC_{50} value for inhibition of *h*LDHA obtained for **7**, using ten inhibitor concentrations and 180 µM substrate (pyruvate) concentration (four replicates).

**IC_{50} (uM)**	4.04 ± 1.13
95% Confidence Intervals	3.036 to 5.317
R square	0.9556

Compound 9

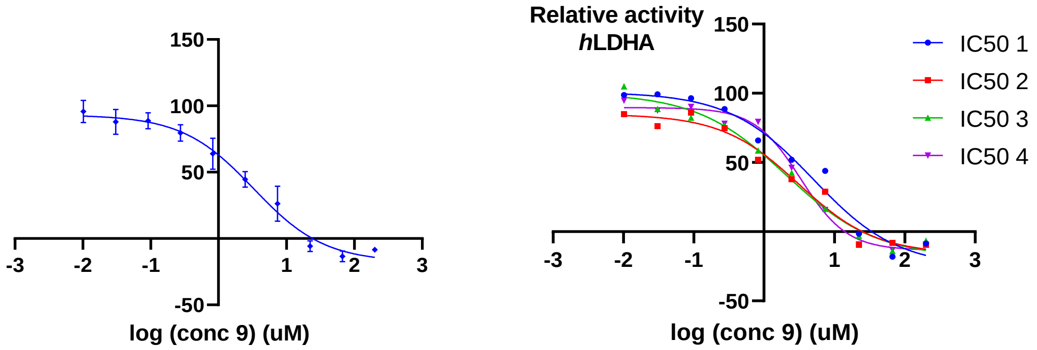

Figure S31. Left: Non-linear “log (conc. **9**) *vs* relative *h*LDHA activity” plot (media of four replicates). Error bars show standard deviation. Right: Non-linear “log (conc. **9**) *vs* relative *h*LDHA activity” plots of the four replicates (using ten concentrations of inhibitor and 180 µM pyruvate).

Table S31. Statistical parameters of the IC_{50} value for inhibition of *h*LDHA obtained for **9**, using ten inhibitor concentrations and 180 µM substrate (pyruvate) concentration (four replicates).

**IC_{50} (uM)**	3.38 ± 1.22
95% Confidence Intervals	2.235 to 4.757
R square	0.9639

Compound 11

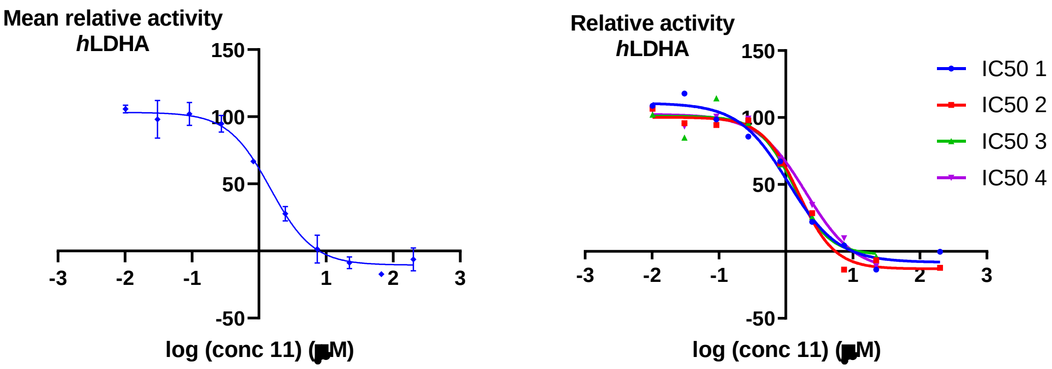

Figure S32. Left: Non-linear “log (conc. **11**) *vs* relative *h*LDHA activity” plot (media of four replicates). Error bars show standard deviation. Right: Non-linear “log (conc. **11**) *vs* relative *h*LDHA activity” plots of the four replicates (using ten concentrations of inhibitor and 180 µM pyruvate).

Table S32. Statistical parameters of the IC_{50} value for inhibition of *h*LDHA obtained for **11**, using ten inhibitor concentrations and 180 µM substrate (pyruvate) concentration (four replicates).

**IC_{50} (uM)**	1.48 ± 0.39
95% Confidence Intervals	1.211 to 1.815
R square	0.9778

Compound 12

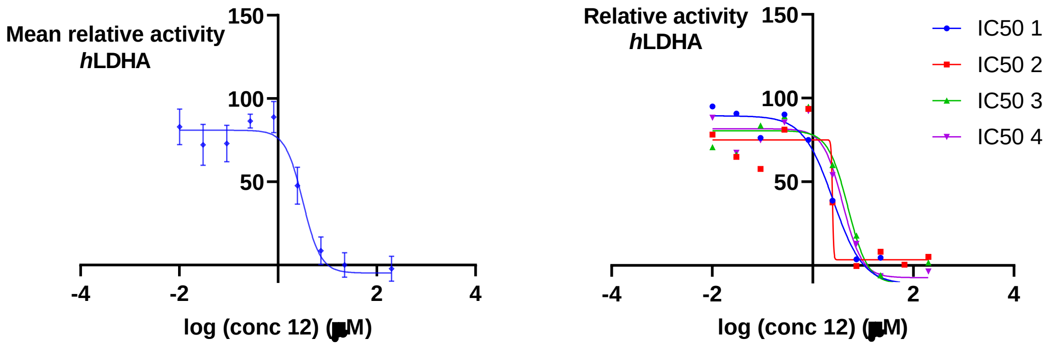

Figure S33. Left: Non-linear "log (conc. **12**) *vs* relative *h*LDHA activity" plot (media of four replicates). Error bars show standard deviation. Right: Non-linear "log (conc. **12**) *vs* relative *h*LDHA activity" plots of the four replicates (using ten concentrations of inhibitor and 180 µM pyruvate).

Table S33. Statistical parameters of the IC_{50} value for inhibition of *h*LDHA obtained for **12**, using ten inhibitor concentrations and 180 µM substrate (pyruvate) concentration (four replicates).

**IC_{50} (uM)**	3.21 ± 0.96
95% Confidence Intervals	2.431 to 4.388
R square	0.9645

Compound 13

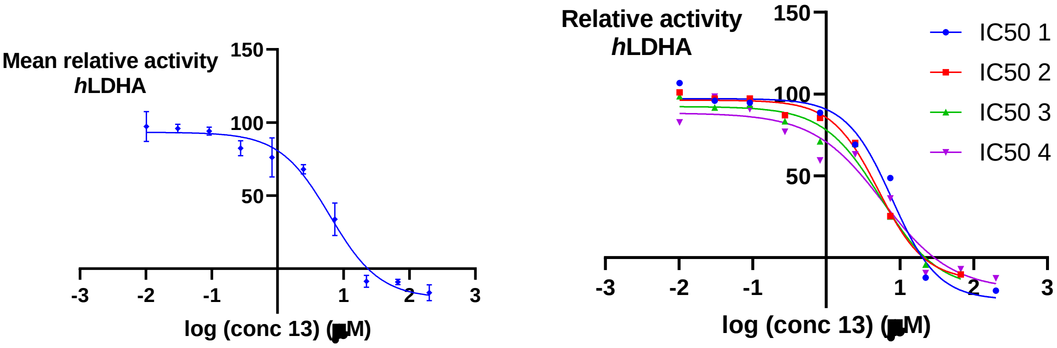

Figure S34. Left: Non-linear "log (conc. **13**) *vs* relative *h*LDHA activity" plot (media of four replicates). Error bars show standard deviation. Right: Non-linear "log (conc. **13**) *vs* relative *h*LDHA activity" plots of the four replicates (using ten concentrations of inhibitor and 180 µM pyruvate).

Table S34. Statistical parameters of the IC_{50} value for inhibition of *h*LDHA obtained for **13**, using ten inhibitor concentrations and 180 µM substrate (pyruvate) concentration (four replicates).

**IC_{50} (uM)**	6.06 ± 1.22
95% Confidence Intervals	4.485 to 8.466
R square	0.9621

Compound 14

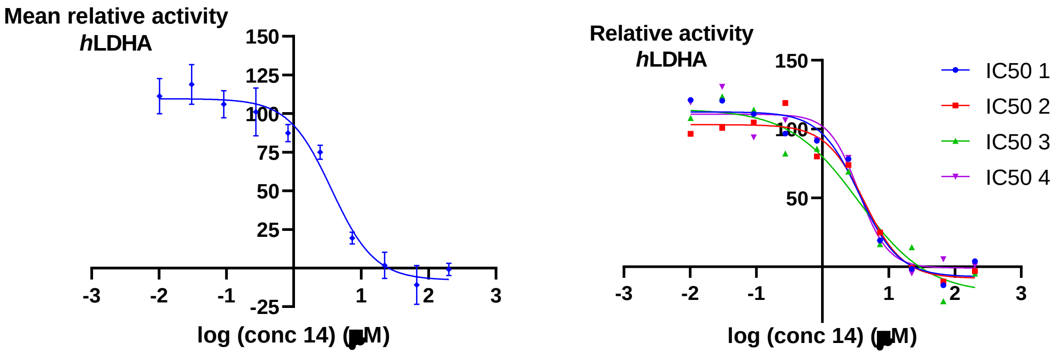

Figure S35. Left: Non-linear "log (conc. **14**) *vs* relative *h*LDHA activity" plot (media of four replicates). Error bars show standard deviation. Right: Non-linear "log (conc. **14**) *vs* relative *h*LDHA activity" plots of the four replicates (using ten concentrations of inhibitor and 180 µM pyruvate).

Table S35. Statistical parameters of the IC_{50} value for inhibition of *h*LDHA obtained for **14**, using ten inhibitor concentrations and 180 µM substrate (pyruvate) concentration (four replicates).

**IC_{50} (uM)**	3.72 ± 0.36
95% Confidence Intervals	2.763 to 4.793
R square	0.9576

Compound 15

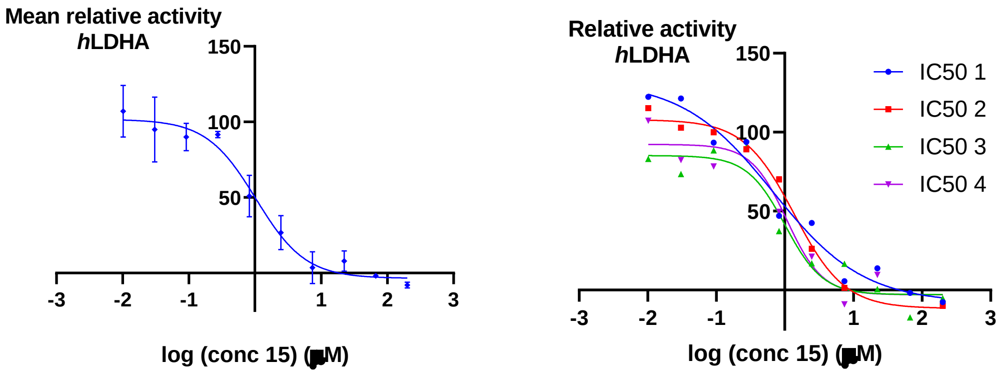

Figure S36. Left: Non-linear "log (conc. **15**) *vs* relative *h*LDHA activity" plot (media of four replicates). Error bars show standard deviation. Right: Non-linear "log (conc. **15**) *vs* relative *h*LDHA activity" plots of the four replicates (using ten concentrations of inhibitor and 180 µM pyruvate).

Table S36. Statistical parameters of the IC_{50} value for inhibition of *h*LDHA obtained for **15**, using ten inhibitor concentrations and 180 µM substrate (pyruvate) concentration (four replicates).

**IC_{50} (uM)**	1.03 ± 0.28
95% Confidence Intervals	0.6858 to 1.557
R square	0.9289

Compound 16

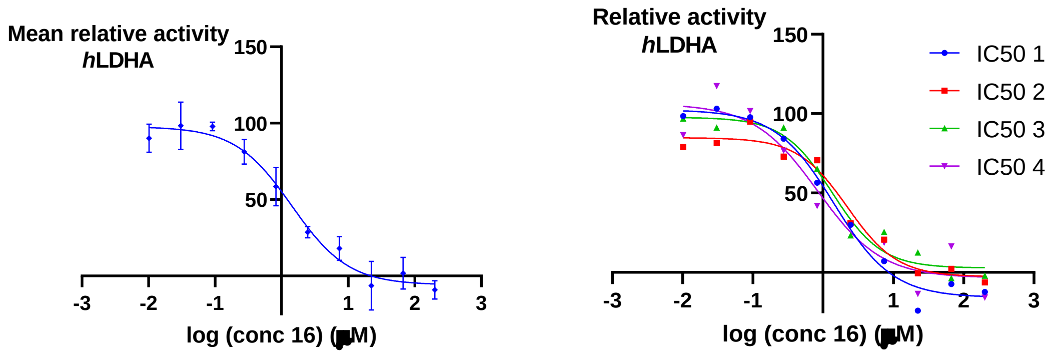

Figure S37. Left: Non-linear "log (conc. **16**) *vs* relative *h*LDHA activity" plot (media of four replicates). Error bars show standard deviation. Right: Non-linear "log (conc. **16**) *vs* relative *h*LDHA activity" plots of the four replicates (using ten concentrations of inhibitor and 180 µM pyruvate).

Table S37. Statistical parameters of the IC_{50} value for inhibition of *h*LDHA obtained for **16**, using ten inhibitor concentrations and 180 µM substrate (pyruvate) concentration (four replicates).

**IC_{50} (uM)**	1.46 ± 0.57
95% Confidence Intervals	0.9680 to 2.090
R square	0.9444

Compound 17

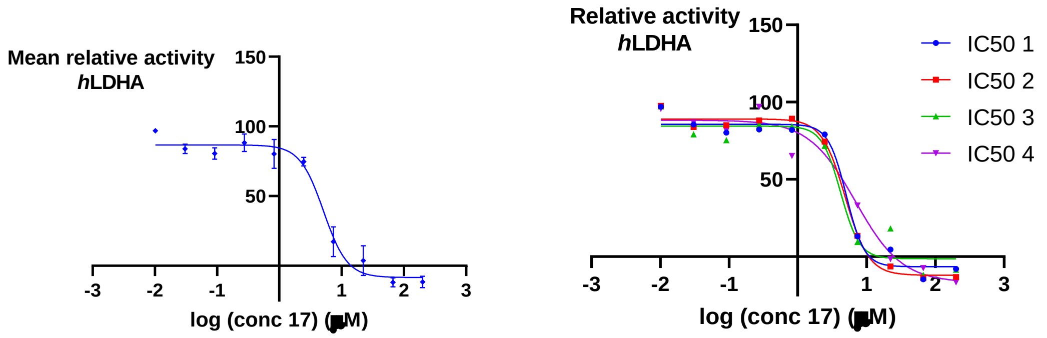

Figure S38. Left: Non-linear "log (conc. **17**) *vs* relative *h*LDHA activity" plot (media of four replicates). Error bars show standard deviation. Right: Non-linear "log (conc. **17**) *vs* relative *h*LDHA activity" plots of the four replicates (using ten concentrations of inhibitor and 180 µM pyruvate).

Table S38. Statistical parameters of the IC_{50} value for inhibition of *h*LDHA obtained for **17**, using ten inhibitor concentrations and 180 µM substrate (pyruvate) concentration (four replicates).

**IC_{50} (uM)**	4.73 ± 0.45
95% Confidence Intervals	3.906 to 5.737
R square	0.9723

Compound 18

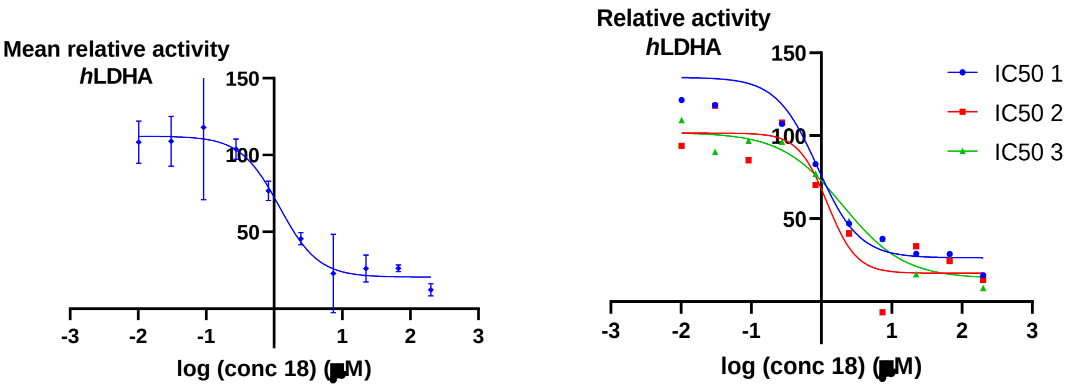

Figure S39. Left: Non-linear "log (conc. **18**) *vs* relative *h*LDHA activity" plot (media of four replicates). Error bars show standard deviation. Right: Non-linear "log (conc. **18**) *vs* relative *h*LDHA activity" plots of the four replicates (using ten concentrations of inhibitor and 180 µM pyruvate).

Table S39. Statistical parameters of the IC_{50} value for inhibition of *h*LDHA obtained for **18**, using ten inhibitor concentrations and 180 µM substrate (pyruvate) concentration (four replicates).

**IC_{50} (uM)**	1.37 ± 0.58
95% Confidence Intervals	0.6571 to 2.219
R square	0.8615

Compound 19

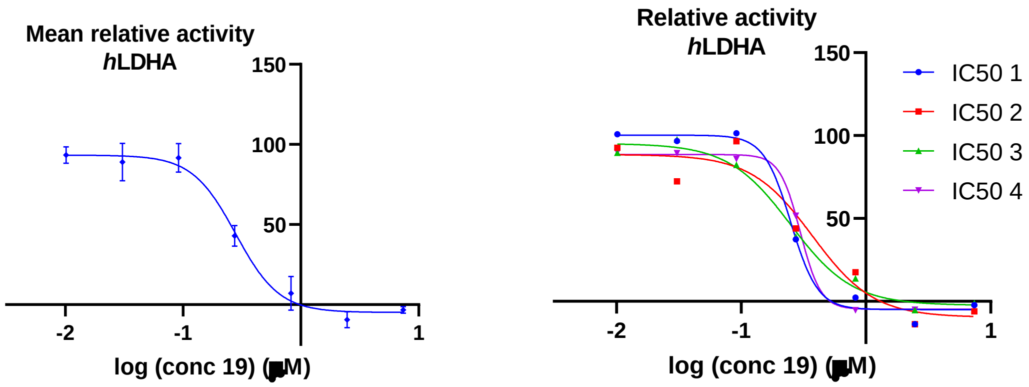

Figure S40. Left: Non-linear "log (conc. **19**) *vs* relative *h*LDHA activity" plot (media of four replicates). Error bars show standard deviation. Right: Non-linear "log (conc. **19**) *vs* relative *h*LDHA activity" plots of the four replicates (using ten concentrations of inhibitor and 180 µM pyruvate).

Table S40. Statistical parameters of the IC_{50} value for inhibition of *h*LDHA obtained for **19**, using ten inhibitor concentrations and 180 µM substrate (pyruvate) concentration (four replicates).

**IC_{50} (uM)**	0.29 ± 0.06
95% Confidence Intervals	0.2363 to 0.3397
R square	0.9693

Compound 20

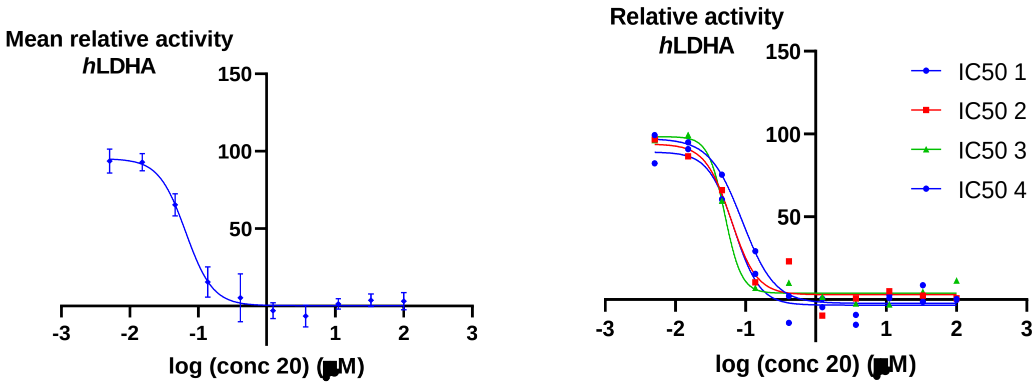

Figure S41. Left: Non-linear "log (conc. **20**) *vs* relative *h*LDHA activity" plot (media of four replicates). Error bars show standard deviation. Right: Non-linear "log (conc. **20**) *vs* relative *h*LDHA activity" plots of the four replicates (using ten concentrations of inhibitor and 180 µM pyruvate).

Table S41. Statistical parameters of the IC_{50} value for inhibition of *h*LDHA obtained for **20**, using ten inhibitor concentrations and 180 µM substrate (pyruvate) concentration (four replicates).

**IC_{50} (uM)**	0.07 ± 0.02
95% Confidence Intervals	0.05470 to 0.07868
R square	0.9633

Compound 21

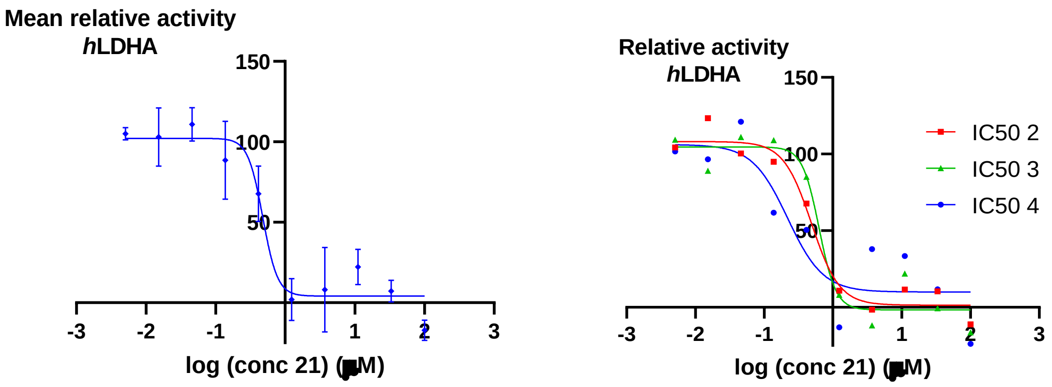

Figure S42. Left: Non-linear “log (conc. **21**) *vs* relative *h*LDHA activity” plot (media of four replicates). Error bars show standard deviation. Right: Non-linear “log (conc. **21**) *vs* relative *h*LDHA activity” plots of the four replicates (using ten concentrations of inhibitor and 180 µM pyruvate).

Table S42. Statistical parameters of the IC_{50} value for inhibition of *h*LDHA obtained for **21**, using ten inhibitor concentrations and 180 µM substrate (pyruvate) concentration (four replicates).

**IC_{50} (uM)**	0.45 ± 0.21
95% Confidence Intervals	0.3147 to 0.6859
R square	0.8888

Compound 22

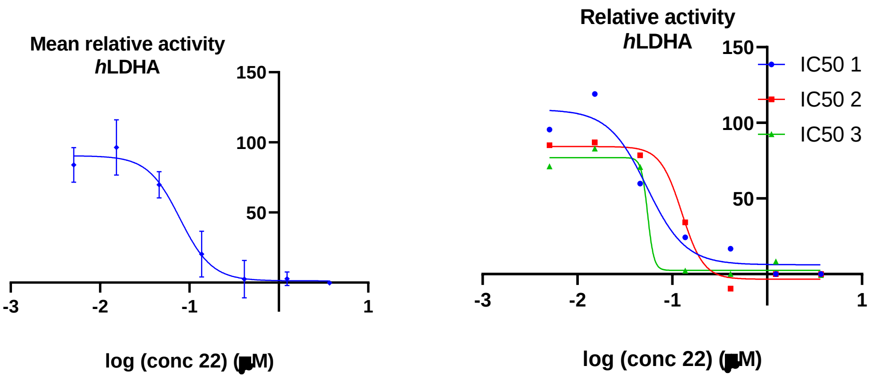

Figure S43. Left: Non-linear “log (conc. **22**) *vs* relative *h*LDHA activity” plot (media of four replicates). Error bars show standard deviation. Right: Non-linear “log (conc. **22**) *vs* relative *h*LDHA activity” plots of the four replicates (using ten concentrations of inhibitor and 180 µM pyruvate).

Table S43. Statistical parameters of the IC_{50} value for inhibition of *h*LDHA obtained for **22**, using ten inhibitor concentrations and 180 µM substrate (pyruvate) concentration (four replicates).

**IC_{50} (uM)**	0.08 ± 0.03
95% Confidence Intervals	0.06099 to 0.1034
R square	0.9471

Compound 23

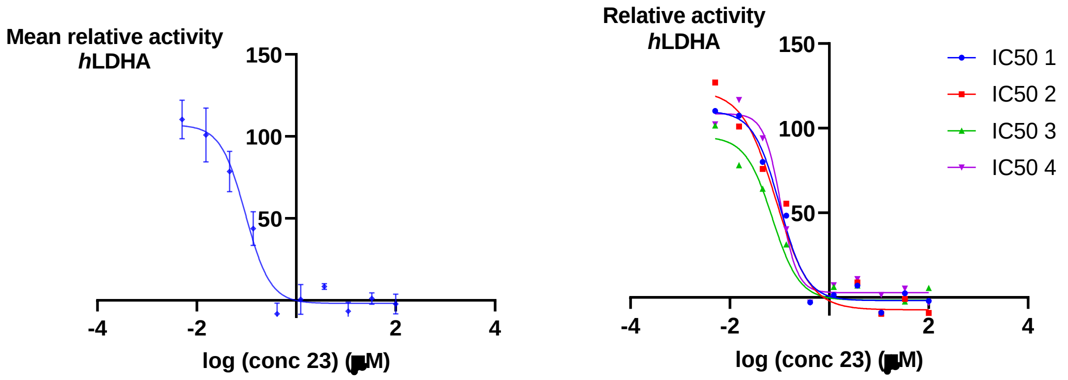

Figure S44. Left: Non-linear "log (conc. **23**) *vs* relative *h*LDHA activity" plot (media of four replicates). Error bars show standard deviation. Right: Non-linear "log (conc. **23**) *vs* relative *h*LDHA activity" plots of the four replicates (using ten concentrations of inhibitor and 180 µM pyruvate).

Table S44. Statistical parameters of the IC_{50} value for inhibition of *h*LDHA obtained for **23**, using ten inhibitor concentrations and 180 µM substrate (pyruvate) concentration (four replicates).

**IC_{50} (uM)**	0.09 ± 0.02
95% Confidence Intervals	0.07314 to 0.1242
R square	0.9465

Compound 24

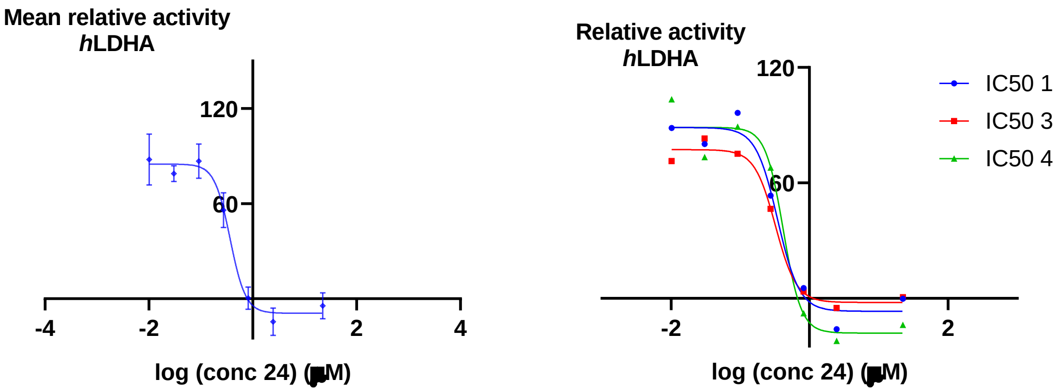

Figure S45. Left: Non-linear "log (conc. **24**) *vs* relative *h*LDHA activity" plot (media of four replicates). Error bars show standard deviation. Right: Non-linear "log (conc. **24**) *vs* relative *h*LDHA activity" plots of the four replicates (using ten concentrations of inhibitor and 180 µM pyruvate).

Table S45. Statistical parameters of the IC_{50} value for inhibition of *h*LDHA obtained for **24**, using ten inhibitor concentrations and 180 µM substrate (pyruvate) concentration (four replicates).

**IC_{50} (uM)**	0.36 ± 0.05
95% Confidence Intervals	0.2805 to 0.4788
R square	0.9557

Compound 25

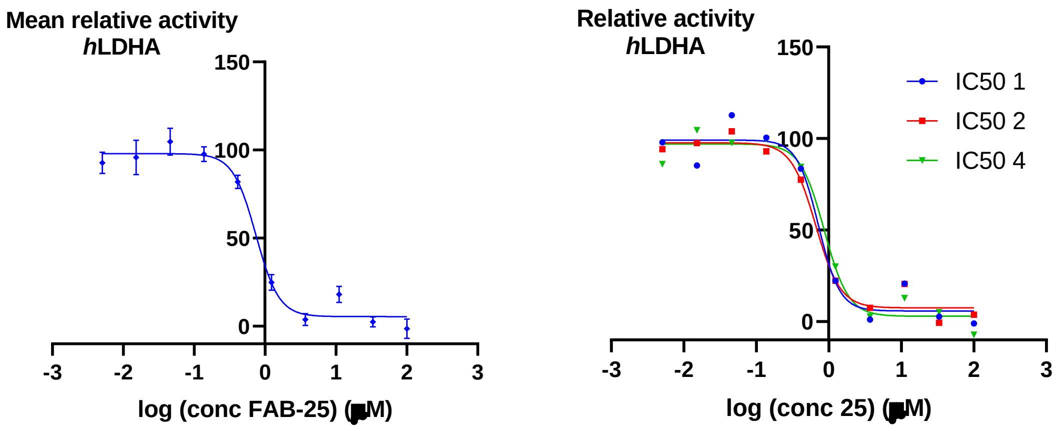

Figure S46. Left: Non-linear "log (conc. **25**) *vs* relative *h*LDHA activity" plot (media of four replicates). Error bars show standard deviation. Right: Non-linear "log (conc. **25**) *vs* relative *h*LDHA activity" plots of the four replicates (using ten concentrations of inhibitor and 180 µM pyruvate).

Table S46. Statistical parameters of the IC_{50} value for inhibition of *h*LDHA obtained for **25**, using ten inhibitor concentrations and 180 µM substrate (pyruvate) concentration (four replicates).

**IC_{50} (uM)**	0.75 ± 0.11
95% Confidence Intervals	0.6125 to 0.9033
R square	0.9732

Compound 26

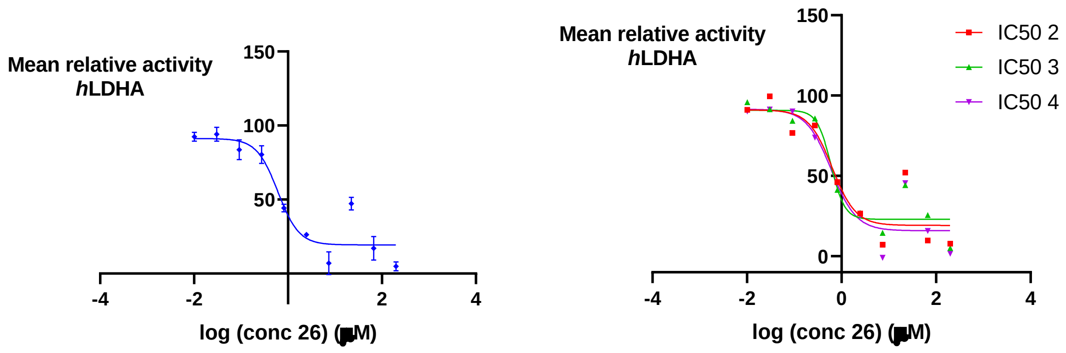

Figure S47. Left: Non-linear "log (conc. **26**) *vs* relative *h*LDHA activity" plot (media of four replicates). Error bars show standard deviation. Right: Non-linear "log (conc. **26**) *vs* relative *h*LDHA activity" plots of the four replicates (using ten concentrations of inhibitor and 180 µM pyruvate).

Table S47. Statistical parameters of the IC_{50} value for inhibition of *h*LDHA obtained for **26**, using ten inhibitor concentrations and 180 µM substrate (pyruvate) concentration (four replicates).

**IC_{50} (uM)**	0.61 ± 0.02
95% Confidence Intervals	0.3582 to 0.9676
R square	0.8795

Compound 27

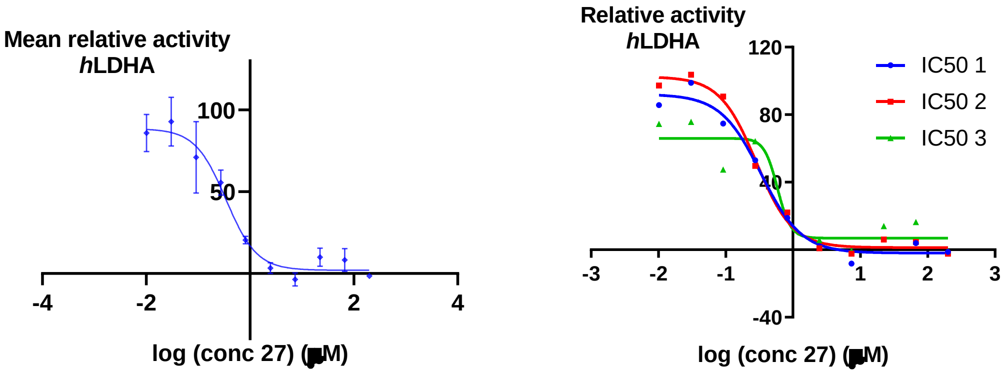

Figure S48. Left: Non-linear "log (conc. **27**) *vs* relative *h*LDHA activity" plot (media of four replicates). Error bars show standard deviation. Right: Non-linear "log (conc. **27**) *vs* relative *h*LDHA activity" plots of the four replicates (using ten concentrations of inhibitor and 180 µM pyruvate).

Table S48. Statistical parameters of the IC_{50} value for inhibition of *h*LDHA obtained for **27**, using ten inhibitor concentrations and 180 µM substrate (pyruvate) concentration (four replicates).

**IC_{50} (uM)**	0.40 ± 0.16
95% Confidence Intervals	0.2238 to 0.5296
R square	0.9308

Compound 28

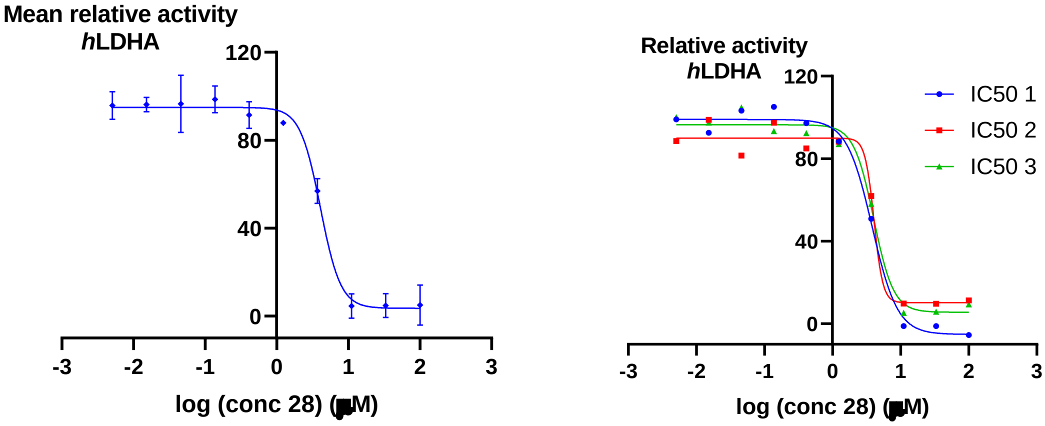

Figure S49. Left: Non-linear "log (conc. **28**) *vs* relative *h*LDHA activity" plot (media of four replicates). Error bars show standard deviation. Right: Non-linear "log (conc. **28**) *vs* relative *h*LDHA activity" plots of the four replicates (using ten concentrations of inhibitor and 180 µM pyruvate).

Table S49. Statistical parameters of the IC_{50} value for inhibition of *h*LDHA obtained for **28**, using ten inhibitor concentrations and 180 µM substrate (pyruvate) concentration (four replicates).

**IC_{50} (uM)**	3.97 ± 0.13
95% Confidence Intervals	3.579 to 4.703
R square	0.9770

Compound 29

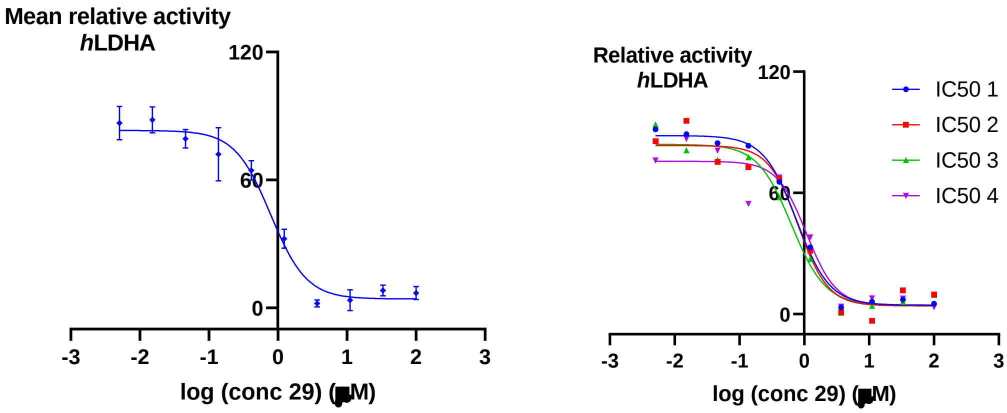

Figure S50. Left: Non-linear "log (conc. **29**) *vs* relative *h*LDHA activity" plot (media of four replicates). Error bars show standard deviation. Right: Non-linear "log (conc. **29**) *vs* relative *h*LDHA activity" plots of the four replicates (using ten concentrations of inhibitor and 180 µM pyruvate).

Table S50. Statistical parameters of the IC_{50} value for inhibition of *h*LDHA obtained for **29**, using ten inhibitor concentrations and 180 µM substrate (pyruvate) concentration (four replicates).

**IC_{50} (uM)**	0.82 ± 0.19
95% Confidence Intervals	0.6288 to 0.9827
R square	0.9639

Compound S2

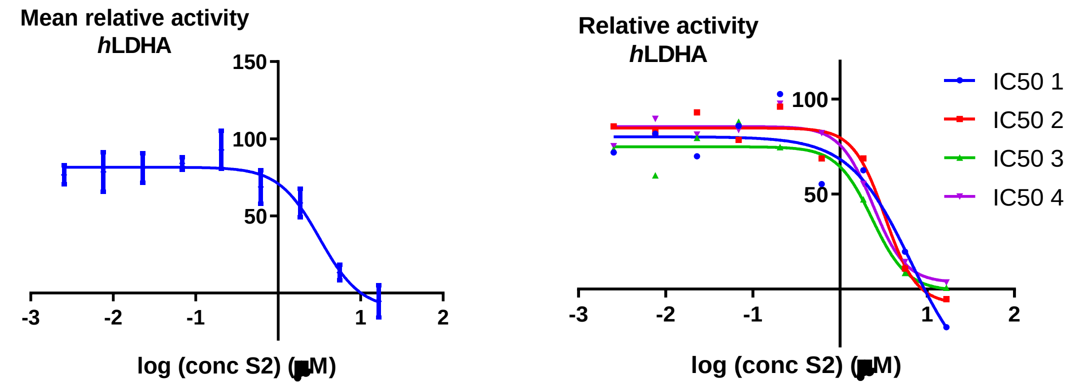

Figure S51. Left: Non-linear "log (conc. **S2**) *vs* relative *h*LDHA activity" plot (media of four replicates). Error bars show standard deviation. Right: Non-linear "log (conc. **S2**) *vs* relative *h*LDHA activity" plots of the four replicates (using ten concentrations of inhibitor and 180 µM pyruvate).

Table S51. Statistical parameters of the IC_{50} value for inhibition of *h*LDHA obtained for **S2**, using ten inhibitor concentrations and 180 µM substrate (pyruvate) concentration (four replicates).

**IC_{50} (uM)**	3.62 ± 1.98
95% Confidence Intervals	2.156 to 4.696
R square	0.9122

Compound S6

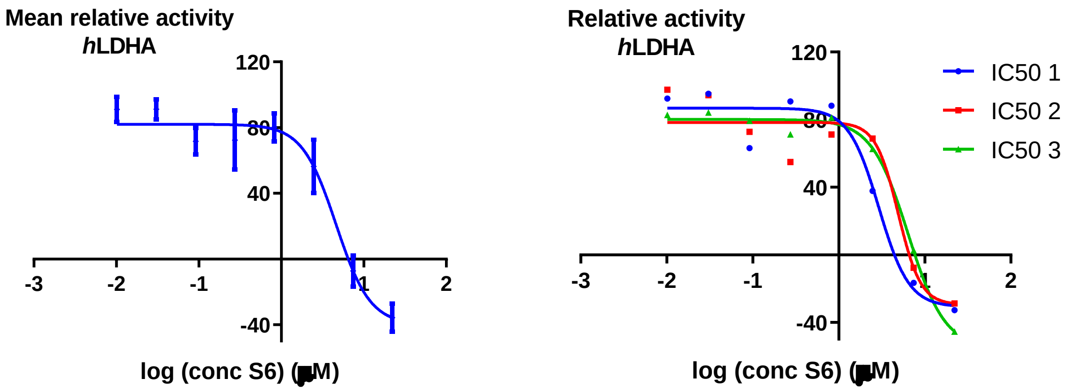

Figure S52. Left: Non-linear "log (conc. **S6**) *vs* relative *h*LDHA activity" plot (media of four replicates). Error bars show standard deviation. Right: Non-linear "log (conc. **S6**) *vs* relative *h*LDHA activity" plots of the four replicates (using ten concentrations of inhibitor and 180 µM pyruvate).

Table S52. Statistical parameters of the IC_{50} value for inhibition of *h*LDHA obtained for **S6**, using ten inhibitor concentrations and 180 µM substrate (pyruvate) concentration (four replicates).

**IC_{50} (uM)**	4.75 ± 1.74
95% Confidence Intervals	3.299 to 6.461
R square	0.9382

S5. Evaluation of dual inhibitors on hepatocytes of PH1 mice (*in vitro*)

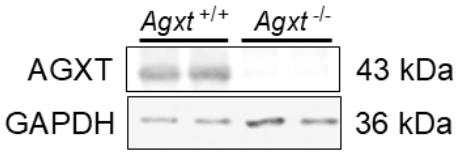

Figure S53. AGXT protein abundance in livers of *Agxt* $^{+/+}$ and *Agxt* $^{-/-}$ mice. Livers from mice *Agxt* $^{+/+}$ (n=2) and *Agxt* $^{-/-}$ (n=2) mice were isolated and AGXT protein expression was assessed.

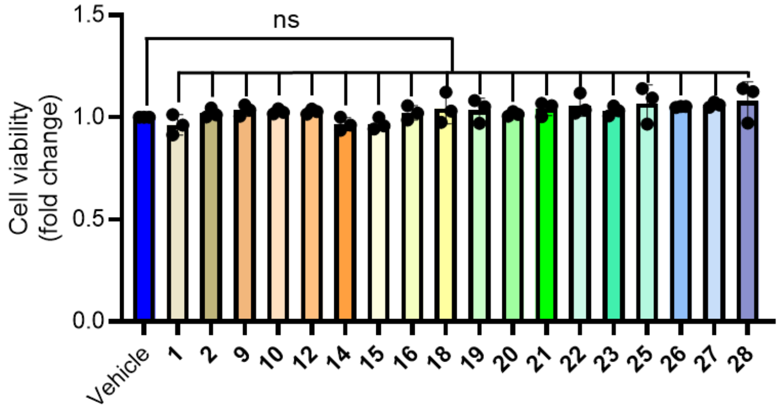

Figure S54. Percent cell viability (n = 3) in the evaluation of dual inhibitors on hepatocytes of PH1 mice. *Agxt1*$^{-/-}$ primary hepatocytes were isolated, stimulated with GA (5 mM) and treated with either vehicle (DMSO) or inhibitors (50µM) for 24 h.

		Compound	Fold change extracellular oxalate	
			10 µM	50 µM
		1	*	*
OPPFSAs	R = Polar functions	9	****	****
		10	****	****
	R = X	12	****	****
	R = Alkyl chains	14	***	***
		15	****	****
		16	****	****
		18	**	**
		19	**	**
		20	**	*
		21	****	****
		22	****	****
		23	***	***
		26	****	****
		2	****	****

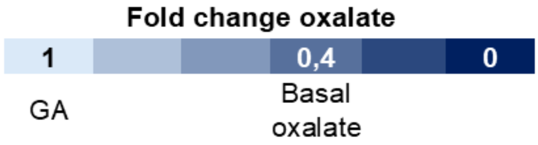

Figure S55. Heatmap of extracellular oxalate levels (fold change) in primary hepatocytes from *Agxt*$^{-/-}$ mice following treatment with OPPFSAs. *Agxt*$^{-/-}$ primary hepatocytes were isolated, incubated in the presence or absence of glycolic acid (GA, 5 mM) and treated with either vehicle (DMSO) or inhibitors at 10 µM and 50 µM for 24 h (n = 4). Statistical analysis: comparisons between GA control and inhibitor-treated groups were performed using one-way ANOVA followed by Tukey's post hoc test. Significance levels (*p < 0.05; **p < 0.01; ***p < 0.001, ****p < 0.0001).

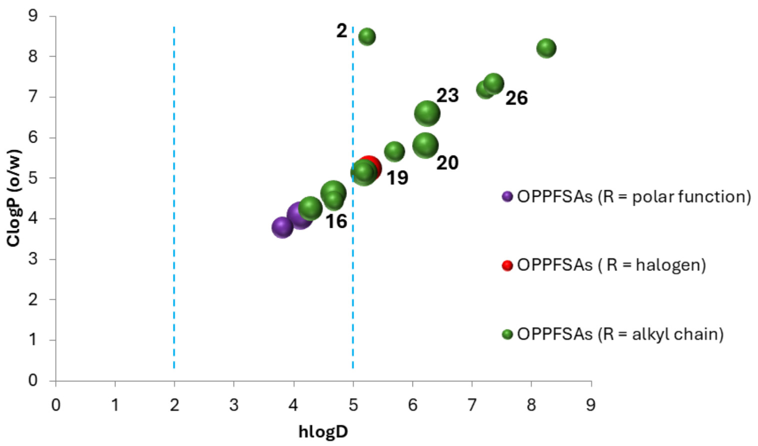

Figure S56. Bubble graphs representing ClogP (o/w) and calculated hlogD of OPPFSAs and reference **1**. Graph: Bubble size indicates fold change intracellular oxalate (small bubble indicates low intracellular oxalate after treatment 10 µM concentrations of the compound). The dashed blue lines represent the hypothesized range of lipophilicity at physiological pH (hlogD) for a compound to ensure an acceptable permeability/solubility balance.[2]

S6. Inhibition kinetics on *h*GO

Compound 20

For compound **20**, one experiment for K_i and inhibition mechanism was carried out following the protocol described. In the experiment, four different inhibitor concentrations and ten different substrate concentrations were used. The K_i value was obtained from the mean of three v_o determinations for each substrate and inhibitor concentration. The following Table summarizes the data obtained in the experiment.

Table S53. Mean values of v_o (Δ fluorescence/min) obtained at each substrate (glycolate) and inhibitor (**20**) concentration for enzyme *h*GO.

Glycolate (mM)	Concentration of 20 (µM)			
	0	0.25	0.5	2
10	2164.7294	1919.94236	1817.14315	864.120431
3.33333333	1656.40605	1420.42532	1416.96308	459.388849
1.11111111	1489.9259	1205.03726	1113.74649	360.096966
0.37037037	1245.38444	1012.5927	907.509476	306.842395
0.12345679	998.154855	763.349062	763.154966	130.577645
0.04115226	755.03316	641.160883	487.835853	119.148448
0.01371742	399.82943	362.659393	195.999467	66.6300625
0.00457247	151.107824	154.727014	56.8323993	24.3280259
0.00152416	51.1484431	51.4032545	23.7851497	8.68395411
0.00050805	14.2706778	11.2710792	1.50056797	-3.38428857

The initial velocity (v_o) was determined as the slope calculated in the linear interval of the “product vs time” graph representing the progression of the enzymatic reaction at each substrate concentration. This linear interval was of 4 min after a total measuring time of 15 min. The represented values of v_o are the mean of three determinations.

Table S54. Values of V_{max} and K_M for *h*GO obtained at each tested concentration of **20** using the data in Table S53.

	**Concentration of 20 (µM)**			
	**0**	**0.25**	**0.5**	**2**
V_{max} (Δ fluorescence/min)	1792	1557	1549	870.5
K_M (µM)	84.7	108.9	165.6	1387
**Std. Error**				
V_{max}	118.9	131.3	116.6	129.9
K_M	28.6	45.4	58.5	659.8

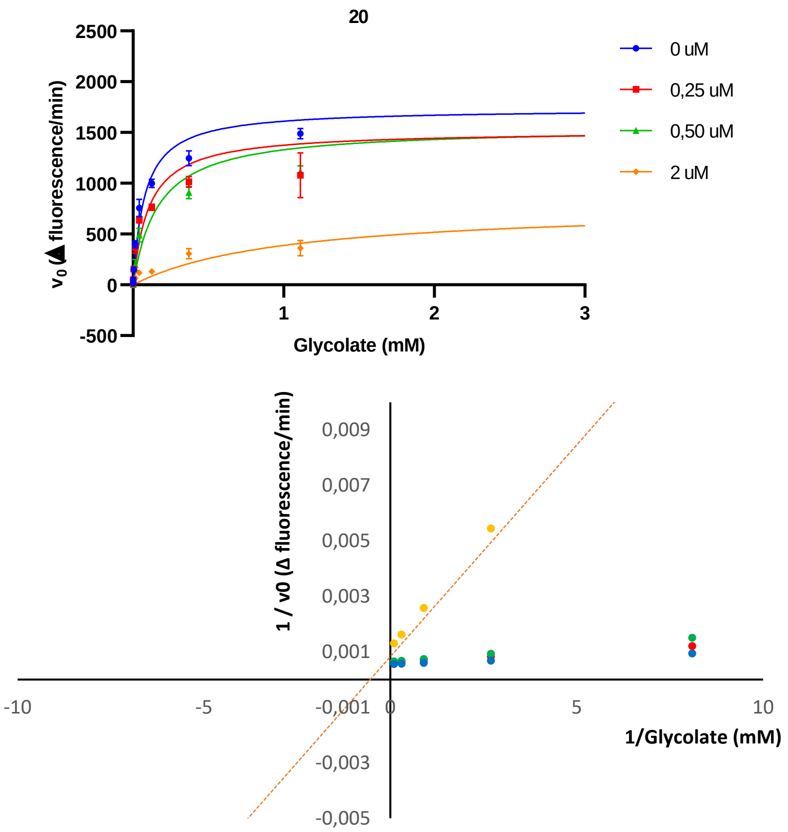

Figure S57. Non-linear concentration *vs* initial velocity (above) and Lineweaver-Burk (below) plots for compound **20** on enzyme *h*GO (using four concentrations of inhibitor and ten different concentrations of glycolate; measurements every minute during 4 min) (built from data in Table S53). Colour code indicates concentration of **20**: 2 µM (orange); 0.50 µM (green); 0.25 µM (red); 0 µM (blue).

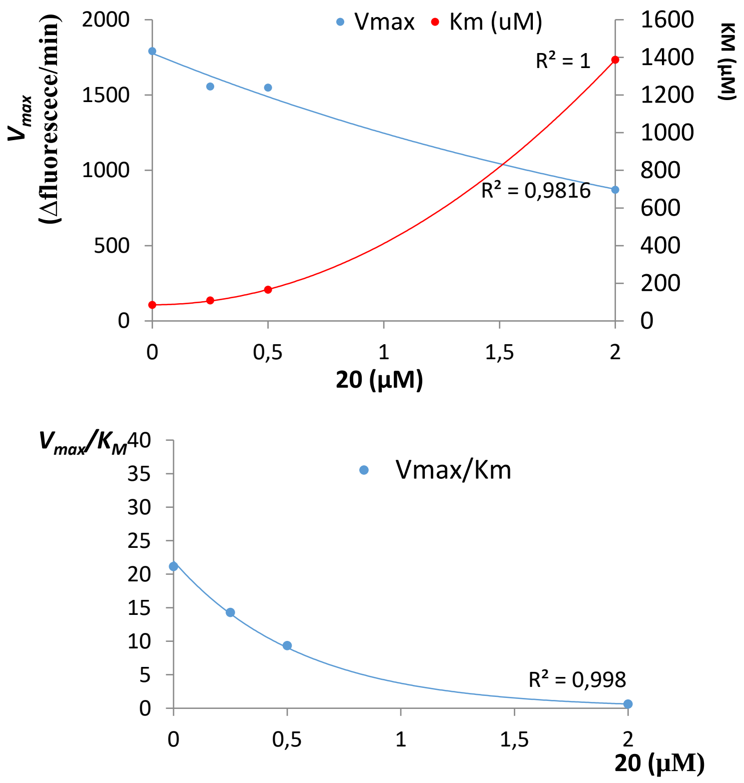

Figure S58. Observed variation on the parameters V_{max} (Δ fluorescence/min) and K_M (μM) and the ratio V_{max}/K_M with increasing concentrations of the inhibitor **20**, showing non-competitive mixed type inhibition (α>1) (built from data in Table S54). Plot above: Curvilinear decrease of V_{max}. and curvilinear increase of K_M. Plot below: Curvilinear decrease of the ration V_{max}/K_M .

Table S55. Parameters of noncompetitive mixed-model inhibition obtained for the compound **20** on *h*GO.

	**MIXED MODEL INHIBITION**
**K_i (uM)**	0.27
**α**	5.82
Std. Error K_i	0.08
Std. Error α	2.17
95% Confidence Intervals K_i	0.1113 to 0.4188
95% Confidence Intervals α	1.429 to 10.21
R square	0.9812

Compound 23

For compound **23**, two experiments for K_i and inhibition mechanism were carried out following the protocol described. In the experiment, three different inhibitor concentrations and ten different substrate concentrations were used. The K_i value was obtained from the mean of three v_o determinations for each substrate and inhibitor concentration. The following Table summarizes the data obtained in one of the experiments.

Table S56. Mean values of v_o (Δ fluorescence/min) obtained at each substrate (glycolate) and inhibitor (**23**) concentration for enzyme *h*GO.

Glycolate (mM)	Concentration of 23 (μM)				
	0	0.75	1	1.5	2
10	1496.117331	688.554534	653.859276	361.422372	172.252473
3.33333333	1420.957653	555.58393	485.089681	268.515864	131.18718
1.11111111	1514.384753	606.688496	489.889009	282.878929	148.273571
0.37037037	1426.123941	505.391791	449.073497	156.257975	123.525717
0.12345679	1243.293378	486.928567	339.65283	184.033038	73.3396172
0.04115226	858.5968149	338.791041	300.899257	154.693331	78.6040755
0.01371742	498.5206865	216.151547	131.800359	77.583847	14.911853
0.00457247	189.4716756	71.615339	61.73166	28.1288362	-6.00224917
0.00152416	70.62899914	16.5706332	28.5438869	-6.38496684	-15.7725093
0.00050805	18.55127878	-4.53375652	-16.8572882	-14.0163981	-21.679057

The initial velocity (v_o) was determined as the slope calculated in the linear interval of the "product vs time" graph representing the progression of the enzymatic reaction at each substrate concentration. This linear interval was of 4 min after a total measuring time of 15 min. The represented values of v_o are the mean of three determinations.

Table S57. Values of V_{max} and K_M for *h*GO obtained at each tested concentration of **23** using the data in Table S56.

	Concentration of 23 (μM)				
	0	0.75	1	1.5	2
V_{max} (Δ fluorescence/min)	1506	609.5	540.8	289.3	154.6
K_M (mM)	0.02884	0.03151	0.04684	0.06061	0.08687
**Std. Error**					
V_{max}	18.73	22.13	30.63	27.03	12.02
K_M	0.002042	0.006460	0.01441	0.02995	0.03433

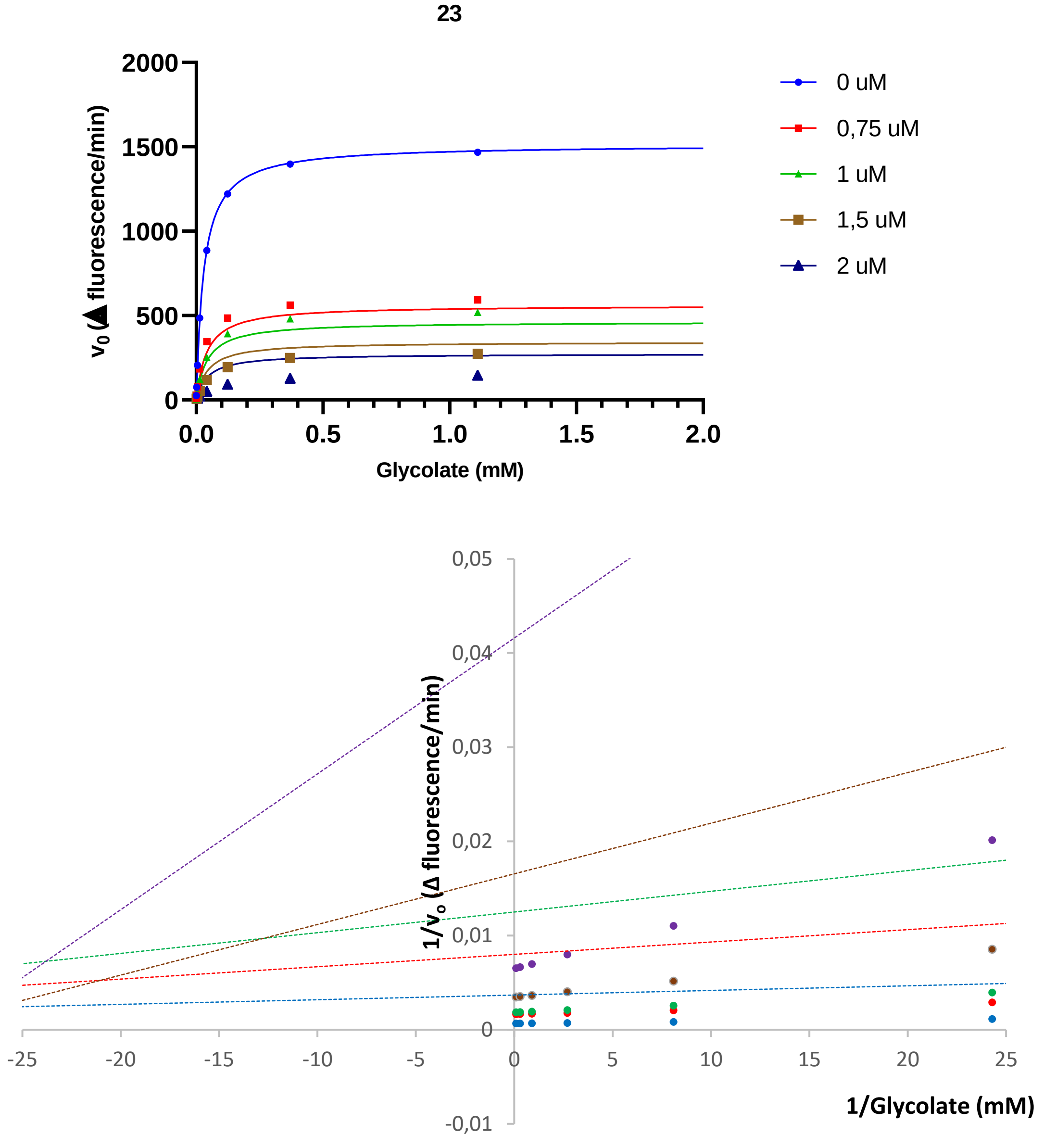

Figure S59. Non-linear concentration *vs* initial velocity (above) and Lineweaver-Burk (below) plots for compound **23** on enzyme *h*GO (using five concentrations of inhibitor and ten different concentrations of glycolate; measurements every minute during 4 min) (built from data in Table S56). Colour code indicates concentration of **23**: 2 µM (purple), 1.5 µM (brown), 1 µM (green), 0.75 µM (red); 0 µM (blue).

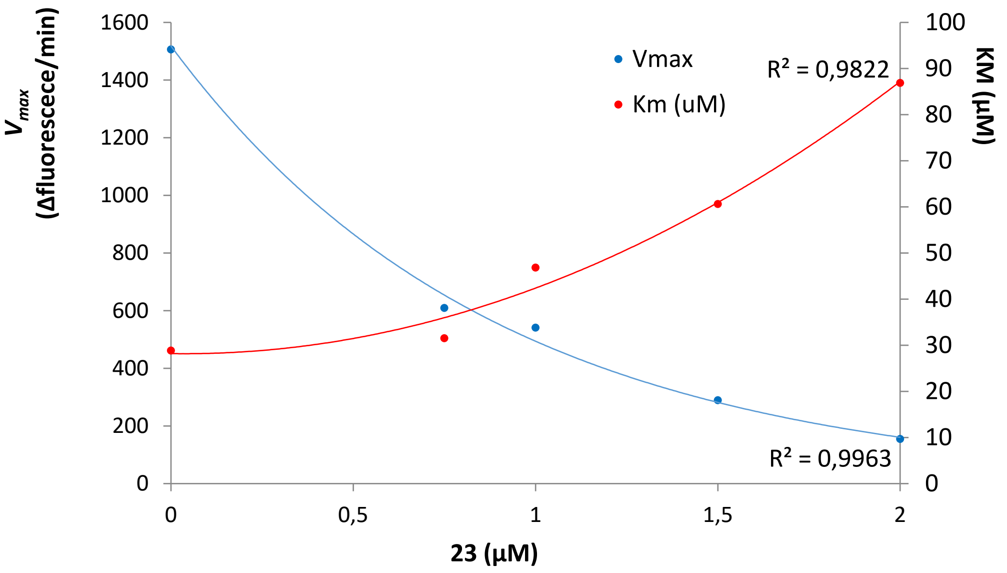

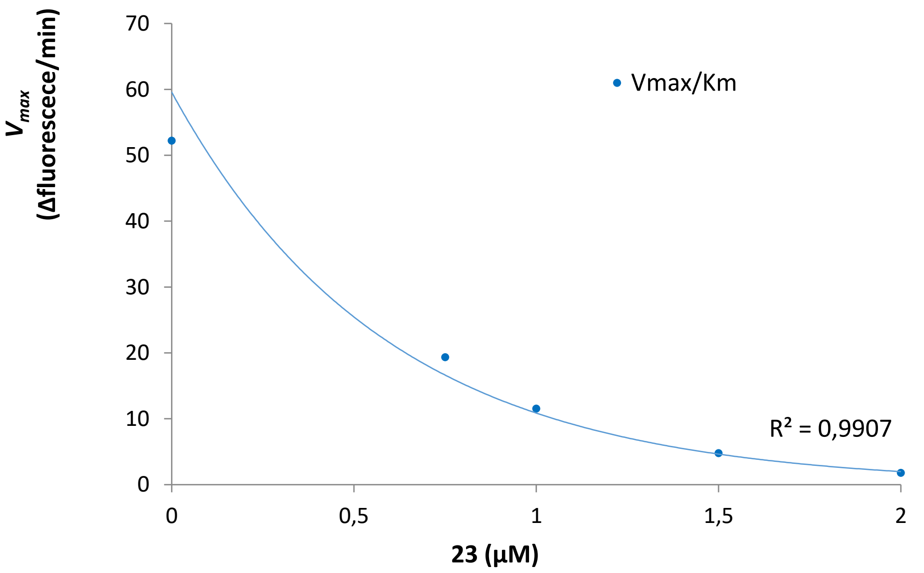

Figure S60. Observed variation on the parameters V_{max} (Δ fluorescence/min) and K_M (µM) and the ratio V_{max}/K_M with increasing concentrations of the inhibitor **23**, showing non-competitive mixed type inhibition (α>1) (built from data in Table S57). Plot above: Curvilinear decrease of V_{max} and curvilinear increase of K_M. Plot below: Curvilinear decrease of the ration V_{max}/K_M .

Table S58. Parameters of noncompetitive mixed-model inhibition obtained for the compound **23** on *h*GO.

	**MIXED MODEL INHIBITION**
**K_i (uM)**	0.268
**α**	1.65
95% Confidence Intervals K_i	0.1238 to 0.4122
95% Confidence Intervals α	0.6657 to 2.625
R square	0.9834

Compound 2

For compound **2**, one experiment for K_i and inhibition mechanism was carried out following the protocol described. In the experiment, four different inhibitor concentrations and ten different substrate concentrations were used. The K_i value was obtained from the mean of three v_o determinations for each substrate and inhibitor concentration. The following Table summarizes the data obtained in the experiment.

Table S59. Mean values of v_o (Δ fluorescence/min) obtained at each substrate (glycolate) and inhibitor (**2**) concentration for enzyme *h*GO.

**Glycolate (mM)**	**Concentration of 2 (µM)**			
	**0**	**0.5**	**0.8**	**1.6**
10	3177.3690000	2493.2050000	1832.1120000	1083.50000000
3.33333333	2458.6080000	1784.4160000	1236.3680000	607.52940000
1.11111111	2295.6810000	1664.9160000	948.4041000	520.13470000
0.37037037	2005.1900000	1314.5040000	791.3109000	410.05080000
0.12345679	1459.6360000	1045.0810000	499.9174000	236.43880000
0.04115226	687.4374000	749.4102000	359.8972000	171.03800000
0.01371742	231.0837000	420.8221000	155.7407000	50.92751000
0.00457247	89.4261700	159.8960000	46.5835300	17.94385000
0.00152416	26.9780900	42.3667500	4.4062430	-3.28873900
0.00050805	30.9364500	6.6413980	8.8441490	-1.91529000

The initial velocity (v_o) was determined as the slope calculated in the linear interval of the "product vs time" graph representing the progression of the enzymatic reaction at each substrate concentration. This linear interval was of 4 min after a total measuring time of 15 min. The represented values of v_o are the mean of three determinations.

Table S60. Values of V_{max} and K_M for *h*GO obtained at each tested concentration of **2** using the data in Table S59.

	**Concentration of 2 (µM)**			
	**0**	**0.5**	**0.8**	**1.6**
V_{max} (Δ fluorescence/min)	2814	2045	1618	977.0
K_M (mM)	0.1363	0.1158	0.4239	0.7154
**95% Confidence Intervals**				
V_{max}	2519 to 3108	1688 to 2403	1258 to 1978	698.6 to 1255
K_M	0.06765 to 0.2049	0.01637 to 0.2152	0.04017 to 0.8076	0.0 to 1.468

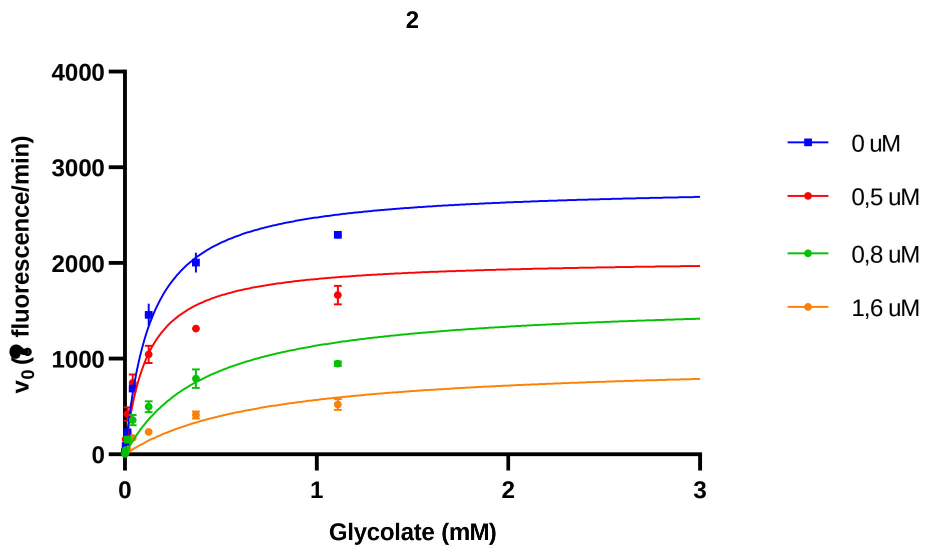

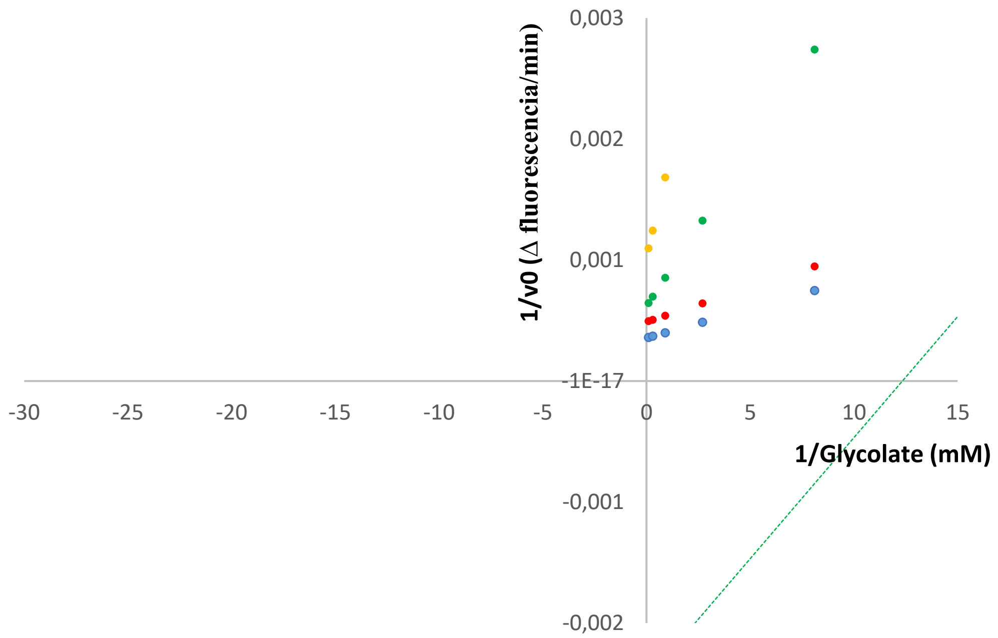

Figure S61. Non-linear concentration *vs* initial velocity (above) and Lineweaver-Burk (below) plots for compound **2** on enzyme *h*GO (using four concentrations of inhibitor and ten different concentrations of glycolate; measurements every minute during 4 min) (built from data in Table S59). Colour code indicates concentration of **2**: 1.6 µM (orange), 0.8 µM (green); 0.5 µM (red); 0 µM (blue).

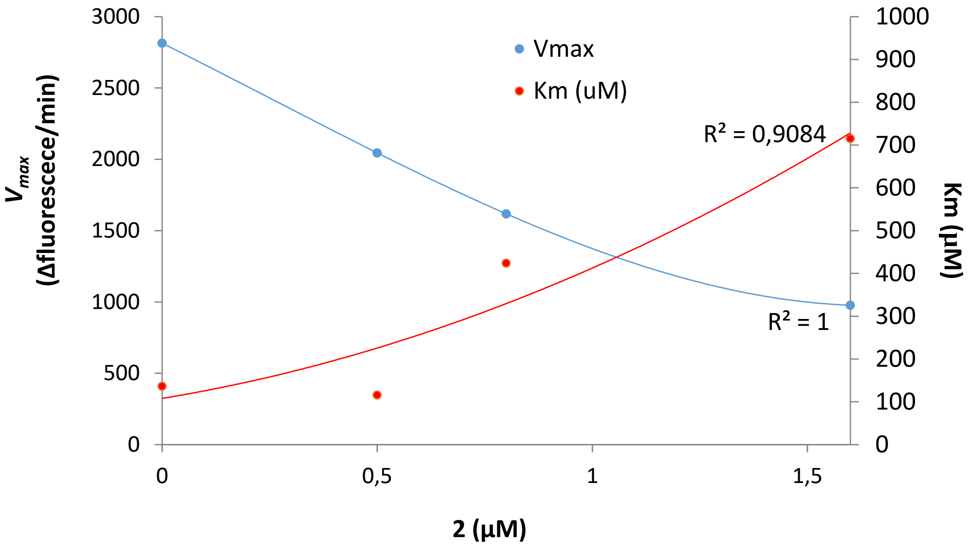

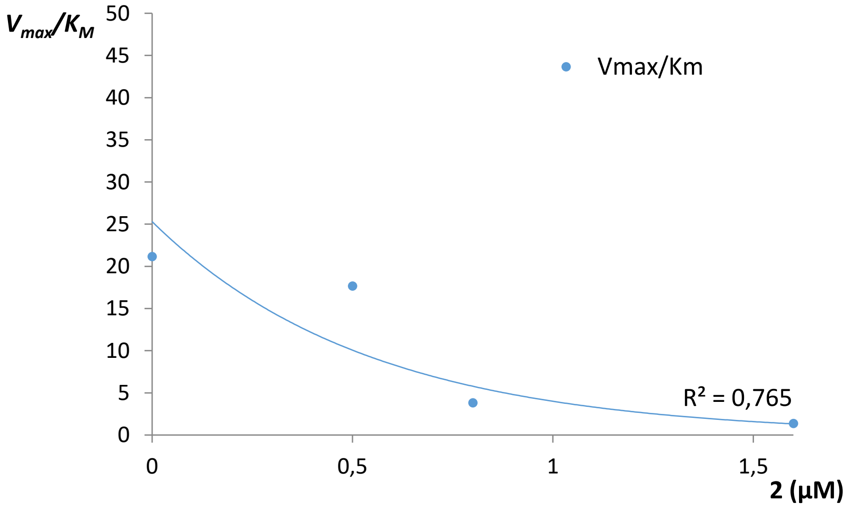

Figure S62. Observed variation on the parameters V_{max} (Δ fluorescence/min) and K_M (µM) and the ratio V_{max}/K_M with increasing concentrations of the inhibitor **2**, showing non-competitive mixed type inhibition (α>1) (built from data in Table S60). Plot above: Curvilinear decrease of V_{max} and curvilinear increase of K_M. Plot below: Curvilinear decrease of the ration V_{max}/K_M .

Table S61. Parameters of noncompetitive mixed-model inhibition obtained for the compound **2** on *h*GO.

	**MIXED MODEL INHIBITION**
**K_i (uM)**	0.39
**α**	2.38
95% Confidence Intervals K_i	0.1623 to 0.6173
95% Confidence Intervals α	0.7018 to 4.063
R square	0.9340

Evaluation of potential titration effects on enzyme *h*GO kinetic assays

Morrison K_i values (**Table S62** and **Figure S63**) for compounds **2**, **20** and **23** on enzyme *h*GO, resulted in good agreement with the classical K_i values. Morrison $K_i/[E]_T$ ratios (Table S62) confirmed that the experiments were conducted near the boundary between Michaelis-Menten

kinetics (K_i/[E]T > 10)and tight-binding regime (0.01 < K_i/[E]T < 10), but outside the titration zone (K_i/[E]T < 0.01).

Table S62. Comparison of classical and Morrison K_i values obtained for compounds **2**, **20** and **23** on enzyme *h*GO.

Compound	*h*GO K_i (nM) (CI)		$K_{i\text{-Morrison}}$ /[E]$_T$
	Classical	Morrison	
**2**[a]	390 (160-620)<br>α = 2.4	313 (253-389)	12.5
**20**[a]	270 (110-420)<br>α = 5.8	234 (177-310)	9.4
**23**[b]	268 (124-412)<br>α = 1.6	242 (179-330)	9.7

CI: 95% confidence interval. [a]For the calculation of Morrison K_i, the following values have been considered: substrate concentration, 180 µM; K_M, 99 µM; concentration of catalytic sites, 25 nM. [b]For the calculation of Morrison K_i, the following values have been considered: substrate concentration, 180 µM; K_M, 22 µM; concentration of catalytic sites, 25 nM (differences in K_M respond to different enzyme batches of recombinant enzyme).

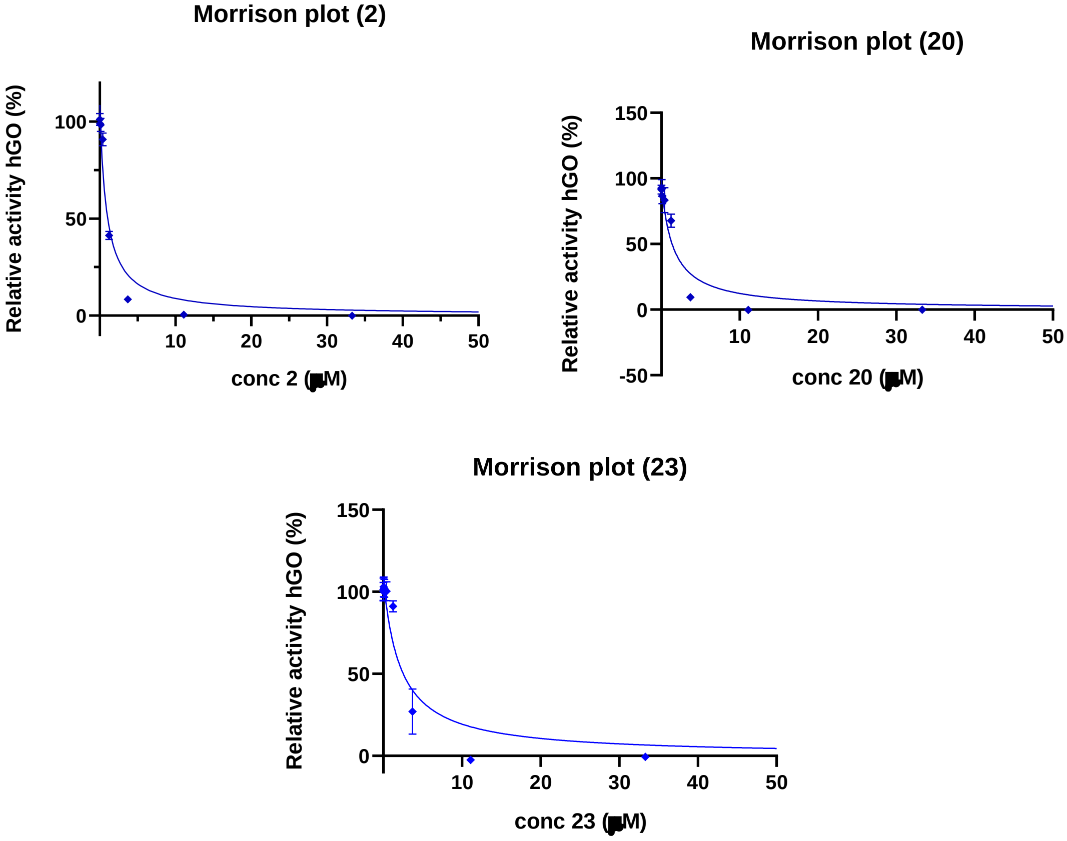

Figure S63. Morrison plots obtained for compounds **2**, **20** and **23**.

S7. Inhibition kinetics on *h*LDHA

Compound 20

For compound **20**, two independent experiments for K_i and inhibition mechanism was carried out following the protocol described. In each experiment we used four/five different inhibitor concentrations and ten different substrate concentrations. Each experiment raised one K_i value obtained from the mean of three v_o determinations for each substrate and inhibitor concentration. The final K_i value indicated in the manuscript is a mean of the two K_i values obtained in the experiments.

Table S63. Mean values of v_0 (Δ fluorescence/min) obtained at each substrate (pyruvate) and inhibitor (**20**) concentration for enzyme *h*LDHA in one of the two independent experiments that were carried out following the protocol above.

Pyruvate (mM)	Concentration of 20 (µM)			
	0	0.050	0.125	0.500
1.000000	-2624.74242	-2249.70691	-777.890224	-164.725297
0.6666667	-2506.97885	-1570.87628	-1084.844	-199.810853
0.4444444	-2699.99858	-2162.35497	-1016.1444	-241.882999
0.2962963	-2302.09936	-1796.67583	-990.599198	-163.00283
0.1975309	-1766.48111	-1473.81907	-691.597128	-168.415464
0.1316872	-1110.13214	-861.55893	-467.729032	-135.565738
0.0877915	-647.897771	-546.4394	-339.675068	-65.5656717
0.05852766	-335.734934	-291.265893	-171.077915	-42.1674724
0.03901844	-195.891104	-142.403177	-92.5077054	-26.6430545
0.02601229	-75.207781	1.30838283	-14.4335964	10.7522323

The initial velocity (v_0) was determined as the slope calculated in the linear interval of the "product *vs* time" graph representing the progression of the enzymatic reaction at each substrate concentration. This linear interval was of 10 min after a total measuring time of 15 min. The represented values of v_0 are the mean of three determinations.

Table S64. Values of V_{max} and K_M for *h*LDHA obtained at each tested concentration of **20** using the data in Table S63.

	Concentration of 8q			
	0 uM	0.050 uM	0.125 uM	0.500 uM
V_{max} (Δfluorescence/min)	3729	2855	1267	251.4
K_M (mM)	0.2759	0.2660	0.1941	0.1633
**95% Confidence Intervals**				
V_{max}	2855 to 5167	2052 to 4405	912.6 to 1856	178.6 to 371.4
K_M	0.1428 to 0.5643	0.1156 to 0.6805	0.07944 to 0.4852	0.06147 to 0.4370

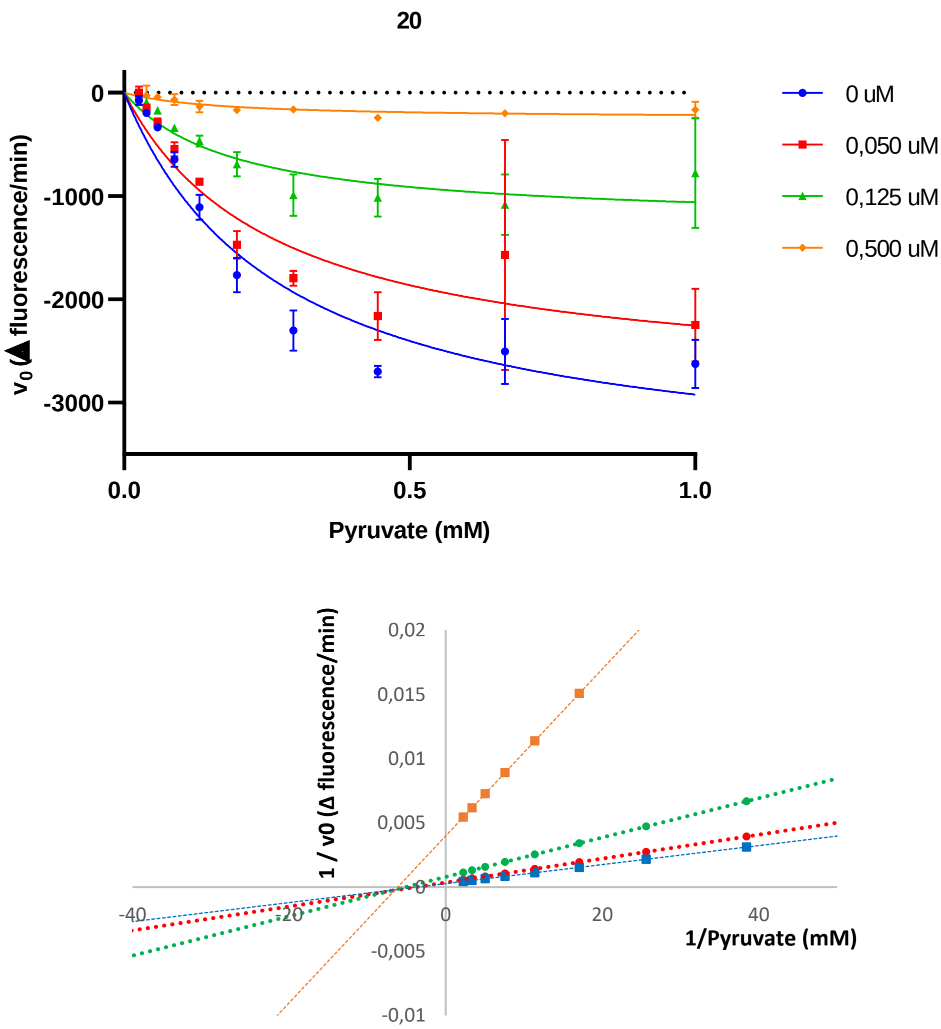

Figure S64. Non-linear concentration *vs* initial velocity (above) and Lineweaver-Burk (below) plots for compound **20** on enzyme *h*LDHA (using four concentrations of inhibitor and ten different concentrations of pyruvate; measurements every minute during 10 min) (built from data in Table S63). Colour code indicates concentration of **20**: 0.5 µM (orange); 0.125 µM (green); 0.05 µM (red) and 0 µM (blue).

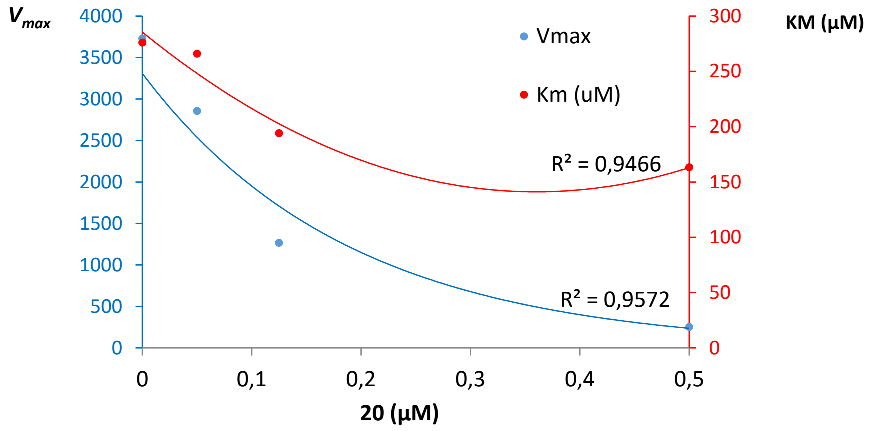

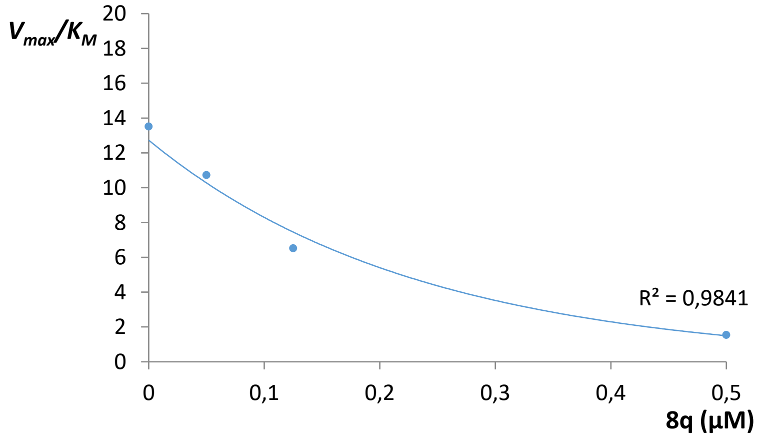

Figure S65. Observed variation on the parameters V_{max} (Δ fluorescence/min) and K_M (µM) and the ratio V_{max}/K_M with increasing concentrations of the inhibitor **20**, showing non-competitive inhibition (built from data in Table S64). Plot above: Curvilinear decrease of V_{max} and no increasing or slightly decreasing trend of K_M. Plot below: Curvilinear decrease of the ration V_{max}/K_M .

Table S65. Parameters of noncompetitive and mixed-model inhibition obtained for the compound **20** on *h*LDHA.

	**NON COMPETITIVE INHIBITION**	**MIXED MODEL INHIBITION**
**K_i (uM)**	0.09253	0.1137
**α**	-	0.7294
Std. Error K_i	0.007625	0.04072
Std. Error α	-	0.3766
95% Confidence Intervals K_i	0.07708 to 0.1080	0.03108 to 0.1962
95% Confidence Intervals α	-	0.000 to 1.493
R square	0.9725	0.9729

Table S66. Statistical parameters in the comparison between non-competitive mixed model inhibition and non-competitive inhibition ($\alpha = 1$).

Comparison of Fits	
**Null hypothesis**	**Noncompetitive inhibition**
**Alternative hypothesis**	**Mixed model inhibition**
**P value**	0.5014
**Conclusion (alpha = 0.05)**	Do not reject null hypothesis
**Preferred model**	Noncompetitive inhibition
**F (DFn, DFd)**	0.4612 (1, 36)

Table S67. Mean of the kinetics parameters (K_i) obtained in two experiments for noncompetitive inhibition (compound **20**, enzyme *h*LDHA).

Experiment	K_i (uM)
1	0.09
2	0.09
**Mean**	**0.09 ± 0.00**

Compound 23.

For compound **23**, one experiment for K_i and inhibition mechanism was carried out following the protocol described. In the experiment we used four different inhibitor concentrations and ten different substrate concentrations. The experiment raised one K_i value obtained from the mean of three v_o determinations for each substrate and inhibitor concentration.

Table S68. Mean values of v_0 (Δ fluorescence/min) obtained at each substrate (pyruvate) and inhibitor (**23**) concentration for enzyme *h*LDHA in the experiment carried out following the protocol above.

Pyruvate (mM)	Concentration of 23 (µM)			
	0	0.050	0.075	0.100
1.000000	-4948.00731	-4857.31395	-3192.45604	-2541.65421
0.6666667	-5413.22501	-4580.29385	-2280.64134	-2063.72048
0.4444444	-5144.94609	-4141.28722	-1432.56089	-1531.8397
0.2962963	-4467.91841	-3119.16783	-774.765252	-634.434491
0.1975309	-3443.28614	-1730.97915	-896.96004	-746.286309
0.1316872	-1957.16106	-1026.33973	-859.532976	-628.637626
0.0877915	-1212.19754	-624.498929	-788.881573	-677.967222
0.05852766	-717.05608	-278.437545	-471.803166	-440.894612
0.03901844	-279.361954	-191.003032	-397.813139	-269.027967
0.02601229	-304.869246	-142.826867	-153.825539	-77.8301198

The initial velocity (v_0) was determined as the slope calculated in the linear interval of the “product *vs* time” graph representing the progression of the enzymatic reaction at each substrate concentration. This linear interval was of 10 min after a total measuring time of 15 min. The represented values of v_0 are the mean of three determinations.

Table S69. Values of V_{max} and K_M for *h*LDHA obtained at each tested concentration of **23** using the data in Table S68.

	**Concentration of 23 (μM)**			
	**0**	**0.050**	**0.075**	**0.100**
V_{max} (Δfluorescence/min)	7458	8964	11007	6149
K_M (mM)	0.2907	0.6941	2.544	1.410
**95% Confidence Intervals**				
V_{max}	-8681 to -6498	-11336 to -7418	-infinity to -5433	-17876 to -3792
K_M	0.2095 to 0.4086	0.4889 to 1.033	0.9318 to +infinity	0.6478 to 5.475

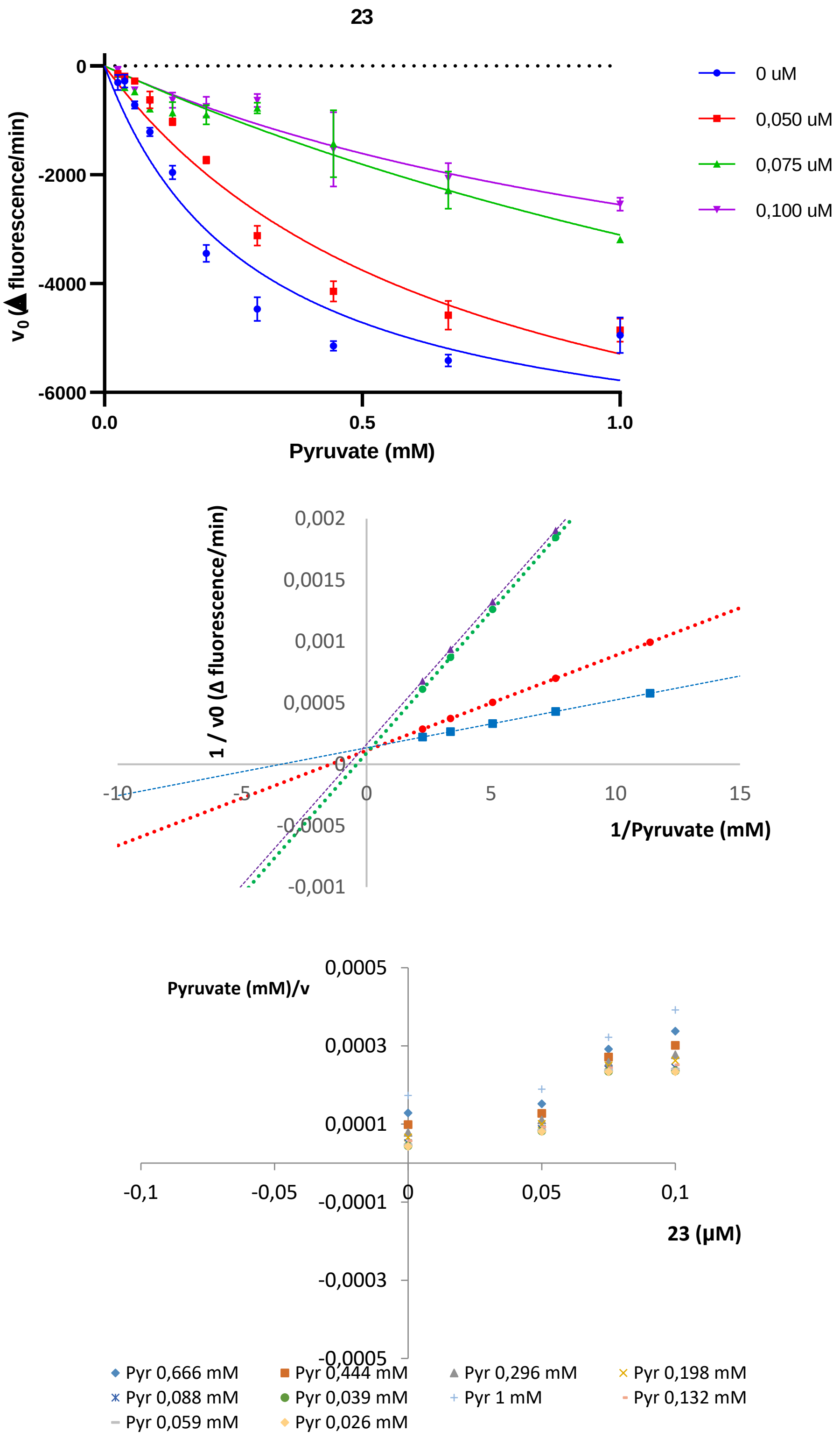

Figure S66. Non-linear concentration *vs* initial velocity (above) and Lineweaver-Burk (middle) plots for compound **23** on enzyme *h*LDHA. Colour code indicates concentration of **23**: 0.1 µM (purple); 0.075 µM (green); 0.05 µM (red) and 0 µM (blue). Cornish-Bowden (below) plot. All plots are prepared using four concentrations of inhibitor and ten different concentrations of pyruvate; measurements every minute during 10 min) (built from data in Table S68).

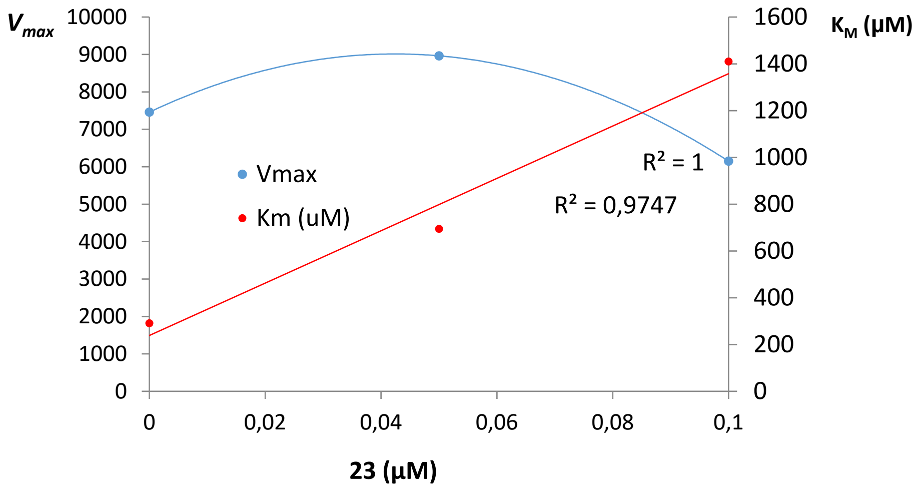

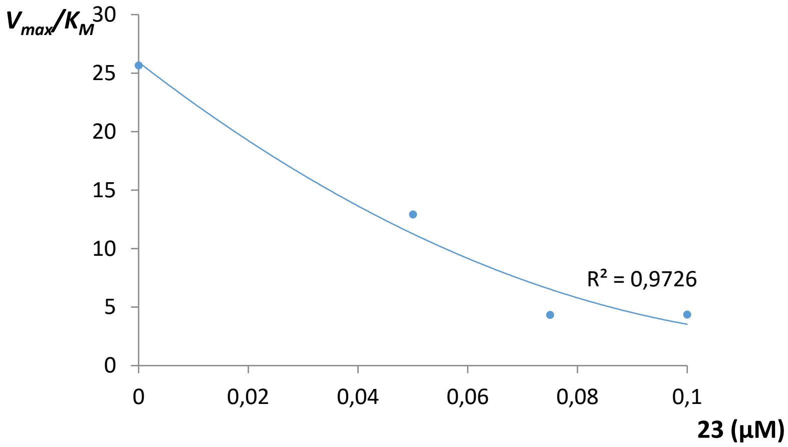

Figure S67. Observed variation on the parameters V_{max} (Δ fluorescence/min) and K_M (µM) and the ratio V_{max}/K_M with increasing concentrations of the inhibitor **23**, showing competitive inhibition (built from data in Table S69). Plot above: No increasing or decreasing trend of V_{max} and linear increase of K_M. Plot below: Curvilinear decrease of the ration V_{max}/K_M.

Table S70. Kinetics parameters (K_i) obtained for competitive inhibition (compound **23**, enzyme *h*LDHA).

	**COMPETITIVE INHIBITION**
**K_i (uM)**	0.02632
95% Confidence Intervals K_i	0.01876 to 0.03715
R square	0.9354

Compound 2

For compound **2**, one experiment for K_i and inhibition mechanism was carried out following the protocol described. In the experiment we used four different inhibitor concentrations and ten different substrate concentrations. The experiment raised one K_i value obtained from the mean of three v_o determinations for each substrate and inhibitor concentration.

Table S71. Mean values of v_0 (Δ fluorescence/min) obtained at each substrate (pyruvate) and inhibitor (**2**) concentration for enzyme *h*LDHA in the experiment carried out following the protocol above.

**Pyruvate (mM)**	**Concentration of 2 (µM)**			
	**0**	**0.010**	**0.040**	**0.120**
1.000000	-3445.84163	-3209.28931	-1454.80234	-342.346049
0.6666667	-3781.67004	-3238.25109	-1858.82005	-502.758091
0.4444444	-3813.44587	-2721.41582	-1826.61477	-398.710764
0.2962963	-2779.93812	-2282.80203	-1445.60075	-315.225685
0.1975309	-1964.52696	-1399.99148	-836.578912	-77.2849175
0.1316872	-1145.90406	-566.743161	-574.001167	-3.48493823
0.0877915	-614.259932	-263.836691	-175.030971	-5.15664821
0.05852766	-512.218994	-246.482952	-113.148484	27.3931416
0.03901844	-331.478553	-163.749372	-120.757776	21.5364547
0.02601229	-167.020108	-106.635833	-100.814265	25.501432

The initial velocity (v_0) was determined as the slope calculated in the linear interval of the "product *vs* time" graph representing the progression of the enzymatic reaction at each substrate concentration. This linear interval was of 10 min after a total measuring time of 15 min. The represented values of v_0 are the mean of three determinations.

Table S72. Values of V_{max} and K_M for *h*LDHA obtained at each tested concentration of **2** using the data in Table S71.

	**Concentration of 2 (µM)**			
	**0**	**0.010**	**0.040**	**0.120**
V_{max} (Δfluorescence/min)	-5549	-5857	-2562	-868.3
K_M (mM)	0.3662	0.6367	0.3603	0.8627
**95% Confidence Intervals**				
V_{max}	-6741 to -4685	-7849 to -4670	-3390 to -2028	-4115 to -485.5
K_M	0.2510 to 0.5478	0.4131 to 1.052	0.2136 to 0.6370	0.3032 to 6.807

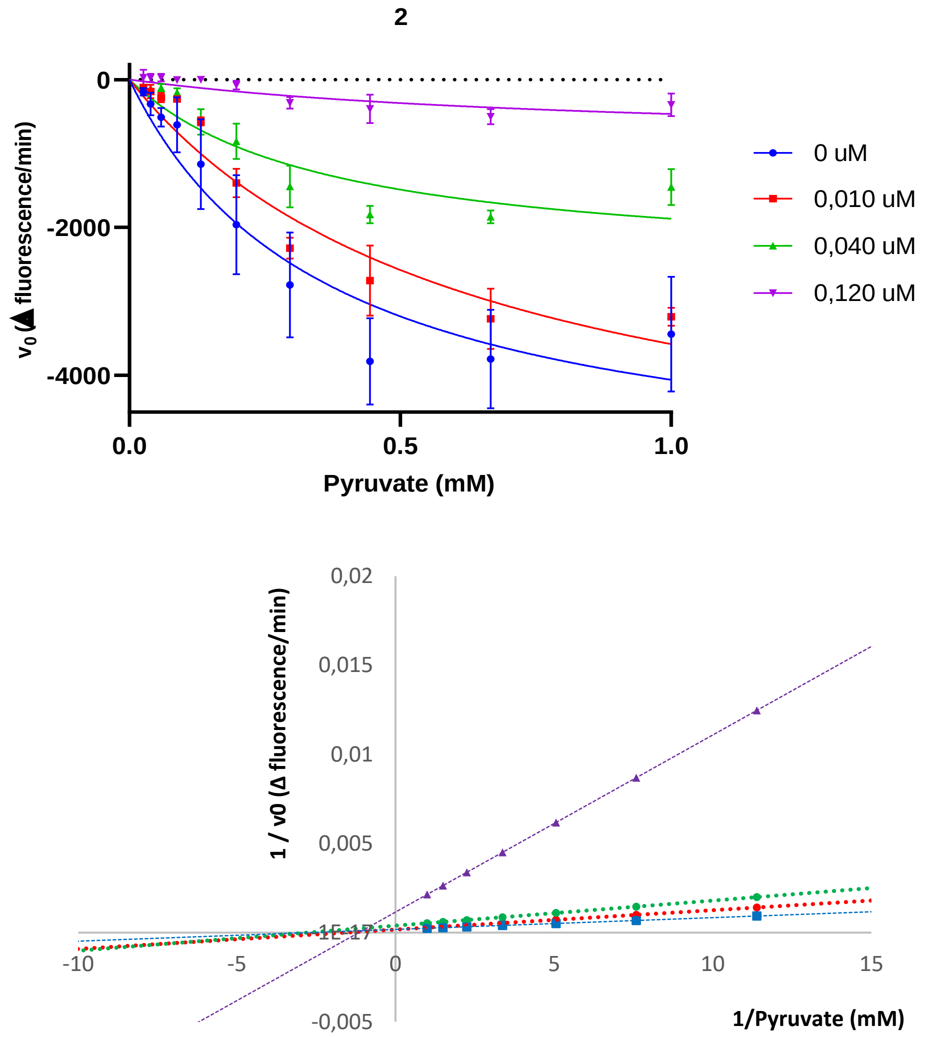

Figure S68. Non-linear concentration *vs* initial velocity (above) and Lineweaver-Burk (below) plots for compound **2** on enzyme *h*LDHA (using four concentrations of inhibitor and ten different concentrations of pyruvate; measurements every minute during 10 min) (built from data in Table S71). Colour code indicates concentration of **2**: 0.12 µM (purple); 0.040 µM (green); 0.010 µM (red) and 0 µM (blue).

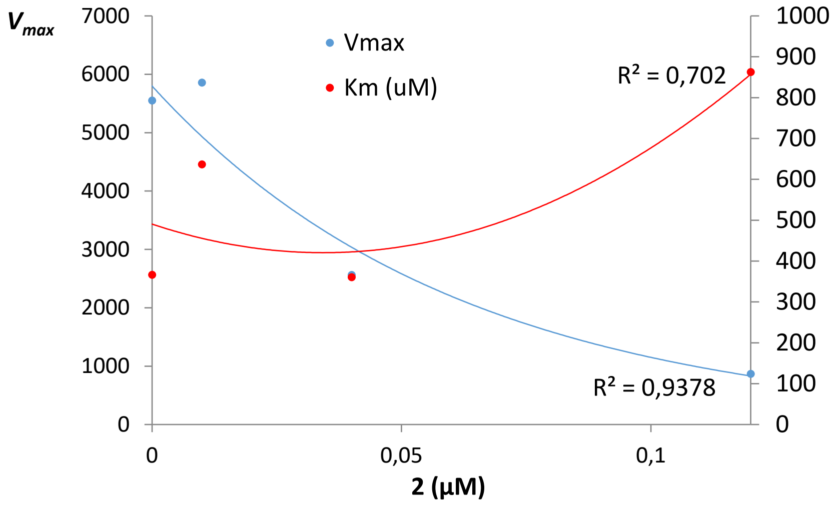

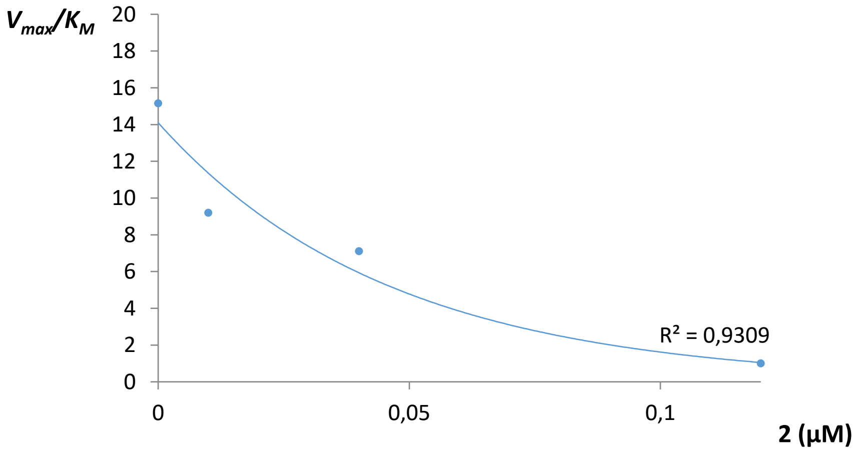

Figure S69. Observed variation on the parameters V_{max} (Δ fluorescence/min) and K_M (µM) and the ratio V_{max}/K_M with increasing concentrations of the inhibitor **2**, showing non-competitive inhibition (built from data in Table S72). Plot above: Curvilinear decrease of V_{max} and no increasing or decreasing trend of K_M. Plot below: Curvilinear decrease of the ration V_{max}/K_M .

Table S73. Parameters of noncompetitive and mixed-model inhibition obtained for the compound **2** on *h*LDHA using the data in Table S71.

	**NON COMPETITIVE INHIBITION**	**MIXED MODEL INHIBITION**
**K_i (uM)**	0.04017	0.04031
**α**	-	0.9934
95% Confidence Intervals K_i	0.03261 to 0.04986	0.02029 to 0.1111
95% Confidence Intervals α	-	0.2139 to 5.485
R square	0.9571	0.9571

Table S74. Statistical parameters in the comparison between non-competitive mixed model inhibition and non-competitive inhibition (α = 1).

Comparison of Fits	
**Null hypothesis**	**Noncompetitive inhibition**
**Alternative hypothesis**	**Mixed model inhibition**
**P value**	0.9926
**Conclusion (alpha = 0.05)**	Do not reject null hypothesis
**Preferred model**	Noncompetitive inhibition
**F (DFn, DFd)**	8.651e-005 (1, 36)

Evaluation of potential titration effects on enzyme *h*LDHA kinetic assays

Morrison K_i values (**Table S75** and **Figure S70**) for compounds **2**, **20** and **23** on enzyme *h*LDHA, resulted slightly lower than classical K_i values. However, both data remain within the same order of magnitude. Morrison $K_i/[E]_T$ ratios place these inhibitors in the tight-binding regime (0.01 < K_i/[E]T < 10) but well above the titration zone (K_i/[E]T < 0.01).

Table S75. Comparison of classical and Morrison K_i values obtained for compounds **2**, **20** and **23** on enzyme *h*LDHA.

Compound	*h*LDHA K_i (nM) (CI)		$K_{i\text{-Morrison}}/[E]_T$
	Classical	Morrison	
**2**	40 (30-50)	23 (17-31)	9.9
**20**	90 (80-110)	12 (8-16)	5.2
**23**	30 (20-40)	17 (12-24)	7.3

CI: 95% confidence interval. For the calculation of Morrison Ki, the following values have been considered: substrate concentration, 1mM; K_M, 311 µM; concentration of catalytic sites, 2.32 nM.

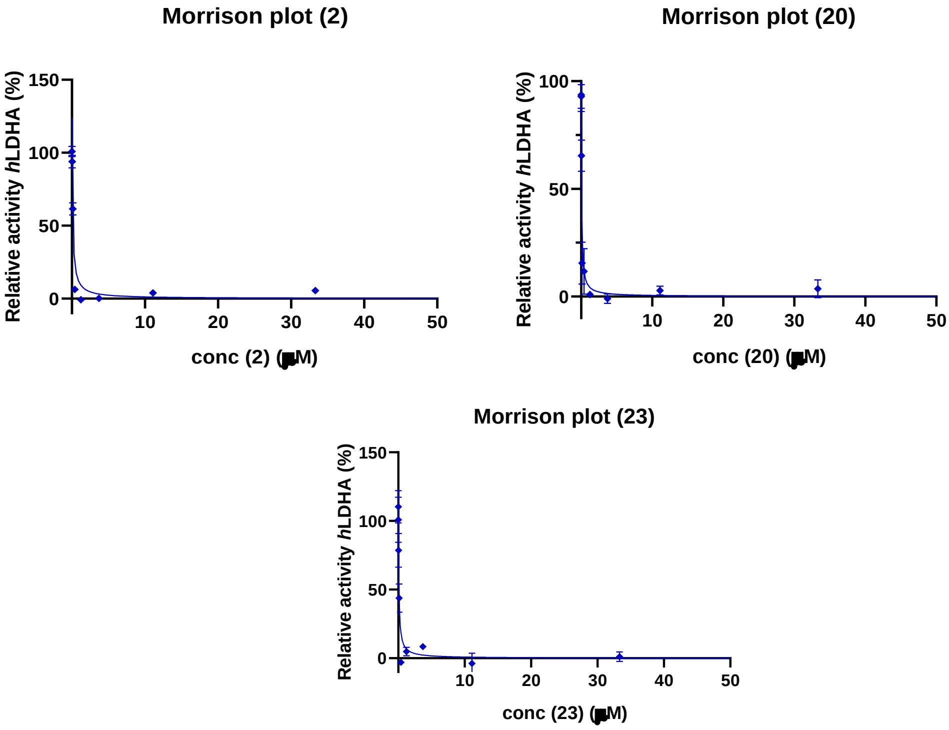

Figure S70. Morrison plots obtained for compounds **2**, **20** and **23**.

S8. Evaluation of compound 2 on enzyme *h*LDHB

Determination of IC_{50}

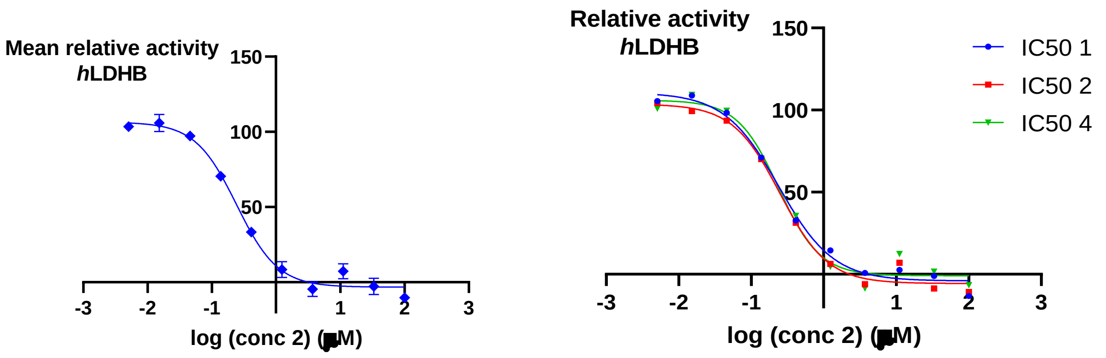

Figure S71. Left: Non-linear "log (conc. **2**) *vs* relative *h*LDHB activity" plot (media of three replicates). Error bars show standard deviation. Right: Non-linear "log (conc. **2**) *vs* relative *h*LDHB activity" plots of the three replicates (using ten concentrations of inhibitor and 1 mM pyruvate).

Table S76. Statistical parameters of the IC_{50} value for inhibition of *h*LDHB obtained for **2**, using ten inhibitor concentrations and 1 mM substrate (pyruvate) concentration (three replicates).

**IC_{50} (uM)**	0.24 ± 0.01
95% Confidence Intervals	0.2016 to 0.2918
R square	0.9863

Determination of K_i and inhibition mechanism

For compound **2**, one experiment for K_i and inhibition mechanism was carried out following the protocol described. In the experiment we used five different inhibitor concentrations and seven different substrate concentrations. The experiment raised one K_i value obtained from the mean of three v_o determinations for each substrate and inhibitor concentration.

Table S77. Mean values of v_0 (Δ fluorescence/min) obtained at each substrate (pyruvate) and inhibitor (**2**) concentration for enzyme *h*LDHB in the experiment carried out following the protocol above.

**Pyruvate (mM)**	**Concentration of 2 (μM)**				
	**0**	**0.2**	**0.4**	**1.0**	**1.2**
0.2962963	-2082.021	-1982.719	-2113.043	-1337.802	-1712.171
0.1975309	-2236.194	-1982.726	-1754.449	-1113.596	-1802.088
0.1316872	-2284.293	-1897.354	-1581.941	-792.9116	-1405.532
0.0877915	-1665.815	-1539.399	-1182.187	-1003.282	-1058.038
0.05852766	-1063.44	-1017.372	-1070.254	-950.7346	-817.8608
0.03901844	-643.8568	-619.762	-649.9927	-685.6838	-536.3426
0.02601229	-269.807	-317.5986	-241.1076	-308.075	-222.1493

The initial velocity (v_0) was determined as the slope calculated in the linear interval of the "product *vs* time" graph representing the progression of the enzymatic reaction at each substrate

concentration. This linear interval was of 10 min after a total measuring time of 15 min. The represented values of v_0 are the mean of three determinations.

Table S78. Values of V_{max} and K_M for *h*LDHB obtained at each tested concentration of **2** using the data in Table S77.

	**Concentration of 2 (μM)**				
	**0**	**0.2**	**0.4**	**1.0**	**1.2**
V_{max} (Δfluorescence/min)	-3319	-2987	-3152	-2821	-1380
K_M (mM)	0.1098	0.1077	0.1446	0.1481	0.08044
**95% Confidence Intervals**					
V_{max}	-5350 to -1289	-4298 to -1675	-4214 to -2089	-4011 to -1631	-1792 to -968.6
K_M	0.000 to 0.2614	0.00015 to 0.2153	0.04494 to 0.2443	0.02137 to 0.2747	0.01954 to 0.1413

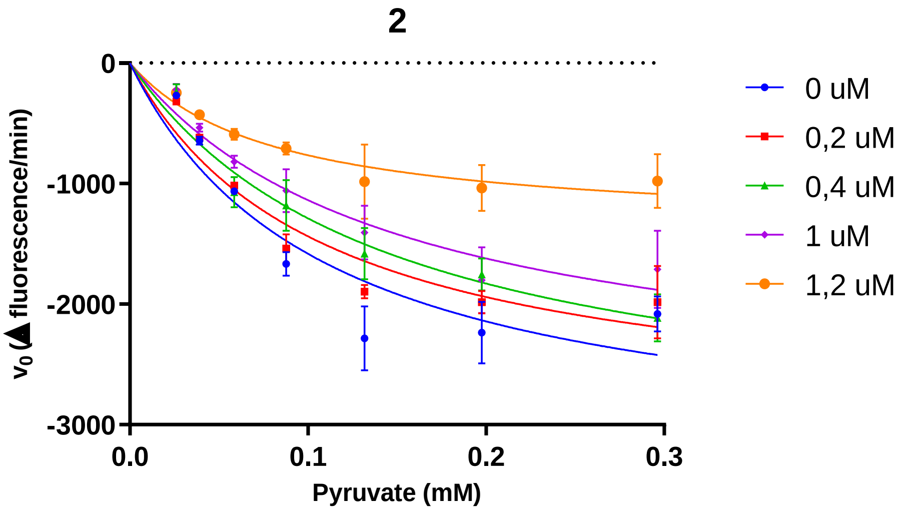

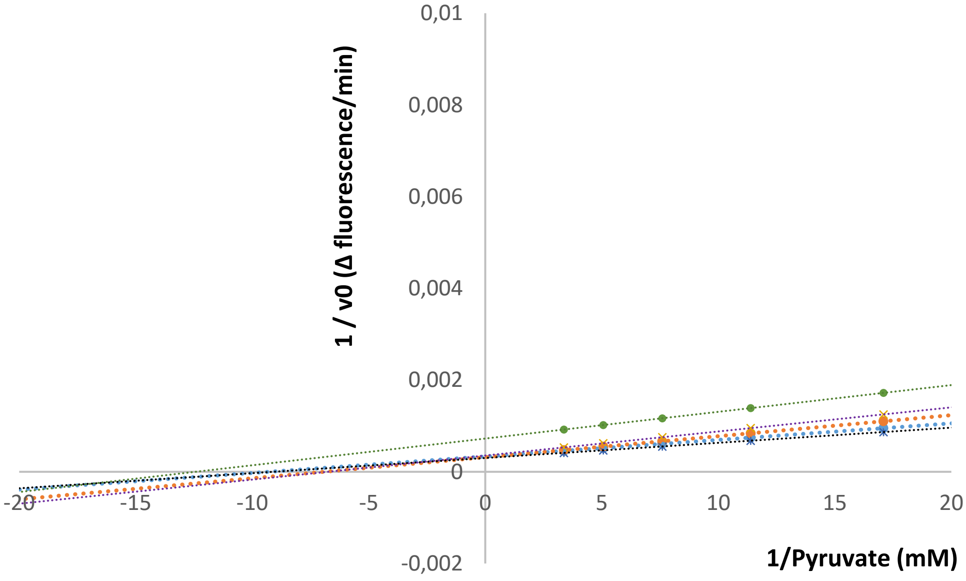

Figure S72. Non-linear concentration *vs* initial velocity (above) and Lineweaver-Burk (below) plots for compound **2** on enzyme *h*LDHB (using five concentrations of inhibitor and seven different concentrations of pyruvate; measurements every minute during 10 min) (built from data in Table S77). Colour code indicates concentration of **2**: 1.2 µM (green);1.0 µM (purple); 0.4 µM (orange); 0.2 µM (blue) and 0 µM (black).

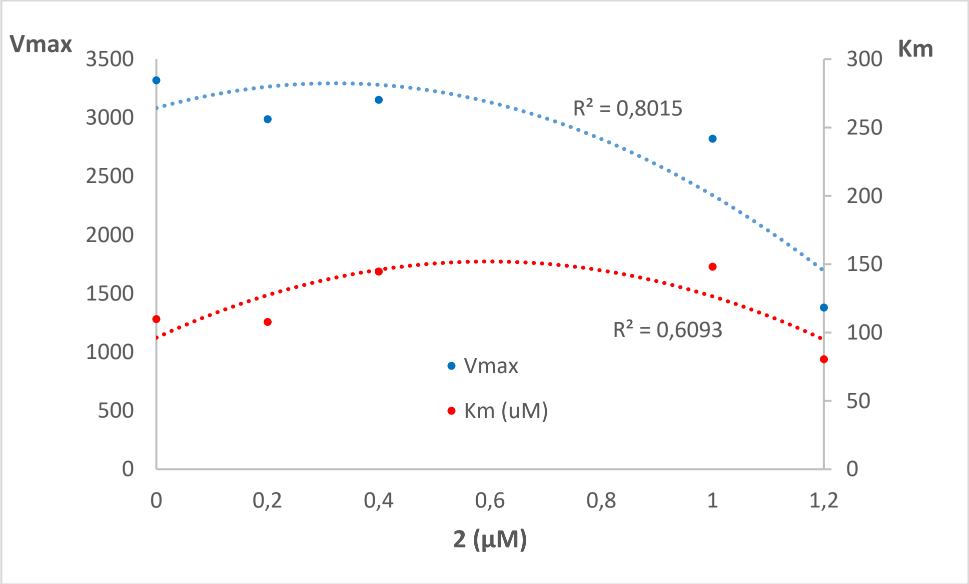

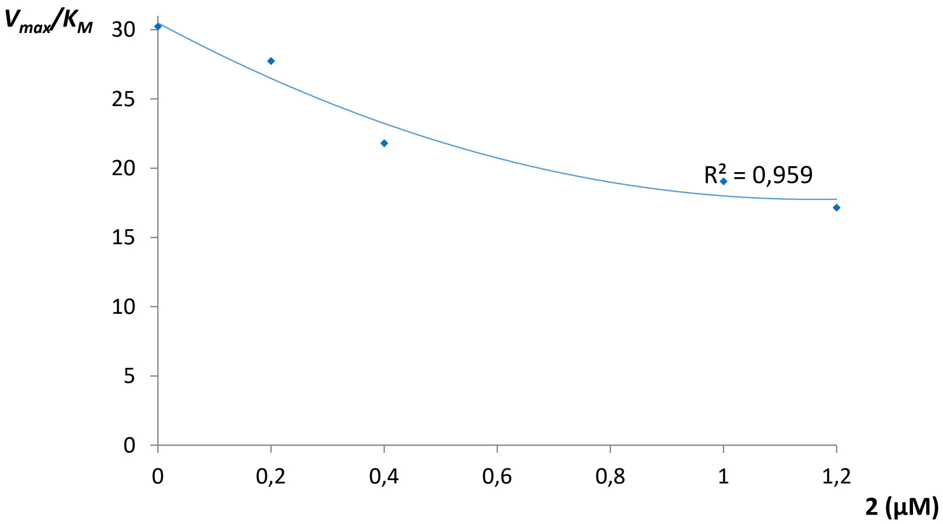

Figure S73. Observed variation on the parameters V_{max} (Δ fluorescence/min) and K_M (µM) and the ratio V_{max}/K_M with increasing concentrations of the inhibitor **2**, showing non-competitive inhibition (built from data in Table S78). Plot above: Curvilinear decrease of V_{max} and no increasing or decreasing trend of K_M. Plot below: Curvilinear decrease of the ration V_{max}/K_M .

Table S79. Parameters of noncompetitive and mixed-model inhibition obtained for the compound **2** on *h*LDHB using the data in Table S78.

	**NON COMPETITIVE INHIBITION**	**MIXED MODEL INHIBITION**
**K_i (uM)**	1.653	1.475
**α**	-	1.228
95% Confidence Intervals K_i	1.335 to 2.100	0.7216 to 5.471
95% Confidence Intervals α	-	0.1923 to 7.335
R square	0.9534	0.9535

Table S80. Statistical parameters in the comparison between non-competitive mixed model inhibition and non-competitive inhibition (α = 1).

Comparison of Fits	
**Null hypothesis**	**Noncompetitive inhibition**
**Alternative hypothesis**	**Mixed model inhibition**
**P value**	0.7904
**Conclusion (alpha = 0.05)**	Do not reject null hypothesis
**Preferred model**	Noncompetitive inhibition
**F (DFn, DFd)**	0.07188 (1, 31)

S9. Assessment of GO and LDHA degradation by compound 26

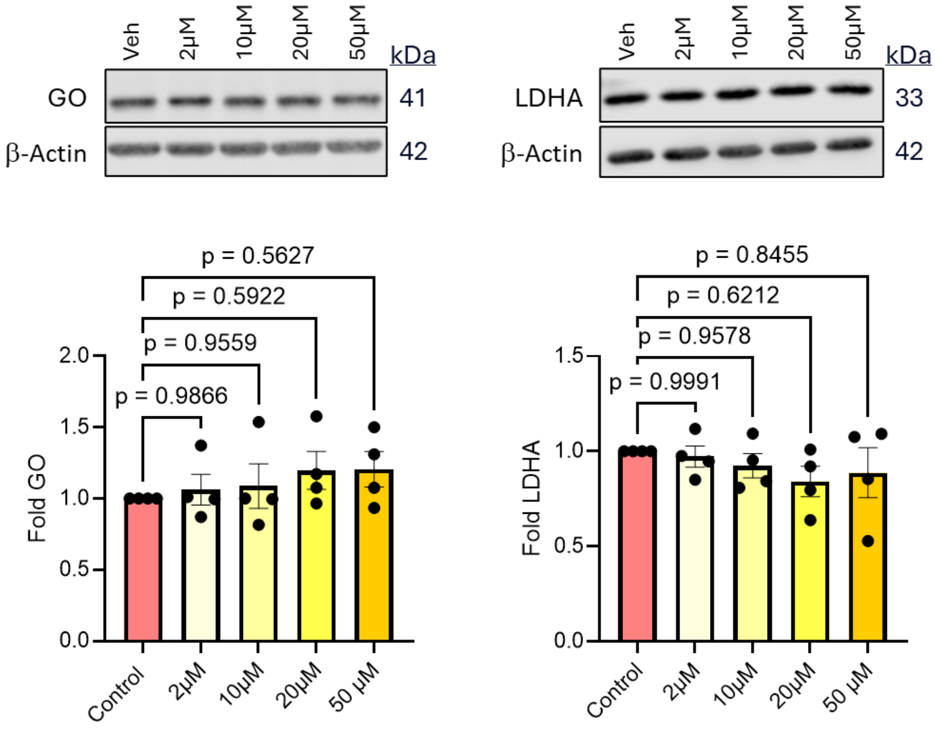

Figure S74. Determination of the proteolytic activity of compound **26**. *Agxt*$^{-/-}$ primary hepatocytes were isolated and treated with either vehicle (DMSO) or increasing concentrations of **26** (2, 10, 20 and 50 μM) for 24 h. **Left**. GO protein abundance and quantification relative to β-Actin (n = 4). **Right**. LDHA protein abundance and quantification relative to β-Actin (n = 4). Data are expressed as mean ± SEM. Normality was assessed using the Shapiro-Wilk test. Comparisons among multiple groups were analyzed using one-way ANOVA followed by Tukey's post hoc test.

S10. Determination of the mechanism for the compound 2-mediated degradation of *h*LDHA

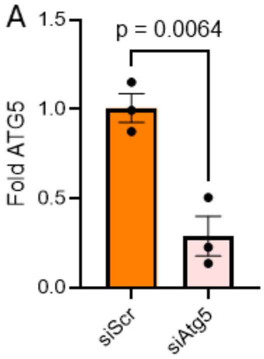

Figure S75. *Agxt*$^{-/-}$ primary hepatocytes were isolated and transfected with either siScr or siAtg5 (20 nM). **A**. Quantification of ATG5 abundance relative to β-Actin (n = 3).

S11. Docking of compound 2

S.11.1. Docking of compound 2 on *h*GO (PDB 2RDT)

Cognate ligand (CDST inhibitor, 5-(dodecylthio)-1H-1,2,3-triazole-4-carboxylic acid) was initially redocked on hGO (PDB ID: 2RDT) to control the validity of the docking analysis (Figure S76). The calculated docking pose shows a similar binding mode to that of the published GO-CDST complex,[3] particularly the 1,2,3-triazole-4-carboxylic moiety. This ring is displayed in the catalytic site forming the same number of h-bonds with the catalytically important residues Tyr26, Arg263, Arg167, His260. The main difference between the cognate ligand and the redocked pose lies in the disposition of the docedylthio chain. While the first section of the chain in the docked pose interact with the important residue Trp110, as does the cognate ligand in the crystal structure 2RDT, the end of the long hydrophobic chain is oriented differently, toward a αE helix located above the entrance of the catalytic site, and interacting with the hydrophobic sidechains of Lys211 and Tyr208.

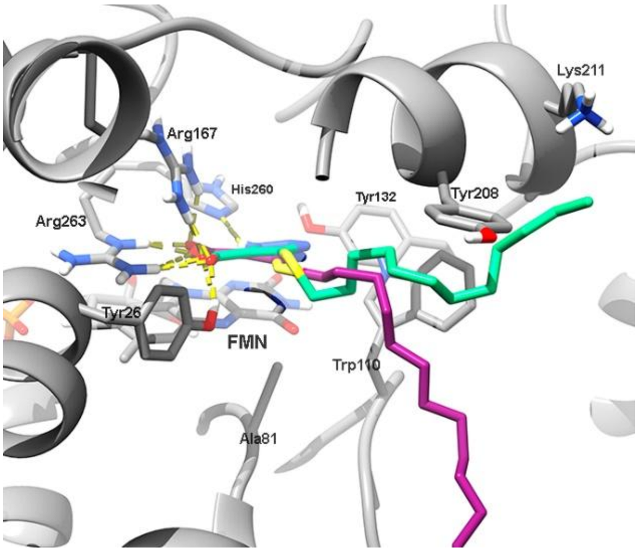

Figure S76. Superposition of cognate crystal structure (purple) and docked pose (green) of CDST in the hGO (2RDT) binding site (grey). Hydrogen bonds are represented by dashed yellow lines.

Ligand **2** was next docked on hGO (PDB ID: 2RDT), and the resulting docking pose is shown in Figure S77. Here, the salicylic moiety of **2** binds into the catalytic hGO site establishing h-bond interactions with the important residues His260, Arg167 and Arg263. The furane ring is set at the entrance of the catalytic site pocket showing a π-stacking interaction with Trp110 on one side and a hydrophobic interaction with Leu205 on the other side of the ring. As for the adamantane methylamino moiety, the hydrophobic adamantane is displayed outside the catalytic pocket and toward a hydrophobic cleft set by residues Ile115, Leu143, Val139 and Tyr134. This region has been identified as an allosteric binding site for novel hGO inhibitors.[4] Moreover, the adamantane orientation is also helped by a h-bond interaction established between the backbone chain of Ala111 and the protonated methylamino moiety.

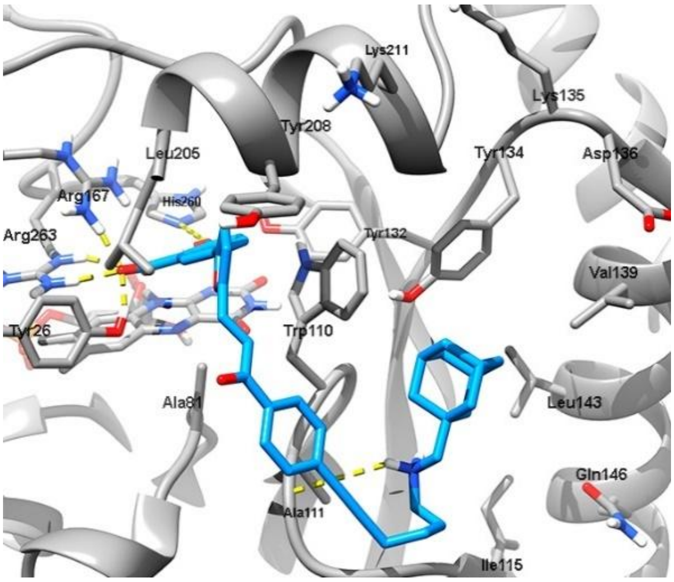

Figure S77. Docking pose of **2** (blue) in the hGO (2RDT) binding site (grey). Hydrogen bonds are represented by dashed yellow lines.

S11.2. Docking of compound 2 on *h*LDHA (PDB 1I10)

Docking studies of **2** were carried out on the apo form of published hLDHA crystal structure with PDB IDs 1I10, portraying a ternary closed conformation of hLDHA. Given the molecular size of **2**, the docking analysis was conducted with a focus on the oxamic acid and cofactor nicotinamide adenine dinucleotide (NAD) binding sites. The final docking pose of **2** (Figure S78a) displays the salicylic moiety well inserted into the catalytic site occupying the same region as oxamic acid (Figure S78b) and binding residues Arg105, His192, and Asn137 as does oxamic acid in the crystal structure.[5] The intermediate carbonylic group in **2** is displayed at the entrance of the catalytic site pocket h-bonding the backbone chain of Ala29, as does the NAD cofactor through one of its oxygen-phosphate groups in the crystalized structure. As for the adamantane methylamino moiety, it is displayed toward the end of the NAD cofactor cleft, with the adamantane group on a hydrophobic region set by Ile115, Val52, Ile119, and Phe118 (Figure S78a).

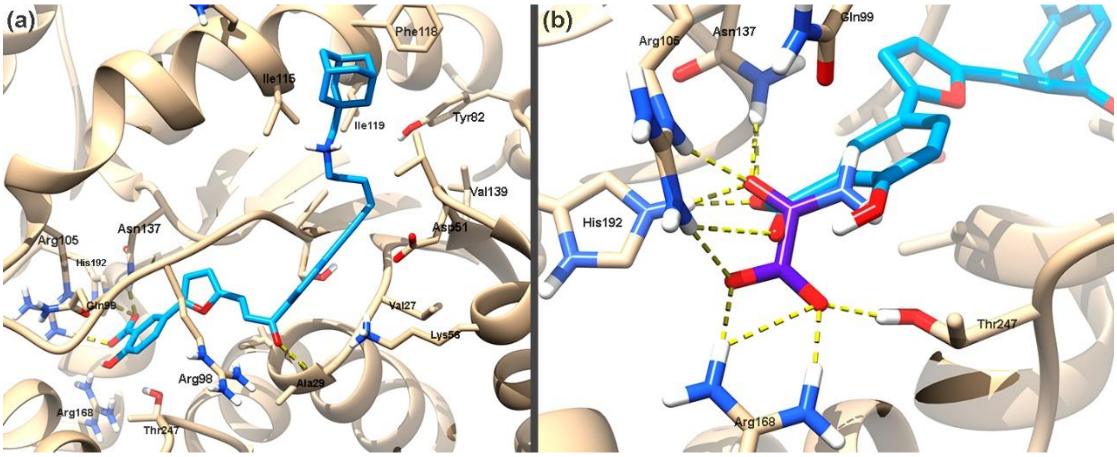

Figure S78. (**a**) Docking pose of **2** (blue) in the hLDHA (1I10) binding site (tan); (**b**) Close-up of the superposed docking pose of **2** (blue) and crystalized oxamic acic (purple). Hydrogen bonds are represented by dashed yellow lines.

S12. Homology modelling and molecular dynamic protocol

S12.1. MD simulation on *h*GO

The stability of both the protein backbone and the **2** ligand during the 100 ns simulation was initially assessed using Root Mean Square Deviation (RMSD) analysis. This analysis measures the average deviation of the atomic positions over time, providing insight into the structural stability of the molecules. The results indicated that both the protein backbone and the **2** ligand reached a stable conformation at approximately 70 ns into the simulation. This stabilization suggests that the molecular interactions between the protein and the ligand are consistent and reliable beyond this point in the simulation (Figure S79).

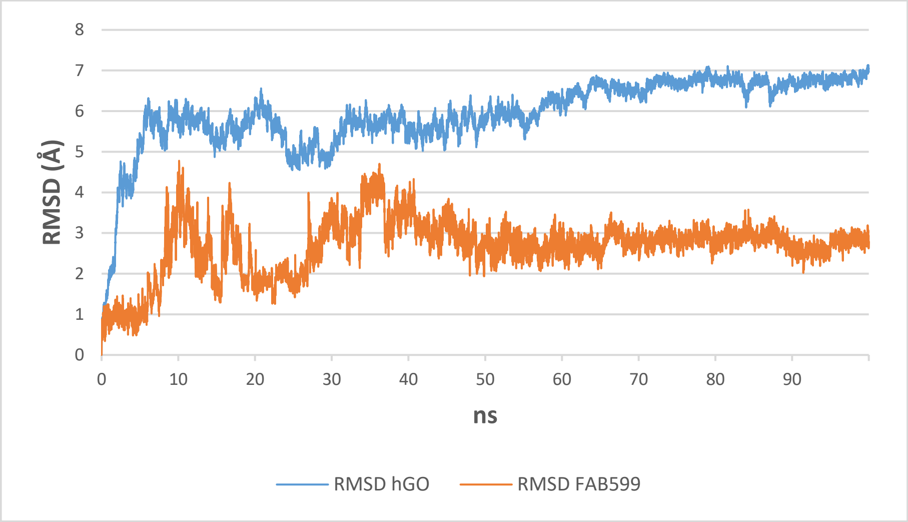

Figure S79. 100 ns MD simulation RMSD graphs of homology-modeled hGO (PDB ID 2RDT) alpha carbons (blue), and standalone ligand **2** (orange) (FAB-599 = **2**).

As previously described, the MD simulation initiates with a homology model of *h*GO, using the docked pose of **2** on PDB ID 2RDT as template. In this crystal structure, the segment of loop 4 encompassing residues 176-204 is missing and was consequently subjected to computational modelling. In the protein structure of the selected model, this loop adopts an extended open conformation exposing its catalytic site, occupied by **2**, to the aqueous surrounding. Close examination of the dynamic of the protein-ligand ensemble during the simulation shows a loop closing within the first 10 ns (Figure S80).

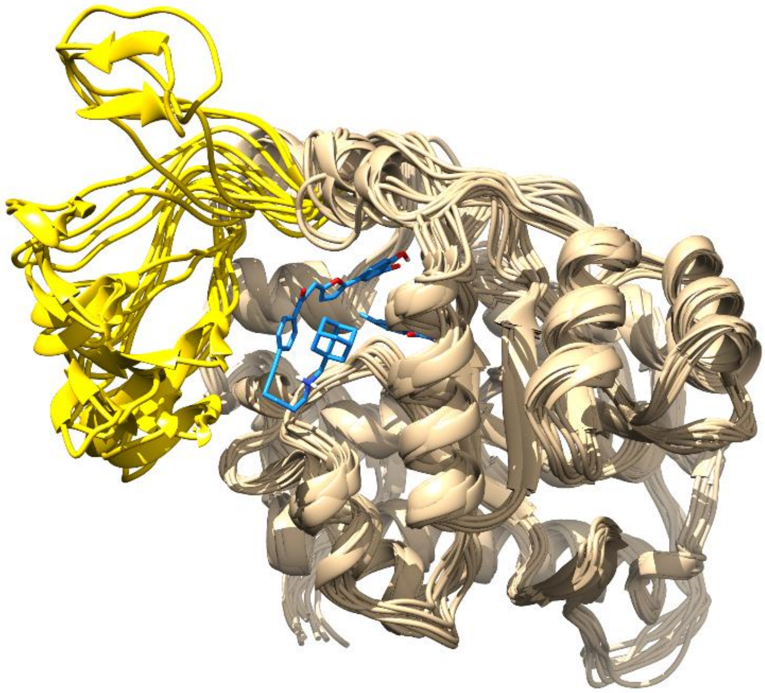

Figure S80. Superpositions of the first 10 frames, starting from frame 0 (t=0) to frame 10 (t=10 ns) at 1 ns interval, are presented. The gating loop (loop 4) is highlighted in yellow, while the rest of the protein is depicted in tan. For reference, **2** and cofactor FMN from frame 0 are shown and colored blue.

As for ligand **2**, its RMSD analysis shows a conformation stabilization at approximately 50 ns. This stabilization is also confirmed by analyzing the distances between the ligand and important catalytic site amino acids, such as Arg167, Arg263, Leu264, Trp110 and Tyr134 whose distances are stabilized toward the end of the 100 ns simulation suggesting a stable ligand conformation (Figure S81).

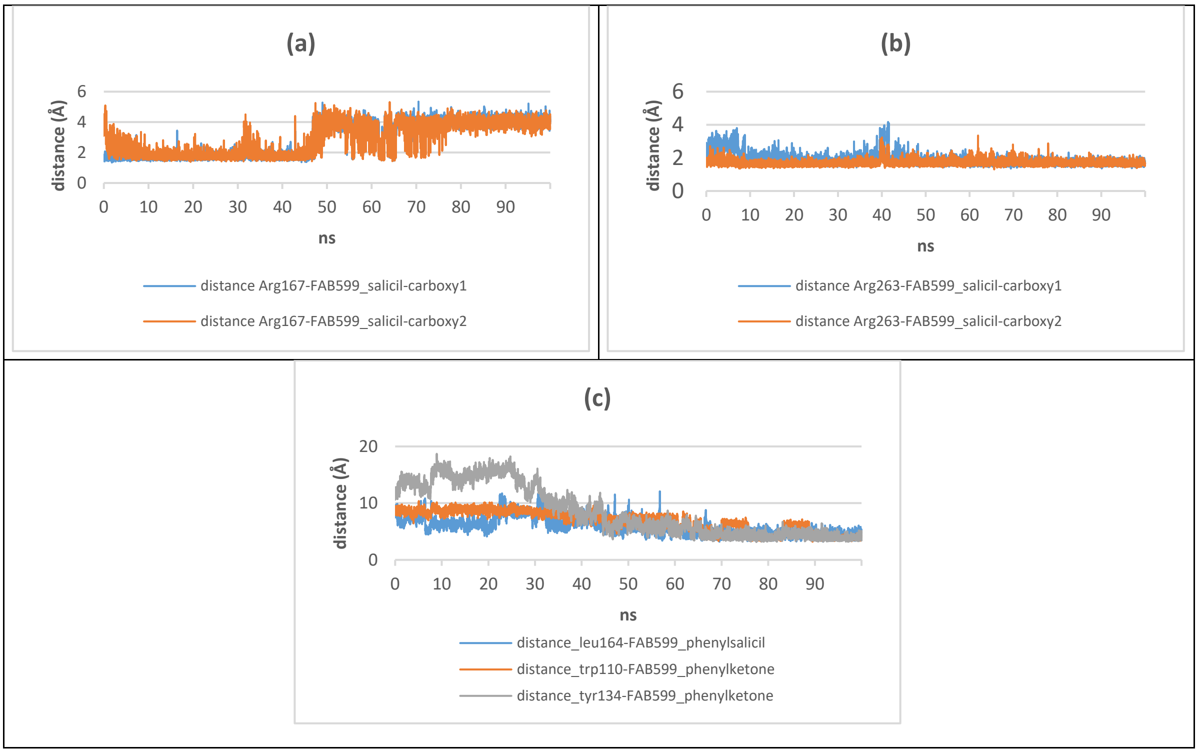

Figure S81. Distances along the 100 ns simulation between **2** and selected amino acids residues. **(a)** Distance (Å) between Arg167 guanidine group and **2** salicylic carboxylic acid oxygen atoms; **(b)** distance (Å) between Arg263 guanidine group and **2** salicylic carboxylic acid oxygen atoms; **(c)** distance (Å) between Leu164 and the phenyl ring of the salicylic ring (blue); Trp110 and **2** phenylketone moiety (orange); Tyr134 and **2** phenylketone moiety (grey).

The stabilization of the system is further validated by a Solvent Accessible Surface Area (SASA) analysis which quantifies the surface area of the protein accessible to the solvent. In this context, both the protein and ligand SASAs show stable values, indicating system stabilization (Figure S82).

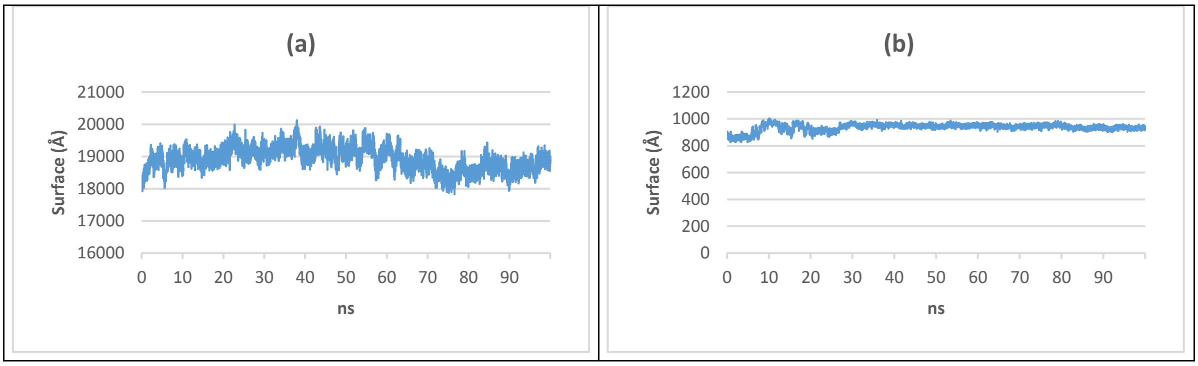

Figure S82. 100 ns simulation SASA graphs of (**a**) hGO and (**b**) ligand **2** on water.

RMDS cluster analysis on the **2**-hGO ensemble MD simulation resulted in the most populated cluster-representative structure depicted in Figure S83 (blue) (t = 100 ns) compared to frame 1 (t = 0) Figure S83 (pink).

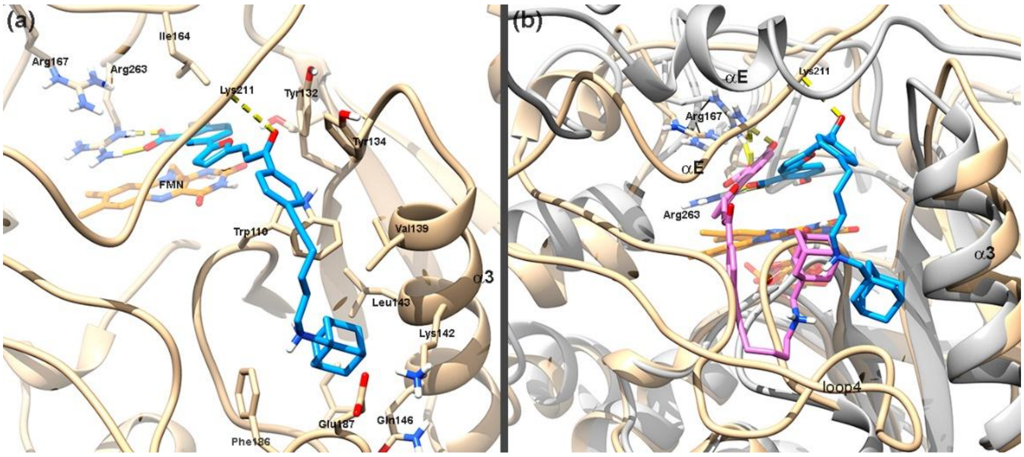

Figure S83. (**a**) Representative pose of **2** (blue) in the most populated cluster of hGO homology model (2RDT) (tan); (**b**) Superposition of the representative pose of **2** (blue) in the most populated cluster of hGO (2RDT) (tan) and frame 0 **2** (pink)-hGO ensemble (grey) that started the MD simulation. Hydrogen bonds are represented by dashed yellow lines.

S12.2. MD simulation on *h*LDHA

The 100 ns MD simulation analysis of the **2**-*h*LDHA dimeric ensemble demonstrates a remarkably stable complex throughout the simulation period. This stability is evident in the RMSD analysis of the protein alpha carbons (Figure S84, blue), where stabilization at 3Å is achieved around 65 ns. When analysed separately, the RMSD of monomer B, which contains the inhibitor **2**, exhibits different behaviour compared to the dimeric *h*LDHA. Notably, in the last 35 ns, there is an approximately 0.5Å stabilization difference observed when compared to the dimeric complex (Figure S84, green). On the other hand, monomer B, which contains the cofactor NAD and inhibitor OXM, exhibits a 0.5Å increase in RMSD during the same last 35 ns of simulation time (Figure S84, orange).

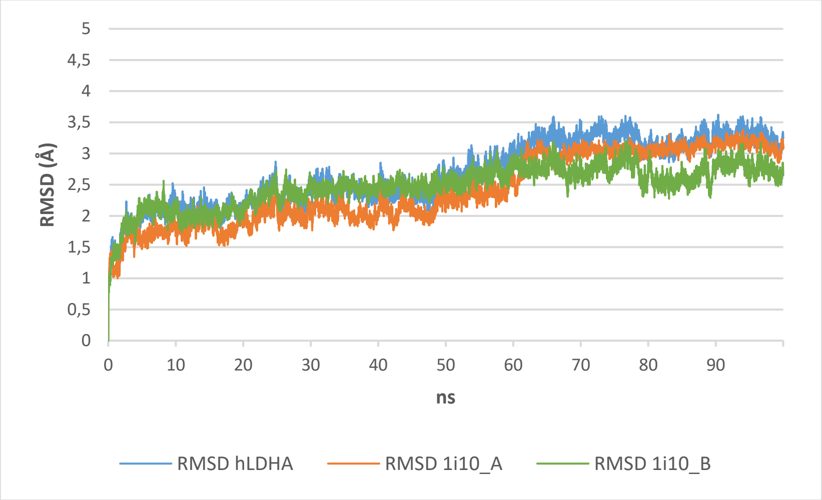

Figure S84. 100 ns simulation RMSD graphs of hLDHA dimeric complex alpha carbons (blue); hLDHA monomer A alpha carbons (orange); hLDHA monomer B alpha carbons (green).

The stabilization induced by **2** when complexing *h*LDHA is also suggested analyzing its Solvent Accessible Surface Area (SASA). Here, an almost steady solvent-exposed surface area is obtained by the end of the simulation time, also pointing to the formation of a stable ligand-protein complex (Figure S85).

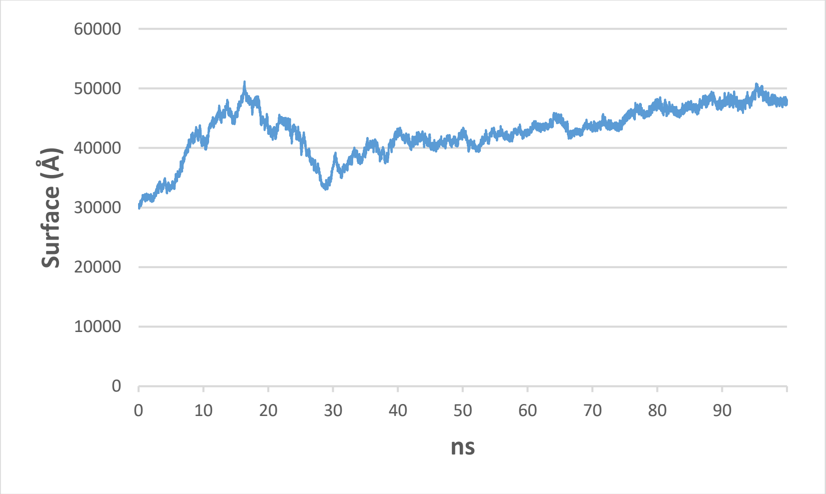

Figure S85. 100 ns simulation SASA graphs of *h*LDHA dimeric complex on water.

Starting from the docked pose, a careful examination of **2** during the 100 ns MD simulation shows a stable conformation on the ligand into the *h*LDHA substrate and cofactor binding sites. RMSD of inhibitor **2** in the 100 ns is shown in Figure S86. The RMDS graph oscillates between 1 to 3,5Å mainly due to the oscillation of the solvent-exposed adamantane tail.

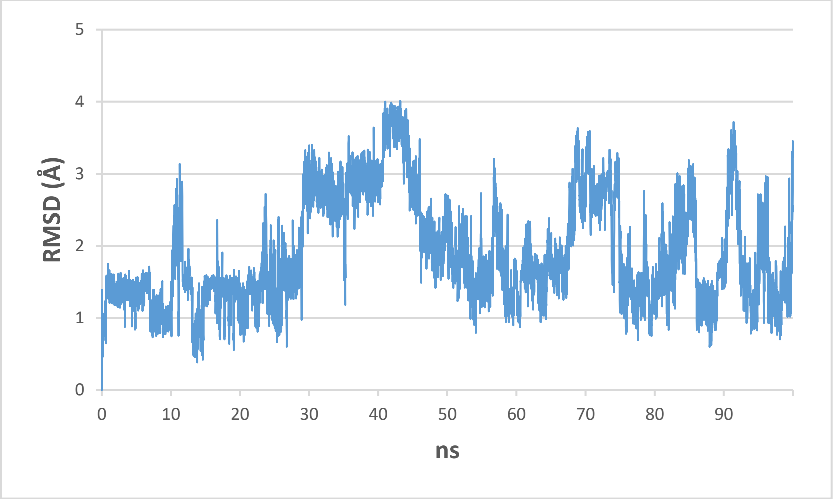

Figure S86. 100 ns simulation RMSD graph of **2** in the dimeric *h*LDHA complex.

The stability of the ligand-protein complex is also observed when analyzing the distances between the ligand and selected amino acids that shape the substrate and cofactor binding sites, such as Arg105, Arg168, Val30 and Arg98 (Figure S87). The consistent distances observed between the ligand and these amino acids throughout the simulation suggest a stable ligand-protein interaction.

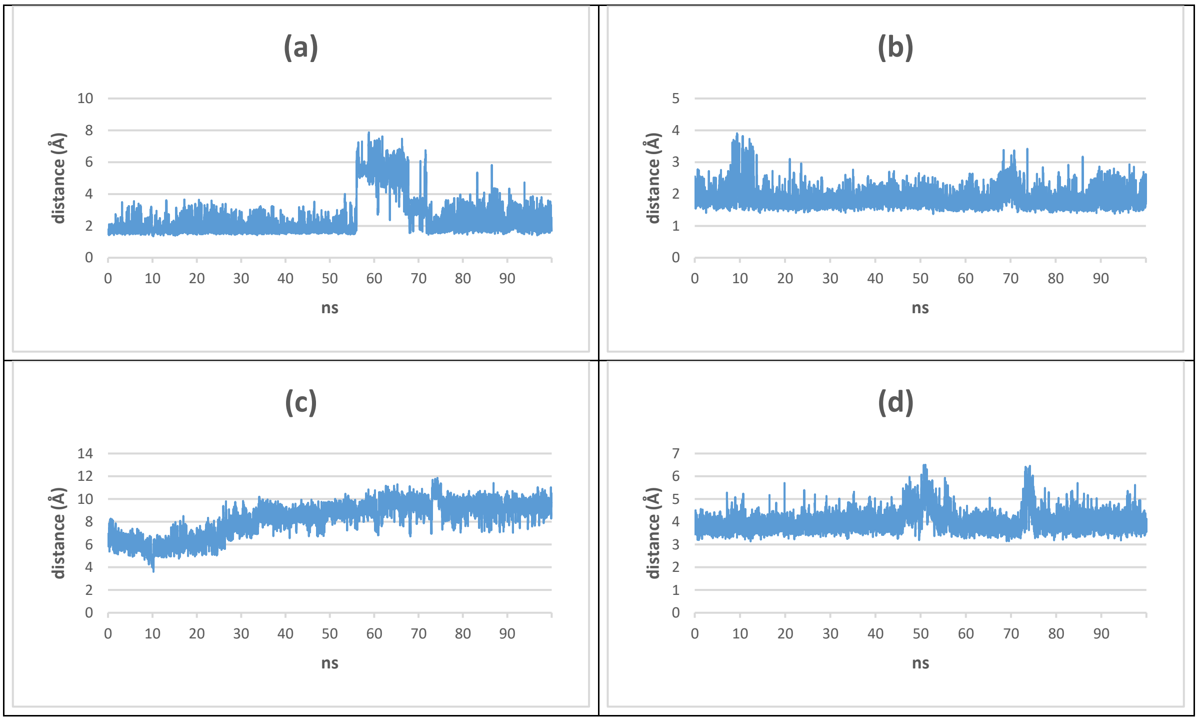

Figure S87. Distances along the 100 ns simulation between **2** and selected amino acids residues. **(a)** Distance (Å) between Arg105 guanidine group and **2** salicylic carboxylic acid; **(b)** distance (Å) between Arg168 guanidine group and **2** salicylic carboxylic acid; **(c)** distance (Å) between Val30 methyl group and **2** furane ring; **(d)** distance (Å) between Arg98 NH and **2** phenyl ring.

A RMDS cluster analysis was also carried out to the **2**-*h*LDHA dimeric ensemble MD simulation. The most populated cluster-representative structure is shown in Figure S88 (blue) (t = 100 ns) compared to frame 1 (t= 0) in Figure S88 (pink).

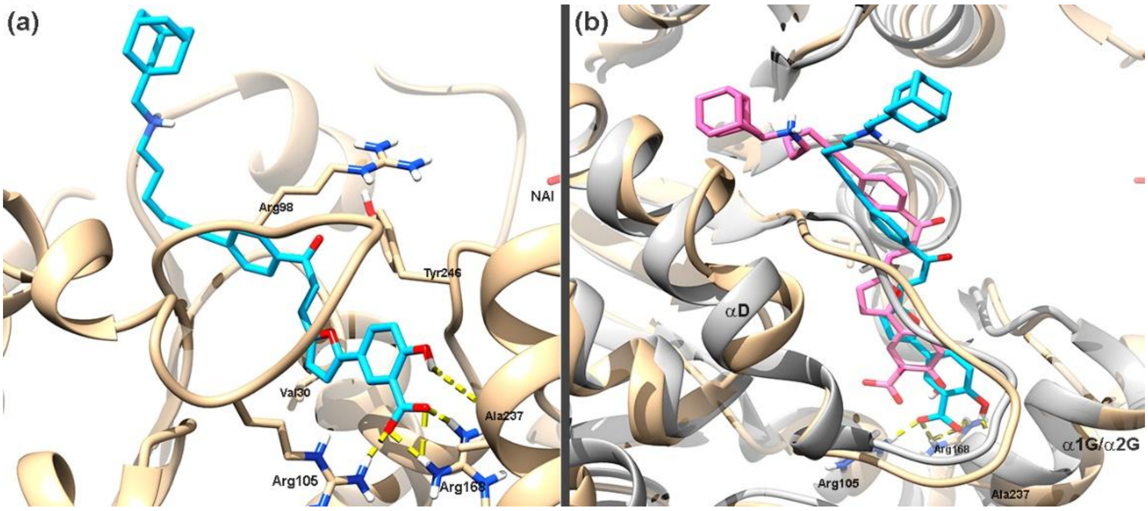

Figure S88. (**a**) Representative pose of **2** (blue) in the most populated cluster of *h*LDHA (1I10) subunit B (tan); (**b**) Superposition of the representative pose of **2** (blue) in the most populated cluster of *h*LDHA (1I10) subunit B (tan) and the frame 0 **2** (pink)-*h*LDHA dimeric ensemble (grey) that started the MD simulation. Hydrogen bonds are represented by dashed yellow lines.

S13. Comparative binding-mode analysis of compounds 2 and 26.

Binding modes in *h*GO: compound 2 vs 26

Both **2** and **26** anchor their salicylic head within the catalytic site of *h*GO (PDB 2RDT), establishing hydrogen bonds with residues Arg263, Arg167, His260, and Tyr26 (Figure S89). These residues correspond to the canonical catalytic triad of *h*GO and are consistent with previously reported interactions for salicylic-based ligands. Molecular dynamics simulations revealed that in *h*GO, the adamantane group of **2** remains buried inside the protein cavity, partially covered by the surface loop, and therefore not solvent-exposed. This structural arrangement may explain the lack of degradation activity observed for *h*GO. Given the shorter linker in **26**, it can be presumed that its adamantane moiety would remain even more tightly enclosed within the hydrophobic pocket, further reducing solvent exposure and likewise preventing degradation.

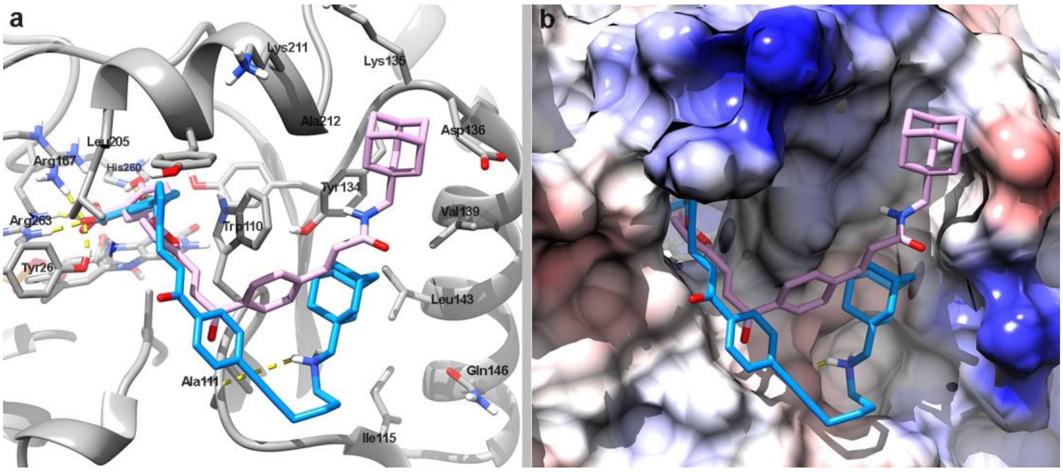

Figure S89. (**a**) Docking poses of **2** (blue) and **26** (pink) in *h*GO (PDB 2RDT). (**b**) Protein surface representation colored by electrostatic potential: blue for positive, red for negative, and white for hydrophobic regions. Yellow dashed lines indicate hydrogen bonds.

Binding modes in *h*LDHA: compound 2 vs 26

In hLDHA, both **2** and **26** insert their salicylic head deeply into the catalytic pocket, forming hydrogen bonds with residues Arg105, Arg168, His192, and Asn137, matching the canonical recognition network described for LDHA inhibitors (Figure S90). The α,β-unsaturated ketone group of both ligands is positioned at the entrance channel, forming a backbone hydrogen bond via the carbonyl group to Ala29. The adamantane tail of **2**, owing to its longer linker, can adopt conformations where the hydrophobic adamantane becomes partially exposed to the solvent, as observed in MD simulations. In contrast, the shorter linker of the **26** may restrict the adamantane moiety to remain adjacent to the protein surface, hindering solvent exposure. This structural constraint may explain why **2** can induce proteolytic degradation of LDHA through hydrophobic-tagging mechanisms, whereas the **26** cannot.

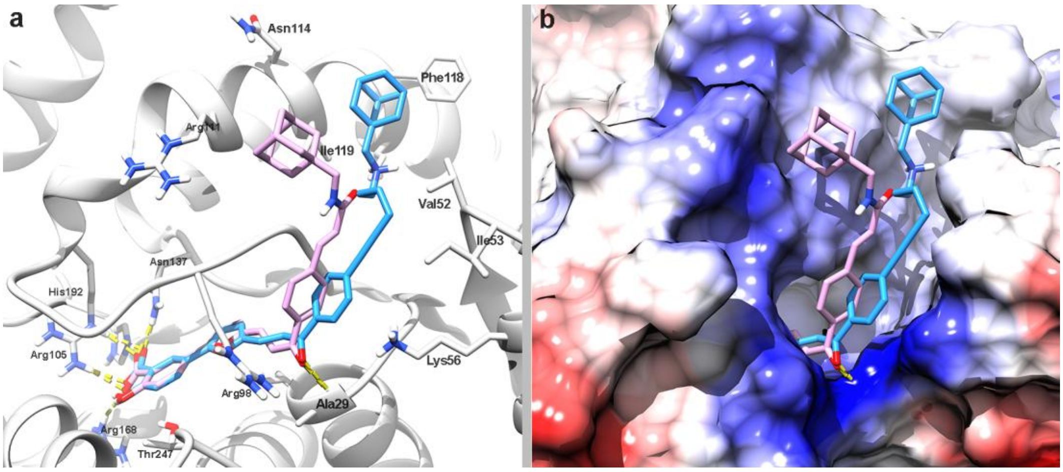

Figure S90. (**a**) Docking poses of **2** (blue) and **26** (pink) in *h*LDHA (PDB 1I10). (**b**) Protein surface representation colored by electrostatic potential (blue: positive, red: negative, white: hydrophobic). Yellow dashed lines indicate hydrogen bonds.

S14. Characterization of chameleonicity of compound 2.

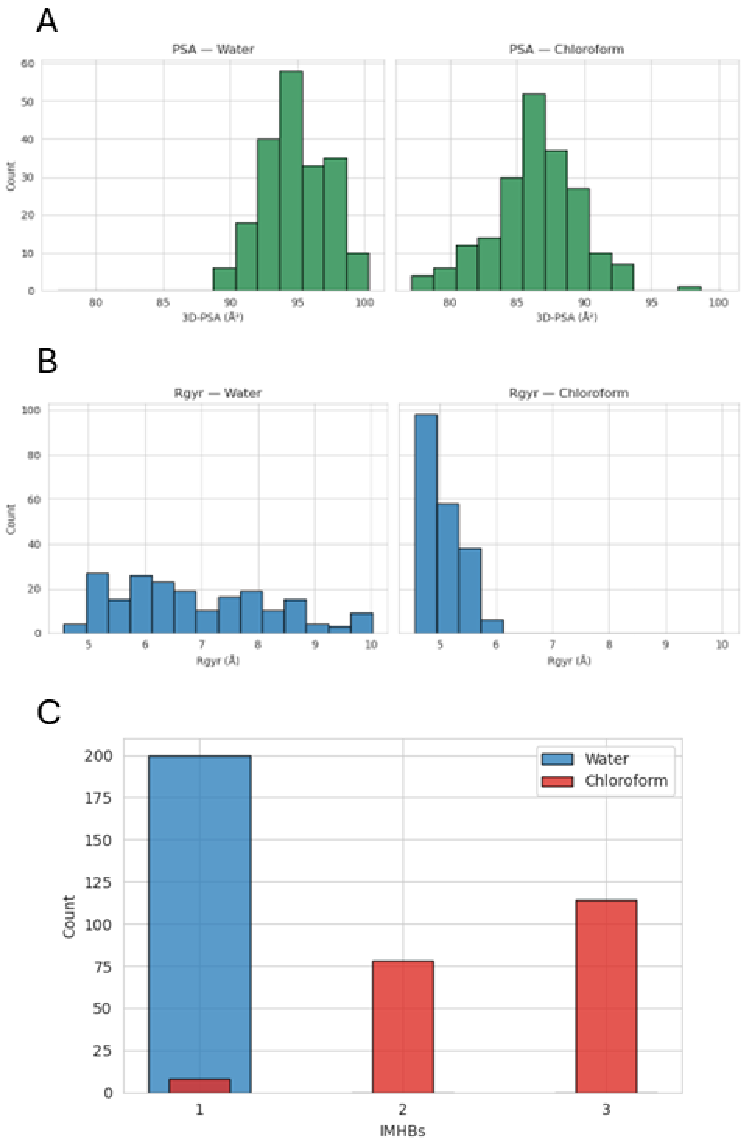

Figure S91. Determination of chameleonicity properties of compound **2**. **A**. Distribution of 3D polar surface area (3D-PSA) values for conformers in water and chloroform. **B**. Distribution of radius of gyration (Rgyr) for conformers in water and chloroform. **C**. Distribution of intramolecular hydrogen bonds (IMHBs) per conformer in water (blue) and chloroform (red).

S15. Evaluation of compound 2 in *Agxt*$^{-/-}$ mice *in vivo*

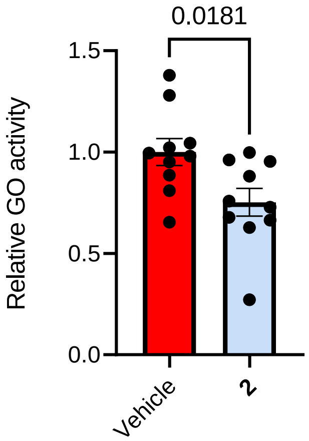

Figure S92. Activity of GO measured in the liver at the end of the treatment with compound **2**. Male *Agxt*$^{-/-}$ mice were orally administered with either vehicle (0.6% methylcellulose and 0.5% Tween® 80 in water, n = 10) or compound **2** (20 mg/kg body weight, n = 10) daily for 10 days. Normality was assessed using the Shapiro-Wilk test. Comparisons between two groups were performed using Student's *t*-test, as normality was assumed in all cases.

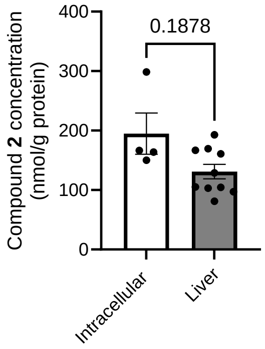

Figure S93. Comparison of intracellular and hepatic levels of compound **2** following treatment. *Agxt*$^{-/-}$ primary hepatocytes were isolated and treated with compound **2** (50 µM, 24 h; n = 4) and male *Agxt*$^{-/-}$ mice were orally administered compound **2** (20 mg/kg body weight, n = 10) daily for 10 days. Compound **2** concentrations were quantified by UPLC-Orbitrap HRMS/MS and normalized to protein concentration. Data are presented as mean ± SEM. Normality was assessed using the Shapiro-Wilk test, and group comparisons were performed using the Mann-Whitney test.

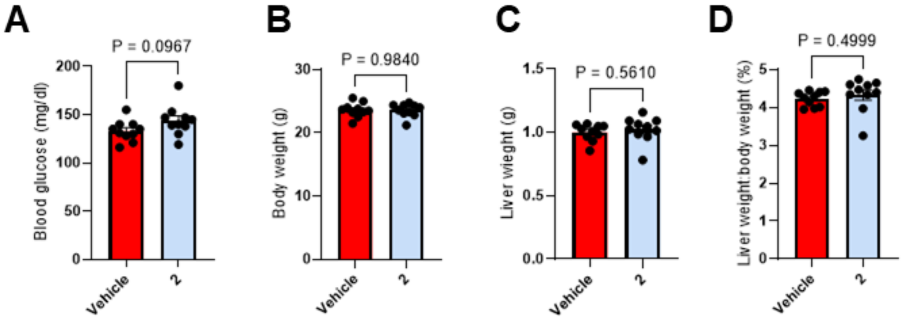

Figure S94. Therapeutic effects of **2** in PH mice. Male *Agxt1*$^{-/-}$ mice were orally administered with either vehicle (0.6% methyl cellulose) (n = 10) or **2** (20mg/kg body weight) (n = 10) daily for 10 days. A. Plasmatic levels of glucose. B. Body weight. C. Liver weight. D. Liver to body weight ratio.

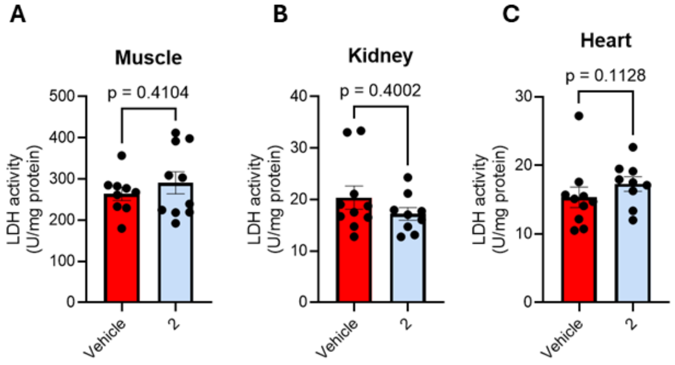

Figure S95. Systemic effects of compound 2 on LDH activity in mice. Male *Agxt*$^{-/-}$ mice were orally administered either vehicle (0.6% methylcellulose and 0.5% Tween® 80 in water; *n* = 10) or compound 2 (20 mg/kg body weight; *n* = 10) daily for 10 days**. A**. LDH activity in gastrocnemius muscle **B**. LDH activity in kidney. **C**. LDH activity in heart**.** Data are presented as mean ± SEM. Normality was assessed using the Shapiro-Wilk test. Comparisons between two groups were performed using Student's *t*-test when data met normality assumptions, or the Mann-Whitney test when normality was not assumed.

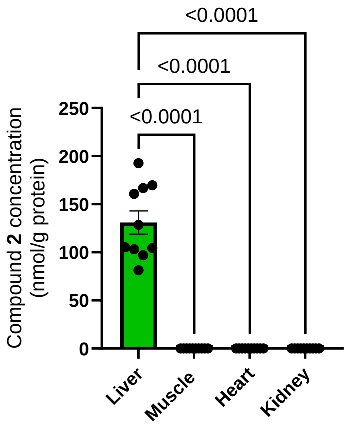

Figure S96. Tissue distribution of compound 2 after oral administration in mice. Male *Agxt*$^{-/-}$ mice were orally administered with compound **2** (20 mg/kg body weight, n = 10) daily for 10 days. Tissues were harvested and homogenized in methanol, and compound **2** concentrations were quantified by UPLC-Orbitrap HRMS/MS and normalized to protein concentration. Data are presented as mean ± SEM. Normality was assessed using the Shapiro-Wilk test, and group comparisons were performed using the Kruskal-Wallis test.

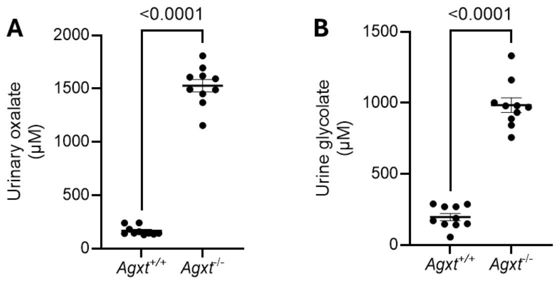

Figure S97. Determination of basal urinary levels in *Agxt*$^{+/+}$ and *Agxt*$^{-/-}$ mice (n = 10). **A**. Oxalate**.** **B**. Glycolate. Data are expressed as mean ± SEM. Normality was assessed using the Shapiro-Wilk test. Comparisons between groups were performed using Students t test or Mann-Whitney test.

S.16. References

(1) Moya-Garzon, M. D.; Rodriguez-Rodriguez, B.; Martin-Higueras, C.; Franco-Montalban, F.; Fernandes, M. X.; Gomez-Vidal, J. A.; Pey, A. L.; Salido, E.; Diaz-Gavilan, M. New Salicylic Acid Derivatives, Double Inhibitors of Glycolate Oxidase and Lactate Dehydrogenase, as Effective Agents Decreasing Oxalate Production. *European Journal of Medicinal Chemistry* **2022**, *237*, 114396. https://doi.org/10.1016/j.ejmech.2022.114396.

(2) Garcia Jimenez, D.; Rossi Sebastiano, M.; Vallaro, M.; Ermondi, G.; Caron, G. IMHB-Mediated Chameleonicity in Drug Design: A Focus on Structurally Related PROTACs. *J. Med. Chem.* **2024**, *67* (13), 11421–11434. https://doi.org/10.1021/acs.jmedchem.4c01200.

(3) Murray, M. S.; Holmes, R. P.; Lowther, W. T. Active Site and Loop 4 Movements within Human Glycolate Oxidase: Implications for Substrate Specificity and Drug Design. *Biochemistry* **2008**, *47* (8), 2439–2449. https://doi.org/10.1021/bi701710r.

(4) Mackinnon, S. R.; Bezerra, G. A.; Krojer, T.; Szommer, T.; von Delft, F.; Brennan, P. E.; Yue, W. W. Novel Starting Points for Human Glycolate Oxidase Inhibitors, Revealed by Crystallography-Based Fragment Screening. *Front. Chem* **2022**, *10*.

(5) Read, J. A.; Winter, V. J.; Eszes, C. M.; Sessions, R. B.; Brady, R. L. Structural Basis for Altered Activity of M- and H-Isozyme Forms of Human Lactate Dehydrogenase. *Proteins: Struct., Funct., Bioinf.* **2001**, *43* (2), 175–185. https://doi.org/10.1002/1097-0134(20010501)43:2<175::AID-PROT1029>3.0.CO;2-#.

S.17. HPLC TRACES

2-Hydroxy-5-[5-(propargylaminomethyl)furan-2-yl]benzoic acid (1)

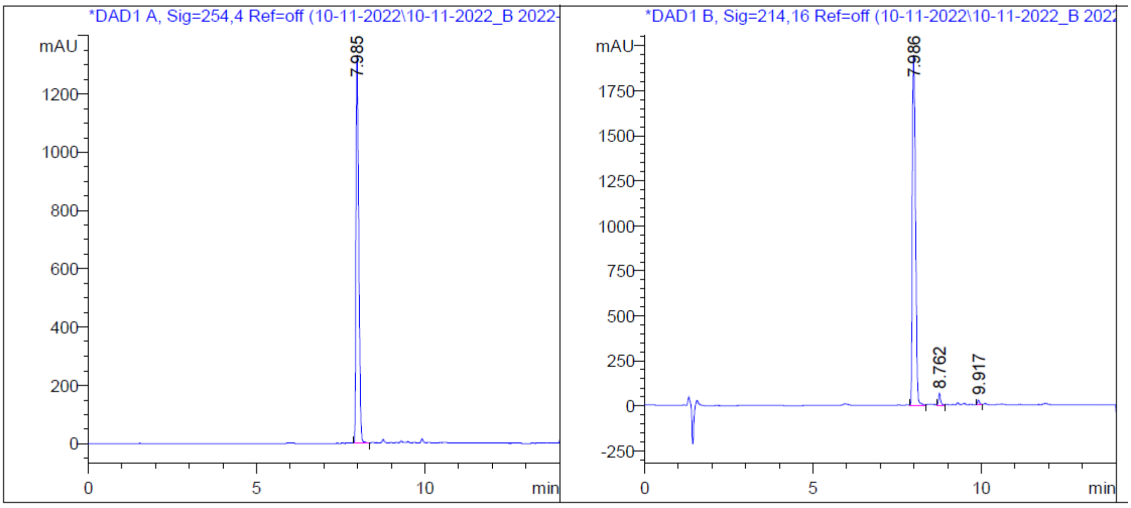

5-{5-{(*E*)-3-{4-[6-(adamantan-1-ylmethylamino)hex-1-ynyl]phenyl}-3-oxoprop-1-enyl}furan-2-yl}-2-hydroxybenzoic acid (2).

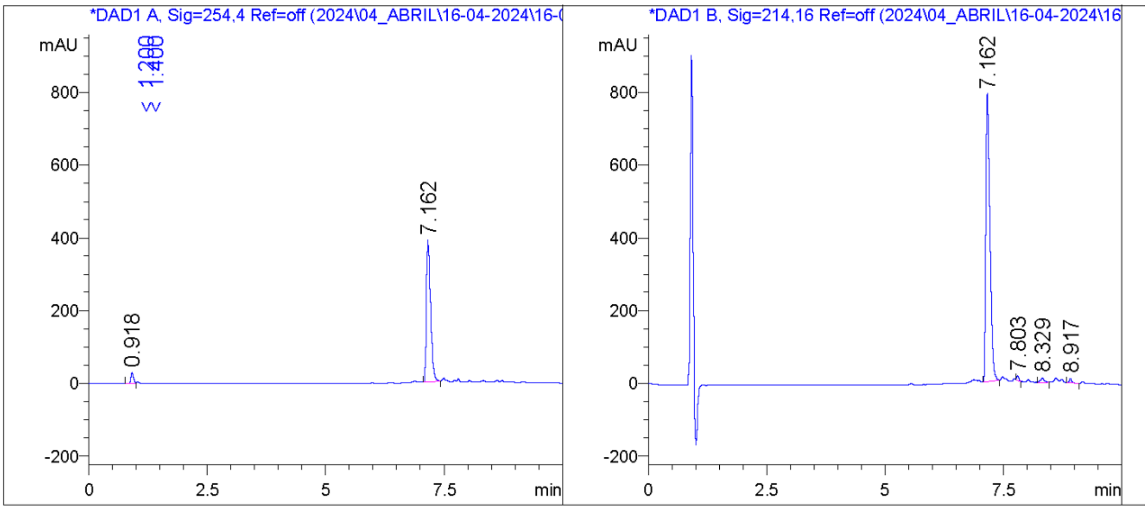

(*E*)-2-Hydroxy-5-[5-(3-phenyl-3-oxoprop-1-en-1-yl)furan-2-yl]benzoic acid (4).

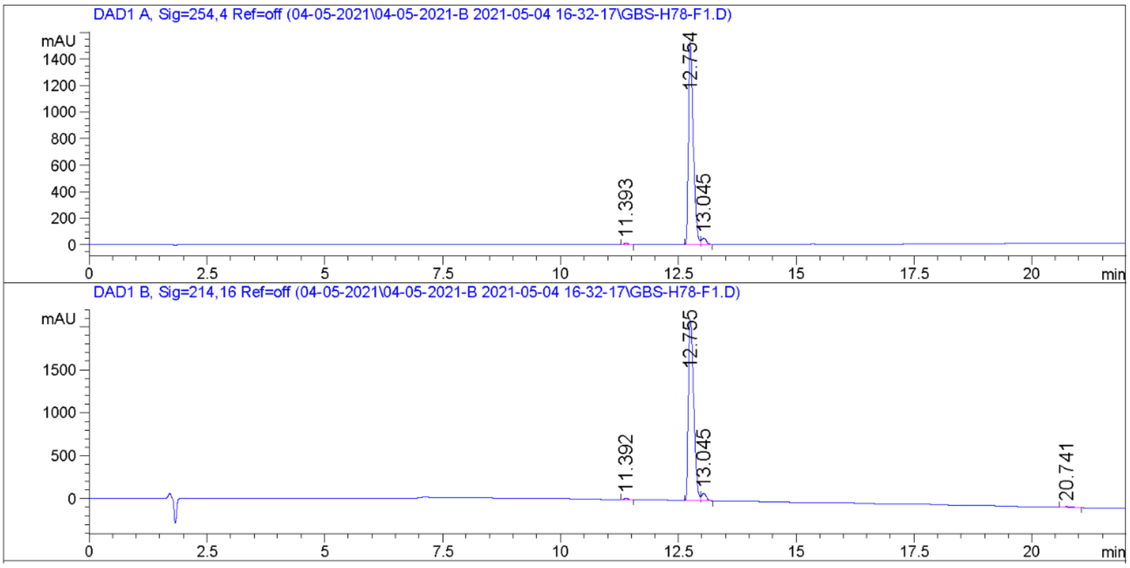

(*E*)-2-Hydroxy-5-{5-[3-(4-nitrophenyl)-3-oxoprop-1-en-1-yl]furan-2-yl}benzoic acid (5).

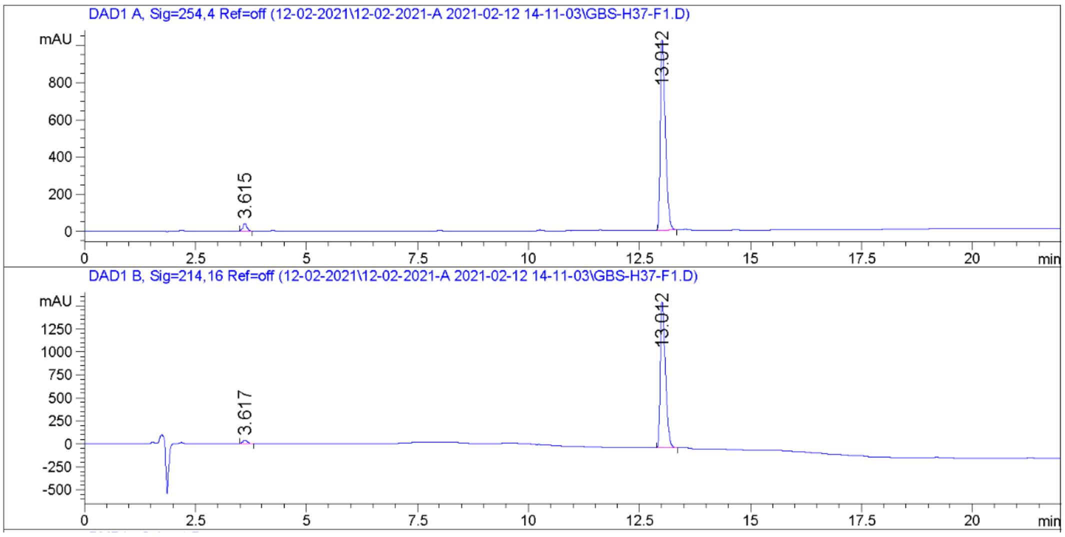

(*E*)-5-{5-[3-(4-Cyanophenyl)-3-oxoprop-1-en-1-yl]furan-2-yl}-2-hydroxybenzoic acid (**6**).

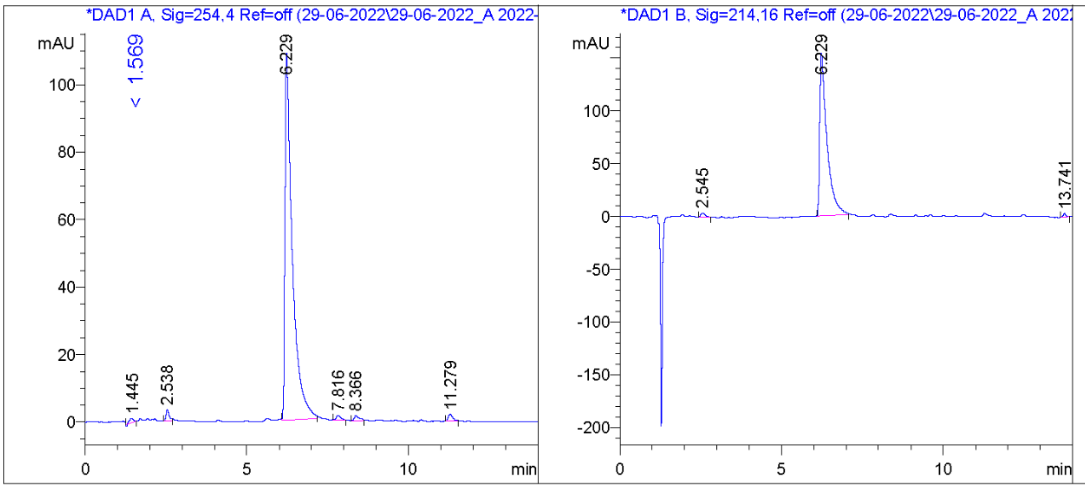

(*E*)-2-Hydroxy-5-{5-[3-(4-hydroxyphenyl)-3-oxoprop-1-en-1-yl]furan-2-yl}benzoic acid (7).

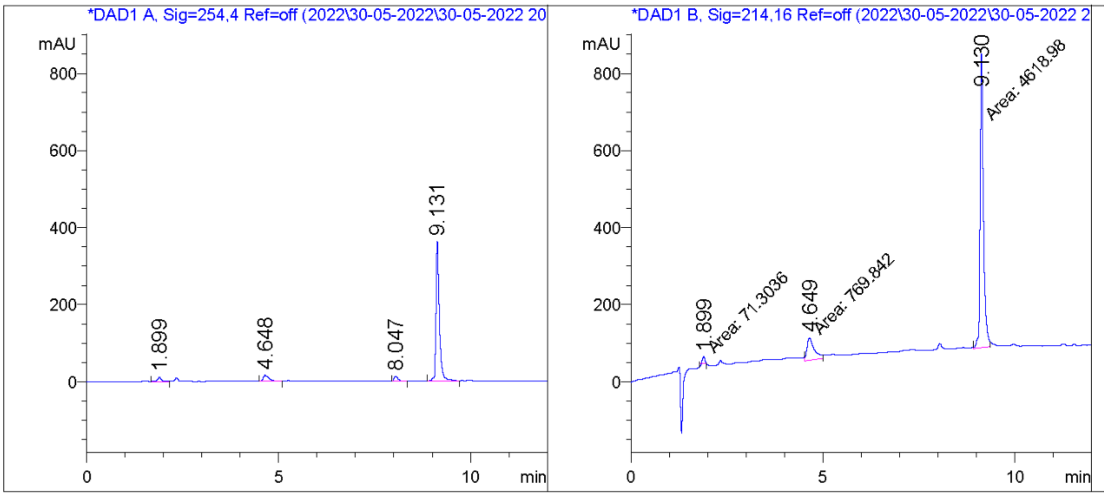

(*E*)-2-Hydroxy-5-{5-{3-[4-(hydroxymethyl)phenyl]-3-oxoprop-1-en-1-yl}furan-2-yl}benzoic acid (8).

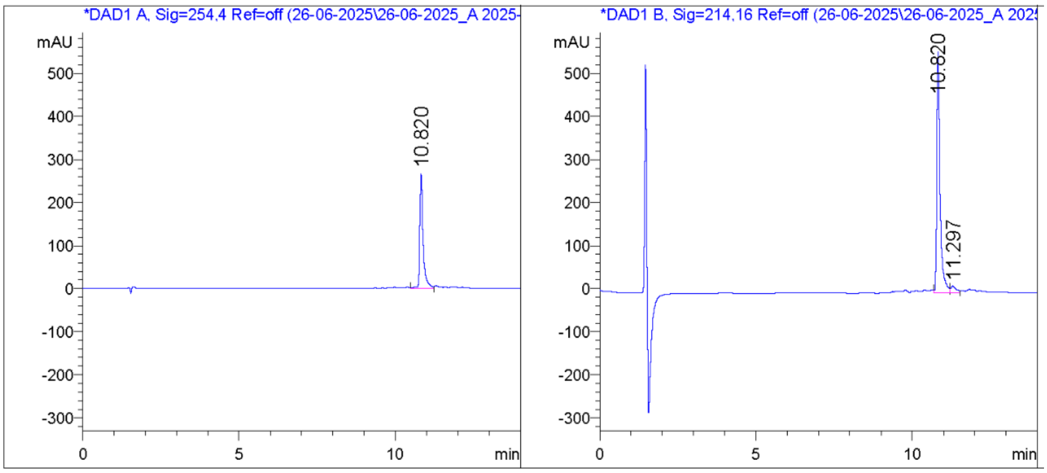

2-Hydroxy-5-{5-{(*E*)-3-[4-((*E*)-3-hydroxy-3-oxoprop-1-enyl)phenyl]-3-oxoprop-1-enyl}furan-2-yl}benzoic acid (9).

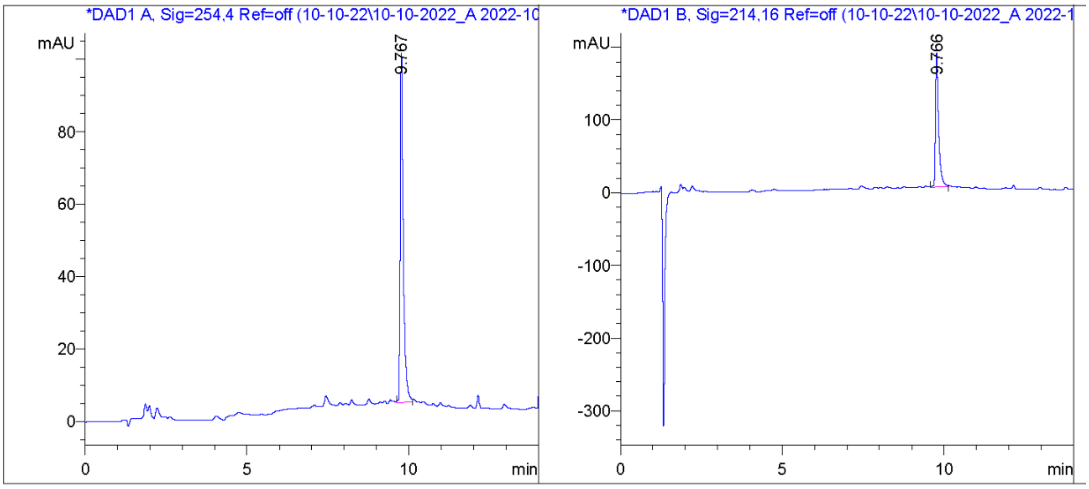

(*E*)-5-{5-{3-[4-(2-Carboxyethyl)phenyl]-3-oxoprop-1-en-1-yl}furan-2-yl}-2-hydroxybenzoic acid (10).

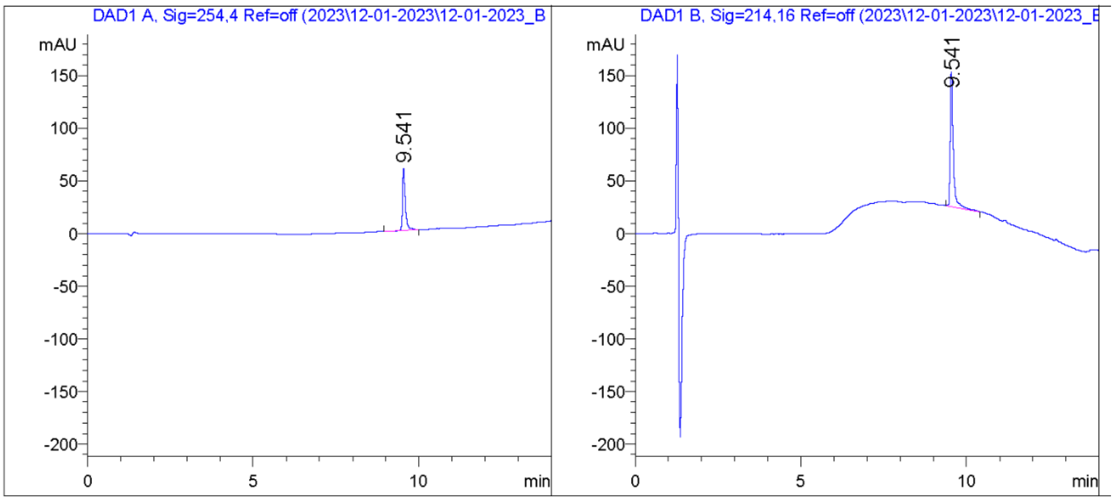

(*E*)-2-Hydroxy-5-{5-[3-(4-bromophenyl)-3-oxoprop-1-en-1-yl]furan-2-yl}benzoic acid (11).

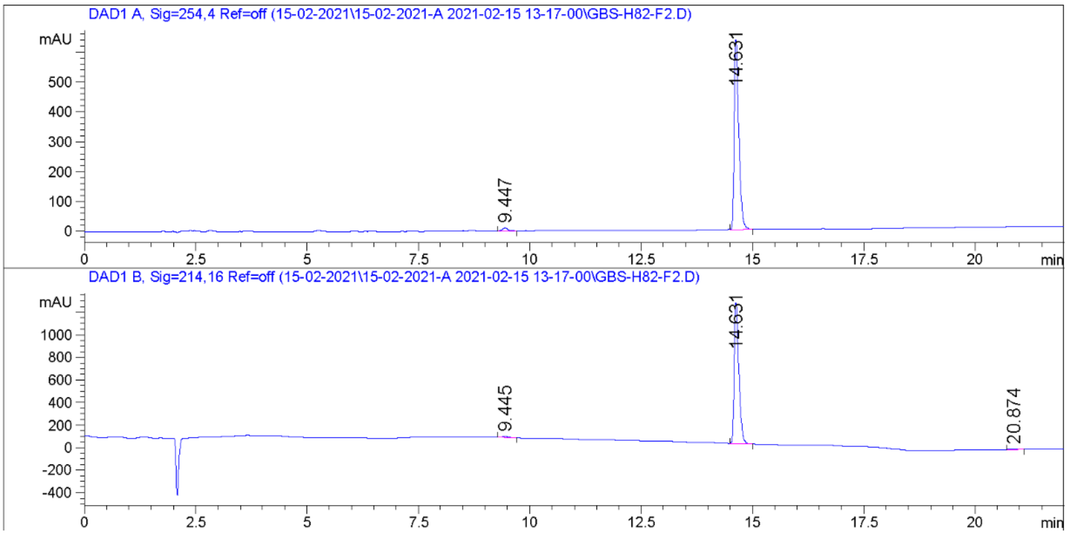

(*E*)-2-Hydroxy-5-{5-[3-(4-iodophenyl)-3-oxoprop-1-en-1-yl]furan-2-yl}benzoic acid (12).

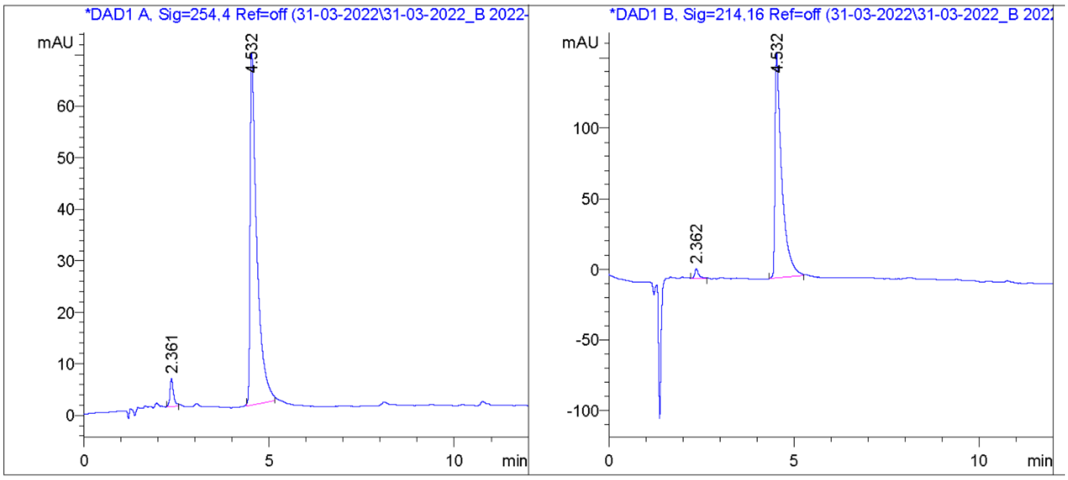

(*E*)-2-Hydroxy-5-{5-[3-(4-methoxyphenyl)-3-oxoprop-1-en-1-yl]-2-furanyl}benzoic acid (13).

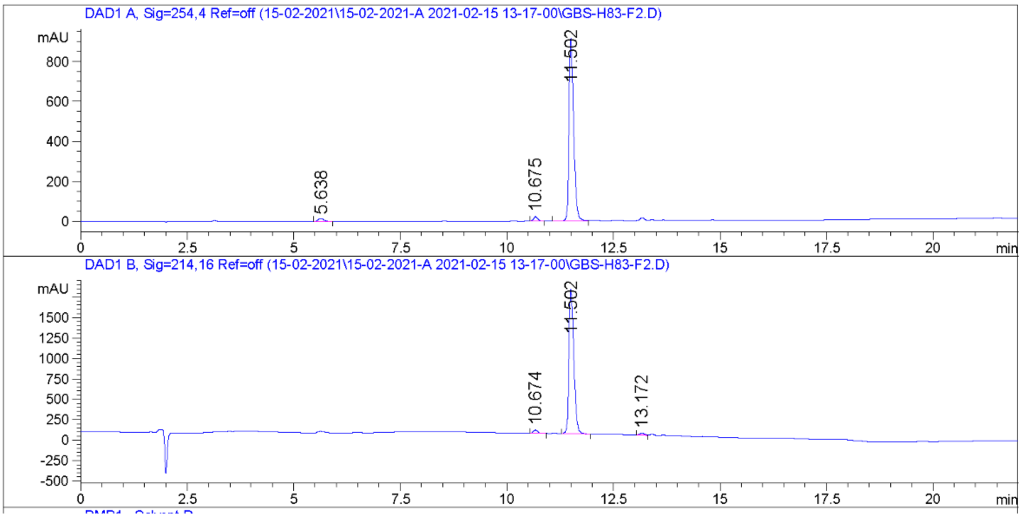

(*E*)-2-Hydroxy-5-{5-{3-(4-(methylthio)phenyl)-3-oxoprop-1-en-1-yl}furan-2-yl}benzoic acid (14)

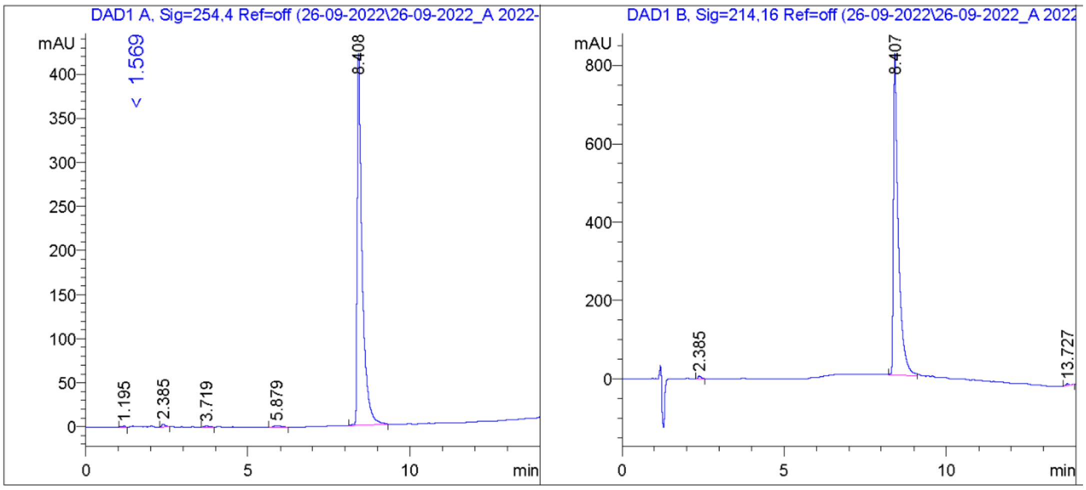

(*E*)-5-{5-{3-[4-(*N,N*-dimethylamino)phenyl]-3-oxoprop-1-en-1-yl}furan-2-yl}-2-hydroxybenzoic acid (15)

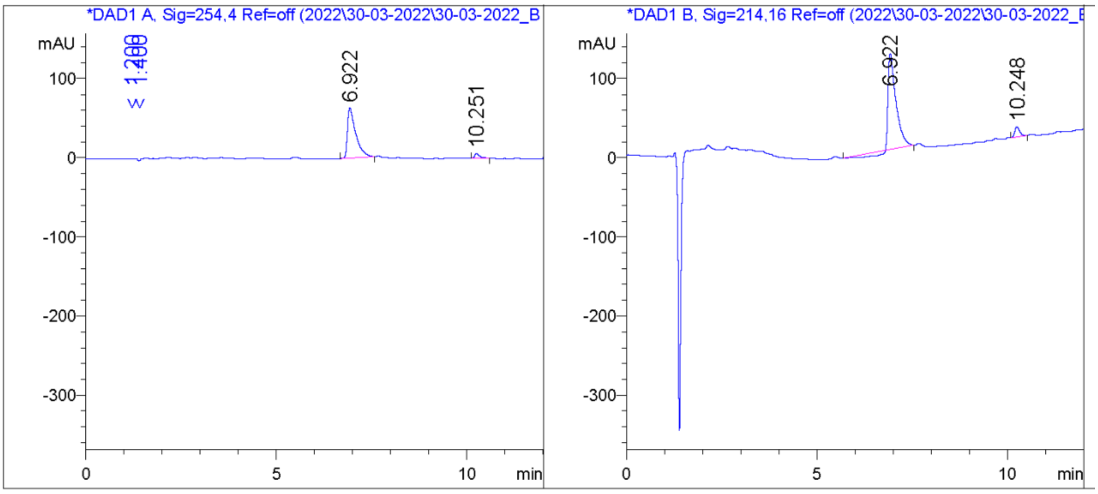

(*E*)-2-Hydroxy-5-{5-[3-oxo-3-(*p*-tolyl)prop-1-en-1-yl]furan-2-yl}benzoic acid (16)

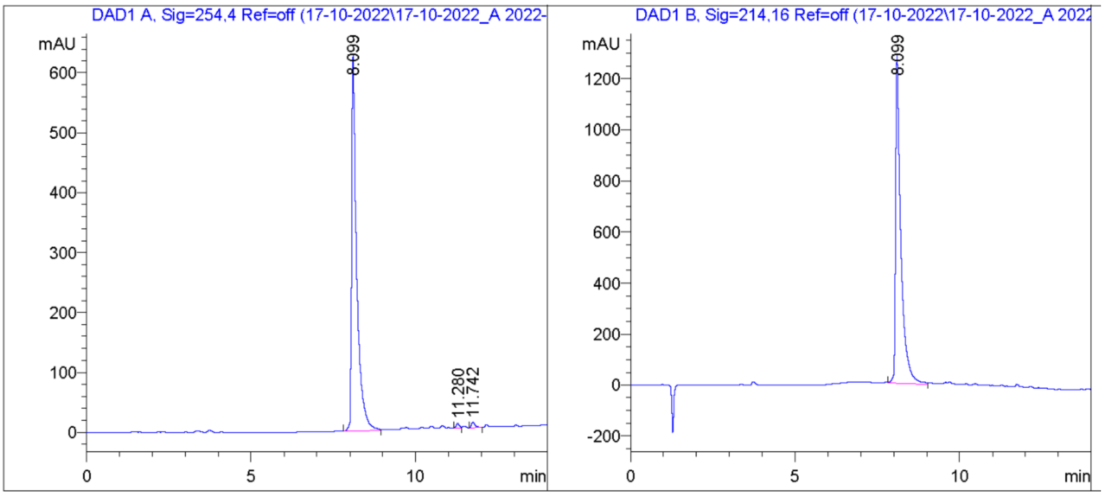

(*E*)-2-Hydroxy-5-{5-{3-[4-(trifluoromethyl)phenyl]-3-oxoprop-1-en-1-yl}furan-2-yl}-benzoic acid (17)

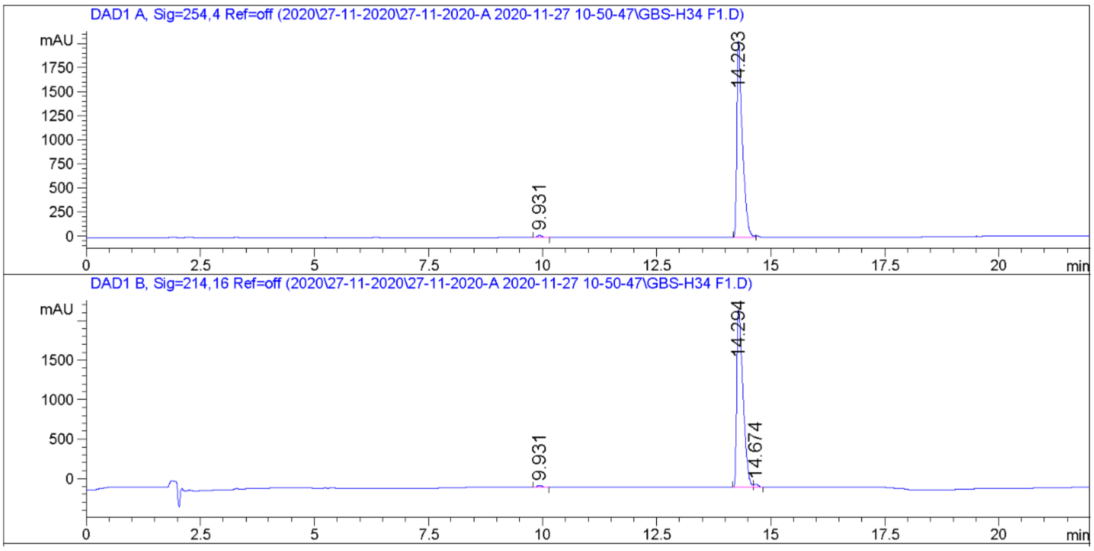

(*E*)-5-{5-[3-(4-Ethylphenyl)-3-oxoprop-1-en-1-yl]furan-2-yl}-2-hydroxybenzoic acid (18)

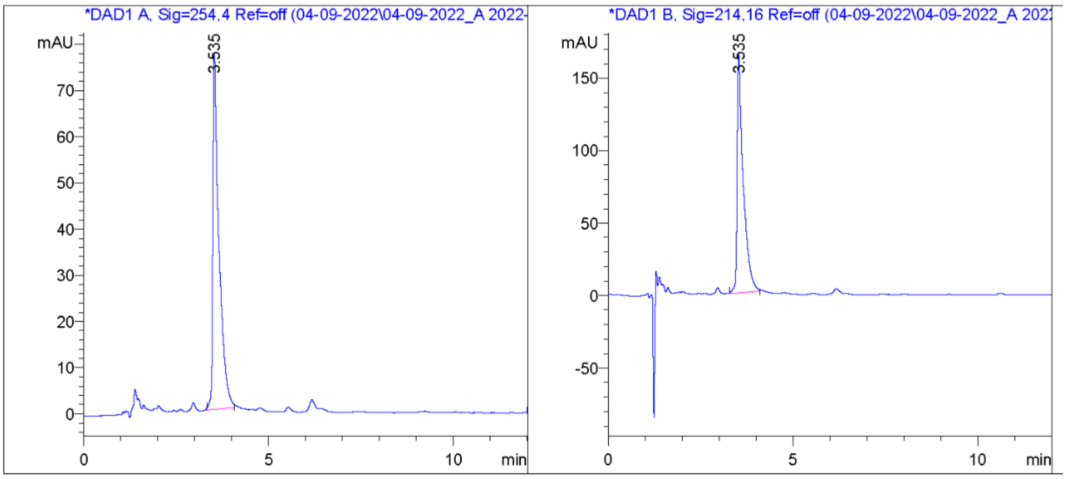

(*E*)-2-Hydroxy-5-{5-[3-(4-isopropylphenyl)-3-oxoprop-1-en-1-yl]furan-2-yl}benzoic acid (19)

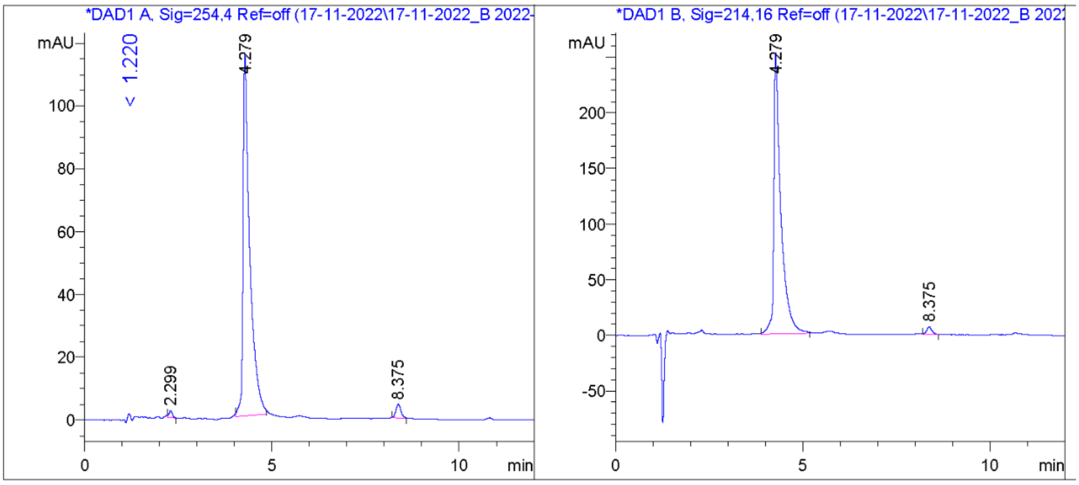

(*E*)-5-{5-[3-(4-Butylphenyl)-3-oxoprop-1-en-1-yl]furan-2-yl}-2-hydroxybenzoic acid (20)

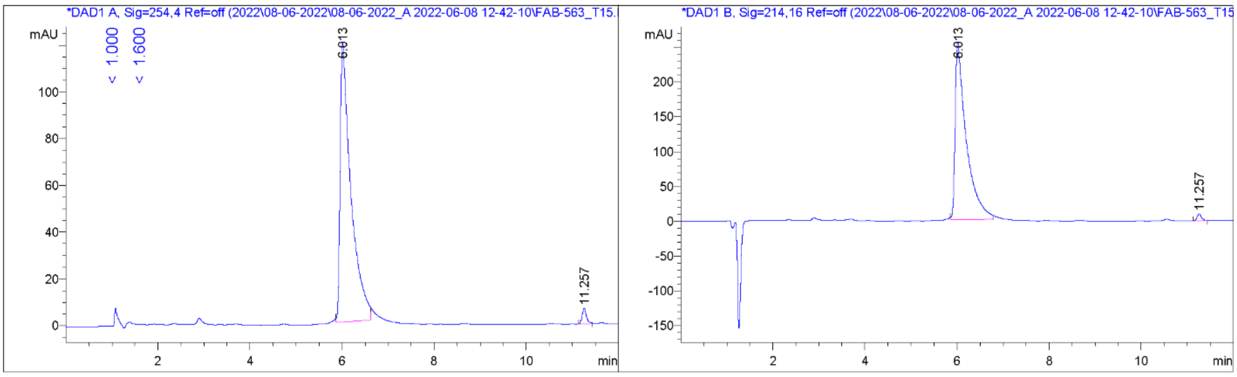

(*E*)-5-{5-[3-(4-Hexylphenyl)-3-oxoprop-1-en-1-yl]furan-2-yl}-2-hydroxybenzoic acid (21)

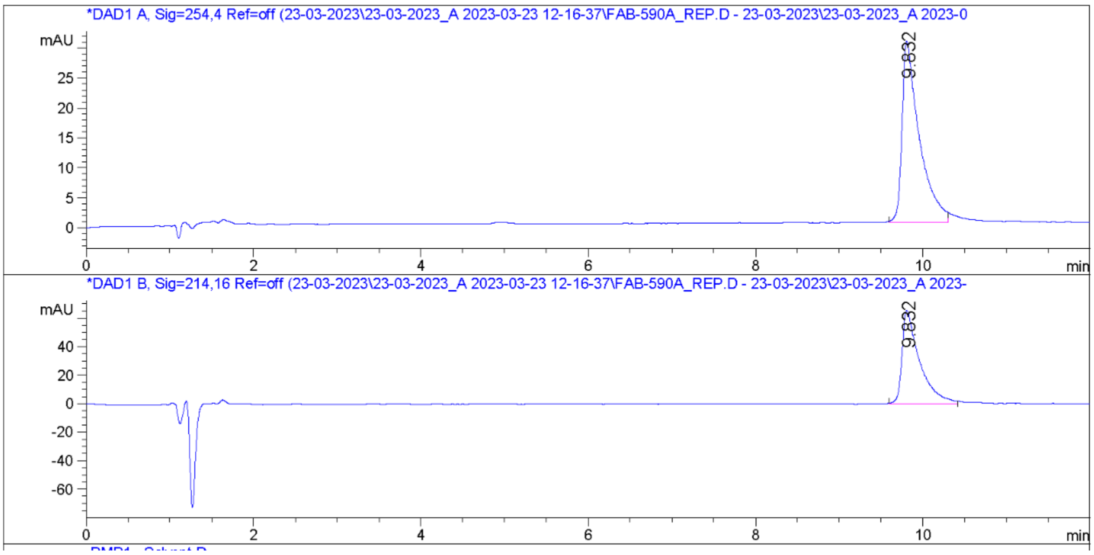

(*E*)-2-Hydroxy-5-{5-[3-(4-octylphenyl)-3-oxoprop-1-en-1-yl]furan-2-yl}benzoic acid (22)

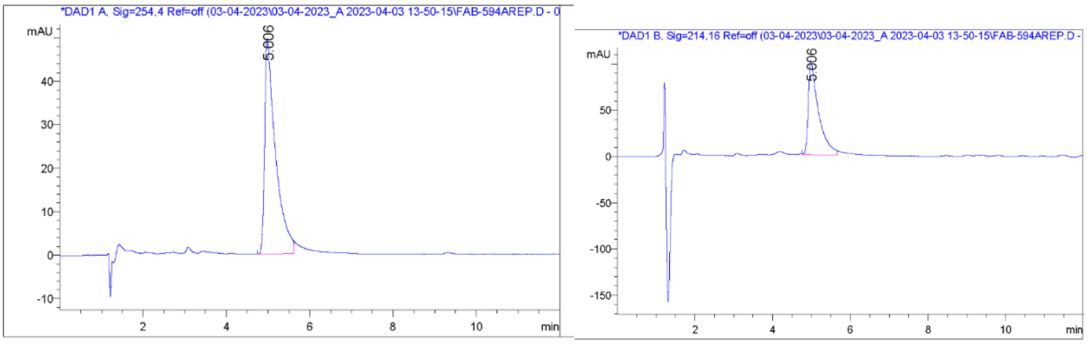

(*E*)-5-{5-{3-[4-(6-Chlorohex-1-yn-1-yl)phenyl]-3-oxoprop-1-en-1-yl}furan-2-yl}-2-hydroxybenzoic acid (23)

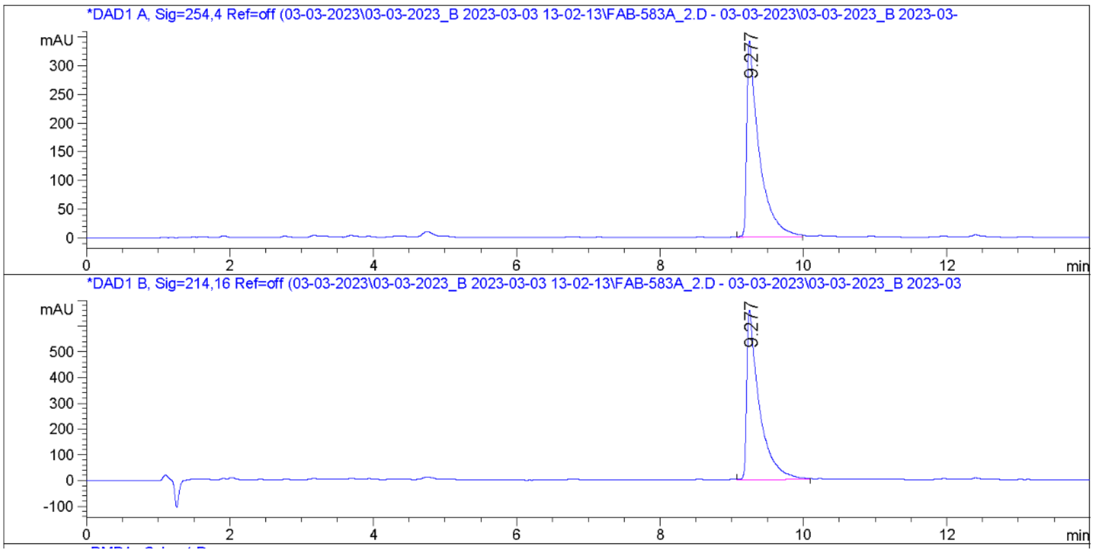

5-{5-{(*E*)-3-{4-[(*E*)-3-(*tert*-Butoxy)-3-oxoprop-1-en-1-yl]phenyl}-3-oxoprop-1-en-1-yl}furan-2-yl}-2-hydroxybenzoic acid (24)

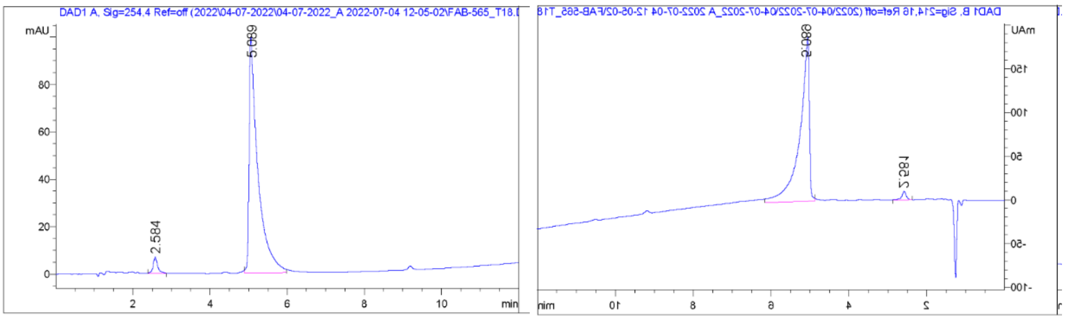

(*E*)-5-{5-{3-{4-[3-(*tert*-Butoxy)-3-oxopropyl]phenyl}-3-oxoprop-1-en-1-yl}furan-2-yl}-2-hydroxybenzoic acid (25)

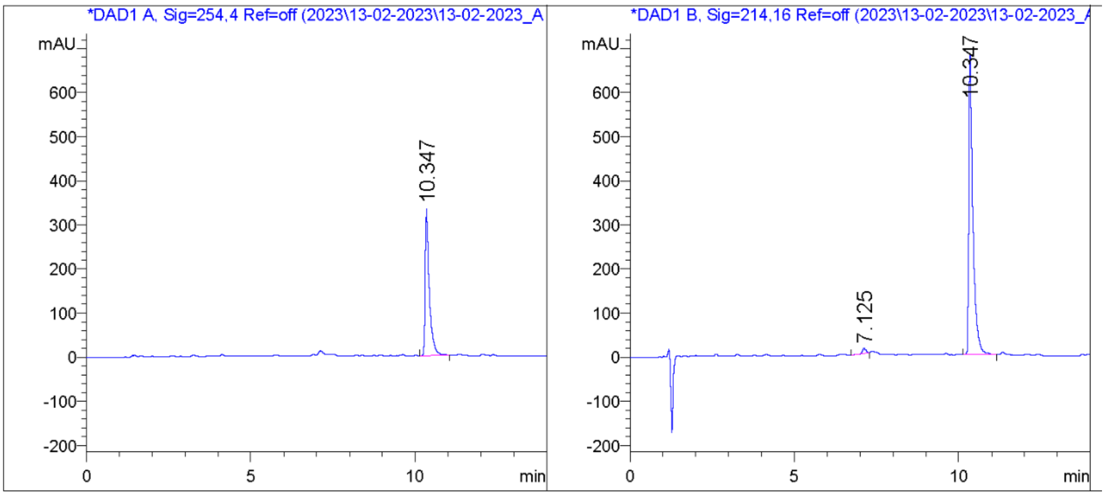

5-{5-{(*E*)-3-{4-[(*E*)-3-(adamantan-1-ylmethylamino)-3-oxoprop-1-enyl]phenyl}-3-oxoprop-1-enyl}furan-2-yl}-2-hydroxybenzoic acid (26)

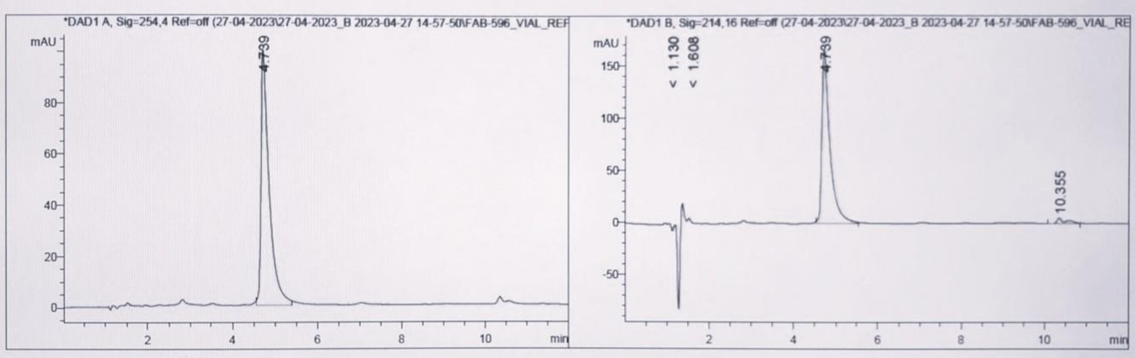

2-Hydroxy-5-{5-{3-oxo-3-[4-(trifluoromethyl)phenyl]propyl}furan-2-yl}benzoic acid (27)

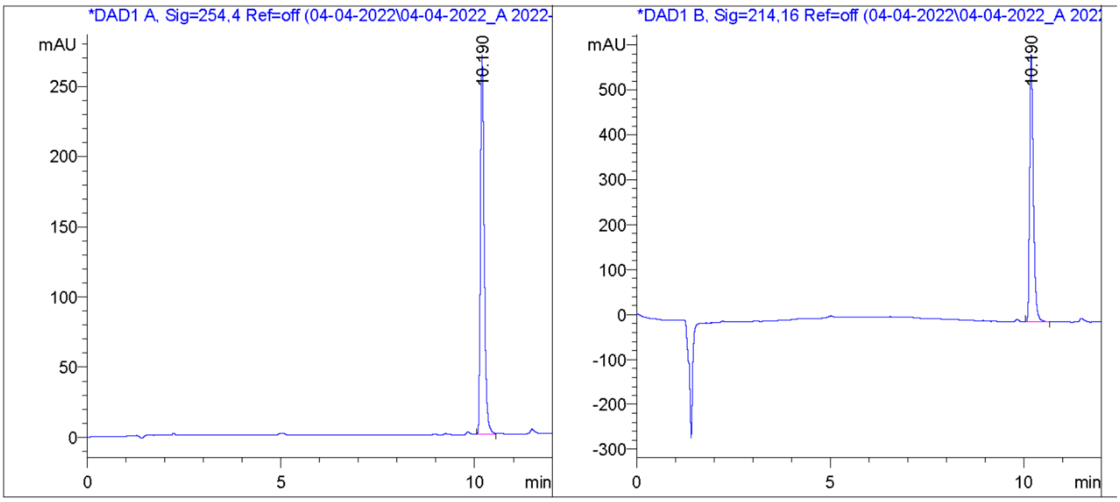

5-{5-[3-(4-Butylphenyl)-3-hydroxypropyl]furan-2-yl}-2-hydroxybenzoic acid (29)

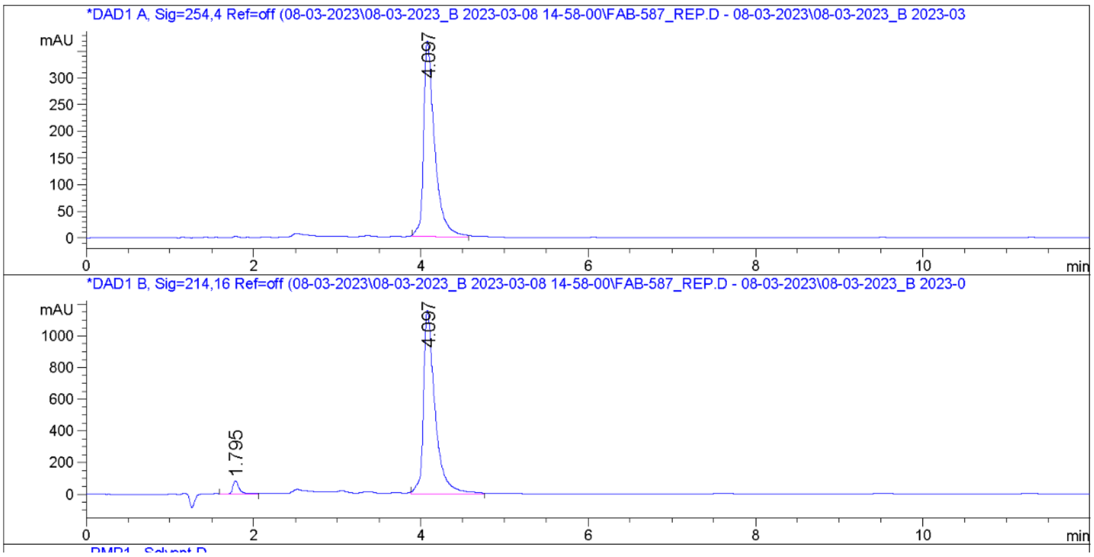

(*E*)-2-Hydroxy-5-[5-(3-oxobut-1-en-1-yl)furan-2-yl]benzoic acid (S1).

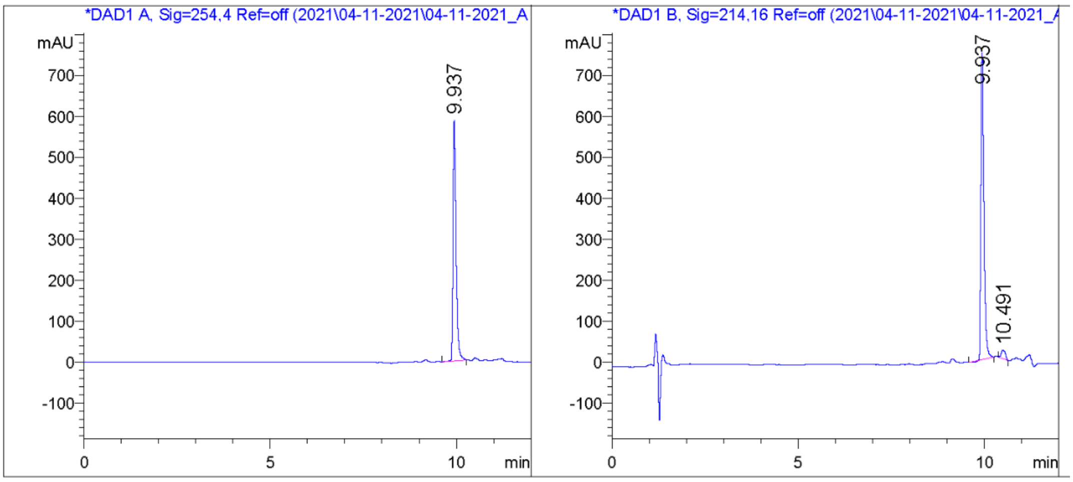

(*E*)-2-Hydroxy-5-[5-(4,4,4-trifluoro-3-oxobut-1-enyl)furan-2-yl]benzoic acid (S2)

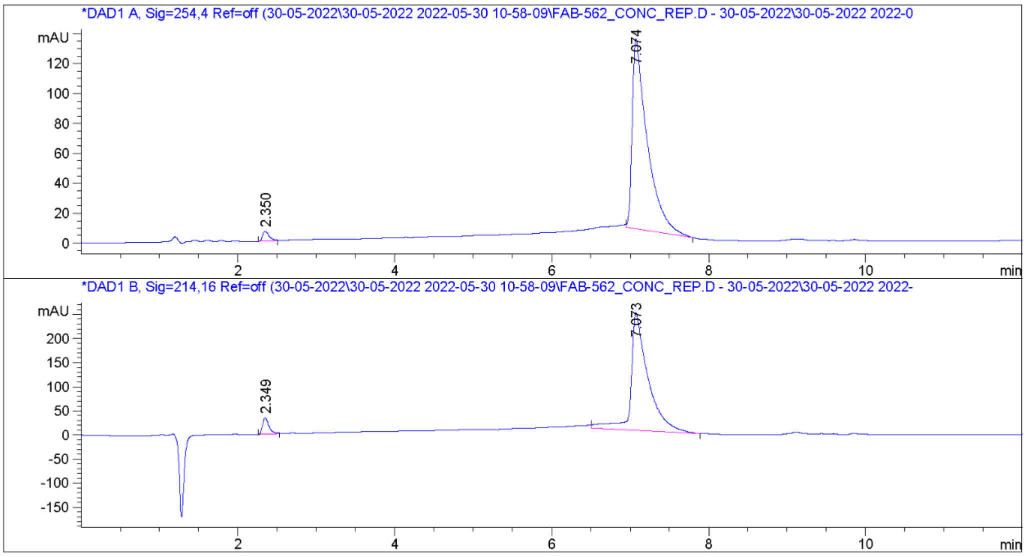

5-[5-(2-Acetyl-3-oxobut-1-enyl)furan-2-yl]-2-hydroxybenzoic acid (S3).

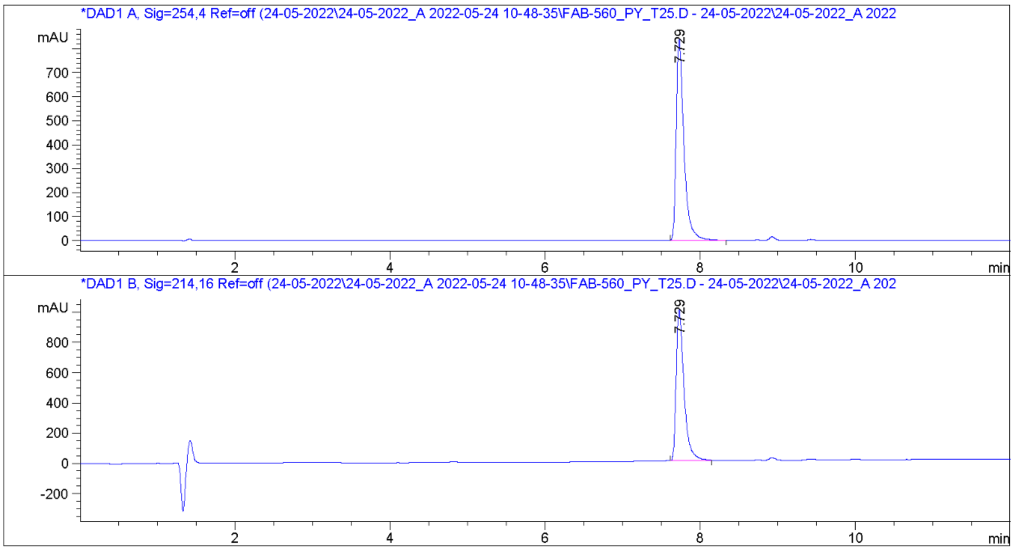

(*E*)-2-Hydroxy-5-{5-[3-(1-methylpyrrol-2-yl)-3-oxoprop-1-en-1-yl]furan-2-yl}benzoic acid (S4).

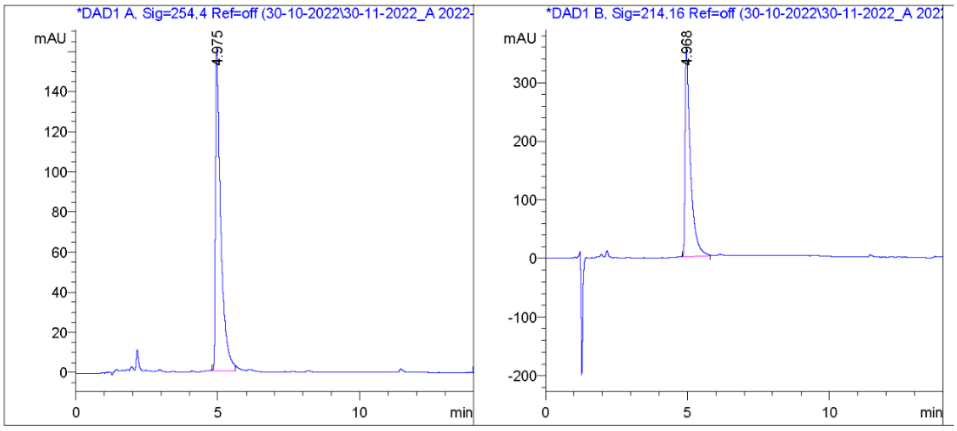

(*E*)-5-{5-[3-(furan-2-yl)-3-oxoprop-1-enyl]furan-2-yl}-2-hydroxybenzoic acid (S5).

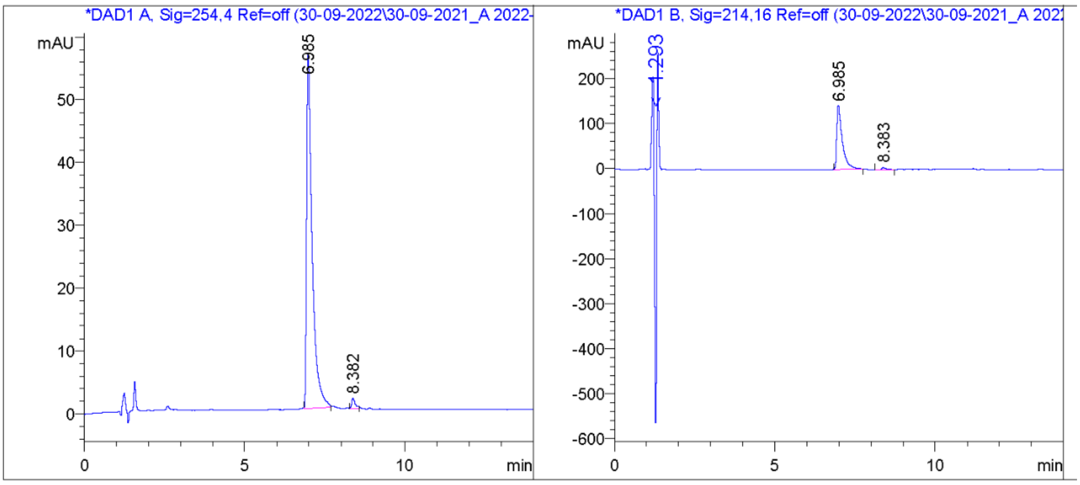

2-Hydroxy-5-{5-[3-hydroxy-3-(*p*-trifluoromethylphenyl)propyl]tetrahydrofuran-2-yl}benzoic acid (S6).

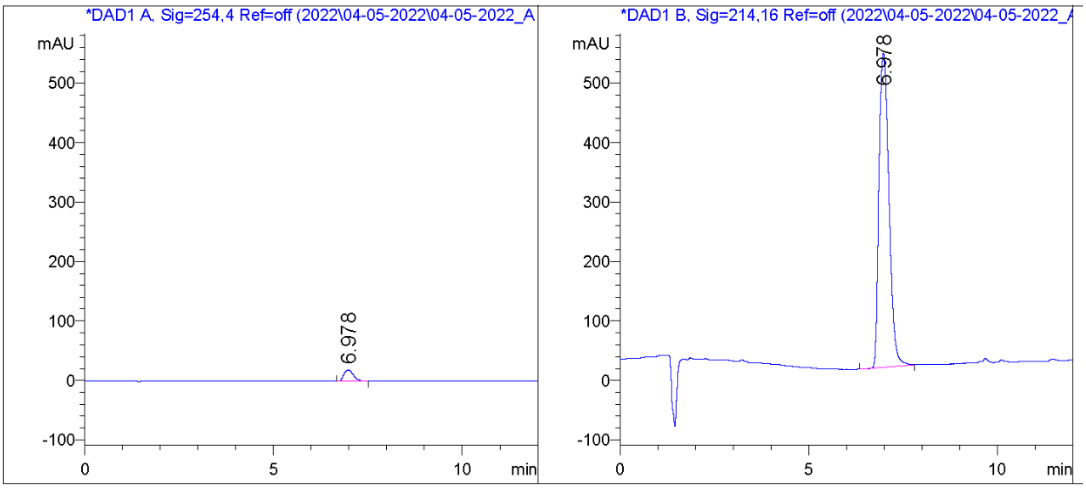

S.18. NMR SPECTRA

5-{5-{(*E*)-3-{4-[6-(adamantan-1-ylmethylamino)hex-1-ynyl]phenyl}-3-oxoprop-1-enyl}furan-2-yl}-2-hydroxybenzoic acid (2).

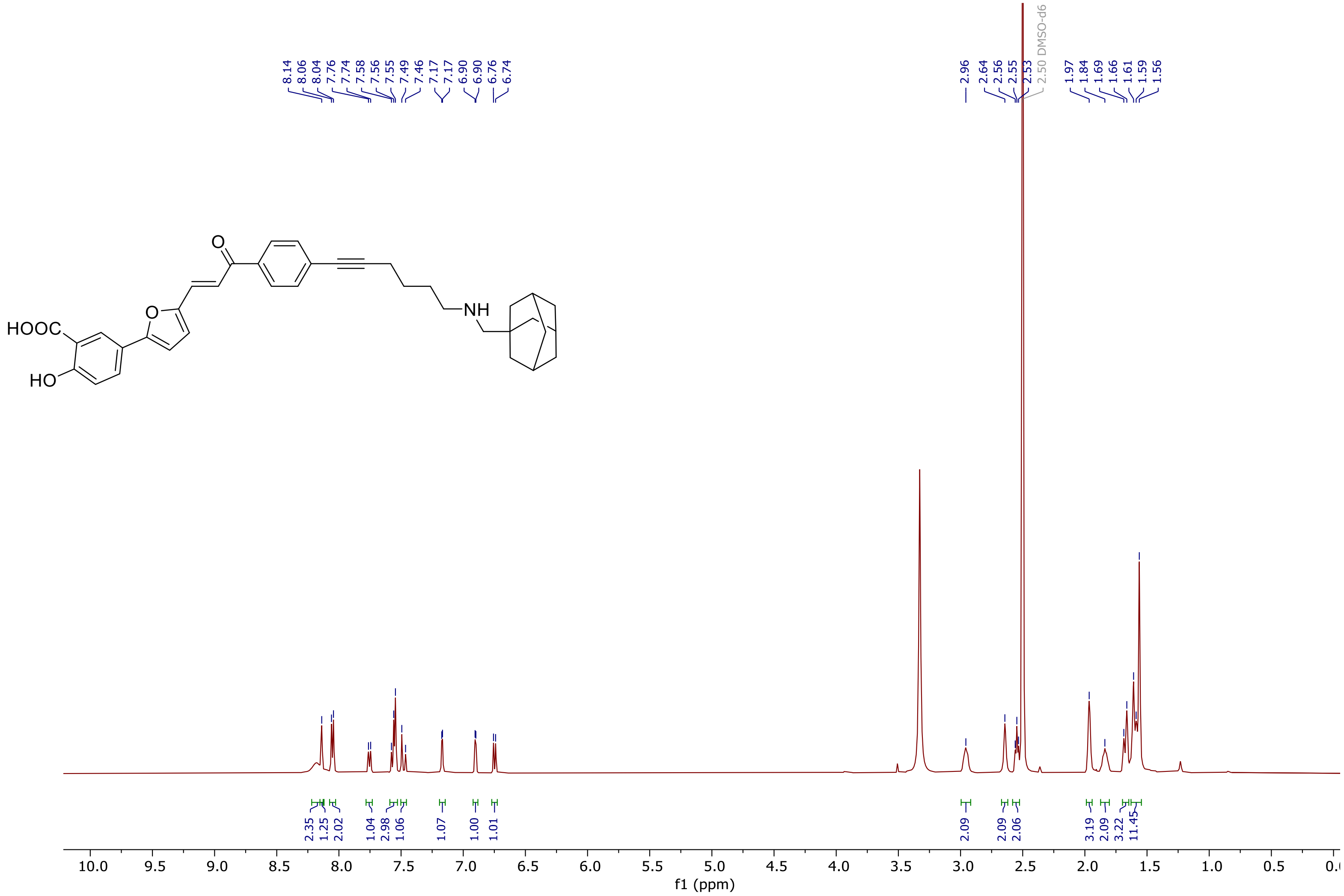

5-{5-{(*E*)-3-{4-[6-(adamantan-1-ylmethylamino)hex-1-ynyl]phenyl}-3-oxoprop-1-enyl}furan-2-yl}-2-hydroxybenzoic acid (2).

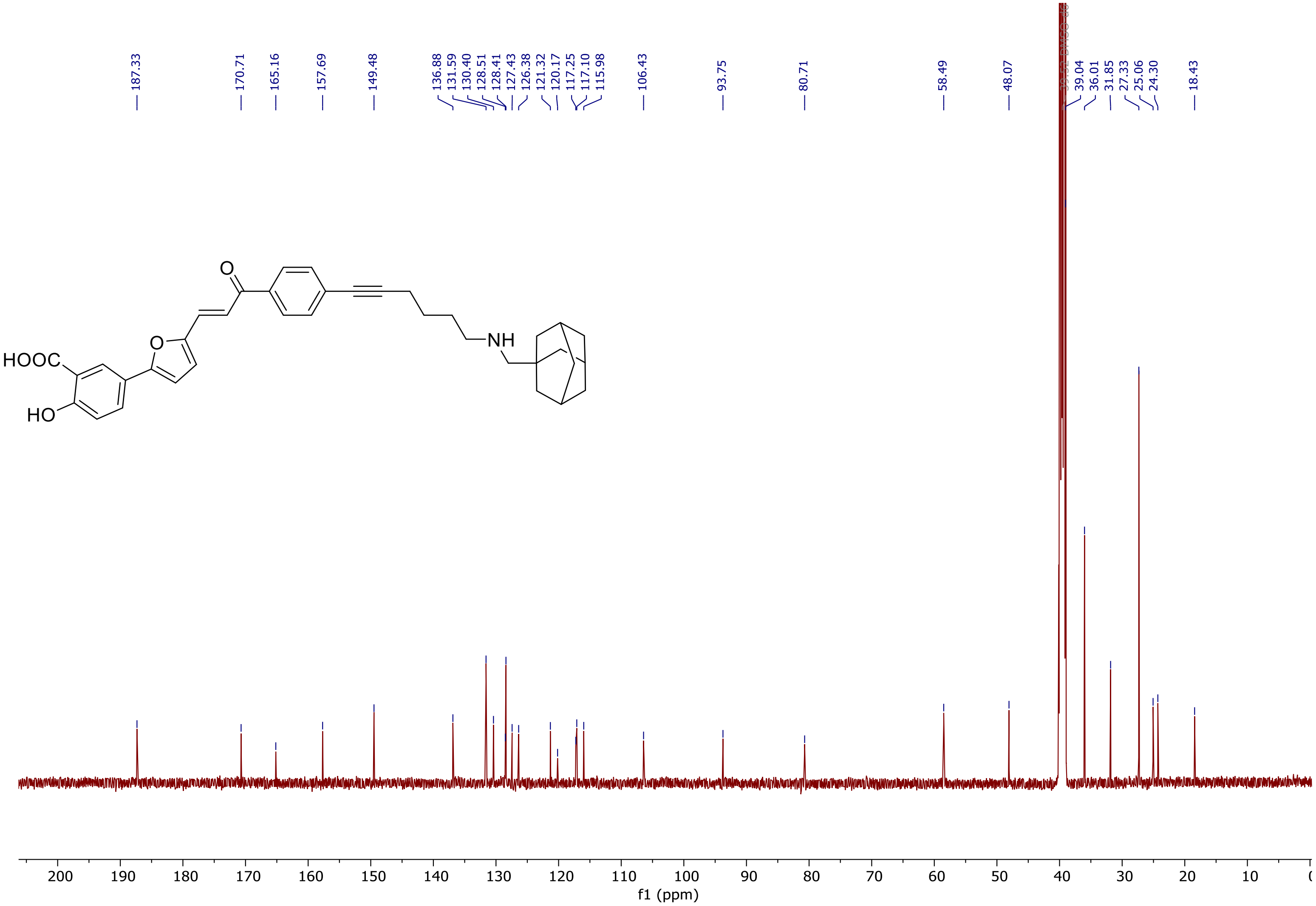

(*E*)-2-Hydroxy-5-[5-(3-phenyl-3-oxoprop-1-en-1-yl)furan-2-yl]benzoic acid (4).

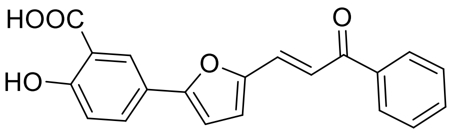

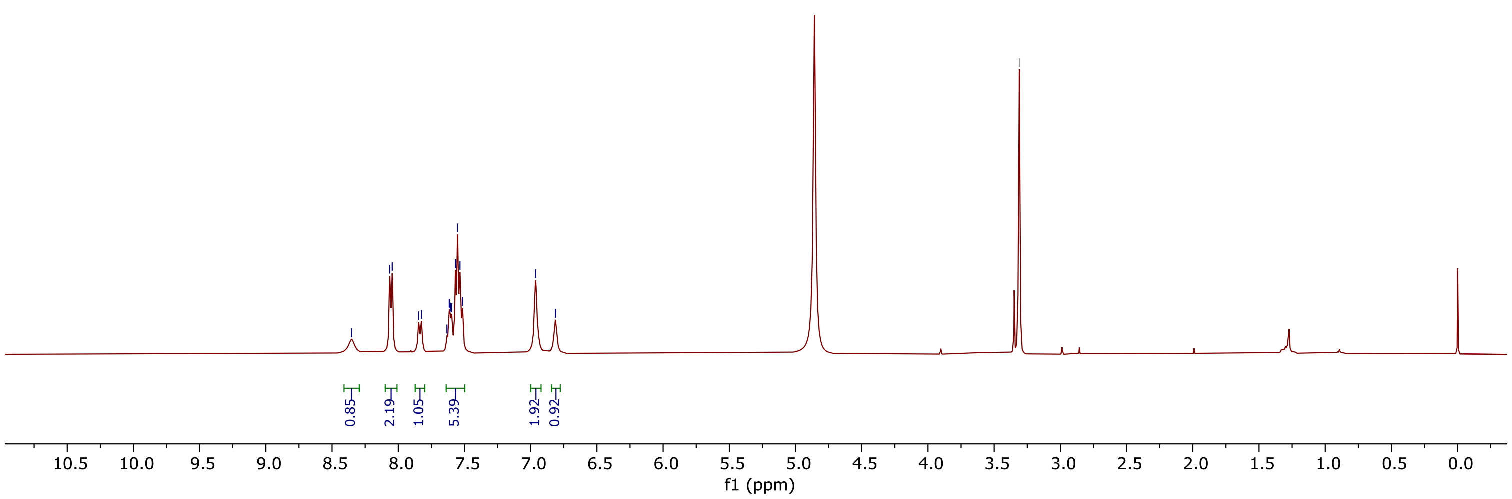

(*E*)-2-Hydroxy-5-[5-(3-phenyl-3-oxoprop-1-en-1-yl)furan-2-yl]benzoic acid (4).

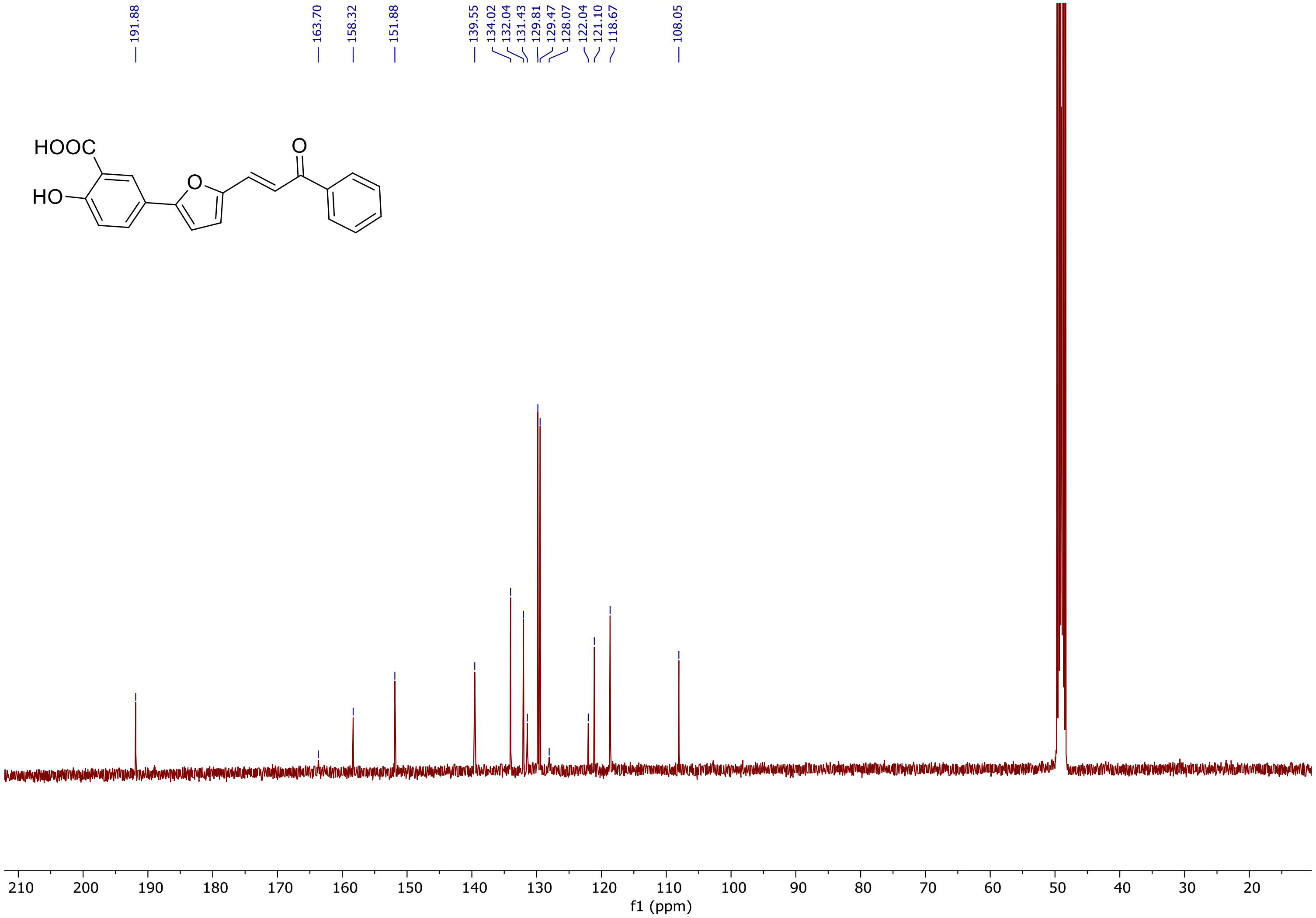

(*E*)-2-Hydroxy-5-{5-[3-(4-nitrophenyl)-3-oxoprop-1-en-1-yl]furan-2-yl}benzoic acid (5)

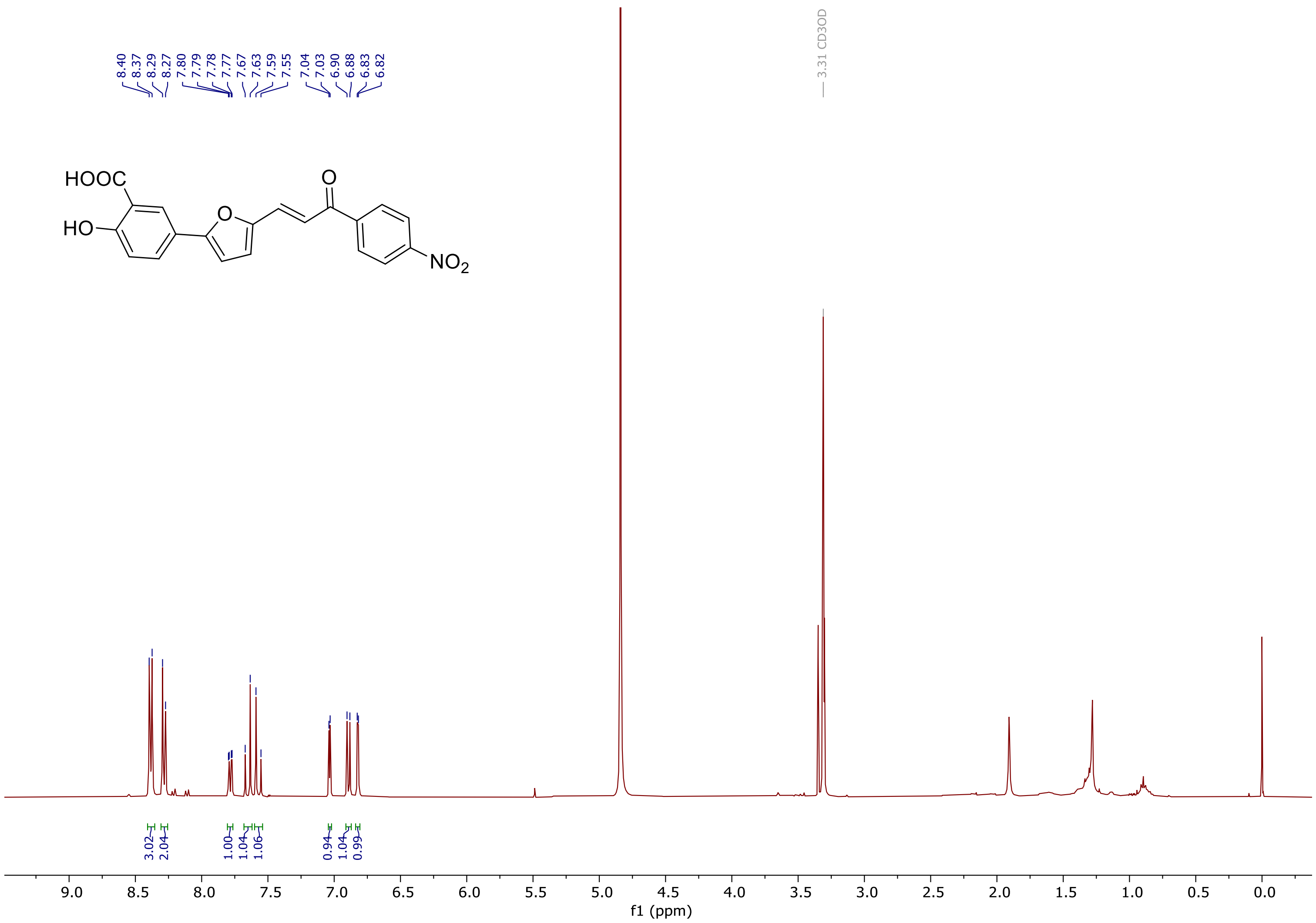

(*E*)-2-Hydroxy-5-{5-[3-(4-nitrophenyl)-3-oxoprop-1-en-1-yl]furan-2-yl}benzoic acid (5)

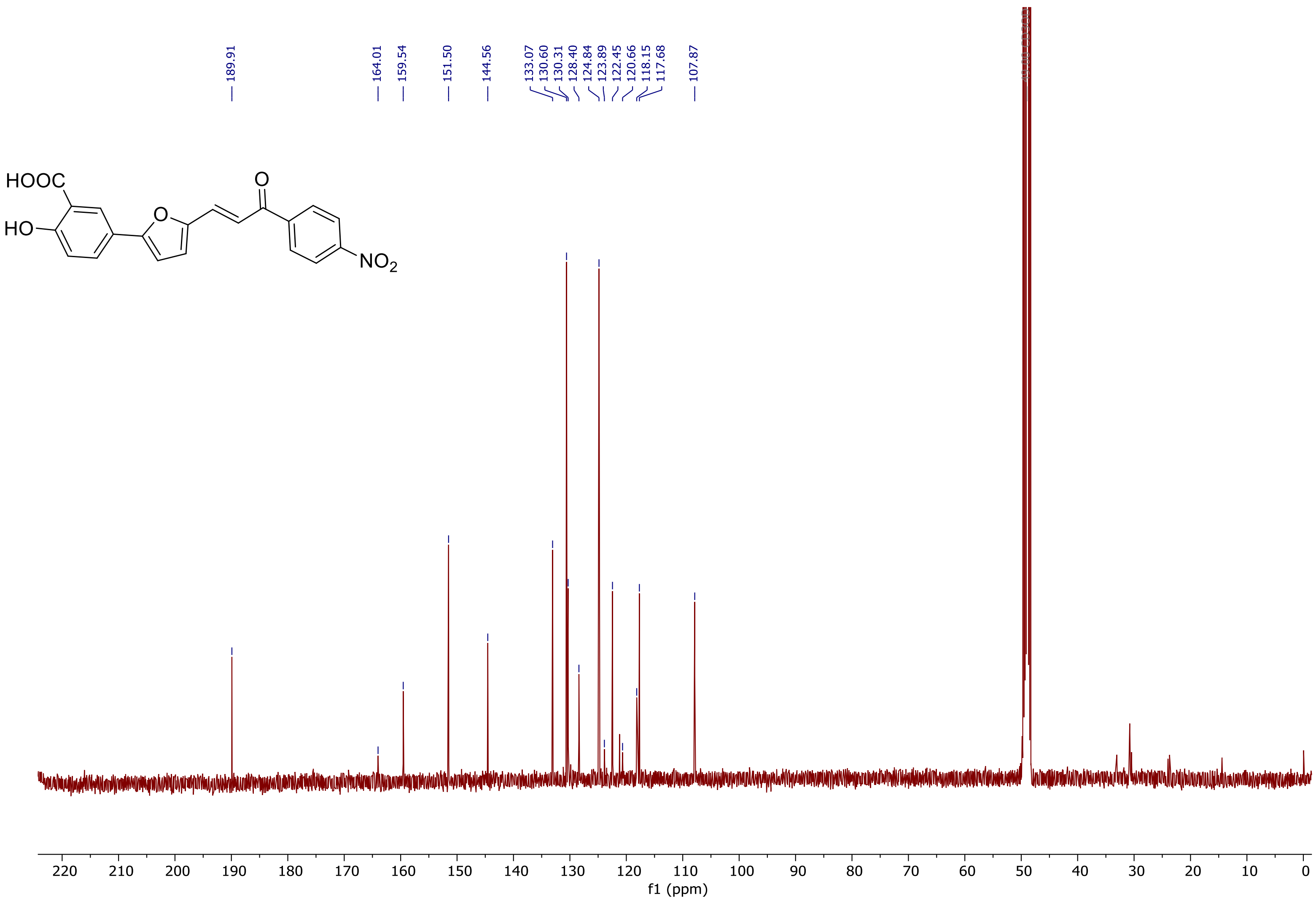

(*E*)-5-{5-[3-(4-Cyanophenyl)-3-oxoprop-1-en-1-yl]furan-2-yl}-2-hydroxybenzoic acid (**6**).

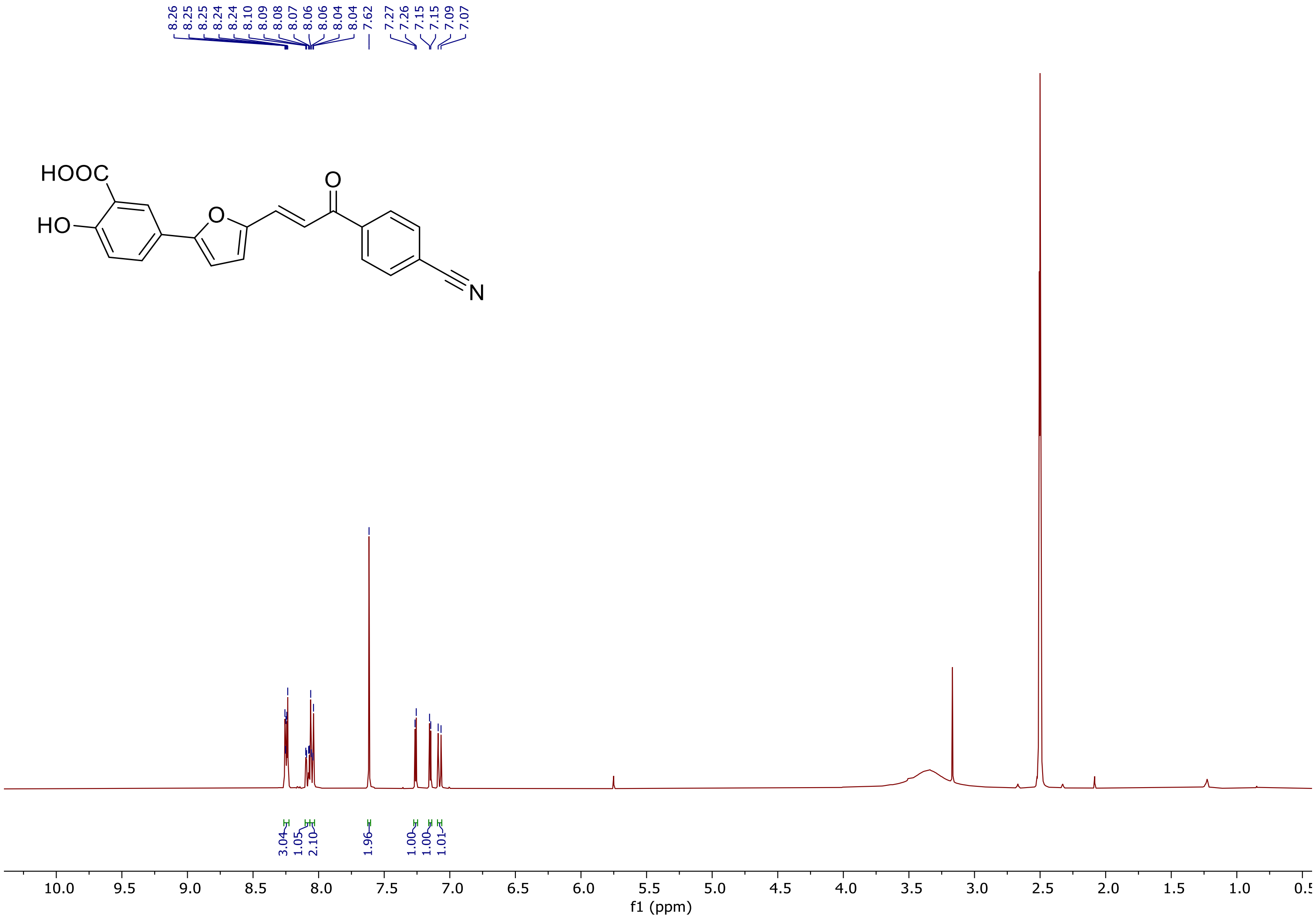

(*E*)-5-{5-[3-(4-Cyanophenyl)-3-oxoprop-1-en-1-yl]furan-2-yl}-2-hydroxybenzoic acid (**6**).

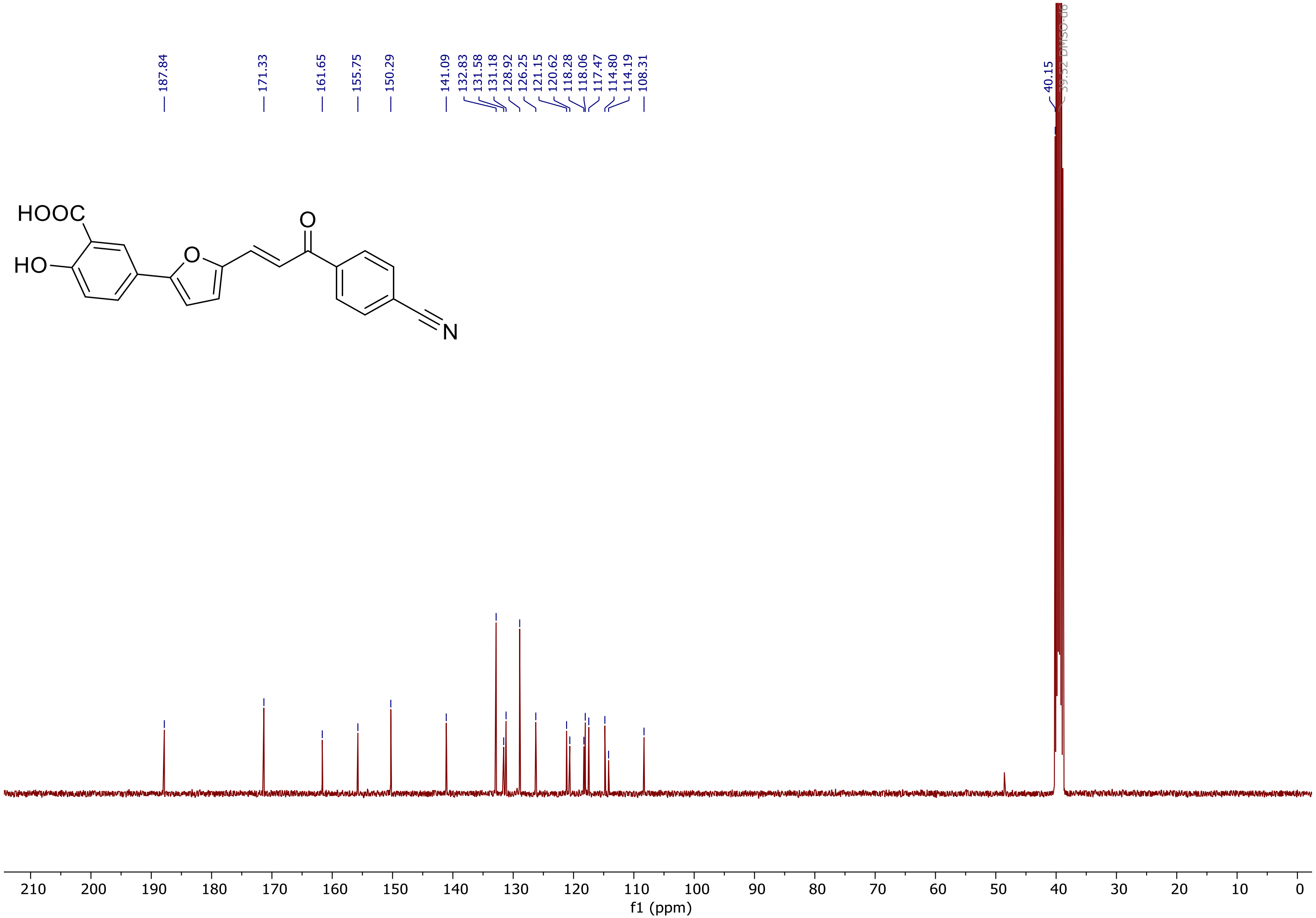

(*E*)-2-Hydroxy-5-{5-[3-(4-hydroxyphenyl)-3-oxoprop-1-en-1-yl]furan-2-yl}benzoic acid (7).

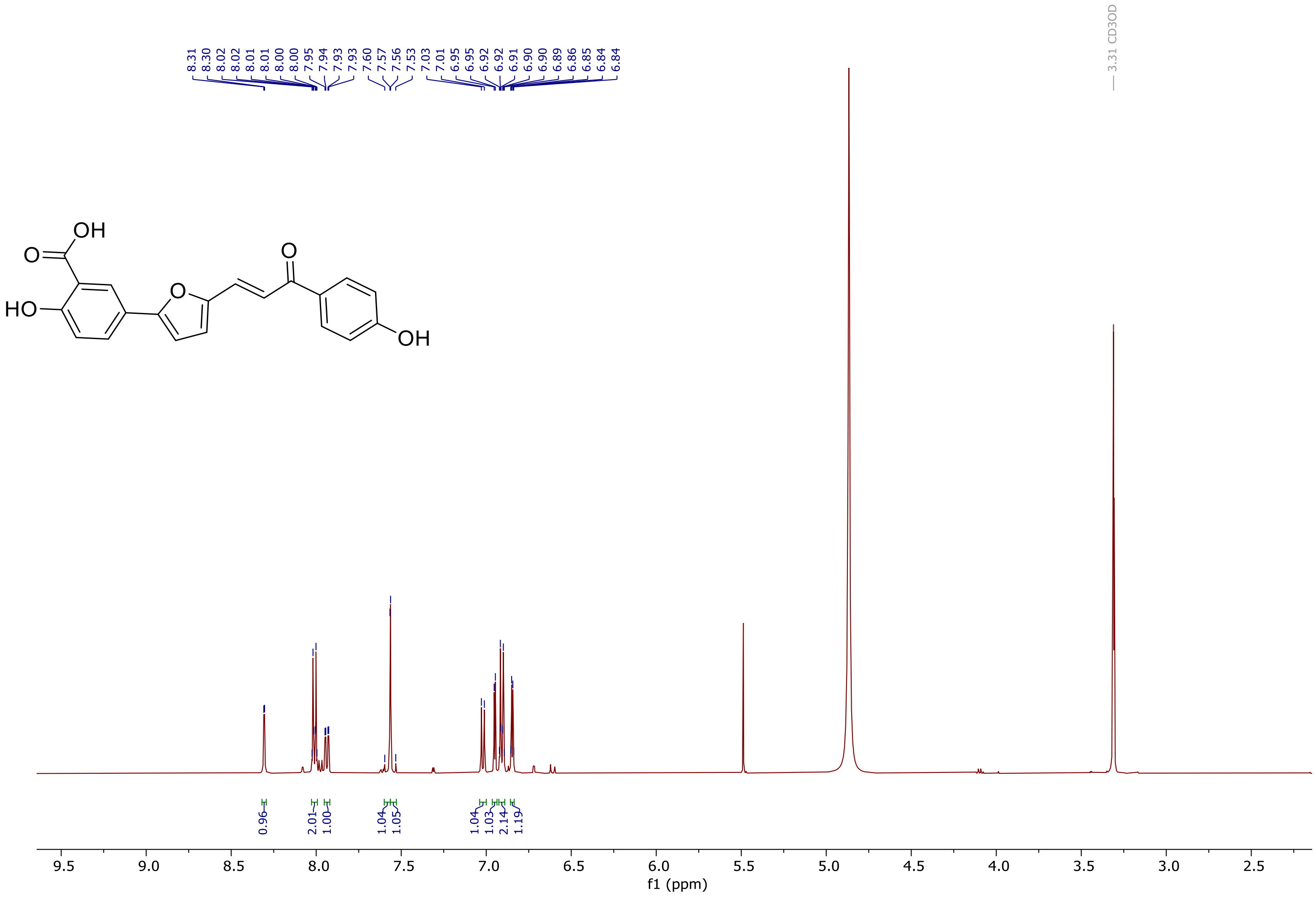

(*E*)-2-Hydroxy-5-{5-[3-(4-hydroxyphenyl)-3-oxoprop-1-en-1-yl]furan-2-yl}benzoic acid (7).

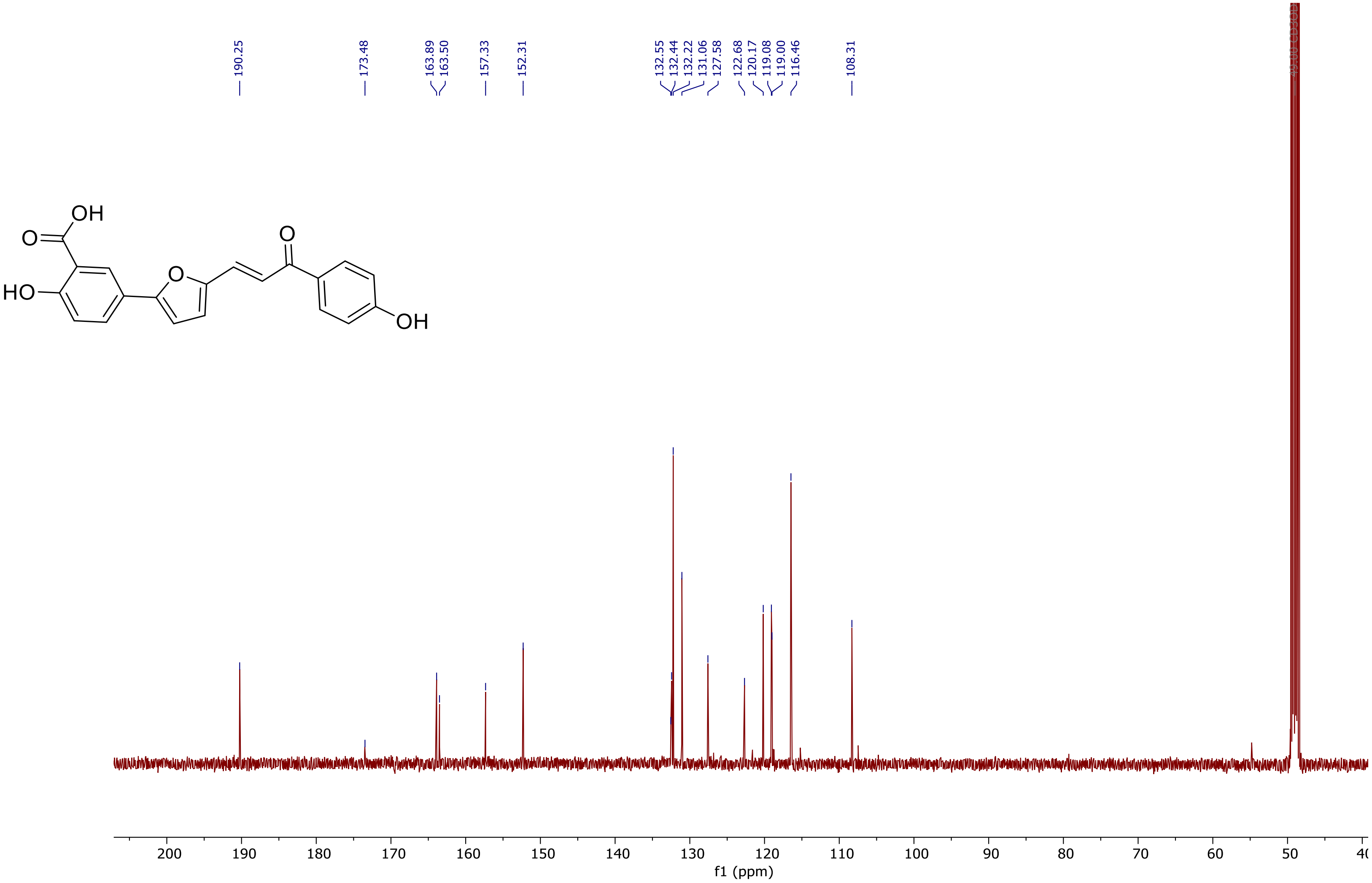

(*E*)-2-Hydroxy-5-{5-{3-[4-(hydroxymethyl)phenyl]-3-oxoprop-1-en-1-yl}furan-2-yl}benzoic acid (8).

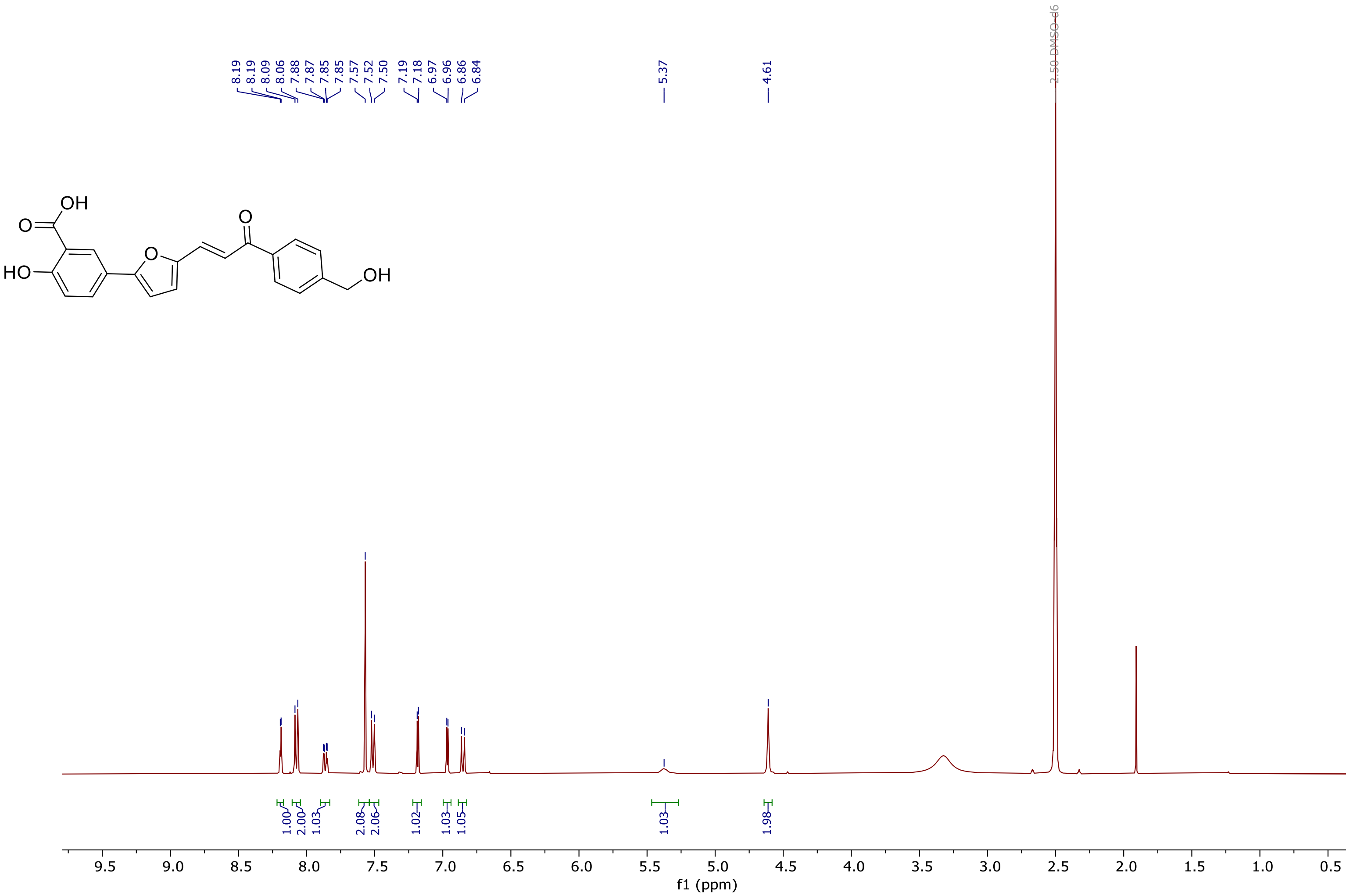

(*E*)-2-Hydroxy-5-{5-{3-[4-(hydroxymethyl)phenyl]-3-oxoprop-1-en-1-yl}furan-2-yl}benzoic acid (8).

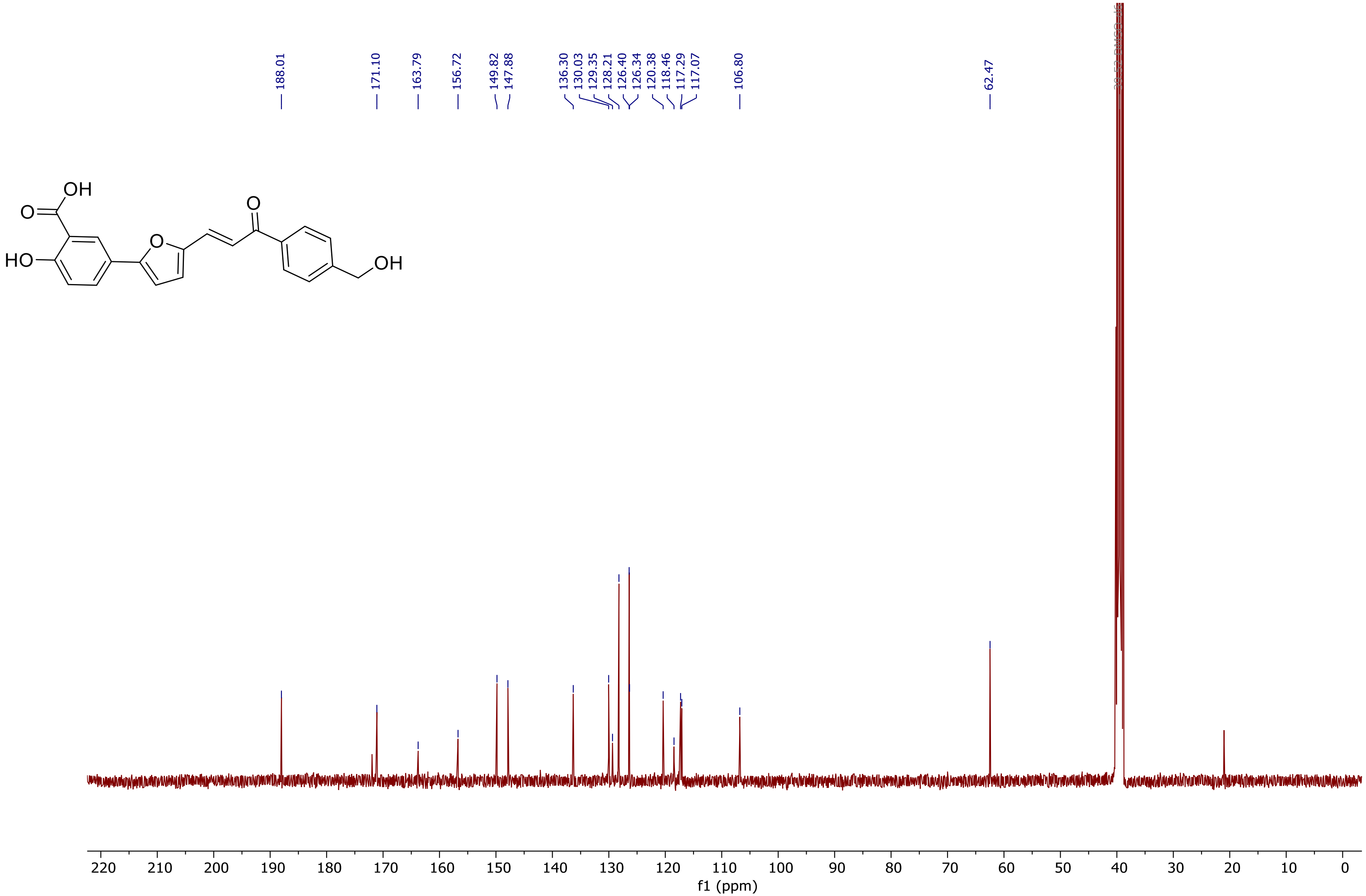

2-Hydroxy-5-{5-{(*E*)-3-[4-((*E*)-3-hydroxy-3-oxoprop-1-enyl)phenyl]-3-oxoprop-1-enyl}furan-2-yl}benzoic acid (9)

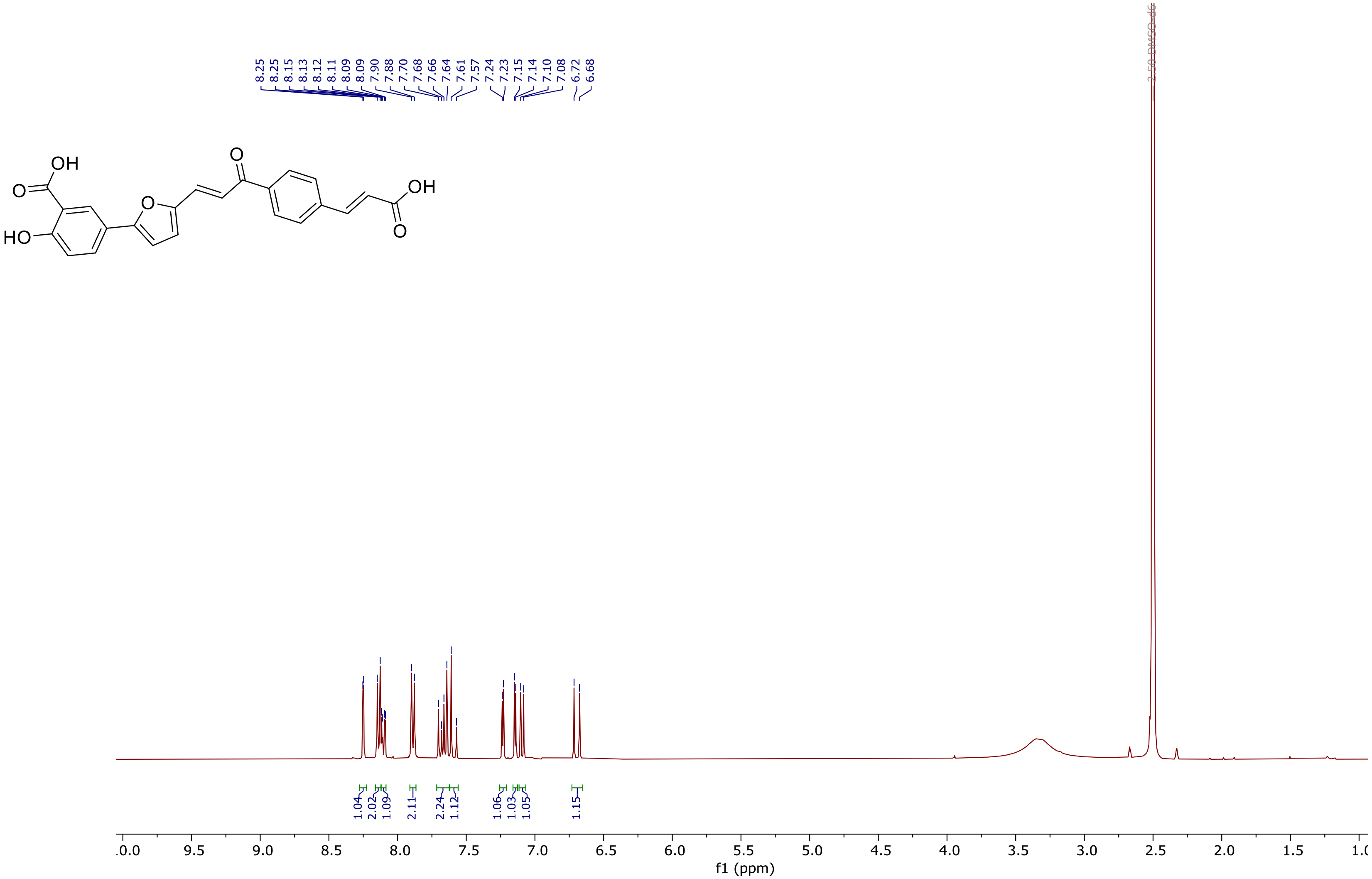

2-Hydroxy-5-{5-{(*E*)-3-[4-((*E*)-3-hydroxy-3-oxoprop-1-enyl)phenyl]-3-oxoprop-1-enyl}furan-2-yl}benzoic acid (9)

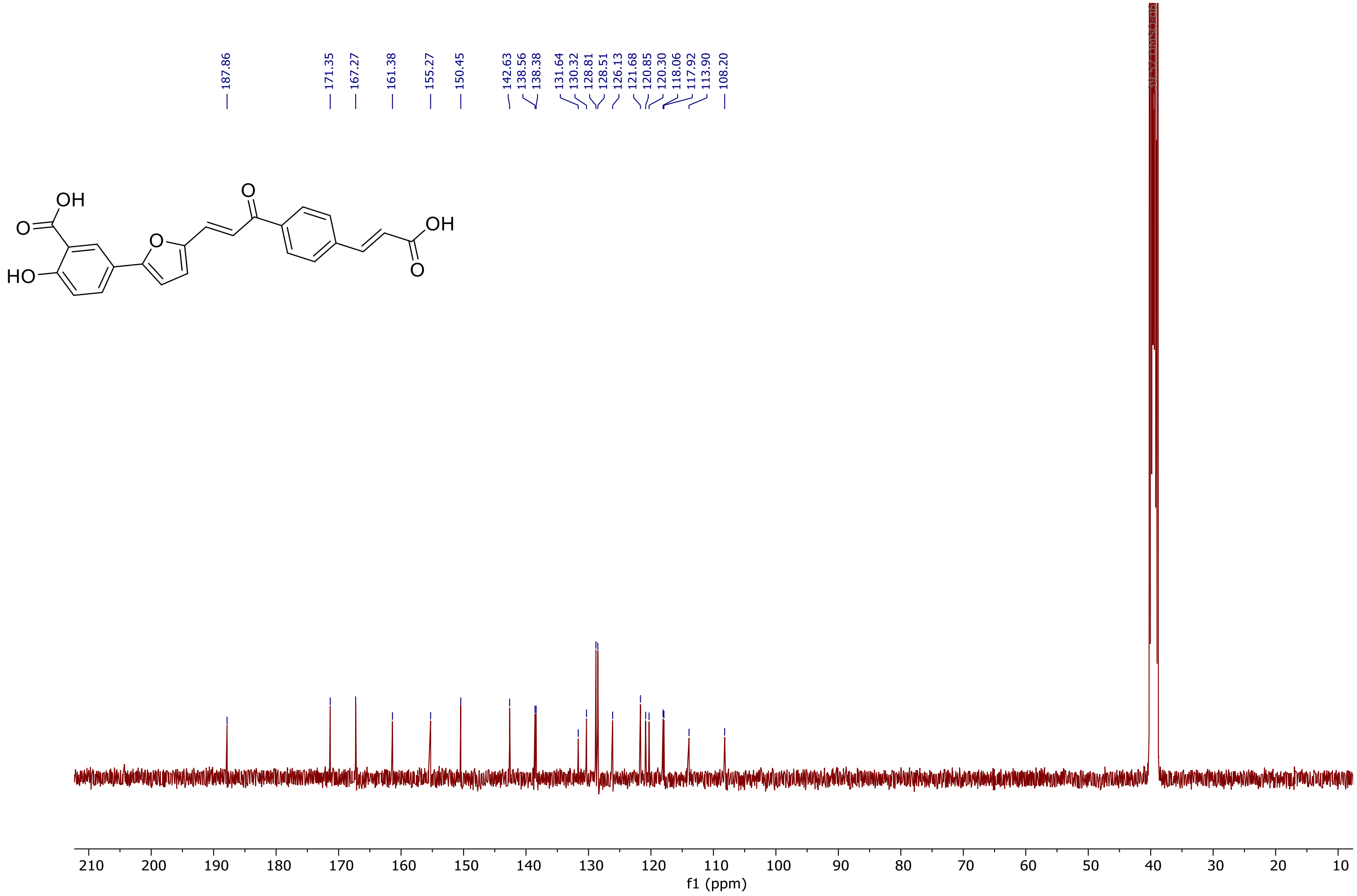

(*E*)-5-{5-{3-[4-(2-Carboxyethyl)phenyl]-3-oxoprop-1-en-1-yl}furan-2-yl}-2-hydroxybenzoic acid (10).

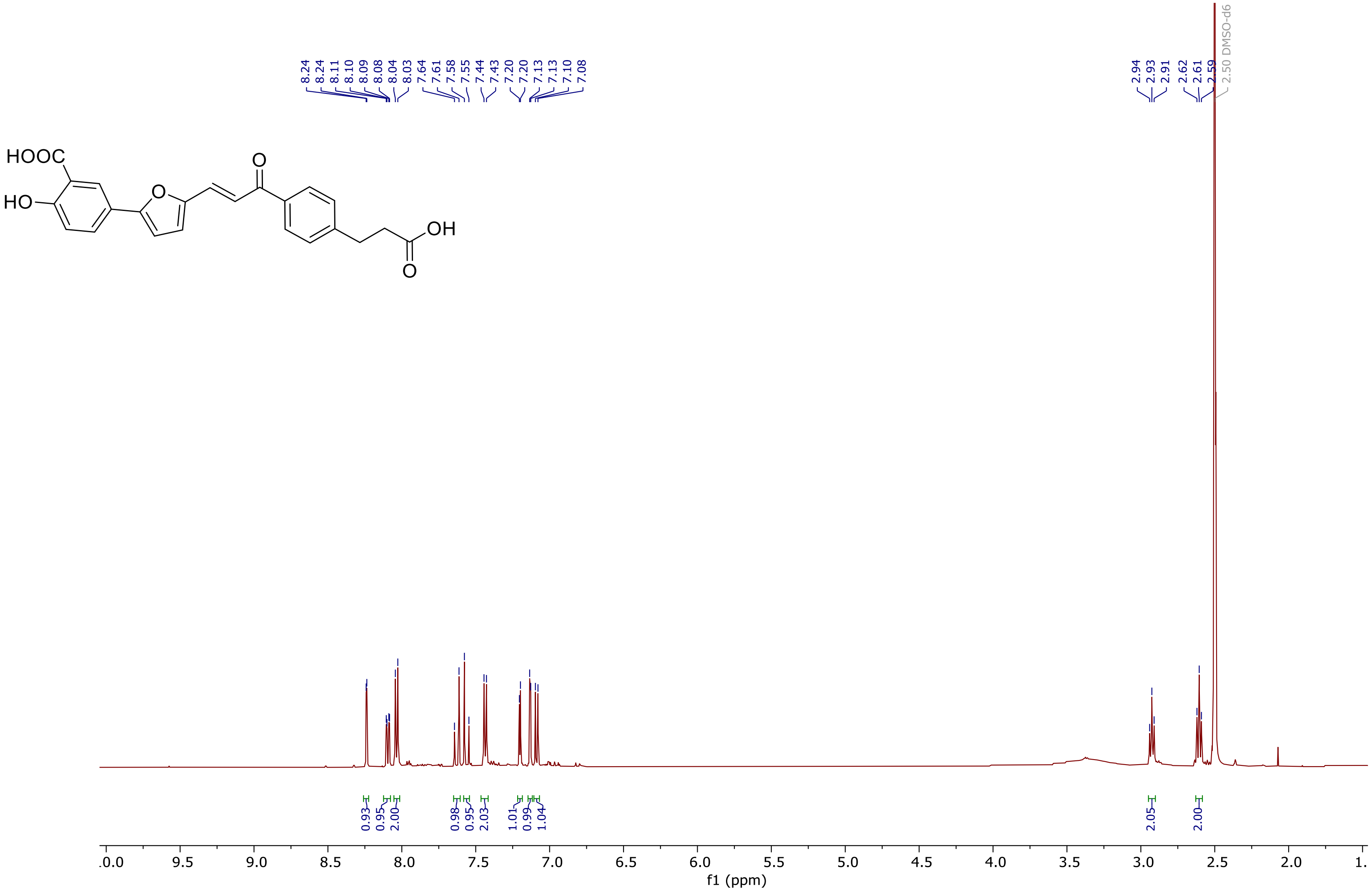

(*E*)-5-{5-{3-[4-(2-Carboxyethyl)phenyl]-3-oxoprop-1-en-1-yl}furan-2-yl}-2-hydroxybenzoic acid (**10**).

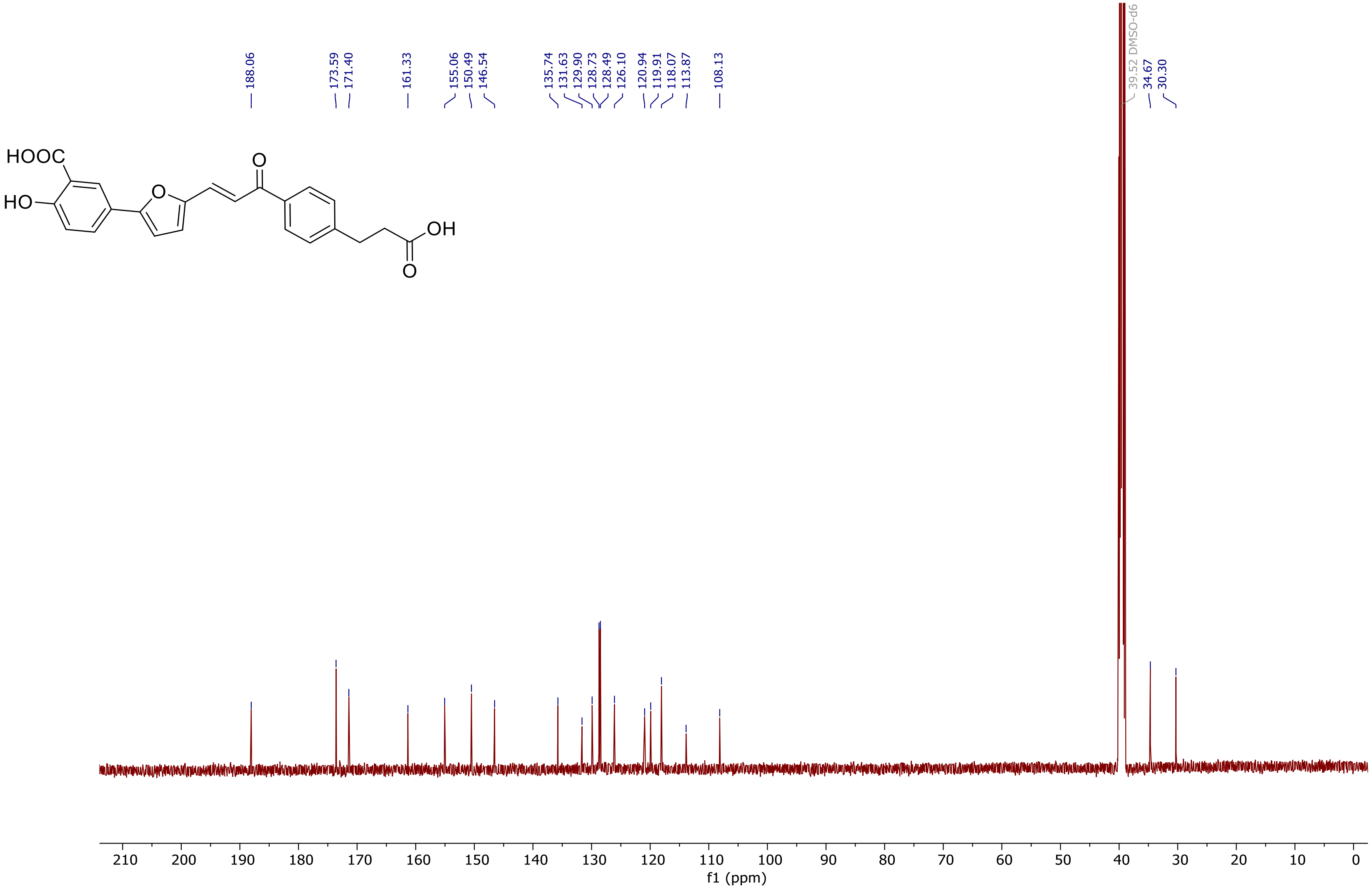

(*E*)-2-Hydroxy-5-{5-[3-(4-bromophenyl)-3-oxoprop-1-en-1-yl]furan-2-yl}benzoic acid (11).

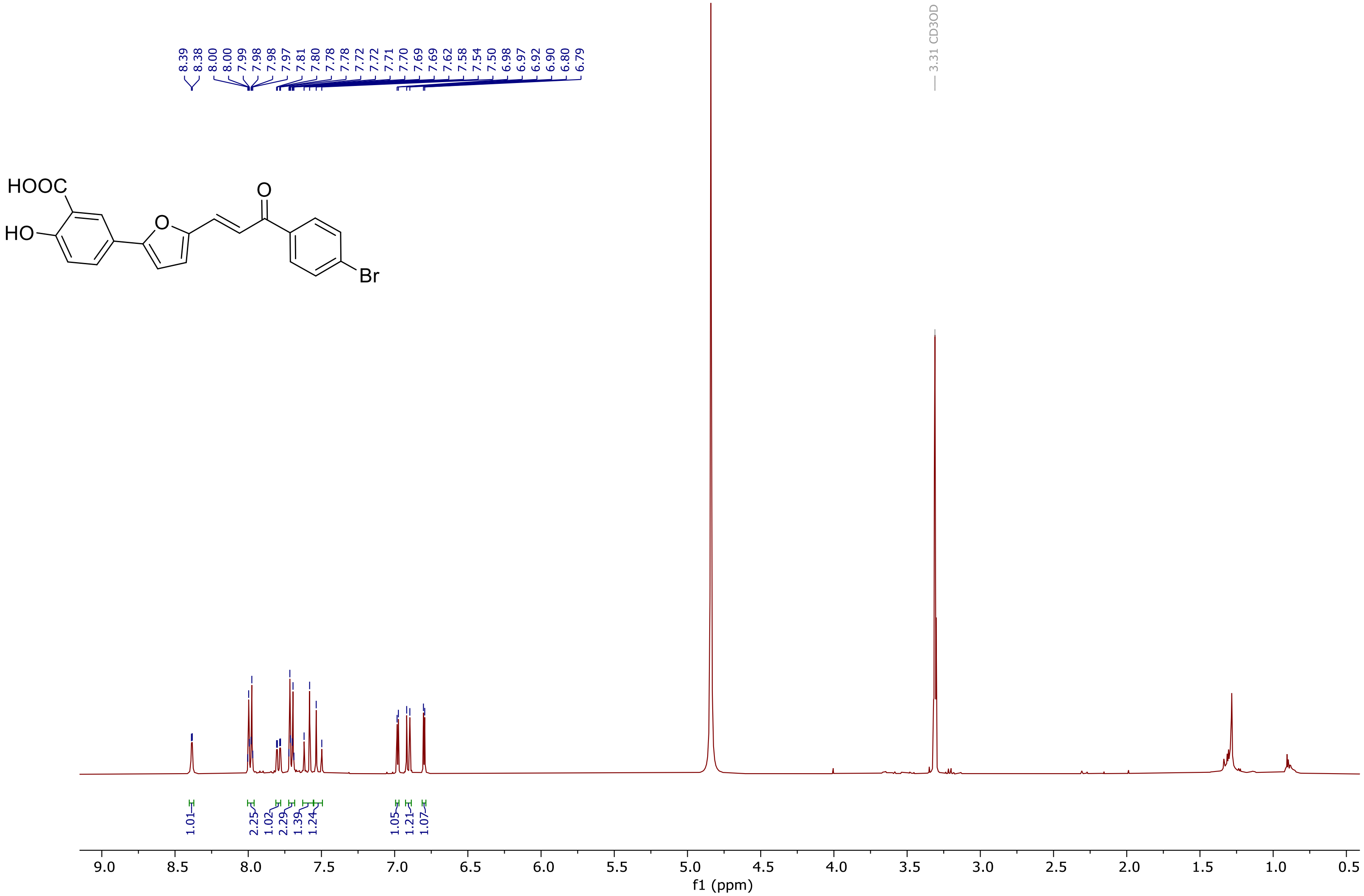

(*E*)-2-Hydroxy-5-{5-[3-(4-bromophenyl)-3-oxoprop-1-en-1-yl]furan-2-yl}benzoic acid (11).

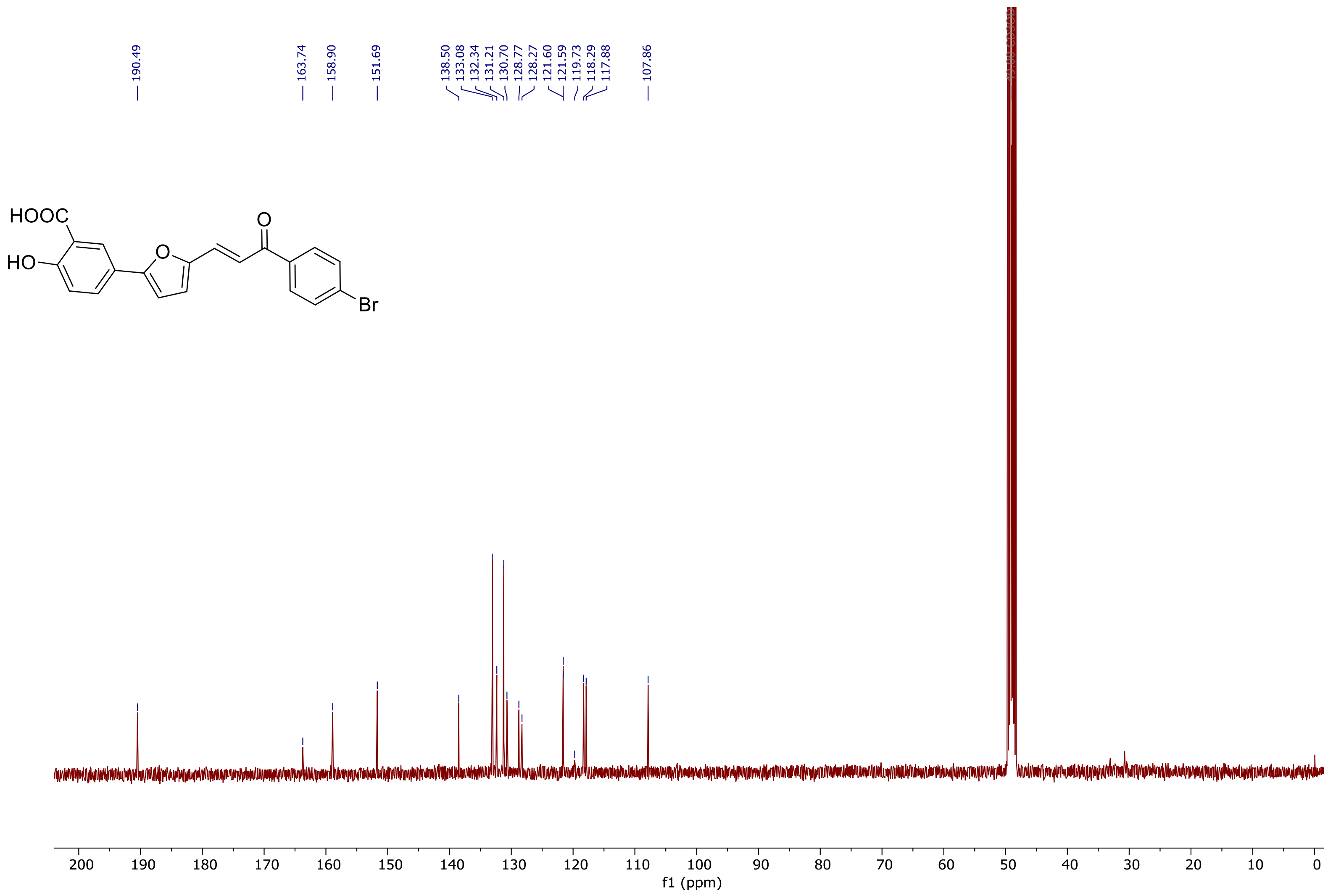

(*E*)-2-Hydroxy-5-{5-[3-(4-iodophenyl)-3-oxoprop-1-en-1-yl]furan-2-yl}benzoic acid (12).

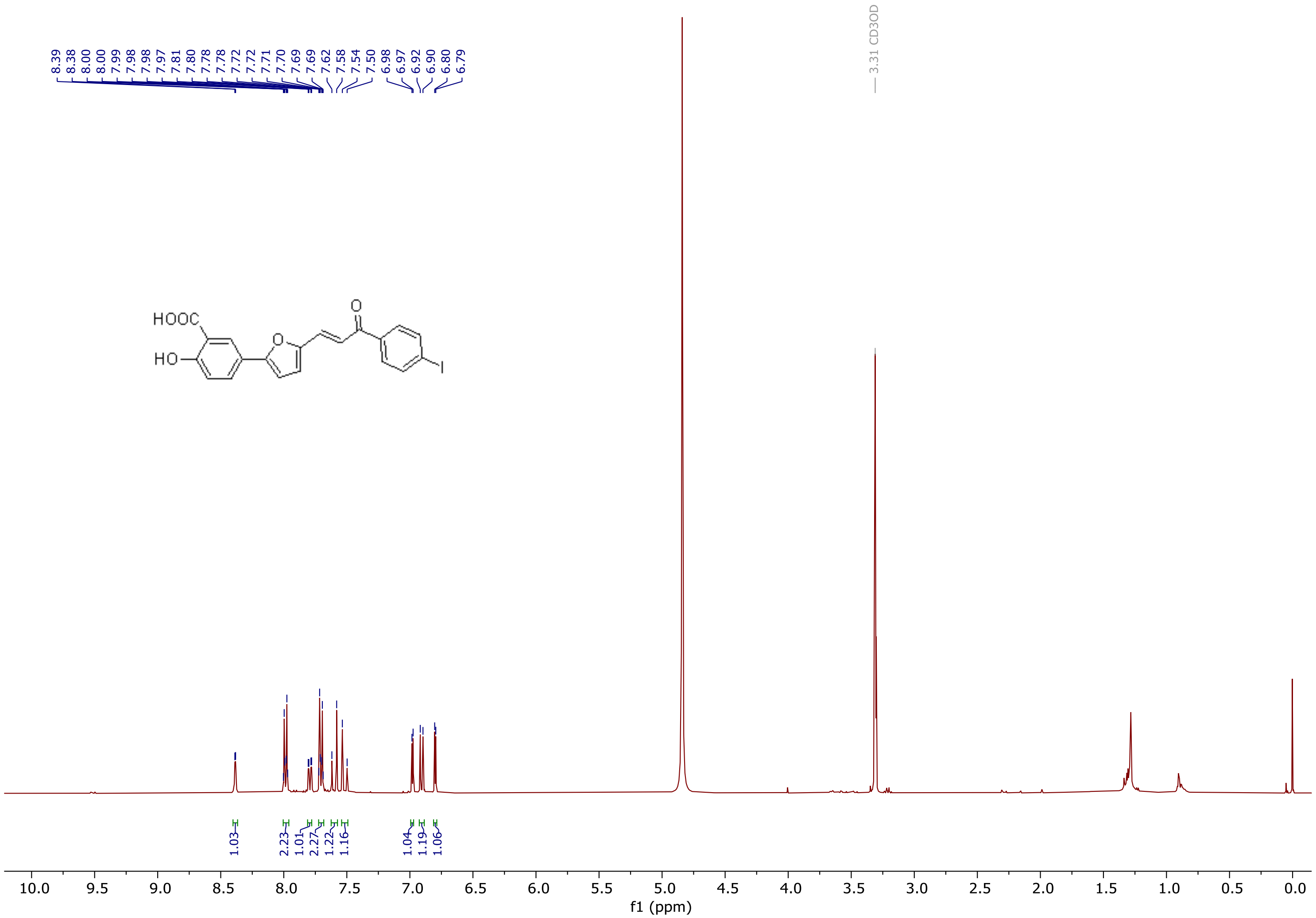

(*E*)-2-Hydroxy-5-{5-[3-(4-iodophenyl)-3-oxoprop-1-en-1-yl]furan-2-yl}benzoic acid (12).

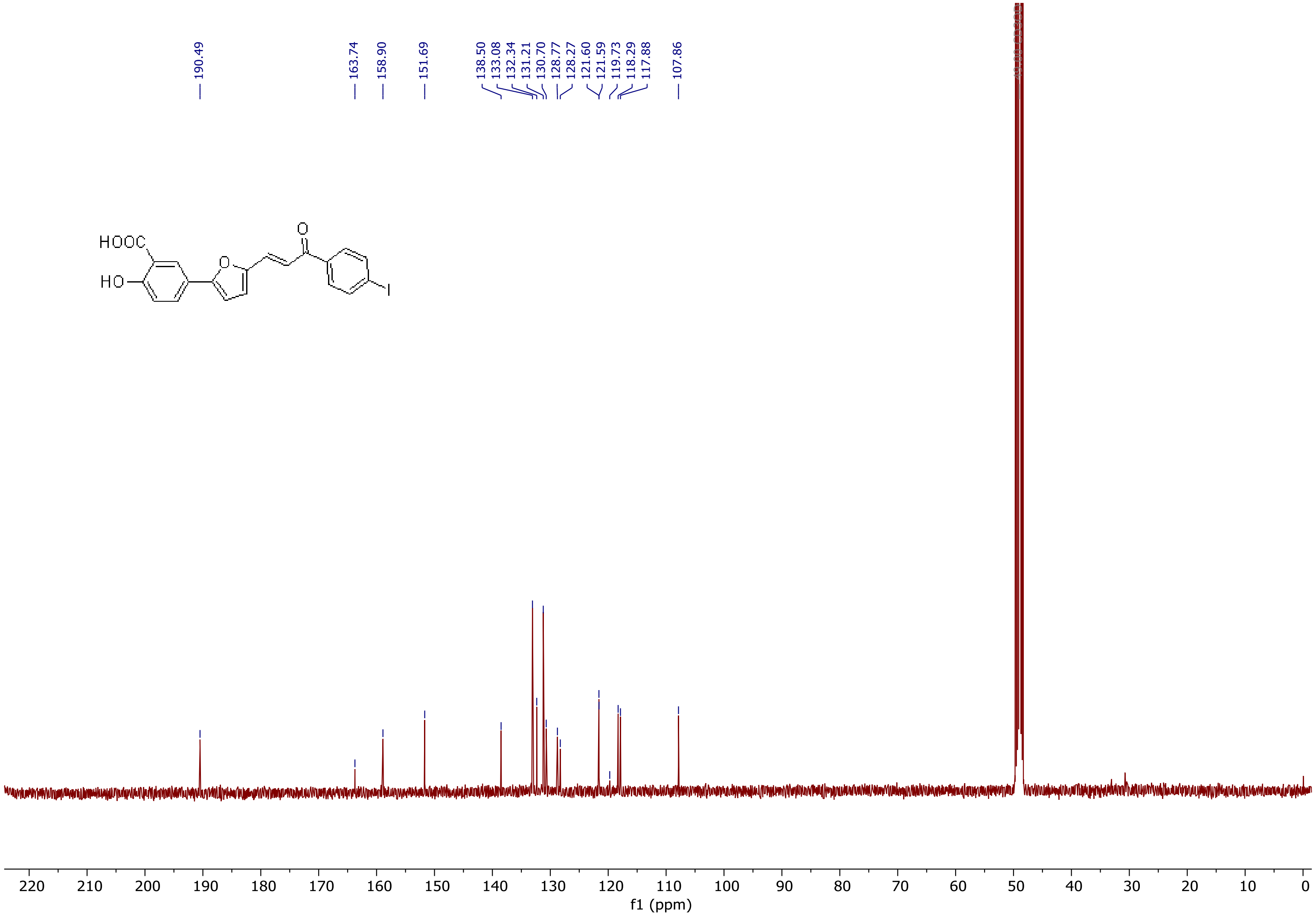

(*E*)-2-Hydroxy-5-{5-[3-(4-methoxyphenyl)-3-oxoprop-1-en-1-yl]-2-furanyl}benzoic acid (13)

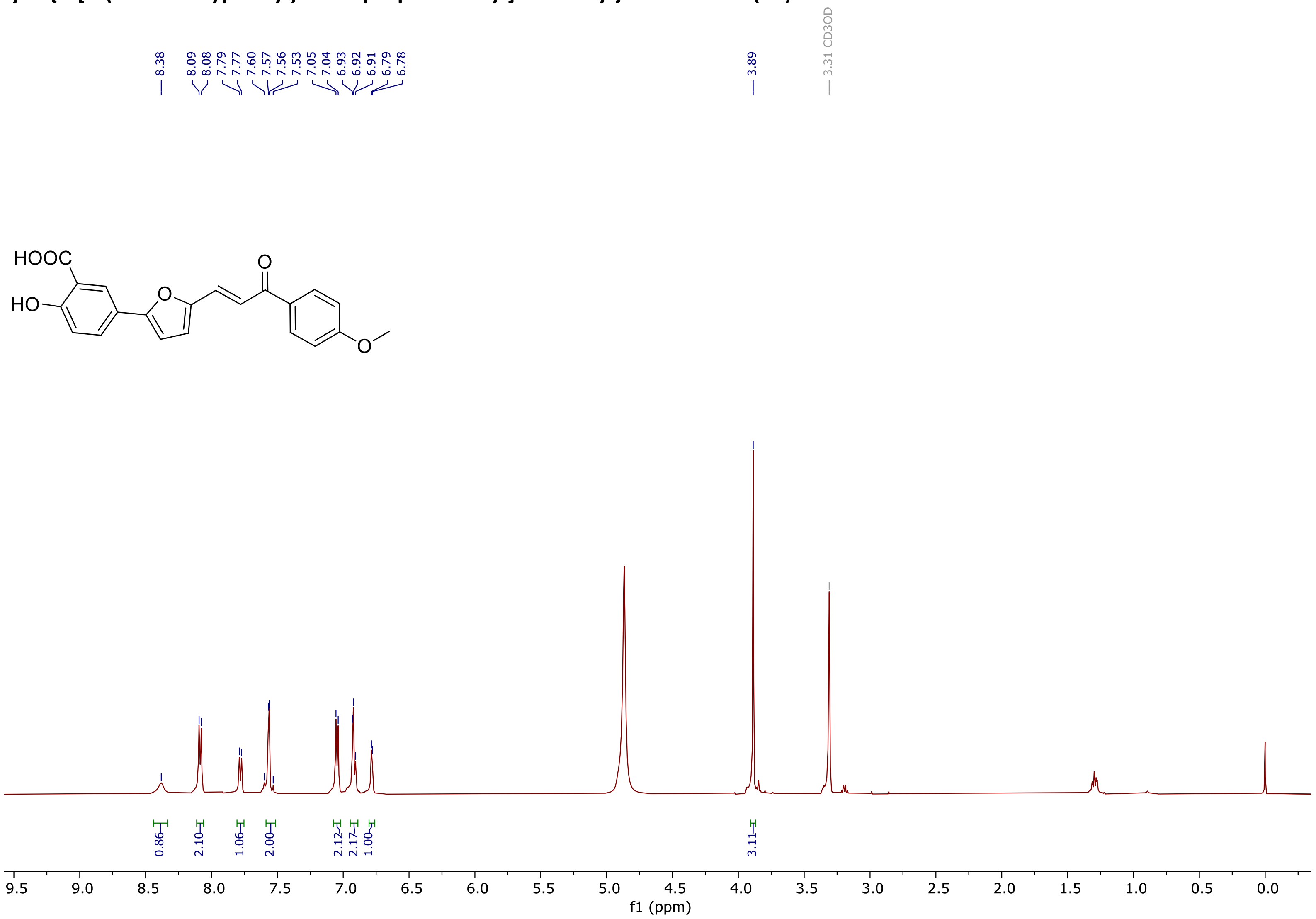

(*E*)-2-Hydroxy-5-{5-[3-(4-methoxyphenyl)-3-oxoprop-1-en-1-yl]-2-furanyl}benzoic acid (13)

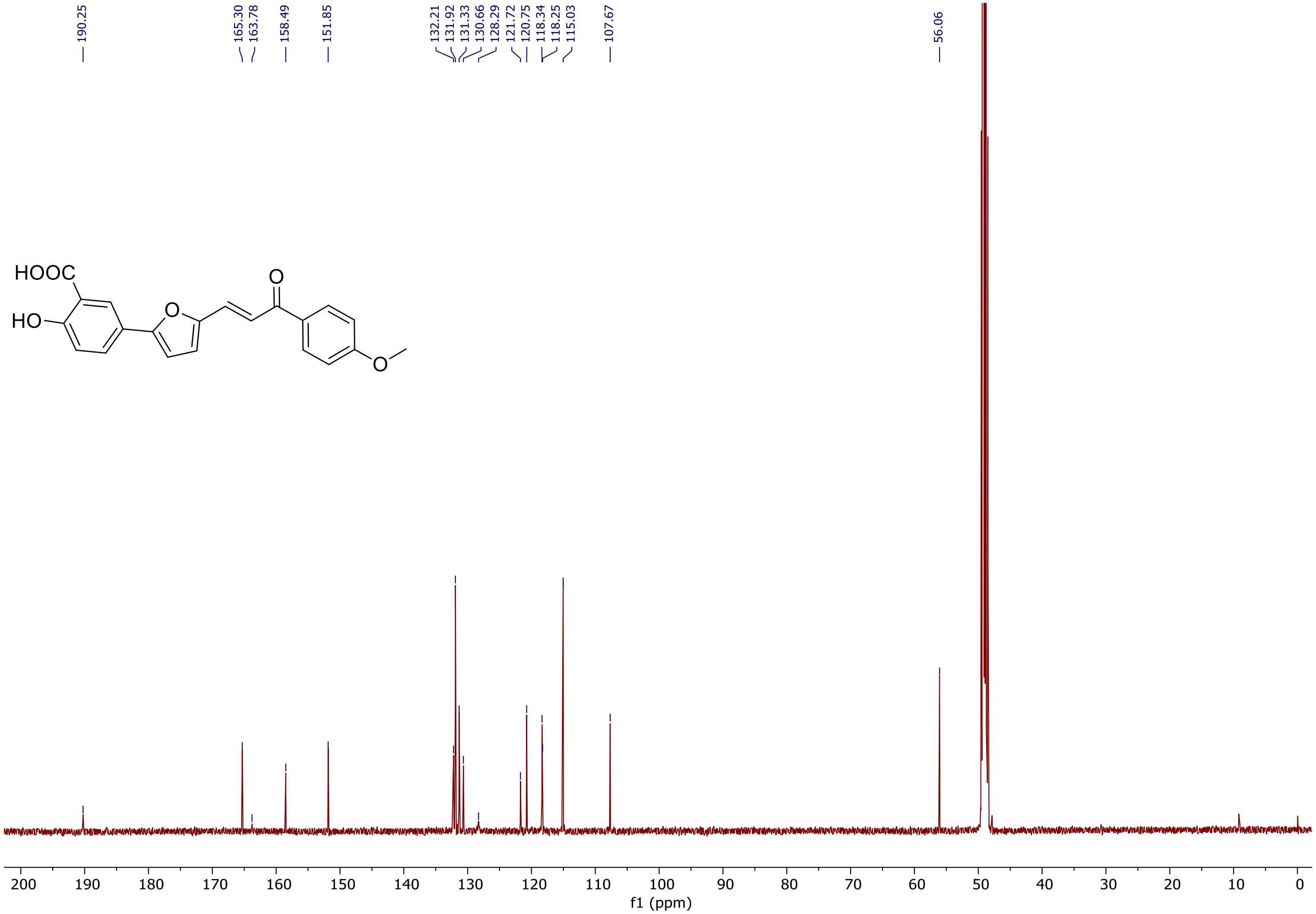

(*E*)-2-Hydroxy-5-{5-{3-(4-(methylthio)phenyl)-3-oxoprop-1-en-1-yl}furan-2-yl}benzoic acid (14)

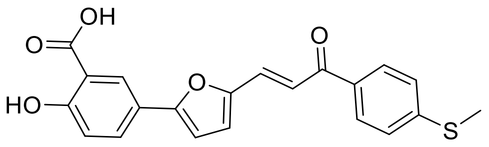

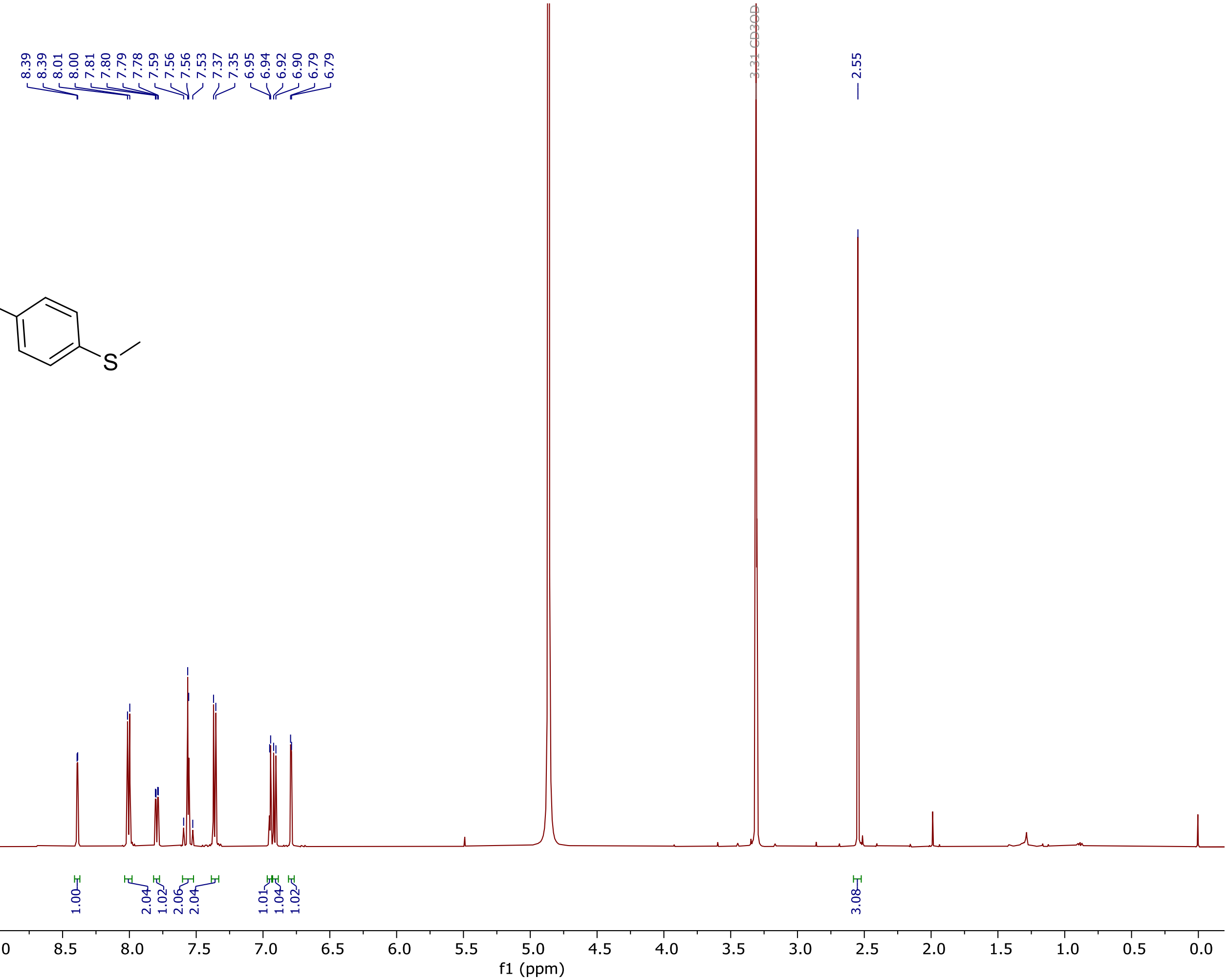

(*E*)-2-Hydroxy-5-{5-{3-(4-(methylthio)phenyl)-3-oxoprop-1-en-1-yl}furan-2-yl}benzoic acid (14).

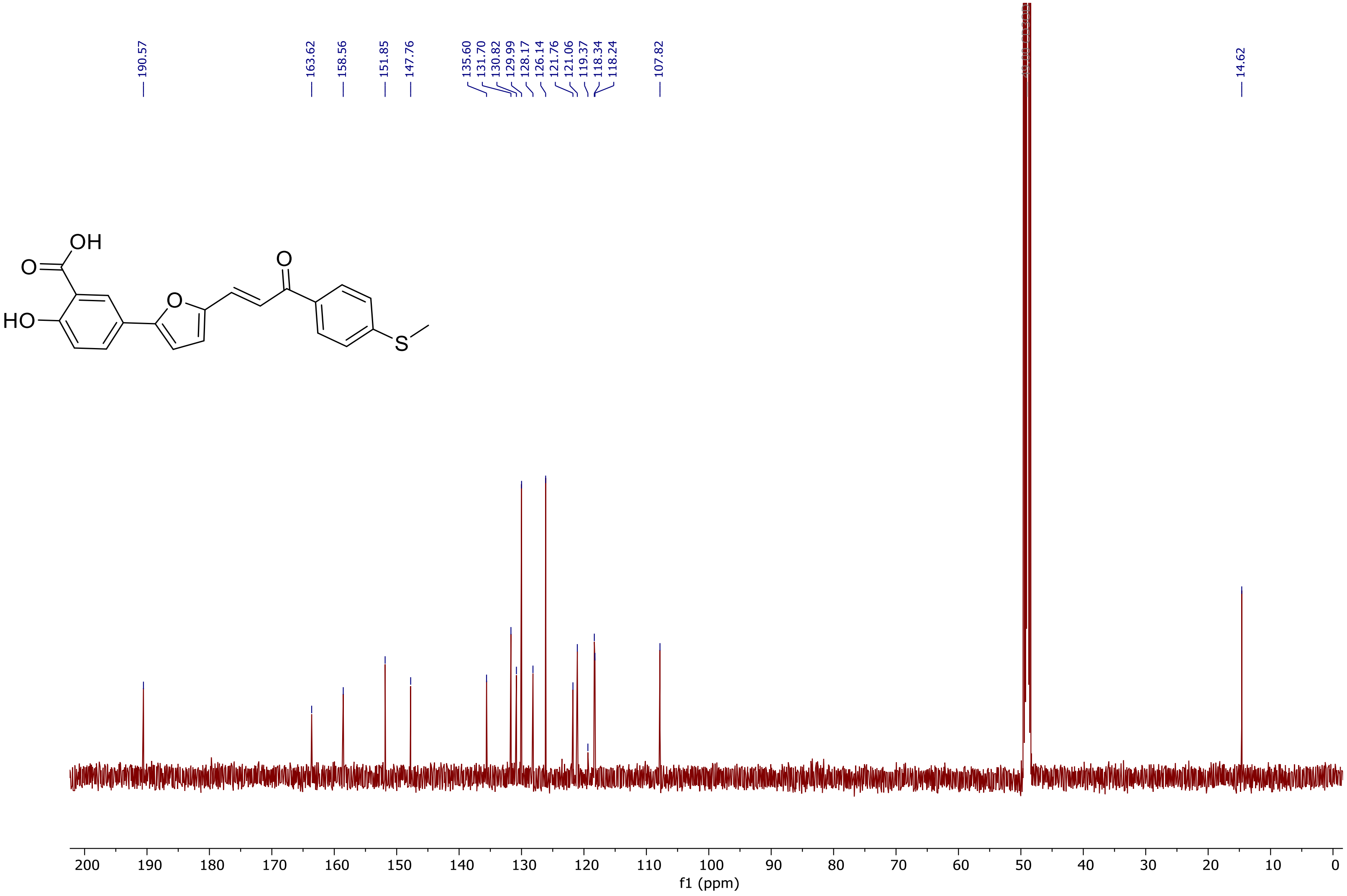

(*E*)-5-{5-{3-[4-(*N,N*-dimethylamino)phenyl]-3-oxoprop-1-en-1-yl}furan-2-yl}-2-hydroxybenzoic acid (15)

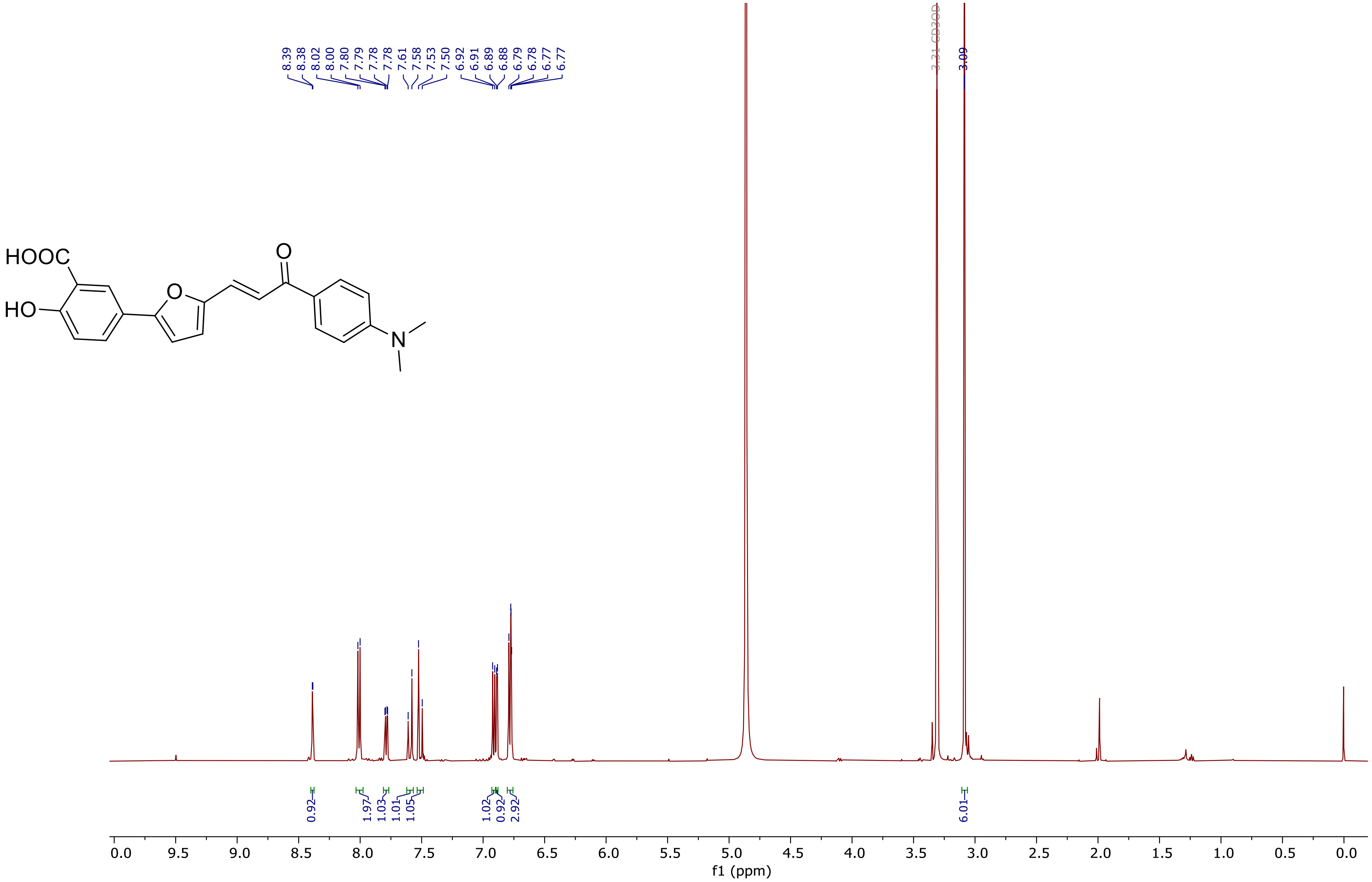

(*E*)-5-{5-{3-[4-(*N,N*-dimethylamino)phenyl]-3-oxoprop-1-en-1-yl}furan-2-yl}-2-hydroxybenzoic acid (15)

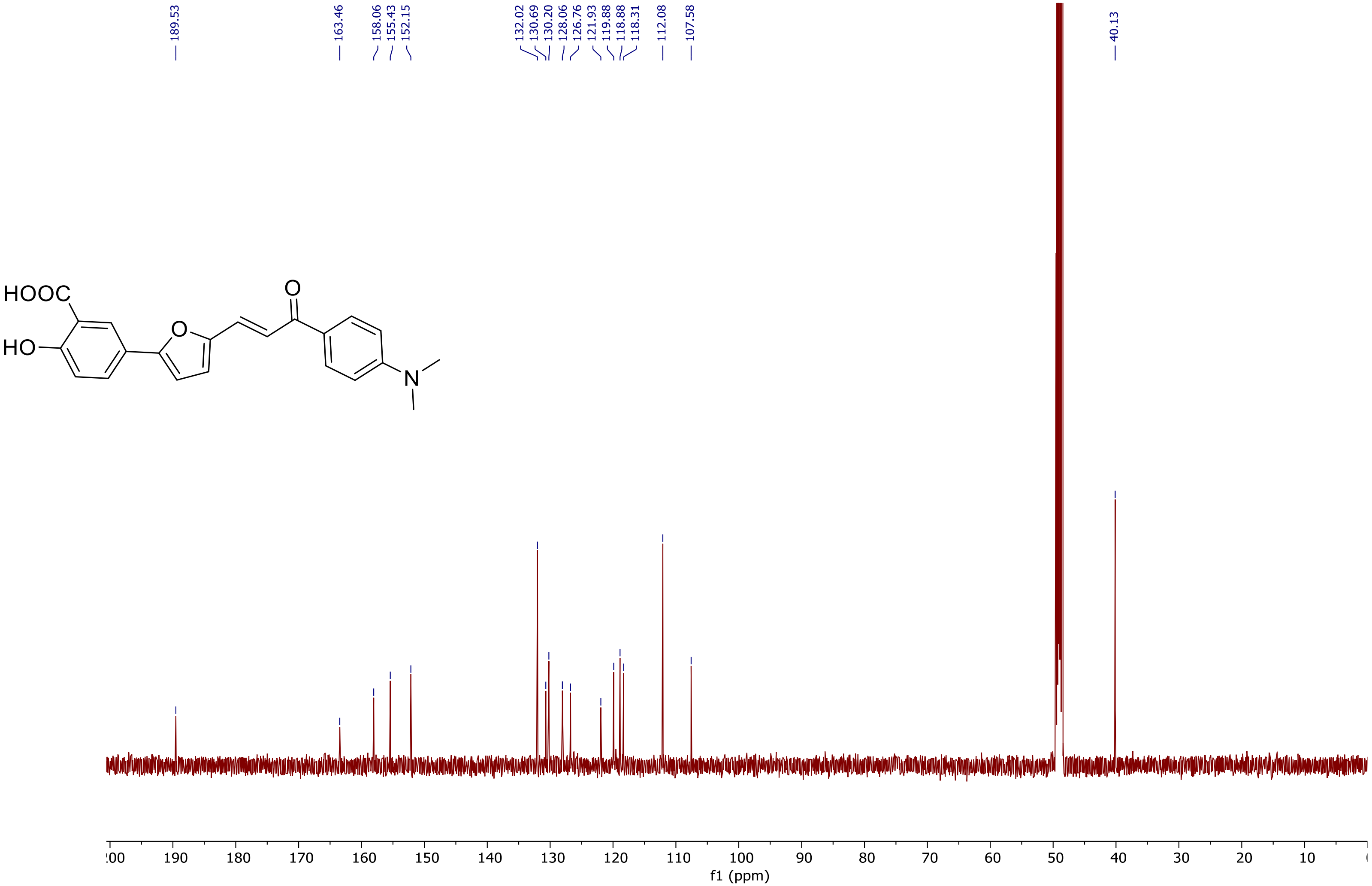

(*E*)-2-Hydroxy-5-{5-[3-oxo-3-(*p*-tolyl)prop-1-en-1-yl]furan-2-yl}benzoic acid (16)

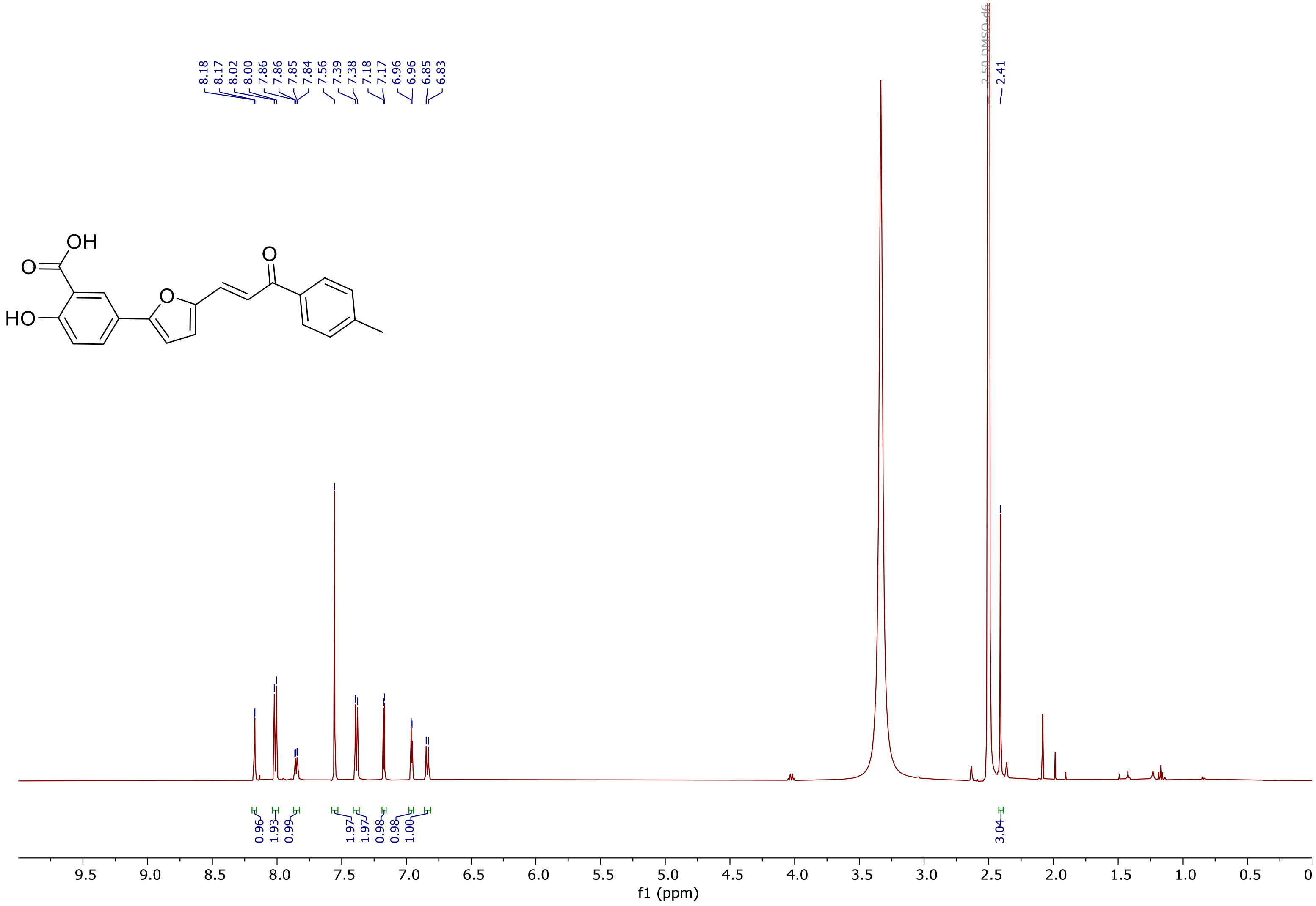

(*E*)-2-Hydroxy-5-{5-[3-oxo-3-(*p*-tolyl)prop-1-en-1-yl]furan-2-yl}benzoic acid (16)

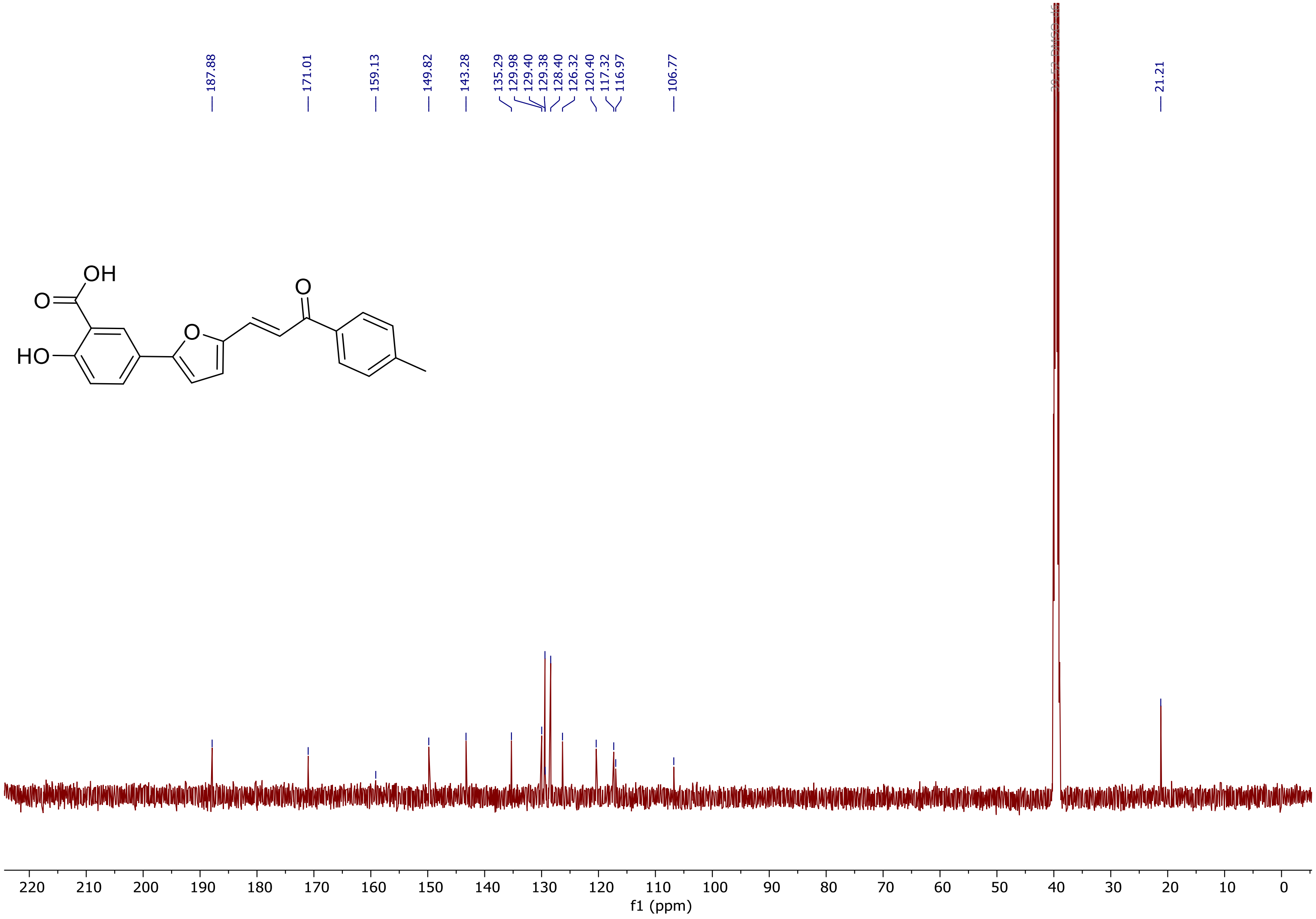

(*E*)-2-Hydroxy-5-{5-{3-[4-(trifluoromethyl)phenyl]-3-oxoprop-1-en-1-yl}furan-2-yl}-benzoic acid (17)

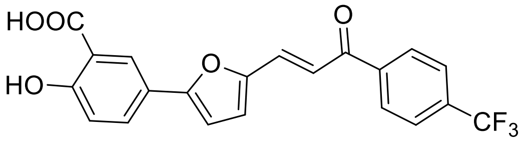

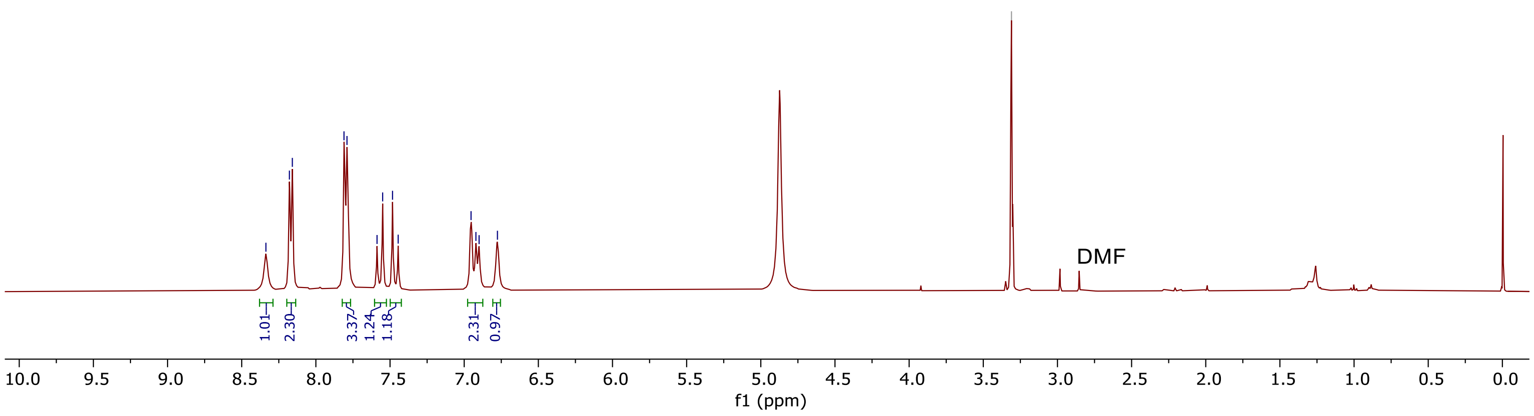

(*E*)-2-Hydroxy-5-{5-{3-[4-(trifluoromethyl)phenyl]-3-oxoprop-1-en-1-yl}furan-2-yl}-benzoic acid (17)

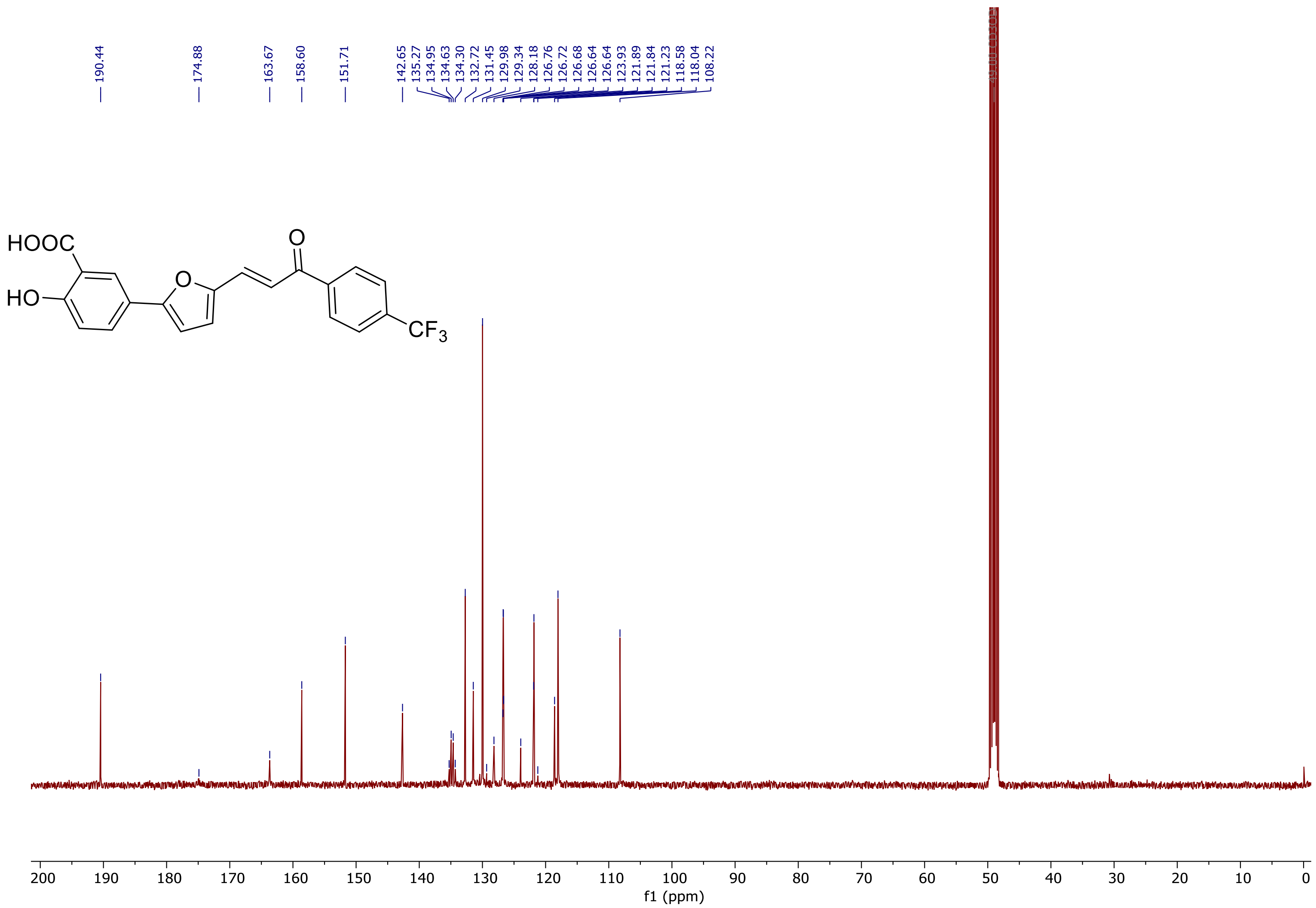

(*E*)-5-{5-[3-(4-Ethylphenyl)-3-oxoprop-1-en-1-yl]furan-2-yl}-2-hydroxybenzoic acid (18)

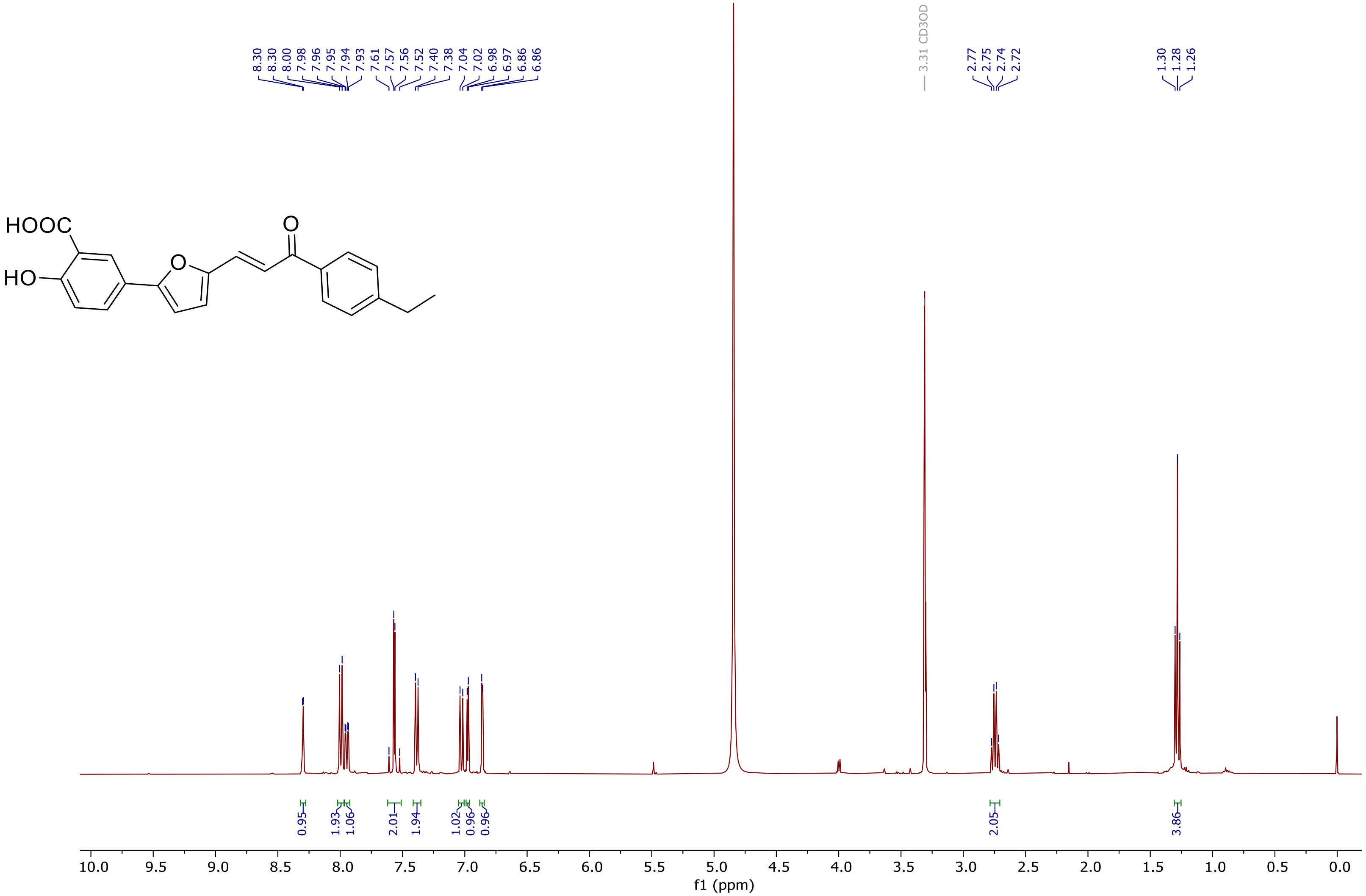

(*E*)-5-{5-[3-(4-Ethylphenyl)-3-oxoprop-1-en-1-yl]furan-2-yl}-2-hydroxybenzoic acid (18)

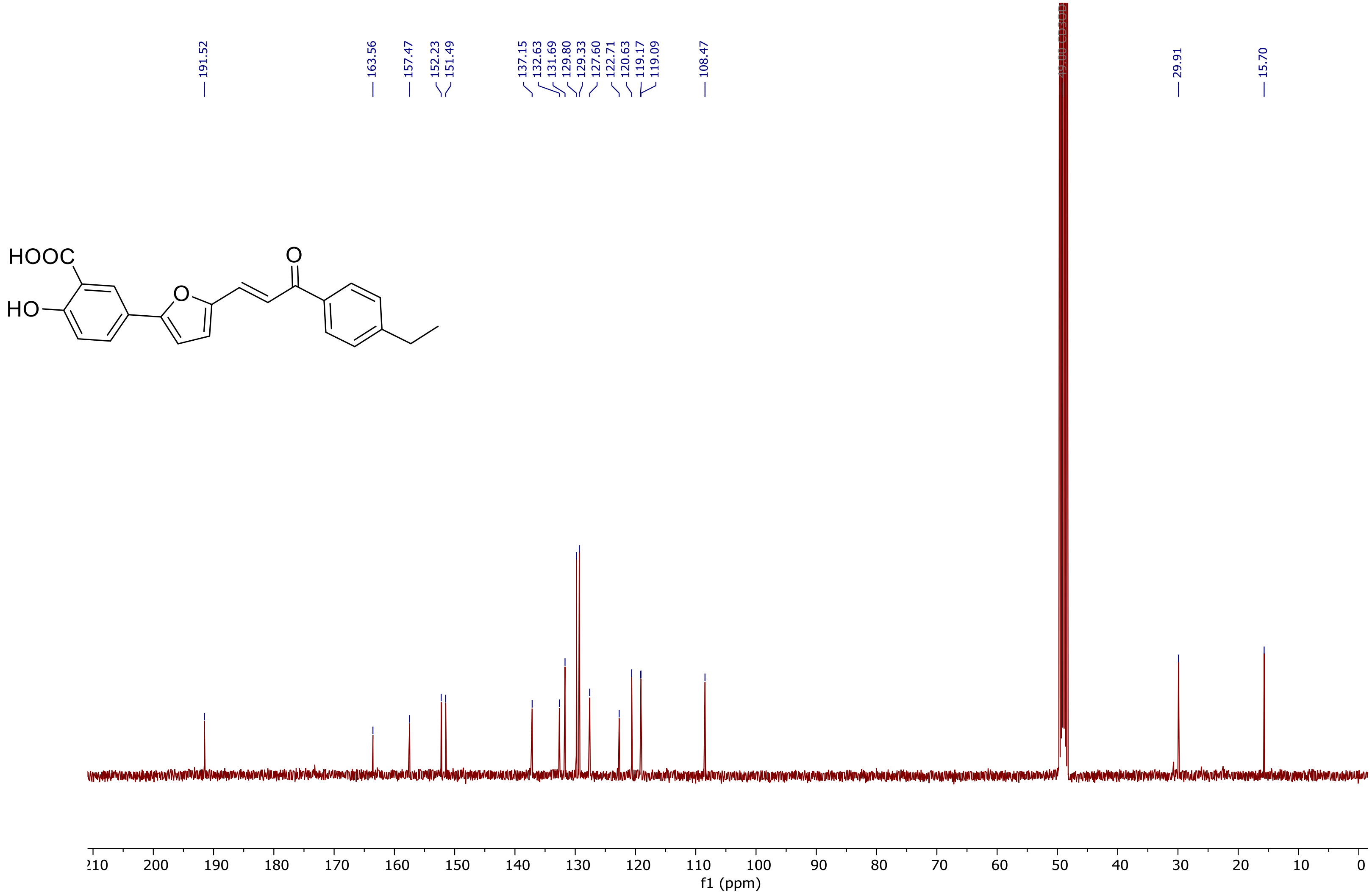

(*E*)-2-Hydroxy-5-{5-[3-(4-isopropylphenyl)-3-oxoprop-1-en-1-yl]furan-2-yl}benzoic acid (19)

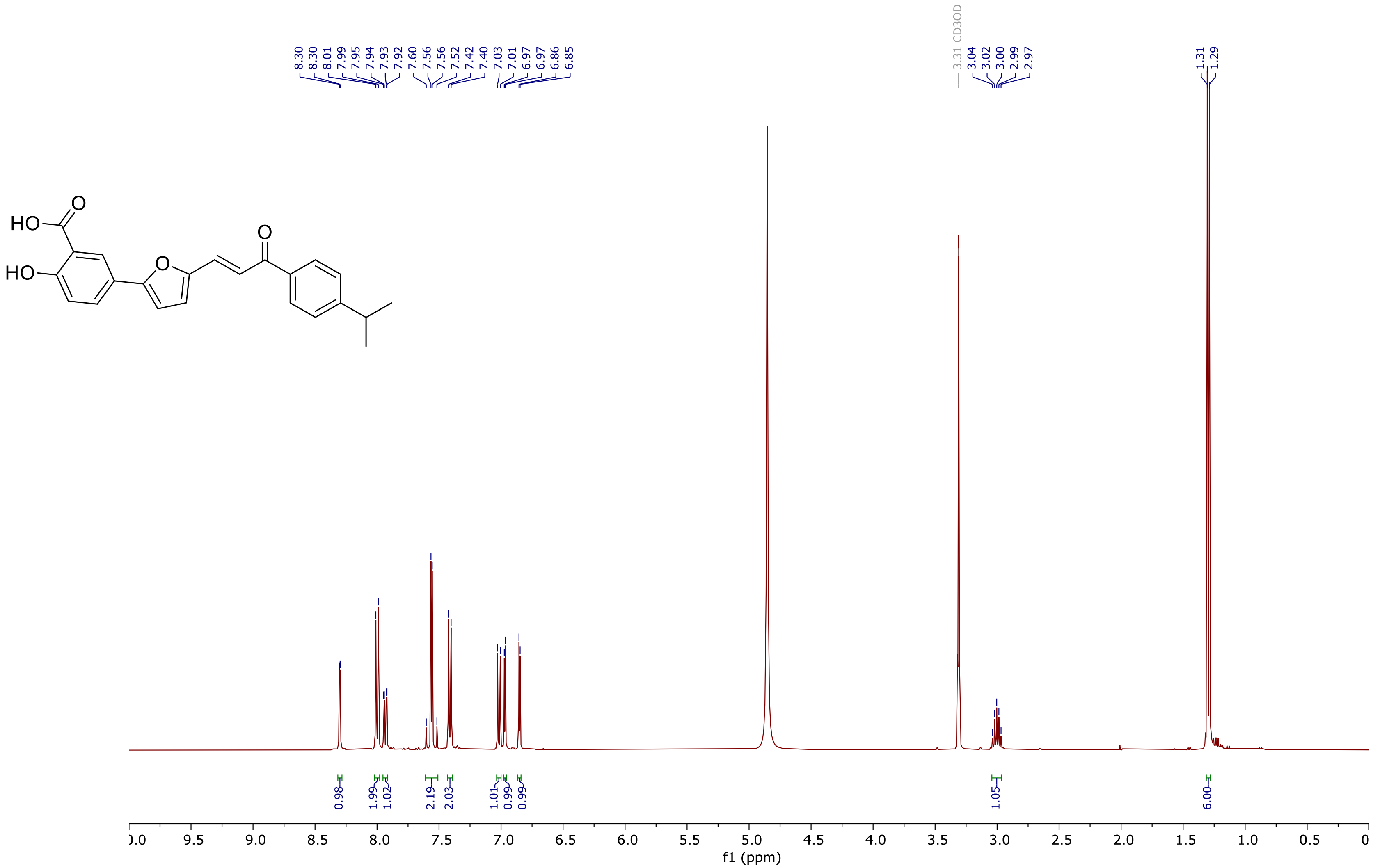

(*E*)-2-Hydroxy-5-{5-[3-(4-isopropylphenyl)-3-oxoprop-1-en-1-yl]furan-2-yl}benzoic acid (19)

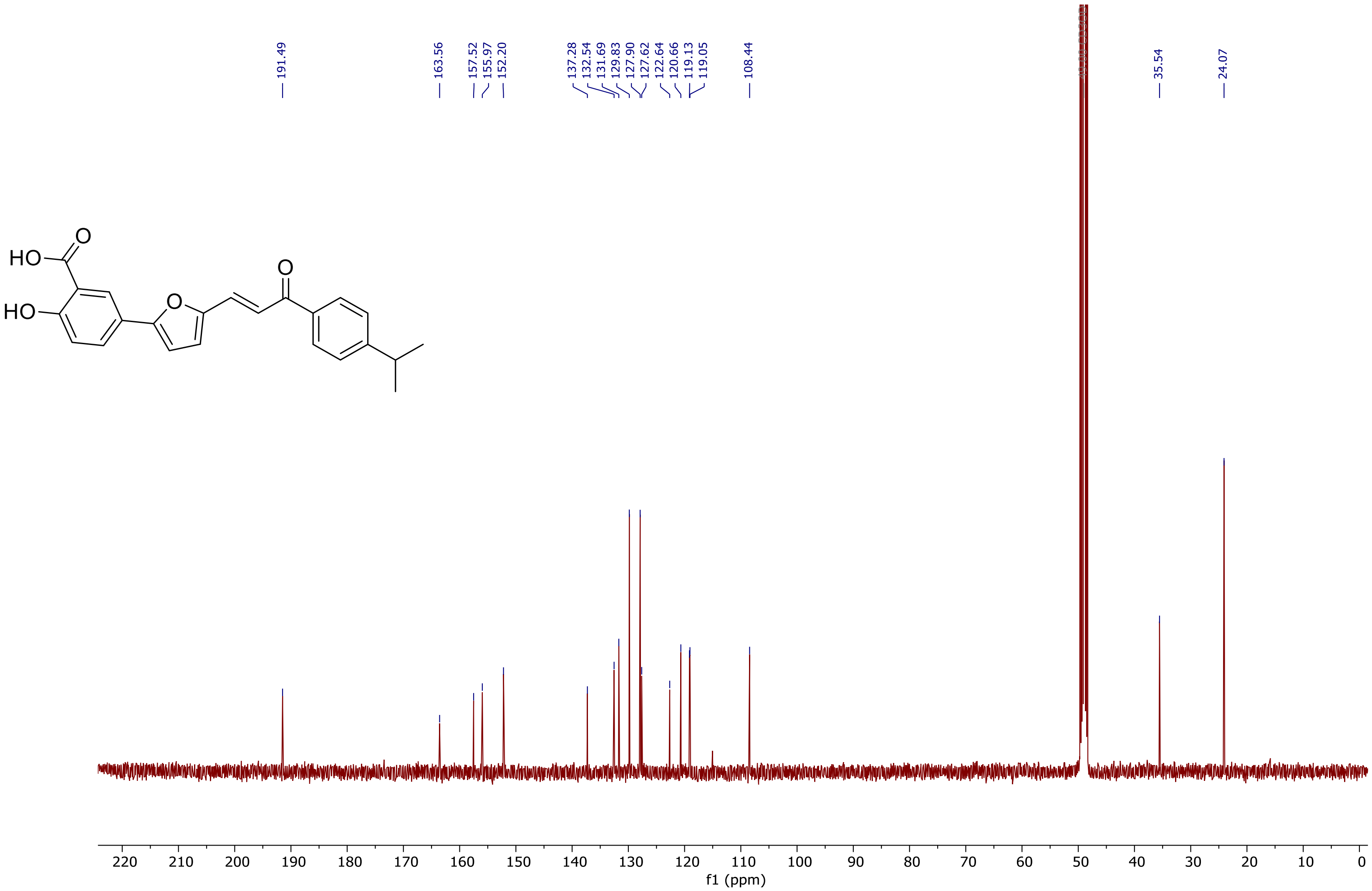

(*E*)-5-{5-[3-(4-Butylphenyl)-3-oxoprop-1-en-1-yl]furan-2-yl}-2-hydroxybenzoic acid (20)

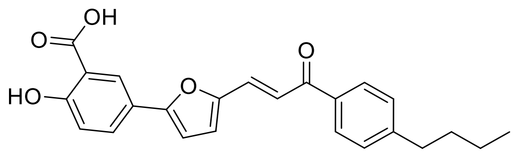

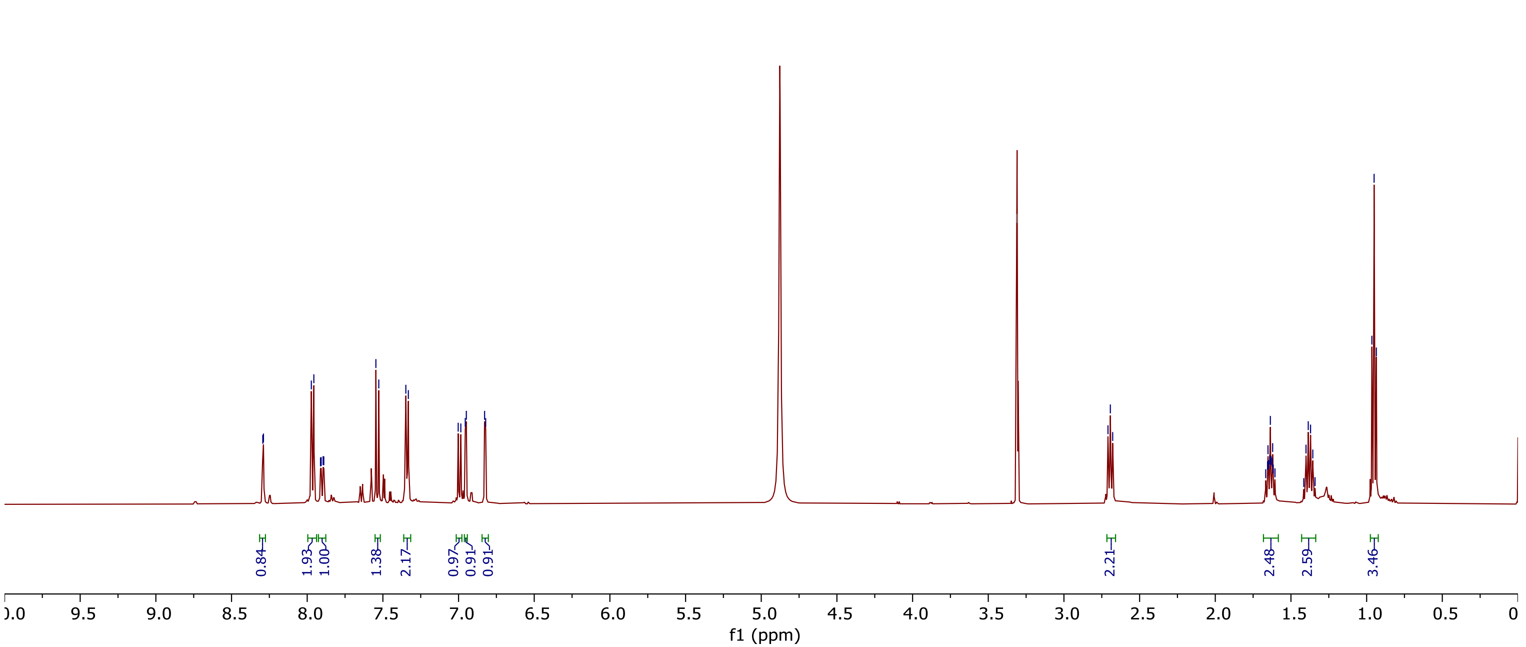

(*E*)-5-{5-[3-(4-Butylphenyl)-3-oxoprop-1-en-1-yl]furan-2-yl}-2-hydroxybenzoic acid (20)

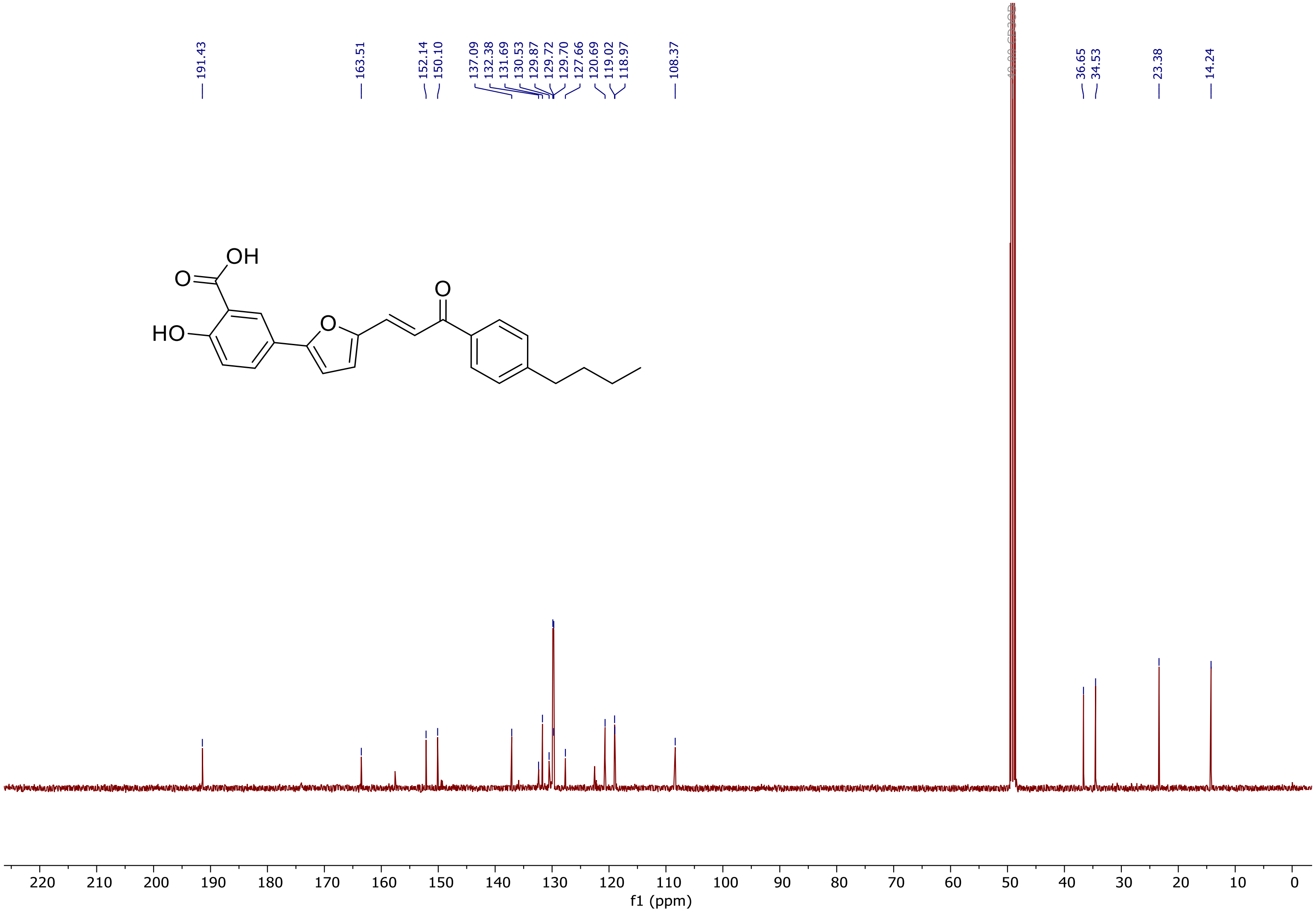

(*E*)-5-{5-[3-(4-Hexylphenyl)-3-oxoprop-1-en-1-yl]furan-2-yl}-2-hydroxybenzoic acid (21)

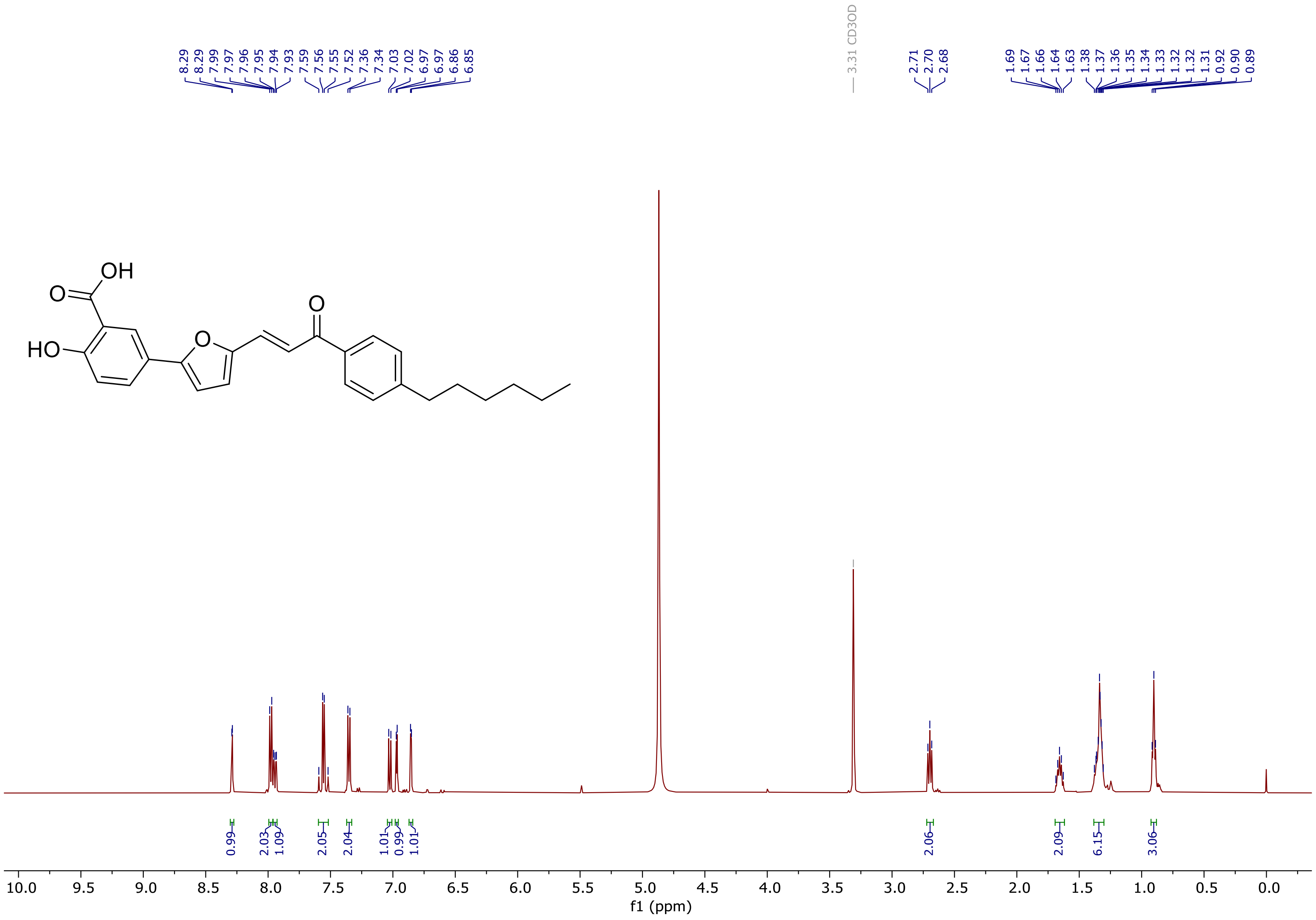

(*E*)-5-{5-[3-(4-Hexylphenyl)-3-oxoprop-1-en-1-yl]furan-2-yl}-2-hydroxybenzoic acid (21)

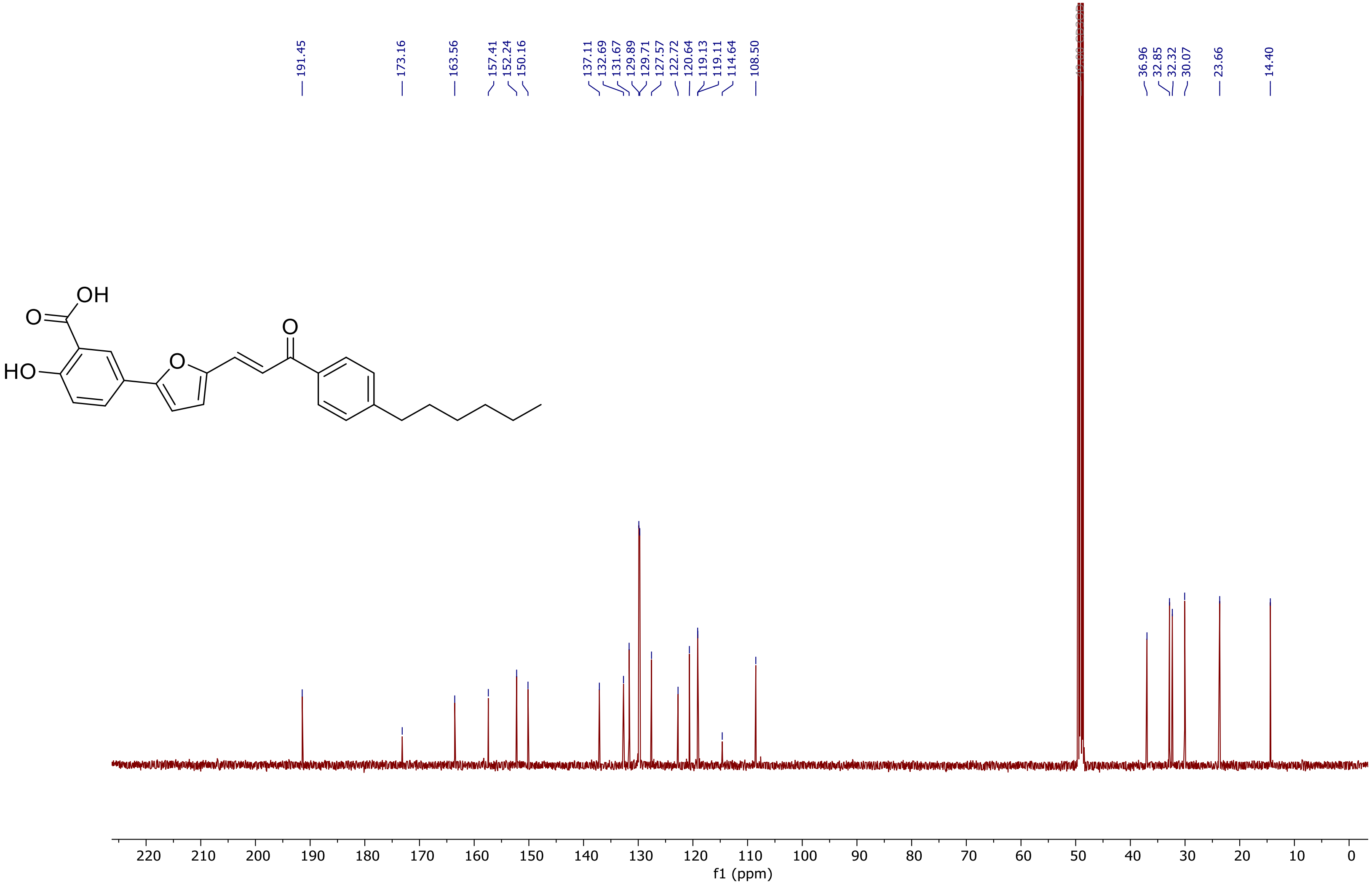

(*E*)-2-Hydroxy-5-{5-[3-(4-octylphenyl)-3-oxoprop-1-en-1-yl]furan-2-yl}benzoic acid (22)

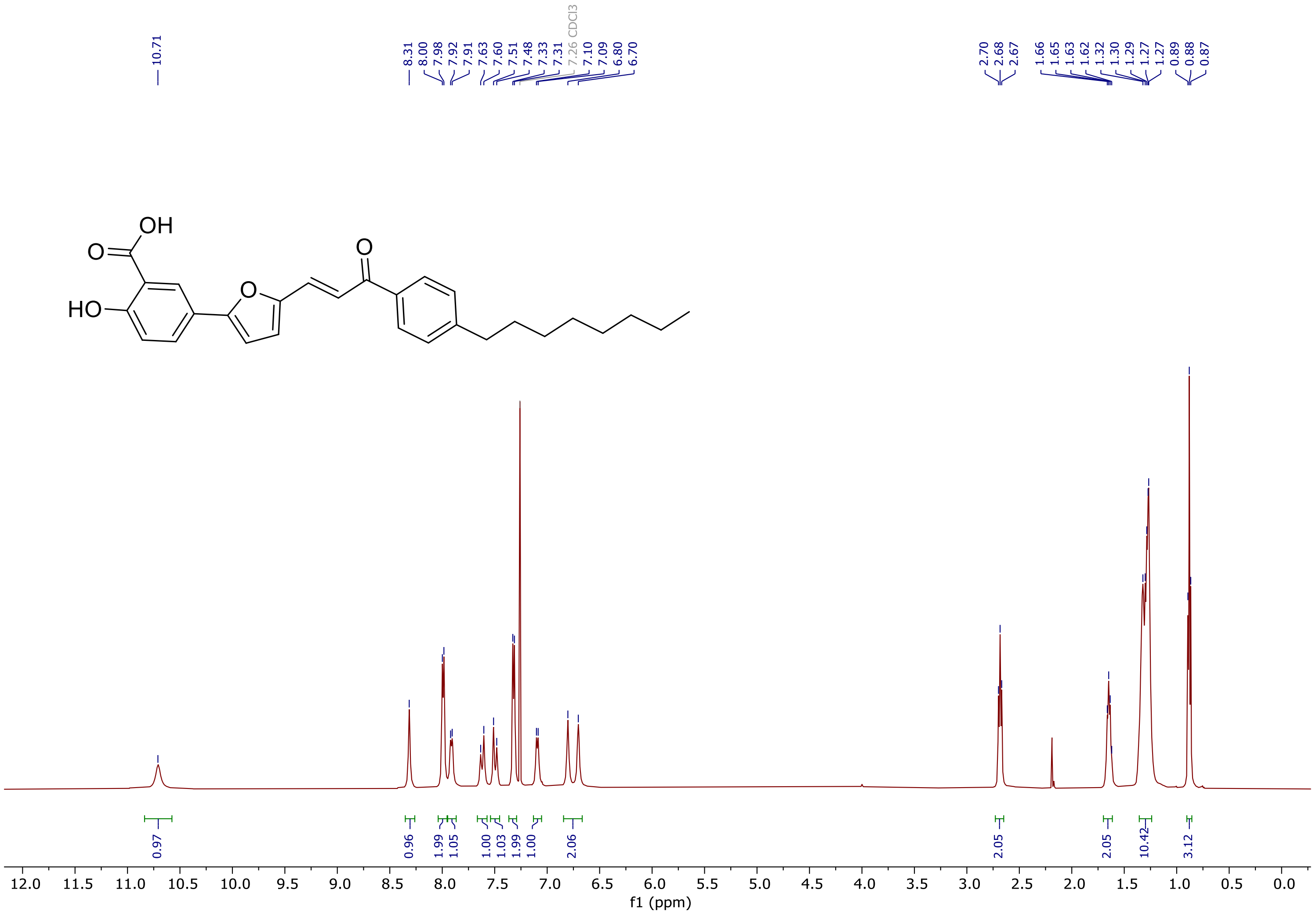

(*E*)-2-Hydroxy-5-{5-[3-(4-octylphenyl)-3-oxoprop-1-en-1-yl]furan-2-yl}benzoic acid (22)

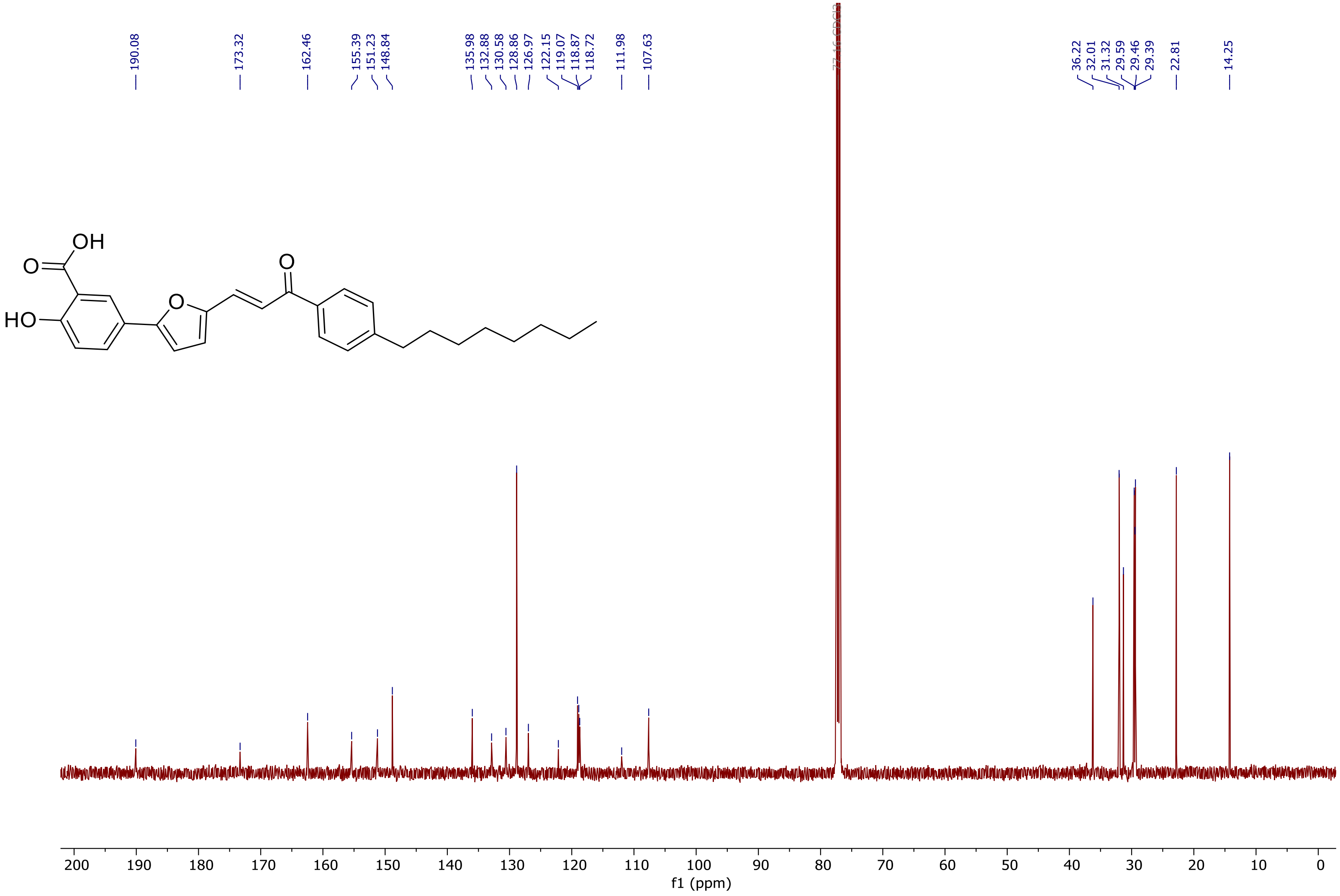

(*E*)-5-{5-{3-[4-(6-Chlorohex-1-yn-1-yl)phenyl]-3-oxoprop-1-en-1-yl}furan-2-yl}-2-hydroxybenzoic acid (23)

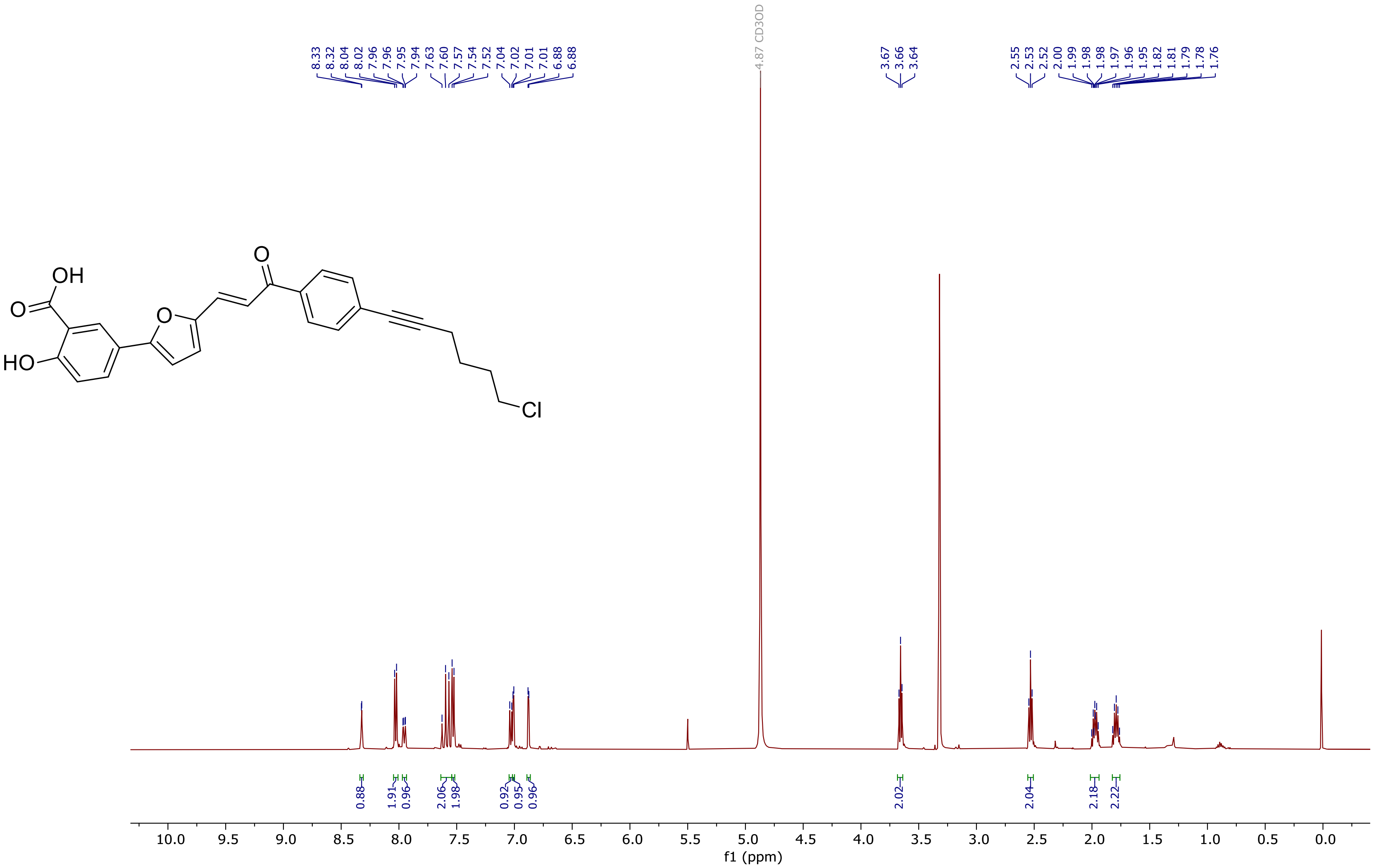

(*E*)-5-{5-{3-[4-(6-Chlorohex-1-yn-1-yl)phenyl]-3-oxoprop-1-en-1-yl}furan-2-yl}-2-hydroxybenzoic acid (23)

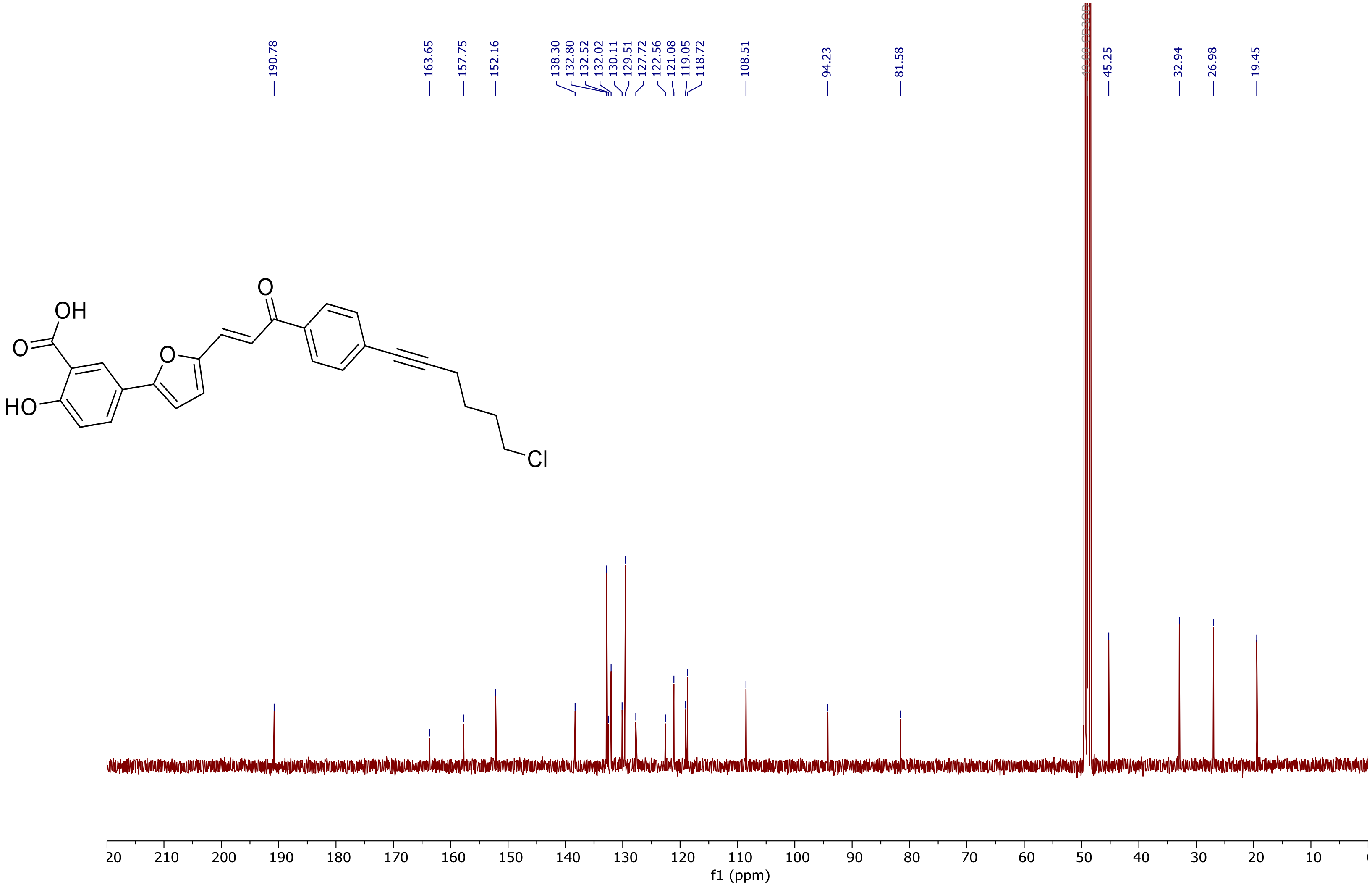

5-{5-{(*E*)-3-{4-[(*E*)-3-(*tert*-Butoxy)-3-oxoprop-1-en-1-yl]phenyl}-3-oxoprop-1-en-1-yl}furan-2-yl}-2-hydroxybenzoic acid (24)

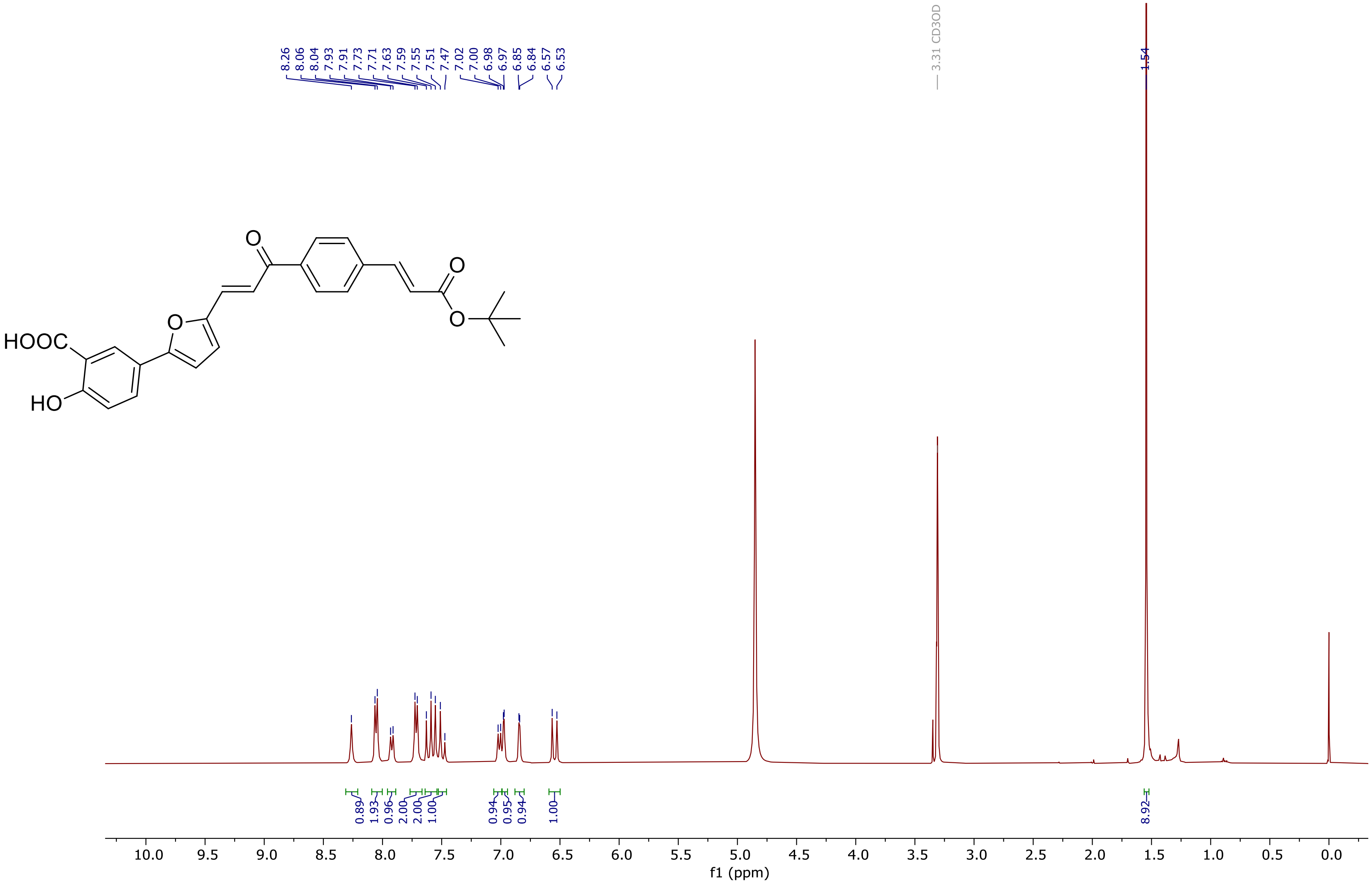

5-{5-{(*E*)-3-{4-[(*E*)-3-(*tert*-Butoxy)-3-oxoprop-1-en-1-yl]phenyl}-3-oxoprop-1-en-1-yl}furan-2-yl}-2-hydroxybenzoic acid (24)

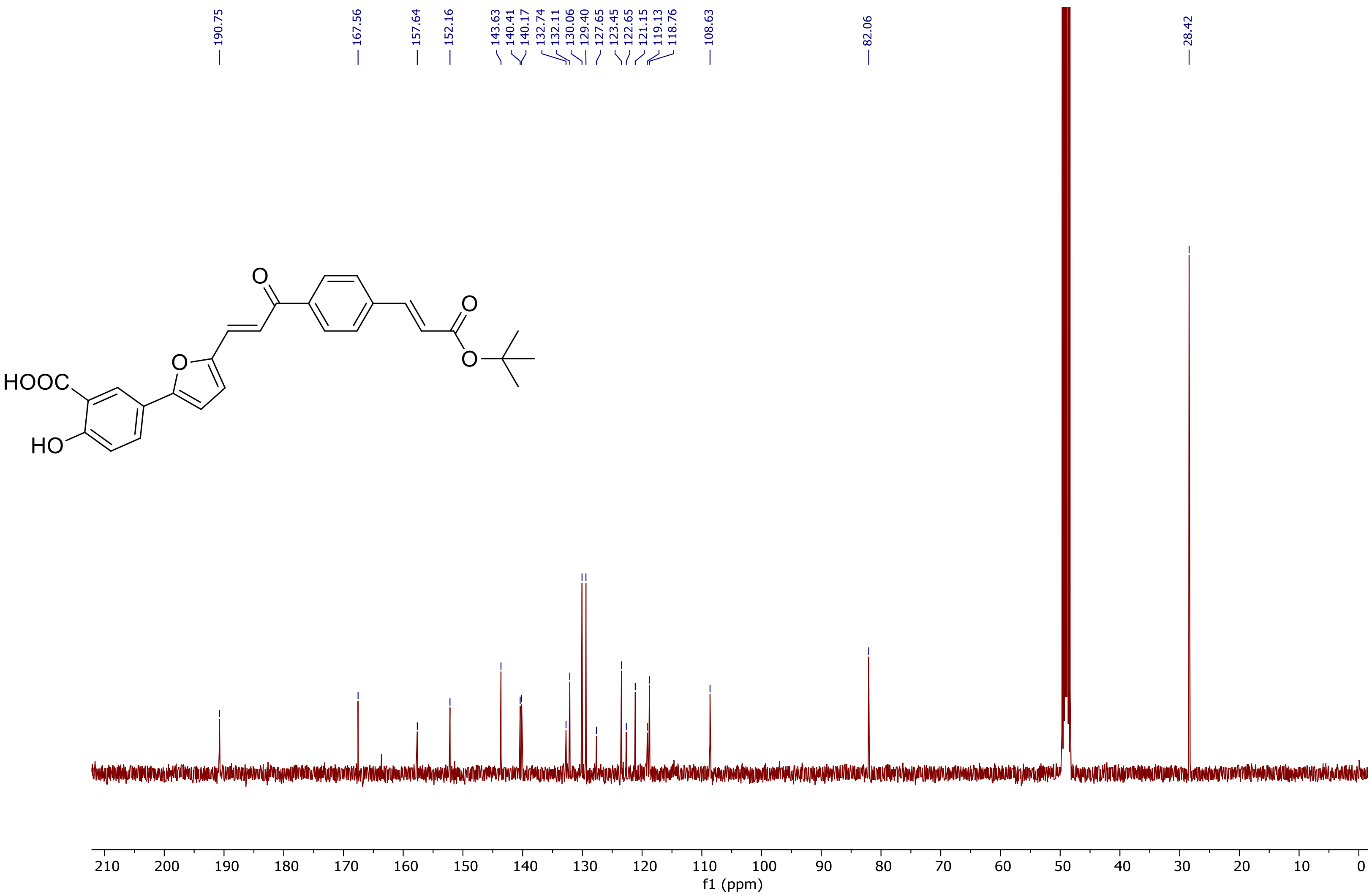

(*E*)-5-{5-{3-{4-[3-(*tert*-Butoxy)-3-oxopropyl]phenyl}-3-oxoprop-1-en-1-yl}furan-2-yl}-2-hydroxybenzoic acid (25)

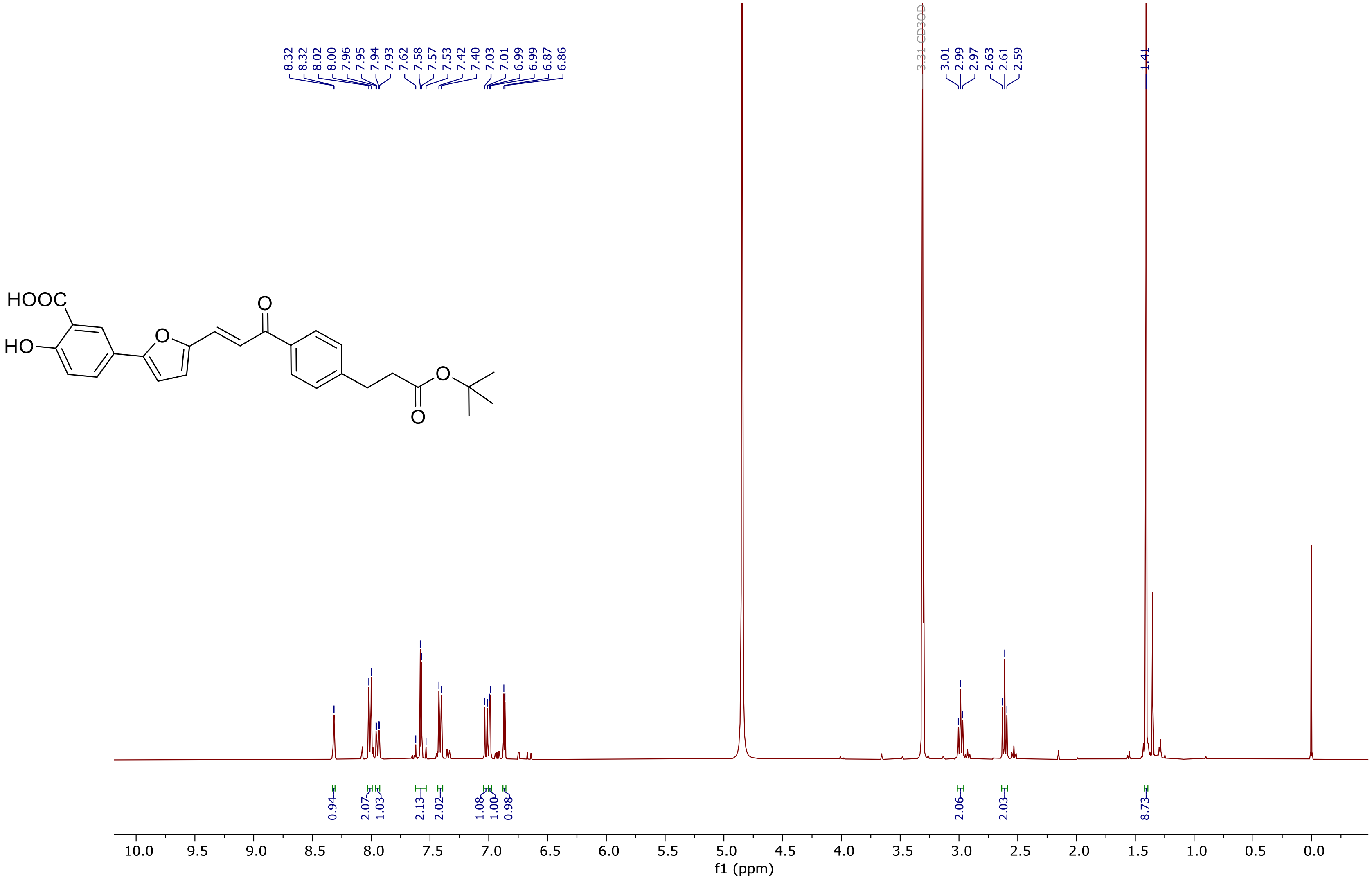

(*E*)-5-{5-{3-{4-[3-(*tert*-Butoxy)-3-oxopropyl]phenyl}-3-oxoprop-1-en-1-yl}furan-2-yl}-2-hydroxybenzoic acid (25)

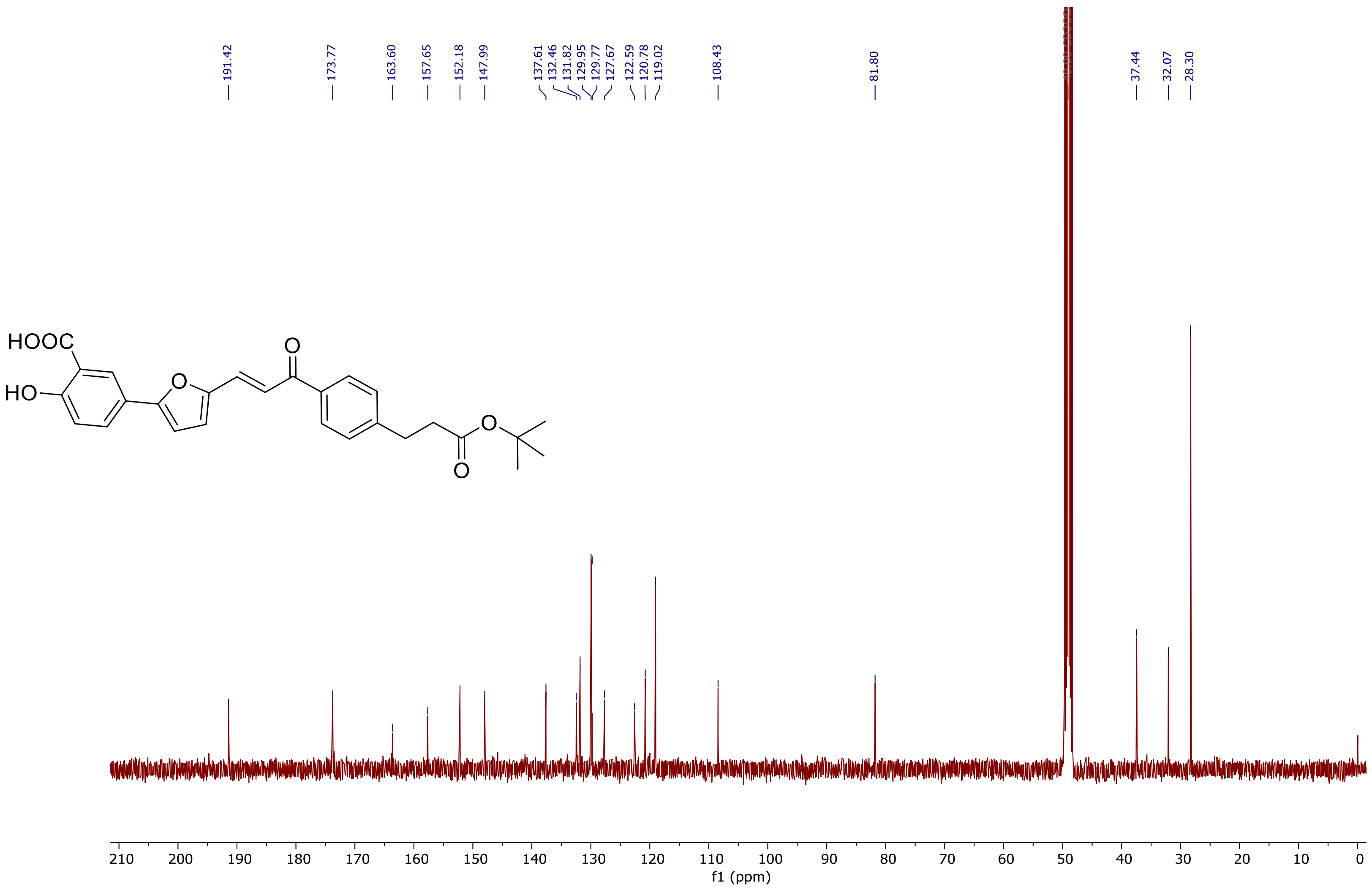

5-{5-{(*E*)-3-{4-[(*E*)-3-(adamantan-1-ylmethylamino)-3-oxoprop-1-enyl]phenyl}-3-oxoprop-1-enyl}furan-2-yl}-2-hydroxybenzoic acid (26)

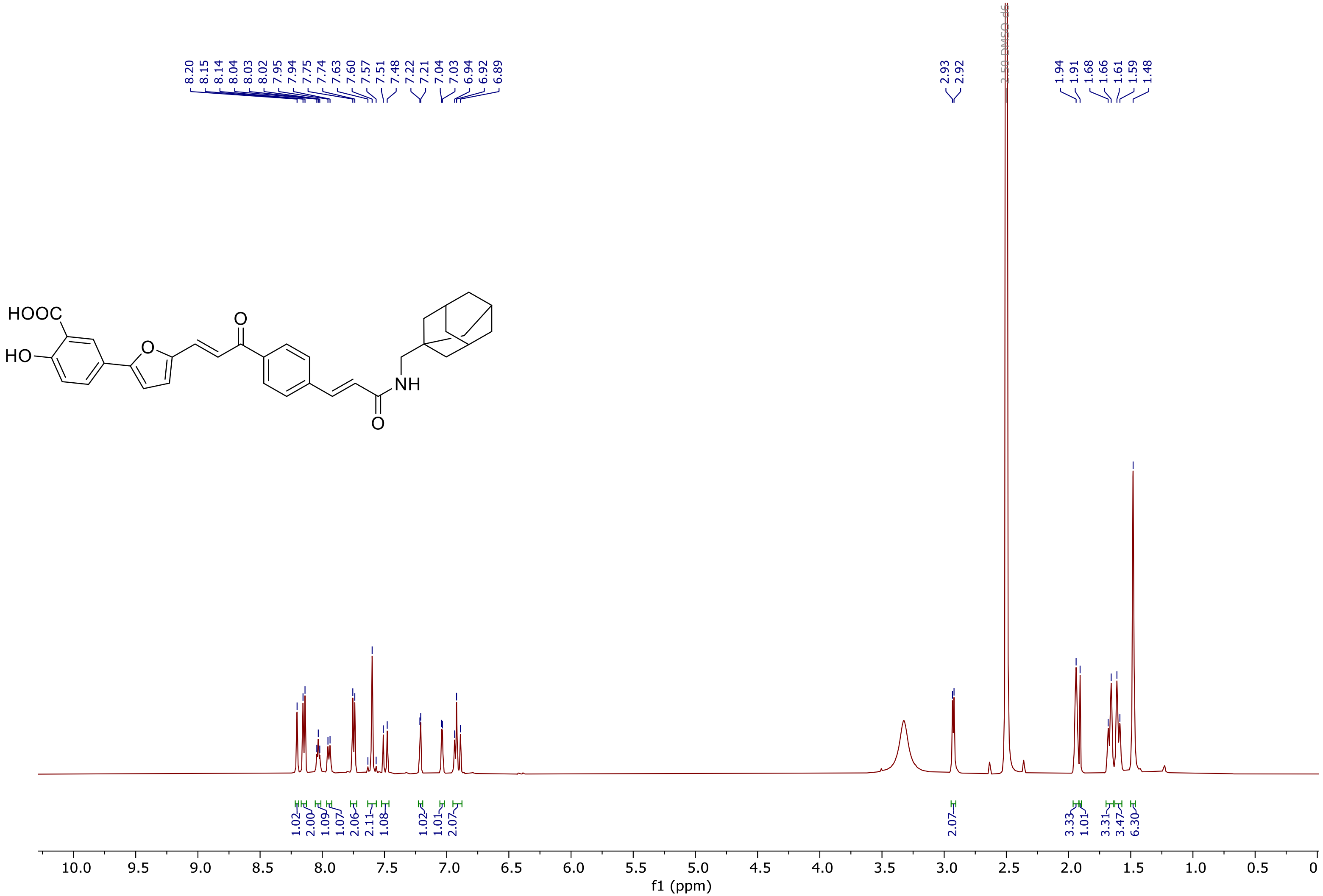

5-{5-{(*E*)-3-{4-[(*E*)-3-(adamantan-1-ylmethylamino)-3-oxoprop-1-enyl]phenyl}-3-oxoprop-1-enyl}furan-2-yl}-2-hydroxybenzoic acid (26)

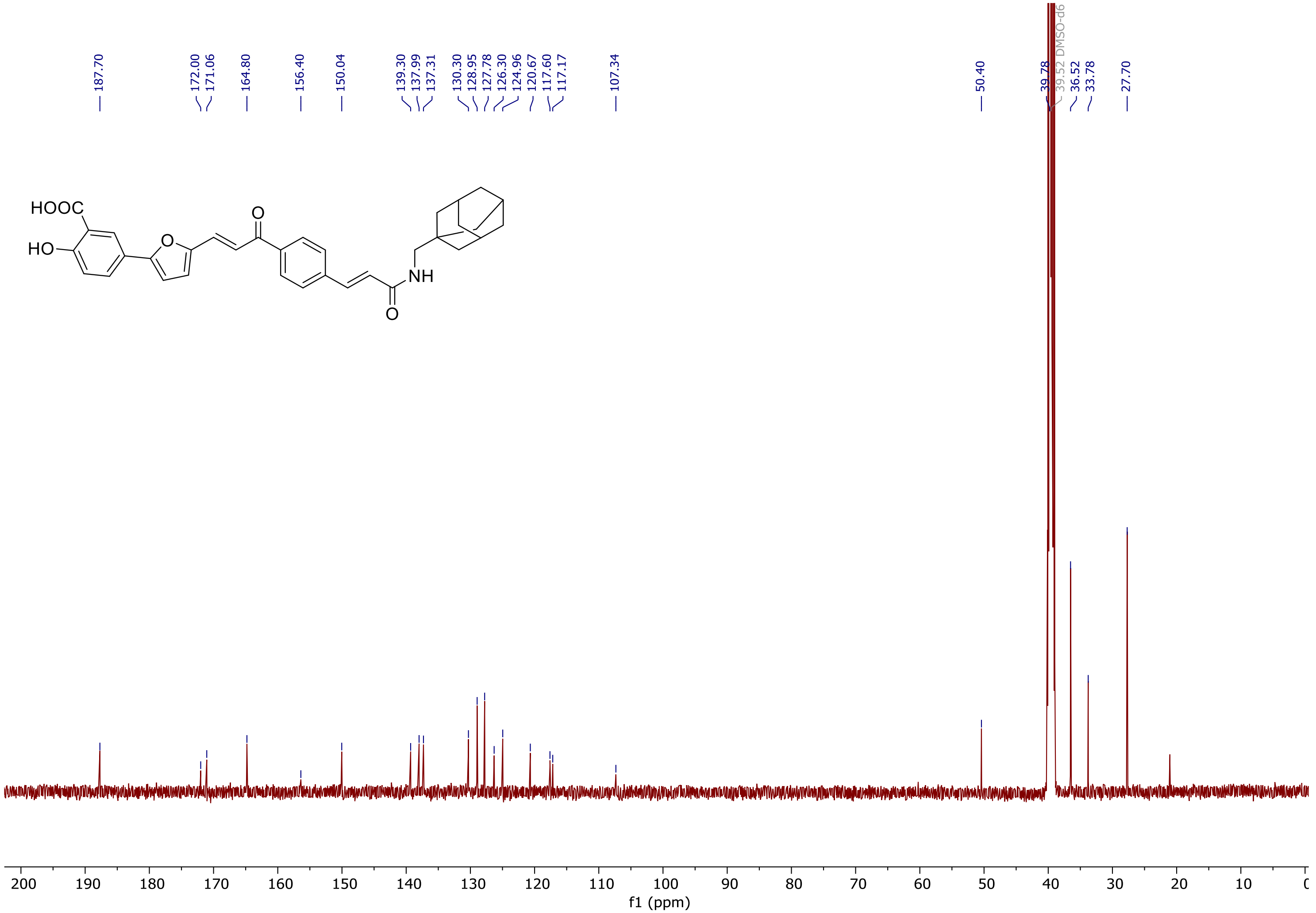

2-Hydroxy-5-{5-{3-oxo-3-[4-(trifluoromethyl)phenyl]propyl}furan-2-yl}benzoic acid (27)

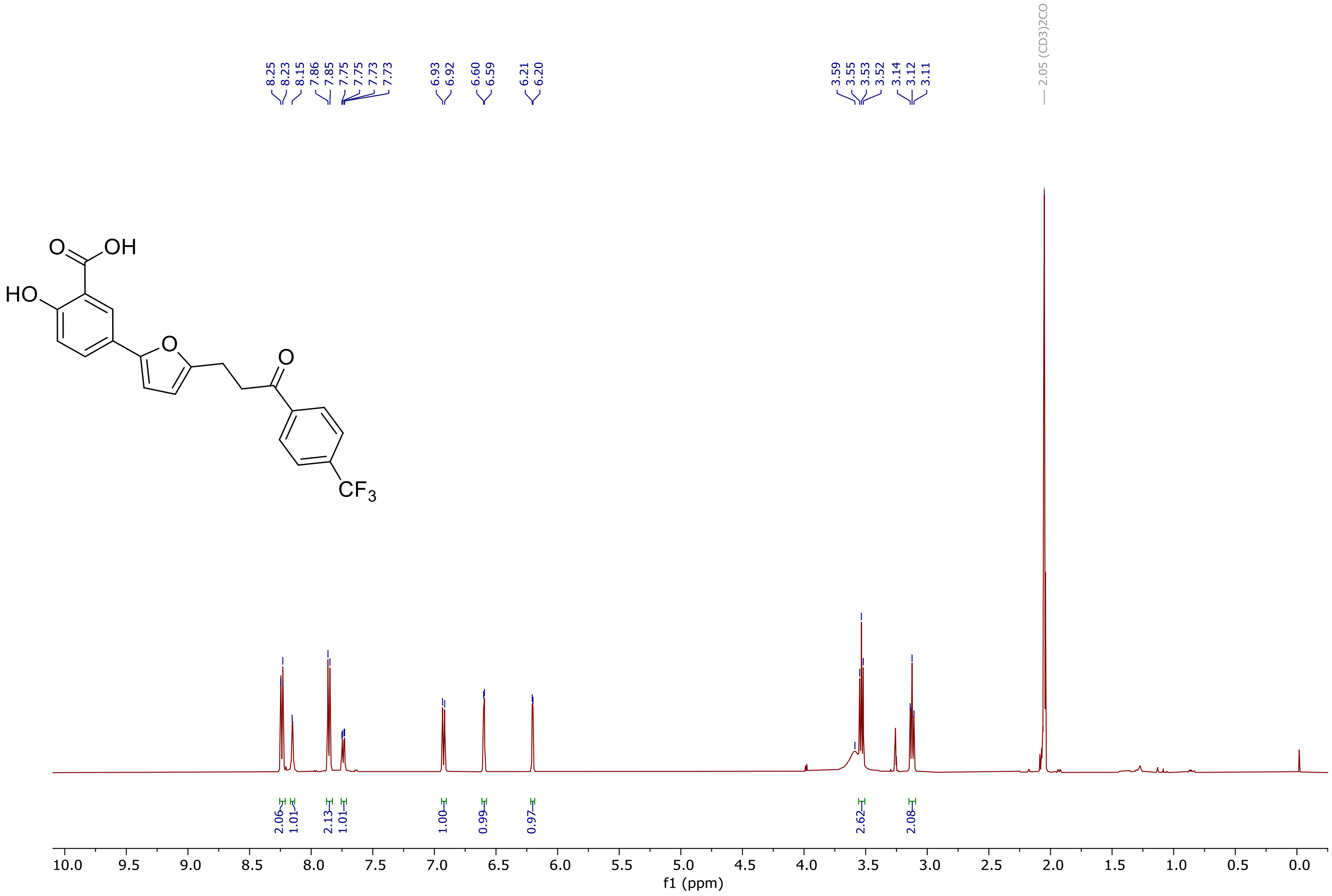

2-Hydroxy-5-{5-{3-oxo-3-[4-(trifluoromethyl)phenyl]propyl}furan-2-yl}benzoic acid (27).

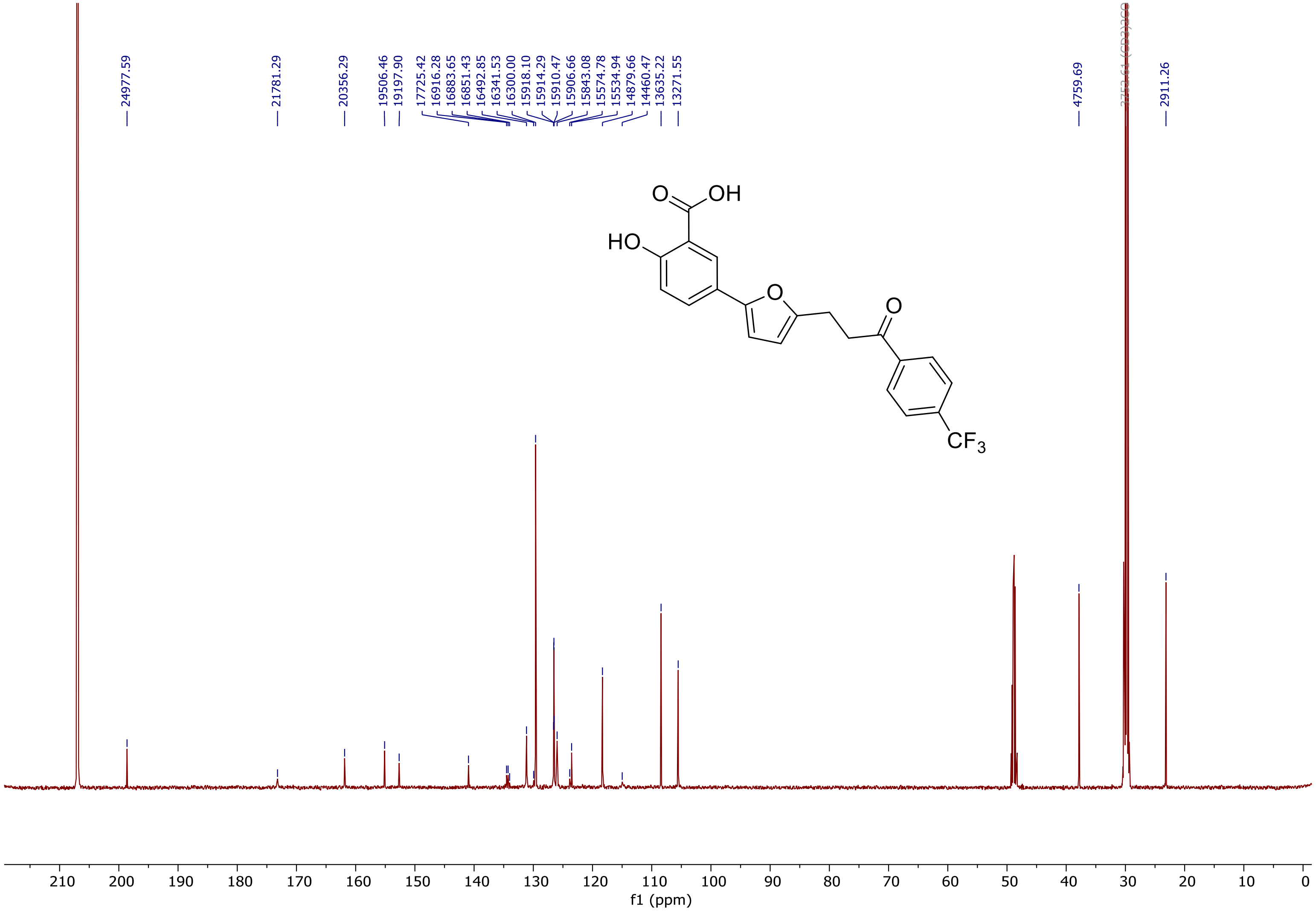

5-{5-[3-(4-Butylphenyl)-3-oxopropyl]furan-2-yl}-2-hydroxybenzoic acid (28)

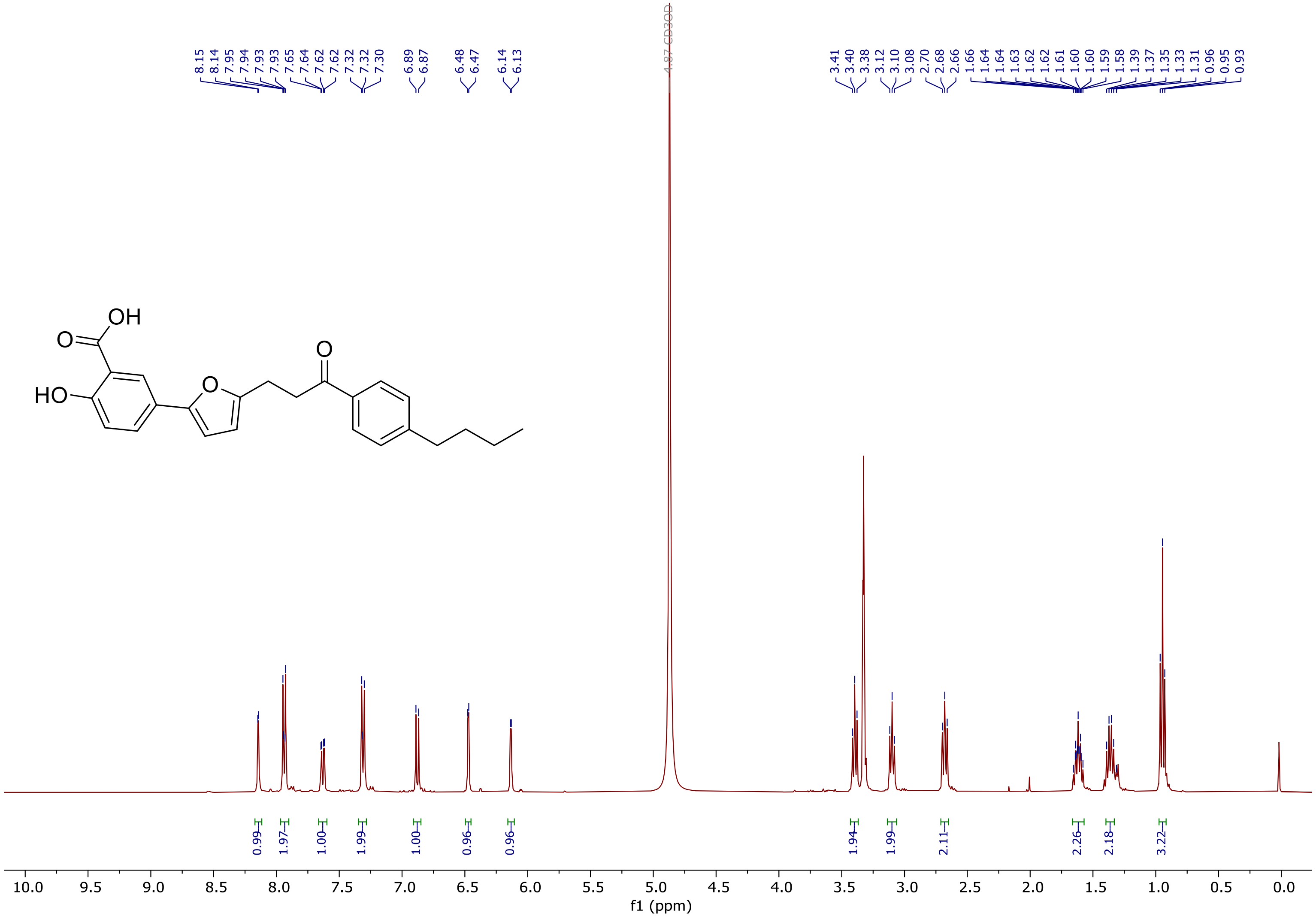

5-{5-[3-(4-Butylphenyl)-3-oxopropyl]furan-2-yl}-2-hydroxybenzoic acid (28)

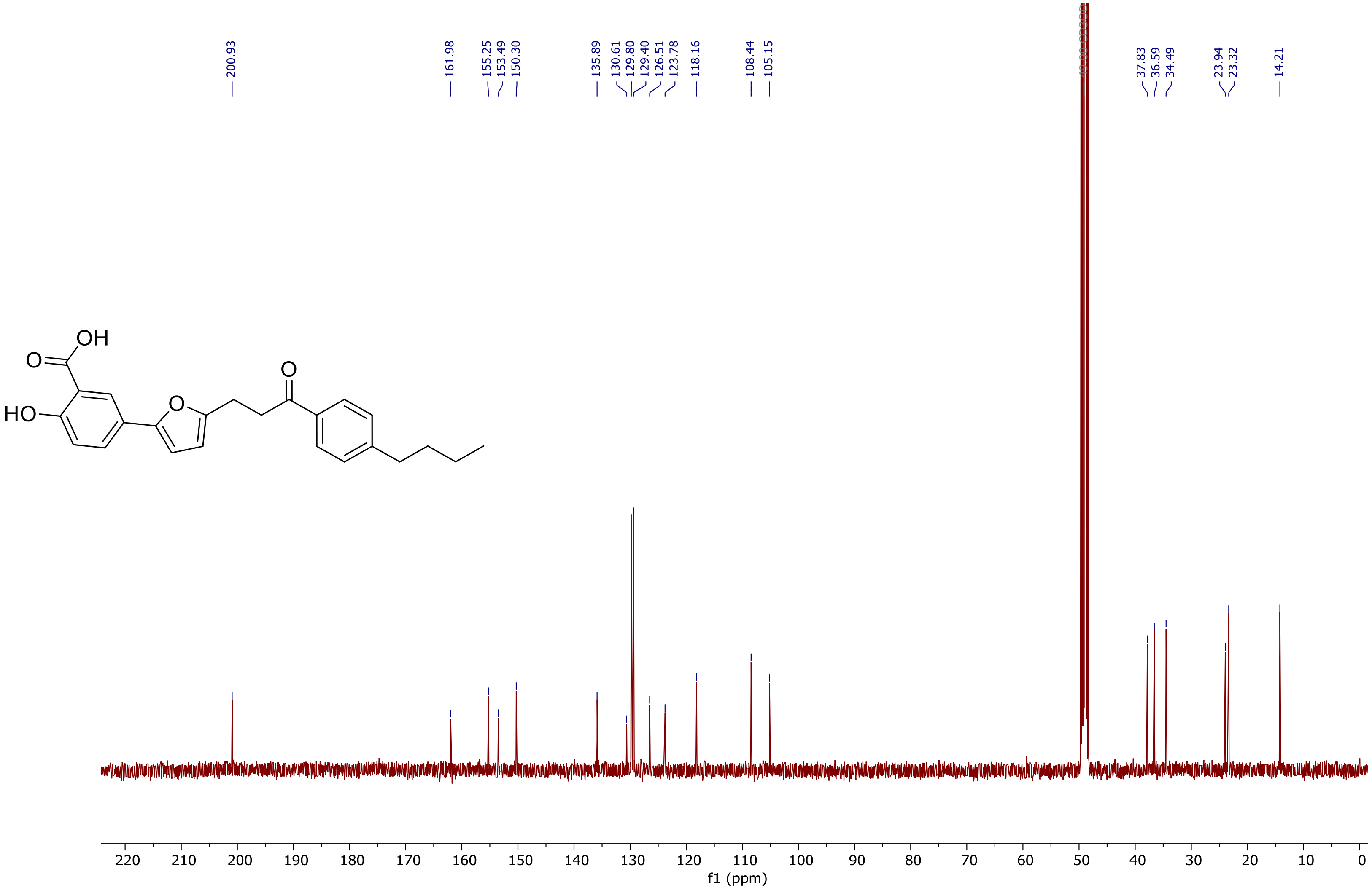

5-{5-[3-(4-Butylphenyl)-3-hydroxypropyl]furan-2-yl}-2-hydroxybenzoic acid (29)

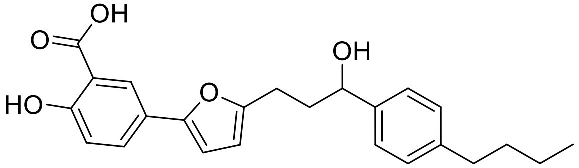

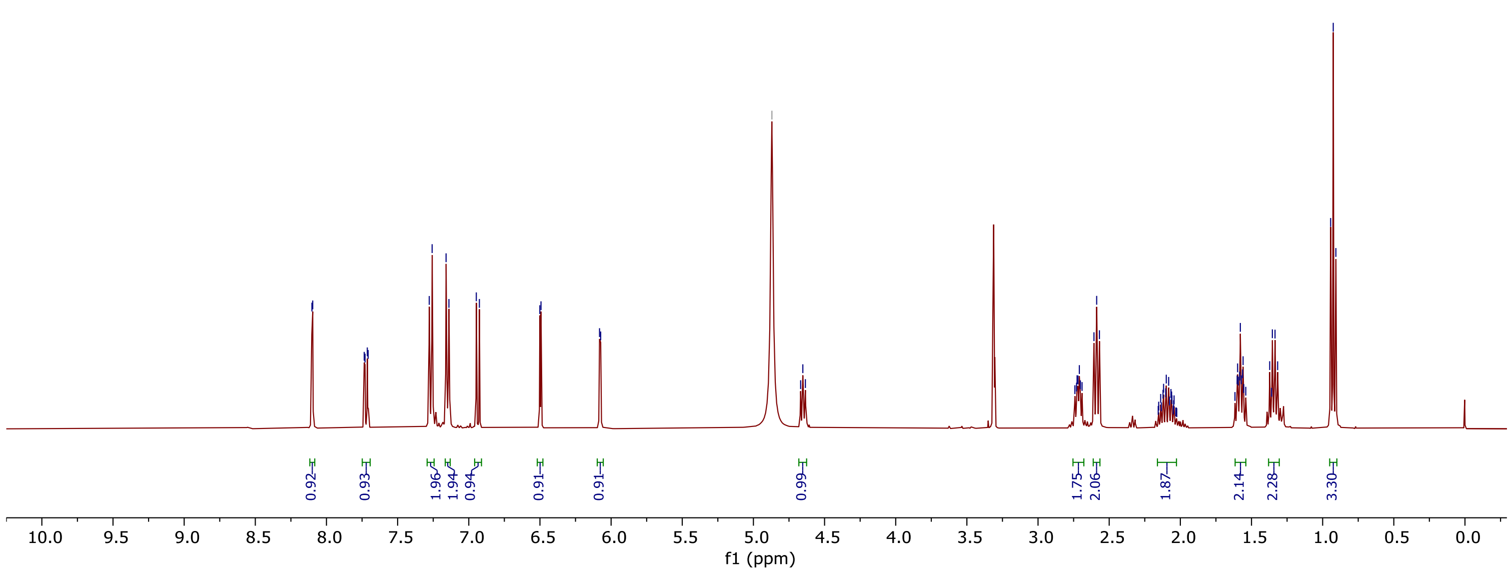

5-{5-[3-(4-Butylphenyl)-3-hydroxypropyl]furan-2-yl}-2-hydroxybenzoic acid (29)

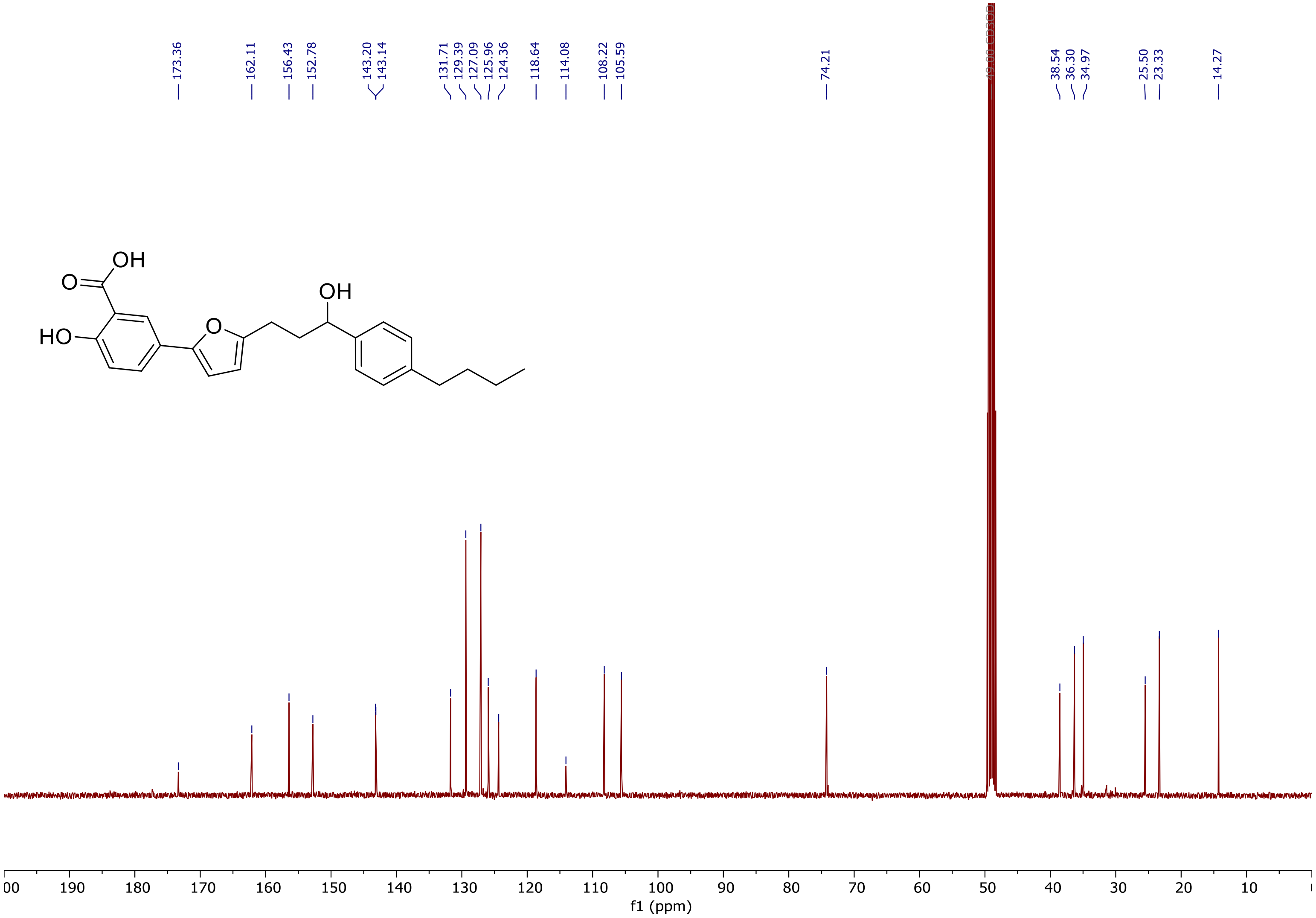

Methyl 5-(5-formylfuran-2-yl)-2-hydroxybenzoate (32)

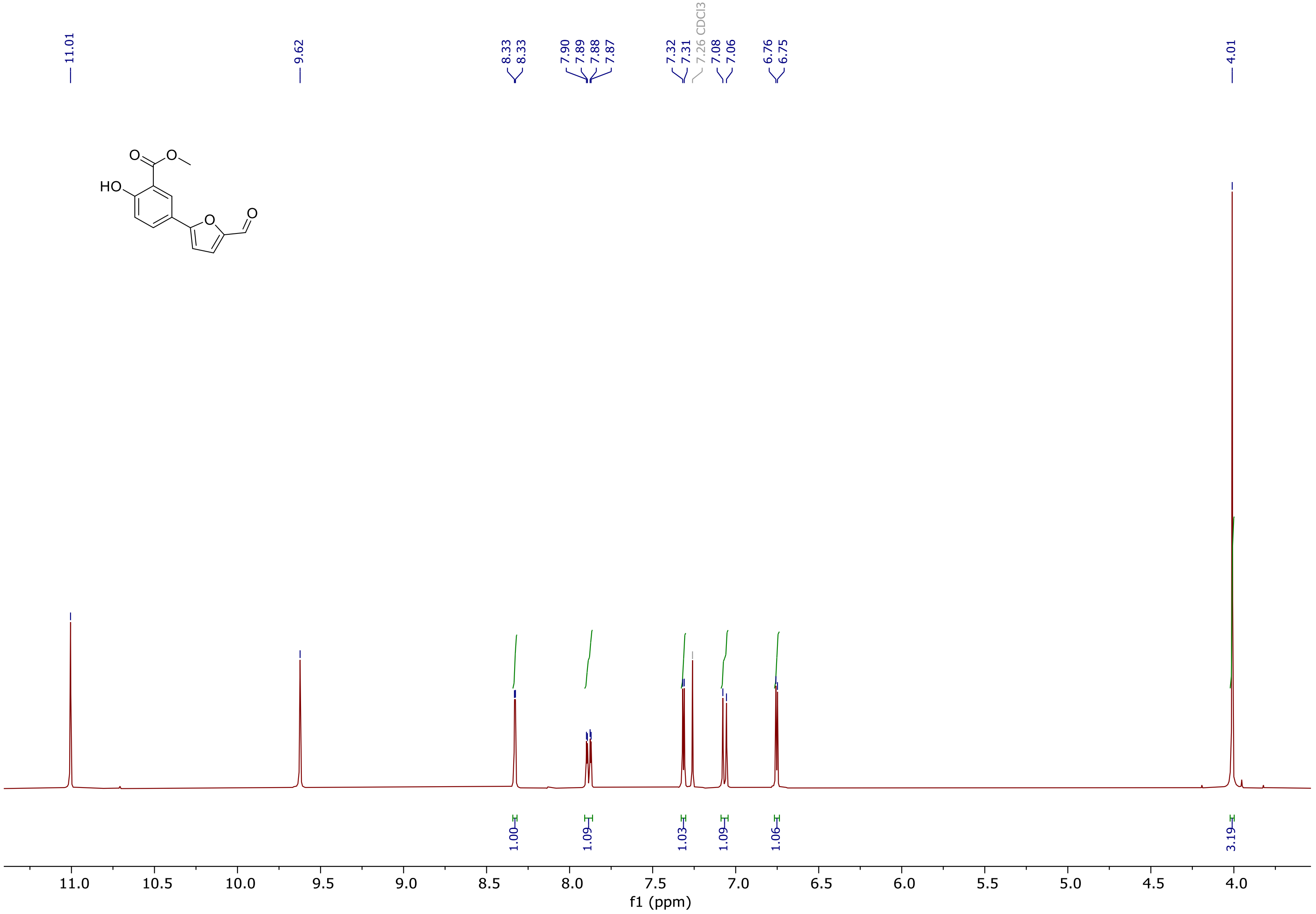

N-(Adamantan-1-ylmethyl)hex-5-yn-1-amine (35)

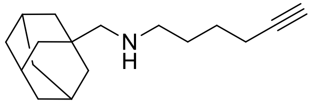

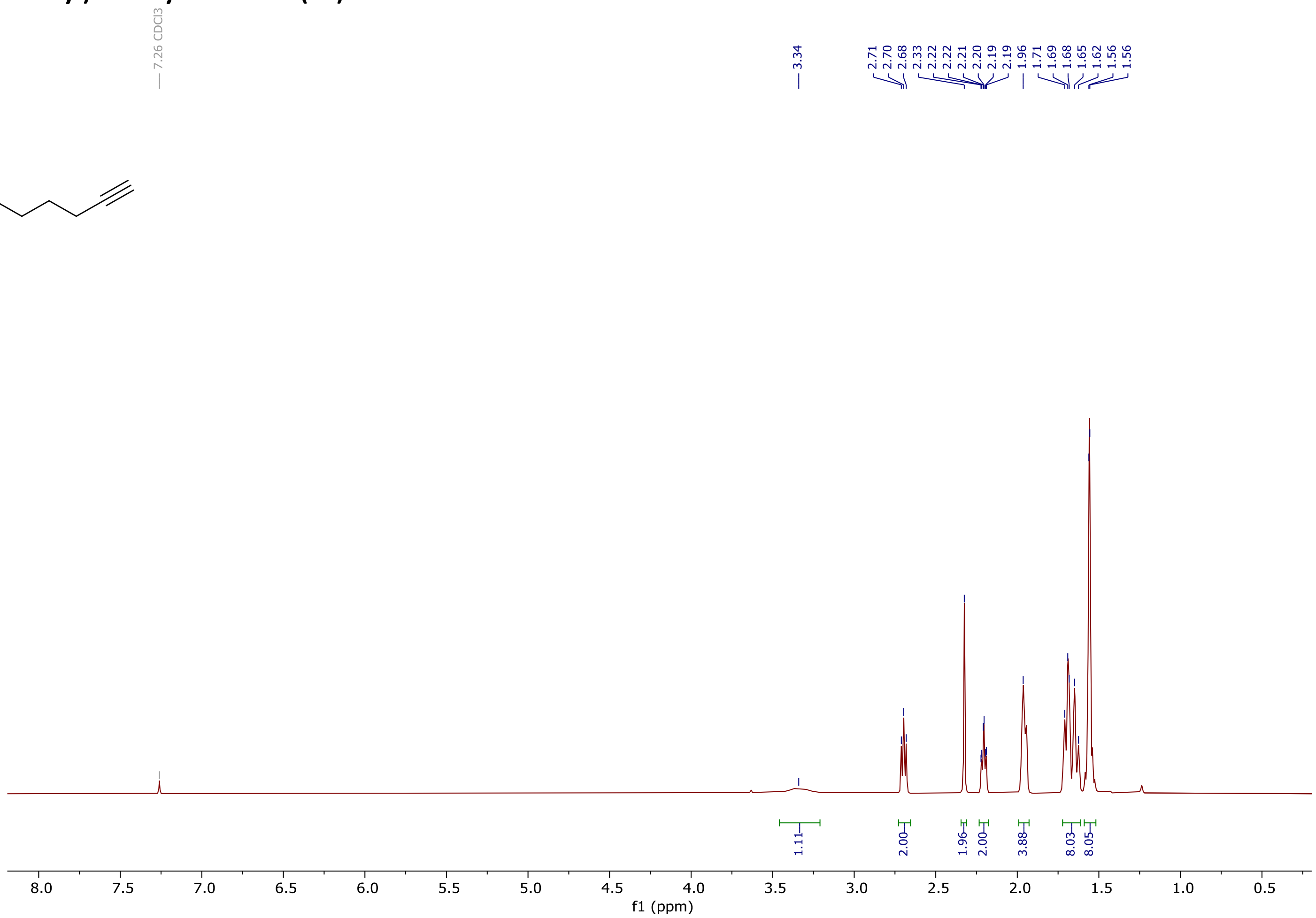

N-(Adamantan-1-ylmethyl)hex-5-yn-1-amine (35)

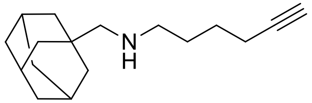

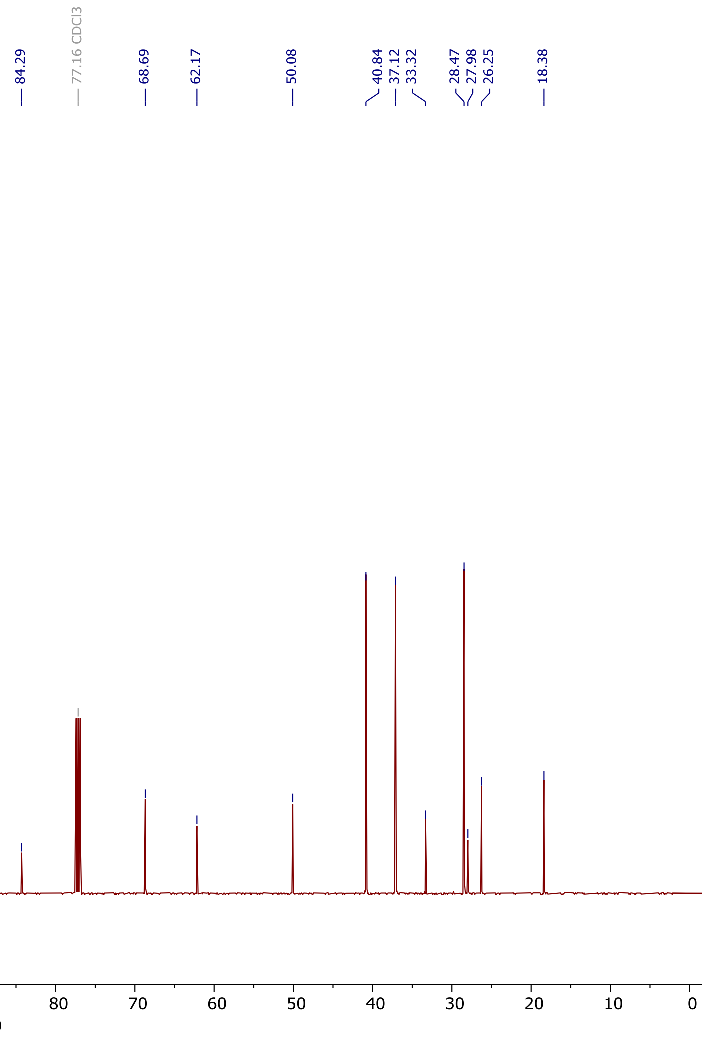

tert-Butyl (_E_)-3-(4-acetylphenyl)acrylate (33-Acrylate-tBu)

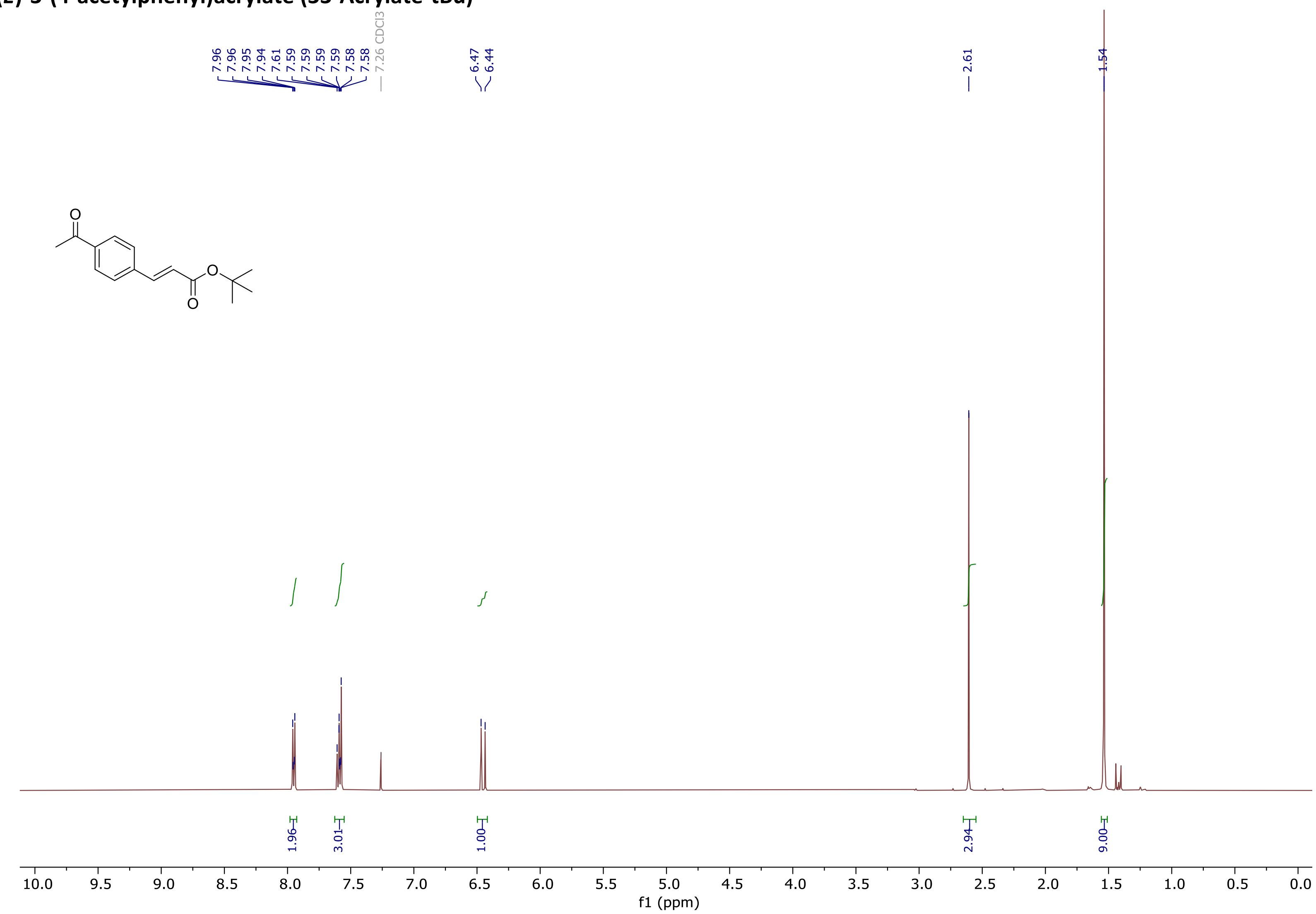

***tert*-Butyl (*E*)-3-(4-acetylphenyl)acrylate (33-Acrylate-tBu)**

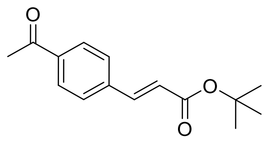

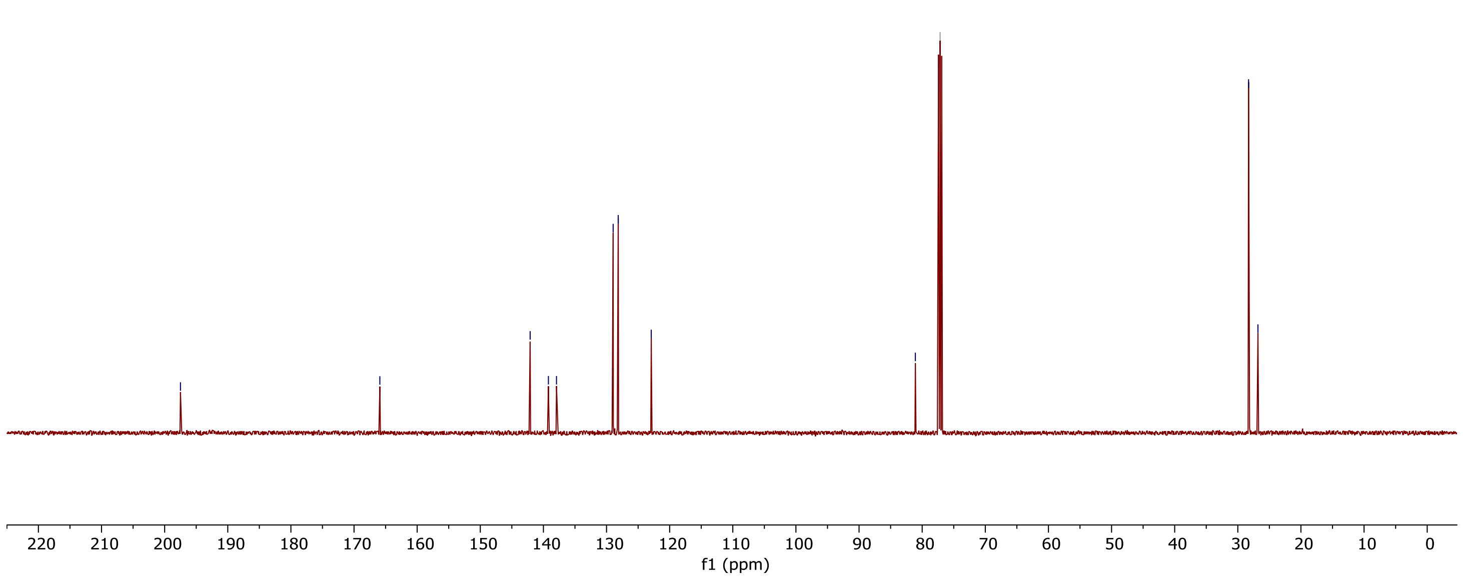

***tert*-Butyl 3-(4-acetylphenyl)propanoate (33-Propionate-tBu)**

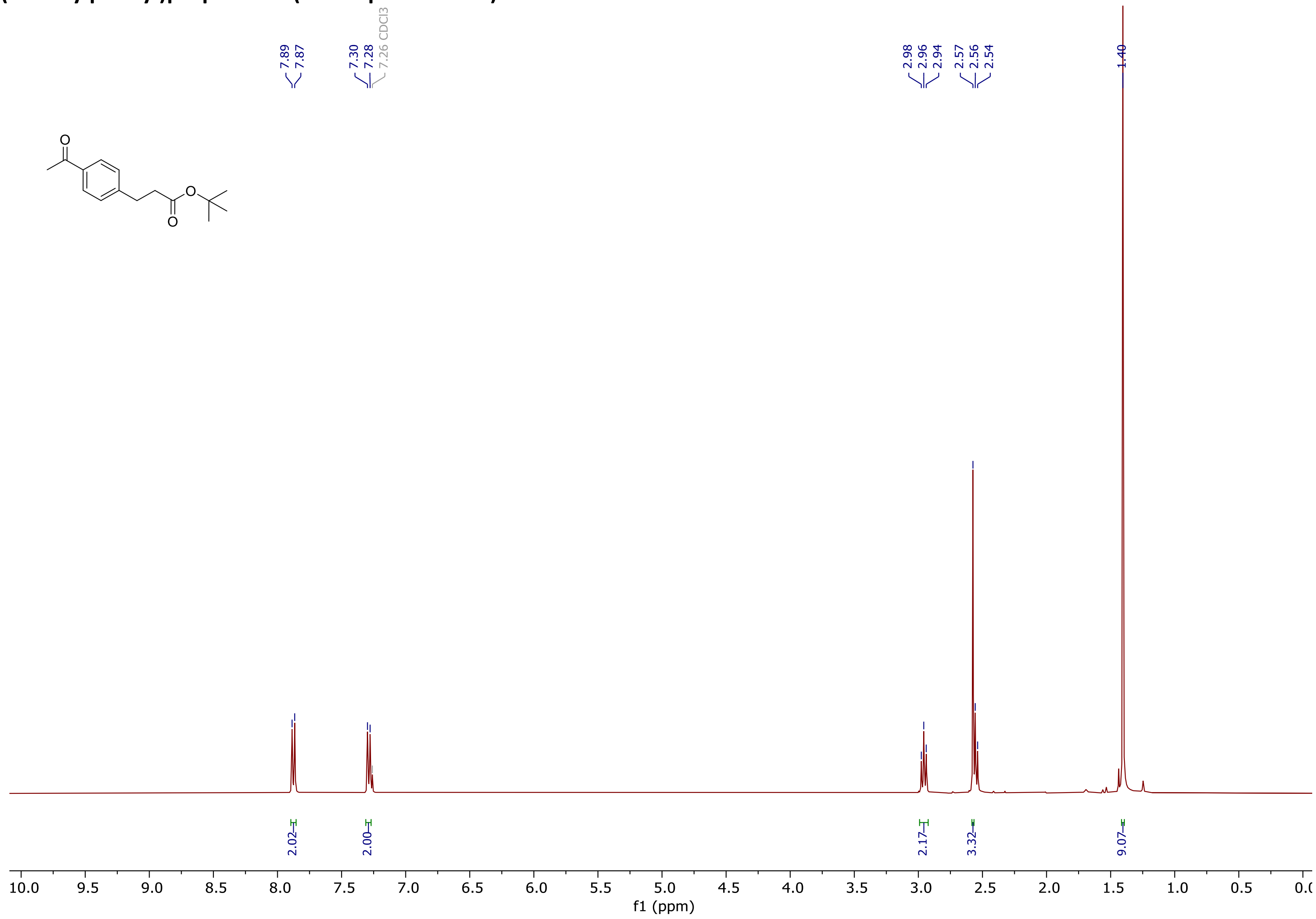

Methyl 5-{5-{(*E*)-3-{4-[6-(adamantan-1-ylmethylamino)hex-1-ynyl]phenyl}-3-oxoprop-1-enyl}furan-2-yl}-2-hydroxybenzoate (34-Ad)

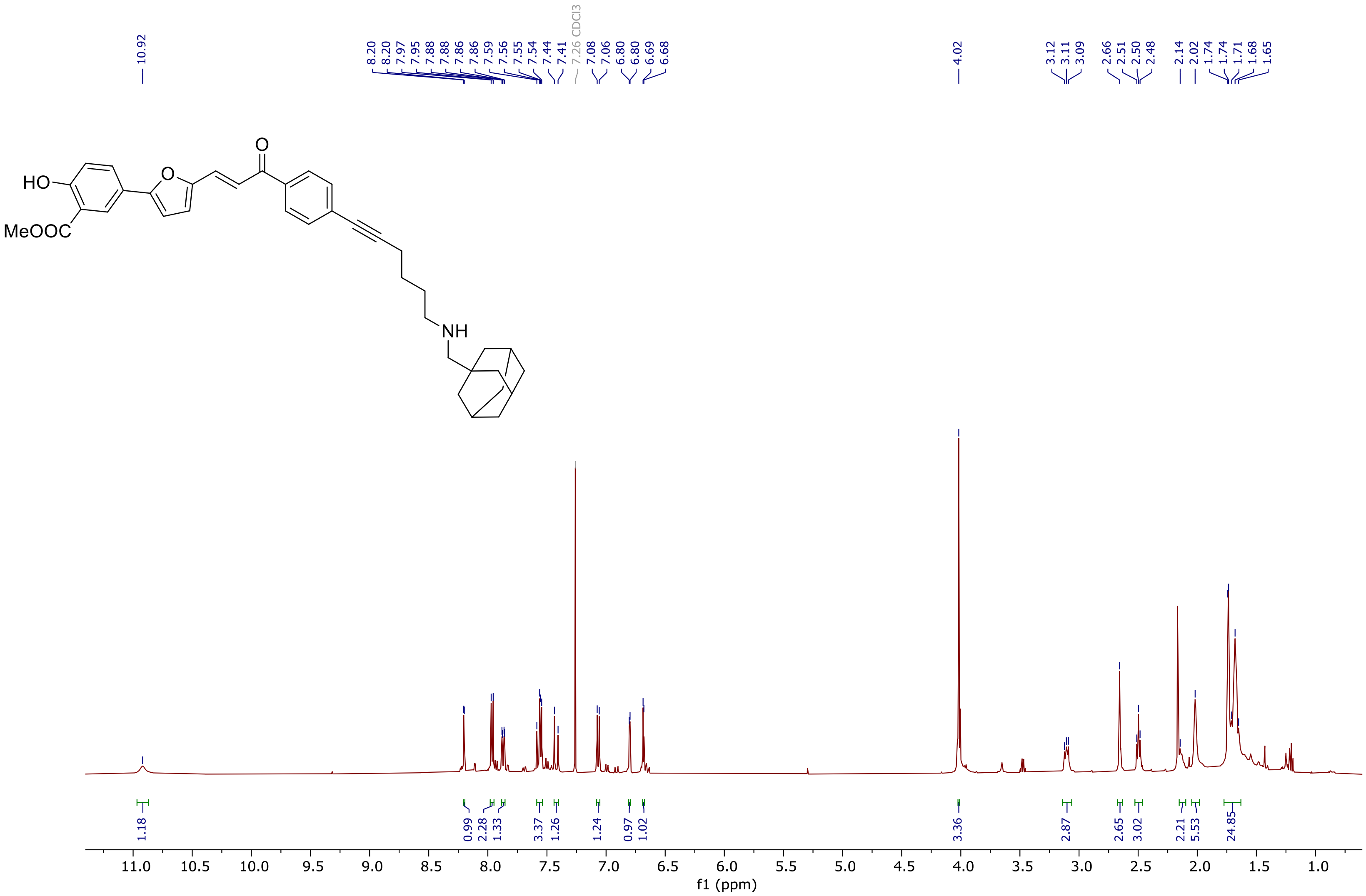

Methyl 5-{5-{(*E*)-3-{4-[6-(adamantan-1-ylmethylamino)hex-1-ynyl]phenyl}-3-oxoprop-1-enyl}furan-2-yl}-2-hydroxybenzoate (34-Ad)

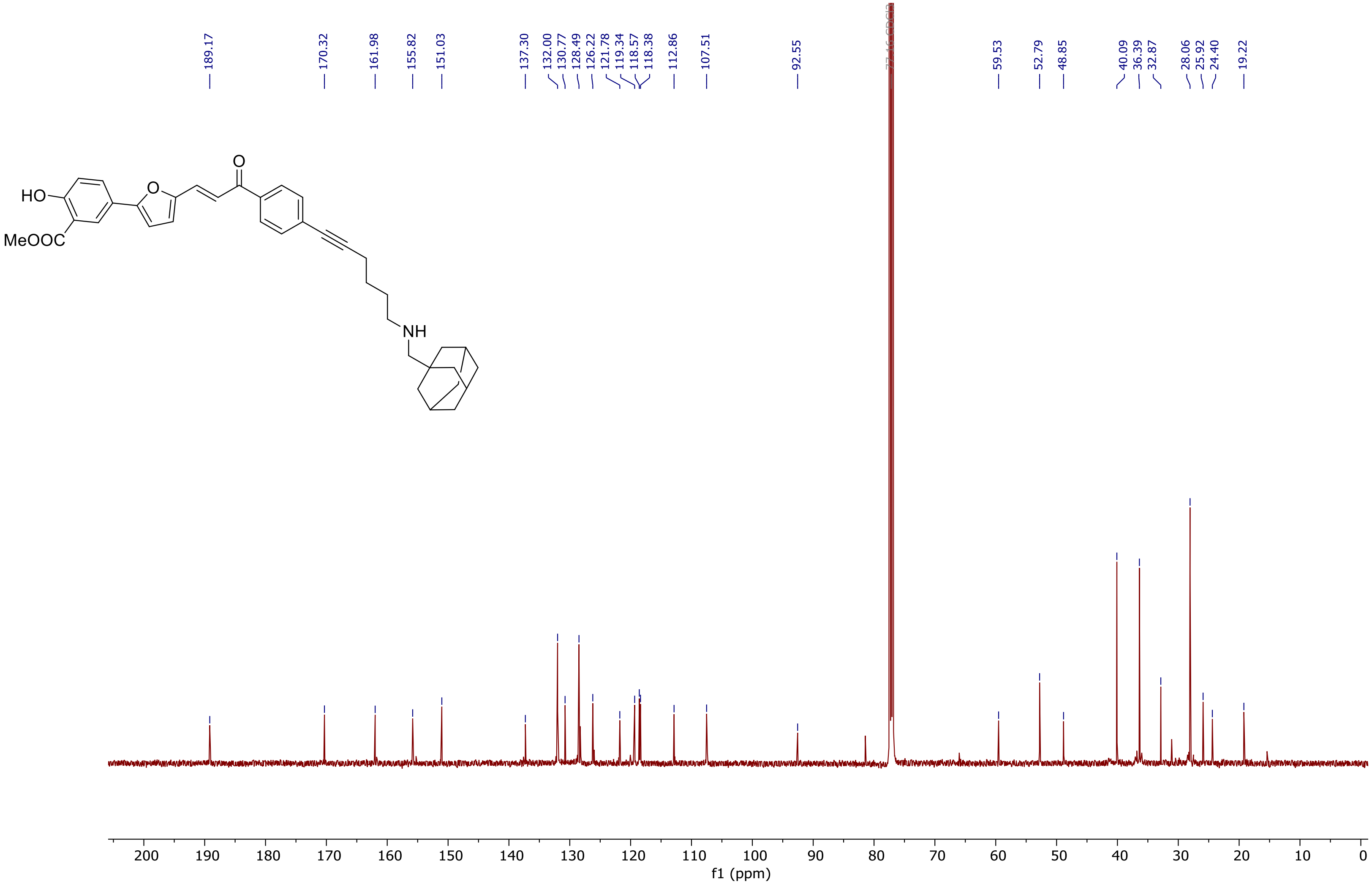

Methyl (*E*)-5-{5-[3-(4-cyanophenyl)-3-oxoprop-1-en-1-yl]furan-2-yl}-2-hydroxybenzoate (34-CN)

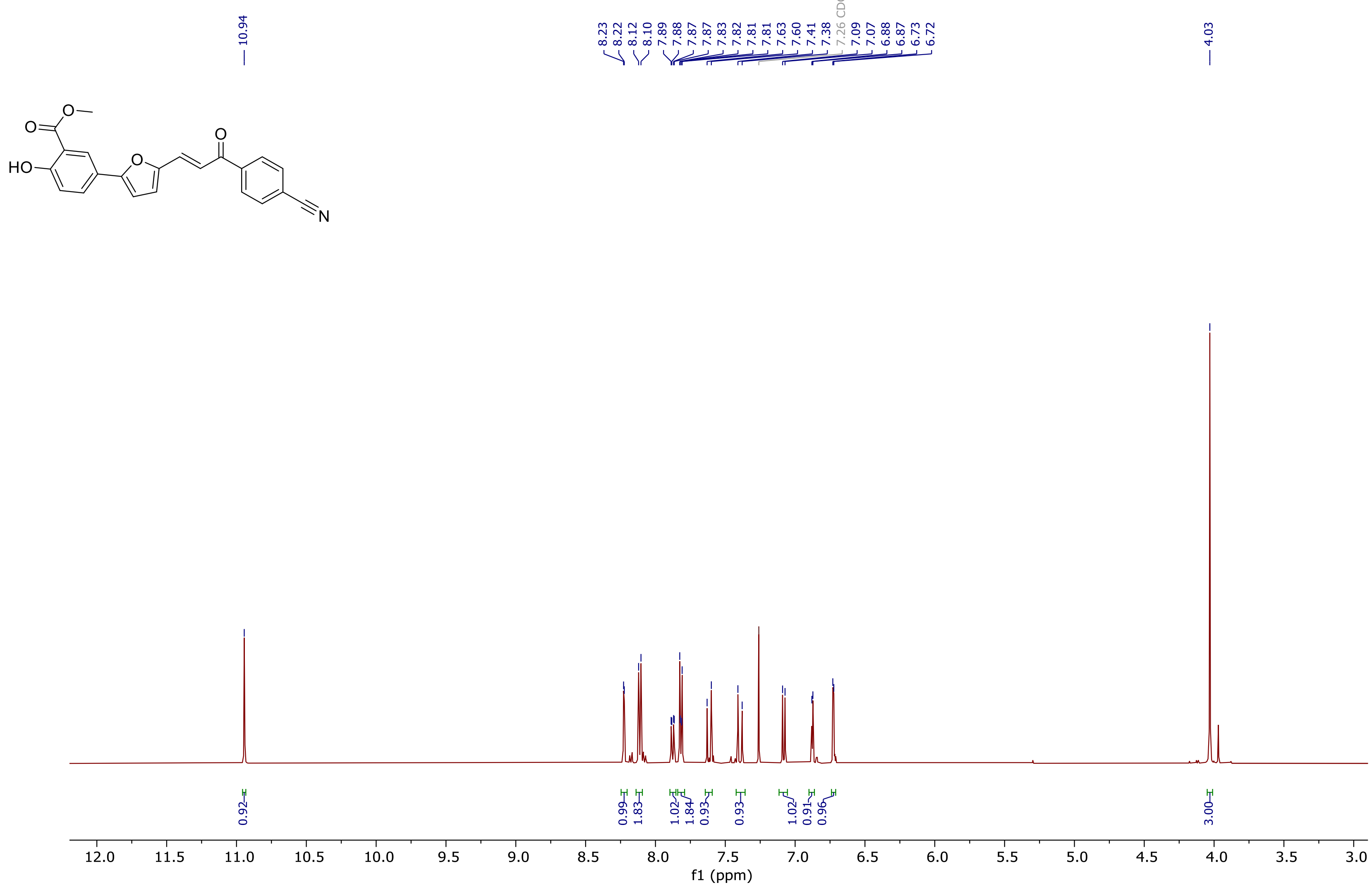

Methyl (*E*)-5-{5-[3-(4-cyanophenyl)-3-oxoprop-1-en-1-yl]furan-2-yl}-2-hydroxybenzoate (34-CN)

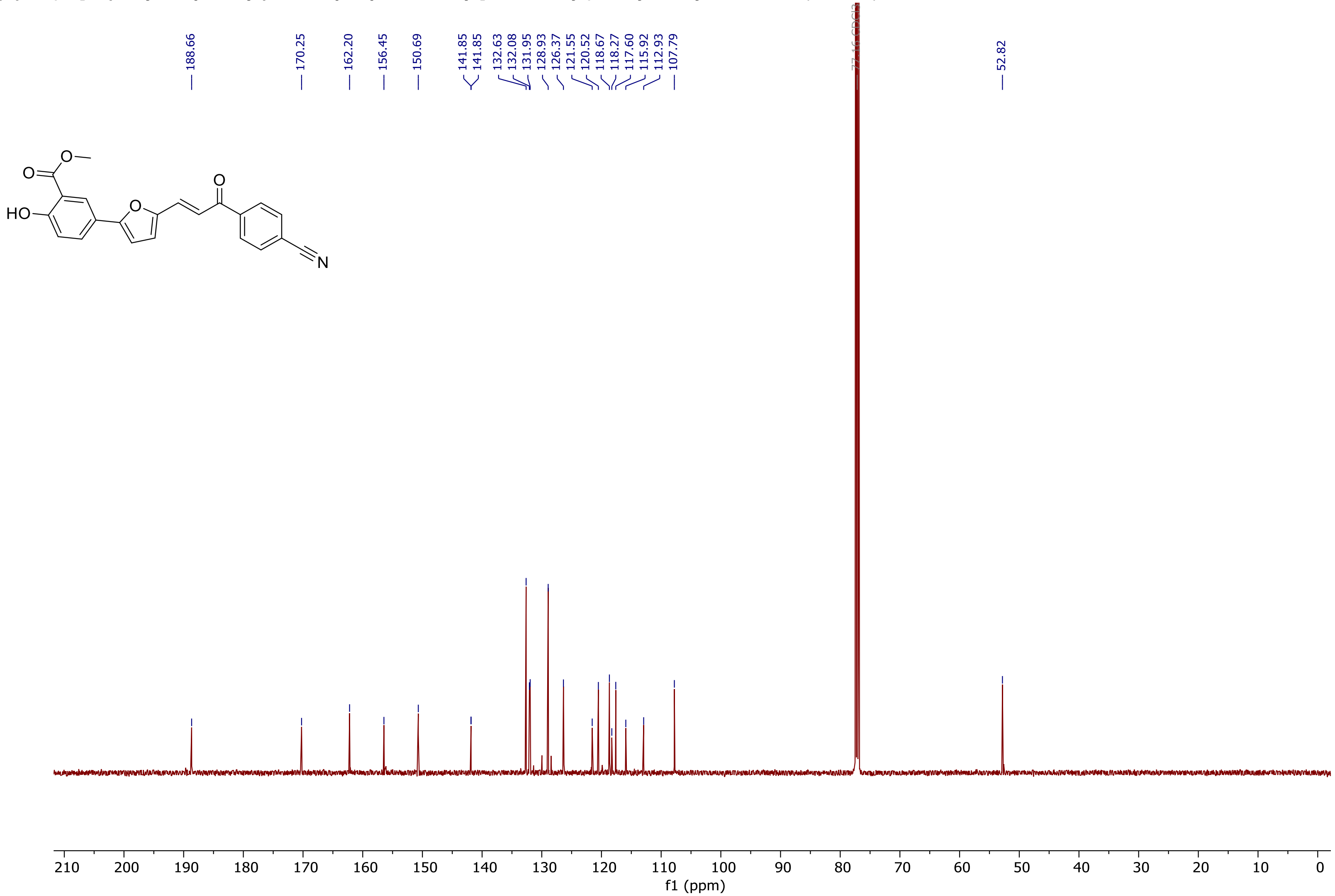

Methyl (*E*)-2-hydroxy-5-{5-{3-[4-(hydroxymethyl)phenyl]-3-oxoprop-1-en-1-yl}furan-2-yl}benzoate (34-CH_2OH)

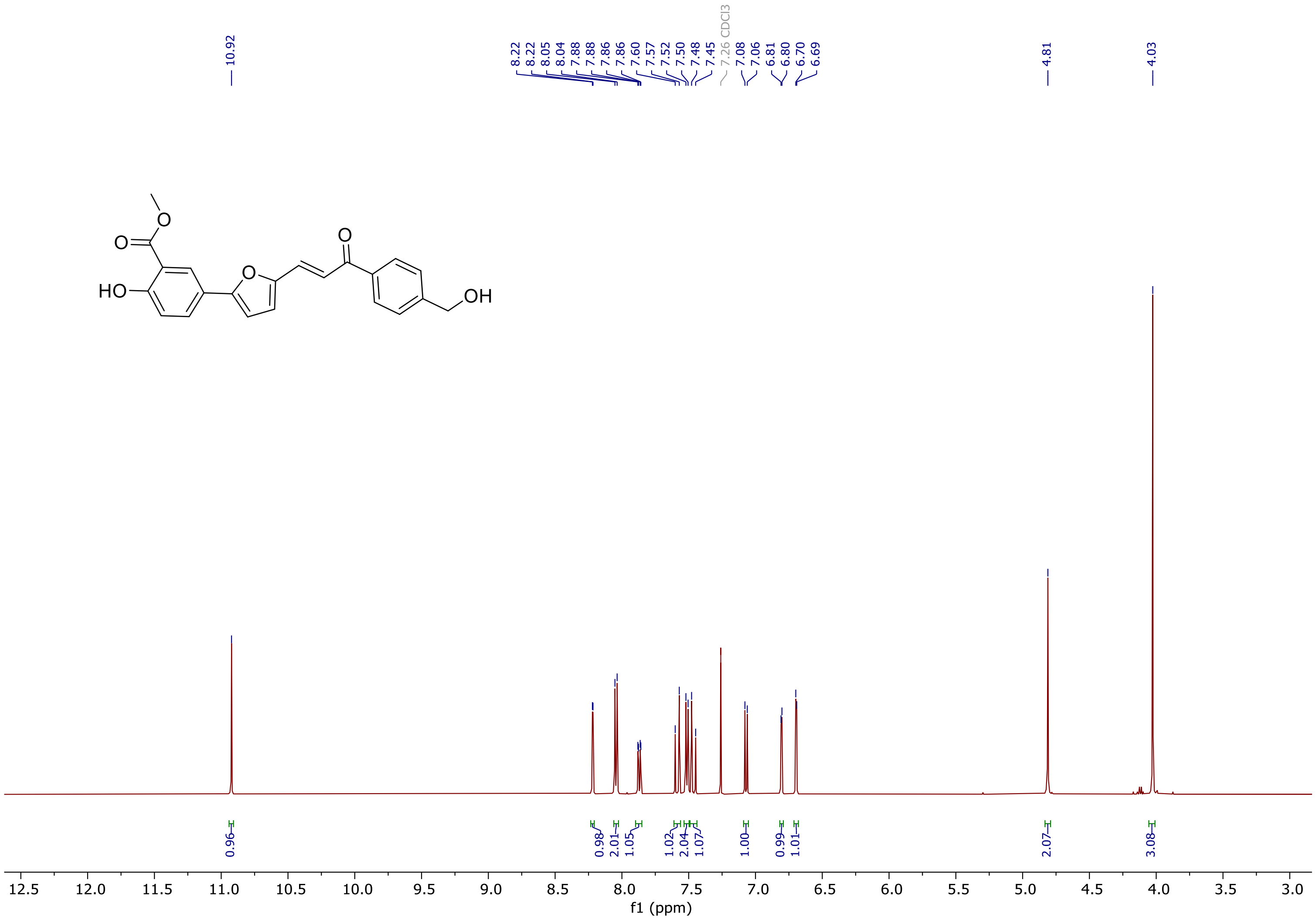

Methyl (*E*)-2-hydroxy-5-{5-{3-[4-(hydroxymethyl)phenyl]-3-oxoprop-1-en-1-yl}furan-2-yl}benzoate (34-CH$_2$OH)

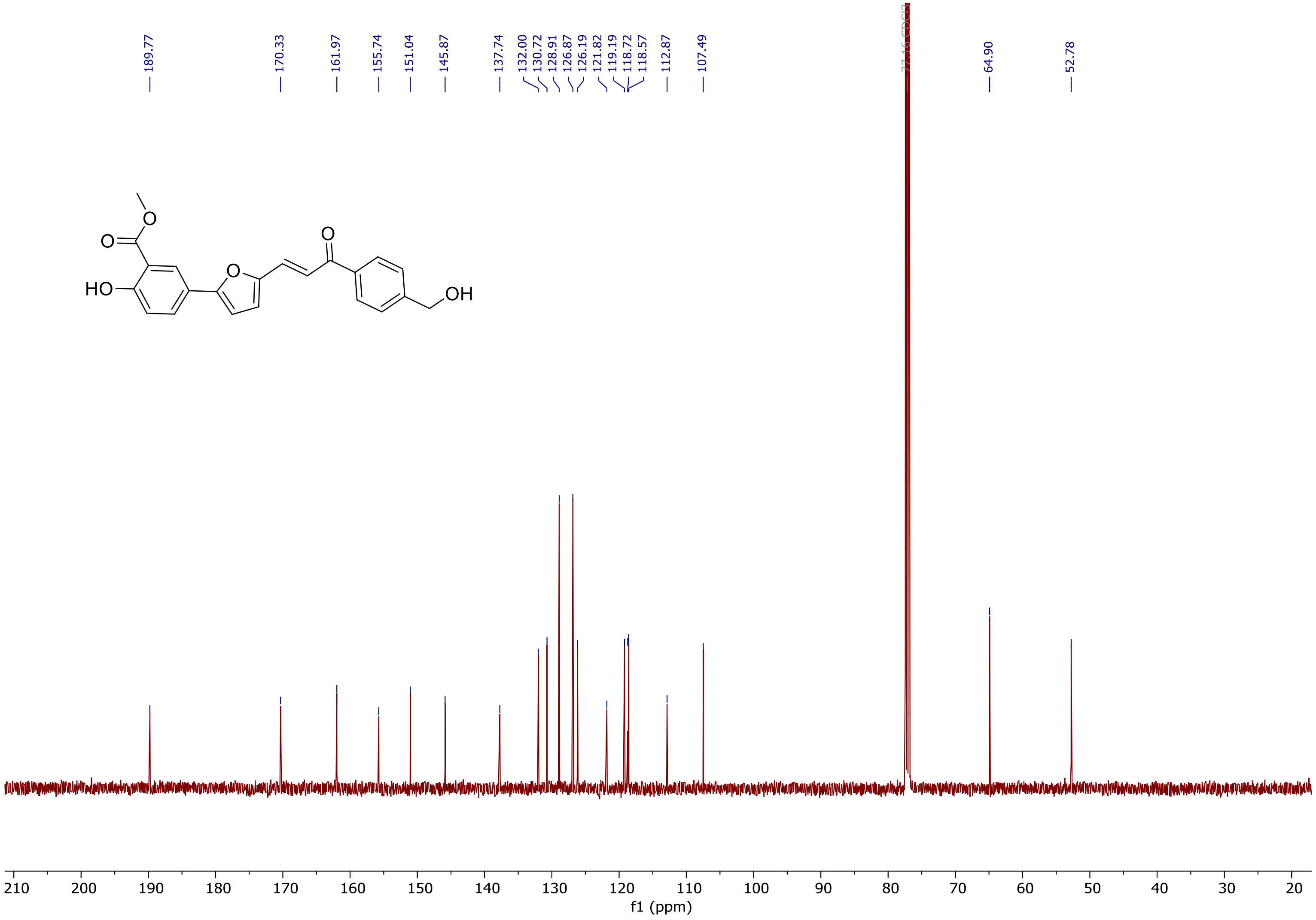

Methyl (*E*)-2-hydroxy-5-{5-[3-(4-iodophenyl)-3-oxoprop-1-en-1-yl]furan-2-yl}benzoate (34-I)

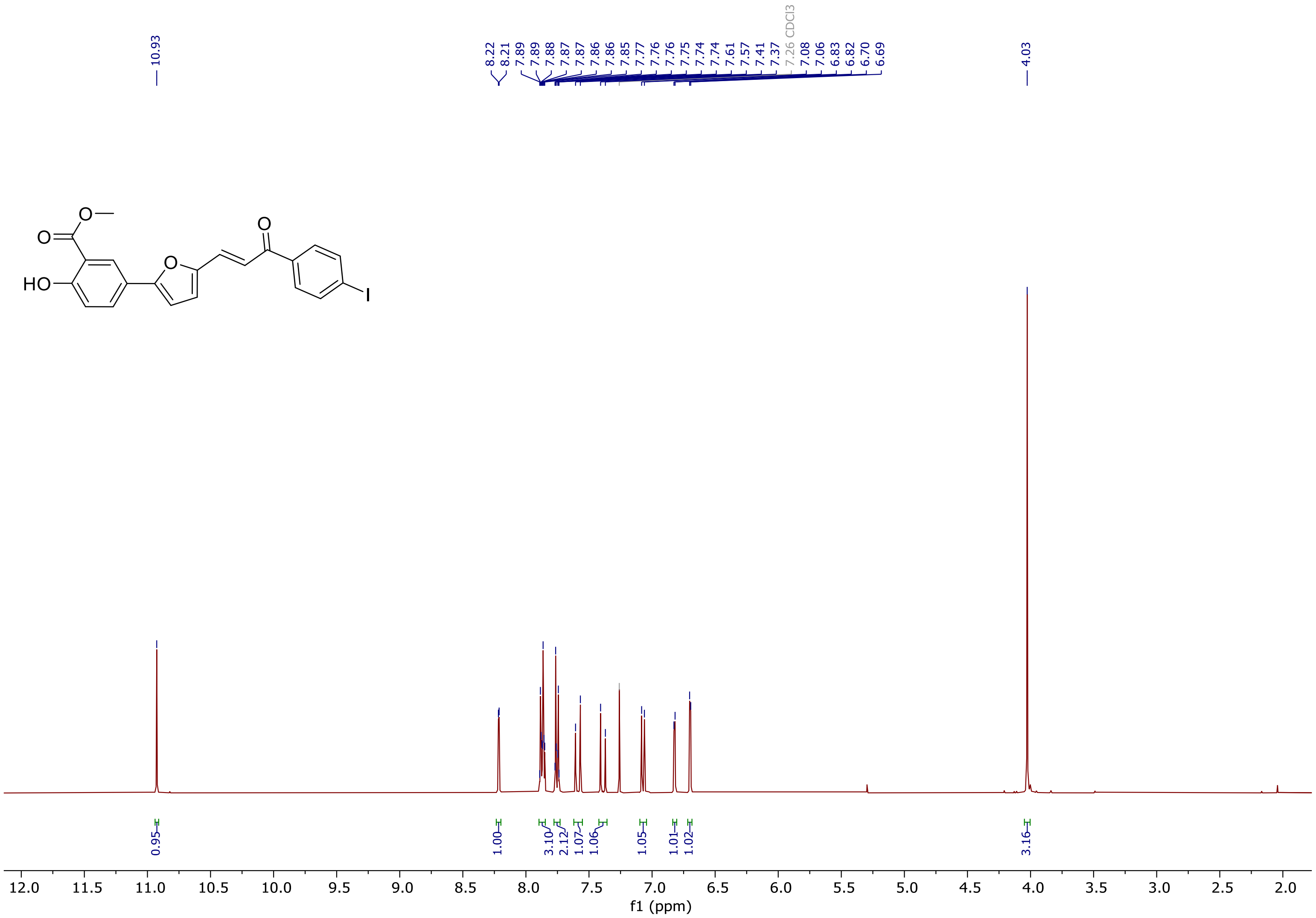

Methyl (*E*)-2-hydroxy-5-{5-[3-(4-iodophenyl)-3-oxoprop-1-en-1-yl]furan-2-yl}benzoate (34-I)

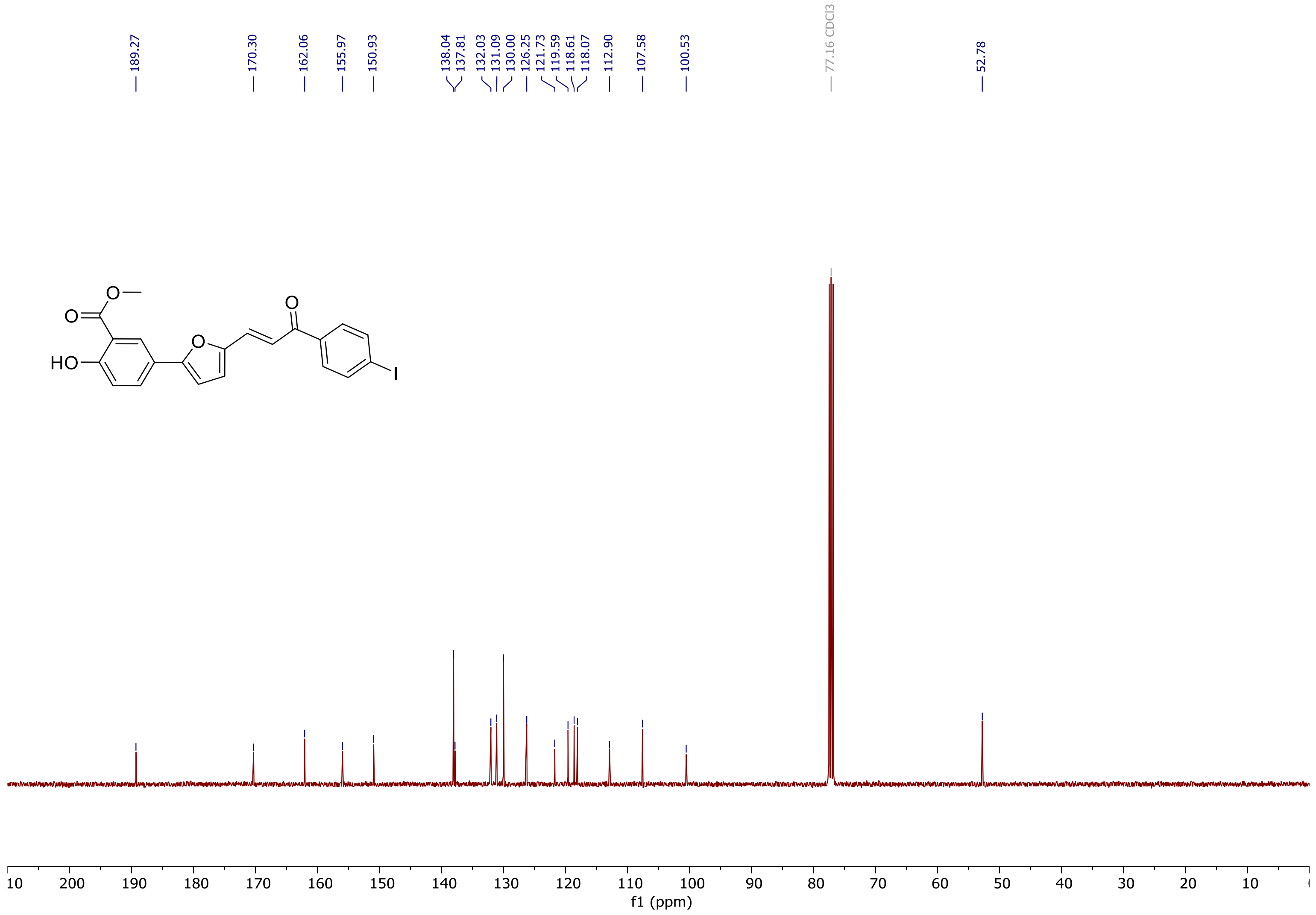

Methyl (*E*)-2-hydroxy-5-{5-{3-[4-(methylthio)phenyl]-3-oxoprop-1-en-1-yl}furan-2-yl}benzoate (34-SMe)

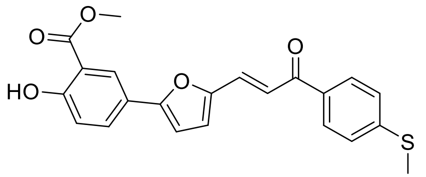

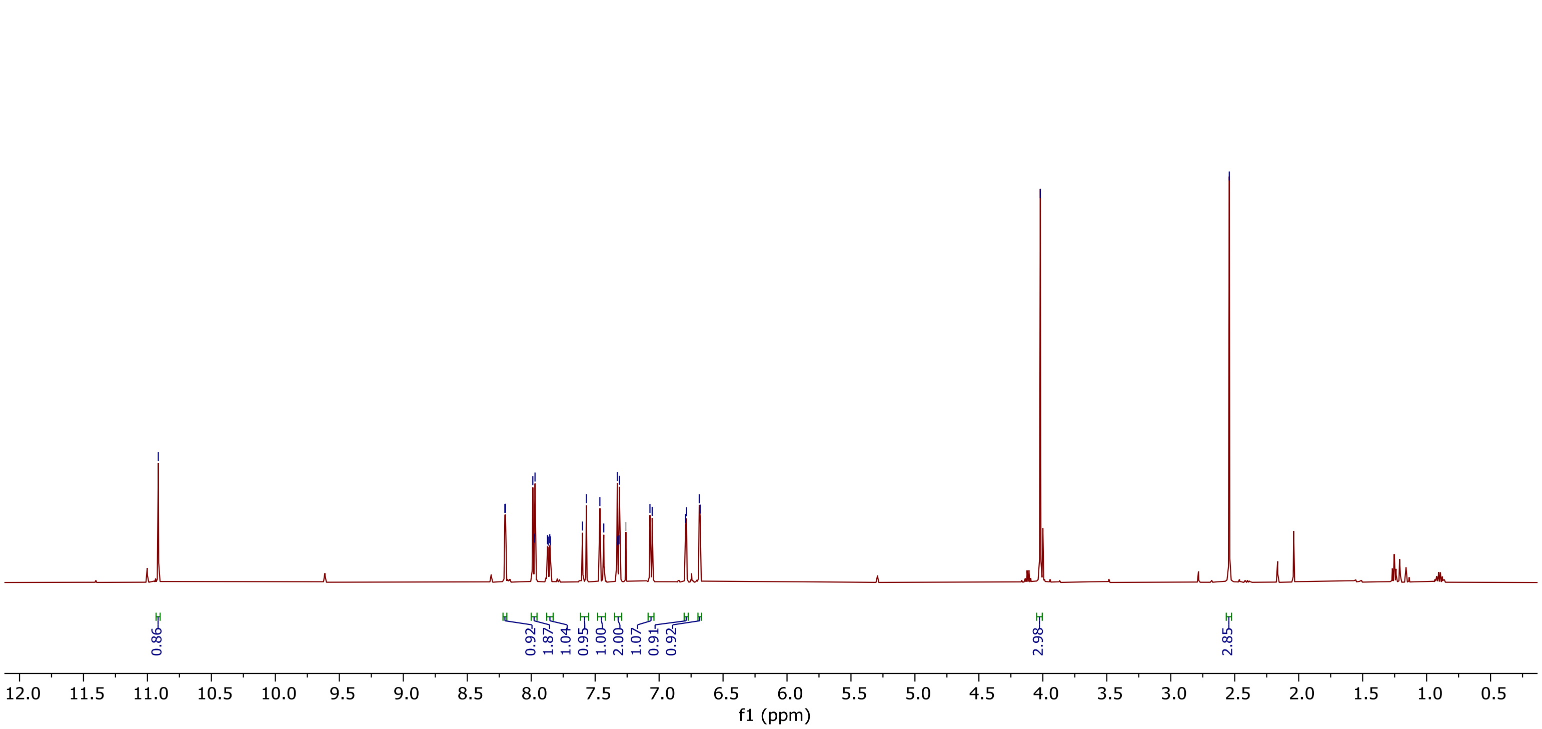

Methyl (*E*)-2-hydroxy-5-{5-{3-[4-(methylthio)phenyl]-3-oxoprop-1-en-1-yl}furan-2-yl}benzoate (34-SMe)

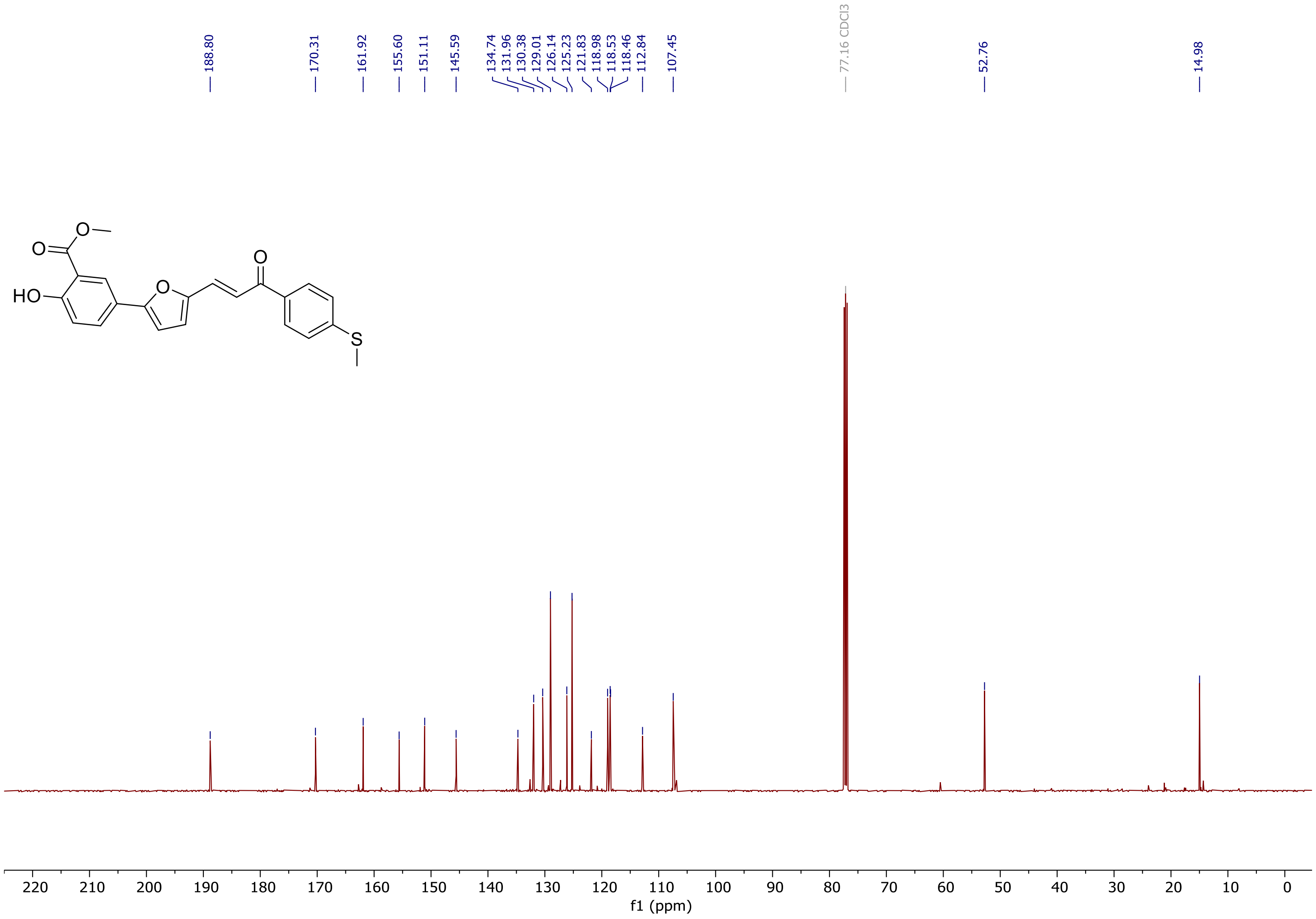

Methyl (*E*)-2-hydroxy-5-{5-[3-oxo-3-(*p*-tolyl)prop-1-en-1-yl]furan-2-yl}benzoate (34-Me)

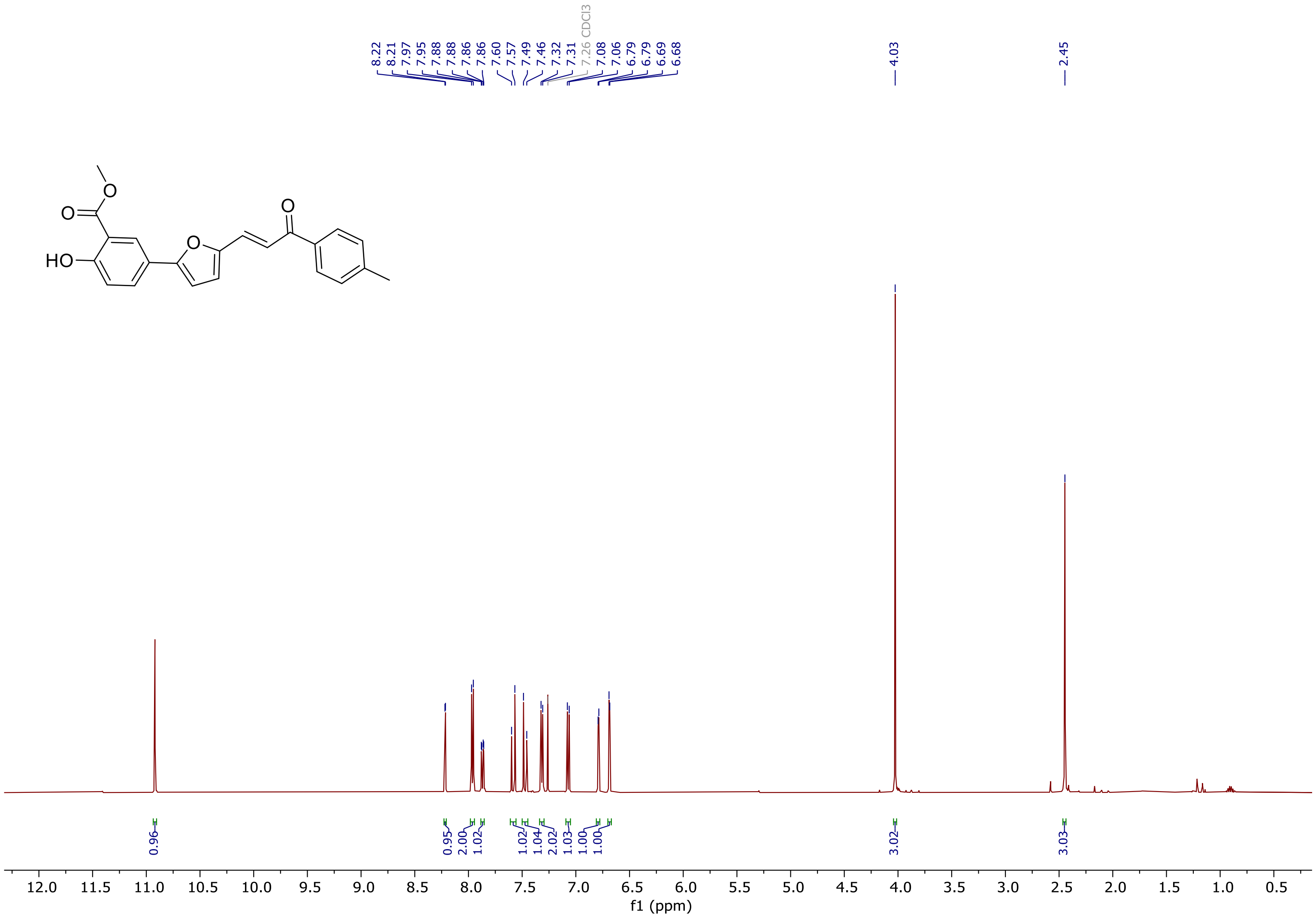

Methyl (*E*)-2-hydroxy-5-{5-[3-oxo-3-(*p*-tolyl)prop-1-en-1-yl]furan-2-yl}benzoate (34-Me)

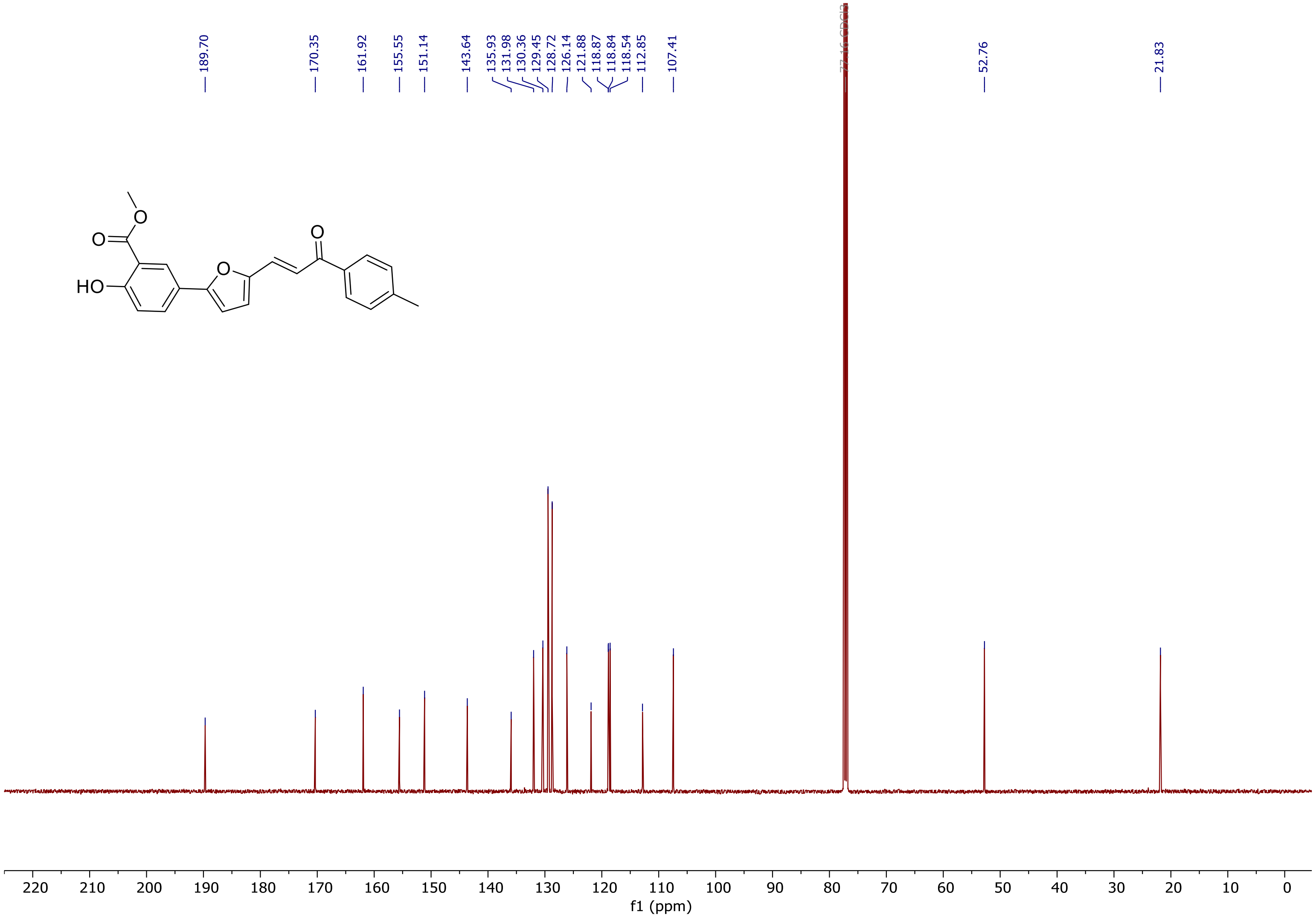

Methyl (*E*)-2-hydroxy-5-{5-{3-oxo-3-[4-(trifluoromethyl)phenyl]prop-1-en-1-yl}furan-2-yl}benzoate (34-CF3)

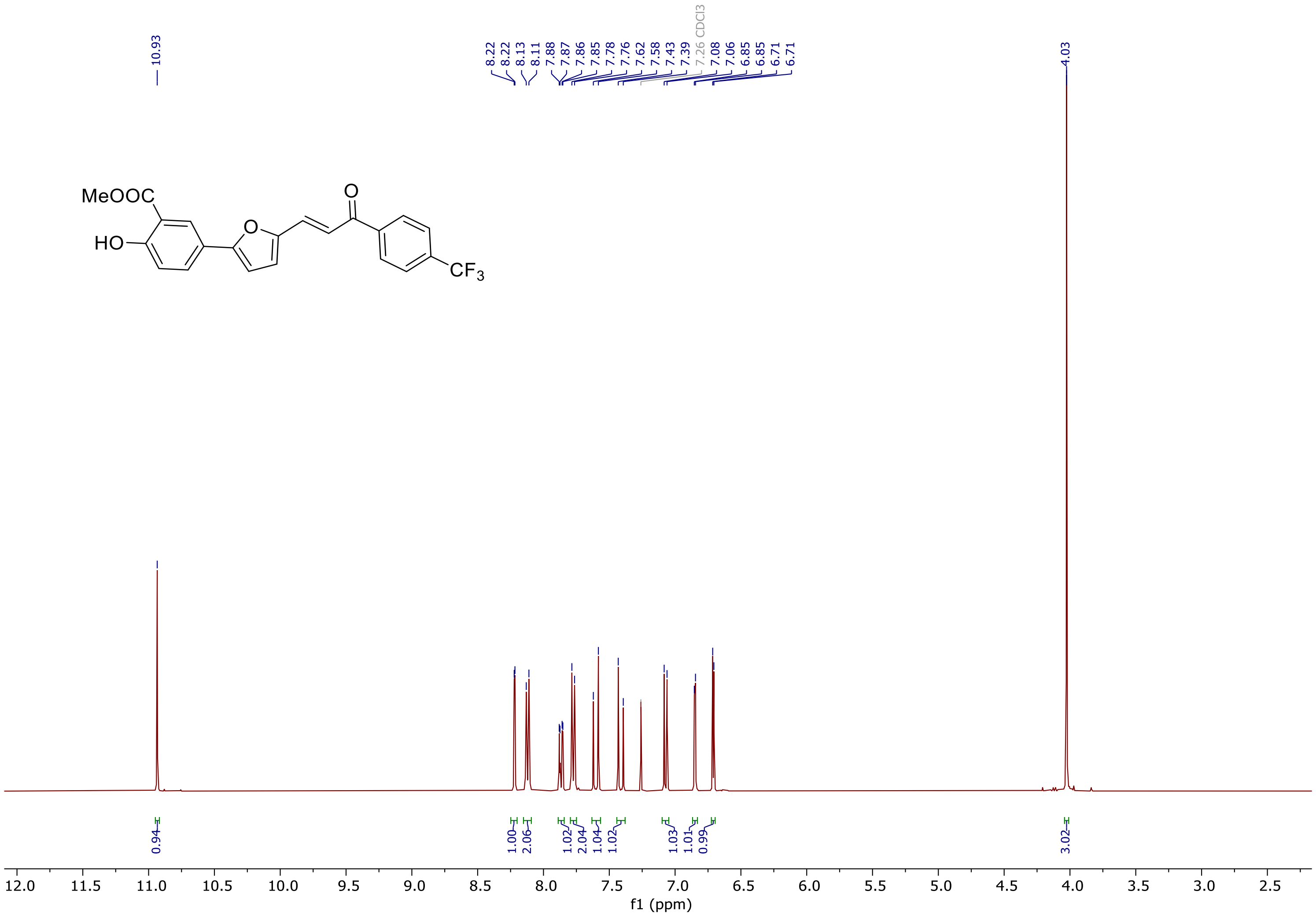

Methyl (*E*)-2-hydroxy-5-{5-{3-oxo-3-[4-(trifluoromethyl)phenyl]prop-1-en-1-yl}furan-2-yl}benzoate (34-CF3)

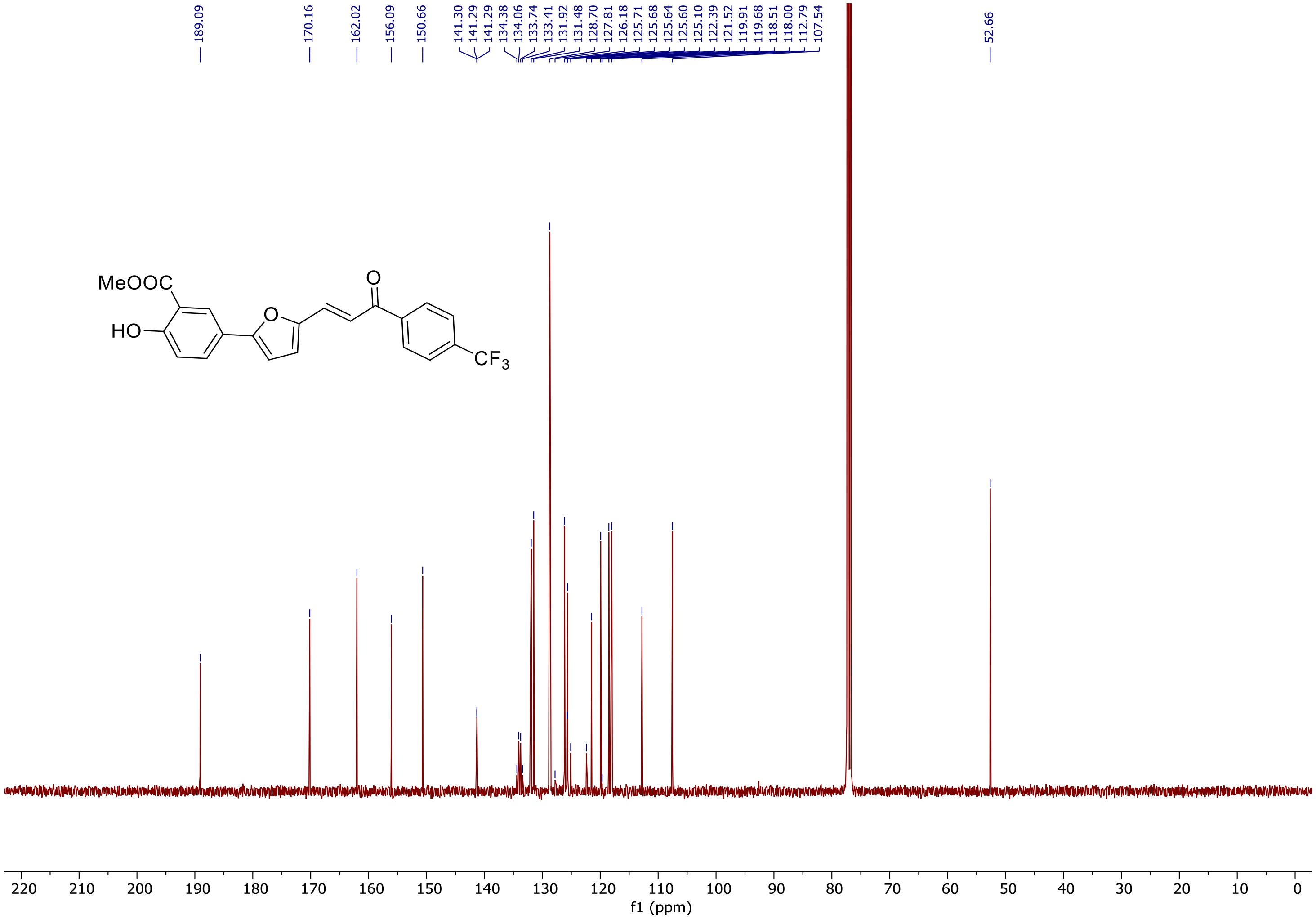

Methyl (*E*)-5-{5-[3-(4-ethylphenyl)-3-oxoprop-1-en-1-yl]furan-2-yl}-2-hydroxybenzoic acid (34-Et)

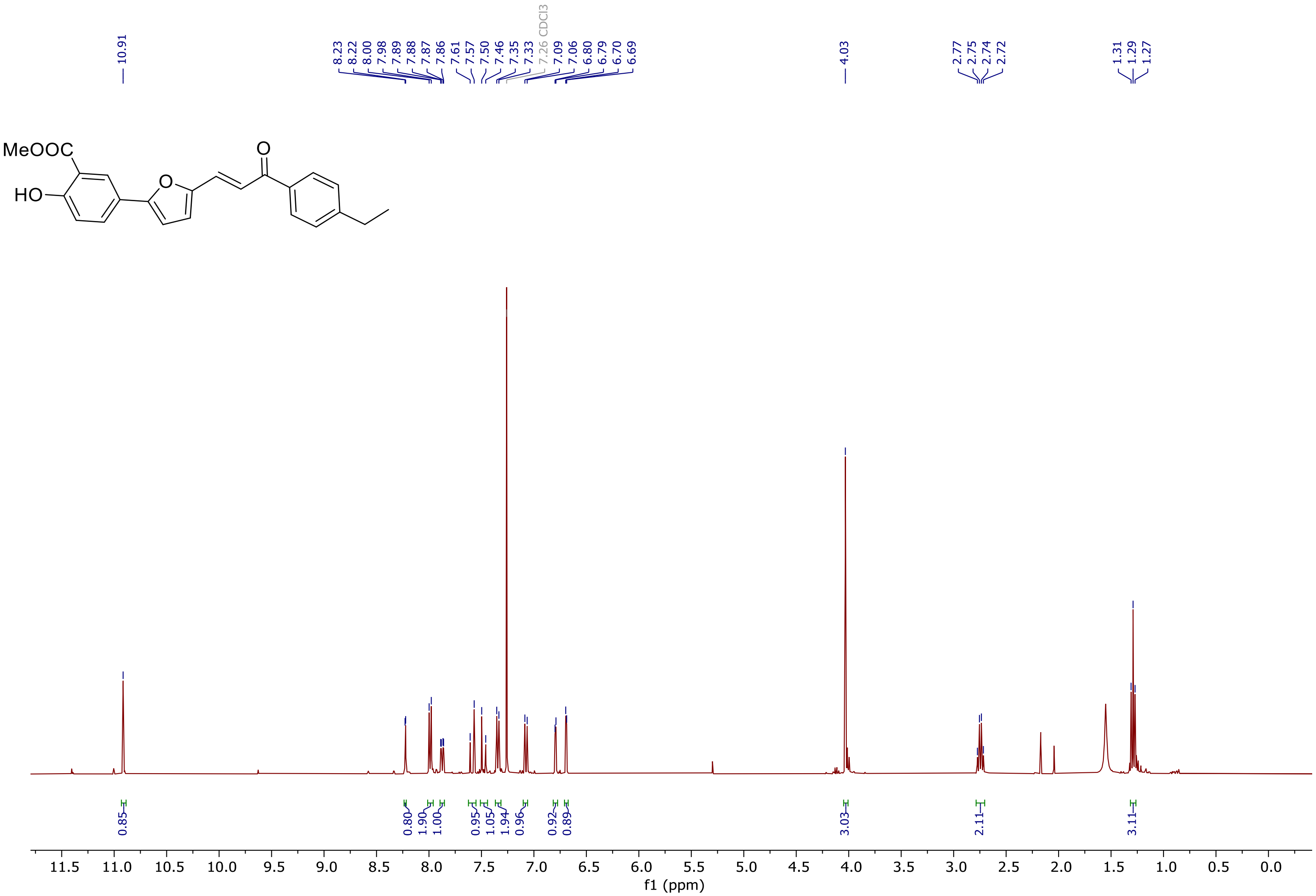

Methyl (*E*)-5-{5-[3-(4-ethylphenyl)-3-oxoprop-1-en-1-yl]furan-2-yl}-2-hydroxybenzoic acid (34-Et)

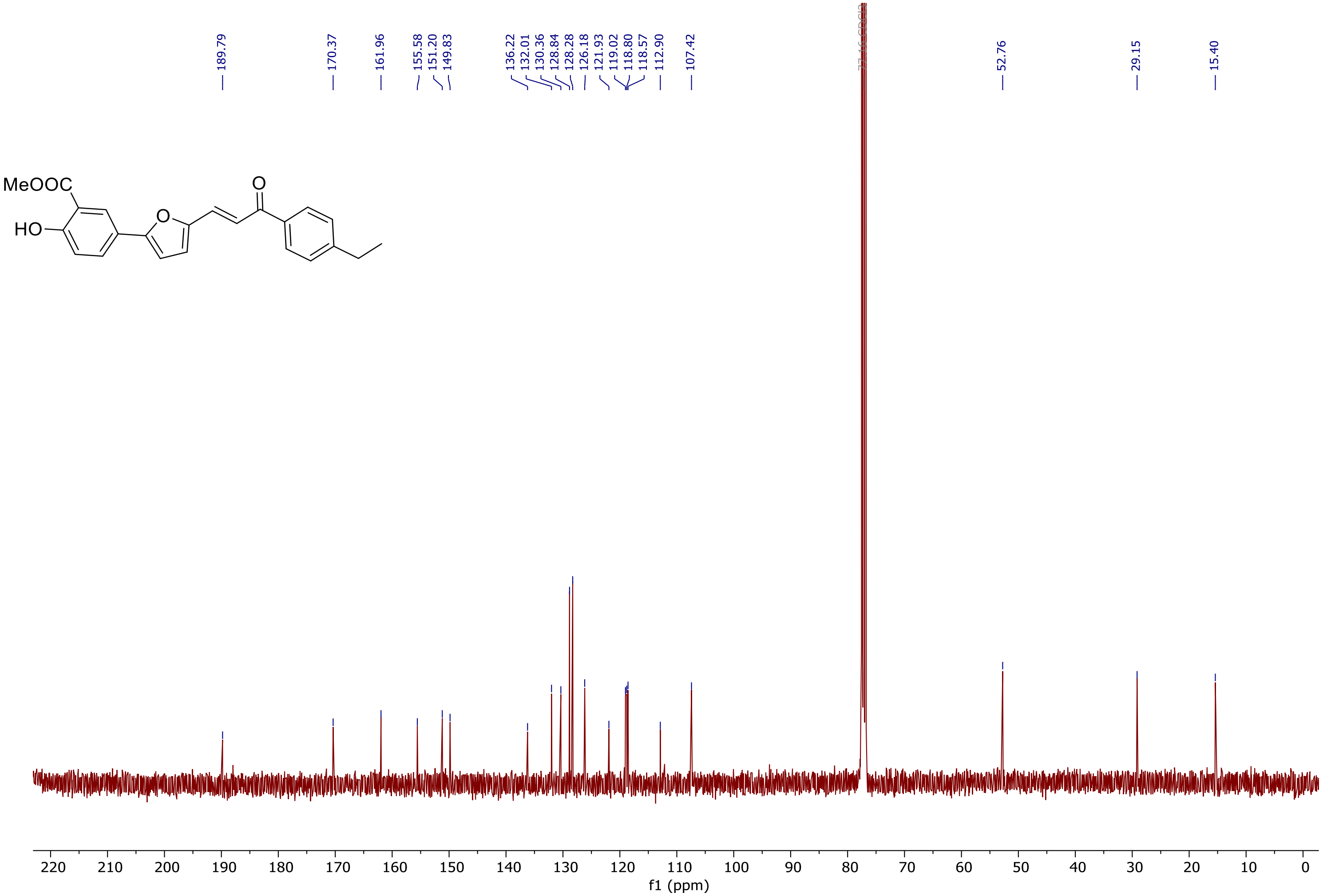

Methyl (*E*)-2-hydroxy-5-{5-[3-(4-isopropylphenyl)-3-oxoprop-1-en-1-yl]furan-2-yl}benzoate (34-iPr)

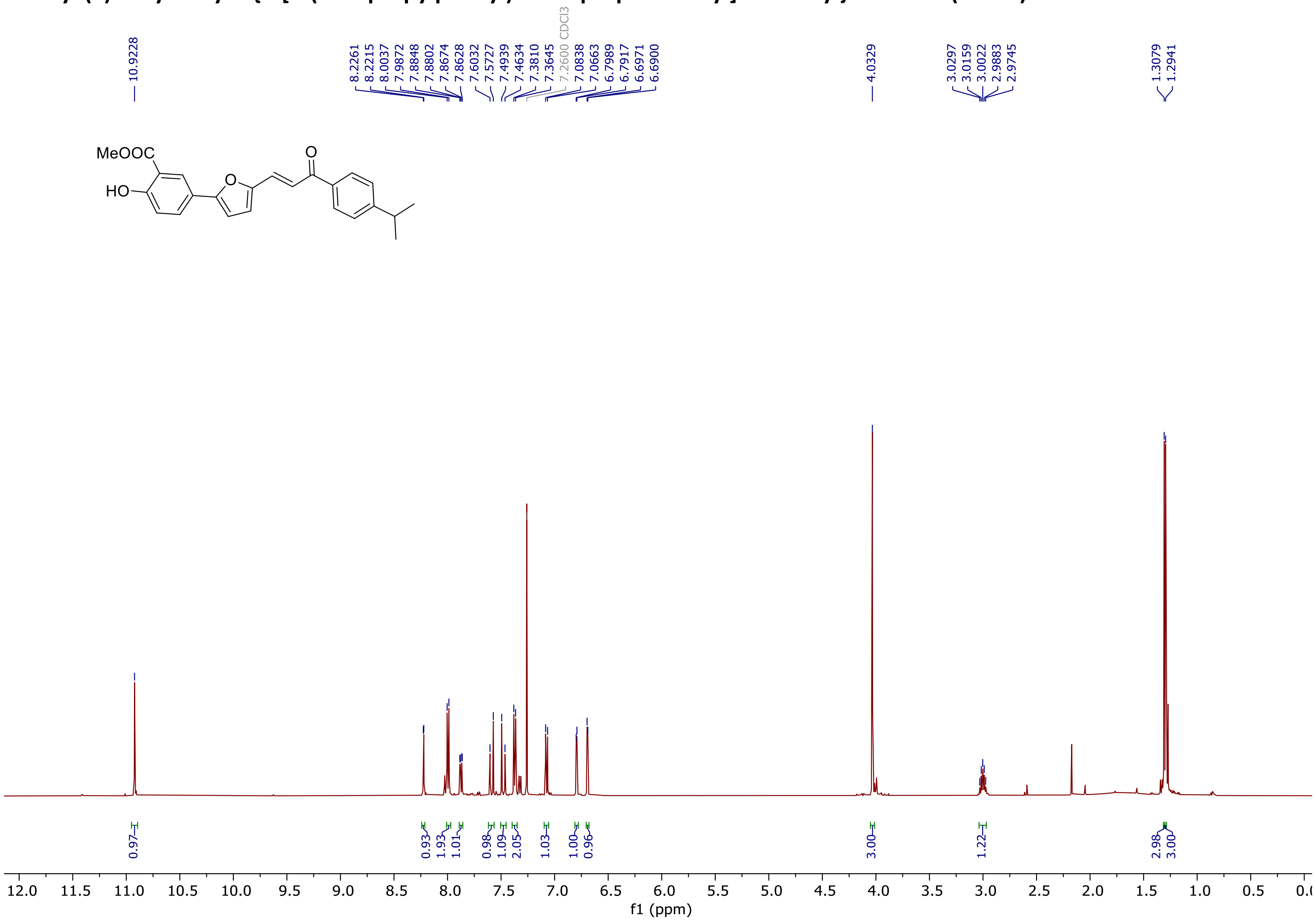

Methyl (*E*)-2-hydroxy-5-{5-[3-(4-isopropylphenyl)-3-oxoprop-1-en-1-yl]furan-2-yl}benzoate (34-iPr)

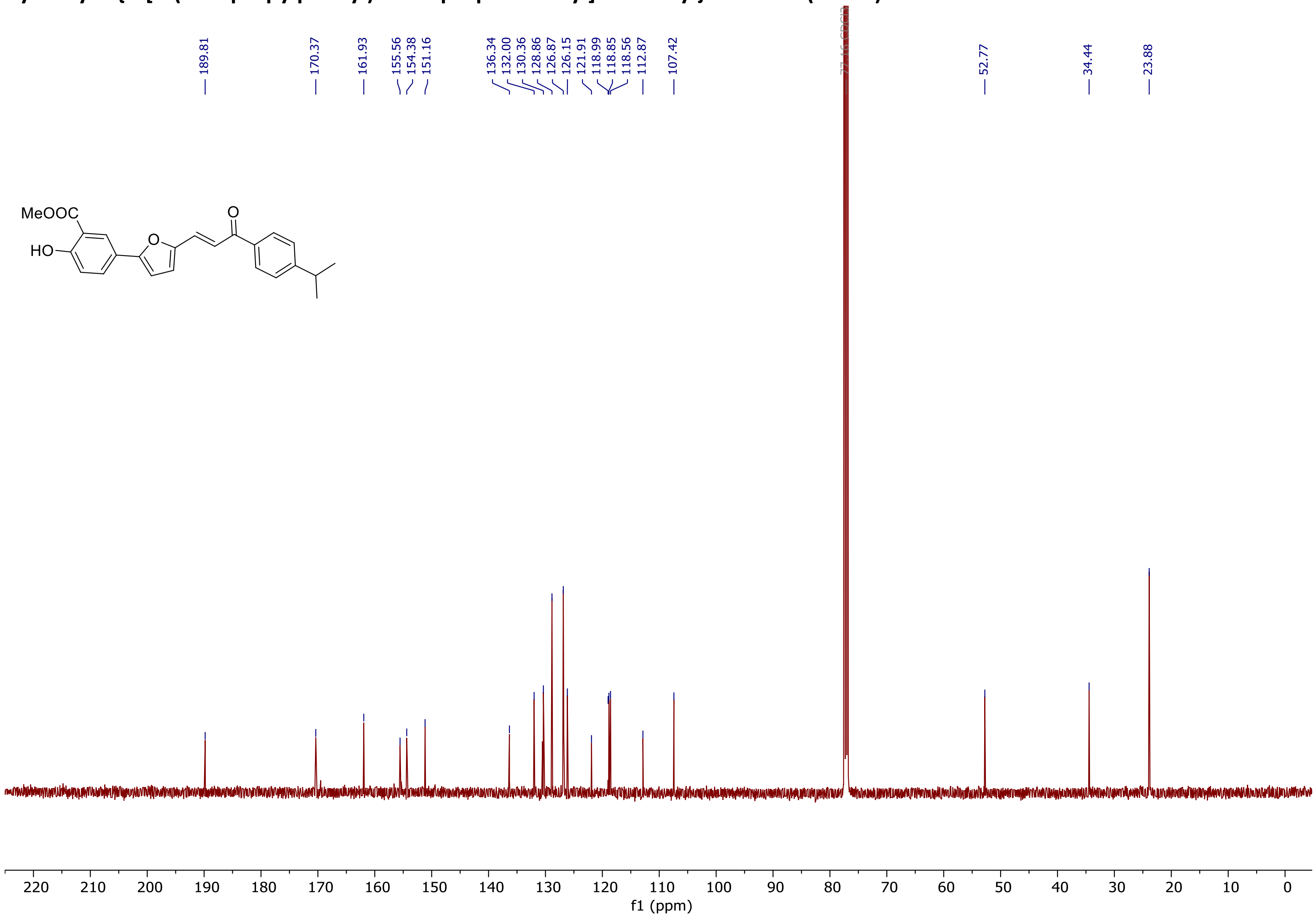

Methyl (*E*)-5-{5-[3-(4-butylphenyl)-3-oxoprop-1-en-1-yl]furan-2-yl}-2-hydroxybenzoate (**34-Bu**).

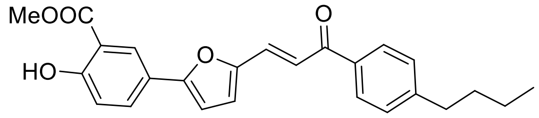

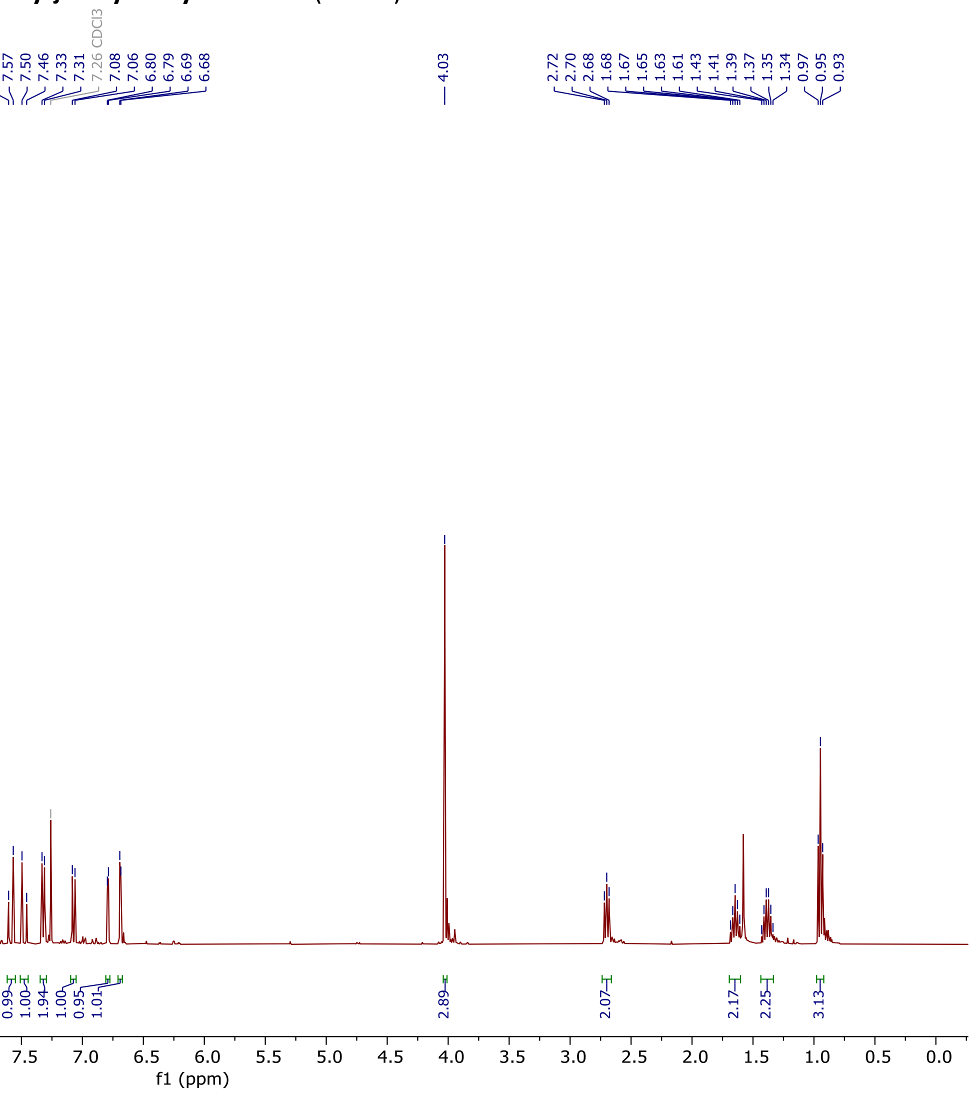

Methyl (*E*)-5-{5-[3-(4-butylphenyl)-3-oxoprop-1-en-1-yl]furan-2-yl}-2-hydroxybenzoate (34-Bu).

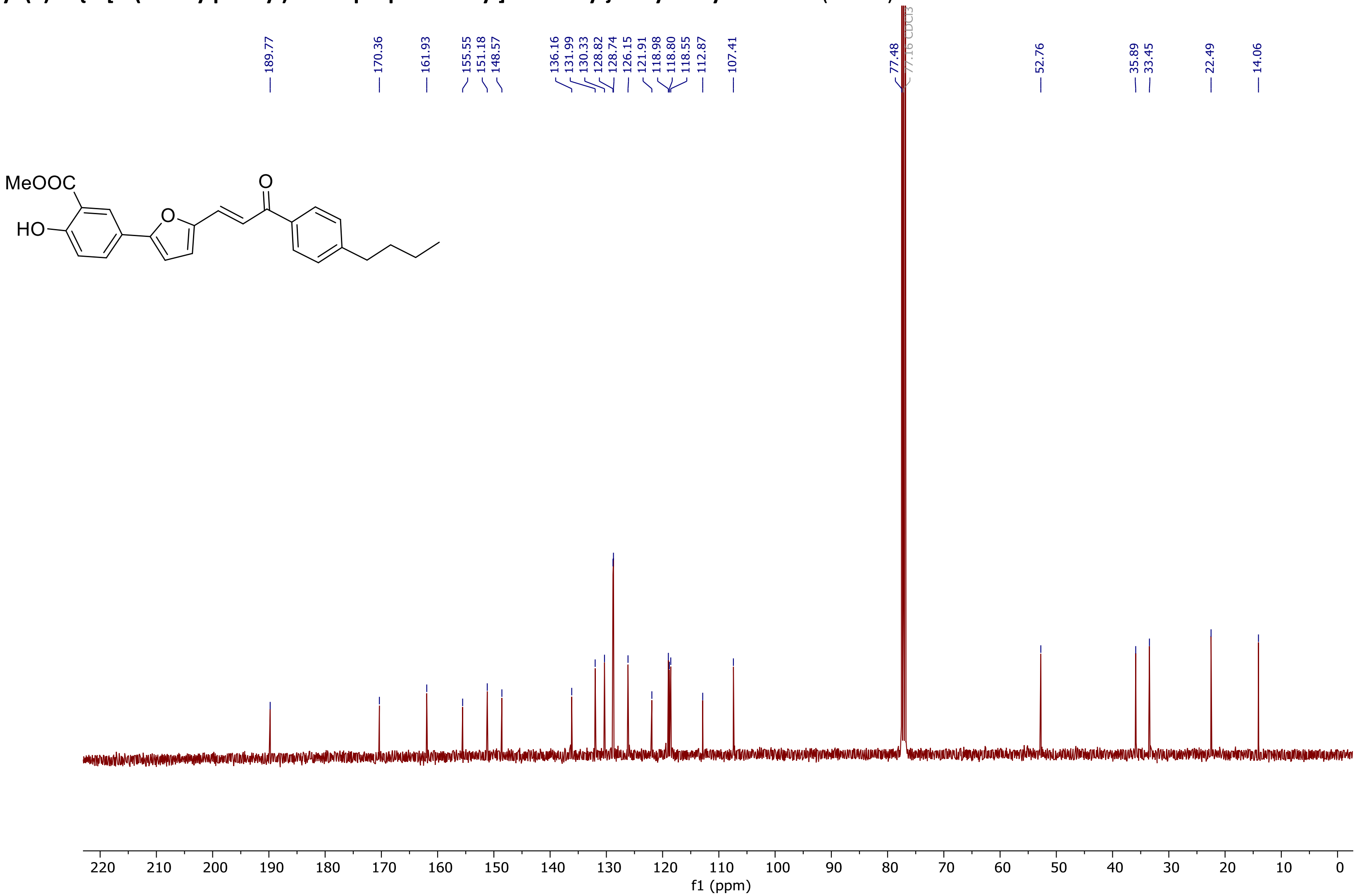

Methyl (*E*)-5-{5-[3-(4-hexylphenyl)-3-oxoprop-1-en-1-yl]furan-2-yl}-2-hydroxybenzoate (34-Hex).

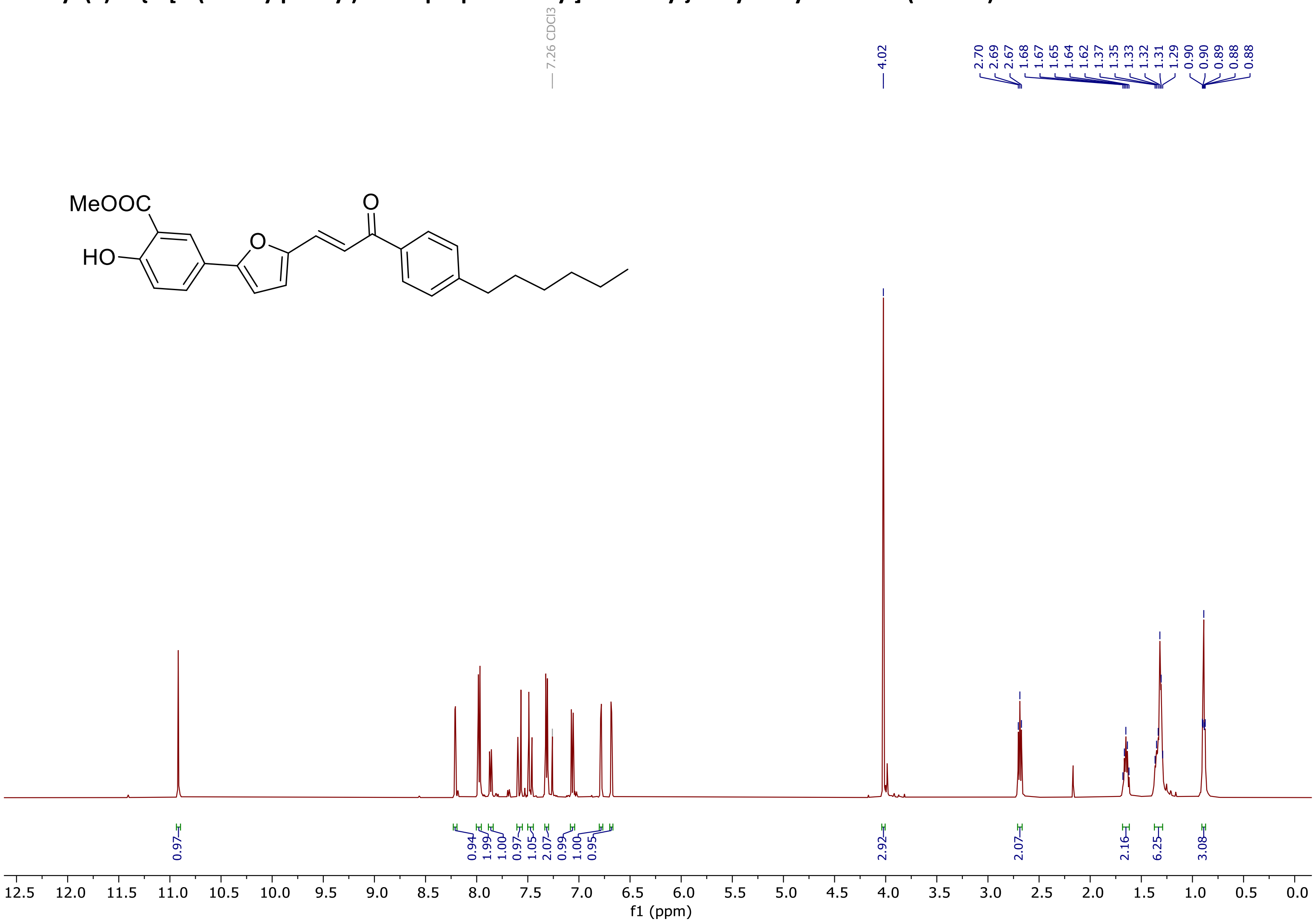

Methyl (*E*)-5-{5-[3-(4-hexylphenyl)-3-oxoprop-1-en-1-yl]furan-2-yl}-2-hydroxybenzoate (34-Hex).

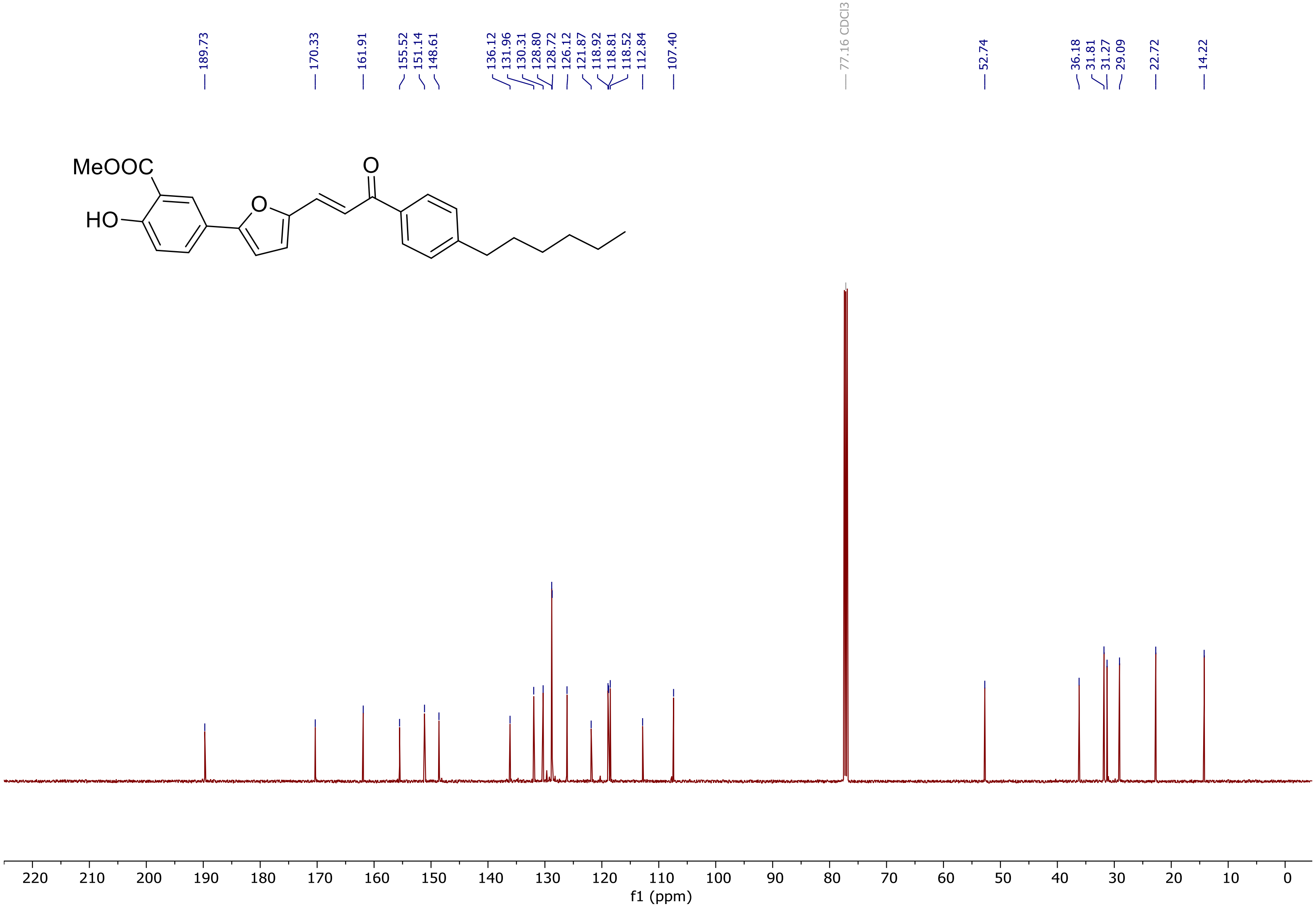

Methyl 2-hydroxy-5-{5-{3-oxo-3-[4-(trifluoromethyl)phenyl]propyl}furan-2-yl}benzoate (37)

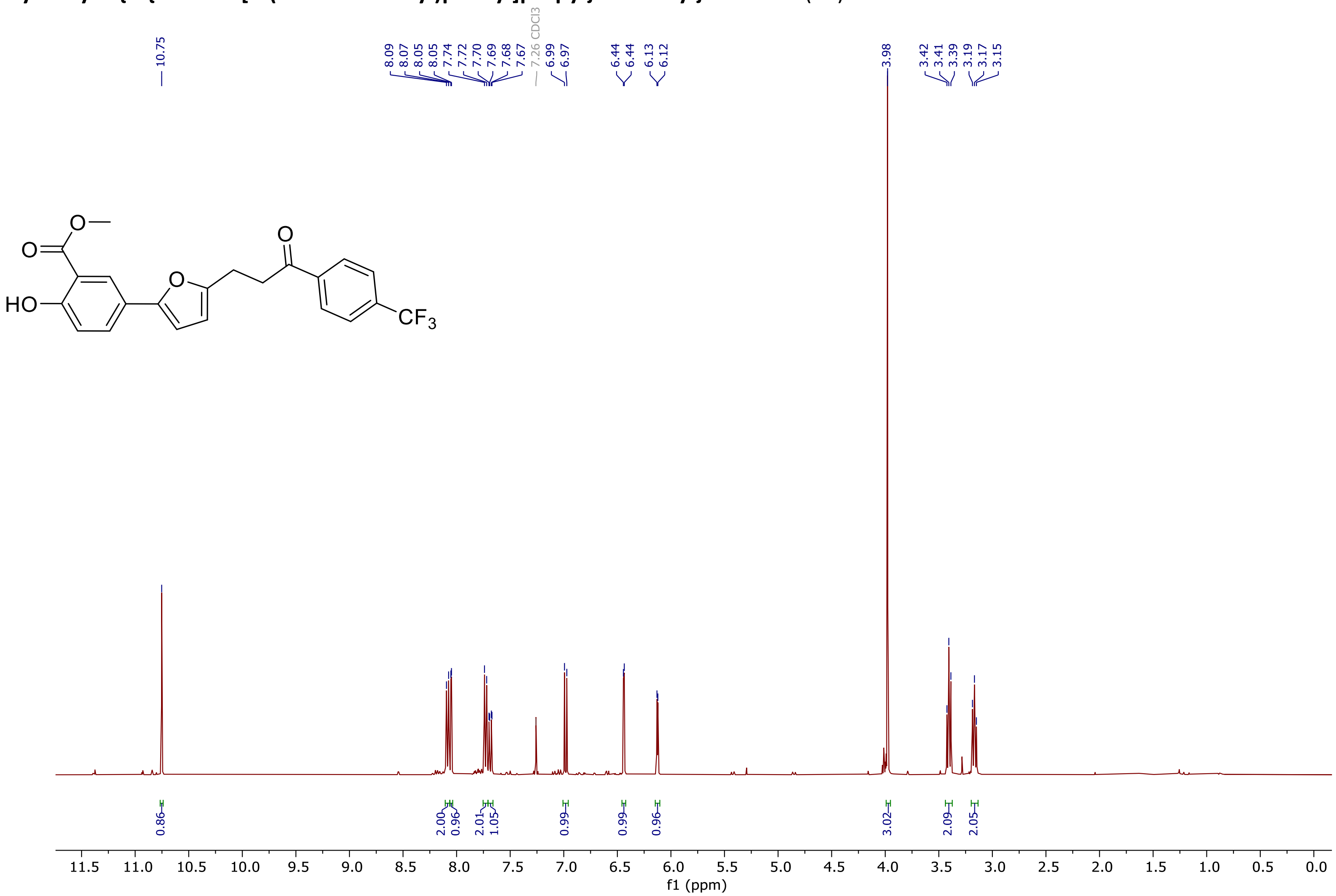

Methyl 2-hydroxy-5-{5-{3-oxo-3-[4-(trifluoromethyl)phenyl]propyl}furan-2-yl}benzoate (37)

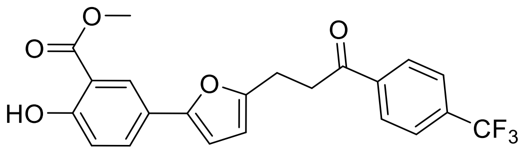

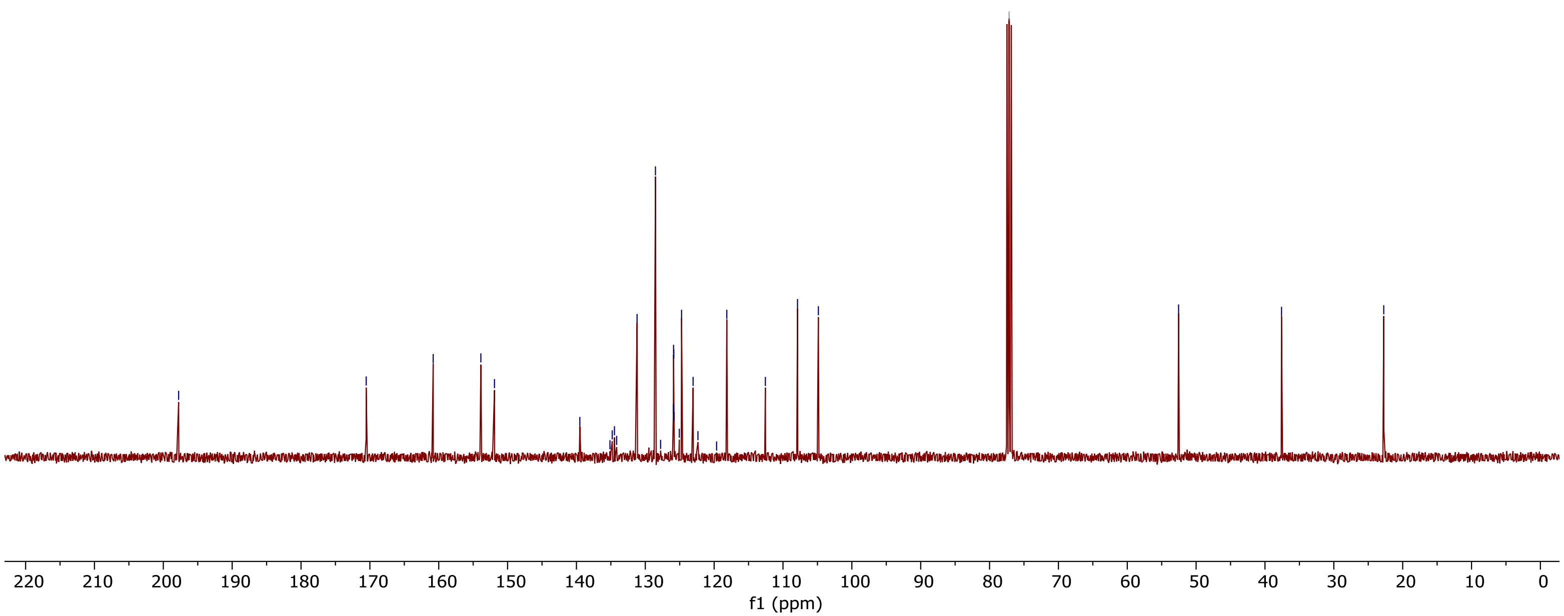

Methyl 5-{5-[3-(4-butylphenyl)-3-oxopropyl]furan-2-yl}-2-hydroxybenzoate (38)

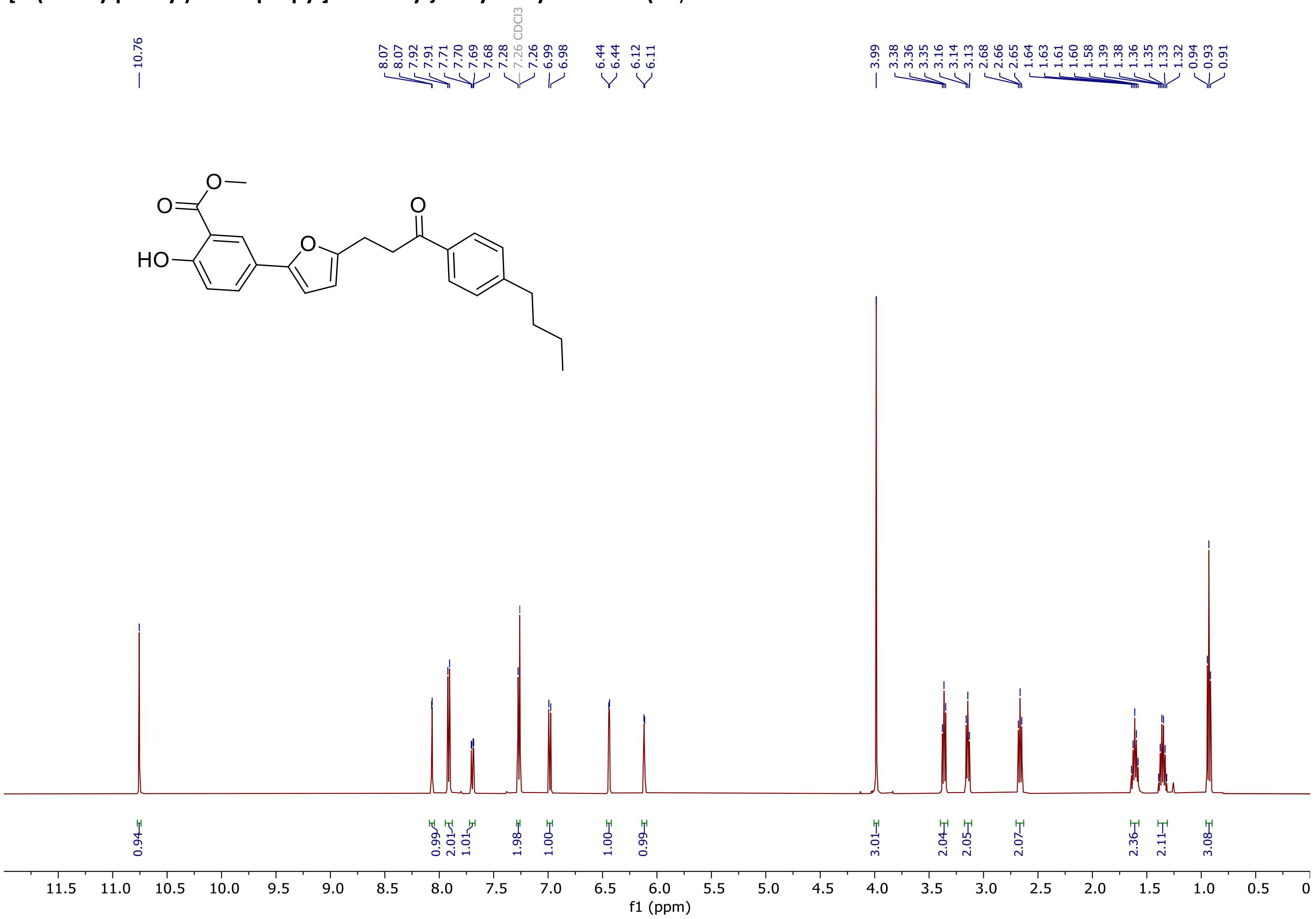

Methyl 5-{5-[3-(4-butylphenyl)-3-oxopropyl]furan-2-yl}-2-hydroxybenzoate (38)

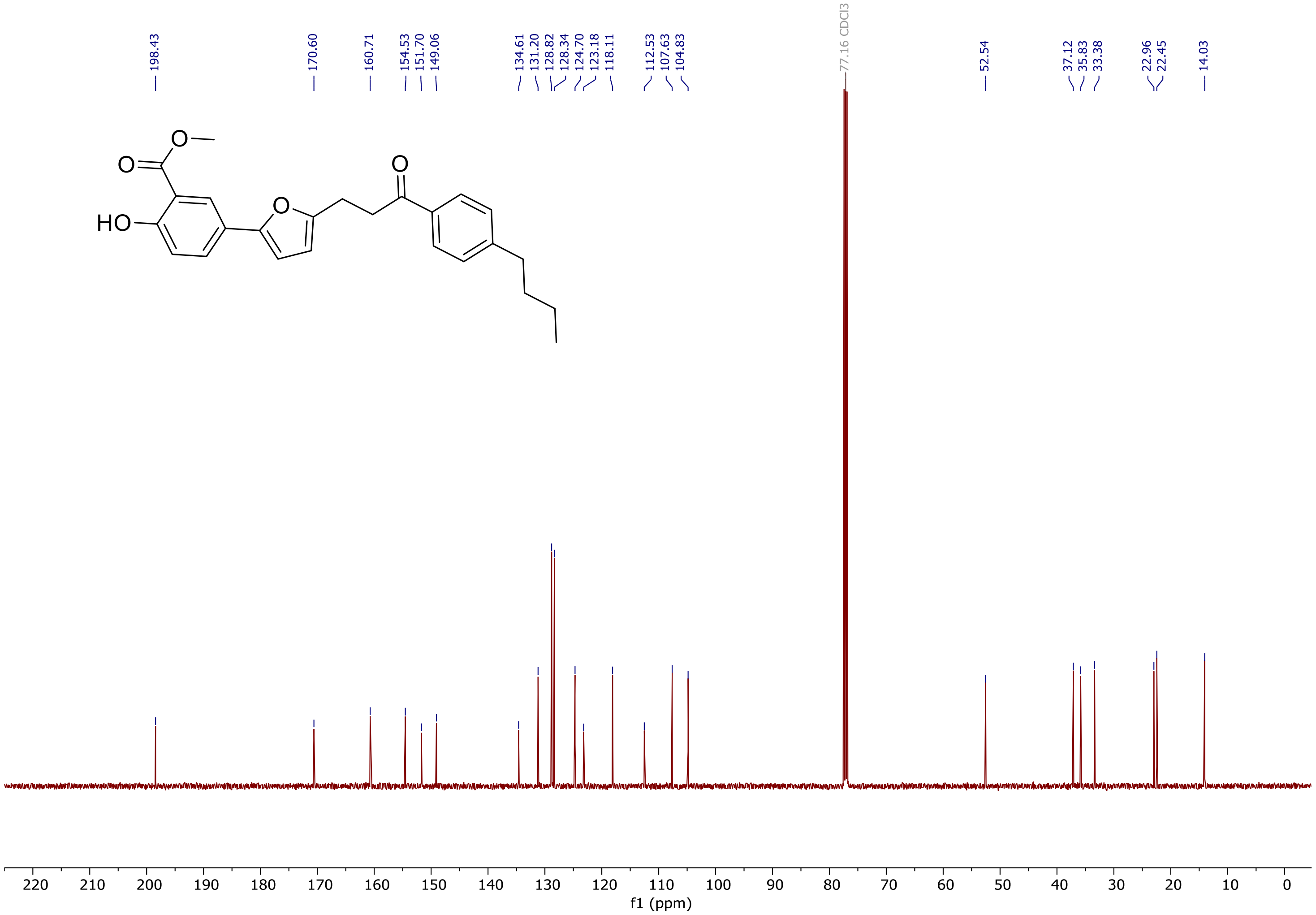

Methyl 5-{5-[3-(4-butylphenyl)-3-hydroxypropyl]furan-2-yl}-2-hydroxybenzoate (39)

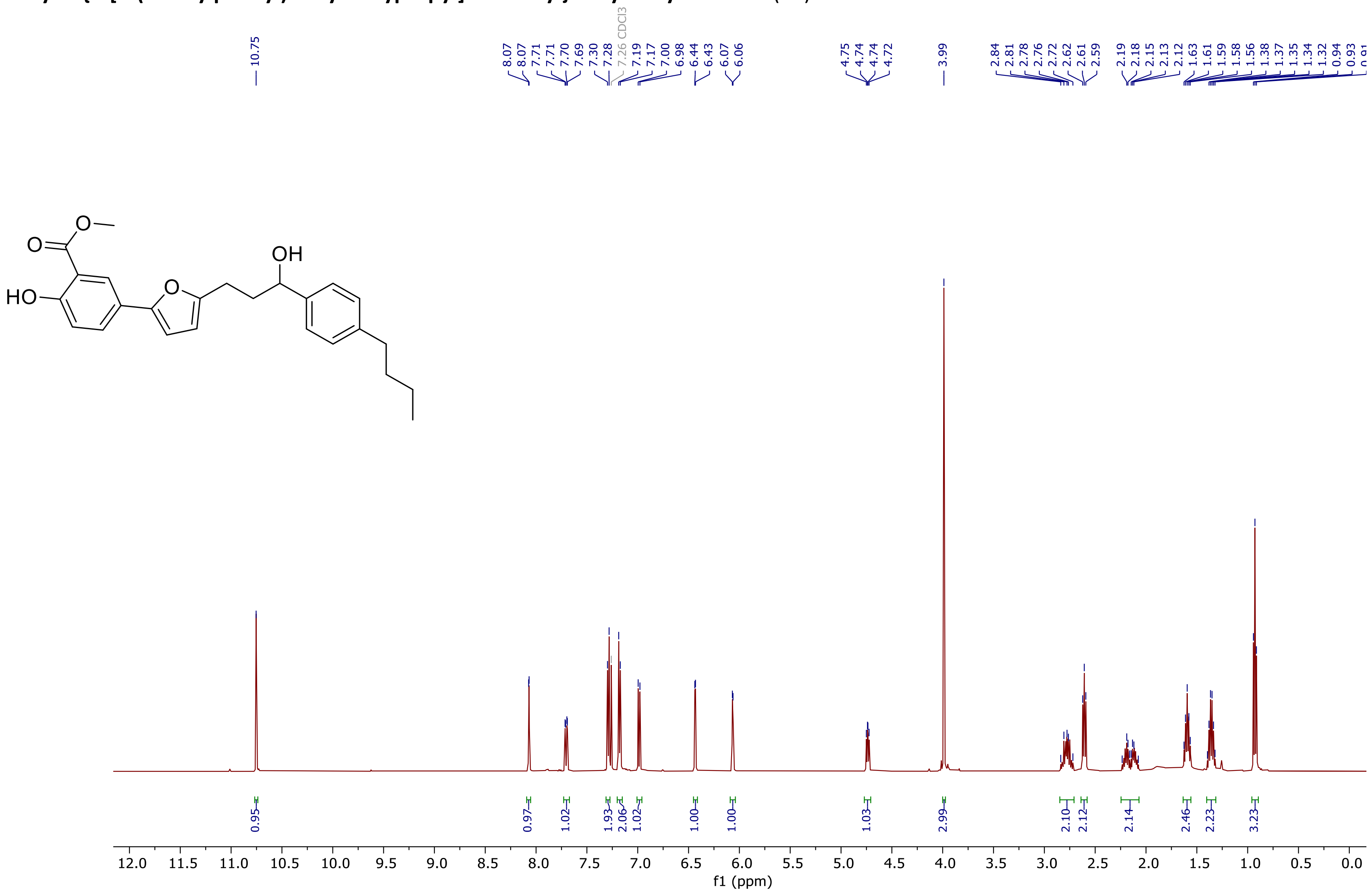

Methyl 5-{5-[3-(4-butylphenyl)-3-hydroxypropyl]furan-2-yl}-2-hydroxybenzoate (39)

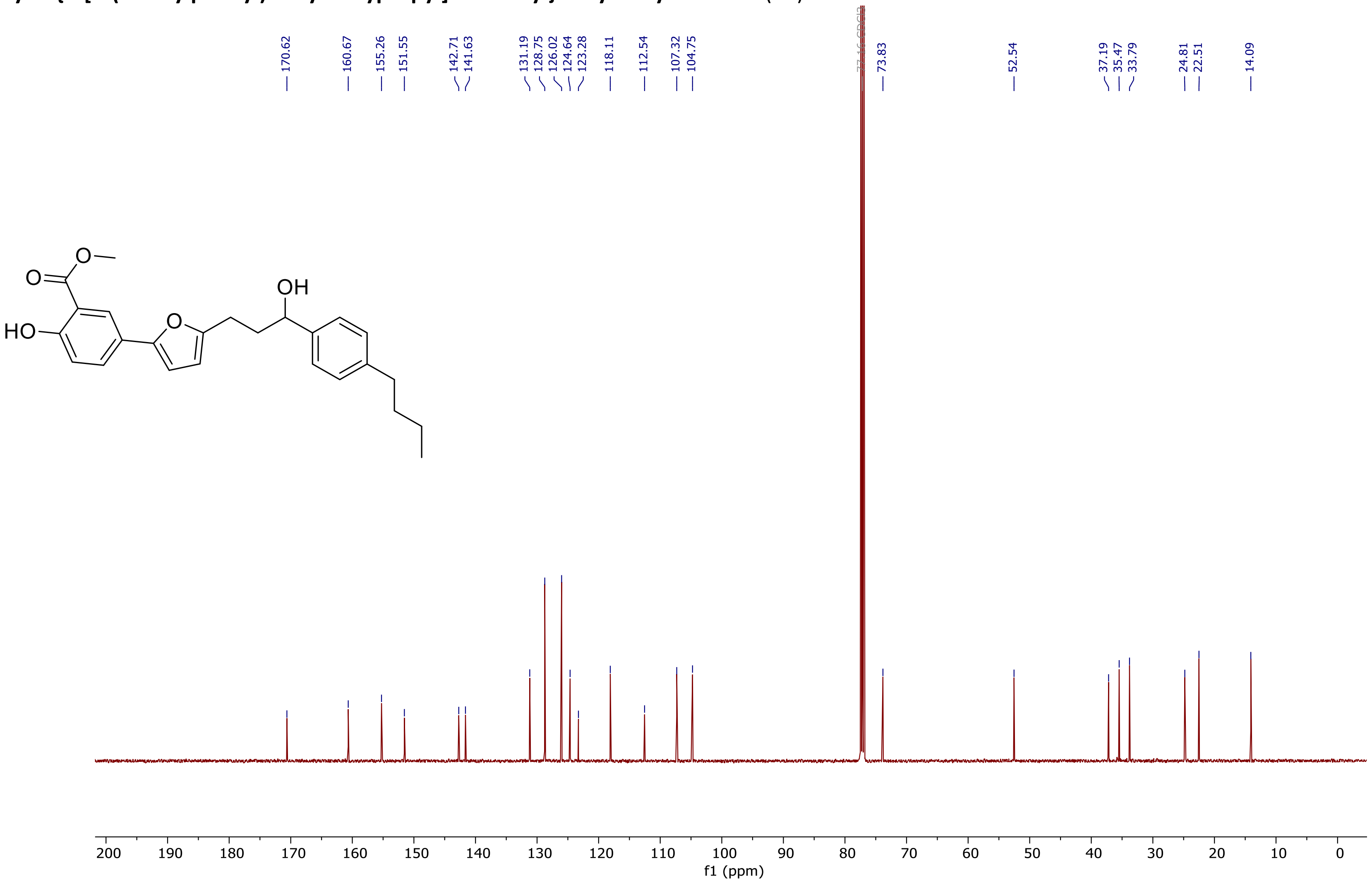

Methyl (*E*)-2-hydroxy-5-[5-(4,4,4-trifluoro-3-oxobut-1-en-1-yl)furan-2-yl]benzoate (40).

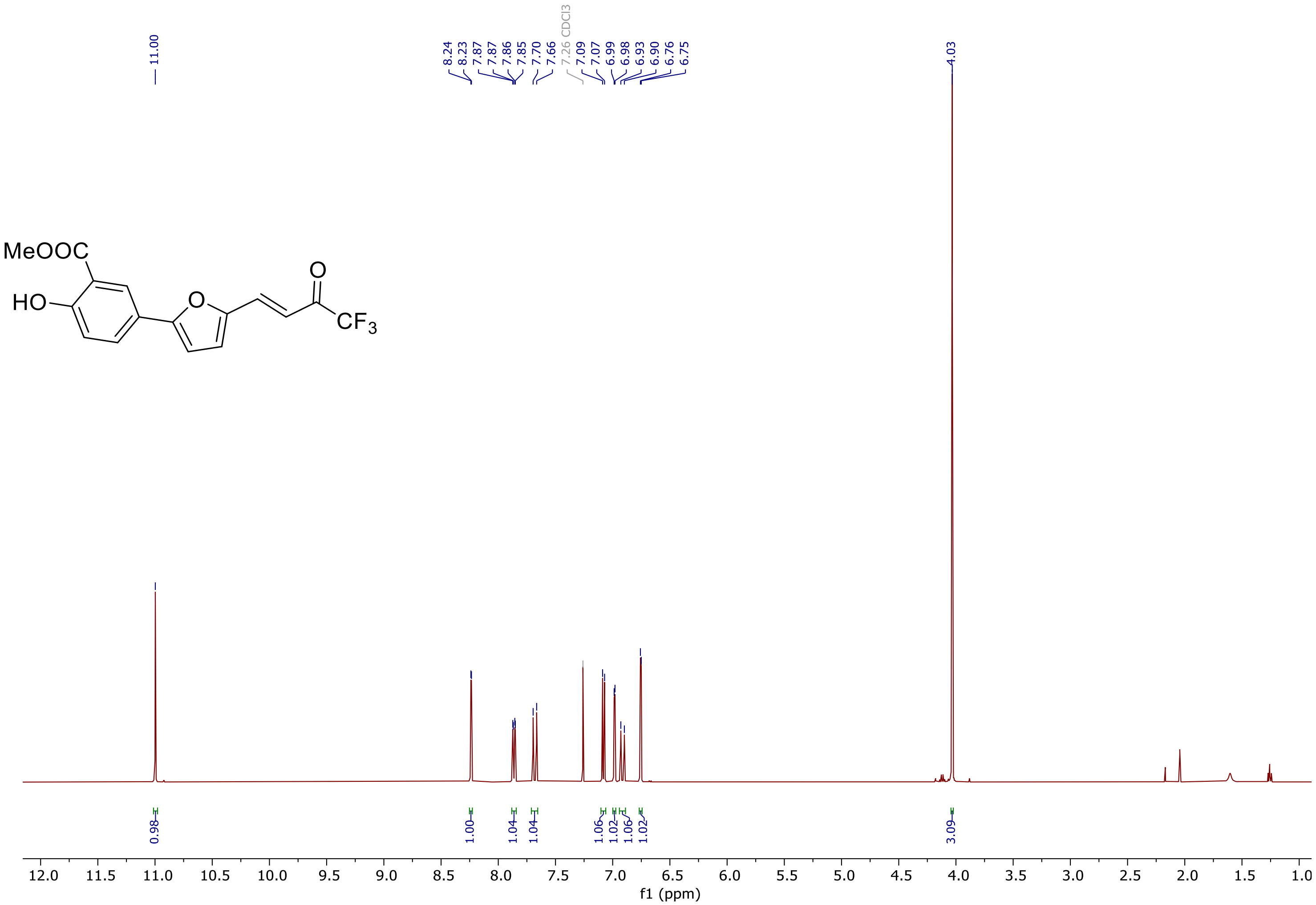

Methyl (*E*)-2-hydroxy-5-[5-(4,4,4-trifluoro-3-oxobut-1-en-1-yl)furan-2-yl]benzoate (40)

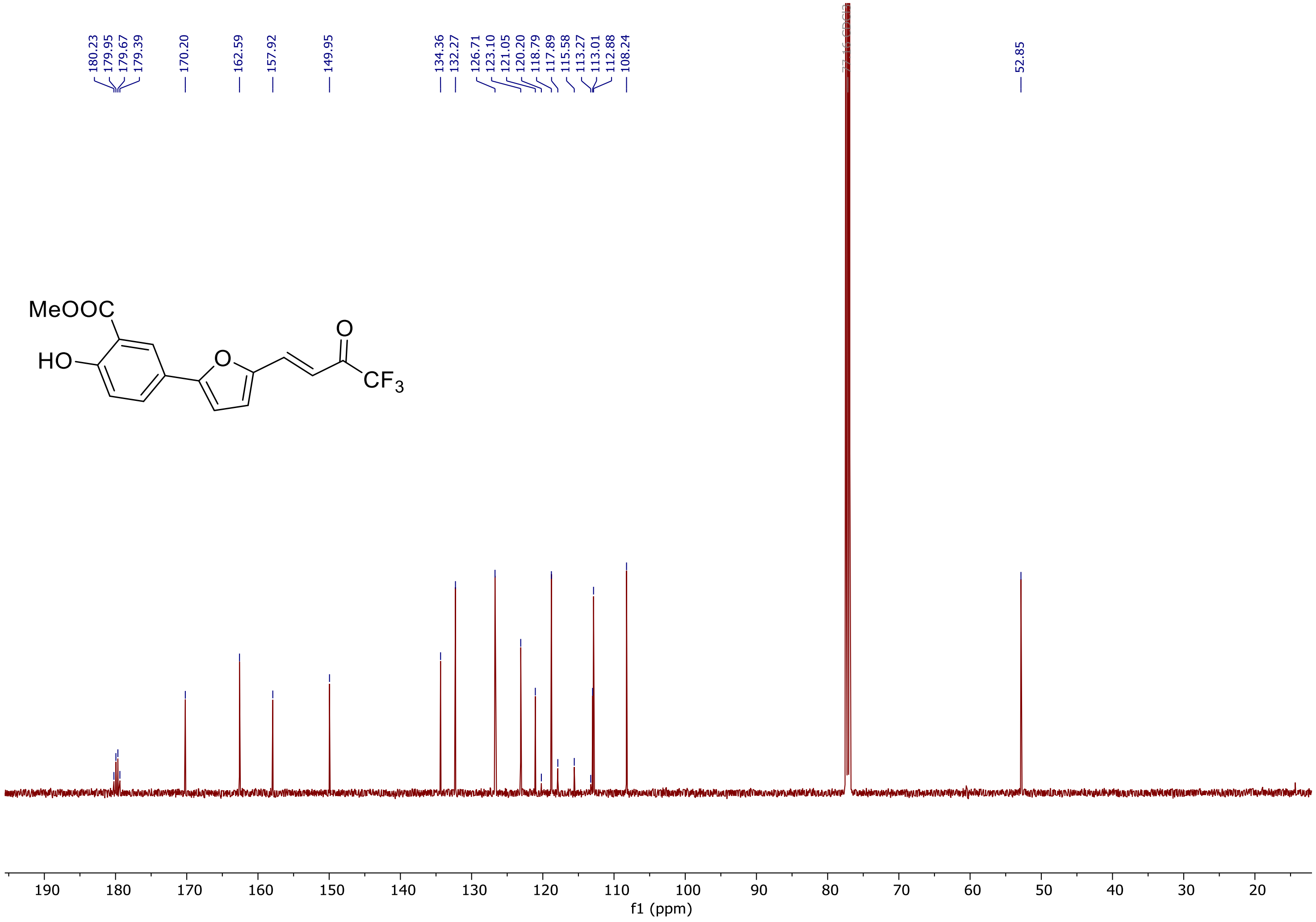

Methyl 5-[5-(2-acetyl-3-oxobut-1-en-1-yl)furan-2-yl]-2-hydroxy-benzoate (41).

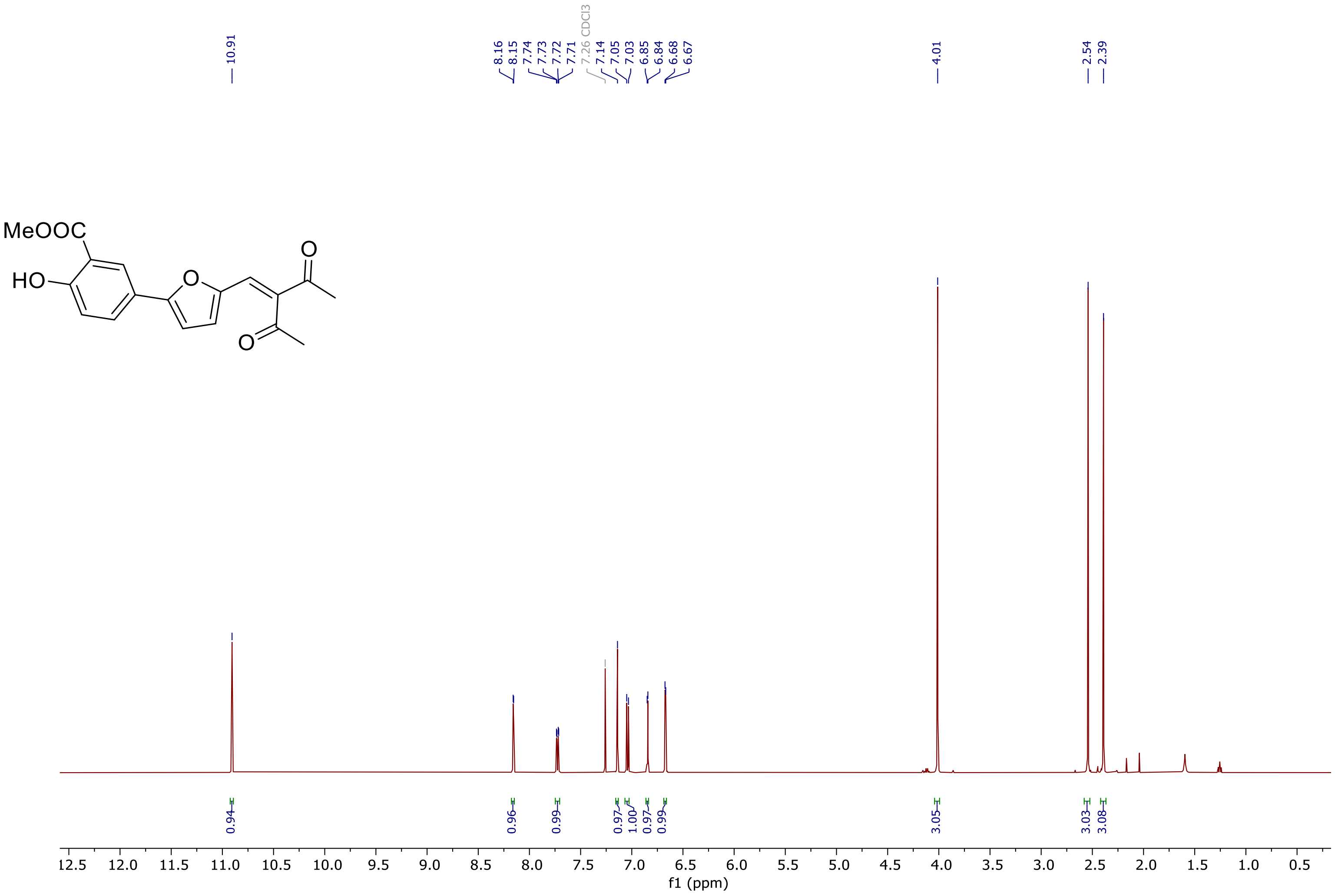

Methyl 5-[5-(2-acetyl-3-oxobut-1-en-1-yl)furan-2-yl]-2-hydroxy-benzoate (41).

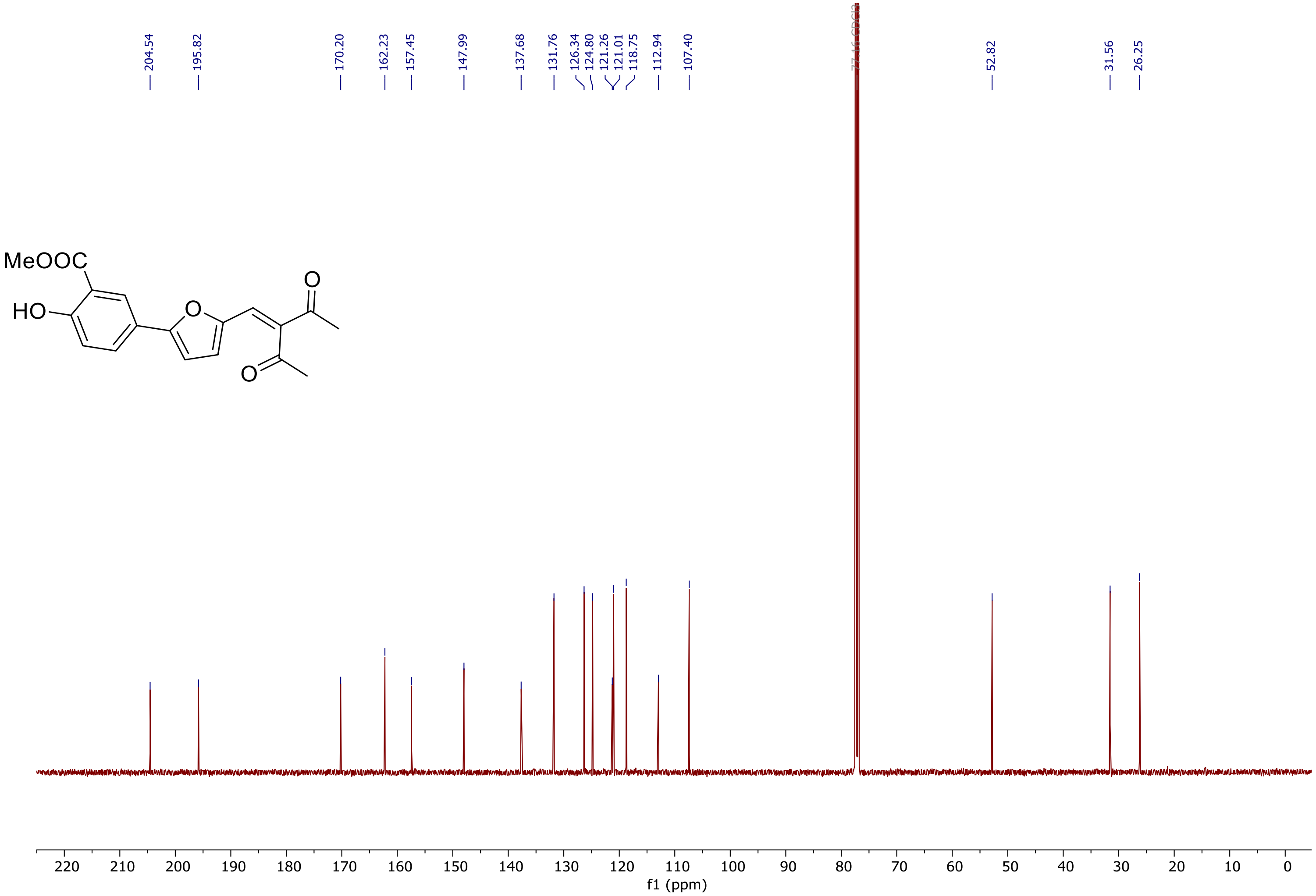

Methyl (*E*)-2-Hydroxy-5-{5-[3-(furan-2-yl)-3-oxoprop-1-en-1-yl]furan-2-yl}benzoic acid (42)

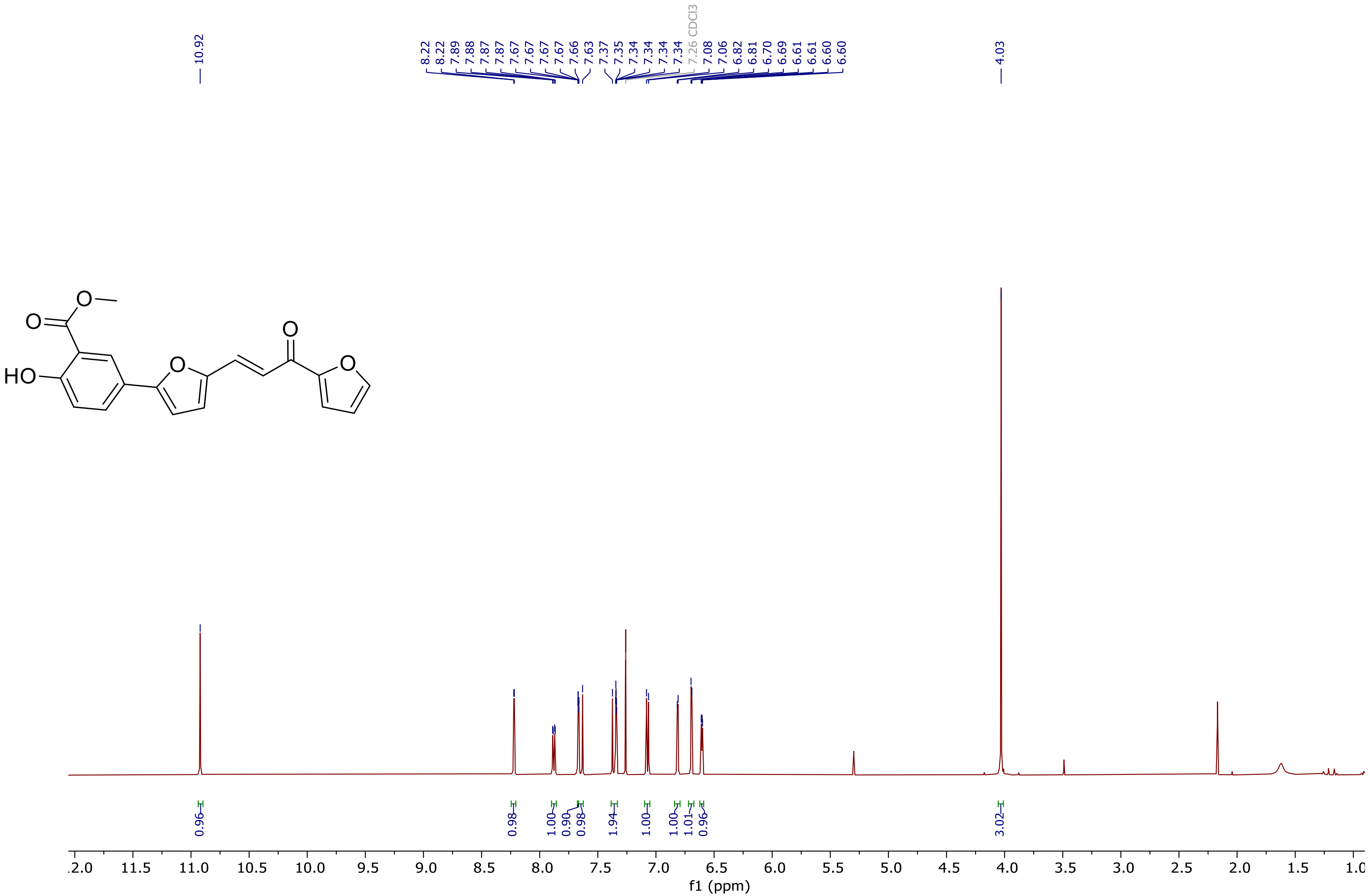

Methyl (*E*)-2-Hydroxy-5-{5-[3-(furan-2-yl)-3-oxoprop-1-en-1-yl]furan-2-yl}benzoic acid (42)

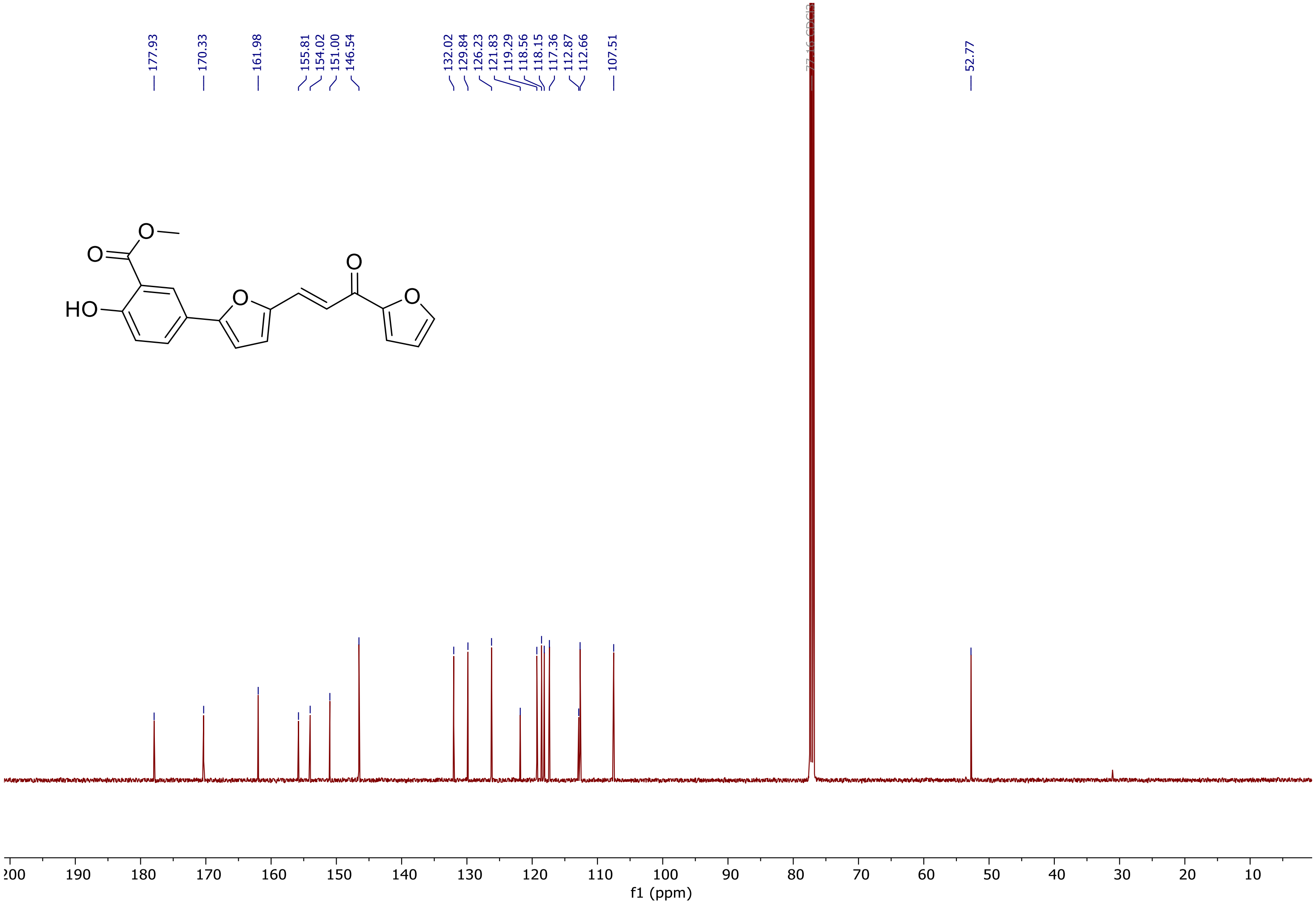

Methyl 2-hydroxy-5-{5-[3-hydroxy-3-(*p*-trifluoromethylphenyl)propyl]tetrahydrofuran-2-yl}benzoate (43)

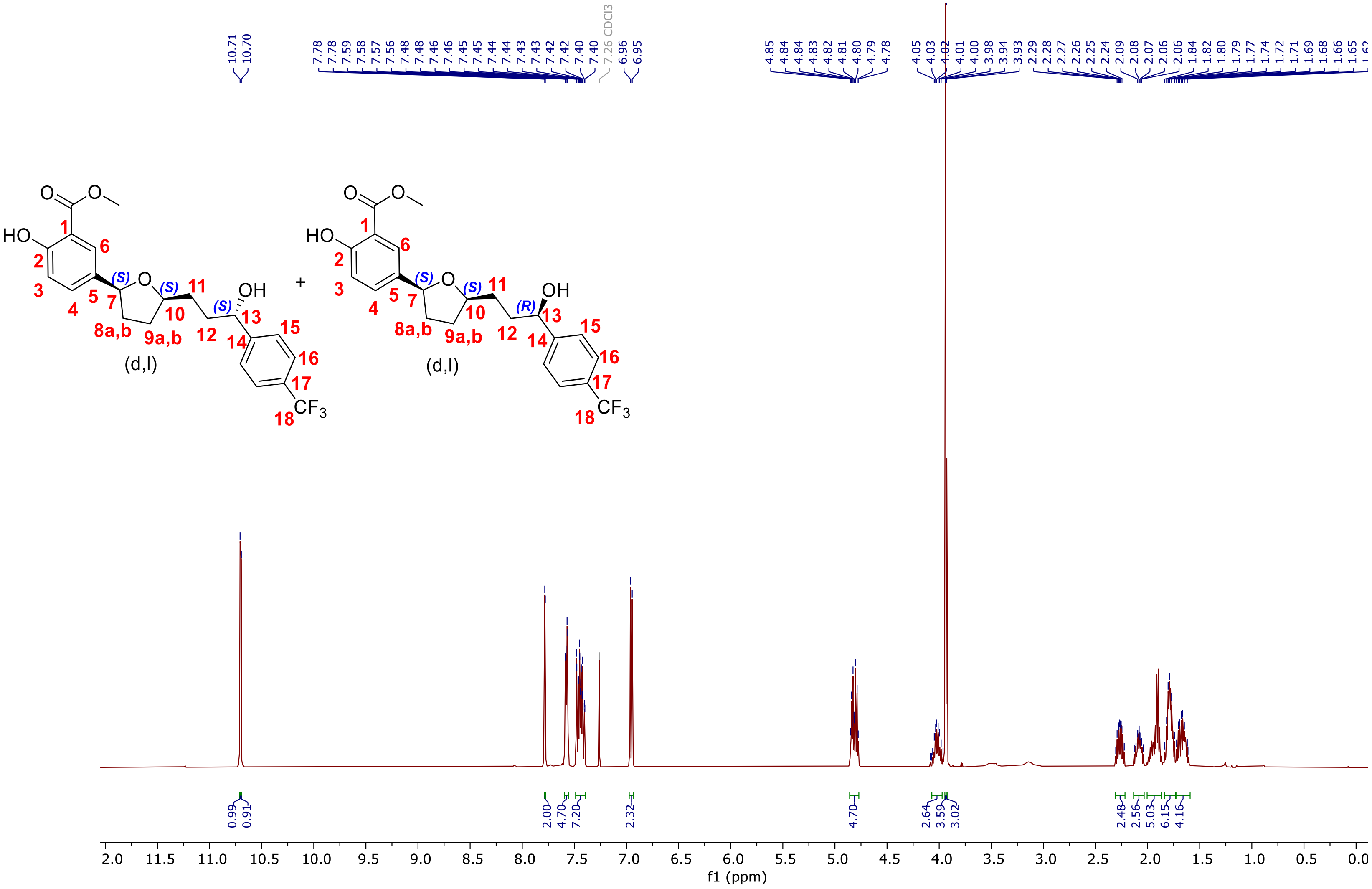

Methyl 2-hydroxy-5-{5-[3-hydroxy-3-(*p*-trifluoromethylphenyl)propyl]tetrahydrofuran-2-yl}benzoate (43)

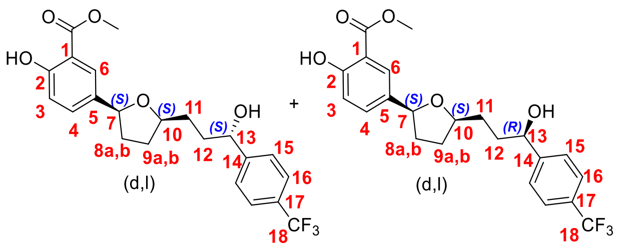

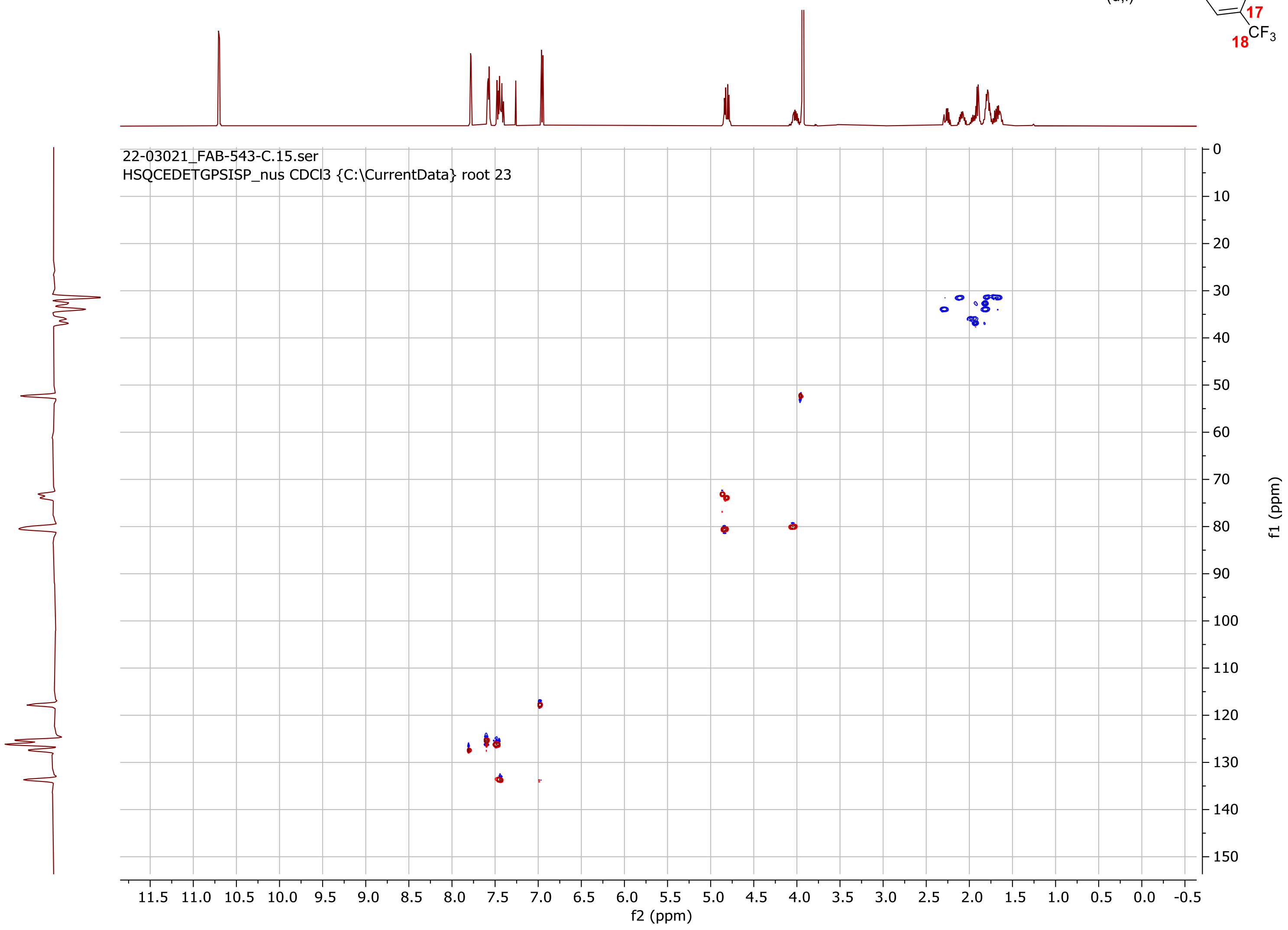

Methyl 2-hydroxy-5-{5-[3-hydroxy-3-(*p*-trifluoromethylphenyl)propyl]tetrahydrofuran-2-yl}benzoate (43)

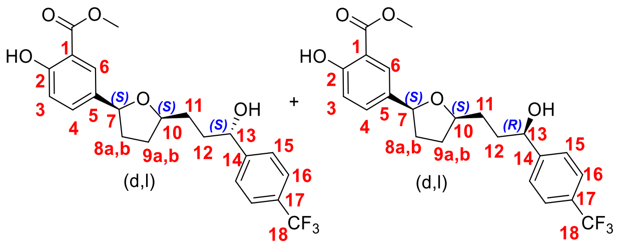

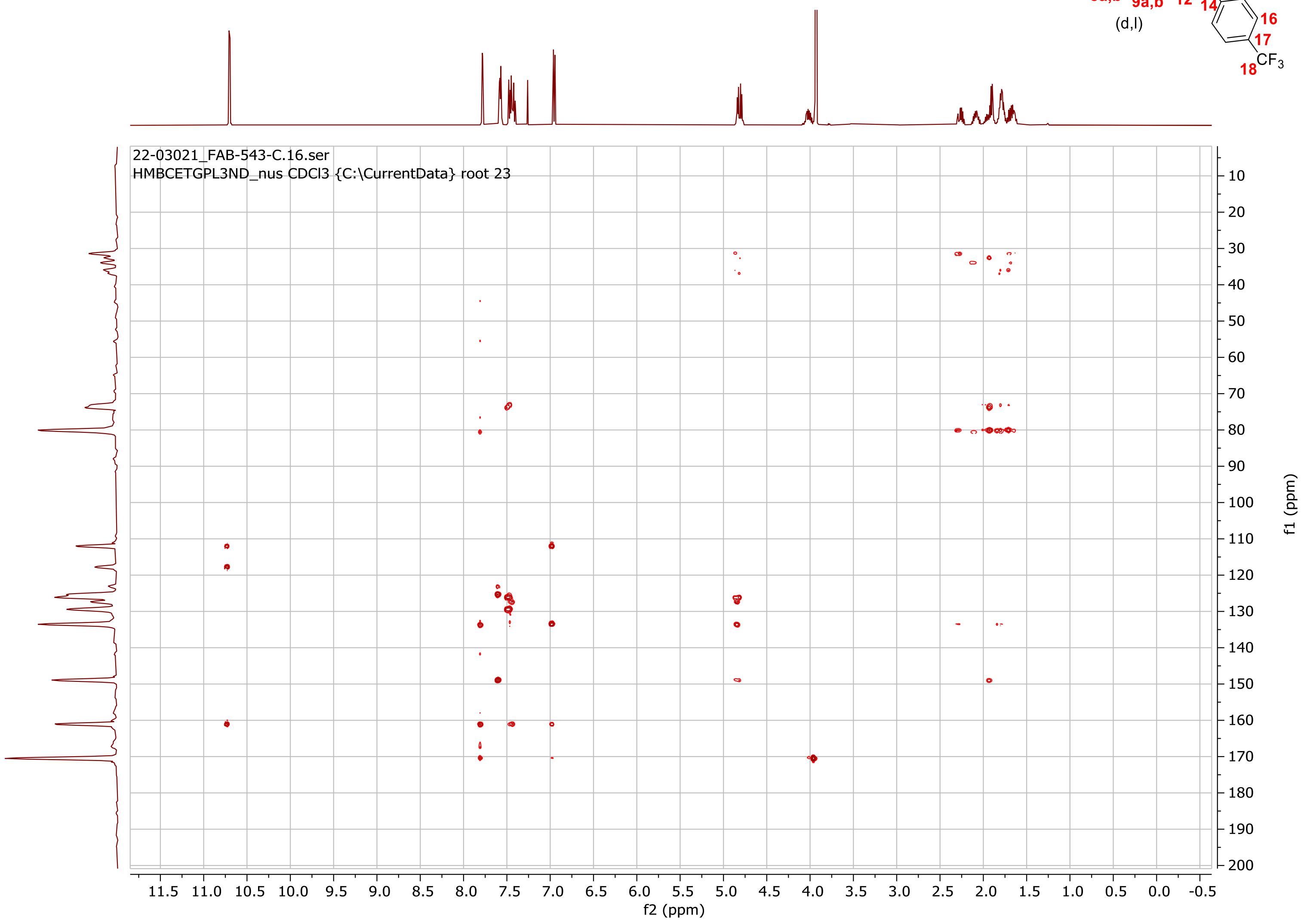

(*E*)-2-Hydroxy-5-[5-(4,4,4-trifluoro-3-oxobut-1-enyl)furan-2-yl]benzoic acid (S2)

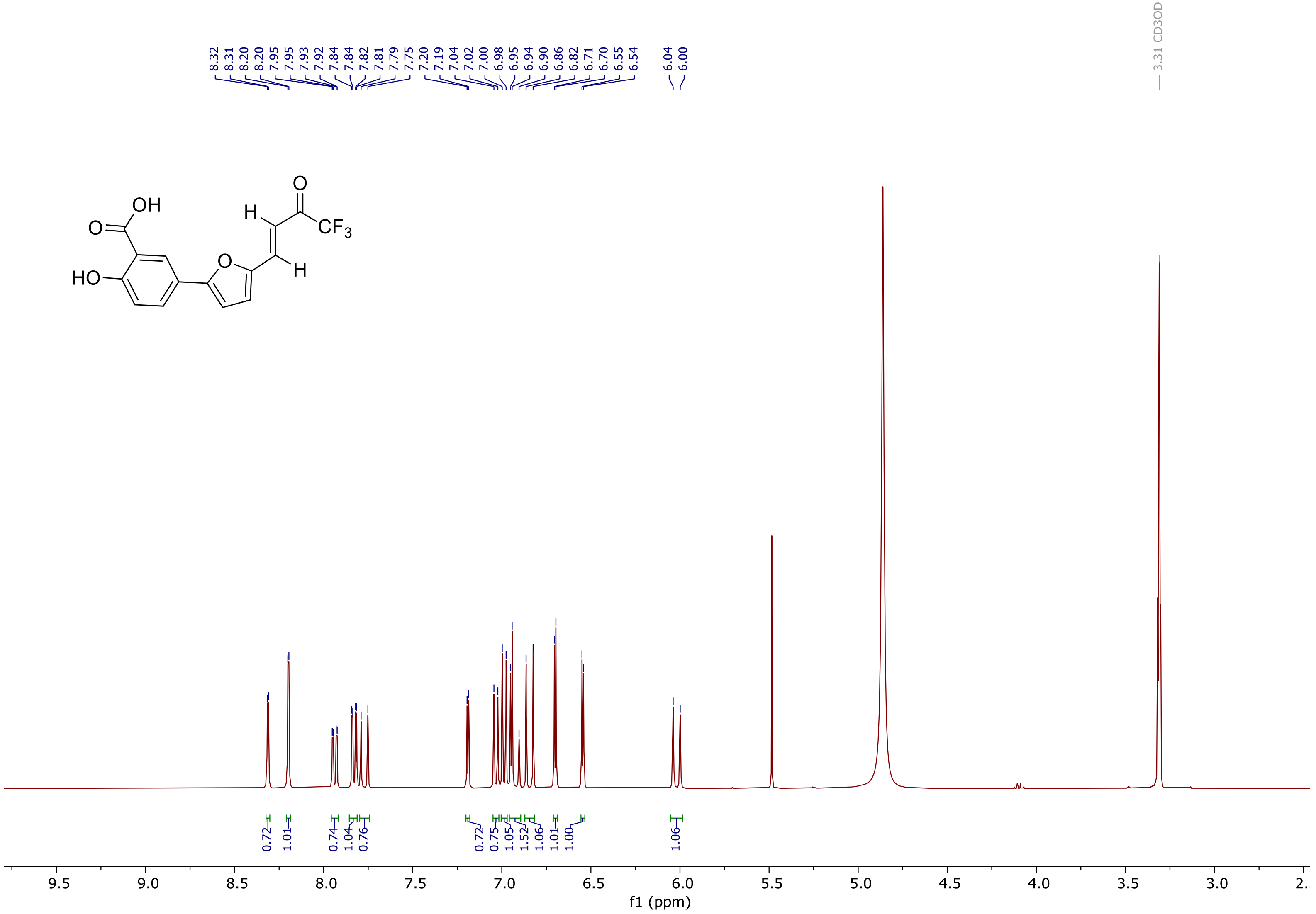

(*E*)-2-Hydroxy-5-[5-(4,4,4-trifluoro-3-oxobut-1-enyl)furan-2-yl]benzoic acid (S2)

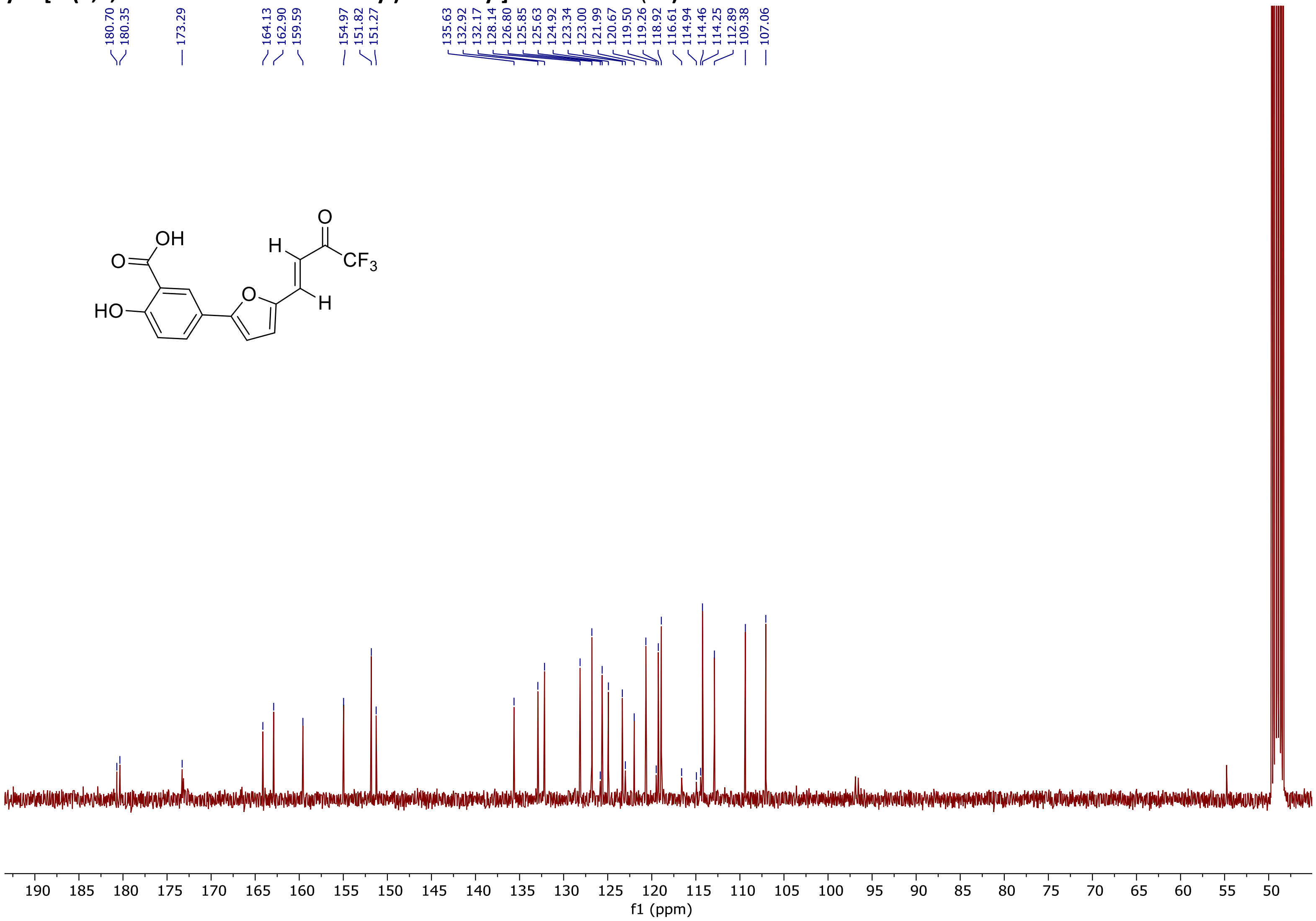

5-[5-(2-Acetyl-3-oxobut-1-enyl)furan-2-yl]-2-hydroxybenzoic acid (S3).

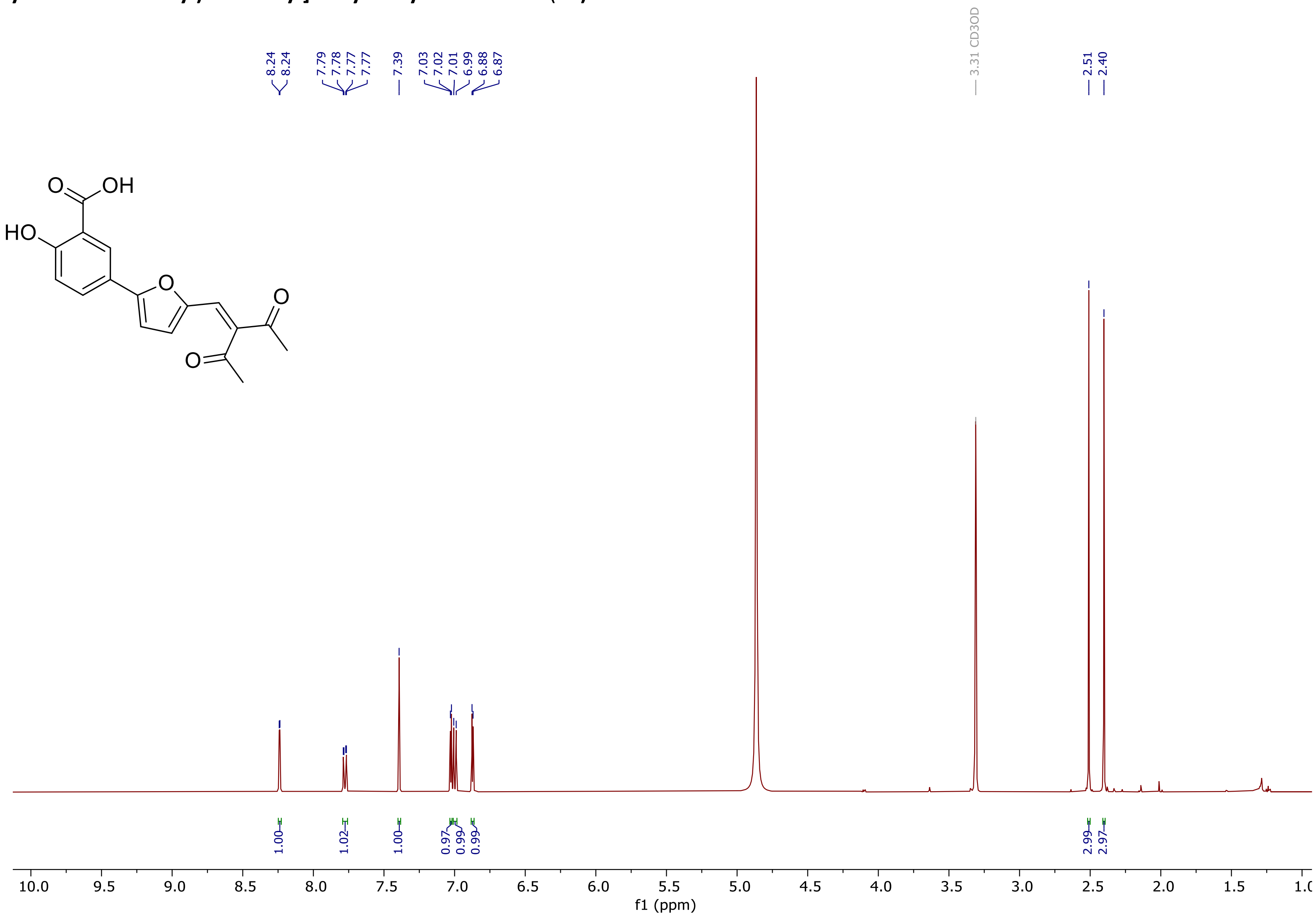

5-[5-(2-Acetyl-3-oxobut-1-enyl)furan-2-yl]-2-hydroxybenzoic acid (S3).

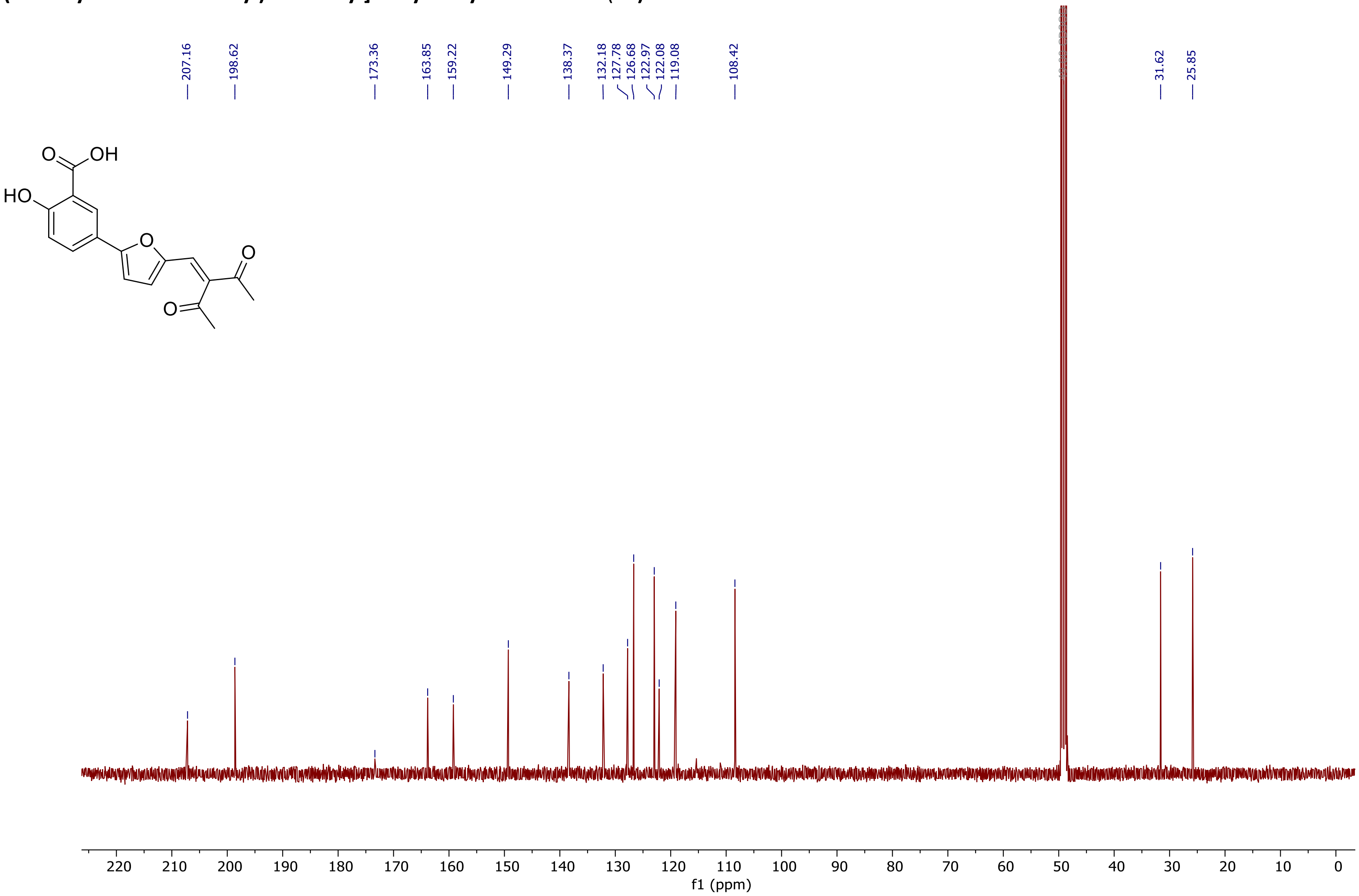

(*E*)-2-Hydroxy-5-{5-[3-(1-methylpyrrol-2-yl)-3-oxoprop-1-en-1-yl]furan-2-yl}benzoic acid (S4).

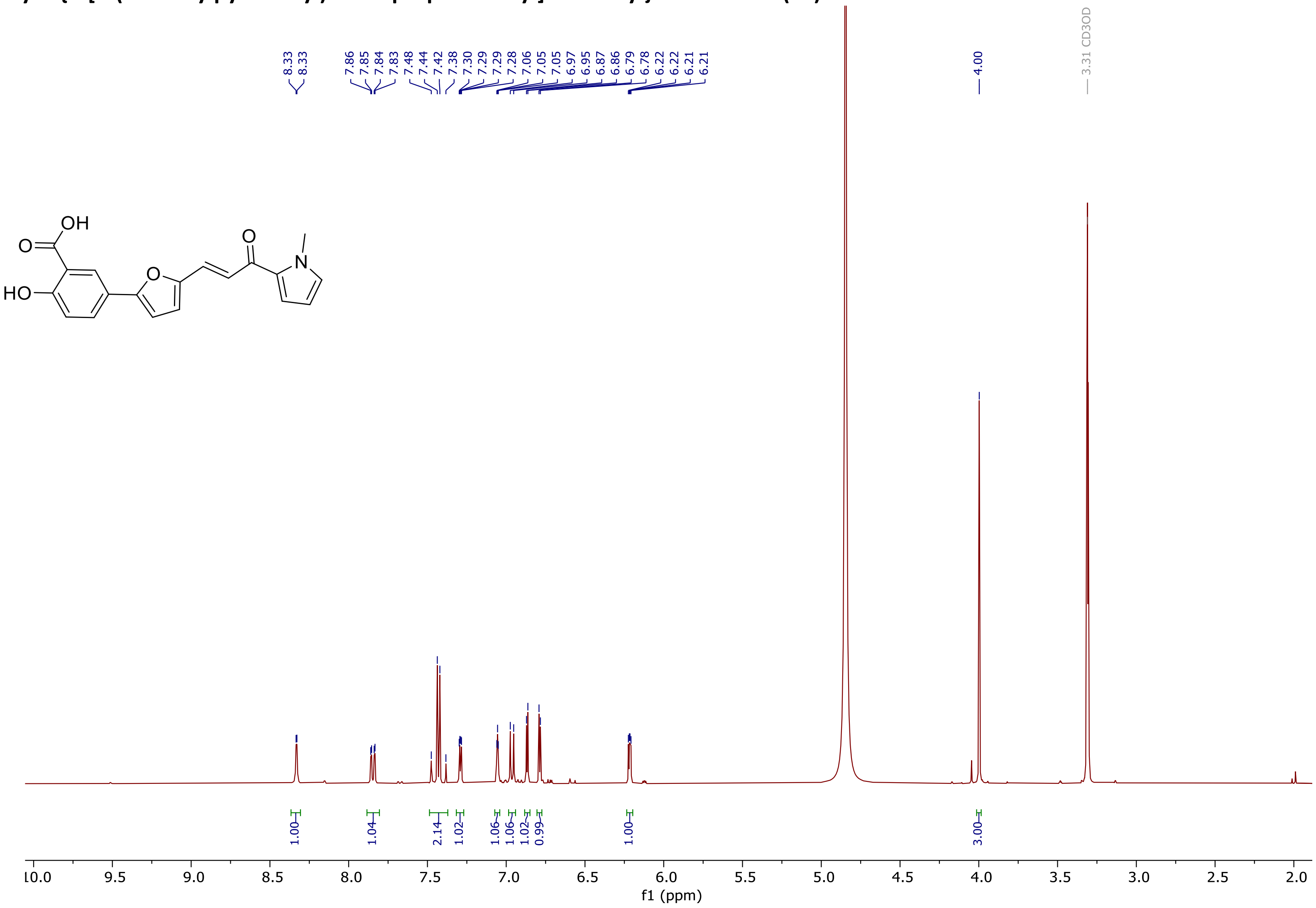

(*E*)-2-Hydroxy-5-{5-[3-(1-methylpyrrol-2-yl)-3-oxoprop-1-en-1-yl]furan-2-yl}benzoic acid (S4).

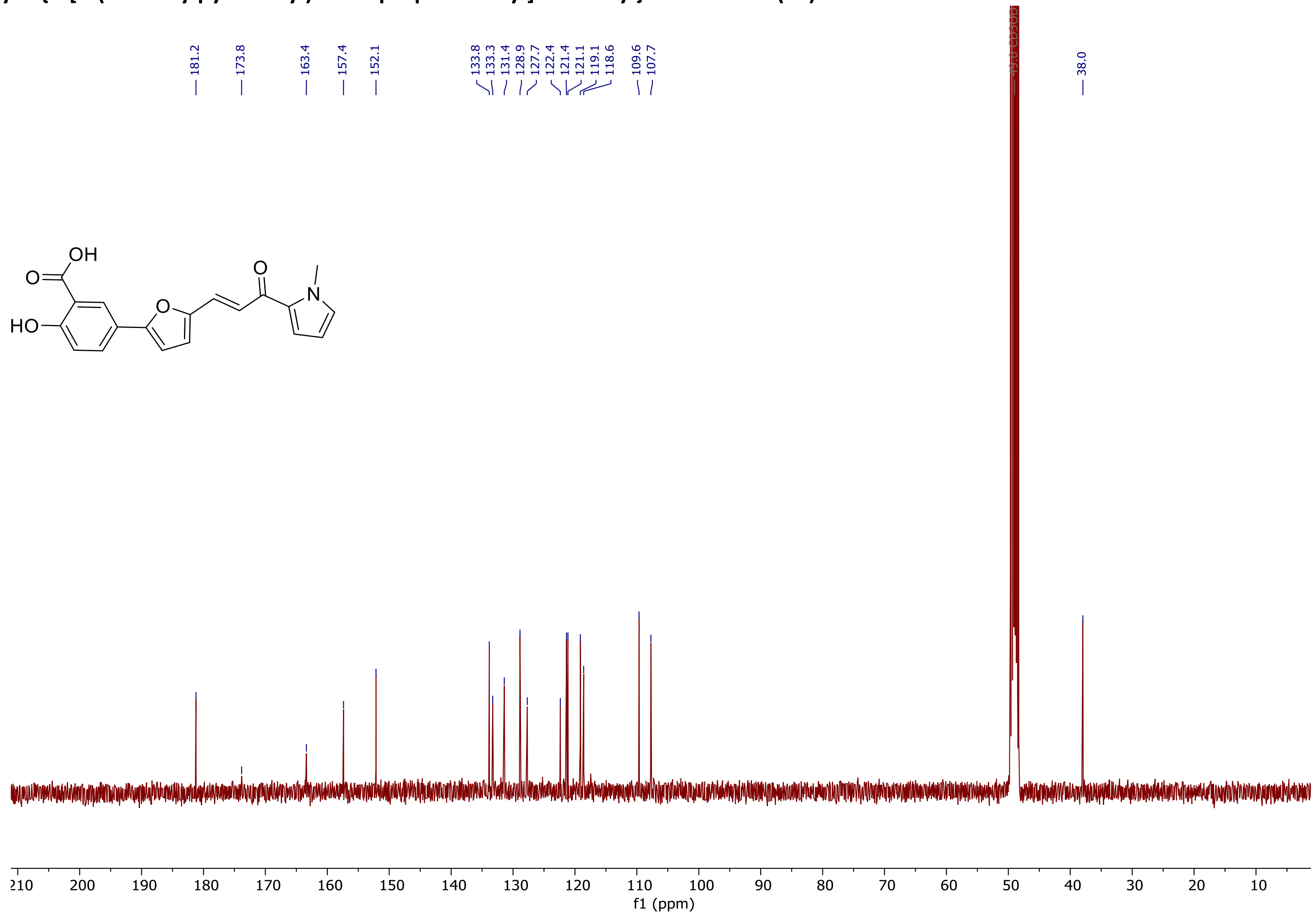

(*E*)-5-{5-[3-(furan-2-yl)-3-oxoprop-1-enyl]furan-2-yl}-2-hydroxybenzoic acid (S5).

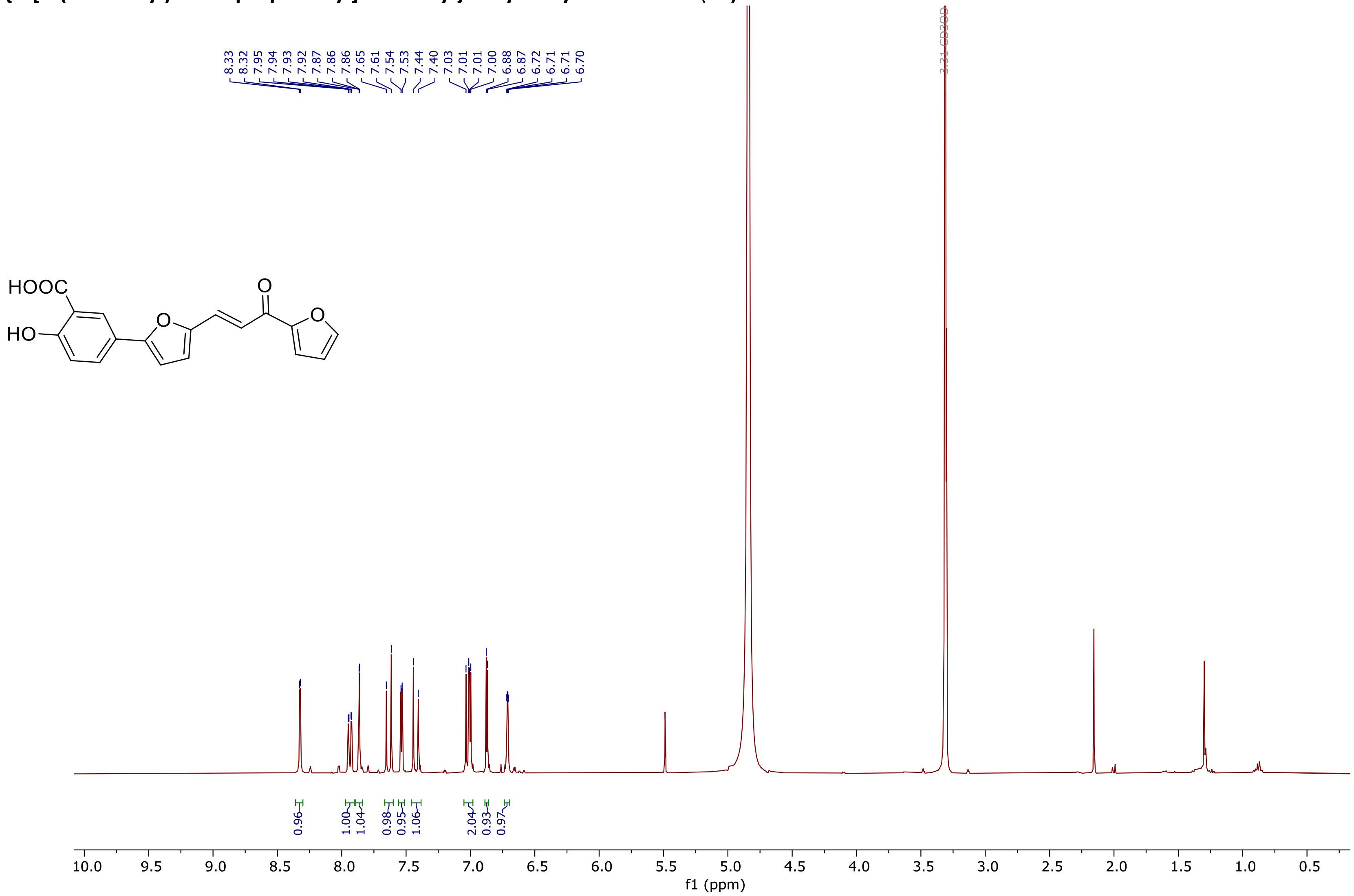

(*E*)-5-{5-[3-(furan-2-yl)-3-oxoprop-1-enyl]furan-2-yl}-2-hydroxybenzoic acid (S5).

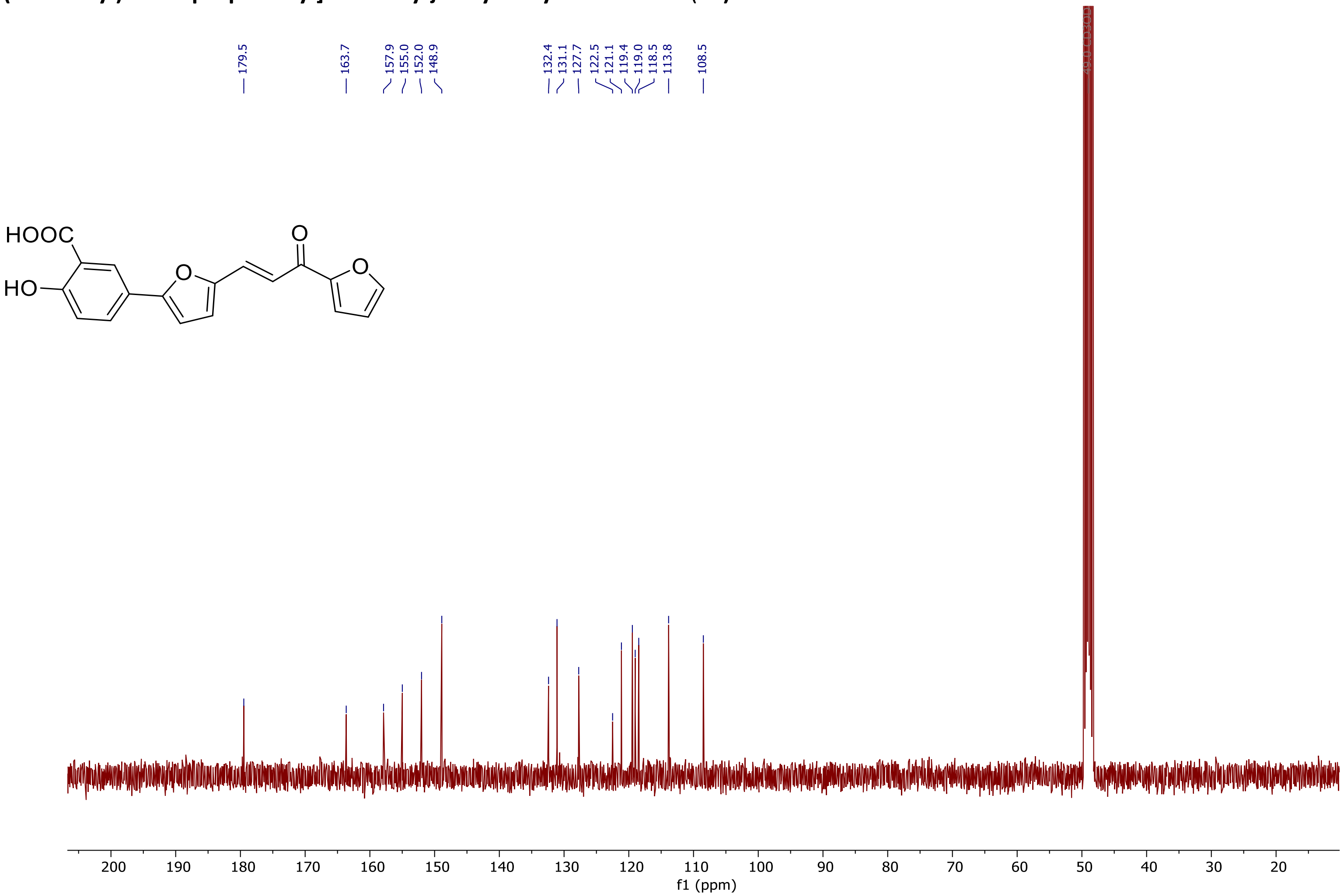

2-Hydroxy-5-{5-[3-hydroxy-3-(*p*-trifluoromethylphenyl)propyl]tetrahydrofuran-2-yl}benzoic acid (S6).

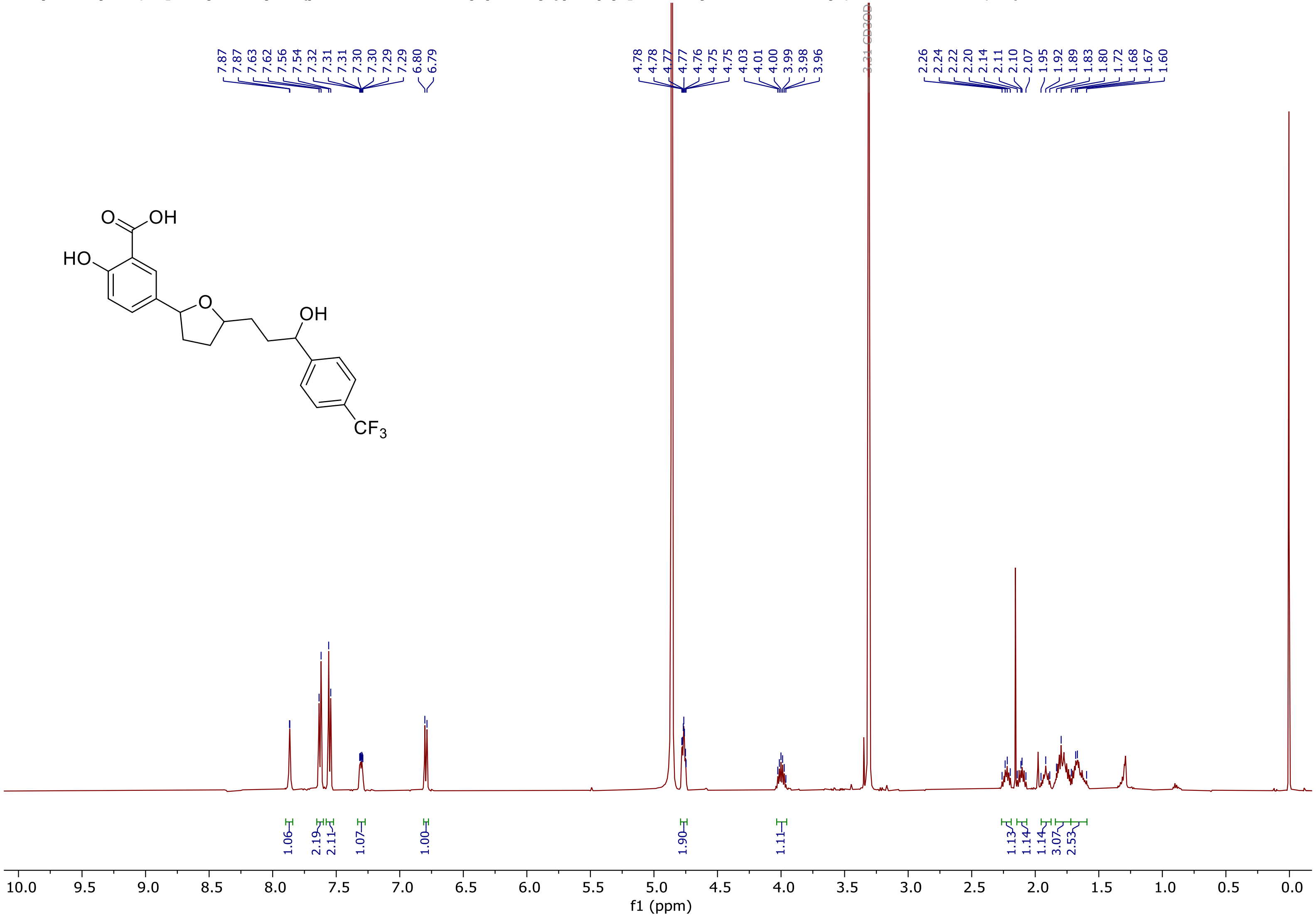

1D SELECTIVE NOESY

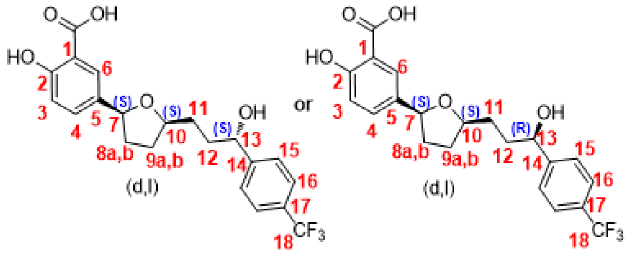

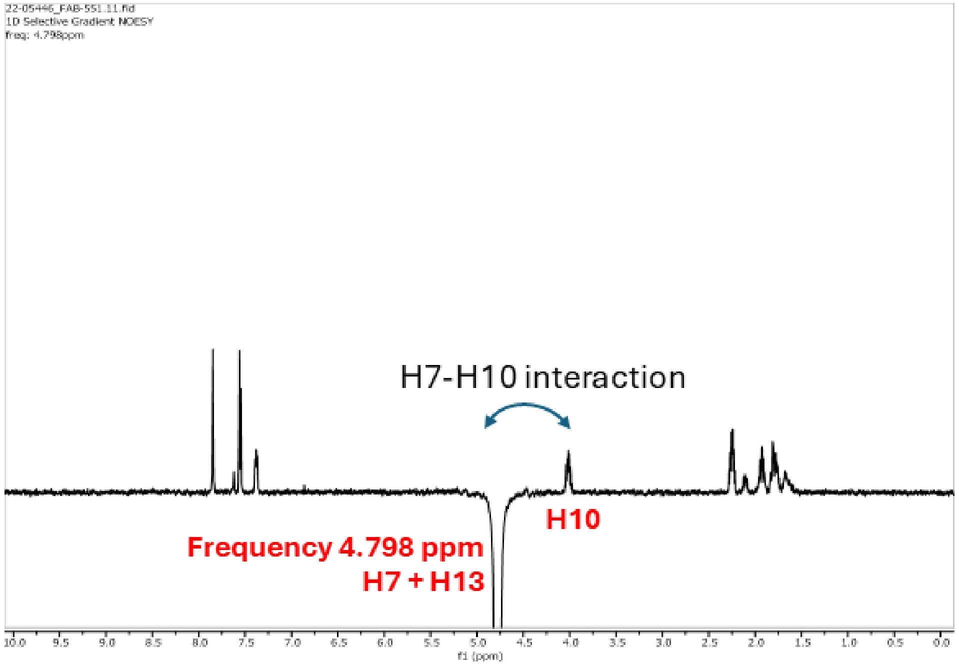

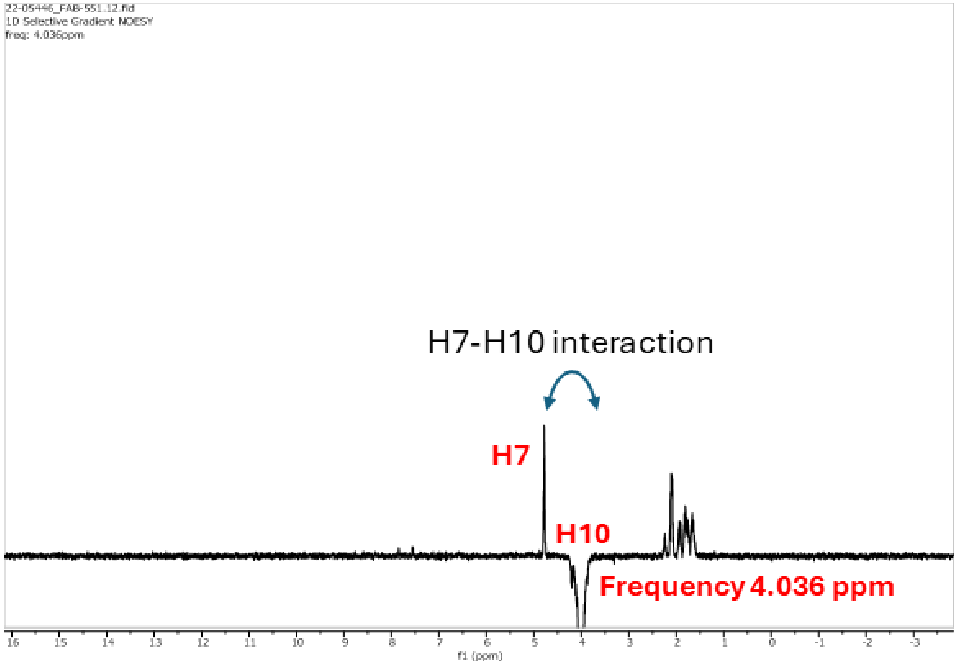

2-Hydroxy-5-{5-[3-hydroxy-3-(*p*-trifluoromethylphenyl)propyl]tetrahydrofuran-2-yl}benzoic acid (S6).

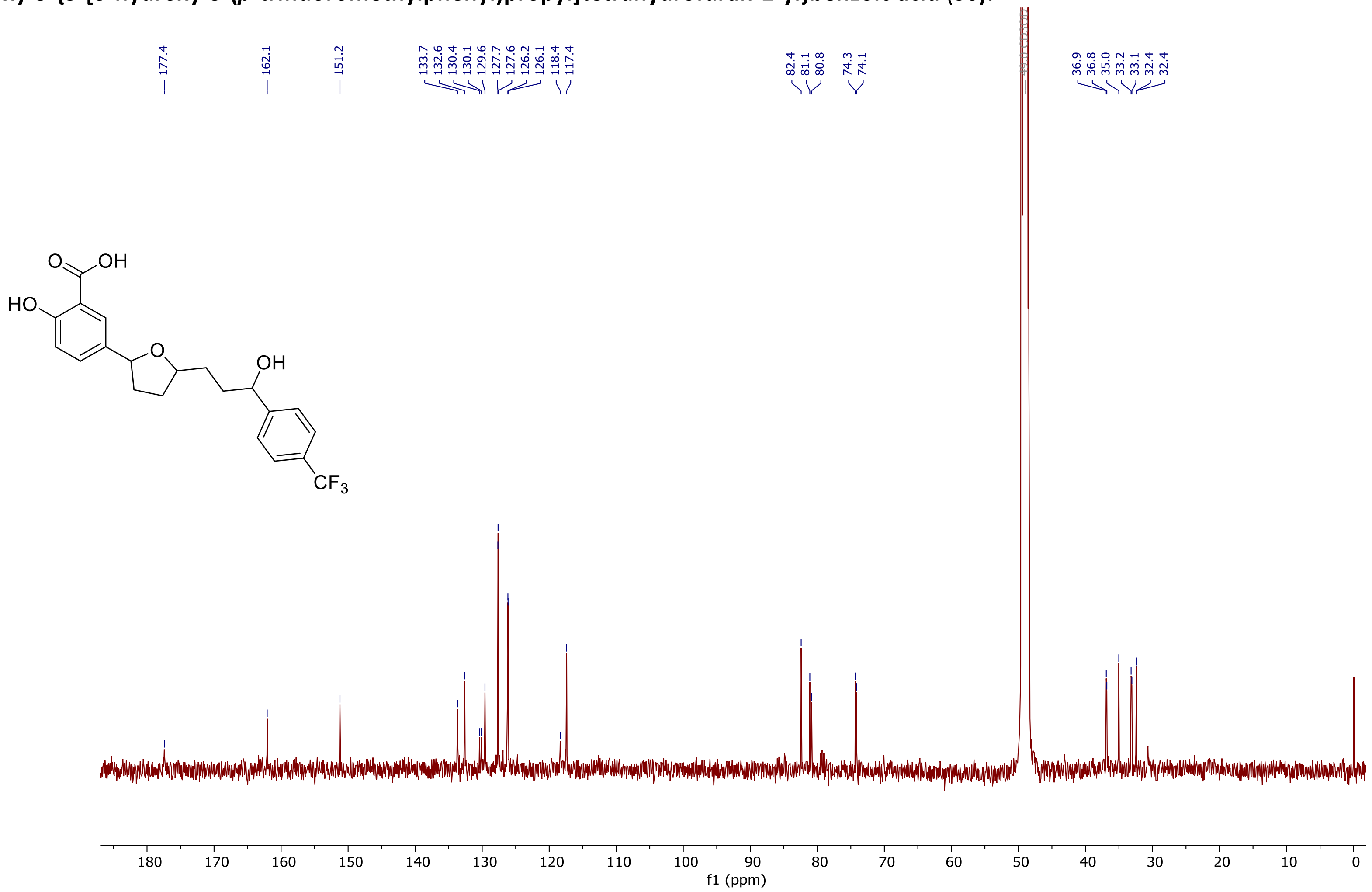

Supplement: Supplementary file 1 [file jm5c02055_si_001.pdf]
